# Enantioselective Synthesis of α‐Aryl Ketones by a Cobalt‐Catalyzed Semipinacol Rearrangement

**DOI:** 10.1002/anie.202414342

**Published:** 2024-11-06

**Authors:** Panagiotis G. Kalomenopoulos, Balakumar Emayavaramban, Craig P. Johnston

**Affiliations:** ^1^ EaStCHEM School of Chemistry University of St Andrews St Andrews Fife KY16 9ST UK

**Keywords:** Cobalt Catalysis, Semipinacol Rearrangement, Cations, Phenonium Ion, Enantioselective

## Abstract

A highly enantioselective cobalt‐catalyzed semipinacol rearrangement of symmetric α,α‐diarylallylic alcohols is disclosed. A chiral cobalt‐salen catalyst generates a highly electrophilic carbocation surrogate following hydrogen atom transfer and radical–polar crossover steps. This methodology provides access to enantioenriched α‐aryl ketones through invertive displacement of a cobalt(IV) complex during 1,2‐aryl migration. A combination of readily available reagents, silane and *N*‐fluoropyridinium oxidant, are used to confer this type of reactivity. An exploration into the effect of aryl substitution revealed the reaction tolerates *para‐* and *meta‐*halogenated, mildly electron‐rich and electron‐poor aromatic rings with excellent enantioselectivities and yields. The yield of the rearrangement diminished with highly electron‐rich aryl rings whereas very electron‐deficient and *ortho*‐substituted arenes led to poor enantiocontrol. A Hammett analysis demonstrated the migratory preference for electron‐rich aromatic rings, which is consistent with the intermediacy of a phenonium cation.

## Graphical abstract

A chiral cobalt‐salen catalyst has been used under mild, oxidative conditions to generate a cobalt(IV)‐bound intermediate that is sufficiently electrophilic to promote an enantioselective 1,2‐aryl migration. The excellent levels of stereocontrol and a Hammett analysis provide key evidence for the involvement of the cobalt catalyst in this cationic hydro‐semipinacol rearrangement.

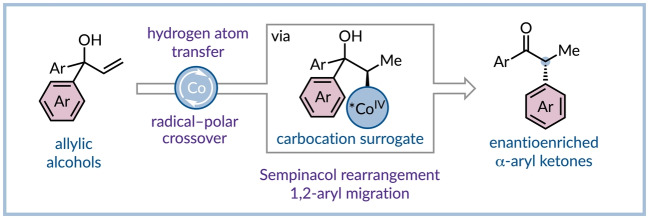

## Introduction

**Figure 1:**
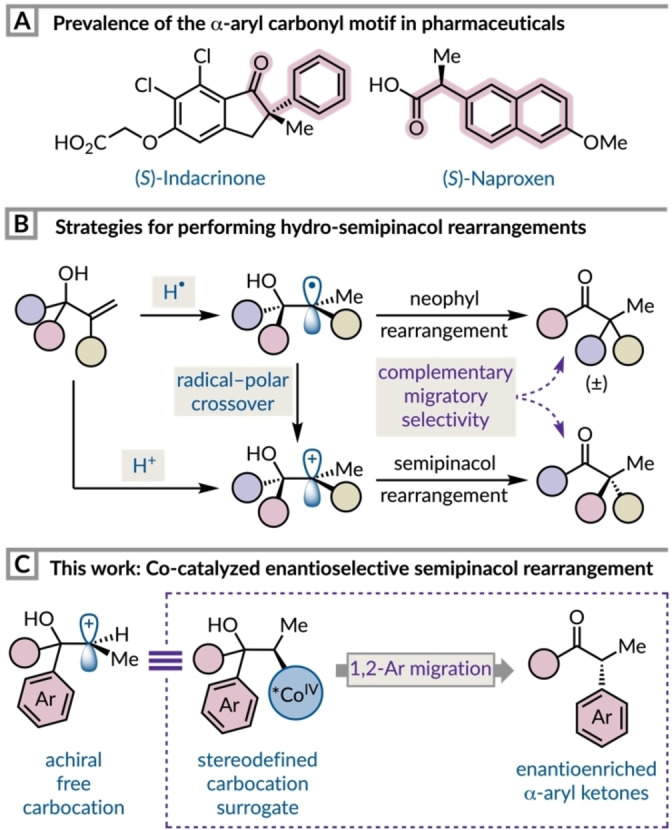
(a) Examples of drugs containing an α‐aryl carbonyl group. (b) Comparing radical and cationic approaches for instigating rearrangements with allylic alcohols. (c) Using alkylcobalt(IV) species as carbocation surrogates to perform enantioselective 1,2‐aryl shifts.

The efficient and selective synthesis of enantioenriched α‐aryl carbonyl compounds is important owing to their presence in marketed pharmaceuticals, bioactive compounds, and natural products (Figure 1A). The conventional approach to this motif involves direct α‐arylation techniques using transition metal catalysts and base‐mediated enolate chemistry.[^1^, ^2^] This strategy for accessing α‐aryl carbonyl compounds can pose limitations when synthesizing tertiary stereocenters as they are base sensitive and prone to racemization by enolization.^[3]^ Therefore, the synthesis of enantiopure tertiary α‐aryl ketones is synthetically challenging and requires procedures that will eliminate the need for an interfering strong base.[^4^, ^5^, ^6^, ^7^, ^8^] The semipinacol rearrangement, when involving a 1,2‐aryl migration, can be harnessed to synthesize α‐aryl carbonyl compounds and is a viable alternative to enolate arylation chemistry. It has been shown that the addition of various radicals to the olefin of an allylic alcohol can produce racemic α‐aryl ketones via a radical 1,2‐aryl migration mechanism.[^9^, ^10^, ^11^] In a few cases, it has been shown that a single electron transfer (SET) event can occur to generate a carbocation before aryl migration ensues.[^12^, ^13^]

The few enantioselective semipinacol rearrangement protocols that involve aryl migration involve enantiospecific nucleophilic displacement of an enantioenriched halonium,[^14^, ^15^] or hypervalent iodine species^[16]^ and create β‐keto functionalised products.^[10]^ Related polar rearrangements involving 1,2‐aryl shifts have employed chiral Brønsted acids, which instead involve non‐covalent interactions between the substrate and catalyst.[^17^, ^18^, ^19^] In the context of hydro‐semipinacol rearrangements with allylic alcohols, the addition of a proton or hydrogen atom to the C=C bond can be used to instigate reactivity (Figure 1B). The latter has been demonstrated via metal‐hydride hydrogen atom transfer (MHAT) using a superstoichiometric quantity of Fe(acac)_3_ with a silane,^[20]^ or photocatalytically using a cobalt catalyst.^[21]^ The carbon‐centered radical generated after the HAT step undergoes a neophyl‐type rearrangement to afford racemic ketones with electron‐deficient aryl rings migrating preferentially. Rendering this process enantioselective is intrinsically challenging although a modest degree of stereoretention has been demonstrated with enantioenriched allylic alcohols in the cobalt system.^[21]^ Alternatively, direct protonation of the olefin generates a carbocation intermediate, which in the context of aryl rearrangement leads to selective migration of the most electron‐rich arene. This polar approach has enabled the development of catalytic enantioselective hydro‐semipinacol rearrangements most commonly using Brønsted acid catalysis.[^22^, ^23^, ^24^, ^25^] These methodologies all feature aliphatic carbon migrations to heteroatom‐stabilized or tertiary carbocations leading to ring‐expanded products. Therefore, secondary carbocations and 1,2‐aryl shifts have not been realized within this framework. In fact, we are aware of only one reported example where a 1,2‐aryl migration occurs in a polar hydro‐semipinacol rearrangement with allylic alcohols.[^26^, ^27^] This scarcity may be related to the incompatibility of α‐vinylbenzyl alcohols with the strongly acidic conditions typically required to facilitate such rearrangements.^[28]^

One approach that combines the milder reaction conditions of MHAT with the migratory tendencies of cationic intermediates invokes a radical–polar crossover process.^[29]^ This tactic has been deployed extensively in cobalt‐catalyzed olefin hydrofunctionalizations to construct C−O, C−N, C−S, and C−C bonds as racemic mixtures.[^26^, ^30^, ^31^, ^32^, ^33^, ^34^, ^35^] Recently, photochemical and electrochemical procedures that negate the requirement for a stoichiometric oxidant in this manifold have been disclosed.[^36^, ^37^, ^38^, ^39^, ^40^, ^41^, ^42^] The species responsible for certain instances of the polar reactivity observed in these oxidative cobalt‐catalyzed MHAT reactions was proposed by Pronin and co‐workers to be an alkylcobalt(IV) intermediate,^[26]^ which was concurrently implicated in the transmetalation step of a process that merged Co MHAT and nickel catalysis.^[43]^ The involvement of a cobalt(IV) intermediate, capable of undergoing stereospecific displacement by nucleophiles, presents an ideal opportunity for asymmetric induction. However, this mode of reactivity has only been harnessed for highly enantioselective transformations in five previous reports by Pronin, Shigehisa, and Zhang.[^44^, ^45^, ^46^, ^47^, ^48^, ^49^] In particular, the cobalt‐catalyzed radical–polar crossover reactions of allylic alcohols were previously investigated by Pronin and co‐workers which focused on the reactivity profile of aliphatic systems.^[26]^ A competition between hydroalkoxylation and semipinacol rearrangement pathways was controlled by the choice of cobalt‐salen complex. Further work demonstrated that a chiral catalyst with extended aromatic systems could deliver epoxide products with high enantioselectivity. Conversely, substrates that underwent the semipinacol rearrangement with 1,2‐alkyl migration showed low levels of enantiocontrol.^[44]^ Here, we demonstrate that an alkylcobalt(IV) species generated from an allylic alcohol can behave as a stereodefined surrogate for a secondary carbocation (Figure 1C). The subsequent asymmetric semipinacol rearrangement proceeds via 1,2‐aryl migration to give ketones bearing tertiary arylated stereocenters.

## Results and Discussion

Previously we have shown that this approach is viable for conducting Wagner–Meerwein rearrangements that involve a 1,2‐aryl migration.^[50]^ These studies unveiled a phenonium ion as a probable intermediate with subsequent regioselective nucleophilic fluorination finalizing the relocation process. Thus, given this reactivity, we postulated that a hydro‐semipinacol rearrangement involving a 1,2‐aryl shift was viable. The *p*‐fluoro substituted substrate **1 a** was chosen for optimization due to the ease of quantitative analysis by ^19^F NMR spectroscopy. During these preliminary investigations, it was revealed that the yield of product (*R*)‐**2 a** was dependent on the amount of air in the reaction vessel and significant quantities of 4,4’‐difluorobenzophenone were identified when the flask was not properly inerted. However, the presence of air did not impact enantioselectivity and this byproduct likely arises from the interception of the carbon‐centered radical generated after the MHAT step by molecular oxygen.^[51]^ Based on our prior work we conducted a cobalt catalyst screen using *N*‐fluoro‐2,4,6‐trimethylpyridinium tetrafluoroborate (Me_3_NFPY⋅BF_4_) as an oxidant and 1,1,3,3‐tetramethyldisiloxane as the hydrogen atom source in chlorobenzene (Table 1). The commercially available cobalt catalyst (*R*,*R*)‐**Co(II)**‐**1**, built from optically pure *trans*‐1,2‐diaminocyclohexane, afforded the desired product **2 a** albeit with a low enantiomeric ratio (Table 1, entry 1). Switching to (*R*,*R*)‐**Co(II)**‐**2** with a 1,2‐diphenyl ethane backbone led to an improved yield and enantioselectivity (Table 1, entry 2). Given the ease of modifying the 1,2‐diphenyl motif, we then explored alternative salen ligands of this type. From this, it was clear that increasing the steric bulk of the aromatic rings was more beneficial than introducing electron‐donating groups (Table 1, entries 3–5). Ultimately, 1,2‐bis(mesityl)‐containing catalyst, (*R*,*R*)‐**Co(II)**‐**5**, was superior affording the ketone (*R*)‐**2 a** in 95 % yield with 99 : 1 er. We also examined conducting the reaction at room temperature rather than at 0 °C, but a slight decrease in enantioselectivity was observed (Table 1, entry 6). Lowering the catalyst loading to 3 mol % or increasing the reaction concentration to 0.2 M were both detrimental to yield within the same two‐hour timeframe (Table 1, entries 7–8). Exploring a range of alternative solvents with (*R*,*R*)‐**Co(II)**‐**5** demonstrated the importance of the reaction medium with large variations in efficiency and levels of asymmetric induction (Table 1, entries 9–12).

**Table 1:** Optimization of the enantioselective semipinacol rearrangement.

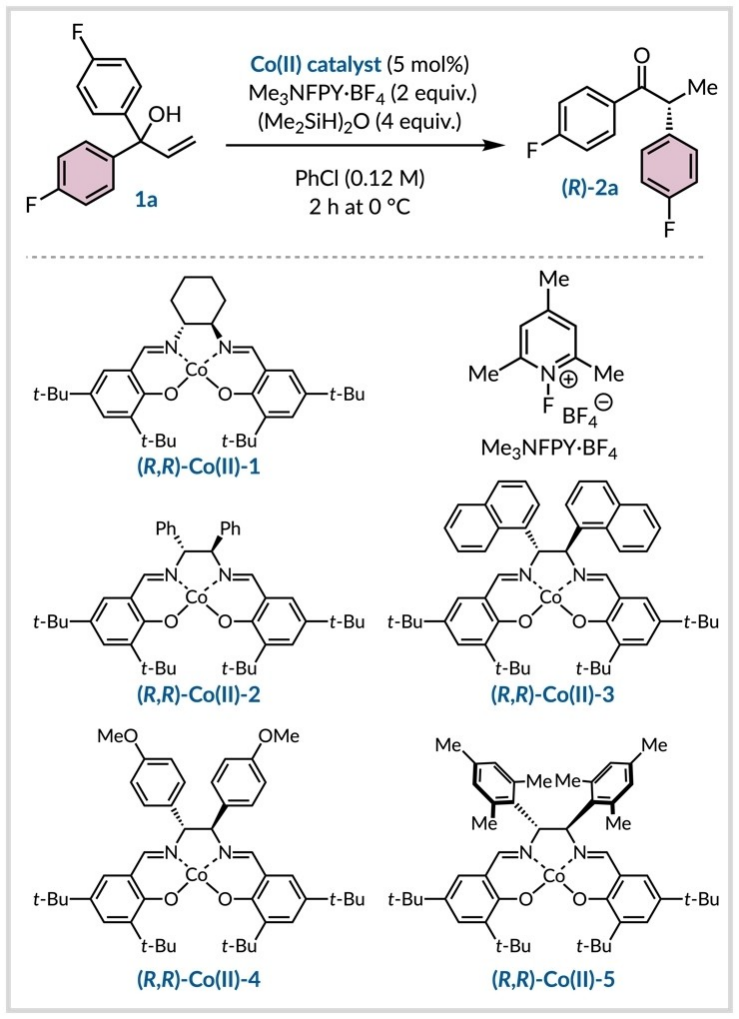				
Entry^[a]^	Catalyst	Deviations	**2 a** [%]^[b]^	er^[c]^
1	**Co(II)‐1**	–	56	58 : 42
2	**Co(II)‐2**	–	85	70 : 30
3	**Co(II)‐3**	–	83	88 : 12
4	**Co(II)‐4**	–	93	66 : 34
5	**Co(II)‐5**	–	95	99 : 1
6	**Co(II)‐5**	RT	90	95 : 5
7	**Co(II)‐5**	3 mol % **Co‐5**	77	96 : 4
8	**Co(II)‐5**	0.2 M	81	98 : 2
9	**Co(II)‐5**	acetone	42	69 : 31
10	**Co(II)‐5**	EtOAc	55	89 : 11
11	**Co(II)‐5**	CH_2_Cl_2_	67	77 : 23
12	**Co(II)‐5**	PhMe	0	–

[a] Unless otherwise stated optimization reactions were performed with **1 a** (0.10 mmol), Me_3_NFPY⋅BF_4_ (2.0 equiv.), silane (4.0 equiv.) for 2 h at 0 °C in PhCl (0.12 M). [b] Yield determined by ^19^F NMR analysis of the crude reaction mixture with PhF (1.0 equiv.) as internal standard. [c] er [(*R*) : (*S*)] determined by chiral HPLC. See Supporting Information for further details on optimization.

**Scheme 1:**
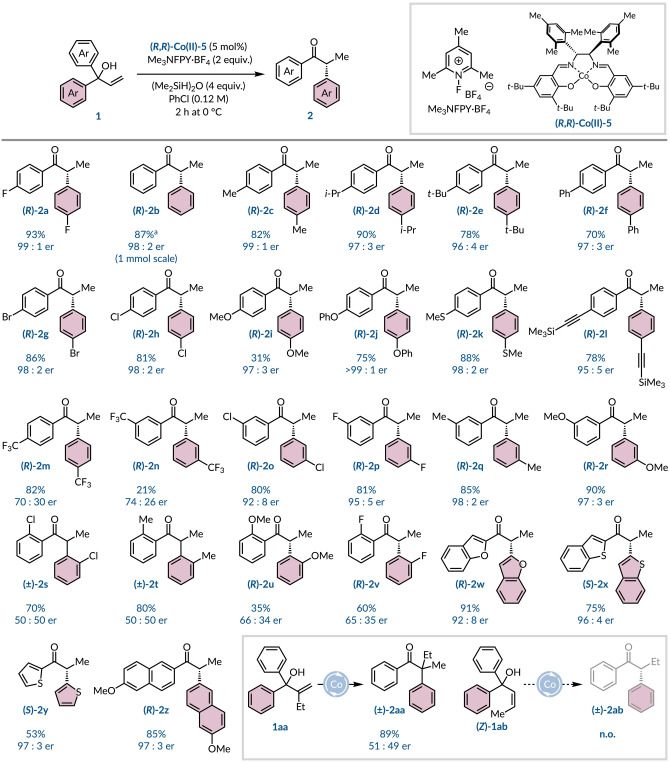
Substrate scope for enantioselective semipinacol rearrangement. Reactions performed with 0.20 mmol of alkene, **(*R***, ***R*** **)‐Co(II)‐5** (5 mol %), Me_3_NFPY⋅BF_4_ (2.0 equiv.), and silane (4.0 equiv.) in PhCl (0.12 M) for 2 h at 0 °C. Yields refer to isolated products. er determined by chiral HPLC.^a^Reaction run on a 1 mmol scale.

After identifying the optimal reaction conditions for conducting the hydro‐semipinacol rearrangement of **1 a** enantioselectively we evaluated these conditions with a series of symmetric α,α‐diarylallylic alcohols (Scheme 1). The simplest substrate **1 b** with unsubstituted arenes delivered product (*R*)‐**2 b** in 87 % yield and 98 : 2 er on a 1 mmol scale. The absolute (*R*)‐configuration within ketone **2 b** was determined by comparing its specific rotation to a literature value,^[52]^ and the remaining product configurations have been assigned by analogy. A range of alkyl substituents in the *para*‐position, some bearing benzylic hydrogens, and a 4‐phenyl analogue all gave comparable results with products, (*R*)‐**2 c**–**2 f**, obtained in 70–90 % yield with high levels of enantioenrichment. The reaction accommodated the presence of bromine (**2 g**) or chlorine (**2 h**) atoms and provides a synthetic handle for further manipulations. Highly electron‐donating substrates were less well tolerated, affording a lower yield of 31 % for the *p*‐methoxy product (*R*)‐**2 i** with significant formation of a 4,4’‐dimethoxybenzophenone byproduct. Despite attempts to thoroughly purge the reaction vessel with inert gas we could not overcome this deleterious side reaction; nevertheless, high levels of enantioselectivity were maintained. Tempering the electron density of the substrate by using a phenyl ether (**2 j**) restored the yields to synthetically useful levels. A thioether (**2 k**) and TMS‐protected alkyne (**2 l**) were compatible under the oxidative reaction conditions with the products having 98 : 2 er and 95 : 5 er, respectively. An evident drop in enantioselectivity was noted by introducing the strongly electron‐withdrawing trifluoromethyl group either at the *para*‐ (**2 m**) or *meta*‐ (**2 n**) position of the aromatic ring. Although, at least for example **2 m** demonstrates the alkylcobalt(IV) intermediate is sufficiently electrophilic to promote 1,2‐migration of a severely electron‐deficient arene. Relatively less electron‐deficient derivatives, such as *m*‐chloro (**2 o**) or *m*‐fluoro (**2 p**) substituents, were better tolerated and gave the corresponding α‐arylated ketones with high levels of enantiopurity. Alternate *meta*‐substituents, including methyl (**2 q**) and methoxy (**2 r**) groups, were also highly amenable to the rearrangement protocol. The presence of an *ortho*‐substituent did not drastically hamper reactivity, but it did significantly affect the enantioselectivity. For example, *o*‐chloro (**2 s**) or *o*‐methyl (**2 t**) groups returned the products in good yield (70–80 %) but as racemic mixtures. Even a small perturbation at this site had a substantial impact as the *o*‐fluoro product (**2 v**) was obtained in 65 : 35 er, which is a notable difference when compared to the *para*‐ and *meta*‐analogues (**2 a** and **2 p**, respectively). We postulate that the presence of an *ortho*‐group might encourage the reaction to proceed via a free carbocation or radical intermediate, facilitated by the heterolytic or homolytic cleavage of the cobalt–carbon bond.[^21^, ^26^] The asymmetric 1,2‐migration process was also amenable to heteroaromatic rings with benzofuran (**2 w**), benzothiophene (**2 x**), and thiophene (**2 y**) all giving commendable levels of enantioenrichment. An electron‐rich naphthyl ring system worked well to afford the product (*R*)‐**2 z** in 85 % yield and in 97 : 3 er. In addition to terminal olefins, employing a 1,1‐disubstituted alkene (**1 aa**) to create a quaternary stereocenter yielded the anticipated product **2 aa** as a racemate, presumably through the intermediacy of a free carbocation or radical species. Lastly, we investigated a challenging 1,2‐disubstituted (*Z*)‐alkene (**1 ab**), however, only partial isomerization of the C=C bond was observed *(Z : E*=99 : 1→93 : 7).

**Figure 2:**
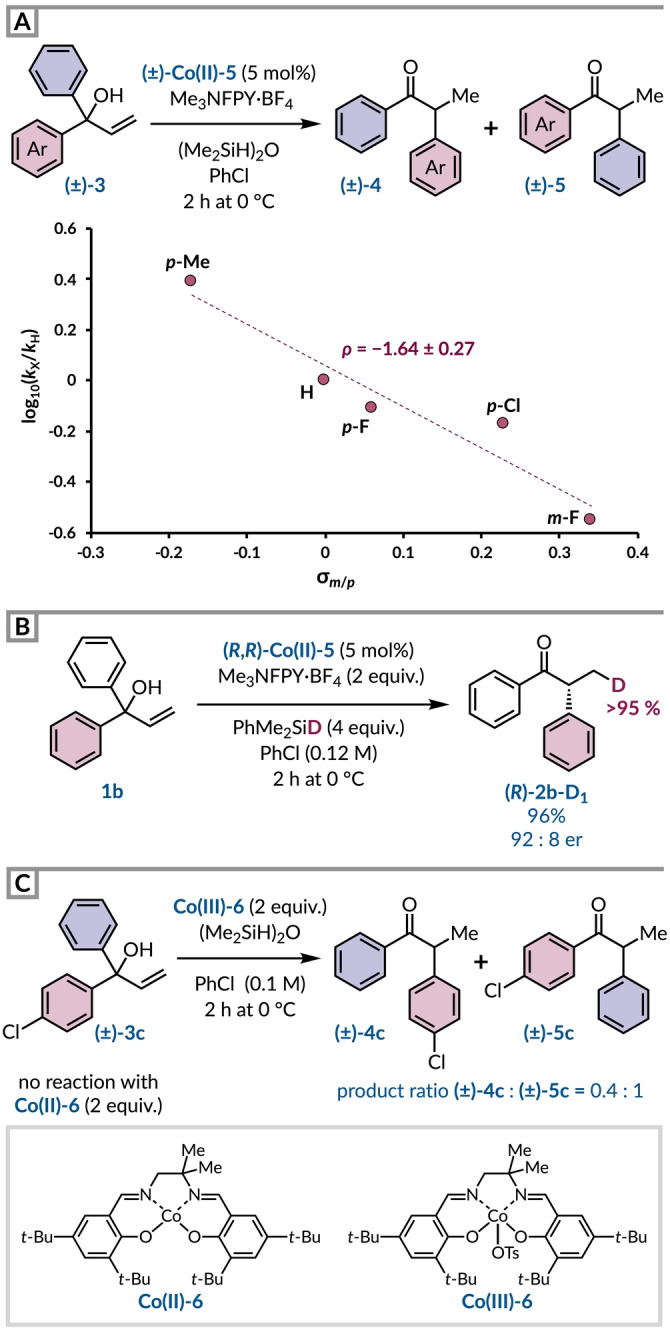
(a) Hammett analysis of the Co‐catalyzed semipinacol rearrangement. (b) Deuterium labelling experiment (c) Evidence for a Co(III) species acting as an oxidant for the alkylCo(III) intermediate.

The use of asymmetric α,α‐diarylallylic alcohols presented a unique opportunity to ascertain the migratory preference for electronically distinct aromatic rings in this Co‐catalyzed hydro‐semipinacol rearrangement. To do this, we performed the cobalt‐catalyzed transformation using racemic catalyst **Co(II)**‐**5** on a set of racemic substrates (±)‐**3** with one phenyl ring unsubstituted and systemically varying the electronic character of the other aryl ring (Figure 2A). Examination of the relative isomeric product ratios [(±)‐**4** : (±)‐**5**] by analysis of the crude reaction mixture by NMR spectroscopy revealed electron‐rich arenes migrated preferentially. Indeed, the inclusion of this data in a Hammett plot gave a good linear correlation (R^2^=0.93) with σ values to give a *ρ* value of −1.64. The sign and magnitude of the *ρ* value suggests that the 1,2‐migration occurs via a phenonium ion intermediate,[^48^, ^53^] and provides further evidence of the cationic behaviour of organocobalt(IV) intermediates.^[54]^ Interestingly, a substrate bearing a *p*‐CF_3_ group was a clear outlier in this analysis, with a higher proportion of the trifluoromethylated arene undergoing migration. This observation can be rationalized by contributions from a migration pathway proceeding via a radical species (for details see the Supporting Information). This outcome may explain why products (*R*)‐**2 m** and (*R*)‐**2 n** exhibited lower levels of enantioselectivity. Having established the rearrangement's polar behaviour, we then conducted a deuterium labelling experiment using PhMe_2_SiD instead of 1,1,3,3‐tetramethyldisiloxane (Figure 2B). The exclusive incorporation of deuterium at the olefin terminus is consistent with an MHAT mechanism and demonstrates that the silane is the source of the hydrogen atom in the product. Next, we wanted to determine the oxidant responsible for the radical–polar crossover event that generates the alkylcobalt(IV) intermediate. A control reaction verified that a stoichiometric quantity of **Co(II)**‐**6** in the absence of *N*‐fluoro‐2,4,6‐trimethylpyridinium tetrafluoroborate was not able to execute the rearrangement process returning only unreacted starting material. However, using 2 equivalents of a **Co(III)**‐**6** complex without any additional oxidant was able to convert tertiary alcohol (±)‐**3 c** into 0.4 : 1 mixture of ketones (±)‐**4 c** and (±)‐**5 c** (Figure 2C). This product ratio reflects the same preference for the migration of the more electron‐rich arene observed under catalytic conditions, implying that the aryl migration is cationic rather than radical in nature. Therefore, this is consistent with a cobalt(III) complex being responsible for the oxidation of the alkylcobalt(III) intermediate.[^54^, ^55^, ^56^]

**Figure 3:**
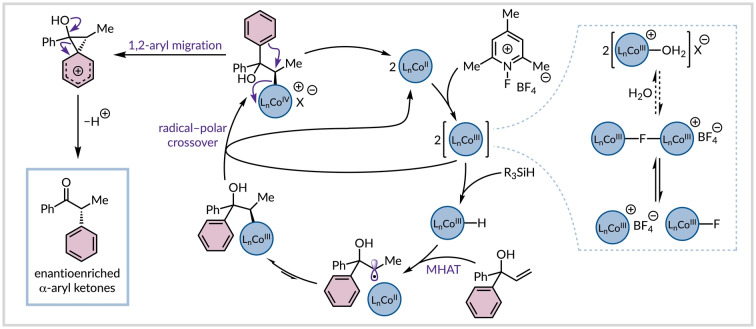
Proposed simplified catalytic cycle for the reaction.

Given previously devised catalytic cycles for similar cobalt‐catalyzed reactions, in‐depth mechanistic studies by others, and our findings, we tentatively propose a mechanism for our enantioselective hydro‐semipinacol rearrangement (Figure 3). Wilson and Holland have shown that *N*‐fluoropyridinium salts react with cobalt(II)‐salen complexes to generate a fluoride‐bridged Co(III) dimer.^[56]^ In wet solvents, this can be hydrolyzed to give a monometallic aquocobalt(III) species that was shown to be catalytically relevant and engages with a silane in the turnover‐limiting step.^[56]^ However, in our system, we have used anhydrous chlorobenzene as a solvent thus it is more probable that the fluoride‐bridged Co(III) dimer reacts directly with the silane or it releases monometallic entities to facilitate this step. Although we did not extensively dry the starting materials, reagents, or catalyst, so we cannot completely exclude the involvement of a water‐bound cobalt(III) intermediate. The subsequently formed transient Co(III) hydride species reacts with the allylic alcohol in a Markovnikov‐selective hydrogen atom transfer process to form an alkyl radical that is in equilibrium with an organocobalt(III) species.^[57]^ The reduced propensity for migration of electron‐poor arenes supports the notion that these intermediates do not undergo 1,2‐aryl migration. Instead, the alkyl‐Co(III) derivative is feasibly oxidized by another cobalt(III) complex to generate an organocobalt(IV) intermediate, which is in agreement with our stoichiometric experiment (Figure 2C).[^54^, ^56^] The high enantioselectivity observed in this transformation corroborates that the 1,2‐aryl migration occurs via stereospecific cleavage of the configurationally defined carbon–cobalt bond rather than a free carbocation intermediate formed after heterolysis.[^58^, ^59^, ^60^] The *ρ* value (−1.64) obtained from the Hammett analysis confirms the intermediacy of a phenonium ion, which leads to an enantioenriched α‐aryl ketone after the loss of a proton.

## Conclusions

In conclusion, a highly enantioselective cobalt‐catalyzed hydro‐semipinacol rearrangement of allylic alcohols involving 1,2‐aryl migration has been established. We demonstrated that alkylcobalt(IV) species can mimic the electrophilic behaviour of a secondary carbocation whilst providing a chiral environment to dictate the stereochemical outcome of the reaction. The polar nature of the rearrangement was probed by a Hammett analysis, which confirmed the preferential migration of the most electron‐rich aryl group. This protocol provides facile access to desirable α‐aryl ketones bearing tertiary stereocenters with excellent enantioselectivities (up to 99 : 1 er) and high yields, except for substrates containing *ortho*‐substituted aromatic rings.

## Conflict of Interests

The authors declare no conflict of interest.

1

## Supporting information

As a service to our authors and readers, this journal provides supporting information supplied by the authors. Such materials are peer reviewed and may be re‐organized for online delivery, but are not copy‐edited or typeset. Technical support issues arising from supporting information (other than missing files) should be addressed to the authors.

Supporting Information

## Data Availability

The research data underpinning this publication can be accessed at https://doi.org/10.17630/68da46d1‐51c0‐439d‐a051‐53536a8927ff.
