# Supplementary material for: Enantioselective Synthesis of α‐Aryl Ketones by a Cobalt‐Catalyzed Semipinacol Rearrangement
Source: Angew Chem Int Ed Engl. 2024 Nov 6;64(2):e202414342. doi: 10.1002/anie.202414342 (PMC11720393; doi:10.1002/anie.202414342)
Supplement: Supplementary file 1 — Supporting Information [file ANIE-64-e202414342-s001.pdf]

Angewandte Chemie
Eine Zeitschrift der Gesellschaft Deutscher Chemiker

Supporting Information

Enantioselective Synthesis of α-Aryl Ketones by a Cobalt-Catalyzed Semipinacol Rearrangement

*P. G. Kalomenopoulos, B. Emayavaramban, C. P. Johnston**

Supporting Information

Enantioselective Synthesis of α-Aryl Ketones by a Cobalt-Catalyzed Semipinacol Rearrangement

Contents

General Information 3

General Experimental Procedures 5

Catalyst Screen 9

Effect of Air on the Reaction 9

Optimisation 10

Limitations 11

Synthesis of Benzhydrols 12

Synthesis of Diaryl Ketones 18

Synthesis of Starting Materials 34

Synthesis of Ligands 69

Synthesis of Catalysts 73

Synthesis of Products 81

Hammett Plot 121

Deuterium-Labelling Experiment 124

Semipinacol Rearrangement with Co^{III} Salen Somplex 128

Eyring Analysis 129

Further Discussion on the Enantiodetermining Step 130

Varying Combination of Catalysts with Substrate 132

NMR Spectra 133

HPLC Data 251

References 280

General Information

Reactions involving moisture sensitive reagents were carried out in flame-dried glassware under an inert atmosphere (N_2) using standard vacuum line techniques. Anhydrous tetrahydrofuran was obtained after passing through an alumina column (Mbraun SPS-800). Anhydrous PhCl was prepared by drying over 4 Å molecular sieves which had been activated at 280 °C overnight under high vacuum. All other solvents and commercial reagents were used as received without further purification unless otherwise stated. Room temperature (RT) refers to 20 – 25 °C. Temperatures of 0 °C and –78 °C were obtained using ice/water and ethyl acetate/liquid nitrogen gas, respectively. Reactions involving heating were performed using DrySyn blocks and a contact thermocouple. Under reduced pressure refers to the use of either a Büchi Rotavapor R-200 with a Büchi V-491 heating bath and Büchi V-800 vacuum controller, a Büchi Rotavapor R-210 with a Büchi V-491 heating bath and Büchi V-850 vacuum controller, a Heidolph Laborota 4001 with vacuum controller, an IKA RV10 rotary evaporator with a IKA HB10 heating bath and ILMVAC vacuum controller, or an IKA RV10 rotary evaporator with an IKA HB10 heating bath and Vacuubrand CVC3000 vacuum controller. Rotary evaporator condensers are fitted to Julabo FL601 Recirculating Coolers filled with ethylene glycol and set to 0 °C. Analytical thin layer chromatography was performed on pre-coated aluminium plates (Kieselgel 60 F254 silica) and visualisation was achieved using ultraviolet light (254 nm) and/or staining with either aqueous $KMnO_4$ solution or ethanolic Vanillin solution followed by heating. Manual column chromatography was performed in glass columns fitted with porosity 3 sintered discs over Kieselgel 60 silica using the solvent system stated. Melting points were recorded on an Electrothermal 9100 melting point apparatus and are uncorrected. Infrared spectra were recorded on a Shimadzu IRAffinity-1 Fourier transform IR spectrophotometer fitted with a Specac Quest ATR accessory (diamond puck). Spectra were recorded neat with characteristic absorption wavenumbers (ν_{max}) reported in cm^{-1}. ^{1}H, $^{13}C\{^{1}H\}$, and $^{19}F\{^{1}H\}$ NMR spectra were acquired on either a Bruker AV400 with a BBFO probe (^{1}H 400 MHz; $^{13}C\{^{1}H\}$ 101 MHz; $^{19}F\{1H\}$ 377 MHz), a Bruker AVII 400 with a BBFO probe (^{1}H 400 MHz; $^{13}C\{^{1}H\}$ 101 MHz; $^{19}F\{^{1}H\}$ 376 MHz), a Bruker AVIII-HD 500 with a SmartProbe BBFO+ probe (^{1}H 500 MHz, $^{13}C\{^{1}H\}$ 126 MHz, $^{19}F\{^{1}H\}$ 471 MHz), a Bruker AVIII 500 with a CryoProbe Prodigy BBO probe (^{1}H 500 MHz, $^{13}C\{^{1}H\}$ 126 MHz, $^{19}F\{^{1}H\}$ 471 MHz), in the deuterated

solvent stated. ^{1}H and ^{13}C NMR spectra were referenced to residual non-deuterated solvent peaks. ^{19}F NMR spectra were referenced externally to $CFCl_3$ (0.00 ppm). All coupling constants, *J*, are quoted in Hz. Multiplicities are indicated as s (singlet), d (doublet), t (triplet), q (quartet), m (multiplet), and multiples thereof. The abbreviation Ar denotes aromatic and app. denotes apparent. Mass spectrometry (m/z) data were acquired by either electrospray ionisation (ESI) or electron impact (EI) at either the University of St Andrews Mass Spectrometry Facility or at SIRCAMS at University of Edinburgh Mass Spectrometry Facility. High Performance Liquid Chromatography (HPLC) was performed on an Agilent 1260 Infinity II LC system consisting of a 1260 Infinity II Quaternary Pump, 1260 Infinity II Vial Sampler, 1260 Infinity II Multicolumn Thermostat, and a 1260 Infinity II Diode Array Detector WR. Separation was achieved using either Daicel CHIRALPAK® AD-H, AS-H, IA, IB, IC, or IG columns or Daicel CHIRALCEL® OJ-H or OD-H columns using the method stated. All columns (4.6 mm ø x 250 mm, 5 μm particle size) were used with a corresponding guard column (4 mm ø x 10 mm, 5 μm particle size). HPLC traces of enantiomerically enriched compounds were compared with authentic racemic samples. Wavelengths (λ) are reported in nm, retention times (t_R) are reported in minutes, temperatures are reported in °C, and solvent flow rates are reported in mL/min. Aryl bromides and benzophenone derivatives were purchased commercially unless otherwise stated.

General Experimental Procedures

General procedure A: Synthesis of benzhydrol derivatives

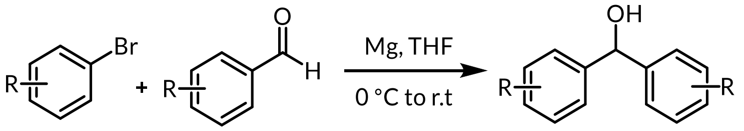

Following a literature procedure by Shi and co-workers[1], a freshly made Grignard solution from bromoarene derivative (1 equiv.) using magnesium turnings (1.1 equiv.) in THF was added to the benzaldehyde derivative (1 equiv.) at 0 °C. The reaction mixture was warmed to room temperature and stirred for 3 hours. The reaction was quenched with saturated aqueous NH_4Cl. The aqueous layer was extracted with Et_2O (× 3). The organic layers were combined, washed with brine, dried over Na_2SO_4, filtered, and concentrated under reduced pressure. The crude product was purified by silica gel column chromatography.

General procedure B: Oxidation of benzhydrol derivatives

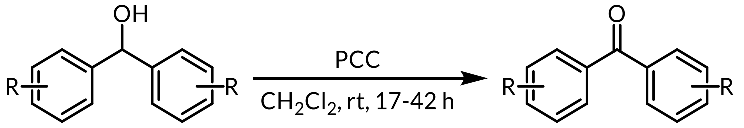

Following a literature procedure by Zhao and co-workers[2], benzhydrol derivative (1 equiv.) in DCM was added to pyridinium chlorochromate (1.5 equiv.) and silica (1.6 equiv.) and stirred at room temperature for 16-42 h. The reaction mixture was filtered through silica gel and the filtrate was purified by flash column chromatography.

Genral procedure C: Synthesis of symmetrical diaryl ketones

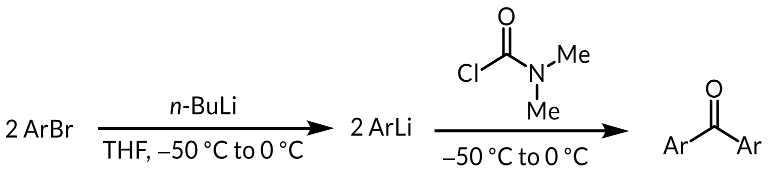

Following a literature procedure by Breau and co-workers[3], *n*-BuLi in hexanes (1.0 equiv.) was added to bromoaryl derivative (1 equiv.) in THF at –50 °C and stirred for 1 h, followed by dimethylcarbomyl chloride (0.5 equiv.) was added and stirred for 3 h at –50 °C, gradually warmed to 0 °C then quenched with 1 M HCl (5 mL). The aqueous layer was extracted using

Et_2O (3 × 10 mL). The organic layers were combined, washed with brine, dried over Na_2SO_4, filtered, and concentrated under reduced pressure. The crude product was purified by silica gel column chromatography to afford the desired diaryl ketone.

General procedure D: Vinyl Grignard reaction of diaryl ketones

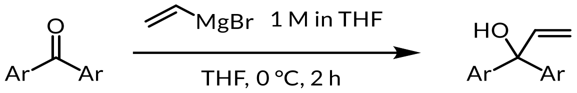

Following a literature procedure by Zhang and co-workers[4], to the appropriate ketone (1.0 equiv.) in THF (0.5 M) at 0 °C was added vinylmagnesium bromide (1.5–3 equiv.) and stirred at 0 °C for 2 h under an inert atmosphere. The reaction was quenched with saturated aqueous NH_4Cl. The aqueous layer was extracted with Et_2O (× 3). The organic layers were combined, washed with brine, dried over Na_2SO_4, filtered, and concentrated under reduced pressure. The crude product was purified by silica gel column chromatography to afford the desired diaryl allylic alcohol.

General procedure E: Synthesis of salen ligands

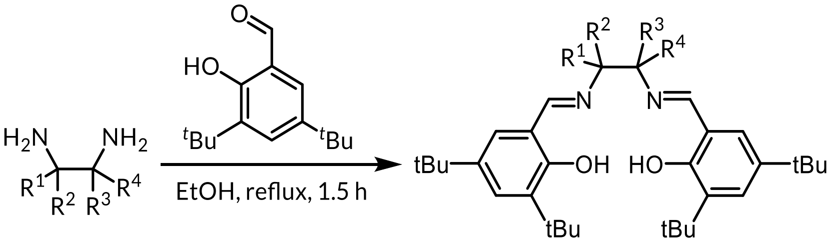

Following an adapted literature procedure[5], diamine (1 equiv.) and 3,5-di-*tert*-butyl-2-hydroxybenzaldehyde (2 equiv.) in EtOH (0.15 M) were heated under reflux for 1.5 hours and allowed to cool to room temperature. The resulting precipitate was vacuum filtered and washed with ice-cold ethanol.

General procedure F: Synthesis of cobalt catalysts

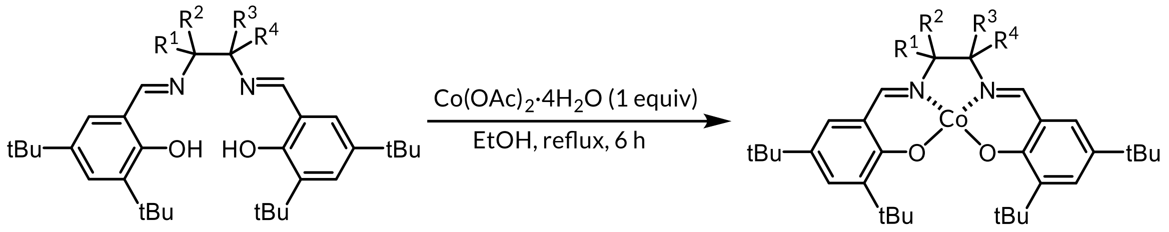

Salen ligand (1 equiv.) and $Co(OAc)_2{\cdot}4H_2O$ (1 equiv.) in ethanol (0.05 M) were heated under reflux and an inert (N_2) atmosphere for 6 hours and cooled in an ice bath. The precipitate was filtered and washed with MeOH at –18 °C (2-3 mL per 100 mg of ligand).

General procedure G: Screening procedure for fluorinated starting materials

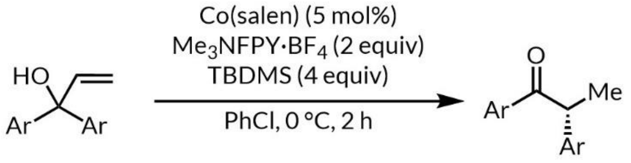

Cobalt salen complex (5 mol%), 1-fluoro-2,4,6-trimethylpyridinium tetrafluoroborate (2 equiv.) and alkene (1 equiv.) were placed in a flame-dried Schlenk flask and the atmosphere purged with nitrogen three times. Chlorobenzene (0.12 M) was degassed by sparging with N_2 for 15 minutes and then added to the Schlenk flask via syringe. The mixture was cooled to 0 °C and 1,1,3,3-tetramethyldisiloxane (4 equiv.) was added dropwise via micro syringe and the reaction was stirred for 2 hours. The reaction mixture was diluted with dichloromethane (5 mL per 0.1 mmol of alkene) to solubilise the reaction components and fluorobenzene (1 equiv.) was added via micro-syringe. A small aliquot (0.1 mL) of the mixture was diluted with $CDCl_3$ and the yield was analysed via quantitative ^{19}F NMR spectroscopy set to 4 scans and 25 second relaxation delay. Preparative thin layer chromatography (hexane : dichloromethane, 50 : 50) was used to obtain a sample for HPLC analysis.

General procedure H: Scope procedure for the cobalt-catalyzed semipinacol rearrangement

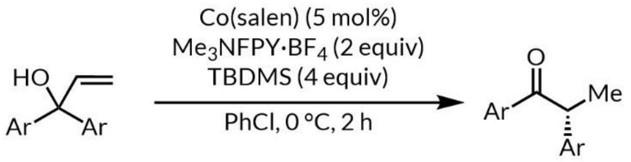

Cobalt salen complex (5 mol%), 1-fluoro-2,4,6-trimethylpyridinium tetrafluoroborate (2 equiv.) and alkene (1 equiv.) were placed in a flame-dried Schlenk flask and the atmosphere purged with nitrogen three times. Chlorobenzene (0.12 M) was degassed by sparging with N_2 for 15 minutes and then added to the Schlenk flask via syringe. The mixture was cooled to 0 °C and 1,1,3,3-tetramethyldisiloxane (4 equiv.) was added dropwise via micro syringe and the reaction was stirred for 2 hours. The crude mixture was loaded onto a column and purified by silica gel column chromatography.

Catalyst Screen

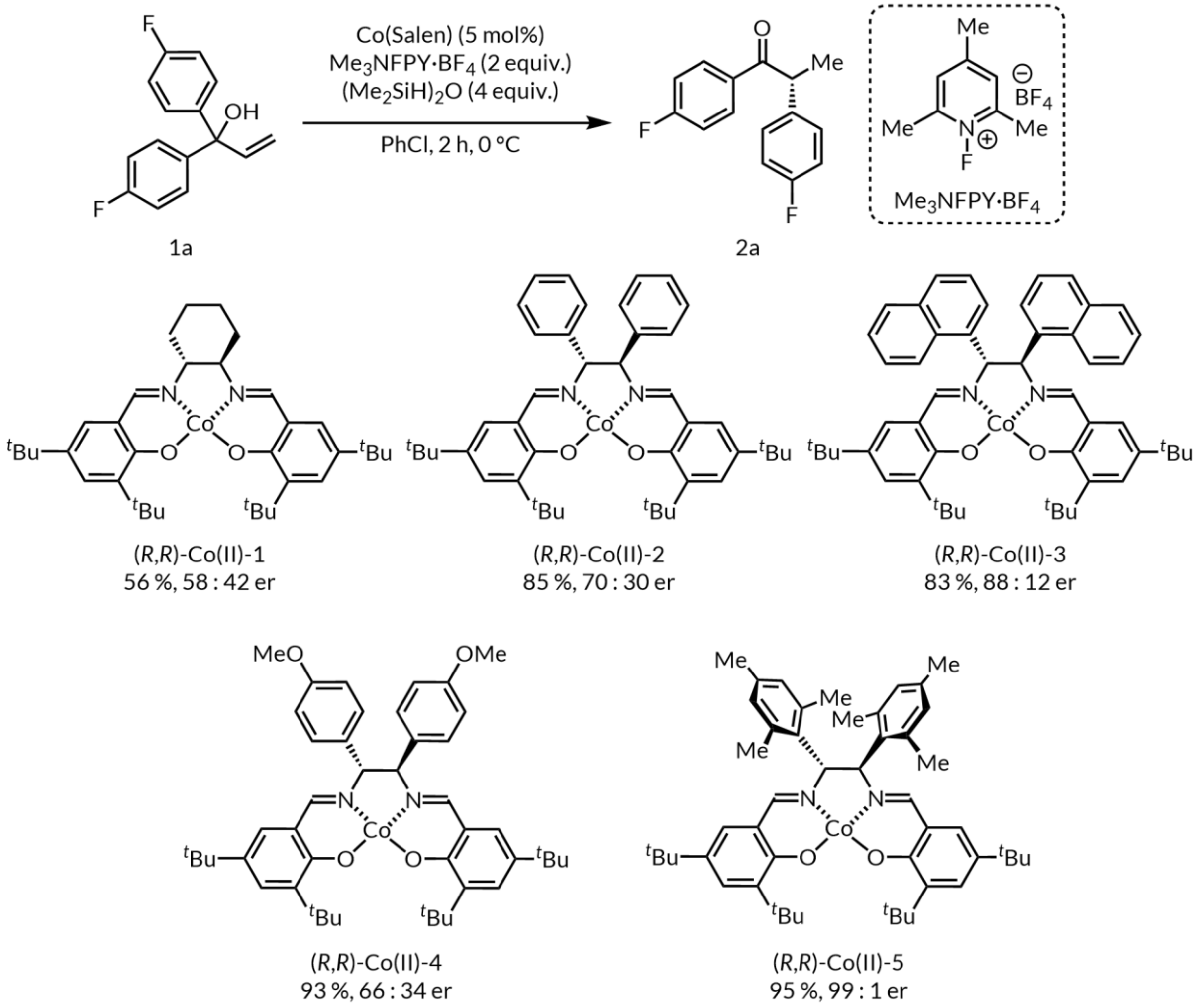

Effect of Air on the Reaction

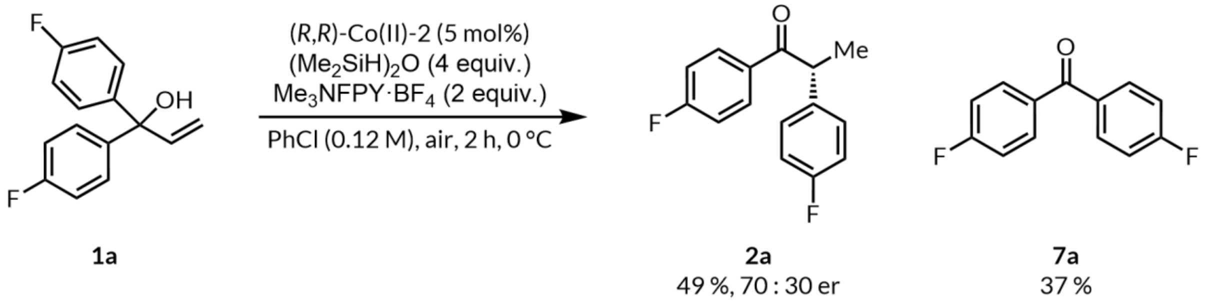

Optimisation

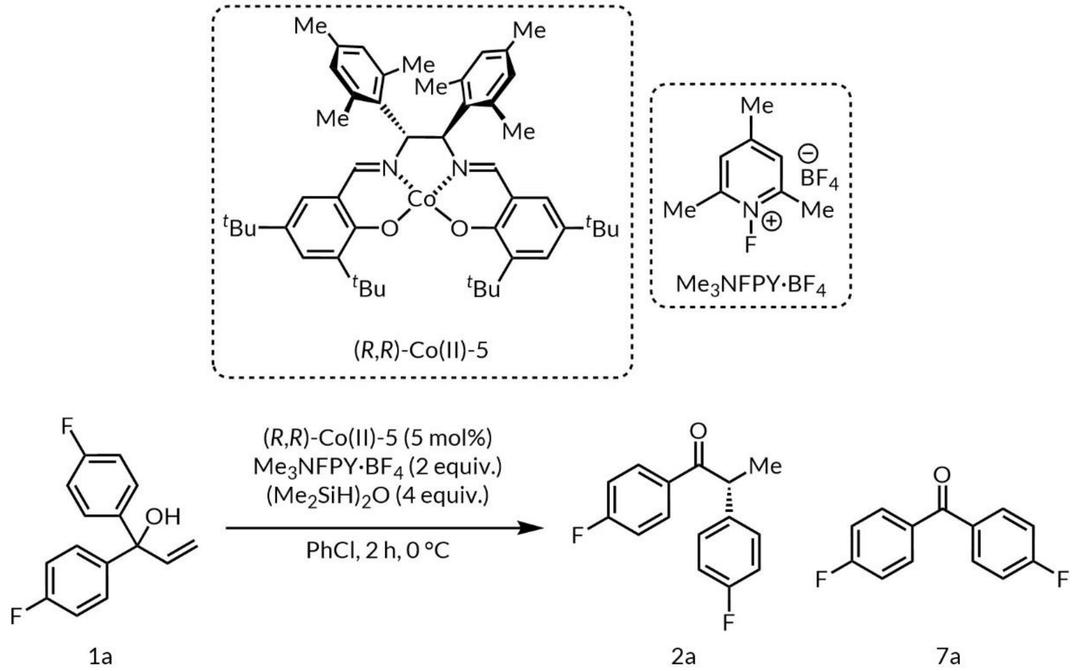

deviations	% yield*			er
	2a	7a	1a	2a
none	95	0	0	99 : 1
1.5 equiv. oxidant	76	0	0	97 : 3
3 silane : 1.5 oxidant	85	0	0	98 : 2
2 equiv. silane	91	4	0	98 : 2
3 equiv. silane	92	0	0	98 : 2
5 equiv. silane	79	12	0	95 : 5
3 mol% catalyst	77	17	0	96 : 4
1 mol% catalyst	69	7	20	96 : 4
0.2 M	81	11	0	98 : 2
RT	90	0	0	95 : 5
5 min	11	0	82	97 : 3
15 min	20	3	72	98 : 2
30 min	43	4	47	98 : 2
1 h	85	6	5	99 : 1
Methylphenylsilane	72	11	0	98 : 2
Methyldiethoxysilane	73	10	0	97: 3

Phenylsilane	26	10	11	97 : 3
Dimethylphenylsilane	94	0	0	96 : 4
Triethylsilane	0	0	92	N/A
Toluene	0	15	63	N/A
Dichloromethane	67	9	15	77 : 23
Tetrahydrofuran	0	20	66	N/A
Ethyl Acetate	55	0	20	89 : 11
Acetonitrile	9	11	20	70 : 30
Benzotrifluoride	87	0	9	95 : 5
Acetone	42	11	40	69 : 31

Limitations

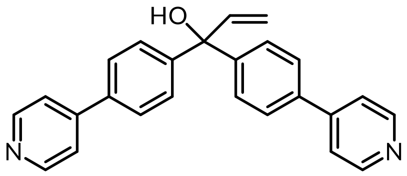

When catalytic procedure H was performed on 1,1-Bis(4-(pyridine-4-yl)phenyl)prop-2-en-1-ol (**1za**) no product was observed by ^{1}H NMR analysis of the crude reaction mixture.

Synthesis of Benzhydrols

Bis(4-(trifluoromethyl)phenyl)methanol (**6m**)

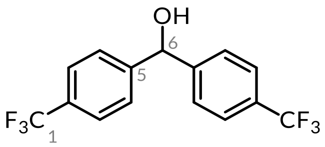

Following general procedure A, 1-bromo-4-(trifluoromethyl)benzene (1.69 g, 7.5 mmol), magnesium turnings (200 mg, 8.3 mmol), 4-trifluoromethylbenzaldehyde (1.31 g, 7.5 mmol), and THF (15 mL) for 2 h gave, after purification by column chromatography (hexane : diethyl ether, 95 : 5 to 70 : 30), alcohol **6m** as an orange powder (1.28 g, 4.0 mmol, 53%).

mp 78 – 79 °C

ν_{max} (film): 3298 (O–H), 1618, 1420, 1321, 1240, 1161, 1111, 1067, 1016, 872, 829, 812, 763.

^{1}H NMR (500 MHz, CDCl$_3$) δ_H: 7.62 (4H, d, *J* 8.1, **3-H**), 7.50 (4H, d, *J* 8.0, **4-H**), 5.94 (1H, d, *J* 3.1, **6-H**), 2.42 (1H, d, *J* 3.4, **OH**).

^{13}C{^{1}H} NMR (126 MHz, CDCl$_3$) δ_C: 146.9 (**C5**), 130.4 (q, *J* 32.5, **C2**), 126.9 (**C4**), 125.84 (q, *J* 3.7, **C3**), 124.1 (q, *J* 272.2, **C1**), 75.3 (**C6**).

^{19}F NMR (471 MHz, CDCl$_3$) δ_F: –62.6 (s, **CF$_3$**).

HRMS: (ESI$^-$) $C_{15}H_9F_6O$ [M–H]$^-$ found 319.0565, requires 319.0563 (+0.5 ppm).

Bis(3-chlorophenyl)methanol (**6o**)

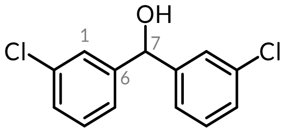

Following general procedure A, 1-bromo-3-chlorobenzene (1.44 g, 7.5 mmol), magnesium turnings (182 mg, 7.5 mmol), 3-chlorobenzaldehyde (1.05 g, 7.5 mmol), and THF (7.5 mL) for 2 h gave, after purification by column chromatography (hexane : diethyl ether, 95 : 5 to 70 : 30), alcohol **6o** as a white powder (1.35 g, 5.3 mmol, 71%).

ν_{max} (film): 3310 (O–H), 2874 (C–H), 1593, 1574, 1472, 1287, 1248, 1186, 1078, 1030, 899, 885, 789.

^{1}H NMR (500 MHz, $CDCl_3$) δ_H: 7.37 (2H, s, **1-H**), 7.31 – 7.21 (6H, m, **3-H**, **4-H**, **5-H**), 5.76 (1H, d, *J* 3.5, **C-7**), 2.30 (1H, d, *J* 3.5, **OH**).

^{13}C{^{1}H} NMR (126 MHz, $CDCl_3$) δ_C: 145.2 (**C6**), 134.7 (**C2**), 130.1 (**C4**), 128.2 (**C3**), 126.8 (**C1**), 124.8 (**C5**), 75.2 (**C7**).

HRMS: (ESI$^-$) $C_{13}H_9{}^{35}Cl_2O$ [M–H]$^-$ found 251.0036, requires 251.0036 (0.0 ppm).

Di-*m*-tolylmethanol (**6q**)

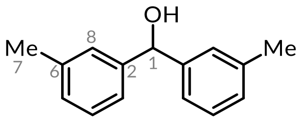

Following general procedure A, 3-bromotoluene (1.0 g, 5.8 mmol), magnesium turnings (156 mg, 6.4 mmol), *m*-tolualdehyde (697 mg, 5.8 mmol), and THF (10 mL) for 3 h gave, after purification by column chromatography (hexane : CH_2Cl_2, 75 : 25), alcohol **6q** as a colourless oil (740 mg, 3.5 mmol, 60%).

ν_{max} (film): 3229 (O–H), 3024 (C–H), 2918 (C–H), 1606, 1487, 1035, 740.

^{1}H NMR (400 MHz, $CDCl_3$) δ_H: 7.29 – 7.25 (4H, m, **4-H, 8-H**), 7.22 – 7.20 (2H, m, **3-H**), 5.77 (1H, s, **1-H**), 2.46 (1H, s, **OH**), 2.39 (6H, s, **7-H**).

^{13}C{^{1}H} NMR (126 MHz, $CDCl_3$) δ_C: 143.9 (**C2**), 138.1 (**C6**), 128.4 (**C4**), 128.3 (**C5**), 127.2 (**C8**), 123.6 (**C3**), 76.3 (**C1**), 21.5 (**C7**).

HRMS: (ESI$^+$) $C_{15}H_{16}{}^{23}NaO$ [M+Na]$^+$ found 235.1088, requires 235.1099 (–4.6 ppm).

Bis(3-methoxyphenyl)methanol (**6r**)

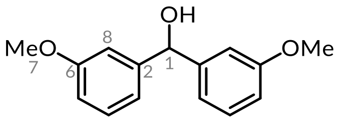

Following general procedure A, 3-bromoanisole (1.0 g, 5.3 mmol), magnesium turnings (143 mg, 5.9 mmol), 3-methoxybenzaldehyde (727 mg, 5.3 mmol), and THF (10 mL) for 3 h gave, after purification by column chromatography (hexane : CH_2Cl_2, 70 : 30), alcohol **6r** as a colourless (956 mg, 3.9 mmol, 74%).

ν_{max} (film): 3421 (O–H), 2933 (C–H), 2835 (C–H), 1597, 1485, 1035, 746.

^{1}H NMR (400 MHz, $CDCl_3$) δ_H: 7.24 – 7.23 (2H, m, **4-H**), 6.96 – 6.94 (4H, m, **3-H, 8-H**), 6.82 – 6.79 (2H, m, **5-H**), 5.76 (1H, s, **1-H**), 3.78 (6H, s, **7-H**), 2.39 (1H, s, **OH**).

^{13}C{^{1}H} NMR (126 MHz, $CDCl_3$) δ_C: 159.8 (**C6**), 145.4 (**C2**), 129.6 (**C4**), 118.9 (**C8**), 113.1 (**C5**), 112.1 (**C3**), 76.1 (**C1**), 55.3 (**C7**).

HRMS: (ESI$^+$) $C_{15}H_{16}{}^{23}NaO$ [M+Na]$^+$ found 267.0986, requires 267.0997 (–4.1 ppm).

Di-*o*-tolylmethanol (**6t**)

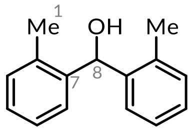

Following general procedure A, *o*-bromotoluene (500 mg, 2.92 mmol), magnesium turnings (78 mg, 3.21 mmol), *o*-tolualdehyde (351 mg, 2.92 mmol), and THF (5 mL) for 3 h gave, after purification by column chromatography (hexane : CH_2Cl_2, 70 : 30), alcohol **6t** as a white powder (446 mg, 2.10 mmol, 72%).

mp 117 – 119 °C

ν_{max} (film): 3194 (O–H), 1458, 1282, 1033, 1020.

^{1}H NMR (500 MHz, $CDCl_3$) δ_H: 7.30 – 7.28 (2H, m, **3-H**), 7.23 – 7.17 (6H, m, **4-H**, **5-H**, **6-H**), 6.14 (1H, d, *J* 4.9 Hz, **8-H**), 2.29 (6H, s, **1-H**), 1.99 (1H, d, *J* 4.9 Hz, **O-H**).

^{13}C{^{1}H} NMR (126 MHz, $CDCl_3$) δ_C: 140.9 (**C7**), 135.9 (**C2**), 130.6 (**C3**), 127.7 (**C5**), 126.6 (**C6**), 126.2 (**C4**), 70.3 (**C8**), 19.1 (**C1**).

HRMS: (ESI$^+$) $C_{16}H_{15}NaO$ [M+Na]$^+$ found 235.1093, requires 235.1099 (–2.5 ppm).

Bis(2-fluorophenyl)methanol (**6v**)

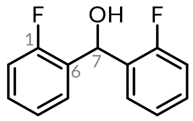

Following a literature procedure by de Franceschi and co-workers[6], to 2-fluorobromobenzene (1 g, 5.7 mmol) in THF (10 mL) at –78 °C was added *n*-BuLi (1.6 M in hexane, 3.6 mL, 5.76 mmol) and stirred for 1 hour under inert atmosphere. The reaction was warmed to –30 °C, ethyl formate (211 mg, 2.8 mmol) was added, the reaction mixture was stirred for 3 h and quenched with saturated aqueous NH_4Cl. The aqueous layer was extracted with Et_2O (3 × 10 mL). The organic layers were combined, washed with brine, dried over Na_2SO_4, filtered, and concentrated under reduced pressure. The crude product was purified by silica gel column chromatography (hexane : CH_2Cl_2, 70 : 30), to yield alcohol **6v** as a white solid (337 mg, 1.53 mmol, 60%).

mp 64 – 66 °C

ν_{max} (film): 3458 (O-H), 2958, 1506, 1411, 827.

^{1}H NMR (500 MHz, $CDCl_3$) δ_H: 7.47 – 7.44 (2H, m, **5-H**), 7.30 – 7.27 (2H, m, **3-H**), 7.17 – 7.14 (2H, m, **4-H**), 7.06 – 7.02 (2H, m, **2-H**), 6.42 (1H, d, *J* 4.3, **7-H**), 2.43 (1H, s, **O-H**).

^{13}C{^{1}H} NMR (126 MHz, $CDCl_3$) δ_C: 160.1 (d, *J* 247.2, **C1**), 129.7 – 129.6 (m, **C6**), 129.5 (**C3**), 128.1 (**C5**), 124.3 (**C4**), 115.6 (d, *J* 21.1, **C2**), 64.7 (t, *J* 3.7, **C7**).

^{19}F NMR (282 MHz, $CDCl_3$) δ_F: –118.2 (**F**).

HRMS: (EI^+) $C_{13}H_{10}F_2O$ $[M]^+$ found 220.0702, requires 220.0694 (+3.6 ppm).

Synthesis of Diaryl Ketones

Bis(4-isopropylphenyl)methanone (**7d**)

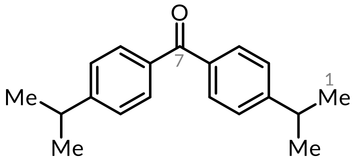

Following general procedure C, 4-bromocumene (500 mg, 2.51 mmol), *n*-BuLi (1.6 M in hexane, 1.6 mL, 2.5 mmol), dimethylcarbomyl chloride (134 mg, 1.26 mmol), THF (5 mL) for 3 h gave, after purification by column chromatography (hexane : CH_2Cl_2, 70 : 30), ketone **7d** as a white powder (206 mg, 0.77 mmol, 62%).

ν_{max} (film): 2960 (C–H), 1653 (C=O), 1653, 1274, 931.

^{1}H NMR (500 MHz, CDCl$_3$) δ_H: 7.76 – 7.75 (4H, m, **5-H**), 7.33 – 7.32 (4H, m, **4-H**), 2.99 (2H, hept, *J* 6.9 Hz, **2-H**), 1.30 (12H, d, *J* 6.9 Hz, **1-H)**.

^{13}C{^{1}H} NMR (126 MHz, CDCl$_3$) δ_C: 196.3 (**C7**), 153.8 (**C3**), 135.7 (**C6**), 130.4 (**C5**), 126.4 (**C4**), 34.4 (**C2**), 23.8 (**C1**).

HRMS: (EI$^+$) $C_{19}H_{22}O$ [M]$^+$ found 266.1665, requires 266.1665 (0.0 ppm).

Bis(4-(*tert*-butyl)phenyl)methanone (**7e**)

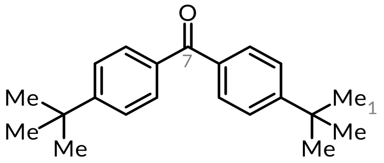

Following general procedure C, 1-bromo-4-*tert*-butylbenzene (500 mg, 2.3 mmol), *n*-BuLi (1.6 M in hexane, 1.4 mL, 2.3 mmol), dimethylcarbomyl chloride (126 mg, 1.17 mmol), THF (5 mL) for 3 h gave, after purification by column chromatography (hexane : CH_2Cl_2, 70 : 30), ketone **7e** as a white powder (193 mg, 0.66 mmol 56%).

mp 134 – 136 °C

ν_{max} (film): 2960, 2902, 1654 (C=O), 1604, 1278, 933.

^{1}H NMR (500 MHz, $CDCl_3$) δ_H: 7.77 – 7.76 (4H, m, **5-H**), 7.50 – 7.48 (4H, m, **4-H**), 1.37 (18H, s, **1-H)**.

^{13}C{^{1}H} NMR (126 MHz, $CDCl_3$) δ_C: 196.3 (**C7**), 156.0 (**C6**), 135.2 (**C3**), 130.1 (**C5**), 125.3 (**C4**), 35.2 (**C2**), 31.3 (**C1**).

HRMS: (EI^+) $C_{21}H_{26}O$ $[M]^+$ found 294.1976, requires 294.1978 (–0.6 ppm).

Di((1,1′-biphenyl)-4-yl)methanone (**7f**)

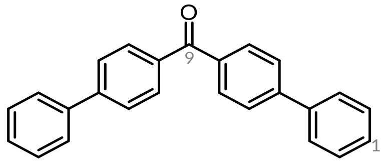

Following a literature procedure by Xu and co-workers[7], bis(4-bromophenyl)methanone (500 mg, 1.5 mmol) and arylboronic acid (434 mg, 3.6 mmol) were dissolved in the mixture of toluene (8 mL), TBAB (327 mg, 1.0 mmol) and 2 M potassium carbonate aqueous solution (2 mL). The mixture was stirred at room temperature under argon atmosphere for 0.5 h, $Pd(PPh_3)_4$ (3.4 mg, 19 mol%) was added and the mixture was heated to 85 °C for 16 h. The mixture was poured into water, extracted with ethyl acetate (x 3) and the organic layer was dried over anhydrous sodium sulfate. After removing the solvent under reduced pressure, the residue was purified by flash coloumn chromatography (hexane : CH_2Cl_2, 50 : 50) to yield ketone **7f** as pink solid (401 mg, 1.20 mmol, 80%).

mp 238 – 240 °C

ν_{max} (film): 3053, 1639 (C=O), 1602, 1400, 1298, 860.

^{1}H NMR (500 MHz, $CDCl_3$) δ_H: 7.95 – 7.93 (4H, m, **7-H**), 7.74 – 7.73 (4H, m, **6-H**), 7.68 – 7.66 (4H, m, **3-H**), 7.51 – 7.48 (4H, m, **2-H**), 7.43 – 7.40 (2H, m, **1-H**).

^{13}C{^{1}H} NMR (126 MHz, $CDCl_3$) δ_C: 196.1 (**C9**), 145.3 (**C5**), 140.1 (**C4**), 136.5 (**C8**), 130.8 (**C7**), 129.1 (**C2**), 128.3 (**C1**), 127.4 (**C3**), 127.1 (**C6**).

HRMS: (EI^+) $C_{25}H_{18}O$ $[M]^+$ found 334.1348, requires 334.1352 (–1.2 ppm).

Bis(4-(methylthio)phenyl)methanone (**7k**)

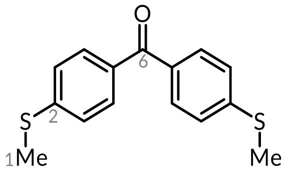

Following general procedure C, 4-bromothioanisole (500 mg, 2.5 mmol), *n*-BuLi (1.6 M in hexane, 1.5 mL, 2.5 mmol), dimethylcarbomyl chloride (134 mg, 1.2 mmol), THF (5 mL) for 4 h gave, after purification by column chromatography (hexane : CH_2Cl_2, 70 : 30), ketone **7k** as a white solid (236 mg, 0.86 mmol, 70%).

mp 120 – 122 °C

ν_{max} **(film):** 2918, 1635 (C=O), 1539, 1292, 1089, 846.

^{1}H NMR (500 MHz, $CDCl_3$) δ_H: 7.72 – 7.71 (4H, m, **4-H**), 7.30 – 7.28 (4H, m, **3-H**), 2.54 (6H, s, **1-H)**.

^{13}C{^{1}H} NMR (126 MHz, $CDCl_3$) δ_C: 195.0 (**C6**), 145.1 (**C2**), 134.0 (**C5**), 130.6 (**C4**), 125.0 (**C3**), 15.0 (**C1**).

HRMS: (EI$^+$) $C_{15}H_{14}OS_2$ $[M]^+$ found 274.0475, requires 274.0480 (–1.8 ppm).

Bis(4-((trimehtylsilyl)ethynyl)phenyl)methanone (**7l**)

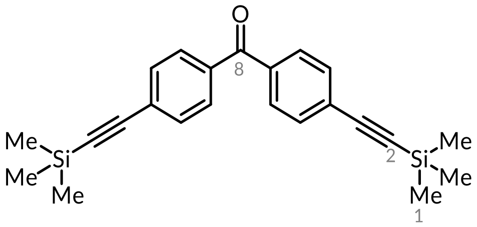

Following a literature procedure by Dong and co-workers[8], 4,4'-dibromobenzophenone (500 mg, 1.5 mmol), $PdCl_2(PPh_3)_2$ (43 mg, 2.5 mol%), CuI (6.9 mg, 2.5 mol%), PPh_3 (190.1 mg, 0.72 mmol), THF (5 mL) and anaerobic TEA (440 mg, 4.3 mmol) where stirred at 70 °C for 30 min. Trimethylsilylacetylene (216 mg, 3.3 mmol) was added and the reaction was stirred for 24 hours, then cooled to room temperature. Solvent was removed under reduced pressure and the crude product was purified by flash coloumn chromatography (hexane : CH_2Cl_2, 70 : 30), to ketone **7l** as a white solid (450 mg, 0.85 mmol, 80%).

mp 165 – 167 °C

ν_{max} (film): 2954 (C–H), 2158, 1654 (C=O), 1598, 1251, 842.

^{1}H NMR (500 MHz, $CDCl_3$) δ_H: 7.72 – 7.70 (4H, m, **6-H**), 7.57 – 7.55 (4H, m, **5-H**), 0.27 (18H, s, **1-H)**.

^{13}C{^{1}H} NMR (126 MHz, $CDCl_3$) δ_C: 195.2 (**C8**), 136.8 (**C4**), 131.9 (**C5**), 129.9 (**C6**), 127.6 (**C7**), 104.1 (**C3**), 98.1 (**C2**), –0.01(**C1**).

HRMS: (EI$^+$) $C_{23}H_{26}OSi_2$ [M]$^+$ found 374.1516, requires 374.1505 (–2.9 ppm).

Bis(4-(trifluoromethyl)phenyl)methanone (**7m**)

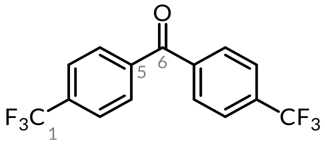

Following general procedure B, bis(4-(trifluoromethyl)phenyl)methanol (**6m**) (1.28 g, 4.0 mmol), silica (6.67 g), pyridinium chlorochromate (1.28 g, 6.0 mmol), and dichloromethane (15 mL) for 20 h gave, after purification by column chromatography (hexane : diethyl ether, 98 : 2 to 80 : 20), ketone **7m** as an white powder (0.92 g, 2.9 mmol, 73%).

mp 108 – 109 °C

ν_{max} (film): 1655 (C=O), 1510, 1410, 1325, 1128, 1069, 1016, 932, 864, 775.

^{1}H NMR (400 MHz, $CDCl_3$) δ_H: 7.91 (4H, d, *J* 8.0, **4-H**), 7.79 (4H, d, *J* 8.1, **3-H**).

^{13}C{^{1}H} NMR (126 MHz, $CDCl_3$) δ_C: 194.5 (**C6**), 139.9 (**C5**), 134.5 (q, *J* 32.9, **C2**), 130.4 (**C4**), 125.8 (q, *J* 3.7, **C3**), 123.7 (q, *J* 272.8, **C1**).

^{19}F NMR (471 MHz, $CDCl_3$) δ_F: –63.2 (s, CF_3).

HRMS: (ESI$^+$) $C_{15}H_8F_6NaO$ [M+Na]$^+$ found 341.0378, requires 341.0372 (+1.8 ppm).

Bis(3-chlorophenyl)methanone (**7o**)

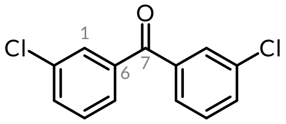

Following general procedure B, bis(3-chlorophenyl)methanol (**6o**) (1.28 g, 5.07 mmol), pyridinium chlorochromate (1.86 g, 8.62 mmol), silica (8.33 g) and CH_2Cl_2 (16 mL) for 45 h gave, after purification by a short silica plug (dichloromethane), ketone **7o** as a white powder (1.14 g, 4.52 mmol, 89%).

mp 121 – 123 °C

ν_{max} (film): 3065 (C–H), 1651 (C=O), 1566, 1416, 1281, 1265, 1078, 799.

^{1}H NMR (500 MHz, $CDCl_3$) δ_H: 7.77 (2H, t, *J* 1.9, **1-H**), 7.65 (2H, dt, *J* 7.7, 1.3, **5-H**), 7.59 (2H, ddd, *J* 8.0, 2.2, 1.1, **3-H**), 7.44 (2H, t, *J* 7.9, **4-H**).

^{13}C{^{1}H} NMR (126 MHz, $CDCl_3$) δ_C: 194.0 (**C7**), 138.8 (**C2**/**C6**), 135.0 (**C2**/**C6**), 132.9 (**C3**), 130.0 (**C1**), 129.9 (**C4**), 128.2 (**C5**).

HRMS: (ESI$^+$) $C_{13}H_8{}^{35}Cl_2NaO$ [M+Na]$^+$ found 272.9843, requires 272.9844 (–0.5 ppm).

Di-*m*-tolylmethanone (**7q**)

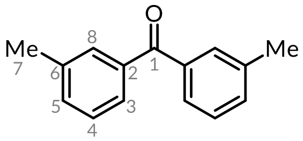

Following general procedure B, di-*m*-tolylmethanol (600 g, 2.83 mmol), silica (272 mg, 4.5 mmol), pyridinium chlorochromate (914 mg, 4.24 mmol), and dichloromethane (10 mL) for 24 h gave, after purification by column chromatography (hexane : CH_2Cl_2, 70 : 30), ketone **7q** as a colourless oil (512 mg, 2.43 mmol, 86%).

ν_{max} (film): 2922, 1654 (C=O), 1600, 1288, 1184, 790.

^{1}H NMR (400 MHz, $CDCl_3$) δ_H: 7.63 (2H, s, **8-H**), 7.58 – 7.56 (2H, m, **3-H**), 7.41 – 7.39 (2H, m, **5-H**), 7.37 – 7.34 (2H, m, **4-H**), 2.42 (6H, s, **7-H**).

^{13}C{^{1}H} NMR (126 MHz, $CDCl_3$) δ_C: 197.3 (**C1**), 138.2 (**C6**), 137.9 (**C2**), 133.2 (**C5**), 130.5 (**C8**), 128.1 (**C4**), 127.4 (**C3**), 21.5 (**C7**).

HRMS: (ESI$^+$) $C_{15}H_{14}{}^{23}NaO$ $[M+Na]^+$ found 233.0932, requires 233.0932 (–4.7 ppm).

Bis(3-methoxyphenyl)methanone (**7r**)

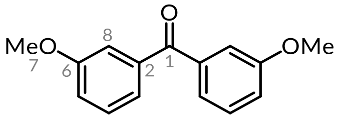

Following general procedure B, bis(3-methoxyphenyl)methanol (600 mg, 2.46 mmol), silica (272 mg, 4.5 mmol), pyridinium chlorochromate (235 mg, 3.9 mmol), and dichloromethane (10 mL) for 24 h gave, after purification by column chromatography (hexane : CH_2Cl_2, 50 : 50), ketone **7r** as a colourless oil (414 mg, 1.7 mmol, 70%). Spectroscopic data were in accordance with the literature.[9]

ν_{max} (film): 2939, 1658 (C=O), 1600, 1595, 1284, 1074, 748.

^{1}H NMR (400 MHz, $CDCl_3$) δ_H: 7.39 – 7.33 (6H, s, **3-H, 4-H, 8-H**), 7.14 – 7.12 (2H, m, **5-H**), 3.86 (6H, s, **7-H**).

^{13}C{^{1}H} NMR (126 MHz, $CDCl_3$) δ_C: 196.4 (**C1**), 159.6 (**C6**), 139.0 (**C2**), 129.3 (**C8**), 122.9 (**C3**), 119.0 (**C5**), 114.4 (**C4**), 55.6 (**C7**).

HRMS: (ESI$^+$) $C_{15}H_{15}O_3$ [M+H]$^+$ found 243.1009, requires 243.1016 (–2.88 ppm).

Di-*o*-tolylmethanone (**7t**)

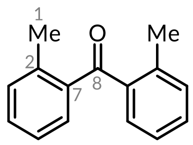

Following general procedure B, di-*o*-tolylmethanol (600 mg, 2.8 mmol), silica (269 mg, 4.5 mmol), pyridinium chlorochromate (914 mg, 4.2 mmol), and dichloromethane (10 mL) for 24 h gave, after purification by column chromatography (hexane : CH_2Cl_2, 70 : 30), ketone **7t** as a colourless oil (493 mg, 2.3 mmol 83%).

ν_{max} (film): 2964, 2926, 1660 (C=O), 1598, 1454, 1255, 923.

^{1}H NMR (400 MHz, $CDCl_3$) δ_H: 7.40 – 7.37 (2H, m, **4-H**), 7.32 – 7.27 (4H, m, **3-H**, **6-H**), 7.22 – 7.18 (2H, m, **5-H**), 2.44 (6H, s, **1-H**).

^{13}C{^{1}H} NMR (126 MHz, $CDCl_3$) δ_C: 200.9 (**C8**), 139.1 (**C7**), 138.3 (**C2**), 131.5 (**C3**), 131.2 (**C4**), 130.4 (**C6**), 125.5 (**C5**), 20.7 (**C1**).

HRMS: (ESI^+) $C_{15}H_{14}NaO$ $[M+Na]^+$ found 233.0936, requires 233.0942 (–2.6 ppm).

Bis(2-methoxyphenyl)methanone (**7u**)

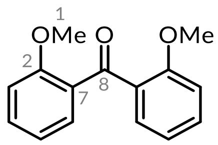

Following general procedure B, bis(2-methoxyphenyl)methanol (1.0 g, 4.1 mmol), silica (394 mg, 6.6 mmol), pyridinium chlorochromate (1.3 g, 6.1 mmol), and dichloromethane (10 mL) for 24 h gave, after purification by column chromatography (hexane : CH_2Cl_2, 50 : 50), ketone **7u** as a white solid (730 mg, 3.0 mmol, 73%). Characterisation data were in accordance with the literature.[3]

mp 102 – 104 °C

ν_{max} (film): 2941, 2837, 1641 (C=O), 1597, 1485, 1247, 925.

^{1}H NMR (400 MHz, $CDCl_3$) δ_H: 7.52 – 7.50 (2H, m, **6-H**), 7.45 – 7.41 (2H, m, **4-H**), 7.00 – 6.97 (2H, m, **5-H**), 6.92 (2H, d, *J* 6.2, **3-H**), 3.67 (6H, s, **1-H**).

^{13}C{^{1}H} NMR (126 MHz, $CDCl_3$) δ_C: 195.4 (**C8**), 158.4 (**C7**), 132.7 (**C4**), 130.5 (**C6**), 130.3 (**C2**), 120.4 (**C5**), 111.5 (**C3**), 55.8 (**C1**).

HRMS: (ESI$^+$) $C_{15}H_{14}{}^{23}NaO_3$ $[M+Na]^+$ found 265.0835, requires 265.0841 (–2.3 ppm).

Bis(2-fluorophenyl)methanone (**7v**)

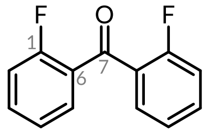

Following general procedure B, bis(2-fluorophenyl)methanol (**6t**) (350 g, 1.60 mmol), pyridinium chlorochromate (685 mg, 3.18 mmol), silica (3.50 g), CH_2Cl_2 (10 mL) for 16 h gave, after purification by flash column chromatography (hexane : CH_2Cl_2, 70 : 30), ketone **7v** as a white powder (279 mg, 1.28 mmol, 81%).

ν_{max} (film): 1660 (C=O), 1608, 1456, 1305, 931.

^{1}H NMR (500 MHz, $CDCl_3$) δ_H: 7.72 – 7.70 (2H, m, **5-H**), 7.56 – 7.52 (2H, m, **3-H**), 7.27 – 7.25 (2H, m, **4-H**), 7.13 – 7.10 (2H, m, **2-H**).

^{13}C{^{1}H} NMR (126 MHz, $CDCl_3$) δ_C: 189.8 (**C7**), 161.2 (d, *J* 255, **C1**), 134.3 (d, *J* 9.2, **C3**), 131.0 (**C5**), 127.6 (d, *J* 12.2, **C6**), 124.4 (**C4**), 116.3 (d, *J* 21.8, **C2**).

^{19}F NMR (282 MHz, $CDCl_3$) δ_F: −112.1 (**F**).

HRMS: (EI^+) $C_{13}H_8F_2O$ $[M]^+$ found 218.0531, requires 218.0537 (−2.7 ppm).

Di(benzofuran-2-yl)methanone (**7w**)

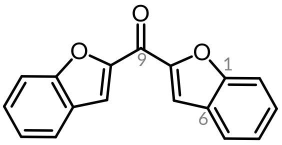

Following general procedure C, 2,3-benzofuran (500 mg, 4.23 mmol), *n*-BuLi (1.6 M in hexane, 2.6 mL, 4.2 mmol), dimethylcarbomyl chloride (227 mg, 2.11 mmol), THF (5 mL) for 4 h gave, after purification by column chromatography (hexane : CH_2Cl_2, 70 : 30), ketone **7w** as a beige solid (331.9 mg, 1.27 mmol, 60%).

mp 153 – 155 °C

ν_{max} (film): 3118, 1645 (C=O), 1546, 1438, 1294, 1028.

^{1}H NMR (500 MHz, $CDCl_3$) δ_H: 8.03 (2H, s, **7-H**), 7.80 – 7.79 (2H, m, **5-H**), 7.68 – 7.67 (2H, m, **2-H**), 7.54 – 7.52 (2H, m, **3-H**), 7.37 – 7.35 (2H, m, **4-H**).

^{13}C{^{1}H} NMR (126 MHz, $CDCl_3$) δ_C: 171.7 (**C9**), 156.0 (**C1**), 151.7 (**C8**), 128.7 (**C3**), 127.2 (**C6**), 124.2 (**C4**), 123.6 (**C5**), 116.3 (**C7**), 112.6 (**C2**).

HRMS: (EI$^+$) $C_{17}H_{10}O_3$ $[M]^+$ found 262.0622, requires 262.0624 (–0.7 ppm).

Bis(thiobenzophen-2-yl)methanone (**7x**)

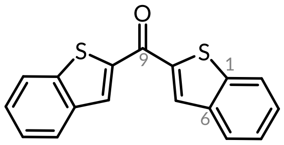

Following general procedure C, benzothiophene (500 mg, 3.73 mmol), *n*-BuLi (1.6 M in hexane, 2.3 mL, 2.7 mmol), dimethylcarbomyl chloride (200 mg, 1.86 mmol), THF (5 mL) for 4 h gave, after purification by column chromatography (hexane : CH_2Cl_2, 50 : 50), ketone **7x** as a yellow solid (301 mg, 0.65 mmol, 55%).

mp 154 – 156 °C

ν_{max} (film): 3057, 1612 (C=O), 1512, 1290, 1153.

^{1}H NMR (500 MHz, $CDCl_3$) δ_H: 8.22 (2H, s, **7-H**), 7.97 – 7.93 (4H, m, **2-H**, **5-H**), 7.53 – 7.44 (4H, m, **3-H**, **4-H**).

^{13}C{^{1}H} NMR (126 MHz, $CDCl_3$) δ_C: 181.6 (**C9**), 142.5 (**C1**), 142.3 (**C8**), 139.1 (**C6**), 130.9 (**C7**), 127.6 (**C3**), 126.2 (**C5**), 125.3 (**C4**), 123.0 (**C2**).

HRMS: (EI^+) $C_{17}H_{10}OS_2$ $[M]^+$ found 294.0173, requires 294.0167 (+2.0 ppm).

Bis(6-methoxynaphthalen-2-yl)methanone (**7z**)

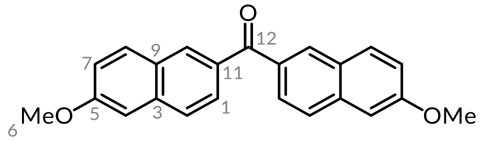

Following general procedure C, 2-bromo-6-methoxynaphthalene (500 mg, 2.11 mmol), *n*-BuLi (1.6 M in hexane, 1.3 mL, 2.1 mmol), dimethylcarbomyl chloride (113 mg, 1.05 mmol), THF (5 mL) for 4 h gave, after purification by column chromatography (hexane : CH_2Cl_2, 50 : 50), ketone **7z** as a beige solid (201 mg, 0.59 mmol, 56%).

mp 172 – 174 °C

ν_{max} (film): 2935 (C–H), 1620 (C=O), 1479, 1197, 1028.

^{1}H NMR (500 MHz, $CDCl_3$) δ_H: 8.26 (2H, s, **10-H**), 7.98 – 7.96 (2H, m, **1-H**), 7.86 – 7.81 (4H, m, **2-H**, **8-H**), 7.22 – 7.21 (4H, m, **4-H**, **7-H**), 3.97 (6H, s, **6-H**).

^{13}C{^{1}H} NMR (126 MHz, $CDCl_3$) δ_C: 196.6 (**C12**), 159.7 (**C5**), 137.0 (**C3**), 133.4 (**C9**), 131.7 (**C10**), 131.1 (**C8**), 127.8 (**C11**), 127.1 (**C2**), 126.8 (**C1**), 119.8 (**C4**), 105.9 (**C7**), 55.5 (**C6**).

HRMS: (EI^+) $C_{23}H_{18}O_3$ $[M]^+$ found 342.1259, requires 342.1251 (+2.3 ppm).

Bis(4-(pyridine-4-yl)phenyl)methanone (**7za**)

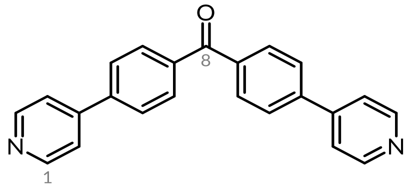

Following a literature procedure by Echegoyen and co-workers[10], bis(4-bromophenyl)methanone (600 mg, 1.76 mmol), 4-(4,4,5,5-tetramethyl-1,3,2-dioxaborolan-2-yl)pyridine (941.2 mg, 4.59 mmol), K_2CO_3 (1.46 g, 10.59 mmol) and tetrakis(triphenylphosphine)palladium (61.2 mg, 0.2 mmol) were dissolved in toluene (15 mL), EtOH (7.5 mL) and water (7.5 mL) and refluxed under nitrogen atmosphere for 24 h. The reaction mixture was cooled to room temperature, solvent was removed under reduce preasure and the crude was purified by flash coloumn chromatograpy (ethylacetate : Methanol, 99 : 1) to yield ketone **7za** as a yellow solid (462 mg, 1.37 mmol, 78% yield). ^{1}H NMR data were in accordance with the literature.[10]

mp 212 – 214 °C

ν_{max} (film): 3025, 1641 (C=O), 1591, 1400, 1292, 931.

^{1}H NMR (500 MHz, CDCl$_3$) δ_H: 8.72 (2H, d, *J* 5.7, **1-H**), 7.96 (2H, d, *J* 8.2, **6-H**), 7.77 (2H, d, *J* 8.2, **5-H**), 7.56 (2H, d, *J* 5.7, **2-H**).

^{13}C{^{1}H} NMR (126 MHz, CDCl$_3$) δ_C: 195.4 (**C8**), 150.6 (**C1**), 147.2 (**C3**), 142.3 (**C4**), 137.7 (**C7**), 130.9 (**C6**), 127.2 (**C5**), 121.8 (**C2**).

HRMS: (EI$^+$) $C_{23}H_{17}N_2O$ [M+H]$^+$ found 336.1335, requires 336.1341 (-1.7 ppm).

Synthesis of Starting Materials

1,1-Bis(4-fluorophenyl)prop-2-en-1-ol (**1a**)

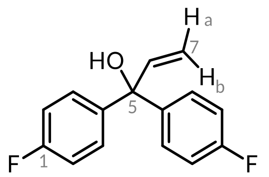

Following general procedure D, bis(4-fluorophenyl)methanone (1.09 g, 5.00 mmol), vinylmagnesium bromide (1 M in THF, 12.5 mL, 12.5 mmol), and THF (10 mL) for 2 h gave, after purification by column chromatography (hexane : diethyl ether 80 : 20), allylic alcohol **1a** as a colourless oil (1.15 g, 4.68 mmol, 94%). All spectroscopic data were in accordance with the literature.[11]

ν_{max} (film): 3446 (O–H), 3066 (C–H), 1504, 1223, 1165 (C–F), 829.

^{1}H NMR (400 MHz, CDCl$_3$) δ_H: 7.37–7.29 (4H, m, **3-H**), 7.06–6.97 (4H, m, **2-H**), 6.44 (1H, dd, *J* 10.6, 17.0, **6-H**), 5.33 (1H, dd, *J* 10.5, 1.0, **7a-H**), 5.28 (1H, dd, *J* 17.0, 1.0, **7b-H**), 2.27 (1H, s, **OH**).

^{13}C{^{1}H} NMR (126 MHz, CDCl$_3$) δ_C: 162.1 (d, *J* 246.8, **C1**), 143.3 (**C6**), 141.8 (d, *J* 2.9, **C4**), 128.8 (d, *J* 8.0, **C3**), 115.2 (d, *J* 20.7, **C2**), 114.6 (**C7**), 78.8 (**C5**).

^{19}F NMR (282 MHz, CDCl$_3$) δ_F: –115.2 (**F**).

HRMS: (ESI$^+$) $C_{15}H_{10}F_2$ [M–OH]$^+$ found 229.0817, requires 229.0823 (–2.6 ppm).

1,1-Diphenylprop-2-en-1-ol (**1b**)

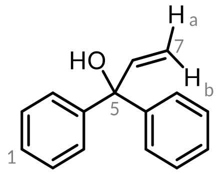

Following general procedure D, benzophenone (364 mg, 2.00 mmol), vinylmagnesium bromide (1 M in THF, 5.0 mL, 5.0 mmol), and THF (2 mL) for 2 h gave, after purification by column chromatography (hexane : diethyl ether, 80 : 20), allylic alcohol **1b** as a colourless oil (384 mg, 1.83 mmol, 91%).

ν_{max} (film): 3428 (O–H), 3057 (C–H), 1489, 1447, 995, 758.

^{1}H NMR (500 MHz, $CDCl_3$) δ_H: 7.42 – 7.37 (4H, m, **3-H**), 7.36 – 7.30 (4H, m, **2-H**), 7.29 – 7.24 (2H, m, **1-H**), 6.59 – 6.46 (1H, m, **6-H**), 5.34 (1H, dd, *J* 6.3, 1.3, **7b-H**), 5.31 (1H, s, **7a-H**), 2.27 (1H, s, **OH**).

^{13}C{^{1}H} NMR (126 MHz, $CDCl_3$) δ_C: 145.9 (**C4**), 143.6 (**C6**), 128.3 (**C2**), 127.4 (**C1**), 127.0 (**C3**), 114.2 (**C7**), 79.5 (**C5**).

HRMS: (ESI$^+$) $C_{15}H_{14}O$ [M+H]$^+$ found 233.0937, requires 233.0936 (–0.4 ppm).

1,1-Di-*p*-tolylprop-2-en-1-ol (**1c**)

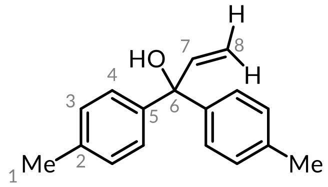

Following general procedure D, di-*p*-tolylmethanone (315 mg, 1.50 mmol), vinylmagnesium bromide (1 M in THF, 3.8 mL, 3.8 mmol), and THF (2 mL) for 2 h gave, after purification by column chromatography (hexane : diethyl ether, 80 : 20), allylic alcohol **1c** as a colourless oil (240 mg, 1.01 mmol, 67%).

ν_{max} (film): 3446 (O–H), 3022 (C–H), 2920 (C–H), 1508, 1406, 1165, 912, 815.

^{1}H NMR (400 MHz, CDCl$_3$) δ_H: 7.30 – 7.23 (4H, m, **4-H**), 7.19 – 7.13 (4H, m, **3-H**), 6.48 (1H, ddd, *J* 17.1, 10.6, 0.7, **7-H**), 5.36 – 5.26 (2H, m, **8-H**), 2.34 (6H, s, **1-H**), 2.22 – 2.19 (1H, m, **OH**).

^{13}C{^{1}H} NMR (126 MHz, CDCl$_3$) δ_C: 143.9 (**C7**), 143.2 (**C5**), 137.0 (**C2**), 129.0 (**C3**), 126.9 (**C4**), 113.7 (**C8**), 79.3 (**C6**), 21.2 (**C1**).

HRMS: (ESI$^+$) $C_{17}H_{17}$ [M–OH]$^+$ found 221.1322, requires 221.1325 (–1.4 ppm).

1,1-Bis(4-isopropylphenyl)prop-2-en-1-ol (**1d**)

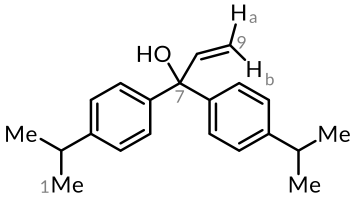

Following general procedure D, bis(4-isopropylphenyl)methanone (**7d**) (300 mg, 1.13 mmol), vinylmagnesium bromide (1 M in THF, 2.2 mL, 2.2 mmol), and THF (3 mL) for 2 h gave, after purification by column chromatography (hexane : dichloromethane, 70 : 30), allylic alcohol **1d** as a colourless oil (259 mg, 0.88 mmol, 78%).

ν_{max} (film): 3454 (O–H), 2958 (C–H), 2927, 1506, 1411, 827.

^{1}H NMR (500 MHz, $CDCl_3$) δ_H: 7.31 (4H, d, *J* 8.1, **5-H**), 7.19 (4H, d, *J* 8.1, **4-H**), 6.51 (1H, dd, *J* 17.1, 10.5, **H-8**), 5.34 (1H, d, *J* 17.1 **9b-H**), 5.29 (1H, d, *J* 9.8, **9a-H**), 2.91 (2H, hept, *J* 6.9, **2-H**), 2.24 (1H, s, **OH**), 1.25 (12H, d, *J* 6.9, **1-H**).

^{13}C{^{1}H} NMR (126 MHz, $CDCl_3$) δ_C: 147.9 (**C3**), 144.0 (**C8**), 143.4 (**C6**), 126.9 (**C5**), 126.2 (**C4**), 113.4 (**C9**), 79.2 (**C7**), 33.8 (**C2**), 24.1 (**C1**).

HRMS: (EI^+) $C_{17}H_{18}O_1$ $[M]^+$ found 294.1989, requires 294.1978 (+3.7 ppm).

1,1-Bis(4-(*tert*-butyl)phenyl)prop-2-en-1-ol (**1e**)

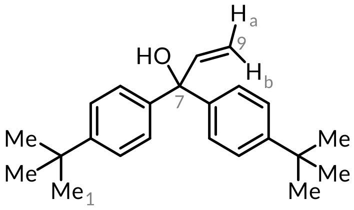

Following general procedure D, bis(4-(trimethylsilyl)phenyl)methanone (**7e**) (300 mg, 1.02 mmol), vinylmagnesium bromide (1 M in THF, 2.0 mL, 2.0 mmol), and THF (3 mL) for 2 h gave, after purification by column chromatography (hexane : dichloromethane, 70 : 30), allylic alcohol **1e** as a colourless oil (270 mg, 0.84 mmol, 82%).

ν_{max} (film): 3444 (O–H), 2960 (C–H), 2902, 1508, 1269, 829.

^{1}H NMR (500 MHz, $CDCl_3$) δ_H: 7.34 (4H, d, *J* 8.6, **4-H**), 7.31 (4H, d, *J* 8.6, **5-H**), 6.51 (1H, dd, *J* 17.1, 10.5, **8-H**), 5.34 (1H, d, *J* 17.1, **9b-H**), 5.28 (1H, d, *J* 10.5, **9a-H**), 2.22 (1H, s, **OH**), 1.31 (18H, s, **1-H**).

^{13}C{^{1}H} NMR (126 MHz, $CDCl_3$) δ_C: 150.1 (**C3**), 143.9 (**C8**), 142.9 (**C6**), 126.6 (**C5**), 125.1 (**C4**), 113.4 (**C9**), 79.1 (**C7**), 34.6 (**C2**), 31.4 (**C1**).

HRMS: (ESI$^+$) $C_{23}H_{30}NaO$ [M+Na]$^+$ found 345.2188, requires 345.2194 (–1.7 ppm).

1,1-Bi([1,1′-biphenyl]-4-yl)prop-2-en-1-ol (**1f**)

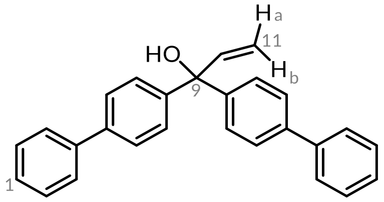

Following general procedure D, 1,1-di([1,1′-biphenyl]-4-yl)methanone (**7f**) (300 mg, 0.89 mmol), vinylmagnesium bromide (1 M in THF, 1.8 mL, 1.8 mmol), and THF (3 mL) for 2 h gave, after purification by column chromatography (hexane : dichloromethane, 50 : 50), allylic alcohol **1f** as a white solid (276 mg, 0.76 mmol, 85%).

ν_{max} (film): 3447, 3028, 1599, 1485, 1398, 1165, 1007, 837, 765.

^{1}H NMR (700 MHz, $CDCl_3$) δ_H: 7.62 – 7.57 (8H, m, **6-H**, **3-H**), 7.50 (4H, d, *J* 8.5, **7-H**), 7.44 (4H, app. t, *J* 7.6, **2-H**), 7.35 (2H, t, *J* 7.4, **1-H**), 6.59 (1H, dd, *J* 17.1, 10.6. **10-H**), 5.42 (1H, dd, *J* 17.1, 1.2, **11b-H**), 5.38 (1H, dd, *J* 10.6, 1.2, **11a-H**), 2.35 (1H, s, **OH**).

^{13}C{^{1}H} NMR (126 MHz, $CDCl_3$) δ_C: 144.9 (**C8**), 143.5 (**C10**), 140.8 (**C4**), 140.4 (**C5**), 128.9 (**C2**), 127.5 (**C1**, **C7**), 127.3 (**C3**), 127.1 (**C6**), 114.3 (**C11**), 79.3 (**C9**).

HRMS: (EI^+) $C_{27}H_{22}O$ $[M]^+$ found 362.1678, requires 362.1665 (+3.5 ppm).

1,1-Bis(4-bromophenyl)prop-2-en-1-ol (**1g**)

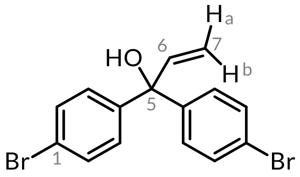

Following general procedure D, bis(4-bromorophenyl)methanone (680 mg, 2.00 mmol), vinylmagnesium bromide (1 M in THF, 5.0 mL, 5.0 mmol), and THF (4 mL) for 2 h gave, after purification by column chromatography (hexane : diethyl ether, 80 : 20), allylic alcohol **1g** as a colourless oil (673 mg, 1.83 mmol, 91%).

ν_{max} (film): 3422 (O–H), 1587 , 1395, 1072, 1009, 907, 814.

^{1}H NMR (500 MHz, CDCl$_3$) δ_H: 7.48 – 7.43 (4H, m, **2-H**), 7.26 – 7.20 (4H, m, **3-H**), 6.41 (1H, dd, *J* 17.1, 10.5 Hz, **6-H**), 5.35 (1H, d, *J* 10.6, **7a-H**), 5.29 (1H, d, *J* 17.1, **7b-H**), 2.25 (1H, s, **OH**).

^{13}C{^{1}H} NMR (126 MHz, CDCl$_3$) δ_C: δ 144.4 (**C4**), 142.6 (**C6**), 131.5 (**C2**), 128.8 (**C3**), 121.8 (**C1**), 115.2 (**C7**), 78.9 (**C5**).

HRMS: (ESI$^-$) $C_{15}H_{11}{}^{79}Br_2O$ [M–H]$^-$ found 364.9174, requires 364.9182 (–2.2 ppm).

1,1-Bis(4-chlorophenyl)prop-2-en-1-ol (**1h**)

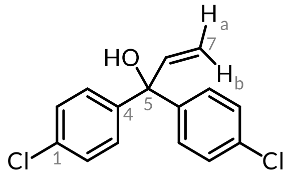

Following general procedure D, bis(4-chlorophenyl)methanone (377 mg, 1.50 mmol), vinylmagnesium bromide (1 M in THF, 2.3 mL, 2.3 mmol), and THF (3 mL) for 2 h gave, after purification by column chromatography (hexane : diethyl ether, 80 : 20), allylic alcohol **1h** as a colourless oil (385 mg, 1.38 mmol, 92%).

ν_{max} (film): 3445 (O–H), 3086 (C–H), 1487, 1396, 1092, 1015, 824.

^{1}H NMR (400 MHz, $CDCl_3$) δ_H: 7.30 (8H, s, **2-H**, **3-H**), 6.42 (1H, dd, *J* 17.1, 10.6, **6-H**), 5.29 (1H, d, *J* 17.1, **7b-H**), 5.29 (1H, dd, *J* 17.1, 1.0, **7a-H**), 2.23 (1H, s, **OH**).

^{13}C{^{1}H} NMR (126 MHz, $CDCl_3$) δ_C: 143.9 (**C4**), 142.8 (**C6**), 133.6 (**C1**), 128.5 (**C2/C3**), 128.4 (**C2/C3**), 115.1 (**C7**), 78.8 (**C5**).

HRMS: (ESI$^+$) $C_{15}H_{11}{}^{35}Cl_2O$ [M–H]$^-$ found 277.0190, requires 277.0192 (–0.7 ppm).

1,1-Bis(4-methoxyphenyl)prop-2-en-1-ol (**1i**)

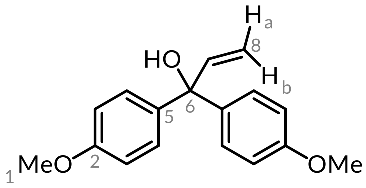

Following general procedure D, bis(4-methoxyphenyl)methanone (363 mg, 1.50 mmol), vinylmagnesium bromide (1 M in THF, 4.5 mL, 4.5 mmol), and THF (2 mL) for 2 h gave, after purification by column chromatography (hexane : diethyl ether, 60 : 40), allylic alcohol **1i** as a colourless oil (368 mg, 1.35 mmol, 90%).

ν_{max} (film): 3472 (O–H), 2953 (C–H), 2835 (C–H), 1607, 1506, 1246, 1175, 1034, 829.

^{1}H NMR (500 MHz, $CDCl_3$) δ_H: 7.31 – 7.26 (4H, m, **4-H**), 6.88 – 6.81 (4H, m, **3-H**), 6.46 (1H, dd, *J* 17.0, 10.6, **7-H**), 5.29 (1H, dd, *J* 11.4, 1.3, **8b-H**), 5.27 (1H, dd, *J* 4.9, 1.2, **8a-H**), 3.80 (6H, s, **1-H**), 2.19 (1H, m, **OH**).

^{13}C{^{1}H} NMR (126 MHz, $CDCl_3$) δ_C: 158.8 (**C2**), 144.0 (**C7**), 138.3 (**C5**), 128.3 (**C4**), 113.5 (**C8, C3**), 79.0 (**C6**), 55.4 (**C1**).

HRMS: (ESI$^+$) $C_{17}H_{18}O_3Na$ [M+Na]$^+$ found 293.1143, requires 293.1148 (−1.8 ppm).

1,1-Bis(4-phenoxyphenyl)prop-2-en-1-ol (**1j**)

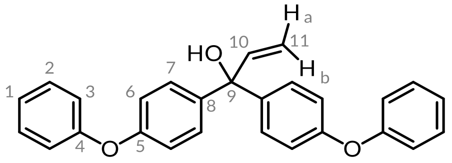

Following general procedure D, bis(4-phenoxyphenyl)methanone (366 mg, 1.00 mmol), vinylmagnesium bromide (1 M in THF, 2.5 mL, 2.5 mmol), and THF (2 mL) for 2 h gave, after purification by column chromatography (hexane : diethyl ether, 60 : 40), allylic alcohol **1j** as a colourless oil (316 mg, 0.80 mmol, 80%).

ν_{max} (film): 3462 (O–H), 3037 (C–H), 1587 (C=C), 1487, 1232, 1166, 869.

^{1}H NMR (500 MHz, CDCl$_3$) δ_H: 7.40 – 7.29 (8H, m, **7-H, 2-H**), 7.11 (2H, td, *J* 7.4, 1.2, **1-H**), 7.05 – 7.00 (4H, m, **3-H**), 6.99 – 6.94 (4H, m, **6-H**), 6.49 (1H, dd, *J* 17.0, 10.6 Hz, **10-H**), 5.34 (1H, d, *J* 8.2, 1.2, **11b-H**), 5.32 (1H, s, **11a-H**), 2.25 (1H, s, **OH**).

^{13}C{^{1}H} NMR (176 MHz, CDCl$_3$) δ_C: 157.1 (**C4**), 156.6 (**C5**), 143.7 (**C10**), 140.6 (**C8**), 129.9 (**C2**), 128.5 (**C7**), 123.5 (**C1**), 119.2 (**C3**), 118.3 (**C6**), 114.1 (**C11**), 79.0 (**C9**).

HRMS: (ESI$^+$) $C_{27}H_{22}O_3Na$ [M+Na]$^+$ found 417.1448, requires 417.1461 (–3.2 ppm).

1,1-Bis(4-(methylthio)phenyl)prop-2-en-1-ol (**1k**)

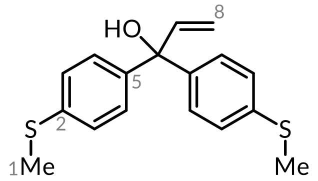

Following general procedure D, bis(4-(methylthio)phenyl)methanone (**7k**) (300 mg, 1.09 mmol), vinylmagnesium bromide (1 M in THF, 2.2 mL, 2.2 mmol), and THF (3 mL) for 2 h gave, after purification by column chromatography (hexane : dichloromethane, 50 : 50), allylic alcohol **1k** as a beige solid (259 mg, 0.86 mmol, 78%).

mp 140 – 142 °C

ν_{max} (film): 3446 (O–H), 2918 (C–H), 1595, 1490, 1093, 815.

^{1}H NMR (500 MHz, $CDCl_3$) δ_H: 7.28 (4H, d, *J* 8.5, **4-H**), 7.20 (4H, d, *J* 8.4, **3-H**), 6.44 (1H, dd, *J* 17.3, 10.3, **7-H**), 5.32 – 5.29 (2H, m, **8-H**), 2.47 (6H, s, **1-H**), 2.29 (1H, s, **OH**).

^{13}C{^{1}H} NMR (126 MHz, $CDCl_3$) δ_C: 143.2 (**C7**), 142.5 (**C5**), 137.6 (**C2**), 127.5 (**C4**), 126.2 (**C3**), 114.3 (**C8**), 79.0 (**C6**), 15.8 (**C1**).

HRMS: (ESI^+) $C_{17}H_{18}NaOS_2$ $[M+Na]^+$ found 325.0693, requires 325.0697 (–1.2 ppm).

1,1-Bis(4-((trimethylsilyl)ethynyl)phenyl)prop-2-en-1-ol (**1l**)

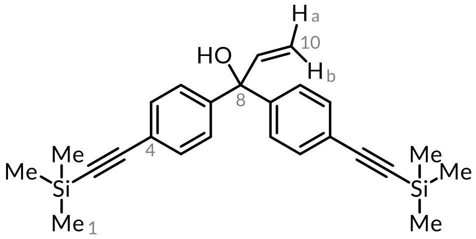

Following general procedure D, bis(4-(trimethylsilyl)phenyl)methanone (**7l**) (350 mg, 0.94 mmol), vinylmagnesium bromide (1 M in THF, 1.9 mL, 1.9 mmol), and THF (3 mL) for 2 h gave, after purification by column chromatography (hexane : dichloromethane, 70 : 30), allylic alcohol **1l** as a beige solid (303 mg, 0.75 mmol, 81%).

mp 145 – 147 °C

ν_{max} (film): 3491 (O-H), 2958 (C-H), 2158 (C≡C), 1498, 1249, 840.

^{1}H NMR (500 MHz, $CDCl_3$) δ_H: 7.42 – 7.40 (4H, m, **5-H**), 7.28 – 7.27 (4H, m, **6-H**), 6.42 (1H, dd, *J* 17.1, 10.6, **9-H**), 5.33 (1H, d, *J* 10.6, **10a-H**), 5.27 (1H, d, *J* 17.1, **10b-H**), 2.24 (1H, s, **OH**), 0.24 (18H, s, **1-H**).

^{13}C{^{1}H} NMR (126 MHz, $CDCl_3$) δ_C: 145.7 (**C3**), 142.7 (**C9**), 131.9 (**C5**), 126.9 (**C6**), 122.4 (**C7**), 115.0 (**C10**), 104.8 (**C4**), 94.7 (**C2**), 79.2 (**C8**), 0.1 (**C1**).

HRMS: (ESI$^+$) $C_{25}H_{30}NaOSi_2$ [M+Na]$^+$ found 425.1730, requires 425.1733 (–0.7 ppm).

1,1-Bis(4-(trifluoromethyl)phenyl)prop-2-en-1-ol (**1m**)

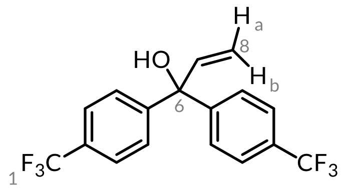

Following general procedure D, bis(4-(trifluoromethyl)phenyl)methanone (**7m**) (477 mg, 1.50 mmol), vinylmagnesium bromide (1 M in THF, 4.5 mL, 4.5 mmol), and THF (3 mL) for 2 h gave, after purification by column chromatography (hexane : diethyl ether, 80 : 20), allylic alcohol **1m** as a colourless oil (452 mg, 1.31 mmol, 87%).

ν_{max} (film): 3431 (O–H), 1412, 1323, 1163, 1123, 1069, 1016, 837.

^{1}H NMR (500 MHz, $CDCl_3$) δ_H: 7.60 (4H, d, *J* 8.2, **3-H**), 7.51 (4H, d, *J* 8.2, **4-H**), 6.50 (1H, dd, *J* 17.1, 10.6, **7-H**), 5.43 (1H, d, *J* 10.6, **8a-H**), 5.34 (1H, d, *J* 17.1, **8b-H**), 2.36 (1H, s, **OH**).

^{13}C{^{1}H} NMR (126 MHz, $CDCl_3$) δ_C: 149.0 (**C5**), 142.2 (**C7**), 130.0 (q, *J* 32.6, **C2**), 127.3 (**C4**), 125.5 (q, *J* 3.8, **C3**), 124.2 (q, *J* 272.2, **C1**), 116.1 (**C8**), 79.1 (**C6**).

^{19}F NMR (282 MHz, CDCl3) δ_F: –62.6 (**CF_3**).

HRMS: (ESI$^-$) $C_{17}H_{11}F_6O$ [M–H]$^-$ found 345.0719, requires 345.0720 (–0.2 ppm).

1,1-Bis(3-(trifluoromethyl)phenyl)prop-2-en-1-ol (**1n**)

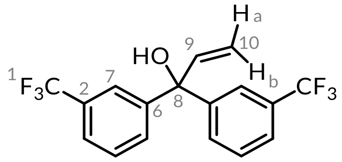

Following general Procedure D, bis(4-(trifluomethyl)phenyl)methanone (477 mg, 1.50 mmol), vinylmagnesium bromide (1 M in THF, 3.8 mL, 3.8 mmol), and THF (2 mL) for 2 h gave, after purification by column chromatography (hexane : diethyl ether, 80 : 20), allylic alcohol **1n** as a colourless oil (475 mg, 1.37 mmol, 91%).

ν_{max} (film): 3445 (O–H), 3069 (C–H), 1611, 1587, 1483, 1438, 1232, 862, 812, 783.

^{1}H NMR (500 MHz, $CDCl_3$) δ_H: 7.72 (2H, s, **7-H**), 7.57 (2H, d, *J* 7.5, **3-H**), 7.50 (2H, d, *J* 7.5, **5-H**), 7.46 (2H, t, *J* 7.7, **4-H**), 6.49 (1H, dd, *J* 17.1, 10.6, **9-H**), 5.43 (1H, app. d, *J* 10.5, **10a-H**), 5.34 (1H, app. d, *J* 17.1, **10b-H**), 2.41 (1H, s, **OH**).

^{13}C{^{1}H} NMR (126 MHz, $CDCl_3$) δ_C: 146.1 (**C6**), 142.2 (**C9**), 130.9 (q, J_{CF} 32.3, **C2**) 130.5 (**C5**), 129.0 (**C4**), 124.7 (q, J_{CF} 3.8, **C3**), 124.2 (q, J_{CF} 272.5, **C1**), 123.56 (q, J_{CF} 3.9, **C7**), 116.1 (**C10**), 79.0 (**C8**).

^{19}F NMR (282 MHz, $CDCl_3$) δ_F: –62.5 (**CF_3**).

HRMS: (ESI^-) $C_{17}H_{11}OF_6$ $[M–H]^-$ found 345.0726, requires 345.0720 (–1.7 ppm).

1,1-Bis(3-chlorophenyl)prop-2-en-1-ol (**1o**)

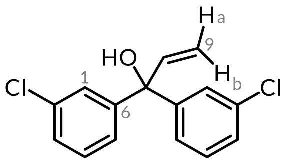

Following general procedure D, bis(3-chlorophenyl)methanone (**7o**) (377 mg, 1.50 mmol), vinylmagnesium bromide (1 M in THF, 4.5 mL, 4.5 mmol), and THF (3 mL) for 2 h gave, after purification by column chromatography (hexane : diethyl ether, 80 : 20), allylic alcohol **1o** as a colourless oil (401 mg, 1.44 mmol, 96%).

ν_{max} (film): 3441 (O–H), 3064 (C–H), 1593, 1572, 1474, 1420, 1171, 1080, 999, 932, 885.

^{1}H NMR (500 MHz, $CDCl_3$) δ_H: 7.43 (2H, s, **1-H**), 7.34 – 7.27 (4H, m, **3-H, 4-H**), 7.27 – 7.22 (2H, m, **5-H**), 6.46 (1H, dd, *J* 17.1, 10.6, **8-H**), 5.40 (1H, d, *J* 10.6, **9a-H**), 5.35 (1H, d, *J* 17.1, **9b-H**), 2.31 (1H, s, **OH**).

^{13}C{^{1}H} NMR (126 MHz, $CDCl_3$) δ_C: 147.3 (**C6**), 142.4 (**C8**), 134.5 (**C2**), 129.7 (**C4**), 127.9 (**C3**), 127.1 (**C1**), 125.2, (**C5**), 115.5 (**C9**), 78.9 (**C7**).

HRMS: (ESI$^+$) $C_{15}H_{12}{}^{35}Cl_2NaO$ $[M+Na]^+$ found 301.0157, requires 301.0157 (0.0 ppm).

1,1-Bis(3-fluorophenyl)prop-2-en-1-ol (**1p**)

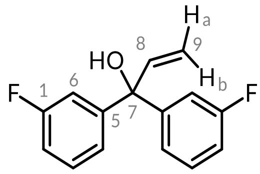

Following general procedure D, bis(3-fluorophenyl)methanone (327 mg, 1.50 mmol), vinylmagnesium bromide (1 M in THF, 2.25 mL, 2.25 mmol), and THF (2 mL) for 2 h gave, after purification by column chromatography (hexane : acetone, 99 : 1 to 80 : 20), allylic alcohol **1p** as a colourless oil (279 mg, 1.13 mmol, 75%).

ν_{max} (film): 3444 (O–H), 3068 (C–H), 1610, 1508, 1483, 1438, 1232, 862, 783.

^{1}H NMR (400 MHz, $CDCl_3$) δ_H: 7.33 – 7.27 (2H, m, **3-H**), 7.14 – 7.10 (4H, m, **4-H**, **6-H**), 7.00 – 6.95 (2H, m, **2-H**), 6.45 (1H, dd, *J* 17.1, 10.6, **8-H**), 5.37 (1H, dd, *J* 10.6, 0.7, **7a-H**), 5.33 (1H, dd, *J* 17.1, 0.7, **7b-H**), 2.34 (1H, s, **OH**).

^{13}C{^{1}H} NMR (126 MHz, $CDCl_3$) δ_C: 162.8 (d, *J* 246.1, **C1**), 147.9 (d, *J* 6.5, **C5**), 142.5 (**C8**), 129.9 (d, *J* 8.1, **C3**), 122.6 (d, *J* 2.9, **C4**), 115.3 (**C9**), 114.5 (d, *J* 21.1, **C2**), 114.1 (d, *J* 22.6, **C6**), 78.8 (**C7**).

^{19}F NMR (471 MHz, $CDCl_3$) δ_F: –112.4 – –112.5 (m, **F**).

HRMS: (ESI^-) $C_{15}H_{11}OF_2$ $[M–H]^-$ found 245.0780, requires 245.0783 (–1.4 ppm).

1,1-di-*m*-tolylprop-2-en-1-ol (**1q**)

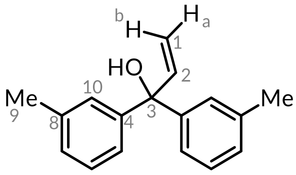

Following general procedure H, di-*m*-tolylmethanone (460 mg, 2.2 mmol), vinylmagnesium bromide (1 M in THF, 4.4 mL, 4.4 mmol), and THF (5 mL) for 2 h gave, after purification by column chromatography (hexane : dichloromethane, 60 : 40), allylic alcohol **1q** as a colourless oil (420 mg, 1.8 mmol, 80%). Spectroscopic data were in accordance with the literature.[12]

ν_{max} (film): 3456 (O–H), 3022 (C–H), 2920 (C–H), 1604, 1483, 1145, 704.

^{1}H NMR (500 MHz, $CDCl_3$) δ_H: 7.23 – 7.20 (4H, m, **6-H, 10-H**), 7.16 – 7.14 (2H, m, **5-H**), 7.09 – 7.08 (2H, m, **7-H**), 6.50 (1H, dd, *J* 17.1, 10.6, **2-H**), 5.34 (1H, dd, *J* 17.1, 1.2, **1b-H**), 5.30 (1H, dd, *J* 10.6, 1.2, **1a-H**), 2.39 (6H, s, **9-H**), 2.24 (1H, s, **OH**).

^{13}C{^{1}H} NMR (126 MHz, $CDCl_3$) δ_C: 145.9 (**C4**), 143.8 (**C2**), 137.9 (**C8**), 128.15 (**C7**), 128.14 (**C6**), 127.5 (**C10**), 124.1 (**C5**), 113. 7 (**C1**), 79.4 (**C3**), 21.7 (**C9**).

HRMS: (ESI$^+$) $C_{17}H_{17}$ [M–OH]$^+$ found 221.1329, requires 221.1325 (+1.8 ppm).

1,1-bis-(3-methoxyphenyl)prop-2-en-1-ol (**1r**)

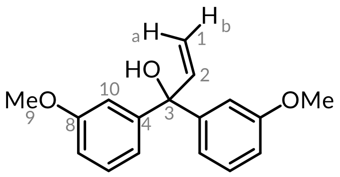

Following general procedure D, bis(3-methoxyphenyl)methanone (400 mg, 1.7 mmol), vinylmagnesium bromide (1 M in THF, 3.4 mL, 3.4 mmol), and THF (5 mL) for 2 h gave, after purification by column chromatography (hexane : dichloromethane, 50 : 50), allylic alcohol **1r** as a colourless oil (360 mg, 1.3 mmol, 81%).

ν_{max} (film): 3477 (O–H), 2939 (C–H), 2835 (C–H), 1597, 1485, 1047, 779.

^{1}H NMR (400 MHz, CDCl$_3$) δ_H: 7.25 – 7.22 (2H, m, **6-H**), 6.99 – 6.98 (2H, m, **10-H**), 6.95 – 6.93 (2H, m, **5-H**), 6.82 – 6.79 (2H, m, **7-H**), 6.48 (1H, dd, *J* 17.1, 10.6, **2-H**), 5.34 (1H, dd, *J* 17.5, 1.3, **1a-H**), 5.31 (1H, dd, *J* 10.5, 1.0, **1b-H**), 3.78 (6H, s, **9-H**), 2.26 (1H, s, **OH**).

^{13}C{^{1}H} NMR (126 MHz, CDCl$_3$) δ_C: 159.6 (**C8**), 147.4 (**C4**), 143.3 (**C2**), 129.2 (**C6**), 119.4 (**C5**), 114.2 (**C1**), 112.9 (**C10**), 112.7 (**C7**), 79.3 (**C3**), 55.3 (**C9**).

HRMS: (ESI$^+$) $C_{17}H_{19}O_3$ [M+H]$^+$ found 271.1338, requires 271.1329 (+3.3 ppm).

1,1-Bis(2-chlorophenyl)prop-2-en-1-ol (**1s**)

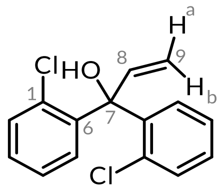

Following general procedure D, bis(2-chlorophenyl)methanone (251 mg, 1.00 mmol), vinylmagnesium bromide (1 M in THF, 2.5 mL, 2.5 mmol), and THF (2 mL) for 2 h gave, after purification by column chromatography (hexane : diethyl ether, 80 : 20), allylic alcohol **1s** as a colourless oil (269 mg, 0.96 mmol, 96%).

ν_{max} (film): 3566 (O–H), 3456 (O–H), 3062 (C–H), 2363, 1589, 1465, 1269, 920.

^{1}H NMR (500 MHz, CDCl$_3$) δ_H: 7.75 (2H, dd, *J* 8.2, 1.8, **5-H**), 7.32 – 7.27 (4H, m, **4-H, 2-H**), 7.25 – 7.21 (2H, m, **3-H**), 6.68 (1H, ddd, *J* 17.1, 10.7, 1.0, **8-H**), 5.54 (1H, dd, *J* 17.1, 1.3, **9b-H**), 5.36 (1H, dd, *J* 10.6, 1.3, **9a-H**), 3.33 (1H, d, *J* 1.0, **OH**).

^{13}C{^{1}H} NMR (126 MHz, CDCl$_3$) δ_C: 142.1 (**C6**), 139.7 (**C8**), 131.6 (**C1**), 131.1 (**C2**), 129.5 (**C5**), 129.0 (**C3**), 126.9 (**C4**), 114.7 (**C9**), 78.9 (**C7**).

HRMS: (ESI$^+$) $C_{15}H_{12}{}^{35}Cl_2NaO$ [M+Na]$^+$ found 301.0149, requires 301.0157 (–2.7 ppm).

1,1-Di-*o*-tolylprop-2-en-1-ol (**1t**)

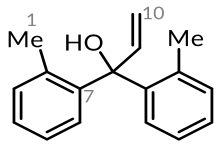

Following general procedure D, 2,2′-dimethylbenzophenone (**7t**) (350 mg, 1.66 mmol), vinylmagnesium bromide (1 M in THF, 2.3 mL, 2.3 mmol), and THF (3 mL) for 2 h gave, after purification by column chromatography (hexane : dichloromethane, 70 : 30), allylic alcohol **1t** as a colourless oil (287 mg, 1.20 mmol, 72%).

ν_{max} (film): 3547 (O–H), 3059 (C–H), 2978 (C–H), 1485, 1456, 997.

^{1}H NMR (500 MHz, $CDCl_3$) δ_H: 7.52 – 7.50 (2H, m, **6-H**), 7.20 – 7.16 (4H, m, **4-H**, **5-H**), 7.10 – 7.09 (2H, m, **3-H**), 6.55 (1H, d, *J* 17.1, 10.5, **9-H**), 5.33 – 5.27 (2H, m, **10-H**), 2.17 (1H, s, **OH**), 1.99 (6H, s, **1-H**).

^{13}C{^{1}H} NMR (126 MHz, $CDCl_3$) δ_C: 143.2 (**C2**), 142.6 (**C9**), 136.6 (**C7**), 132.4 (**C3**), 127.6 (**C4**), 127.3 (**C6**), 125.7 (**C5**), 113.1 (**C10**), 80.5 (**C8**), 21.6 (**C1**).

HRMS: (EI$^+$) $C_{17}H_{18}O_1$ [M]$^+$ found 238.1349, requires 238.1352 (–1.2 ppm).

1,1-Bis(2-methoxyphenyl)prop-2-en-1-ol (**1u**)

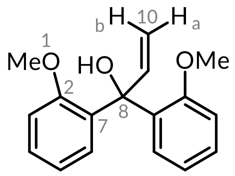

Following general procedure D, bis(2-methoxyphenyl)methanone (**7u**) (250 mg, 1.03 mmol), vinylmagnesium bromide (1 M in THF, 2.0 mL, 2.0 mmol), and THF (3 mL) for 2 h gave, after purification by column chromatography (hexane : dichloromethane, 75 : 25), allylic alcohol **1u** as a white solid (223 mg, 0.83 mmol, 80%).

mp 84 – 86 °C

ν_{max} (film): 3525 (O–H), 2937 (C–H), 1597, 1485, 1236, 1026.

^{1}H NMR (500 MHz, $CDCl_3$) δ_H: 7.48 (2H, d, *J* 7.8, **6-H**), 7.23 (2H, t, *J* 7.8, **4-H**), 6.96 (2H, t, *J* 7.6, **5-H**), 6.85 – 6.83 (2H, m, **3-H**), 6.67 (1H, dd, *J* 17.2, 10.6, **9-H**), 5.36 (1H, d, *J* 17.2, **10b-H**), 5.24 (1H, d, *J* 10.6, **10a-H**), 4.77 (1H, s, **OH**), 3.55 (6H, s, **1-H**).

^{13}C{^{1}H} NMR (126 MHz, $CDCl_3$) δ_C: 156.7 (**C2**), 141.5 (**C9**), 134.5 (**C7**), 128.3 (**C4**), 128.0 (**C6**), 120.6 (**C5**), 113.4 (**C10**), 112.3 (**C3**), 77.8 (**C8**), 55.7 (**C1**).

HRMS: (EI^+) $C_{17}H_{18}O_3$ $[M]^+$ found 270.1241, requires 270.1250 (–3.3 ppm).

1,1-Bis(2-fluoroyphenyl)prop-2-en-1-ol (**1v**)

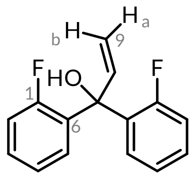

Following general procedure D, bis(2-fluorophenyl)methanone (**7v**) (250 mg, 1.15 mmol), vinylmagnesium bromide (1 M in THF, 2.2 mL, 2.2 mmol), and THF (3 mL) for 2 h gave, after purification by column chromatography (hexane : dichloromethane, 70 : 30), allylic alcohol **1v** as a colourless oil (225 mg, 0.91 mmol, 80%).

ν_{max} (film): 3591 (O–H), 3068 (C–H), 1581, 1485, 1219, 914.

^{1}H NMR (500 MHz, $CDCl_3$) δ_H: 7.55 – 7.52 (2H, m, **5-H**), 7.32 – 7.27 (2H, m, **3-H**), 7.17 – 7.14 (2H, m, **4-H**), 7.02 – 6.98 (2H, m, **2-H**), 6.61 (1H, dd, *J* 17.2, 10.6, **8-H**), 5.42 (1H, d, *J* 17.2, **9b-H**), 5.36 (1H, d, *J* 10.6, **9a-H**), 2.98 (1H, s, **OH**).

^{13}C{^{1}H} NMR (126 MHz, $CDCl_3$) δ_C: 160.2 (d, *J* 247.2, **C1**), 140.3 (**C8**), 132.0 (d, *J* 10.5, **C6**), 129.7 (d, *J* 8.9, **C3**), 128.1 (**C5**), 124.1 (**C4**), 116. 2 (d, *J* 22.1, **C2**), 114.7 (**C9**), 76.2 (**C7**).

^{19}F NMR (282 MHz, $CDCl_3$) δ_F: –111.8 (**F**).

HRMS: (EI^+) $C_{15}H_{12}OF_2$ $[M]^+$ found 246.0849, requires 246.0850 (–0.4 ppm).

1,1-Di(benzofuran-2-yl)prop-2-en-1-ol (**1w**)

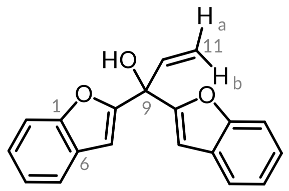

Following general procedure D, di(benzofuran-2-yl)methanone (**7w**) (300 mg, 1.14 mmol), vinylmagnesium bromide (1 M in THF, 2.2 mL, 2.2 mmol), and THF (3 mL) for 2 h gave, after purification by column chromatography (hexane : dichloromethane, 60 : 40), allylic alcohol **1w** as a white solid (217 mg, 0.75 mmol, 65%).

ν_{max} (film): 3427 (O–H), 3064 (C–H), 1452, 1251, 1159, 987.

^{1}H NMR (500 MHz, CDCl$_3$) δ_H: 7.57 (2H, d, *J* 7.6, **5-H**), 7.47 (2H, d, *J* 8.2, **2-H**), 7.31 – 7.28 (2H, m, **3-H**), 7.24 (2H, t, *J* 7.5, **4-H**), 6.76 (2H, s, **7-H**), 6.57 (1H, dd, *J* 17.1, 10.5, **10-H**), 5.61 (1H, d, *J* 17.1, **11b-H**), 5.51 (1H, d, *J* 10.5, **11a-H**), 3.11 (1H, s, **OH**).

^{13}C{^{1}H} NMR (126 MHz, CDCl$_3$) δ_C: 157.0 (**C8**), 155.2 (**C1**), 137.2 (**C10**), 128.0 (**C6**), 124.7 (**C3**), 123.1 (**C4**), 121.5 (**C5**), 117.1 (**C11**), 111.6 (**C2**), 104.8 (**C7**), 73.1 (**C9**).

HRMS: (ESI$^+$) $C_{19}H_{14}NaO_3$ [M+Na]$^+$ found 313.0837, requires 313.0841 (–1.2 ppm).

1,1-Bis(benzothiophen-2-yl)prop-2-en-1-ol (**1x**)

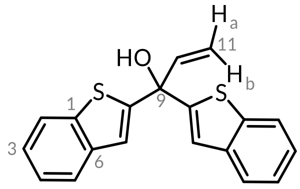

Following general procedure D, bis(benzothiophen-2-yl)methanone (**7x**) (300 mg, 1.02 mmol), vinylmagnesium bromide (1 M in THF, 2.0 mL, 2.0 mmol), and THF (3 mL) for 2 h gave, after purification by column chromatography (hexane : dichloromethane, 60 : 40), allylic alcohol **1x** as a yellow oil (289 mg, 0.90 mmol, 88%).

ν_{max} (film): 3517 (O–H), 3055 (C–H), 1458, 935.

^{1}H NMR (500 MHz, $CDCl_3$) δ_H: 7.81 – 7.80 (2H, m, **2-H**), 7.72 – 7.71 (2H, m, **5-H**), 7.36 – 7.31 (4H, m, **3-H**, **4-H**), 7.28 (2H, s, **7-H**), 6.62 (1H, dd, *J* 17.0, 10.4, **10-H**), 5.60 (1H, d, *J* 17.0, **11b-H**), 5.45 (1H, d, *J* 10.4, **11a-H**), 2.88 (1H, s, **OH**).

^{13}C{^{1}H} NMR (126 MHz, $CDCl_3$) δ_C: 149.9 (**C8**), 141.4 (**C10**), 140.1 (**C1**), 139.4 (**C6**), 124.7 (**C3**), 124.5 (**C4**), 124.0 (**C5**), 122.5 (**C2**), 122.3 (**C7**), 115.4 (**C11**), 76.6 (**C9**).

HRMS: (EI^+) $C_{19}H_{14}OS_2$ $[M]^+$ found 322.0475, requires 322.0480 (–1.5 ppm).

1,1-Di(thiophen-2-yl)prop-2-en-1-ol (**1y**)

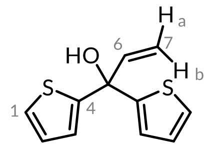

Following general procedure D, di-2-thienyl ketone (194 mg, 1.00 mmol), vinylmagnesium bromide (1 M in THF, 2.5 mL, 2.5 mmol), and THF (2 mL) for 2 h gave, after purification by column chromatography (hexane : diethyl ether, 80 : 20), allylic alcohol **1y** as a yellow oil (207 mg, 0.93 mmol, 93%).

ν_{max} (film): 3524 (O–H), 3102 (C–H), 1638, 1433, 1404, 1231, 986, 930, 831, 810.

^{1}H NMR (400 MHz, $CDCl_3$) δ_H: 7.30 – 7.27 (2H, m, **2-H**), 6.99 – 6.96 (4H, m, **1-H**, **3-H**), 6.52 (1H, dd, *J* 16.9, 10.4, **6-H**), 5.48 (1H, dd, *J* 16.9, 0.8, **7b-H**), 5.33 (1H, dd, *J* 10.4, 0.9, **7a-H**), 2.71 (1H, s, **OH**).

^{13}C{^{1}H} NMR (126 MHz, $CDCl_3$) δ_C: 150.2 (**C4**), 142.5 (**C6**), 126.8 (**C1**/**C3**), 125.7 (**C2**), 125.5 (**C1**/**C3**), 114.1 (**C7**), 76.0 (**C5**).

HRMS: (ESI$^+$) $C_{11}H_{10}OS_2Na$ $[M+Na]^+$ found 245.0064, requires 245.0065 (–0.4 ppm).

1,1-Bis(6-methoxynaphthalen-2-yl)prop-2-en-1-ol (**1z**)

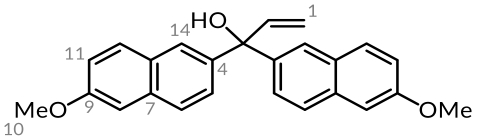

Following general procedure D, bis(6-methoxynaphthalen-2-yl)methanone (**7z**) (300 mg, 0.88 mmol), vinylmagnesium bromide (1 M in THF, 1.8 mL, 1.76 mmol), and THF (3 mL) for 2 h gave, after purification by column chromatography (hexane : dichloromethane, 50 : 50), allylic alcohol **1z** as a white solid (244 mg, 0.66 mmol, 75%).

mp 140 – 142 °C

ν_{max} (film): 3483 (O–H), 2954 (C–H), 1604, 1263, 1029, 852.

^{1}H NMR (500 MHz, $CDCl_3$) δ_H: 7.86 (2H, s, **14-H**), 7.72 – 7.67 (4H, m, **6-H, 12-H**), 7.43 – 7.41 (2H, m, **5-H**), 7.16 – 7.12 (4H, m, **8-H, 11-H**), 6.68 (1H, dd, *J* 17.1, 10.6, **2-H**), 5.44 – 5.39 (2H, m, **1-H**), 3.92 (6H, s, **10-H**), 2.42 (1H, s, **OH**).

^{13}C{^{1}H} NMR (126 MHz, $CDCl_3$) δ_C: 158.0 (**C9**), 143.6 (**C2**), 140.9 (**C4**), 133.9 (**C7**), 129.9 (**C12**), 128.5 (**C13**), 127.0 (**C6**), 126.2 (**C5**), 125.3 (**C14**), 119.0 (**C11**), 114.3 (**C1**), 105.7 (**C8**), 79.7 (**C3**), 55.4 (**C10**).

HRMS: (ESI$^+$) $C_{25}H_{22}NaO_3$ $[M+Na]^+$ found 393.1460, requires 393.1467 (–1.7 ppm).

2-Methylene-1,1-diphenylbutan-1-ol (**1aa**)

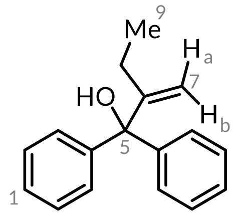

A freshly made Grignard solution from 2-bromo-1-butene (607 mg, 4.50 mmol) using magnesium turnings (109 mg, 4.50 mmol) in THF (2 mL) was added to benzophenone (247 mg, 1.50 mmol) in THF (1 mL) at 0 °C and stirred for 2. The reaction was quenched with saturated aqueous ammonium chloride and the aqueous layer was extracted with diethyl ether (x 3). The organic layers were combined, washed with brine, dried over Na_2SO_4, filtered and concentrated under reduced pressure. The crude product was purification by silica gel column chromatography (hexane : ethyl acetate, 98 : 2 to 80 : 20) to afford allylic alcohol **1aa** as a white solid (141 mg, 0.59 mmol, 40%). NMR spectroscopic data were in accordance with the literature.[13]

mp 64-66 °C

^{1}H NMR (400 MHz, $CDCl_3$) δ_H: 7.38 – 7.23 (10H, m, **1-H, 2-H, 3-H**), 5.18 (1H, s, **7a-H/7b-H**), 4.83 (1H, s, **7a-H/7b-H**), 2.48 (1H, s, **OH**), 2.10 (2H, q, *J* 7.5, **8-H**), 1.06 (3H, t, *J* 7.4, **9-H**).

^{13}C{^{1}H} NMR (126 MHz, $CDCl_3$) δ_C: 155.1 (**C6**), 145.4 (**C4**), 128.0 (**C2/C3**), 127.9 (**C2/C3**), 127.4 (**C1**), 113.0 (**C7**), 83.7 (**C5**), 25.0 (**C8**), 12.8 (**C9**).

(*Z*)-1,1-diphenylbut-2-en-1-ol (**1ab**)

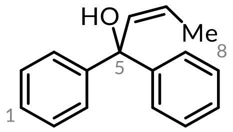

A freshly made Grignard solution from 1-bromo-1-propene (607 mg, 4.50 mmol) using magnesium turnings (109 mg, 4.50 mmol) in THF (2 mL) was added to benzophenone (247 mg, 1.50 mmol) in THF (1 mL) at 0 °C and stirred for 2. The reaction was quenched with saturated aqueous ammonium chloride and the aqueous layer was extracted with diethyl ether (x 3). The organic layers were combined, washed with brine, dried over Na_2SO_4, filtered and concentrated under reduced pressure. The crude product was purification by silica gel column chromatography (hexane : dichloromethane, 90 : 10 to 60 : 40) to afford allylic alcohol **1ab** as a colourless oil (210 mg, 0.93 mmol, 62%). Characterisation data were in accordance with the literature.[14]

Note: isolated as a 99 : 1 ratio of *Z*/*E* isomers.

ν_{max} (film): 3443 (O–H), 3024 (C–H), 2914 (C–H), 1597, 1489, 1447, 976, 760, 698.

^{1}H NMR (700 MHz, $CDCl_3$) δ_H: 7.45 (4H, d, *J* 7.2, **3-H**), 7.31 (4H, t, *J* 7.8, **2-H**), 7.23 (2H, t, *J* 7.3, **1-H**), 6.15 (1H, dd, *J* 11.6, 1.9, **6-H**), 5.81 (1H, dq, *J* 11.6, 7.3, **7-H**), 2.36 (1H, s, **OH**), 1.56 (3H, dd, *J* 7.3, 1.8, **8-H**).

^{13}C{^{1}H} NMR (126 MHz, $CDCl_3$) δ_C: 147.7 (**C5**), 137.1 (**C6**), 129.3 (**C7**), 128.3 (**C2**), 127.0 (**C1**), 126.5 (**C3**), 79.1 (**C5**), 14.9 (**C8**).

1,1-Bis(4-(pyridine-4-yl)phenyl)prop-2-en-1-ol (**1za**)

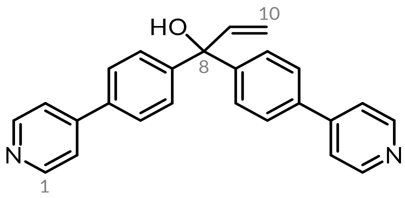

Following general procedure D, bis(4-(pyridin-4-yl)phenyl)methanone (**7za**) (300 mg, 0.89 mmol), vinylmagnesium bromide (1 M in THF, 1.8 mL, 1.8 mmol), and THF (3 mL) for 2 h gave, after purification by column chromatography (ethyl acetate : methanol, 99 : 1), allylic alcohol **1za** as a white solid (259 mg, 0.72 mmol, 80%).

mp 188 – 190 °C

ν_{max} (film): 3138 (O–H), 3032 (C–H), 1597, 1398, 910.

^{1}H NMR (500 MHz, CDCl$_3$) δ_H: 8.57 (4H, d, *J* 6.1, **1-H**), 7.61 – 7.59 (4H, m, **5-H**), 7.57 – 7.55 (4H, m, **6-H**), 7.48 – 7.46 (4H, m, **2-H**), 6.58 (1H, dd, *J* 17.1, 10.5, **9-H**), 5.44 – 5.38 (2H, m, **10-H**).

^{13}C{^{1}H} NMR (126 MHz, CDCl$_3$) δ_C: 150.1 (**C1**), 148.0 (**C3**), 147.0 (**C7**), 143.2 (**C9**), 137.0 (**C4**), 127.8 (**C6**), 126.9 (**C5**), 121.6 (**C2**), 114.9 (**C10**), 78.9 (**C8**).

HRMS: (ESI$^+$) $C_{25}H_{21}N_2O$ [M+H]$^+$ found 365.1647, requires 365.1654 (–1.9 ppm).

(±)-1-Phenyl-1-(p-tolyl)prop-2-en-1-ol (**(±)-3a**)

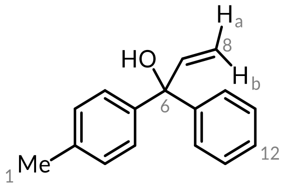

Following general procedure D, 4-methylbenzophenone (294 mg, 1.50 mmol), vinylmagnesium bromide (1 M in THF, 3.0 mL, 3.0 mmol), and THF (3 mL) for 2 h gave, after purification by column chromatography (hexane : diethyl ether, 90 : 10), allylic alcohol **(±)-3a** as a colourless oil (289 mg, 1.29 mmol, 86%).

ν_{max} (film): 3441 (O–H), 3024 (C–H), 2918 (C–H), 1510, 1447, 1165, 995, 922, 816, 760.

^{1}H NMR (500 MHz, $CDCl_3$) δ_H: 7.41 (2H, d, *J* 8.1, **10-H**), 7.34 (2H, app. t, *J* 7.5, **11-H**), 7.31 – 7.25 (3H, m, **4-H**, **12-H**), 7.16 (2H, d, *J* 7.9, **3-H**), 6.52 (1H, dd, *J* 17.1, 10.6, **7-H**), 5.34 (1H, d, *J* 13.5, **8b-H**), 5.32 (1H, d, *J* 6.8, **8a-H**), 2.36 (3H, s, **1-H**), 2.29 (1H, d, *J* 2.4, **OH**).

^{13}C{^{1}H} NMR (126 MHz, $CDCl_3$) δ_C: 146.0 (**C9**), 143.8 (**C7**), 143.1 (**C5**), 137.1 (**C2**), 129.0 (**C3**), 128.2 (**C11**), 127.3 (**C12**), 127.0 (**C10**, **C4**), 113.9 (**C8**), 79.4 (**C6**), 21.2 (**C1**).

HRMS: (ESI$^+$) $C_{16}H_{16}ONa$ [M+Na]$^+$ found 247.1095, requires 247.1093 (+0.7 ppm).

(±)-1-(4-Fluorophenyl)-1-phenylprop-2-en-1-ol ((**±)-3b**)

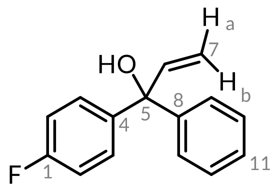

Following general procedure D, 4-fluorobenzophenone (400 mg, 2.00 mmol), vinylmagnesium bromide (1 M in THF, 5.0 mL, 5.0 mmol), and THF (2 mL) for 2 h gave, after purification by column chromatography (hexane : diethyl ether, 80 : 20), allylic alcohol **(±)-3b** as a colourless oil (426 mg, 1.87 mmol, 93%).

ν_{max} (film): 3444 (O–H), 3059 (C–H), 1601, 1506, 1447, 1223, 1159, 833, 762.

^{1}H NMR (500 MHz, $CDCl_3$) δ_H: 7.39 – 7.31 (6H, m, **3-H**, **9-H**, **10-H**), 7.30 – 7.25 (1H, m, **11-H**), 7.05 – 6.95 (2H, m, **2-H**), 6.48 (1H, dd, *J* 17.1, 10.6, **6-H**), 5.33 (1H, dd, *J* 5.5, 1.1, **7a-H**), 5.30 (1H, dd, *J* 11.8, 1.1, **7b-H**), 2.26 (1H, d, *J* 1.0, **OH**).

^{13}C{^{1}H} NMR (126 MHz, $CDCl_3$) δ_C: 162.1 (d, *J* 246.1, **C1**), 145.7 (**C8**), 143.5 (**C6**), 141.6 (d, *J* 3.2, **C4**), 128.9 (d, *J* 8.1, **C3**), 128.4 (**C10**), 127.6 (**C11**), 126.9 (**C9**), 115.0 (d, *J* 21.3, **C2**), 114.4 (**C7**), 79.2 (**C5**).

^{19}F NMR (471 MHz, $CDCl_3$) δ_C: −115.5 – −115.6 (m).

HRMS: (ESI^+) $C_{15}H_{13}FO$ $[M+Na]^+$ found 251.0836, requires 251.0843 (−2.65 ppm).

(±)-1-(4-Chlorophenyl)-1-phenylprop-2-en-1-ol (**(±)-3c**)

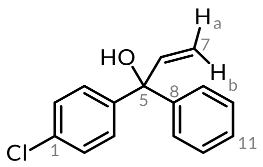

Following general procedure D, 4-chlorobenzophenone (325 mg, 1.50 mmol), vinylmagnesium bromide (1 M in THF, 3.8 mL, 3.8 mmol), and THF (2 mL) for 2 h gave, after purification by column chromatography (hexane : diethyl ether, 85 : 15), allylic alcohol **(±)-3c** as a colourless oil (313 mg, 1.28 mmol, 85%).

ν_{max} (film): 3446 (O–H), 3059 (C–H), 1489, 1447, 1398, 1092, 827, 763.

^{1}H NMR (500 MHz, $CDCl_3$) δ_H 7.39 – 7.26 (9H, m, **2-H, 3-H, 9-H, 10-H, 11-H**), 6.47 (1H, dd, *J* 17.1, 10.6, **6-H**), 5.33 (1H, dd, *J* 10.6, 1.0, **7a-H**), 5.29 (1H, dd, *J* 17.0, 1.1, **7b-H**), 2.27 (1H, s, **OH**).

^{13}C{^{1}H} NMR (176 MHz, $CDCl_3$) δ_C: 145.5 (**C4/C8**), 144.3 (**C4/C8**), 143.2 (**C6**), 133.2 (**C1**), 128.5 (**C2/C3/C9/C10**), 128.4 (**C2/C3/C9/C10**), 128.4 (**C2/C3/C9/C10**), 127.7 (**C11**), 126.9 (**C2/C3/C9/C10**), 114.6 (**C7**), 79.2 (**C5**).

HRMS: (ESI$^-$) $C_{15}H_{12}O^{35}Cl$ [M–H]$^-$ found 243.0573, requires 243.0582 (–3.8 ppm).

(±)-1-(3-Fluorophenyl)-1-phenylprop-2-en-1-ol (**(±)-3d**)

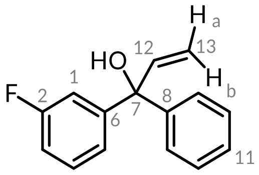

Following general procedure D, 3-fluorobenzophenone (400 mg, 2.00 mmol), vinylmagnesium bromide (1 M in THF, 5.0 mL, 5.0 mmol), and THF (2 mL) for 2 h gave, after purification by column chromatography (hexane : diethyl ether, 80 : 20), allylic alcohol **(±)-3d** as a colourless oil (420 mg, 1.84 mmol, 92%).

ν_{max} (film): 3451 (O–H), 3061 (C–H), 1612 (C–H), 1587, 1481, 1437, 1236, 993, 928, 876, 835, 784.

^{1}H NMR (500 MHz, $CDCl_3$) δ_H: 7.43 – 7.34 (4H, m, **9-H, 10-H**), 7.35 – 7.25 (2H, m, **4-H, 11-H**), 7.21 – 7.10 (2H, m, **1-H, 5-H**), 7.04 – 6.94 (1H, m, **3-H**), 6.51 (1H, dd, *J* 17.0, 10.6, **12-H**), 5.38 (1H, d, *J* 3.7, **13a-H**), 5.35 (1H, d, *J* 10.3, **13b-H**), 2.34 (1H, s, **OH**).

^{13}C{^{1}H} NMR (126 MHz, $CDCl_3$) δ_C: 162.8 (d, *J* 245.7, **C2**), 148.5 (d, *J* 6.6, **C6**), 145.4 (**C8**), 143.0 (**C12**), 129.7 (d, *J* 8.0, **C4**), 128.5 (**C10**), 127.7 (**C11**), 127.0 (**C9**), 122.7 (d, *J* 3.0, **C5**), 114.7 (**C13**), 114.3 (d, *J* 21.7, **C3**), 114.1 (d, *J* 22.6, **C1**), 79.2 (**C7**).

^{19}F NMR (471 MHz, $CDCl_3$) δ_F: –112.7 – –112.8 (m, **F**).

HRMS: (ESI$^+$) $C_{15}H_{13}FONa$ [M+Na]$^+$ found 251.0838, requires 251.0843 (–1.9 ppm).

(±)-1-Phenyl-1-(4-(trifluoromethyl)phenyl)prop-2-en-1-ol (**(±)-3e**)

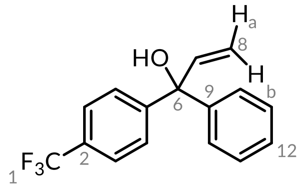

Following general procedure D, 4-trifluoromethylbenzophenone (375 mg, 1.50 mmol), vinylmagnesium bromide (1 M in THF, 3.0 mL, 3.0 mmol), and THF (3 mL) for 2 h gave, after purification by column chromatography (hexane : diethyl ether, 85 : 15), allylic alcohol **(±)-3e** as a colourless oil (140 mg, 0.50 mmol, 33%).

ν_{max} (film): 3428 (O–H), 3061 (C–H), 1616, 1410, 1325, 1165, 1126, 1069, 1018, 839, 764.

^{1}H NMR (500 MHz, CDCl$_3$) δ_H: 7.58 (2H, d, *J* 8.3, **3-H**), 7.52 (2H, d, *J* 8.3, **4-H**), 7.40 – 7.32 (4H, m, **10-H**, **11-H**), 7.32 – 7.28 (1H, m, **12-H**), 6.50 (1H, dd, *J* 17.1, 10.6, **7-H**), 5.37 (1H, d, *J* 10.6, **8a-H**), 5.34 (1H, d, *J* 17.2, **8b-H**), 2.32 (1H, s, **OH**).

^{13}C{^{1}H} NMR (126 MHz, CDCl$_3$) δ_C: 149.7 (**C5**), 145.3 (**C9**), 142.9 (**C7**), 129.5 (q, *J* 32.3, **C2**), 128.6 (**C10/C11**), 127.9 (**C12**), 127.3 (**C4**), 127.0 (**C10/C11**), 125.2 (q, *J* 3.8, **C3**), 124.3 (q, *J* 272.0, **C1**), 115.1 (**C8**), 79.3 (**C6**).

^{19}F NMR (376 MHz, CDCl$_3$) δ_F: –62.5 (**F**).

HRMS: (ESI$^-$) $C_{16}H_{12}F_3O$ [M–H]$^-$ found 277.0850, requires 277.0846 (+1.7 ppm).

(±)-1-(3-Fluoro-4-(trifluoromethyl)phenyl)-1-phenylprop-2-en-1-ol (**(±)-3f**)

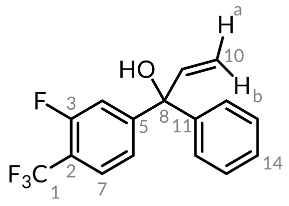

Following general procedure D, 4-trifluoromethyl-3-fluorobenzophenone (402 mg, 1.50 mmol), vinylmagnesium bromide (1 M in THF, 3.8 mL, 3.8 mmol), and THF (2 mL) for 2 h gave, after purification by column chromatography (hexane : diethyl ether, 80 : 20), allylic alcohol **(±)-3f** as a colourless oil (432 mg, 1.50 mmol, 97%).

ν_{max} (film): 3318 (O–H), 2967 (C–H), 2837 (C–H), 1672, 1599, 1510, 1246, 1169, 1031, 833, 781.

^{1}H NMR (500 MHz, $CDCl_3$) δ_H: 7.55 (1H, app. t, *J* 7.8, **7-H**), 7.37 (4H, d, *J* 4.3, **12-H, 13-H**), 7.35 – 7.28 (2H, m, **4-H**, **14-H**), 7.24 (1H, d, *J* 8.3, **6-H**), 6.47 (1H, dd, *J* 17.1, 10.6, **9-H**), 5.40 (1H, d, *J* 10.6, **10a-H**), 5.36 (1H, d, *J* 17.1, **10b-H**), 2.36 (1H, s, **OH**).

^{13}C{^{1}H} NMR (126 MHz, $CDCl_3$) δ_C: 159.7 (dq, *J* 256.1, 2.4, **C3**), 152.8 (d, *J* 7.0, **C5**), 144.8 (**C11**), 142.3 (**C9**), 128.7 (**C12**), 128.2 (**C14**), 127.0 (q, *J* 3.4, **C7**), 127.0 (**C13**), 122.8 (q, *J* 271.9, **C1**), 122.5 (d, *J* 3.6, **C6**), 117.0 (qd, *J* 33.3, 12.8, **C2**), 115.6 (**C10**), 115.5 (d, *J* 21.9, **C4**), 79.0 (**C8**).

^{19}F NMR (471 MHz, $CDCl_3$) δ_F: –61.2 (**CF_3**), –113.9 – –114.1 (m, **F**).

HRMS: (ESI$^+$) $C_{16}H_{12}OF_4Na$ [M+Na]$^+$ found 319.0718, requires 319.0717 (+0.3 ppm).

Synthesis of Ligands

6,6'-((1*E*,1'*E*)-(((1*R*,2*R*)-1,2-Di(naphthalen-1-yl)ethane-1,2-diyl)bis(azaneylylidene))bis(methaneylylidene))bis(2,4-di-*tert*-butylphenol) (**(*R*,*R*)-L-3**)

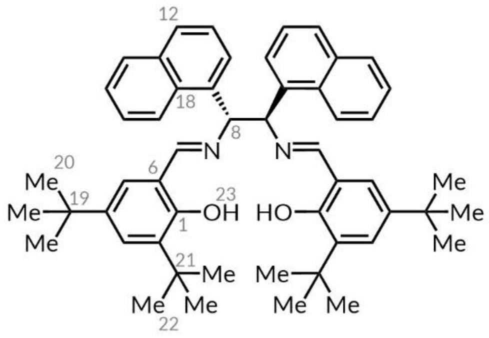

Following general procedure E, 3,5-di-*tert*-butyl-2-hydroxybenzaldehyde 239 (117 mg, 0.50 mmol), ((1*R*,2*R*)-1,2-di(naphthalen-1-yl)ethane-1,2-diamine (78 mg, 0.25 mmol) and EtOH (1.7 mL), gave salen **(*R*,*R*)-L-3** as a bright yellow powder (153 mg, 0.22 mmol, 91%).

mp 242 – 244 °C

$[\alpha]_D^{20}$ +103.6 (*c* 0.6 in $CHCl_3$).

ν_{max} **(film):** 2955, 2924, 2361, 1624, 1458, 1362, 1250, 1175, 912, 775.

^{1}H NMR (400 MHz, $CDCl_3$) δ_H: 13.80 (2H, s, **23-H**), 8.41 (2H, s, **7-H**), 8.17 (2H, br s, **H_{Ar}**), 7.74 – 7.67 (4H, m, **H_{Ar}**), 7.60 (2H, d, *J* 8.1, **H_{Ar}**), 7.46 – 7.28 (8H, m, **H_{Ar}**), 6.95 (2H, d, *J* 2.4, **5-H**), 5.86 (2H, s, **8-H**), 1.45 (18H, s, **22-H**), 1.22 (18H, s, **20-H**).

^{13}C{^{1}H} NMR (126 MHz, $CDCl_3$) δ_C: 167.7 (**C7**), 158.3 (**C1**), 140.1 (**C4**), 136.7 (**C2**), 136.2 (**C_{ArQ}**), 133.8 (**C_{ArQ}**), 130.9 (**C_{ArQ}**), 129.0 (**C_{ArH}**), 128.1 (**C_{ArH}**), 127.3 (**C3**), 127.0 (**C_{ArH}**), 126.5 (**C5**), 125.9 (**C_{ArH}**), 125.41 (**C_{ArH}**), 125.38 (**C_{ArH}**), 123.1 (br s, **C_{ArH}**), 118.0 (**C6**), 35.2 (**C21**), 34.2 (**C19**), 31.6 (**C20**), 29.6 (**C22**).

Note: Peak for C8 is not observed.

HRMS: (ESI$^+$) $C_{52}H_{61}O_2N_2$ $[M+H]^+$ found 745.4747, requires 745.4728 (+1.9 ppm).

6,6'-((1*E*,1'*E*)-(((1*R*,2*R*)-1,2-Bis(4-methoxyphenyl)ethane-1,2-diyl)bis(azaneylylidene))bis(methaneylylidene))bis(2,4-di-*tert*-butylphenol) (**(*R*,*R*)-L-4**)

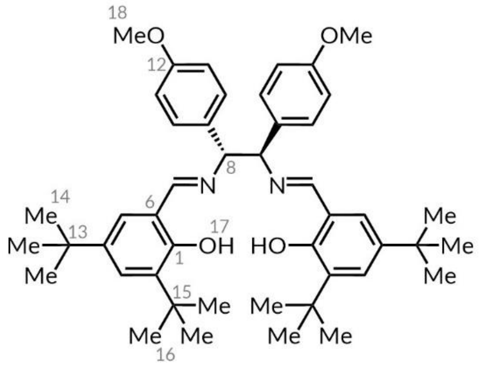

Following general procedure E, 3,5-di-*tert*-butyl-2-hydroxybenzaldehyde (231 mg, 0.98 mmol), (1*R*,2*R*)-1,2-bis(4-methoxyphenyl)ethane-1,2-diamine (134 mg, 0.49 mmol) and MeOH (2.5 mL), gave salen **(*R*,*R*)-L-4** as a bright yellow powder (241 mg, 0.34 mmol, 69%).

mp 179 – 181 °C

$[\alpha]_D^{20}$ –7.8 (*c* 0.5 in $CHCl_3$).

ν_{max} (film): 2955, 1624, 1512, 1248, 1175, 1038, 827.

^{1}H NMR (400 MHz, $CDCl_3$) δ_H: 13.63 (2H, s, **17-H**), 8.38 (2H, s, **7-H**), 7.31 (2H, d, *J* 2.4, **3-H**), 7.10 (4H, d, *J* 8.5, **10-H**), 6.98 (2H, d, *J* 2.4, **5-H**), 6.76 (4H, d, *J* 8.5, **11-H**), 4.66 (2H, s, **8-H**), 3.76 (6H, s, **18-H**), 1.43 (18H, s, **16-H**), 1.23 (18H, s, **14-H**).

^{13}C{^{1}H} NMR (126 MHz, $CDCl_3$) δ_C: 167.0 (**C7**), 158.9 (**C12**), 158.1 (**C1**), 140.1 (**C4**), 136.5 (**C2**), 132.3 (**C9**), 129.2 (**C10**), 127.2 (**C3**), 126.4 (**C5**), 118.0 (**C6**), 113.8 (**C11**), 79.6 (**C8**), 55.3 (**C18**), 35.1 (**C15**), 34.2 (**C13**), 31.6 (**C14**), 29.6 (**C16**).

HRMS: (ESI$^+$) $C_{46}H_{61}O_4N_2$ $[M+H]^+$ found 705.4616, requires 705.4626 (–1.4 ppm).

6,6'-((1*E*,1'*E*)-(((1*R*,2*R*)-1,2-Dimesitylethane-1,2-diyl)bis(azaneylylidene))bis(methaneylylidene))bis(2,4-di-*tert*-butylphenol) (**(*R*,*R*)-L-5**)

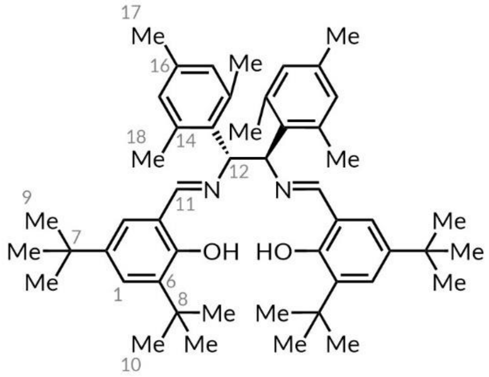

Following general procedure E, (1*R*,2*R*)-1,2-bis(2,4,6-trimethylphenyl)ethylenediamine dihydrochloride (100 mg, 0.27 mmol) (free-based), 3,5-di-*tert*-butyl-2-hydroxybenzaldehyde (127 mg, 0.54 mmol) and EtOH (4 mL) gave salen **(*R*,*R*)-L-5** as a bright yellow powder (159 mg, 0.25 mmol, 81%).

mp 196 – 198 °C

$[\alpha]_D^{20}$ +92.0 (*c* 0.5 in $CHCl_3$).

Chiral HPLC: Chiralpak OJ-H (hexane : IPA, 100 : 0, flow rate = 0.3 mL min^{-1}, λ = 254 nm, 30 °C) t_R(3*R*,4*R*): 11.4 min, t_R(3*S*,4*S*): 12.1 min, >99 : 1 er.

ν_{max} (film): 2949 (C–H), 2870, 1618 (C=N), 1436, 1249, 1172, 812.

^{1}H NMR (500 MHz, $CDCl_3$) δ_H: 13.54 (2H, s, **OH**), 8.42 (2H, s, **11-H**), 7.27 (2H, d, *J* 2.5, **1-H**), 7.03 (2H, d, *J* 2.4, **3-H**), 6.86 (2H, s, **15/15′-H**), 6.62 (2H, s, **15/15′-H**), 5.63 (2H, s, **12-H**), 2.71 (6H, s, **18/18′-H**), 2.21 (6H, s, **17-H**), 1.86 (6H, s, **18/18′-H**), 1.29 (18H, s, **10-H**), 1.26 (18H, s, **9-H**).

^{13}C{^{1}H} NMR (126 MHz, $CDCl_3$) δ_C: 166.4 (**C11**), 158.1 (**C5**), 139.7 (**C2**), 137.2 (**C14/C14′**), 137.1 (**C14/C14′**), 136.9 (**C16**), 136.4 (**C6**), 134.1 (**C13**), 131.2 (**C15/C15′**), 129.0 (**C15/C15′**), 126.9 (**C1**), 126.3 (**C3**), 118.2 (**C4**), 71.0 (**C12**), 35.0 (**C8**), 34.2 (**C7**), 31.6 (**C9**), 29.5 (**C10**), 22.4 (**C18/C18′**), 20.91 (**C17/C18/C18′**), 20.89 (**C17/C18/C18′**).

HRMS: (ESI^+) $C_{50}H_{69}N_2O_2$ $[M+H]^+$ found 729.5334, requires 729.5354 (–2.7 ppm).

6,6'-((1*E*,1'*E*)-((2-Methylpropane-1,2-diyl)bis(azaneylylidene))bis(methaneylylidene))bis (2,4-di-*tert*-butylphenol) (**L-6**)

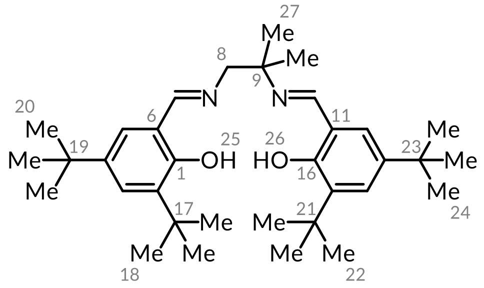

Following general procedure E, 3,5-di-*tert*-butyl-2-hydroxybenzaldehyde (2.00 g, 8.53 mmol), 2-methylpropane-1,2-diamine (0.48 mL, 4.27 mmol) and EtOH (30 mL), gave salen **L-6** as a yellow powder (1.87 g, 5.59 mmol, 84%) with spectroscopic data in accordance with the literature.[15]

mp 170 – 172 °C

^{1}H NMR (400 MHz, CDCl$_3$) δ_H: 14.22 (1H, s, **25-H/26-H**), 13.68 (1H, s, **25-H/26-H**), 8.39 (1H, s, **7-H/10-H**), 8.36 (1H, s, **7-H/10-H**), 7.37 (1H, d, *J* 2.7, **Ar-H**), 7.35 (1H, d, *J* 2.7, **Ar-H**), 7.09 (1H, d, *J* 2.5, **Ar-H**), 7.07 (1H, d, *J* 2.5, **Ar-H**), 3.71 (2H, s, **8-H**), 1.44 (9H, s, ^{t}**Bu**), 1.43 (9H, s, ^{t}**Bu**), 1.43 (6H, s, **27-H**), 1.30 (9H, s, ^{t}**Bu**), 1.29 (9H, s, ^{t}**Bu**).

Synthesis of Catalysts

Co(II)[6,6'-((1*E*,1'*E*)-(((1*R*,2*R*)-cyclohexane-1,2-diyl)bis(azaneylylidene))bis(methaneylylidene))bis(2,4-di-*tert*-butylphenol)] (**(*R*,*R*)-Co(II)-1**)

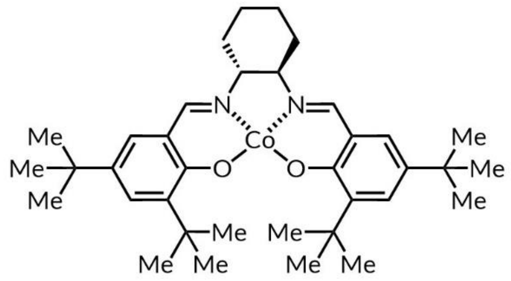

Catalyst **(*R*,*R*)-Co(II)-1** was purchased from Sigma−Aldrich.

Co(II)[6,6'-((1*E*,1'*E*)-(((1*R*,2*R*)-1,2-diphenylethane-1,2-diyl)bis(azaneylylidene))bis(methaneylylidene))bis(2,4-di-*tert*-butylphenol)] (**(*R*,*R*)-Co(II)-2**)

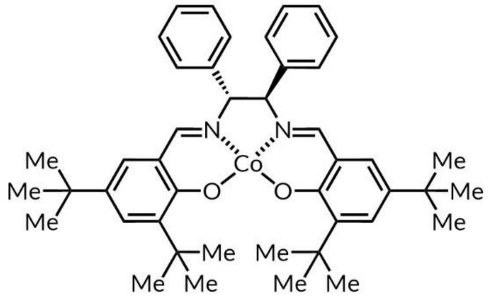

Following general procedure F, Co(OAc)$_2$ · 4H$_2$O (309 mg, 1.24 mmol), 6,6'-((1*E*,1'*E*)-(((1*R*,2*R*)-1,2-diphenylethane-1,2-diyl)bis(azaneylylidene))bis(methaneylylidene))bis(2,4-di-*tert*-butylphenol) **(*R*,*R*)-L-2** (800 mg, 1.24 mmol), and EtOH (6.2 mL), gave cobalt salen **(*R*,*R*)-Co(II)-2** as a red powder (703 mg, 1.00 mmol, 81%).

mp >300 °C

ν_{max} (film): 2949, 1587, 1526, 1248, 1179.

HRMS: (ESI$^+$) $C_{44}H_{54}CoN_2O_2$ [M]$^+$ found 701.3496, requires 701.3512 (–2.3 ppm).

Co(II)[6,6'-((1*E*,1'*E*)-(((1*R*,2*R*)-1,2-Di(naphthalen-1-yl)ethane-1,2-diyl)bis(azaneylylidene))bis(methaneylylidene))bis(2,4-di-*tert*-butylphenol)] ((***R*,*R*)-Co(II)-3**)

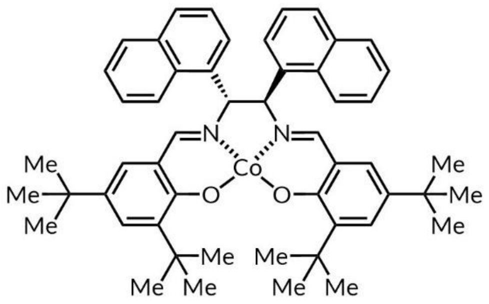

Following general procedure F, $Co(OAc)_2 \cdot 4H_2O$ (25 mg, 0.10 mmol), 6,6'-((1*E*,1'*E*)-(((1*R*,2*R*)-1,2-Bis(4-methoxyphenyl)ethane-1,2-diyl)bis(azaneylylidene))bis(methaneylylidene))bis(2,4-di-*tert*-butylphenol) ((***R*,*R*)-L-3**) (75 mg, 0.10 mmol), and EtOH (0.5 mL), gave cobalt salen complex **(*R*,*R*)-Co(II)-3** as a brown powder (69 mg, 0.09 mmol, 86%).

mp 235 °C (*dec*)

ν_{max} **(film):** 2935, 1580, 1522, 1252, 779.

HRMS: (ESI$^+$) $C_{52}H_{58}CoN_2O_2$ $[M]^+$ found 801.3817, requires 801.3825 (–1.0 ppm).

Co(II)[6,6'-((1*E*,1'*E*)-(((1*R*,2*R*)-1,2-Bis(4-methoxyphenyl)ethane-1,2-diyl)bis(azaneylylidene))bis(methaneylylidene))bis(2,4-di-*tert*-butylphenol)] (**(*R,R*)-Co(II)-4**)

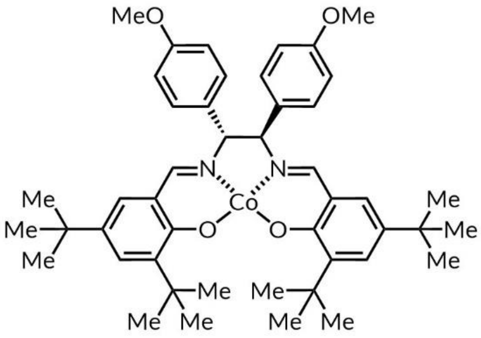

Following general procedure F, $Co(OAc)_2 \cdot 4H_2O$ (71 mg, 0.28 mmol), 6,6'-((1*E*,1'*E*)-(((1*R*,2*R*)-1,2-Bis(4-methoxyphenyl)ethane-1,2-diyl)bis(azaneylylidene))bis(methaneylylidene))bis(2,4-di-*tert*-butylphenol) (**(*R,R*)-L-4**) (200 mg, 0.28 mmol), and EtOH (2.8 mL), gave cobalt salen complex **(*R,R*)-Co(II)-4** as a red powder (100 mg, 0.13 mmol, 46%).

mp >300 °C

ν_{max} (film): 2949, 1510, 1250, 1173, 1023.

HRMS: (ESI$^+$) $C_{46}H_{58}CoN_2O_4$ $[M]^+$ found 761.3716, requires 761.3723 (–0.9 ppm).

Co(II)[6,6'-((1*E*,1'*E*)-(((1*R*,2*R*)-1,2-Dimesitylethane-1,2iyl)bis(azaneylylidene))bis(methaneylylidene))bis(2,4-di-*tert*-butylphenol)] (**(*R*,*R*)-Co(II)-5**)

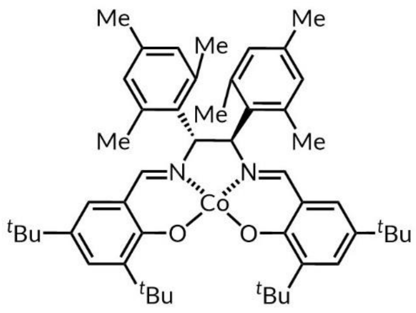

Following general procedure F, 6,6'-((1*E*,1'*E*)-(((1*R*,2*R*)-1,2-dimesitylethane-1,2-diyl)bis(azaneylylidene))bis(methaneylylidene))bis(2,4-di-*tert*-butylphenol) (**(*R*,*R*)-L-5**) (572 mg, 0.78 mmol), $Co(OAc)_2 \cdot 4H_2O$ (195 mg, 0.78 mmol) and EtOH (7.5 mL) for 6 hours gave cobalt salen complex **(*R*,*R*)-Co(II)-5** as a dark red powder (573 mg, 0.73 mmol, 93%).

mp 278 °C (*dec*)

ν_{max} (film): 2947, 2864, 1608, 1584 (C=N), 1522, 1458, 1356, 1250, 1177, 856, 841, 788.

HRMS: (ESI^+) $C_{50}H_{66}N_2O_2Co$ $[M]^+$ found 785.4439, requires 785.4451 (–1.5 ppm).

Co(II)[6,6'-((1*E*,1'*E*)-((2-Methylpropane-1,2-diyl)bis(azaneylylidene))bis(methaneylylidene))bis(2,4-di-*tert*-butylphenol)] (**Co(II)-6**)

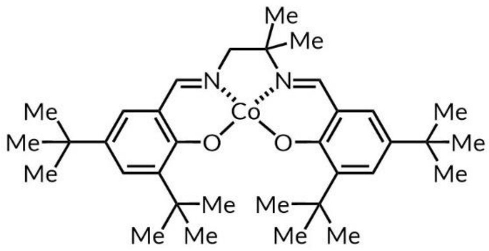

Following general procedure F, $Co(OAc)_2 \cdot 4H_2O$ (409 mg, 1.64 mmol), **L-6** (856 mg, 1.64 mmol), and EtOH (16 mL), gave cobalt salen **Co(II)-6** as a red powder (950 mg, 1.64 mmol, 94%).

mp >250 °C

ν_{max} (film): 2951, 1591 (C=N), 1525, 1254, 1177, 785.

HRMS: (ESI$^+$) $C_{34}H_{50}CoN_2O_2$ $[M]^+$ found 577.3190, requires 577.3199 (–1.5 ppm).

Co(II)[6,6'-((1*E*,1'*E*)-(((1*S*,2*S*)-1,2-di([1,1'-biphenyl]-2-yl)ethane-1,2-diyl)bis(azaneylylidene))bis(methaneylylidene))bis(2,4-di-tert-butylphenol)] (**(*S*,*S*)-Co(II)-7**)

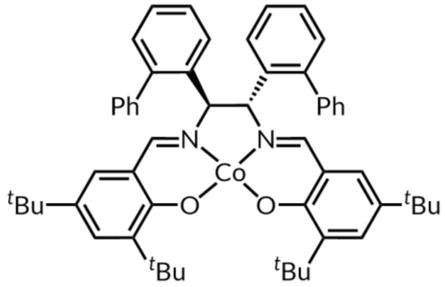

Catalyst **(*S*,*S*)-Co(II)-7** was prepared according to literature procedure by Zhang and co-workers.[16]

Cobalt(III) Salen Catalyst (**Co(III)-6**)

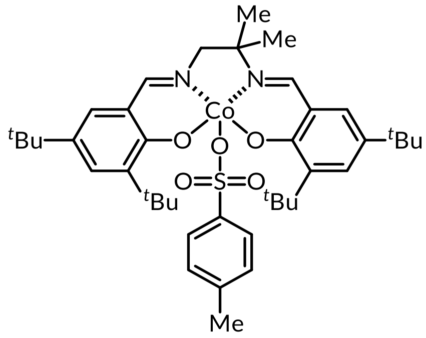

Following an adapted procedure by Jacobsen and co-workers[17], cobalt catalyst **Co(II)-6** (1.16 g, 2.0 mmol) and *p*-toluenesulfonic acid monohydrate (418 mg, 2.2 mmol) were stirred in dichloromethane (20 mL) at room temperature for 16 h. Dichloromethane was removed under reduced pressure, the product was dissolved in pentane and filtered to afford cobalt complex **Co(III)-6** as a green solid (1.26 g, 1.7 mmol, 84%).

mp 213 °C (*dec*)

ν_{max} **(film):** 2951, 1647, 1522 (C=N), 1458, 1361, 1233, 1165, 1034, 1009, 818.

HRMS: (ESI^+) $C_{34}H_{50}CoN_2O_2$ $[M–OTs]^+$ found 577.3198, requires 577.3199 (–0.2 ppm).

Synthesis of Products

(*R*)-1,2-Bis(4-fluorophenyl)propan-1-one ((***R***)**-2a**)

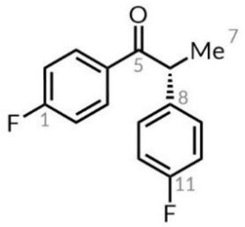

Following general procedure H, 1,1-bis(4-fluorophenyl)prop-2-en-1-ol (**1a**) (49 mg, 0.20 mmol), cobalt catalyst **(*R*,*R*)-Co(II)-5** (7.9 mg, 0.01 mmol), 1-fluoro-2,4,6-trimethylpyridinium tetrafluoroborate (91 mg, 0.40 mmol), 1,1,3,3-tetramethyldisiloxane (141 μL, 0.80 mmol), PhCl (1.7 mL) gave, after purification by column chromatography (hexane : CH_2Cl_2, 55 : 45), α-aryl ketone **(*R*)-2a** as a colourless oil (46 mg, 0.19 mmol, 93%). NMR spectroscopic data were in accordance with the literature.[18, 19]

$[\alpha]_D^{20}$ –156.5 (*c* 1.3 in $CHCl_3$).

Chiral HPLC: Chiralcel OJ-H (hexane : IPA, 99 : 1), flow rate = 1 mL min^{-1}, λ = 254 nm, 30 °C) t_R (*S*): 16.1 min, t_R (*R*): 18.1 min, 1 : 99 er.

ν_{max} (film): 3075, 2978, 1682 (C=O), 1597, 1506, 1220, 1155, 953.

^{1}H NMR (500 MHz, $CDCl_3$) δ_H: 8.01 – 7.89 (2H, m, **3-H**), 7.26 – 7.20 (2H, m, **9-H**), 7.10 – 7.03 (2H, m, **2-H**), 7.01 – 6.96 (2H, m, **10-H**), 4.63 (1H, q, *J* 6.8 Hz, **6-H**), 1.51 (3H, d, *J* 6.9 Hz, **7-H**).

^{13}C{^{1}H} NMR (126 MHz, $CDCl_3$) δ_C: 198.7 (**C5**), 165.7 (d, J_{CF} 255.0 Hz, **C1**), 162.0 (d, J_{CF} 245.7 Hz, **C11**), 137.1 (d, J_{CF} 3.3 Hz, **C8**), 132.7 (d, J_{CF} 3.1 Hz, **C4**), 131.5 (d, J_{CF} 9.3 Hz, **C3**), 129.4 (d, J_{CF} 8.0 Hz, **C9**), 116.1 (d, J_{CF} 21.4 Hz, **C10**), 115.8 (d, J_{CF} 21.9 Hz, **C2**), 47.2 (**C6**), 19.7 (**C7**).

^{19}F NMR (282 MHz, $CDCl_3$) δ_F: –105.2 (**F1**), –115.6 (**F11**).

HRMS: (ESI$^+$) $C_{15}H_{13}F_2O$ [M+H]$^+$ found 247.0923, requires 247.0929 (–2.4 ppm).

(*R*)-1,2-Diphenylpropan-1-one ((***R*)-2b**)

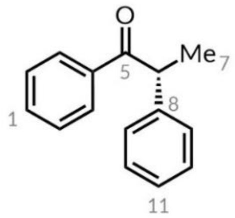

Following general procedure H, 1,1-diphenylprop-2-en-1-ol (**1b**) (210 mg, 1.00 mmol), cobalt catalyst **(*R*,*R*)-Co(II)-5** (39.3 mg, 0.05 mmol), 1-fluoro-2,4,6-trimethylpyridinium tetrafluoroborate (454 mg, 2.00 mmol), 1,1,3,3-tetramethyldisiloxane (707 μL, 4.00 mmol), PhCl (8.5 mL) gave, after purification by column chromatography (hexane : CH_2Cl_2, 60 : 40), α-aryl ketone **(*R*)-2b** as a colourless oil (183 mg, 0.87 mmol, 87%). Characterisation data were in accordance with the literature.[20]

$[\alpha]_D^{20}$ −152.5 (*c* 0.9 in $CHCl_3$).

Chiral HPLC: Chiralcel OD-H (hexane : IPA, 99 : 1), flow rate = 0.6 mL min^{-1}, λ = 254 nm, 30 °C) t_R (*S*): 11.4 min, t_R (*R*): 13.9 min, 2 : 98 er.

ν_{max} (film): 3061, 2928, 1680 (C=O), 1564, 1489, 1449, 1250, 1220, 950.

^{1}H NMR (500 MHz, $CDCl_3$) δ_H: 7.99 – 7.93 (2H, m, **3-H**), 7.51 – 7.45 (1H, m, **1-H**), 7.41 – 7.35 (2H, m, **2-H**), 7.31 – 7.27 (4H, m, **9-H**, **10-H**), 7.23 – 7.17 (1H, m, **11-H**), 4.69 (1H, q, *J* 6.9 Hz, **6-H**), 1.54 (3H, d, *J* 6.8 Hz, **7-H**).

^{13}C{^{1}H} NMR (126 MHz, $CDCl_3$) δ_C: 200.5 (**C5**), 141.6 (**C8**), 136.6 (**C4**), 132.9 (**C1**), 129.1 (**C10**), 128.9 (**C3**), 128.6 (**C2**), 127.9 (**C9**), 127.0 (**C11**), 48.0 (**C6**), 19.7 (**C7**).

HRMS: (ESI$^+$) $C_{15}H_{14}NaO$ [M+Na]$^+$ found 233.0933, requires 233.0937 (−1.7 ppm).

(R)-1,2-Bis(4-methylphenyl)propan-1-one (**(R)-2c**)

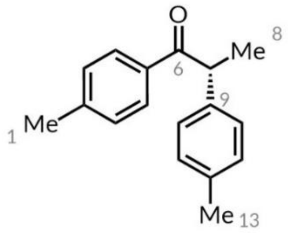

Following general procedure H, 1,1-bis(4-methylphenyl)prop-2-en-1-ol (**1c**) (48 mg, 0.20 mmol), cobalt catalyst **(R,R)-Co(II)-5** (7.9 mg, 0.01 mmol), 1-fluoro-2,4,6-trimethylpyridinium tetrafluoroborate (91 mg, 0.40 mmol), 1,1,3,3-tetramethyldisiloxane (141 μL, 0.80 mmol), PhCl (1.7 mL) gave, after purification by column chromatography (hexane : CH_2Cl_2, 50 : 50), α-aryl ketone **(R)-2c** as a colourless oil (39 mg, 0.16 mmol, 82%). Spectroscopic data were in accordance with the literature.[1]

$[\alpha]_D^{20}$ −116.2 (c 1.0 in $CHCl_3$).

Chiral HPLC: Chiralcel AS-H (hexane : IPA, 99.7 : 0.3), flow rate = 0.5 mL min^{-1}, λ = 254 nm, 30 °C) t_R (S): 14.1 min, t_R (R): 16.8 min, 1 : 99 er.

ν_{max} (film): 2974, 2928 (C–H), 1676 (C=O), 1604, 1224, 1175, 950, 815, 767.

^{1}H NMR (500 MHz, $CDCl_3$) δ_H: 7.89 – 7.81 (2H, m, **4-H**), 7.21 – 7.14 (4H, m, **3-H, 10-H**), 7.13 – 7.03 (2H, m, **11-H**), 4.63 (1H, q, J 6.8 Hz, **7-H**), 2.34 (3H, s, **1-H**), 2.28 (3H, s, **13-H**), 1.50 (3H, d, J 6.9 Hz, **8-H**).

^{13}C{^{1}H} NMR (126 MHz, $CDCl_3$) δ_C: 200.2 (**C6**), 143.6 (**C2**), 138.8 (**C9**), 136.6 (**C12**), 134.1 (**C5**), 129.8 (**C11**), 129.3 (**C3**), 129.0 (**C4**), 127.7 (**C10**), 47.4 (**C7**), 21.7 (**C1**), 21.2 (**C13**), 19.6 (**C8**).

HRMS: (ESI$^+$) $C_{17}H_{19}O$ [M+H]$^+$ found 239.1428, requires 239.1430 (−0.8 ppm).

(*R*)-1,2-Bis(4-(isopropyl)phenyl)propan-1-one ((***R***)**-2d**)

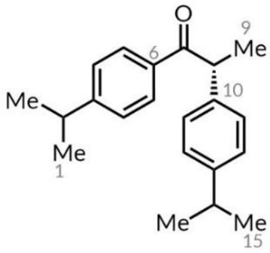

Following general procedure H, 1,1-bis(4-isopropylphenyl)prop-2-en-1-ol (**1d**) (59 mg, 0.20 mmol), cobalt catalyst (***R,R***)**-Co(II)-5** (7.9 mg, 0.01 mmol), 1-fluoro-2,4,6-trimethylpyridinium tetrafluoroborate (91 mg, 0.40 mmol), 1,1,3,3-tetramethyldisiloxane (141 µL, 0.80 mmol), PhCl (1.7 mL) gave, after purification by column chromatography (hexane : CH_2Cl_2, 70 : 30), α-aryl ketone (***R***)**-2d** as a colourless oil (53 mg, 0.18 mmol, 90%).

$[\alpha]_D^{20}$ –40.4 (*c* 0.5 in $CHCl_3$).

Chiral HPLC: Chiralpak IG (hexane : IPA, 99.5 : 0.5), flow rate = 1.0 mL min^{-1}, λ = 254 nm, 30 °C) t_R(*S*): 14.4 min, t_R(*R*): 15.6 min, 3 : 97 er.

ν_{max} (film): 2960, 2929, 1678 (C=O), 1604, 1224, 952.

^{1}H NMR (400 MHz, $CDCl_3$) δ_H: 7.92 (2H, d, *J* 8.3, **5-H**), 7.25 – 7.21 (4H, m, **4-H, 11-H**), 7.15 (2H, d, *J* 8.2, **12-H**), 4.66 (1H, q, *J* 6.8, **8-H**), 2.91(1H, sept, *J* 6.9, **2-H**), 2.85 (1H, sept, *J* 6.9, **14-H**), 1.51 (3H, d, *J* 6.8, **9-H**), 1.29 – 1.20 (12H, m, **1-H, 15-H**).

^{13}C{^{1}H} NMR (126 MHz, $CDCl_3$) δ_C: 200.3 (**C7**), 154.3 (**C3**), 147.4 (**C13**), 138.9 (**C10**), 134.5 (**C6**), 129.2 (**C5**), 127.7 (**C11**), 127.1 (**C12**), 126.7 (**C4**), 47.3 (**C8**), 34.3 (**C2**), 33.7 (**C14**), 24.0 (**C15**), 23.7 (**C1**), 19.7 (**C9**).

HRMS: (ESI$^+$) $C_{21}H_{26}NaO$ [M+Na]$^+$ found 317.1878, requires 317.1881 (–0.9 ppm).

(*R*)-1,2-bis(4-(*tert*-butyl)phenyl)propan-1-one (**(*R*)-2e**)

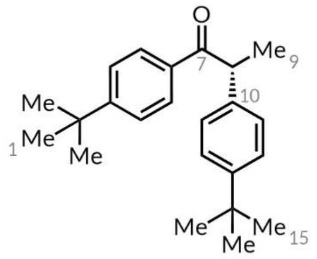

Following general procedure H, 1,1-bis(4-(*tert*-butyl)phenyl)prop-2-en-1-ol (**1e**) (64 mg, 0.20 mmol), cobalt catalyst **(*R*,*R*)-Co(II)-5** (7.9 mg, 0.01 mmol), 1-fluoro-2,4,6-trimethylpyridinium tetrafluoroborate (91 mg, 0.40 mmol), 1,1,3,3-tetramethyldisiloxane (141 μL, 0.80 mmol), PhCl (1.7 mL) gave, after purification by column chromatography (hexane : CH_2Cl_2, 70 : 30), α-aryl ketone **(*R*)-2e** as a white solid (50 mg, 0.16 mmol, 78%).

mp 85 – 87 °C

$[\alpha]_D^{20}$ –29 (*c* 0.5 in $CHCl_3$).

Chiral HPLC: Chiralpak IC (hexane : IPA, 99.5 : 0.5), flow rate = 1 mL min^{-1}, λ = 254 nm, 30 °C) t_R (*S*): 7.1 min, t_R (*R*): 8.6 min, 4 : 96 er.

ν_{max} (film): 2962 (C–H), 2904, 1678 (C=O), 1604, 1224, 952.

^{1}H NMR (400 MHz, $CDCl_3$) δ_H: 7.94 (2H, d, *J* 8.4, **5-H**), 7.42 (2H, d, *J* 8.4, **4-H**), 7.31 (2H, d, *J* 8.3, **12-H**) 7.24 (2H, d, *J* 8.3, **11-H**) 4.68 (1H, q, *J* 6.8, **8-H**), 1.52 (3H, d, *J* 6.8, **9-H**), 1.31 (9H, s, **1-H**), 1.28 (9H, s, **15-H**).

^{13}C{^{1}H} NMR (126 MHz, $CDCl_3$) δ_C: 200.2 (**C7**), 156.5 (**C3**), 149.7 (**C13**), 138.5 (**C10**), 134.1 (**C6**), 128.9 (**C5**), 127.5 (**C11**), 125.9 (**C12**), 125.5 (**C4**), 47.1 (**C8**), 35.1(**C2**), 34.5 (**C14**), 31.4 (**C15**), 31.1 (**C1**), 19.7 (**C9**).

HRMS: (ESI$^+$) $C_{23}H_{30}NaO$ [M+Na]$^+$ found 345.2195, requires 345.2194 (–0.2 ppm).

(*R*)-1,2-Di([1,1′-biphenyl]-4-yl)propan-1-one (**(*R*)-2f**)

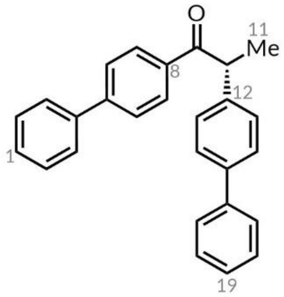

Following general procedure H, 1,1-di([1,1'-biphenyl]-4-yl)prop-2-en-1-ol (**1f**) (72 mg, 0.20 mmol), cobalt catalyst **(*R*,*R*)-Co(II)-5** (7.9 mg, 0.01 mmol), 1-fluoro-2,4,6-trimethylpyridinium tetrafluoroborate (91 mg, 0.40 mmol), 1,1,3,3-tetramethyldisiloxane (141 μL, 0.80 mmol), PhCl (1.7 mL) gave, after purification by column chromatography (hexane : CH_2Cl_2, 60 : 40), α-aryl ketone **(*R*)-2f** as a white solid (51 mg, 0.14 mmol, 70%).

mp 137 – 139 °C

$[\alpha]_D^{20}$ +57.4 (*c* 0.5 in $CHCl_3$).

Chiral HPLC: Chiralcel OD-H (hexane : IPA, 99 : 1), flow rate = 1 mL min^{-1}, λ = 254 nm, 30 °C) t_R(*S*): 18.9 min, t_R(*R*): 20.4 min, 97 : 3 er.

ν_{max} (film): 3030, 2974, 2929, 1674 (C=O), 1600, 1485, 1220, 1049, 893.

^{1}H NMR (400 MHz, $CDCl_3$) δ_H: 8.06 – 8.06 (2H, m, **7-H**), 7.63 (2H, d, *J* 8.0, **6-H**), 7.59 (2H, d, *J* 7.6, **3-H**), 7.56 – 7.55 (3H, m, **1-H, 14-H**), 7.46 – 7.37 (7H, m, **2-H, 13-H, 18-H, 19-H**), 7.32 (2H, t, *J* 7.4, **17-H**), 4.78 (1H, q, *J* 6.4, **10-H**), 1.60 (3H, d, *J* 6.8, **11-H**).

^{13}C{^{1}H} NMR (126 MHz, $CDCl_3$) δ_C: 199.9 (**C9**), 145.6 (**C5**), 140.7 (**C16**), 140.6 (**C12**), 139.9 (**C15, C4**), 135.2 (**C8**), 129.5 (**C7**), 129.0 (**C2**), 128.8 (**C18**), 128.3 (**C13**), 128.3 (**C14**), 127.8 (**C1**), 127.4 (**C6**), 127.4 (**C3**), 127.3 (**C17**), 127.2 (**C19**), 47.6 (**C10**), 19.6 (**C11**).

HRMS: (ESI$^+$) $C_{27}H_{22}ONa$ $[M+Na]^+$ found 385.1568, requires 385.1568 (0 ppm).

(*R*)-1,2-Bis(4-bromophenyl)propan-1-one (**(*R*)-2g**)

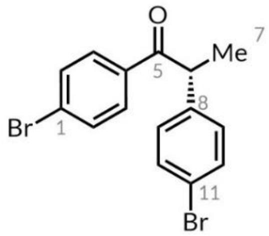

Following general procedure H, 1,1-bis(4-bromophenyl)prop-2-en-1-ol (**1g**) (74 mg, 0.20 mmol), cobalt catalyst **(*R*,*R*)-Co(II)-5** (7.9 mg, 0.01 mmol), 1-fluoro-2,4,6-trimethylpyridinium tetrafluoroborate (91 mg, 0.40 mmol), 1,1,3,3-tetramethyldisiloxane (141 μL, 0.80 mmol), PhCl (1.7 mL) gave, after purification by column chromatography (hexane : CH_2Cl_2 60 : 40), α-aryl ketone **(*R*)-2g** as a colourless oil (64 mg, 0.17 mmol, 86%).

$[\alpha]_D^{20}$ –35.7 (*c* 0.9 in $CHCl_3$).

Chiral HPLC: Chiralcel OJ-H (hexane : IPA, 99 : 1), flow rate = 0.5 mL min^{-1}, λ = 254 nm, 30 °C) t_R (*S*): 36.0 min, t_R (*R*): 38.4 min, 2 : 98 er.

ν_{max} (film): 2976, 2979, 1682 (C=O), 1583, 1487, 1394, 1217, 1173, 1072, 1005, 951, 826, 777.

^{1}H NMR (500 MHz, $CDCl_3$) δ_H: 7.80 – 7.74 (2H, m, **3-H**), 7.55 – 7.51 (2H, m, **2-H**), 7.44 – 7.40 (2H, m, **10-H**), 7.15 – 7.10 (2H, m, **9-H**), 4.58 (1H, q, *J* 6.8, **6-H**), 1.50 (3H, d, *J* 6.8, **7-H**).

^{13}C{^{1}H} NMR (126 MHz, $CDCl_3$) δ_C: 198.9 (**C5**), 140.2 (**C8**), 135.0 (**C4**), 132.4 (**C10**), 132.1 (**C2**), 130.4 (**C3**), 129.6 (**C9**), 128.4 (**C1**), 121.2 (**C11**), 47.5 (**C6**), 19.4 (**C7**).

HRMS: (ESI$^+$) $C_{15}H_{12}{}^{79}Br_2NaO$ $[M+Na]^+$ found 388.9151, requires 388.9147 (+1.0 ppm).

(R)-1,2-Bis(4-chlorophenyl)propan-1-one ((***R*)-2h**)

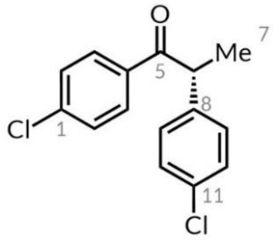

Following general procedure H, 1,1-bis(4-chlorophenyl)prop-2-en-1-ol (**1h**) (56 mg, 0.20 mmol), cobalt catalyst **(*R,R*)-Co(II)-5** (7.9 mg, 0.01 mmol), 1-fluoro-2,4,6-trimethylpyridinium tetrafluoroborate (91 mg, 0.40 mmol), 1,1,3,3-tetramethyldisiloxane (141 μL, 0.80 mmol), PhCl (1.7 mL) gave, after purification by column chromatography (hexane : CH_2Cl_2, 60 : 40), α-aryl ketone **(*R*)-2h** as a pale yellow oil that solidified at room temperature overnight (45 mg, 0.16 mmol, 81%).

mp 88 – 89 °C

$[\alpha]_D^{20}$ –78.4 (*c* 0.8 in $CHCl_3$).

Chiral HPLC: Chiralcel OJ-H (hexane : IPA, 99.5 : 0.5), flow rate = 0.5 mL min^{-1}, λ = 254 nm, 30 °C) t_R (*S*): 36.9 min, t_R (*R*): 40.3 min, 2 : 98 er.

ν_{max} (film): 2976 (C–H), 1683 (C=O), 1589, 1490, 1398, 1217, 1093, 1004, 950, 831, 781.

^{1}H NMR (500 MHz, $CDCl_3$) δ_H: 7.90 – 7.82 (2H, m, **3-H**), 7.39 – 7.34 (2H, m, **2-H**), 7.27 (2H, m, **10-H**), 7.23 – 7.16 (2H, m, **9-H**), 4.60 (1H, q, *J* 6.8, **6-H**), 1.51 (3H, d, *J* 6.8, **7-H**).

^{13}C{^{1}H} NMR (126 MHz, $CDCl_3$) δ_C: 198.8 (**C5**), 139.7 (**C8**), 139.6 (**C4**), 134.6 (**C1**), 133.1 (**C11**), 130.3 (**C3**), 129.4 (**C10**), 129.2 (**C9**), 129.1 (**C2**), 47.4 (**C6**), 19.5 (**C7**).

HRMS: (ESI^+) $C_{15}H_{13}{}^{35}Cl_2O$ $[M+H]^+$ found 279.0339, requires 279.0338 (–0.4 ppm).

(*R*)-1,2-Bis(4-methoxyphenyl)propan-1-one (**(*R*)-2i**)

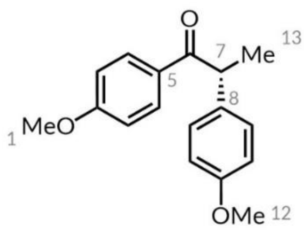

Following general procedure H, 1,1-bis(4-methoxyphenyl)prop-2-en-1-ol (**1i**) (54 mg, 0.20 mmol), cobalt catalyst **(*R*,*R*)-Co(II)-5** (7.9 mg, 0.01 mmol), 1-fluoro-2,4,6-trimethylpyridinium tetrafluoroborate (91 mg, 0.40 mmol), 1,1,3,3-tetramethyldisiloxane (141 μL, 0.80 mmol), PhCl (1.7 mL) gave, after purification by column chromatography (hexane : diethyl ether, 95 : 5 – 65 : 35), α-aryl ketone **(*R*)-2i** as a yellow oil (17 mg, 0.06 mmol, 31%). NMR spectroscopic data were in accordance with the literature.[21]

$[\alpha]_D^{20}$ –61.0 (*c* 0.6 in $CHCl_3$).

Chiral HPLC: Chiralcel OD-H (hexane : IPA, 99 : 1), flow rate = 0.6 mL min^{-1}, λ = 254 nm, 30 °C) t_R (*S*): 32.2 min, t_R (*R*): 34.4 min, 3 : 97 er.

ν_{max} (film): 2967, 2931, 2837, 1672 (C=O), 1599, 1510, 1246, 1169, 1031, 833, 781.

^{1}H NMR (400 MHz, $CDCl_3$) δ_H: 7.98 – 7.91 (2H, m, **4-H**), 7.23 – 7.14 (2H, m, **9-H**), 6.89 – 6.83 (2H, m, **3-H**), 6.84 – 6.79 (2H, m, **10-H**), 4.60 (1H, q, *J* 6.8, **7-H**), 3.82 (3H, s, **1-H**), 3.75 (3H, s, **12-H**), 1.49 (3H, d, *J* 6.9, **13-H**).

^{13}C{^{1}H} NMR (126 MHz, $CDCl_3$) δ_C: 199.2 (**C6**), 163.3 (**C2**), 158.5 (**C11**), 134.1 (**C8**), 131.2 (**C4**), 129.6 (**C5**), 128.8 (**C9**), 114.5 (**C10**), 113.8 (**C3**), 55.5 (**C1**), 55.3 (**C12**), 46.7 (**C7**), 19.7 (**C13**).

HRMS: (ESI$^+$) $C_{17}H_{19}O_3$ [M+H]$^+$ found 271.1328, requires 247.0929 (–0.2 ppm).

(*R*)-1,2-Bis(4-phenoxyphenyl)propan-1-one (**(*R*)-2j**)

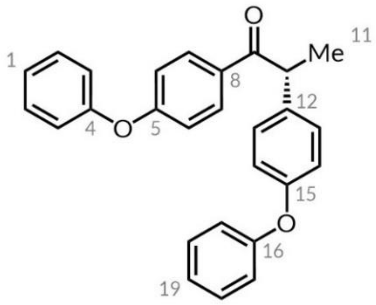

Following general procedure H, 1,1-bis(4-phenoxyphenyl)prop-2-en-1-ol (**1j**) (79 mg, 0.20 mmol), cobalt catalyst **(*R*,*R*)-Co(II)-5** (7.9 mg, 0.01 mmol), 1-fluoro-2,4,6-trimethylpyridinium tetrafluoroborate (91 mg, 0.40 mmol), 1,1,3,3-tetramethyldisiloxane (141 μL, 0.80 mmol), PhCl (1.7 mL) gave, after purification by column chromatography (hexane : CH_2Cl_2, 80 : 20), α-aryl ketone **(*R*)-2j** as a white solid (59 mg, 0.15 mmol, 75%).

mp 132 – 134 °C

$[\alpha]_D^{20}$ −15.5 (*c* 0.8 in $CHCl_3$).

Chiral HPLC: Chiralcel OD-H (hexane : IPA, 98.5 : 1.5), flow rate = 1 mL min^{-1}, λ = 254 nm, 30 °C) t_R (*S*): 23.1 min, t_R (*R*): 27.9 min, < 1 : 99 er.

ν_{max} (film): 3055, 2972, 1666 (C=O), 1585, 1487, 1236 , 1167, 870.

^{1}H NMR (500 MHz, $CDCl_3$) δ_H: 7.99 – 7.93 (2H, m, **7-H**), 7.42 – 7.36 (2H, m, **2-H/18-H**), 7.35 – 7.29 (2H, m, **2-H/18-H**), 7.25 – 7.21 (2H, m, **13-H**), 7.21 – 7.17 (1H, m, **1-H/19-H**), 7.10 (1H, tt, *J* 7.4, 1.1, **1-H/19-H**), 7.07 – 7.03 (2H, m, **3-H/17-H**), 7.00 – 6.97 (2H, m, **3-H/17-H**), 6.96 – 6.91 (4H, m, **14-H**, **6-H**), 4.63 (1H, q, *J* 6.9, **10-H**), 1.52 (3H, d, *J* 6.8, **11-H**).

^{13}C{^{1}H} NMR (126 MHz, $CDCl_3$) δ_C: 198.9 (**C9**), 161.8 (**C5**), 157.0 (**C4/C16**), 156.2 (**C15**), 155.4 (**C4/C16**), 136.3 (**C12**), 131.1 (**C7**), 130.9 (**C8**), 130.1 (**C2/C18**), 129.8 (**C2/C18**), 129.0 (**C13**), 124.7 (**C1/C19**), 123.4 (**C1/C19**), 120.3 (**C3/C17**), 119.1 (**C14**), 119.0 (**C3/C17**), 117.2 (**C6**), 46.8 (**C10**), 19.6 (**C11**).

HRMS: (ESI$^+$) $C_{27}H_{22}O_3Na$ $[M+Na]^+$ found 417.1456, requires 417.1461 (−1.2 ppm).

(*R*)-1,2-Bis(4-(methylthio)phenyl)propan-1-one (**(*R*)-2k**)

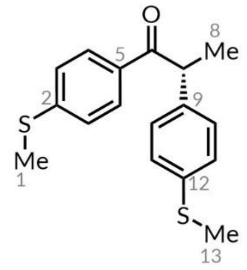

Following general procedure H, 1,1-bis(4-(methylthio)phenyl)prop-2-en-1-ol (**1k**) (60 mg, 0.20 mmol), cobalt catalyst **(*R*,*R*)-Co(II)-5** (7.9 mg, 0.01 mmol), 1-fluoro-2,4,6-trimethylpyridinium tetrafluoroborate (91 mg, 0.40 mmol), 1,1,3,3-tetramethyldisiloxane (141 μL, 0.80 mmol), PhCl (1.7 mL) gave, after purification by column chromatography (hexane : CH_2Cl_2, 80 : 20), α-aryl ketone **(*R*)-2k** as a white solid (53 mg, 0.17 mmol, 88%).

mp 128 – 130 °C

$[\alpha]_D^{20}$ +37.2 (*c* 0.5 in $CHCl_3$).

Chiral HPLC: Chiralcel OD-H (hexane : IPA, 99 : 1), flow rate = 1 mL min^{-1}, λ = 254 nm, 30 °C) t_R (*S*): 19.1 min, t_R (*R*): 20.5 min, 2 : 98 er.

ν_{max} (film): 2920 (C–H), 1670 (C=O), 1585, 1093, 850.

^{1}H NMR (400 MHz, $CDCl_3$) δ_H: 7.86 – 7.84 (2H, m, **4-H**), 7.19 – 7.17 (6H, m, **3-H**, **10-H**, **11-H**), 4.59 (1H, q, *J* 6.8, **7-H**), 2.47 (3H, s, **1-H**), 2.43 (3H, s, **13-H**), 1.50 (3H, d, *J* 6.8, **8-H**).

^{13}C{^{1}H} NMR (126 MHz, $CDCl_3$) δ_C: 199.2 (**C6**), 145.7 (**C2**), 138.6 (**C9**), 137.0 (**C12**), 132.7 (**C5**), 129.3 (**C4**), 128.3 (**C10**), 127.3 (**C3**), 125.0 (**C11**), 47.2 (**C7**), 19.5 (**C8**), 15.9 (**C13**), 14.8 (**C1**).

HRMS: (ESI$^+$) $C_{17}H_{18}NaOS_2$ $[M+Na]^+$ found 325.0698, requires 325.0697 (–0.3 ppm).

(*R*)-1,2-Bis(4-((trimethylsilyl)ethynyl)phenyl)propan-1-one (**(*R*)-2l**)

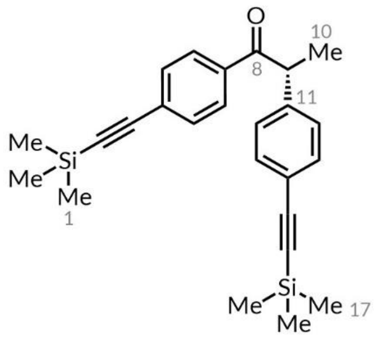

Following general procedure H, 1,1-bis(4-((trimethylsilyl)ethynyl)phenyl)prop-2-en-1-ol (**1l**) (80 mg, 0.20 mmol), cobalt catalyst **(*R*,*R*)-Co(II)-5** (7.9 mg, 0.01 mmol), 1-fluoro-2,4,6-trimethylpyridinium tetrafluoroborate (91 mg, 0.40 mmol), 1,1,3,3-tetramethyldisiloxane (141 μL, 0.80 mmol), PhCl (1.7 mL) gave, after purification by column chromatography (hexane : CH_2Cl_2, 70 : 30), α-aryl ketone **(*R*)-2l** as a white solid (63 mg, 0.16 mmol, 78%).

mp 129 – 131 °C

$[\alpha]_D^{20}$ +40.0 (*c* 0.5 in $CHCl_3$).

Chiral HPLC: Chiralpak IA (hexane : IPA, 99.5 : 0.5), flow rate = 1 mL min^{-1}, λ = 254 nm, 30 °C) t_R (*S*): 5.1 min, t_R (*R*): 6.0 min, 5 : 95 er.

ν_{max} (film): 2958 (C–H), 2158, 1683 (C=O), 1598, 1249, 862.

^{1}H NMR (400 MHz, $CDCl_3$) δ_H: 7.82 (2H, d, *J* 8.3, **6-H**), 7.43 (2H, d, *J* 8.3, **5-H**), 7.38 (2H, d, *J* 8.2, **13-H**), 7.17 (2H, d, *J* 8.2, **12-H**), 4.60 (1H, q, *J* 6.8, **9-H**), 1.50 (3H, d, *J* 6.8, **10-H**), 0.24 (9H, s, **1-H/17-H**), 0.22 (9H, s, **1-H/17-H**).

^{13}C{^{1}H} NMR (126 MHz, $CDCl_3$) δ_C: 199.3 (**C8**), 141.8 (**C11**), 135.7 (**C7**), 132.7 (**C13**), 132.1 (**C5**), 128.6 (**C6**), 127.8 (**C4**), 127.7 (**C12**), 122.0 (**C14**), 104.7 (**C15**), 104.1 (**C3**), 98.2 (**C2/16C**), 94.6 (**C2/16C**), 48.1 (**C9**), 19.3 (**C10**), 0.08 (**C1/C17**), –0.04 (**C1/C17**).

HRMS: (ESI$^+$) $C_{25}H_{30}NaOSi_2$ [M+Na]$^+$ found 425.1732, requires 425.1733 (–0.2 ppm).

(*R*)-1,2-Bis(4-(trifluoromethyl)phenyl)propan-1-one (**(*R*)-2m**)

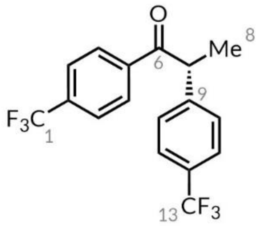

Following general procedure H, 1,1-bis(4-(trifluoromethyl)phenyl)prop-2-en-1-ol (**1m**) (69 mg, 0.20 mmol), cobalt catalyst **(*R*,*R*)-Co(II)-5** (7.9 mg, 0.01 mmol), 1-fluoro-2,4,6-trimethylpyridinium tetrafluoroborate (91 mg, 0.40 mmol), 1,1,3,3-tetramethyldisiloxane (141 μL, 0.80 mmol), PhCl (1.7 mL) gave, after purification by column chromatography (hexane : diethyl ether, 95 : 5 to 75 : 25), α-aryl ketone **(*R*)-2m** as a yellow oil (57 mg, 0.16 mmol, 82%).

$[\alpha]_D^{20}$ –87.2 (*c* 0.3 in $CHCl_3$).

Chiral HPLC: Chiralcel AS-H (hexane : IPA, 99.7 : 0.3), flow rate = 0.5 mL min^{-1}, λ = 254 nm, 30 °C) t_R (*S*): 12.2 min, t_R (*R*): 13.9 min, 30 : 70 er.

ν_{max} (film): 2938, 1692 (C=O), 1410, 1323, 1167, 1126, 1113, 1069, 845.

^{1}H NMR (400 MHz, $CDCl_3$) δ_H: 8.04 (2H, d, *J* 8.1, **4-H**), 7.69 (2H, d, *J* 8.2, **3-H**), 7.60 (2H, d, *J* 8.0, **11-H**), 7.41 (2H, d, *J* 8.0, **10-H**), 4.76 (1H, q, *J* 6.8, **7-H**), 1.59 (3H, d, *J* 6.9, **8-H**).

^{13}C{^{1}H} NMR (126 MHz, $CDCl_3$) δ_C: 198.8 (**C6**), 144.8 (**C9**), 138.9 (**C5**), 134.6 (q, *J* 32.7, **C2**), 129.7 (q, *J* 32.6, **C12**), 129.2 (**C4**), 128.3 (**C10**), 126.3 (q, *J* 3.7, **C11**), 125.9 (q, *J* 3.7, **C3**), 124.1 (q, *J* 272.0, **C1**), 123.6 (q, *J* 272.7, **C13**), 48.2 (**C7**), 19.5 (**C8**).

^{19}F NMR (471 MHz, $CDCl_3$) δ_F: –62.6 (**13-CF_3**), –63.2 (**1-CF_3**).

HRMS: (ESI^+) $C_{17}H_{12}F_6NaO$ $[M+Na]^+$ found 369.0684, requires 369.0685 (–0.3 ppm).

(*R*)-1,2-Bis(3-trifluoromethylphenyl)propan-1-one ((***R*)-2n**)

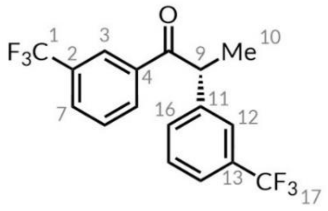

Following general procedure H, 1,1-bis(3-trifluoromethylphenyl)prop-2-en-1-ol (**1n**) (35 mg, 0.10 mmol), cobalt catalyst **(*R*,*R*)-Co(II)-5** (3.9 mg, 0.005 mmol), 1-fluoro-2,4,6-trimethylpyridinium tetrafluoroborate (45 mg, 0.20 mmol), 1,1,3,3-tetramethyldisiloxane (71 μL, 0.40 mmol), PhCl (0.9 mL) gave, after purification by preparative thin layer chromatography (hexane : diethyl ether, 80 : 20), α-aryl ketone **(*R*)-2n** as a colourless oil (7.4 mg, 0.02 mmol, 21%).

$[\alpha]_D^{20}$ –41.7 (*c* 0.4 in $CHCl_3$).

Chiral HPLC: Chiralcel OD-H (hexane : IPA, 99 : 1), flow rate = 0.6 mL min^{-1}, λ = 254 nm, 30 °C) t_R (*S*): 9.1 min, t_R (*R*): 10.5 min, 26 : 74 er.

ν_{max} (film): 2933, 1692 (C=O), 1329, 1166, 1126, 1074, 804.

^{1}H NMR (500 MHz, $CDCl_3$) δ_H: 8.21 (1H, s, **3-H**), 8.09 (1H, d, *J* 7.9, **5-H**), 7.76 (1H, d, *J* 7.8, **7-H**), 7.59 – 7.53 (2H, m, **6-H, 12-H**), 7.52 – 7.40 (3H, m, **14-H**, **15-H**, **16-H**), 4.75 (1H, q, *J* 6.9, **9-H**), 1.59 (3H, d, *J* 6.8, **10-H**).

^{13}C{^{1}H} NMR (126 MHz, $CDCl_3$) δ_C: 198.2 (**C8**), 141.6 (**C11**), 136.6 (**C4**), 131.8 (**C5**), 131.6 (q, *J* 17.3, **C13**), 131.3 (q, *J* 18.1, **C2**), 131.1 (**C16**), 129.63 (**C15**), 129.56 (q, *J* 3.7, **C7**), 129.4 (**C6**), 125.6 (q, *J* 3.5, **C3**), 124.6 (q, *J* 4.0, **C12**), 124.2 (q, *J* 3.9, **C14**), 123.88 (q, *J* 272.5, **C17**), 123.57 (q, *J* 272.0, **C1**), 47.8 (**C9**), 19.4 (**C10**).

^{19}F NMR (471 MHz, $CDCl_3$) δ_F: –62.7 (**1-CF_3**), –63.0 (**17-CF_3**).

HRMS: (ESI^+) $C_{17}H_{12}F_6ONa$ $[M+Na]^+$ found 369.0684, requires 369.0685 (–0.3 ppm).

(*R*)-1,2-Bis(3-chlorophenyl)propan-1-one (**(*R*)-2o**)

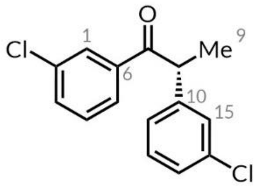

Following general procedure H, 1,1-bis(3-chlorophenyl)prop-2-en-1-ol (**1o**) (56 mg, 0.20 mmol), cobalt catalyst **(*R*,*R*)-Co(II)-5** (7.9 mg, 0.01 mmol), 1-fluoro-2,4,6-trimethylpyridinium tetrafluoroborate (91 mg, 0.40 mmol), 1,1,3,3-tetramethyldisiloxane (141 μL, 0.80 mmol), PhCl (1.7 mL) gave, after purification by column chromatography (hexane : diethyl ether, 95 : 5 to 75 : 25), α-aryl ketone **(*R*)-2o** as a yellow oil (44 mg, 0.16 mmol, 80%).

$[\alpha]_D^{20}$ –57.9 (*c* 0.2 in $CHCl_3$).

Chiral HPLC: Chiralcel OD-H (hexane : IPA, 99 : 1), flow rate = 0.6 mL min^{-1}, λ = 254 nm, 30 °C) t_R (*S*): 11.0 min, t_R (*R*): 15.9 min, 8 : 92 er.

ν_{max} (film): 3065, 2959, 2930, 1688 (C=O), 1572, 1472, 11420, 1215, 1084, 1009, 787.

^{1}H NMR (400 MHz, $CDCl_3$) δ_H: 7.91 (1H, t, *J* 1.9, **1-H**), 7.78 (1H, ddd, *J* 7.8, 1.7, 1.1, **5-H**), 7.47 (1H, ddd, *J* 8.0, 2.1, 1.1, **3-H**), 7.34 (1H, t, *J* 7.9, **4-H**), 7.28 – 7.18 (3H, m, **12-H**, **13-H**, **15-H**), 7.14 (1H, dt, *J* 7.3, 1.7, **11-H**), 4.60 (1H, q, *J* 6.8, **8-H**), 1.52 (3H, d, *J* 6.9, **9-H**).

^{13}C{^{1}H} NMR (126 MHz, $CDCl_3$) δ_C: 198.6 (**C7**), 142.9 (**C10**), 137.9 (**C6**), 135.2 (**C2**), 135.0 (**C14**), 133.1 (**C3**), 130.5 (**C12**), 130.1 (**C4**), 128.9 (**C1**), 128.0 (**C15**), 127.6 (**C13**), 126.9 (**C5**), 126.1 (**C11**), 47.8 (**C8**), 19.5 (**C9**).

HRMS: (ESI$^+$) $C_{15}H_{12}{}^{35}Cl_2NaO$ $[M+Na]^+$ found 301.0156, requires 301.0157 (–0.3 ppm).

(*R*)-1,2-Bis(3-fluorophenyl)propan-1-one (**(*R*)-2p**)

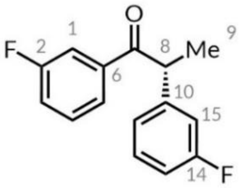

Following general procedure H, 1,1-bis(3-fluorophenyl)prop-2-en-1-ol (**1p**) (44 mg, 0.20 mmol), cobalt catalyst **(*R*,*R*)-Co(II)-5** (7.9 mg, 0.01 mmol), 1-fluoro-2,4,6-trimethylpyridinium tetrafluoroborate (91 mg, 0.40 mmol), 1,1,3,3-tetramethyldisiloxane (141 μL, 0.80 mmol), PhCl (1.7 mL) gave, after purification by column chromatography (hexane : CH_2Cl_2, 60 : 40), α-aryl ketone **(*R*)-2p** as a pale yellow oil (44 mg, 0.16 mmol, 81%).

$[\alpha]_D^{20}$ −133.4 (*c* 1.3 in $CHCl_3$).

Chiral HPLC: Chiralcel OD-H (hexane : IPA, 99 : 1), flow rate = 0.3 mL min^{-1}, λ = 254 nm, 30 °C) t_R (*S*): 21.3 min, t_R (*R*): 27.7 min, 5 : 95 er.

ν_{max} (film): 3074, 2932, 1684 (C=O), 1587, 1483, 1439, 1250, 1140, 910, 871, 787.

^{1}H NMR (500 MHz, $CDCl_3$) δ_H: 7.70 (1H, ddd, *J* 7.8, 1.6, 1.0, **1-H**), 7.61 (1H, ddd, *J* 9.6, 2.7, 1.6, **5-H**), 7.37 (1H, td, *J* 8.0, 5.5, **4-H**), 7.27 (1H, ddd, *J* 13.9, 7.9, 13.9, **12-H**), 7.20 (1H, tdd, *J* 8.2, 2.7, 1.0, **3-H**), 7.05 (1H, dt, *J* 7.8, 1.4, **11-H**), 6.98 (1H, ddd, *J* 9.8, 2.6, 1.7, **15-H**), 6.91 (1H, tdd, *J* 8.4, 2.6, 1.0, **13-H**), 4.62 (1H, q, *J* 6.9, **8-H**), 1.53 (3H, d, *J* 6.9, **9-H**).

^{13}C{^{1}H} NMR (126 MHz, $CDCl_3$) δ_C: 198.6 (d, J_{CF} 2.3, **C7**), 163.2 (d, J_{CF} 246.9, **C14**), 162.9 (d, J_{CF} 247.9, **C2**), 143.5 (d, J_{CF} 7.2, **C10**), 138.5 (d, J_{CF} 6.0, **C6**), 130.7 (d, J_{CF} 8.2, **C12**), 130.4 (d, J_{CF} 7.7, **C4**), 124.6 (d, J_{CF} 3.0, **C1**), 123.6 (d, J_{CF} 2.8, **C11**), 120.2 (d, J_{CF} 21.5 (**C3**), 115.6 (d, J_{CF} 22.3, **C5**), 114.8 (d, J_{CF} 21.7, **C15**), 114.3 (d, J_{CF} 21.1, **C13**), 47.9 (**C8**), 19.5 (**C9**).

^{19}F NMR (471 MHz, $CDCl_3$) δ_F: −111.60 – −111.72 (m, **F2**), −111.99 – −112.09 (m, **F14**).

HRMS: (ESI$^+$) $C_{15}H_{12}F_2ONa$ $[M+Na]^+$ found 269.0747, requires 269.0748 (−0.4 ppm).

(R)-1,2-*m*-tolylpropan-1-one (**(R)-2q**)

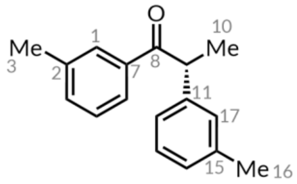

Following general procedure E, 1,1-di-*m*-tolylprop-2-en-1-ol (48 mg, 0.20 mmol), cobalt catalyst **(R,R)-Co(II)-5** (7.9 mg, 0.01 mmol), 1-fluoro-2,4,6-trimethylpyridinium tetrafluoroborate (91 mg, 0.40 mmol), 1,1,3,3-tetramethyldisiloxane (141 μL, 0.80 mmol), PhCl (1.7 mL) gave, after purification by column chromatography (hexane : CH_2Cl_2, 70 : 30), α-aryl ketone **(R)-2q** as a colourless oil (40 mg, 0.17 mmol, 85%).

$[\alpha]_D^{20}$ −155.6 (c 0.45 in $CHCl_3$).

Chiral HPLC: Chiralcel AD-H (hexane : IPA, 99 : 1), flow rate = 1 mL min^{-1}, λ = 220 nm, 30 °C) t_R (S) : 6.8 min, t_R (R): 7.6 min, 2 : 98 er.

ν_{max} (film): 2974 (C–H), 1680 (C=O), 1602, 1452, 1255, 702.

^{1}H NMR (400 MHz, $CDCl_3$) δ_H: 7.80 (1H, s, **1-H**), 7.76 – 7.75 (1H, m, **6-H**), 7.30 – 7.29 (1H, m, **4-H**), 7.28 – 7.25 (1H, m, **5-H**), 7.21 – 7.17 (1H, m, **13-H**), 7.11 – 7.10 (2H, m, **12-H**, **17-H**), 7.02 (1H, d, J 7.5, **14-H**), 4.66 (1H, q, J 6.8, **9-H**), 2.36 (3H, s, **3-H**), 2.31 (3H, s, **16-H**), 1.53 (3H, d, J 6.8, **10-H**).

^{13}C{^{1}H} NMR (126 MHz, $CDCl_3$) δ_C: 200.7 (**C8**), 141.5 (**C11**), 138.7 (**C15**), 138.3 (**C2**), 136.7 (**C7**), 133.6 (**C4**), 129.3 (**C1**), 128.9 (**C13**), 128.44 (**C5**), 128.43 (**C17**), 127.7 (**C14**), 126.1 (**C6**), 125.0 (**C12**), 47.8 (**C9**), 21.5 (**C3**), 21.4 (**C16**), 19.6 (**C10**).

HRMS: (ESI$^+$) $C_{17}H_{19}O_3$ $[M+H]^+$ found 239.1427, requires 239.1430 (−1.3 ppm).

(R)-1,2-bis(3-methoxyphenyl)propan-1-one ((***R*)-2r**)

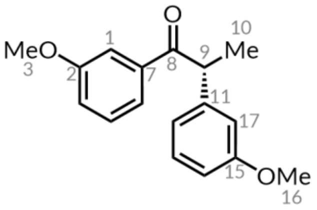

Following general procedure E, 1,1-bis-(3-methoxyphenyl)prop-2-en-1-ol (54 mg, 0.20 mmol), cobalt catalyst **(*R*,*R*)-Co(II)-5** (7.9 mg, 0.01 mmol), 1-fluoro-2,4,6-trimethylpyridinium tetrafluoroborate (91 mg, 0.40 mmol), 1,1,3,3-tetramethyldisiloxane (141 µL, 0.80 mmol), PhCl (1.7 mL) gave, after purification by column chromatography (hexane : CH_2Cl_2, 50 : 50), α-aryl ketone **(*R*)-2r** as a colourless oil (49 mg, 0.18 mmol, 90%).

$[\alpha]_D^{20}$ –150.9 (*c* 1.33 in $CHCl_3$).

Chiral HPLC: Chiralcel AD-H (hexane : IPA, 99 : 1), flow rate = 1 mL min^{-1}, λ = 220 nm, 30 °C) t_R(S): 6.7 min, t_R(R): 7.6 min, 2 : 98 er.

ν_{max} (film): 2931 (C–H), 1681 (C=O), 1595, 1485, 1257, 1041, 702.

^{1}H NMR (400 MHz, $CDCl_3$) δ_H: 7.54 – 7.75 (1H, m, **6-H**), 7.50 – 7.49 (1H, m, **1-H**), 7.29 – 7.26 (1H, m, **5-H**), 7.23 – 7.19 (1H, m, **13-H**), 7.03 – 7.01 (1H, m, **4-H**), 6.88 – 6.87 (1H, m, **12-H**), 6.88 – 6.81 (1H, m, **17-H**), 6.75 – 6.73 (1H, m, **14-H**), 4.63 (1H, q, *J* 6.8, **9-H**), 3.80 (3H, s, **3-H**), 3.76 (3H, s, **16-H**), 1.53 (3H, d, *J* 6.8, 3H, **10-H**).

^{13}C{^{1}H} NMR (126 MHz, $CDCl_3$) δ_C: 199.9 (**C8**), 160.0 (**C15**), 159.7 (**C7**), 143.0 (**C11**), 137.8 (**C2**), 129.9 (**C13**), 129.4 (**C5**), 121.4 (**C6**), 120.1 (**C12**), 119.3 (**C4**), 113.4 (**C17**), 113.0 (**C1**), 112.1 (**C14**), 55.3 (**C3**), 55.1 (**C16**), 48.0 (**C9**), 19.4 (**C10**).

HRMS: (ESI^+) $C_{17}H_{19}O_3$ $[M+H]^+$ found 271.1331, requires 271.1329 (+0.7 ppm).

(±)-1,2-Bis(2-chlorophenyl)propan-1-one (**(±)-2s**)

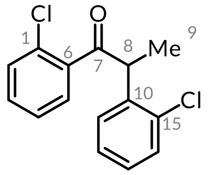

Following general procedure H, 1,1-bis(2-chlorophenyl)prop-2-en-1-ol (**1s**) (28 mg, 0.10 mmol), cobalt catalyst **(*R*,*R*)-Co(II)-5** (3.9 mg, 0.005 mmol), 1-fluoro-2,4,6-trimethylpyridinium tetrafluoroborate (45 mg, 0.20 mmol), 1,1,3,3-tetramethyldisiloxane (71 µL, 0.80 mmol), PhCl (0.9 mL) gave, after purification by preparative thin layer chromatography (hexane : diethyl ether, 80 : 20), α-aryl ketone **(±)-2s** as a colourless oil (19 mg, 0.70 mmol, 70%).

Chiral HPLC: Chiralcel OD-H (hexane : IPA, 99 : 1), flow rate = 0.6 mL min^{-1}, λ = 254 nm, 30 °C) t_R: 12.8 min, t_R: 16.5 min, 50 : 50 er.

ν_{max} (film): 3065, 2978, 1701 (C=O), 1589, 1474, 1433, 1034, 754.

^{1}H NMR (500 MHz, $CDCl_3$) δ_H: δ 7.35 (1H, d, *J* 7.9 Hz, **2-H**), 7.33 – 7.27 (4H, m, **3-H, 5-H, 11-H, 12-H**), 7.24 – 7.17 (2H, m, **4-H, 14-H**), 7.14 (1H, t, *J* 7.6 Hz, **13-H**), 5.08 (1H, q, *J* 6.9 Hz, **8-H**), 1.54 (3H, d, *J* 6.9 Hz, **9-H**).

^{13}C{^{1}H} NMR (126 MHz, $CDCl_3$) δ_C: 202.6 (**C7**), 139.0 (**C6**), 137.7 (**C10**), 134.0 (**C15**), 131.5 (**C3/5**), 131.3 (**C1**), 130.6 (**C2**), 129.9 (**C12**), 129.2 (**C11**), 128.8 (**C3/5**), 128.6 (**C13**), 127.4 (**C14**), 126.7 (**C4**), 47.9 (**C8**), 17.0 (**C9**).

HRMS: (ESI^+) $C_{15}H_{13}{}^{35}Cl_2NaO$ $[M+Na]^+$ found 301.0158, requires 301.0157 (+0.2 ppm).

(±)-1,2-Di-*o*-tolylpropan-1-one (**(±)-2t**)

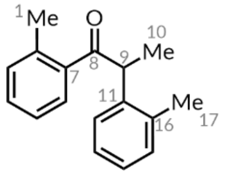

Following general procedure H, 1,1-di-*o*-tolylprop-2-en-1-ol (**1t**) (48 mg, 0.20 mmol), cobalt catalyst **(*R*,*R*)-Co(II)-5** (7.9 mg, 0.01 mmol), 1-fluoro-2,4,6-trimethylpyridinium tetrafluoroborate (91 mg, 0.40 mmol), 1,1,3,3-tetramethyldisiloxane (141 μL, 0.80 mmol), PhCl (1.7 mL) gave, after purification by column chromatography (hexane : CH_2Cl_2, 70 : 30), α-aryl ketone **(±)-2t** as colourless oil (38 mg, 0.16 mmol, 80%).

Chiral HPLC: Chiralcel OJ-H (hexane : IPA, 99 : 1), flow rate = 1.0 mL min^{-1}, λ = 254 nm, 30 °C) t_R: 8.8 min, t_R: 10.6 min, 50 : 50 er.

ν_{max} (film): 2970, 2927, 1681(C=O), 1454, 1220, 939.

^{1}H NMR (400 MHz, $CDCl_3$) δ_H: 7.41 (1H, d, *J* 7.7, **6-H**), 7.26 (1H, t, *J* 8.2, **4-H**), 7.18 – 7.08 (6H, m, **3-H**, **5-H**, **12-H**, **13-H**, **14-H**, **15H**), 4.71 (1H, q, *J* 6.8 Hz, **9-H**), 2.39 (3H, s, **1-H**), 2.33 (3H, s, **17-H**), 1.50 (3H, d, *J* 6.8 Hz, **10-H**).

^{13}C{^{1}H} NMR (126 MHz, $CDCl_3$) δ_C: 204.9 (**C8**), 139.2 (**C11**), 138.7 (**C7**), 138.0 (**C2**), 135.3 (**C16**), 131.7 (**C3**), 130.8 (**C4**), 130.7 (**C15**), 127.6 (**C6**), 127.1 (**C12**), 126.9 (**C14**), 126.6 (**C13**), 125.5 (**C5**), 46.5 (**C9**), 20.9 (**C1**), 19.8 (**C17**), 16.6 (**C10**).

HRMS: (ESI$^+$) $C_{17}H_{18}NaO$ $[M+Na]^+$ found 261.1251, requires 261.1255 (–1.5 ppm).

(*R*)-1,2-Bis(2-methoxyphenyl)propan-1-one ((***R*)-2u**)

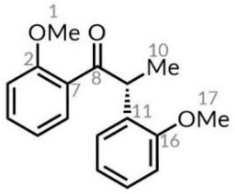

Following general procedure H, 1,1-bis(2-methoxyphenyl)prop-2-en-1-ol (**1u**) (55 mg, 0.20 mmol), cobalt catalyst **(*R*,*R*)-Co(II)-5** (7.9 mg, 0.01 mmol), 1-fluoro-2,4,6-trimethylpyridinium tetrafluoroborate (91 mg, 0.40 mmol), 1,1,3,3-tetramethyldisiloxane (141 µL, 0.80 mmol), PhCl (1.7 mL) gave, after purification by column chromatography (hexane : CH_2Cl_2, 70 : 30), α-aryl ketone **(*R*)-2u** as a colourless oil (19 mg, 0.07 mmol, 35%).

$[\alpha]_D^{20}$ +41.4 (*c* 0.5 in $CHCl_3$).

Chiral HPLC: Chiralcel OD-H (hexane : IPA, 99 : 1), flow rate = 1 mL min^{-1}, λ = 254 nm, 30 °C) t_R (*S*): 17.8 min, t_R (*R*): 22.1 min, 66 : 34 er.

ν_{max} (film): 2968, 2933, 1681(C=O), 1597, 1246, 1026.

^{1}H NMR (400 MHz, $CDCl_3$) δ_H: 7.50 (1H, dd, *J* 7.6, 1.8, **12-H**), 7.32 – 7.28 (1H, m, **14-H**), 7.14 – 7.11 (2H, m, **4-H**, **6-H**), 6.90 – 6.87 (1H, m, **13-H**), 6.85 – 6.82 (1H, m, **3-H/5-H**), 6.78 (1H, d, *J* 8.3, **15-H**), 6.75 – 6.73 (1H, m, **3-H/5-H**) 4.88 (1H, q, *J* 6.9, **9-H**), 3.76 (3H, s, **17-H**), 3.71 (3H, s, **1-H**), 1.45 (3H, d, *J* 6.9, **10-H**).

^{13}C{^{1}H} NMR (126 MHz, $CDCl_3$) δ_C: 200.4 (**C8**), 157.5 (**C16**),156.6 (**C2**), 132.3 (**C14**), 130.5 (**C7**), 130.2 (**C12**), 129.3 (**C11**), 128.9 (**C4/C6**), 127.8 (**C4/C6**), 120.6 (**C13**), 120.3 (**C3/C5)**, 111.1 (**C15**), 110.4 (**C3/C5)**, 55.4 (**C17**), 55.2 (**C1**), 46.4 (**C9**), 16.6 (**C10**).

HRMS: (ESI$^+$) $C_{17}H_{18}NaO_3$ [M+Na]$^+$ found 293.1159, requires 293.1154 (+1.7 ppm).

(*R*)-1,2-Bis(2-fluorophenyl)propan-1-one (**(*R*)-2v**)

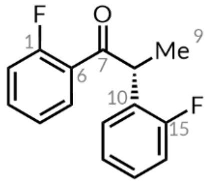

Following general procedure H, 1,1-bis(2-fluorophenyl)prop-2-en-1-ol (**1v**) (49 mg, 0.20 mmol), cobalt catalyst **(*R*,*R*)-Co(II)-5** (7.9 mg, 0.01 mmol), 1-fluoro-2,4,6-trimethylpyridinium tetrafluoroborate (91 mg, 0.40 mmol), 1,1,3,3-tetramethyldisiloxane (141 μL, 0.80 mmol), PhCl (1.7 mL) gave, after purification by column chromatography (hexane : CH_2Cl_2, 70 : 30), α-aryl ketone **(*R*)-2v** as a colourless oil (30 mg, 0.12 mmol, 60%).

$[\alpha]_D^{20}$ −15.4 (*c* 0.5 in $CHCl_3$).

Chiral HPLC: Chiralcel OD-H (hexane : IPA, 99 : 1), flow rate = 1 mL min^{-1}, λ = 254 nm, 30 °C) t_R (*S*): 6.5 min, t_R (*R*): 7.5 min, 35 : 65 er.

ν_{max} (film): 2980, 2933, 1683 (C=O), 1608, 1450, 1213, 956.

^{1}H NMR (400 MHz, $CDCl_3$) δ_H: 7.77 (1H, td, *J* 7.6, 1.9, **5-H**), 7.45 – 7.38 (1H, m, **3-H**), 7.22 – 7.13 (3H, m, **4-H**, **11-H**, **13-H**), 7.08 – 6.96 (3H, m, **2-H**, **12-H**, **14-H**), 4.85 (1H, q, *J* 6.9, **8-H**), 1.52 (3H, d, *J* 6.9, **9-H**).

^{13}C{^{1}H} NMR (126 MHz, $CDCl_3$) δ_C: 199.1 (d, *J* 4.2, **C7**), 161.1 (d, *J* 254.3, **C1**), 160.6 (d, *J* 246.1, **C15**), 134.2 (d, *J* 9.0, **C3**), 131.0 (d, *J* 2.8, **C5**), 129.3 (d, *J* 4.1, **C13**), 128.8 (d, *J* 8.3, **C11**), 128.0 (d, *J* 15.3, **C10**), 125.9 (d, *J* 13.1, **C6**), 124.5 (d, *J* 3.6, **C4/C12**), 124.4 (d, *J* 3.7, **C4/C12**), 116.6 (d, *J* 23.6, **C2**), 115.6 (d, *J* 22.3, **C14**), 45.1 (d, *J* 6.1, **C8**), 17.4 (**C9**).

^{19}F NMR (282 MHz, $CDCl_3$) δ_F: −110.5 (d, *J* 5.7, **F1**), −118.1 (d, *J* 6.0, **F15**).

HRMS: (ESI$^+$) $C_{15}H_{12}F_2NaO$ $[M+Na]^+$ found 269.0749, requires 269.0754 (+1.8 ppm).

(R)-1,2-Di(benzofuran-2-yl)propan-1-one (**(R)-2w**)

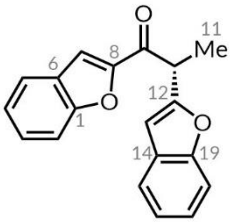

Following general procedure H, 1,1-di(benzofuran-2-yl)prop-2-en-1-ol (**1w**) (58 mg, 0.20 mmol), cobalt catalyst **(R,R)-Co(II)-5** (7.9 mg, 0.01 mmol), 1-fluoro-2,4,6-trimethylpyridinium tetrafluoroborate (91 mg, 0.40 mmol), 1,1,3,3-tetramethyldisiloxane (141 μL, 0.80 mmol), PhCl (1.7 mL) gave, after purification by column chromatography (hexane : CH_2Cl_2, 70 : 30), α-aryl ketone **(R)-2w** as a white solid (53 mg, 0.18 mmol, 91%).

mp 83 – 85 °C

$[\alpha]_D^{20}$ +86.2 (*c* 0.5 in $CHCl_3$).

Chiral HPLC: Chiralcel OD-H (hexane : IPA, 99 : 1), flow rate = 1 mL min^{-1}, λ = 254 nm, 30 °C) t_R (S): 18.9 min, t_R (R): 20.4 min, 8 : 92 er.

ν_{max} (film): 2926, 1681 (C=O), 1552, 1454, 1255, 935.

^{1}H NMR (400 MHz, $CDCl_3$) δ_H: 7.70 (1H, d, *J* 7.9, **4-H**), 7.62 (1H, s, **7-H**), 7.58 (1H, d, *J* 8.4, **3-H**), 7.51 – 7.46 (2H, m, **2-H, 15-H**), 7.42 (1H, d, *J* 8.1, **18-H**), 7.31 (1H, t, *J* 7.5, **5-H**), 7.24 – 7.21 (1H, m, **17-H**), 7.18 (1H, t, *J* 7.4, **16-H**), 6.64 (1H, s, **13-H**), 4.87 (1H, q, *J* 7.1, **10-H**), 1.72 (3H, d, *J* 7.1, **11-H**).

^{13}C{^{1}H} NMR (126 MHz, $CDCl_3$) δ_C: 188.5 (**C9**), 156.4 (**C12**), 155.8 (**C6**), 154.9 (**C19**), 151.7 (**C8**), 128.6 (**C2**), 128.5 (**C14**), 127.1 (**C1**), 124.1 (**C5**), 124.0 (**C17**), 123.5 (**C4**), 122.8 (**C16**), 120.9 (**C15**), 114.4 (**C7**), 112.6 (**C3**), 111.2 (**C18**), 103.8 (**C13**), 42.5 (**C10**), 15.6 (**C11**).

HRMS: (ESI$^+$) $C_{19}H_{14}NaO_3$ $[M+Na]^+$ found 313.0841, requires 313.0841 (0.0 ppm).

(*S*)-1,2-Bis(benzothiophen-2-yl)propan-1-one (**(*S*)-2x**)

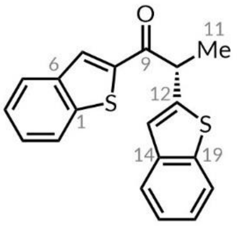

Following general procedure H, 1,1-bis(benzo[b]thiophen-2-yl)prop-2-en-1-ol (**1x**) (64 mg, 0.20 mmol), cobalt catalyst **(*R*,*R*)-Co(II)-5** (7.9 mg, 0.01 mmol), 1-fluoro-2,4,6-trimethylpyridinium tetrafluoroborate (91 mg, 0.40 mmol), 1,1,3,3-tetramethyldisiloxane (141 μL, 0.80 mmol), PhCl (1.7 mL) gave, after purification by column chromatography (hexane : CH_2Cl_2, 70 : 30), α-aryl ketone **(*S*)-2x** as a beige solid (48 mg, 0.15 mmol, 75%).

mp 124 – 126 °C

$[\alpha]_D^{20}$ +98.4 (*c* 0.5 in $CHCl_3$).

Chiral HPLC: Chiralcel AD-H (hexane : IPA, 97 : 3), flow rate = 1 mL min^{-1}, λ = 254 nm, 30 °C) t_R (*R*): 22.0 min, t_R (*S*): 29.3 min, 4 : 96 er.

ν_{max} (film): 3057, 2929, 1658 (C=O), 1512, 1217, 894.

^{1}H NMR (400 MHz, $CDCl_3$) δ_H: 8.06 (1H, s, **7-H**), 7.88 – 7.84 (2H, m, **5-H, 4-H**), 7.70 (1H, d, *J* 7.8, **16-H**), 7.68 (1H, d, *J* 7.5, **15-H**), 7.46 – 7.43 (1H, m, **3-H**), 7.40 – 7.37 (1H, m, **2-H**), 7.32 – 7.25 (2H, m, **17-H, 18-H**), 7.21 (1H, s, **13-H**), 4.98 (1H, q, *J* 6.8, **10-H**), 1.75 (3H, d, *J* 6.8, **11-H**).

^{13}C{^{1}H} NMR (126 MHz, $CDCl_3$) δ_C: 193.1 (**C9**), 144.2 (**C12**), 142.8 (**C1**), 142.5 (**C8**), 139.8 (**C14**), 139.6 (**C19**), 139.1 (**C6**), 130.0 (**C7**), 127.7 (**C3**), 126.2 (**C5**), 125.1 (**C2**), 124.4 (**C18**), 124.2 (**C17**), 123.4 (**C15**), 123.0 (**C4**), 122.3 (**C16**), 121.8 (**C13**), 44.6 (**C10**), 19.7 (**C11**).

HRMS: (ESI$^+$) $C_{19}H_{14}NaOS_2$ $[M+Na]^+$ found 345.0376, requires 345.0384 (–2.3 ppm).

(*S*)-1,2-Di(thiophen-2-yl)propan-1-one (**(*S*)-2y**)

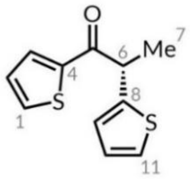

Following general procedure H, 1,1-bis(2-thiophenyl)prop-2-en-1-ol (**1y**) (46 mg, 0.20 mmol), cobalt catalyst **(*R*,*R*)-Co(II)-5** (7.9 mg, 0.01 mmol), 1-fluoro-2,4,6-trimethylpyridinium tetrafluoroborate (91 mg, 0.40 mmol), 1,1,3,3-tetramethyldisiloxane (141 μL, 0.80 mmol), PhCl (1.7 mL) gave, after purification by column chromatography (hexane : CH_2Cl_2, 60 : 40), α-aryl ketone **(*S*)-2y** as a yellow oil (23 mg, 0.11 mmol, 53%).

$[\alpha]_D^{20}$ –39.8 (*c* 1.2 in $CHCl_3$).

Chiral HPLC: Chiralcel OD-H (hexane : IPA, 99 : 1), flow rate = 0.6 mL min^{-1}, λ = 254 nm, 30 °C) t_R (*R*): 23.4 min, t_R (*S*): 16.9 min, 3 : 97 er.

ν_{max} (film): 3102, 2976, 2929, 1657 (C=O), 1412, 1236, 851.

^{1}H NMR (400 MHz, $CDCl_3$) δ_H: 7.78 (1H, dd, *J* 3.8, 1.1, **3-H**), 7.63 (1H, dd, *J* 5.0, 1.1, **1-H**), 7.19 (1H, dd, *J* 4.9, 1.5, **11-H**), 7.11 (1H, dd, *J* 5.0, 3.8, **2-H**), 6.96 – 6.90 (2H, m, **9-H, 10-H**), 4.82 (1H, q, *J* 7.0, **6-H**), 1.64 (3H, d, *J* 7.0, **7-H**).

^{13}C{^{1}H} NMR (126 MHz, $CDCl_3$) δ_C: 192.2 (**C5**), 143.6 (**C8**), 143.2 (**C4**), 134.3 (**C1**), 132.7 (**C3**), 128.3 (**C2**), 127.0 (**C10**), 125.2 (**C9**), 124.7 (**C11**), 44.1 (**C6**), 20.2 (**C7**).

HRMS: (ESI$^+$) $C_{11}H_{10}NaOS_2$ $[M+Na]^+$ found 245.0066, requires 245.0065 (–0.41 ppm).

(R)-1,2-Bis(6-methoxynaphthalen-2-yl)propan-1-one (**(R)-2z**)

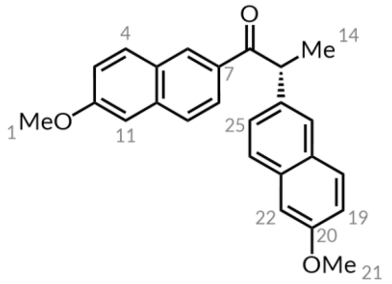

Following general procedure H, 1,1-bis(6-methoxynaphthalen-2-yl)prop-2-en-1-ol (**1z**) (62 mg, 0.20 mmol), cobalt catalyst **(R,R)-Co(II)-5** (7.9 mg, 0.01 mmol), 1-fluoro-2,4,6-trimethylpyridinium tetrafluoroborate (91 mg, 0.40 mmol), 1,1,3,3-tetramethyldisiloxane (141 μL, 0.80 mmol), PhCl (1.7 mL) gave, after purification by column chromatography (hexane : CH_2Cl_2, 70 : 30), α-aryl ketone **(R)-2z** as a white solid (53 mg, 0.17 mmol, 85%).

mp 147 – 149 °C

$[\alpha]_D^{20}$ +93.6 (*c* 0.5 in $CHCl_3$).

Chiral HPLC: Chiralcel OD-H (hexane : IPA, 97 : 3), flow rate = 1 mL min^{-1}, λ = 254 nm, 30 °C) t_R (S): 16.8 min, t_R (R): 20.1 min, 3 : 97 er.

ν_{max} (film): 2968, 2931, 1672 (C=O), 1479, 1267, 1029, 854.

^{1}H NMR (400 MHz, $CDCl_3$) δ_H: 8.45 (1H, s, **6-H**), 8.02 (1H, dd, *J* 8.7, 1.8, **8-H**), 7.77 (1H, d, *J* 9.0, **4-H**), 7.70 (1H, s, **16-H**), 7.70 – 7.65 (3H, m, **9-H, 18-H, 24-H**), 7.45 (1H, dd, *J* 8.5, 1.8, **25-H**), 7.14 (1H, dd, *J* 9.0, 2.5, **3-H**), 7.10 (1H, dd, *J* 9.0, 2.5, **19-H**), 7.07 (2H, app. t, **11-H, 22-H**), 4.95 (1H, q, *J* 6.8, **13-H**), 3.90 (3H, s, **1-H**), 3.88 (3H, s, **21-H**), 1.65 (3H, d, *J* 6.8, **14-H**).

^{13}C{^{1}H} NMR (126 MHz, $CDCl_3$) δ_C: 200.3 (**C12**), 159.8 (**C2**), 157.7 (**C20**), 137.2 (**C10**), 137.1 (**C15**), 133.6 (**C23**), 132.1 (**C7**), 131.3 (**C4**), 130.5 (**C6**), 129.4 (**C18**), 129.3 (**C17**), 127.9 (**C5**), 127.7 (**C9**), 127.1 (**C24**), 126.6 (**C25**), 126.4 (**C16**), 125.5 (**C8**), 119.7 (**C3**), 119.1 (**C19**), 105.7 (**C11/C22**), 105.7 (**C11/C22**), 55.5 (**C1**), 55.4 (**C21**), 47.8 (**C13**), 19.8 (**C14**).

HRMS: (ESI$^+$) $C_{25}H_{22}NaO_3$ [M+Na]$^+$ found 393.1472, requires 393.1467 (+1.2 ppm).

(±)-2-Methyl-1,2-diphenylbutan-1-one (**2aa**)

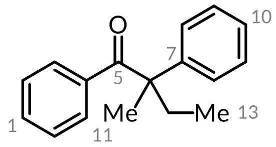

Following general procedure H, 2-methylene-1,1-diphenylbutan-1-ol (**1aa**) (48 mg, 0.20 mmol), cobalt catalyst **(*R*,*R*)-Co(II)-5** (7.9 mg, 0.01 mmol), 1-fluoro-2,4,6-trimethylpyridinium tetrafluoroborate (91 mg, 0.40 mmol), 1,1,3,3-tetramethyldisiloxane (141 µL, 0.80 mmol), PhCl (1.7 mL) gave, after purification by column chromatography (hexane : diethyl ether, 98 : 2 to 80 : 20), α-aryl ketone **2aa** as a yellow oil (43 mg, 0.18 mmol, 89%). NMR spectroscopic data were in accordance with the literature.[22]

Chiral HPLC: Chiralcel OJ-H (hexane : IPA, 0.5 : 99.5), flow rate = 0.5 mL min^{-1}, λ = 254 nm, 30 °C) t_R (*R*): 13.3 min, t_R (*S*): 15.1 min, 49 : 51 er.

^{1}H NMR (500 MHz, $CDCl_3$) δ_H: 7.47 – 7.43 (2H, m, **3-H**), 7.38 – 7.33 (3H, m, **9-H**, **1-H**), 7.32 – 7.24 – 7.19 (3H, m, **8-H**, **10-H**), 7.24 – 7.19 (2 H, m, **2-H**), 2.18 (1H, m, **12-H**), 2.12 – 2.03 (1H, m, **12-H**), 1.56 (3H, s, **11-H**), 0.76 (3H, t, *J* 7.4, **13-H**).

^{13}C{^{1}H} NMR (126 MHz, $CDCl_3$) δ_C: 204.0 (**C5**), 144.5 (**C7**), 137.1 (**C4**), 131.7 (**C1**), 129.6 (**C3**), 129.0 (**C9**), 128.1 (**C2**), 126.9 (**C10**), 126.4 (**C8**), 55.1 (**C6**), 32.2 (**C12**), 23.9 (**C11**), 8.8 (**C13**).

Isomerisation of alkene **1ab**

Following general procedure H, 1,1-diphenylbut-2-en-1-ol (**1ab**) (99 : 1 *Z*/*E*) (45 mg, 0.20 mmol), cobalt catalyst **(*R*,*R*)-Co(II)-5** (7.9 mg, 0.01 mmol), 1-fluoro-2,4,6-trimethylpyridinium tetrafluoroborate (91 mg, 0.40 mmol), 1,1,3,3-tetramethyldisiloxane (141 µL, 0.80 mmol), PhCl (1.7 mL) gave a ^{1}H NMR yield of 82 % of ***Z*-1ab** 6 % ***E*-1ab**. Purification of the crude product by column chromatography gave the mixture of geometric isomers as a colourless oil (39 mg, 174 mmol, 87 %).

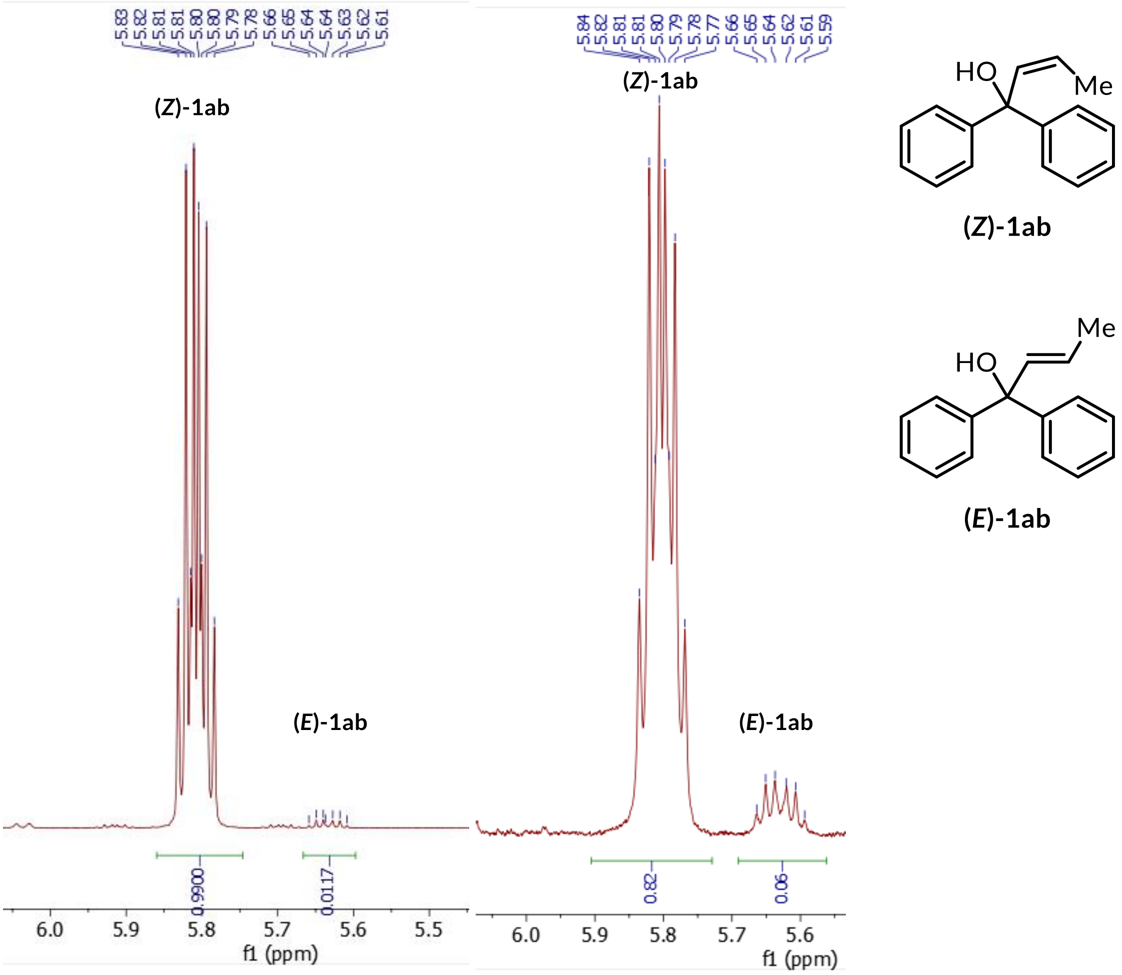

Substrate **1ab** before and after subjection to the reaction conditions (general procedure H).

(±)-1-Phenyl-2-(*p*-tolyl)propan-1-one (**(±)-4a**) and (±)-2-phenyl-1-(*p*-tolyl)propan-1-one (**(±)-5a**)

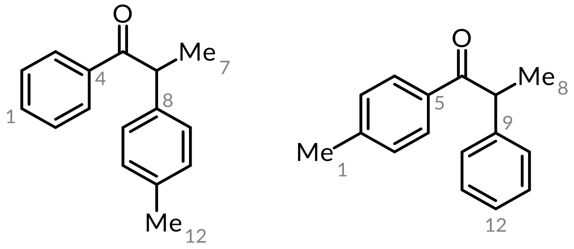

(±)-4a **(±)-5a**

Following general procedure H, 1-(4-methylphenyl)-1-phenylprop-2-en-1-ol (**3a**) (45 mg, 0.20 mmol), cobalt catalyst **Co(II)-6** (5.8 mg, 0.01 mmol), 1-fluoro-2,4,6-trimethylpyridinium tetrafluoroborate (91 mg, 0.40 mmol), 1,1,3,3-tetramethyldisiloxane (141 μL, 0.80 mmol), PhCl (1.7 mL) gave a 3.10 : 1.00 mixture of α-aryl ketones **(±)-4a** and **(±)-5a** as determined by ^{1}H NMR spectroscopy of the crude reaction mixture. After purification by column chromatography (hexane : CH_2Cl_2, 60 : 40), α-aryl ketones **(±)-4a** and **(±)-5a** were isolated as a 2.94 : 1.00 mixture as a yellow oil (45 mg, 0.20 mmol, 100%). NMR spectroscopic data were in accordance with the literature.[23]

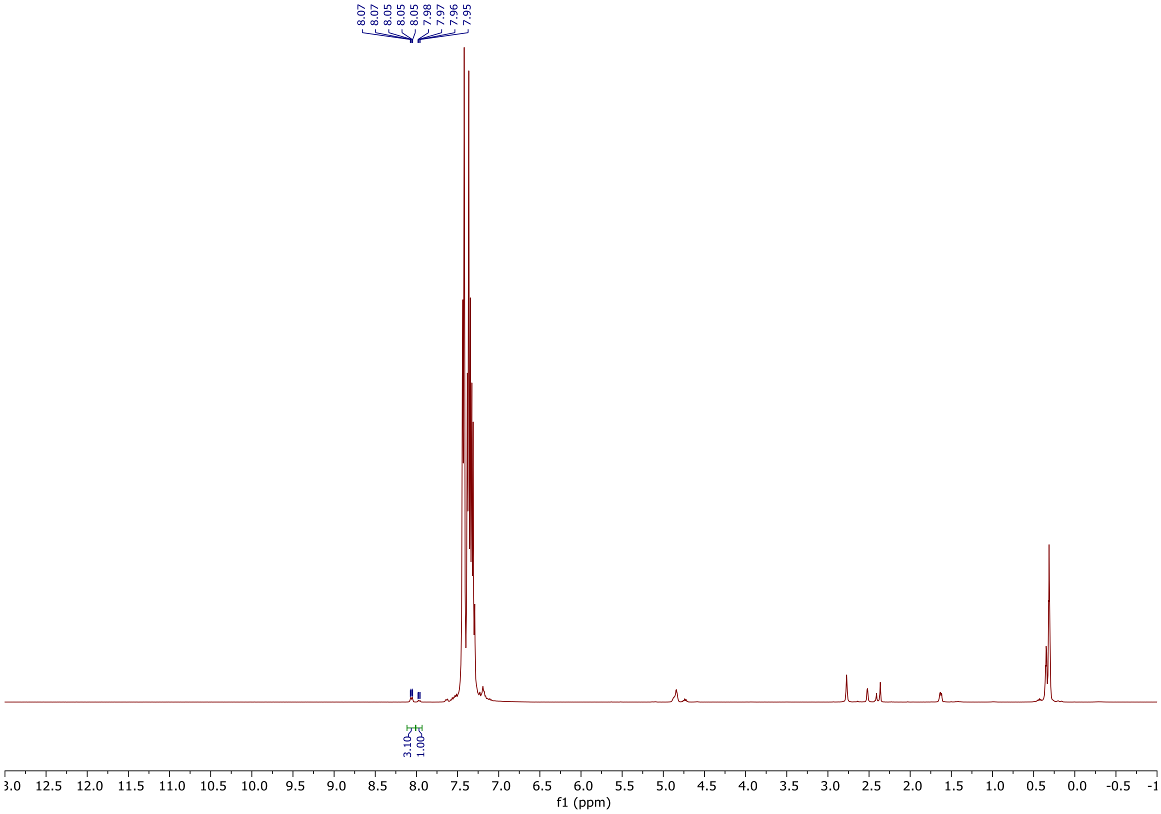

^{1}H NMR spectrum of the crude reaction mixture in $CDCl_3$

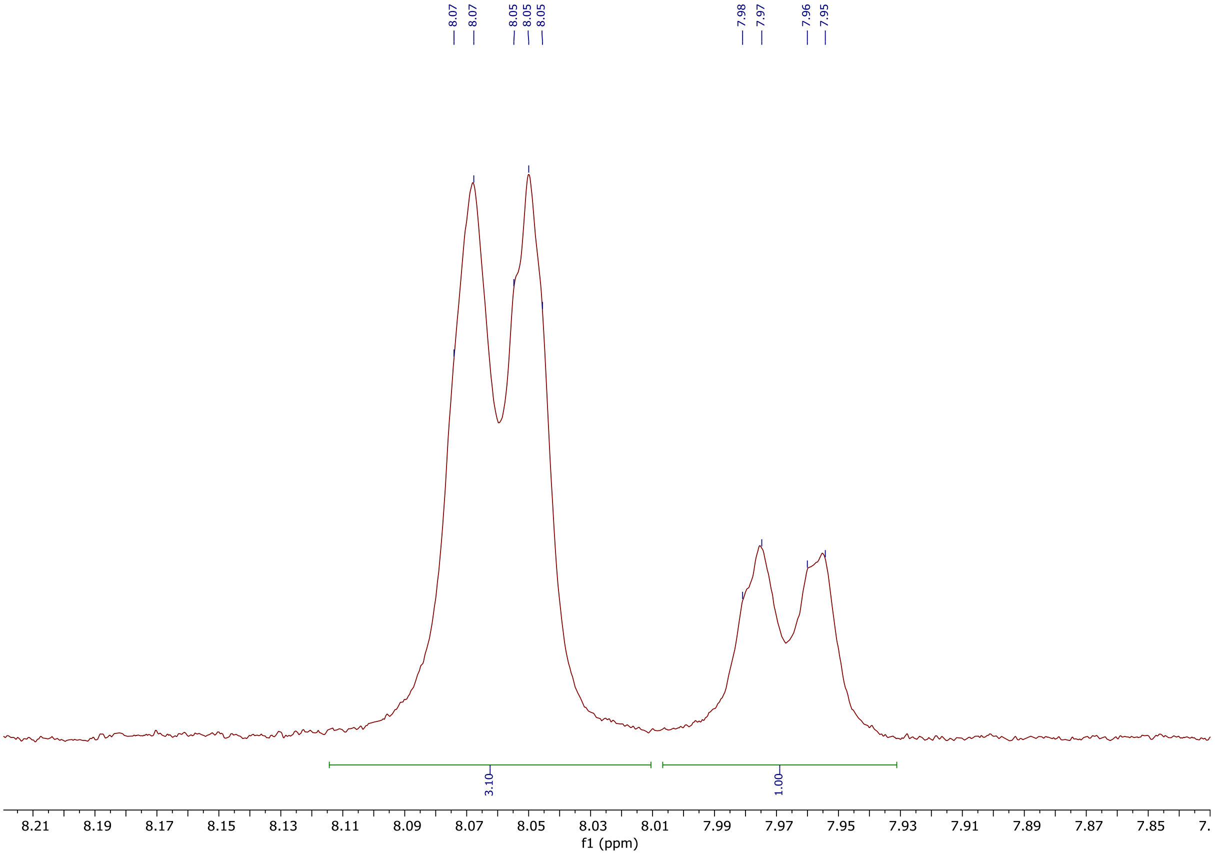

^{1}H NMR spectrum of the crude reaction mixture in $CDCl_3$

(±)-4a:

^{1}H NMR (500 MHz, $CDCl_3$) δ_H: distinguishable/diagnostic peaks only: 7.96 (2H, d, *J* 7.9, **3-H**), 7.47 (1H, t, *J* 7.8, **1-H**), 7.38 (2H, t, *J* 7.7, **2-H**), 7.11 (2H, d, *J* 7.9, **10-H**), 4.71 – 4.63 (1H, m, **6-H**) 2.29 (3H, s **12-H**), 1.53 (3H, d, *J* 6.7, **7-H**).

^{13}C{^{1}H} NMR (126 MHz, $CDCl_3$) δ_C: 200.6 (**C5**), 138.6 (**C8**), 136.7 (**C11**), 136.6 (**C4**), 132.8 (**C1**), 129.8 (**C10**), 128.9 (**C3**), 128.6 (**C2**), 127.8 (**C9**), 47.8 (**C6**), 21.1 (**C12**), 19.6 (**C7**).

(±)-5a:

^{1}H NMR (500 MHz, $CDCl_3$) δ_H: distinguishable/diagnostic peaks only: 7.87 (2H, d, *J* 8.0, **4-H**), 4.71 – 4.63 (2H, m, **7-H**), 2.35 (3H, s, **1-H**), 1.54 (3H, d, *J* 6.9, **8-H**).

^{13}C{^{1}H} NMR (126 MHz, $CDCl_3$) δ_C: 200.1 (**C6**), 143.7 (**C5**), 141.8 (**C9**), 134.1 (**C2**), 129.3 (**C3**), 129.1 (**C4/C10**), 129.0 (**C4/C10**), 47.8 (**C7**), 21.7 (**C1**), 19.6 (**C8**).

(±)-1-Phenyl-2-(4-fluorophenyl)propan-1-one (**(±)-4b**) and (±)-1-(4-fluorophenyl)-2-phenylpropan-1-one (**(±)-5b**)

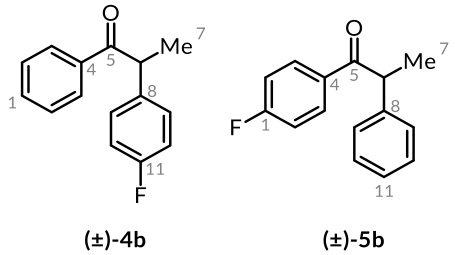

Following general procedure H, 1-(4-fluorophenyl)-1-phenylprop-2-en-1-ol (**3b**) (46 mg, 0.20 mmol), cobalt catalyst **Co(II)-6** (5.8 mg, 0.01 mmol), 1-fluoro-2,4,6-trimethylpyridinium tetrafluoroborate (91 mg, 0.40 mmol), 1,1,3,3-tetramethyldisiloxane (141 µL, 0.80 mmol), PhCl (1.7 mL) gave a 0.82 : 1.00 mixture of α-aryl ketones **(±)-4b** and **(±)-5b** as determined by ^{19}F NMR spectroscopy of the crude reaction mixture. After purification by column chromatography (hexane : CH_2Cl_2, 60 : 40), α-aryl ketones **(±)-4b** and **(±)-5b** were isolated as a 0.75 : 1.00 mixture as a yellow oil (40 mg, 0.18 mmol, 88%). NMR spectroscopic data were in accordance with the literature.[23]

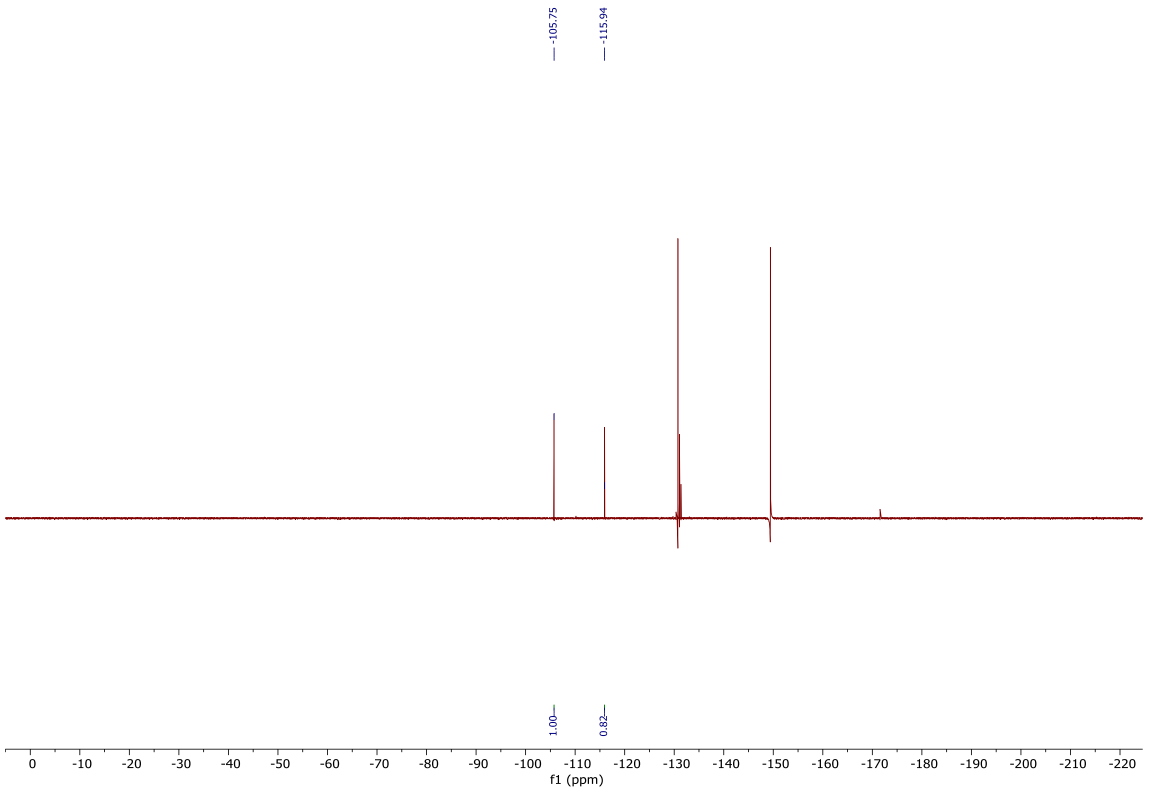

Quantitative ^{19}F NMR spectrum of the crude reaction mixture in $CDCl_3$

(±)-4b

^{1}H NMR (500 MHz, $CDCl_3$) δ_H: 8.02 – 7.91 (2H, m, **3-H**), 7.53 – 7.47 (1H, m, **1-H**), 7.40 (2H, t, *J* 7.7, **2-H**), 7.33 – 7.23 (2H, m, **9-H**), 7.01 – 6.95 (2H, m, **10-H**), 4.69 (1H, q, *J* 6.9, **6-H**), 1.52 (3H, d, *J* 6.9, **7-H**).

^{13}C{^{1}H} NMR (126 MHz, $CDCl_3$) δ_C: 200.4 (**C5**), 161.9 (d, *J* 245.5, **C11**), 137.2 (d, *J* 3.3, **C8**), 136.4 (**C4**), 133.1 (**C1**), 129.4 (d, *J* 8.0, **C9**), 128.9 (**C3**), 128.7 (**C2**), 115.7, (d, *J* 21.8, **C10**), 47.1 (**C6**), 19.7 (**C7**).

^{19}F NMR (471 MHz, $CDCl_3$) δ_F: –115.7 – –115.9 (m, **F**).

(±)-5b

^{1}H NMR (500 MHz, $CDCl_3$) δ_H: 8.02 – 7.91 (2H, m, **3-H**), 7.33 – 7.23 (5H, m, **9-H, 10-H, 11-H**), 7.04 (2H, t, *J* 8.6, **2-H**), 4.69 (1H, q, *J* 6.9, **6-H**), 1.52 (3H, d, *J* 6.9, **7-H**).

^{13}C{^{1}H} NMR (126 MHz, $CDCl_3$) δ_C: 198.8 (**C5**), 165.6 (d, *J* 254.7, **C1**), 141.5 (**C8**), 133.0 (d, *J* 3.1, **C4**), 131.5 (d, *J* 9.2, **C3**), 129.2 (**C10**), 127.8 (**C9**), 127.2 (**C11**), 116.0 (d, *J* 21.4, **C2**), 48.1 (**C6**), 19.6 (**C7**).

^{19}F NMR (471 MHz, $CDCl_3$) δ_F: –105.5 – –105.8 (m, **F**).

(±)-1-Phenyl-2-(4-chlorophenyl)propan-1-one (**(±)-4c**) and (±)-1-(4-chlorophenyl)-2-phenylpropan-1-one (**(±)-5c**)

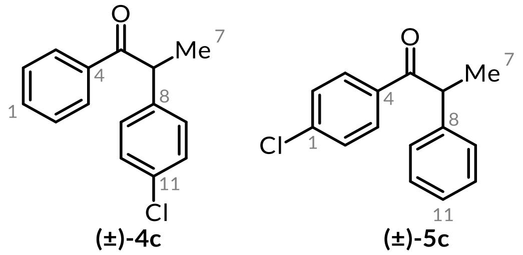

Following general procedure H, 1-(4-chlorophenyl)-1-phenylprop-2-en-1-ol (**3c**) (49 mg, 0.20 mmol), cobalt catalyst **Co(II)-6** (5.8 mg, 0.01 mmol), 1-fluoro-2,4,6-trimethylpyridinium tetrafluoroborate (91 mg, 0.40 mmol), 1,1,3,3-tetramethyldisiloxane (141 µL, 0.80 mmol), PhCl (1.7 mL) gave a 0.41 : 1.00 mixture of α-aryl ketones **(±)-4c** and **(±)-5c** as determined by ^{1}H NMR spectroscopy of the crude reaction mixture. After purification by column chromatography (hexane : CH_2Cl_2, 60 : 40), α-aryl ketones **(±)-4c** and **(±)-5c** were isolated as a 0.41 : 1.00 mixture as a yellow oil (49 mg, 0.20 mmol, 100%). NMR spectroscopic data were in accordance with the literature.[24,25]

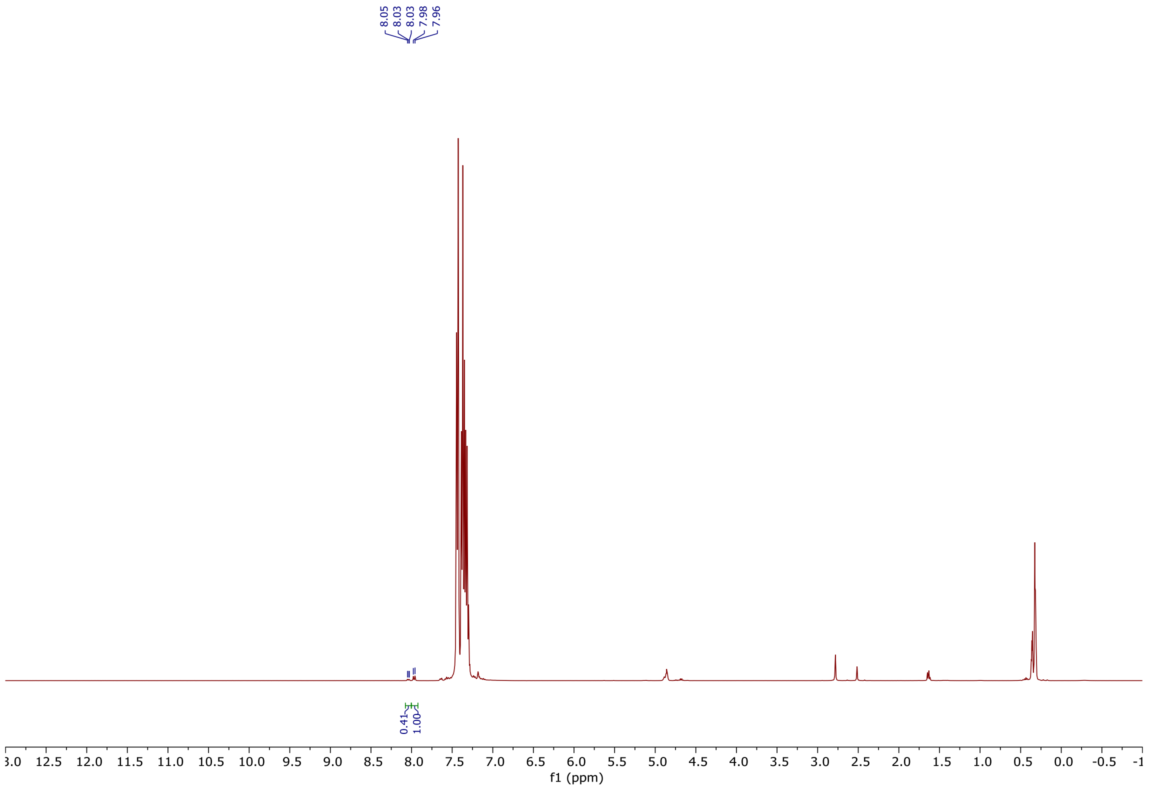

^{1}H NMR spectrum of the crude reaction mixture in $CDCl_3$

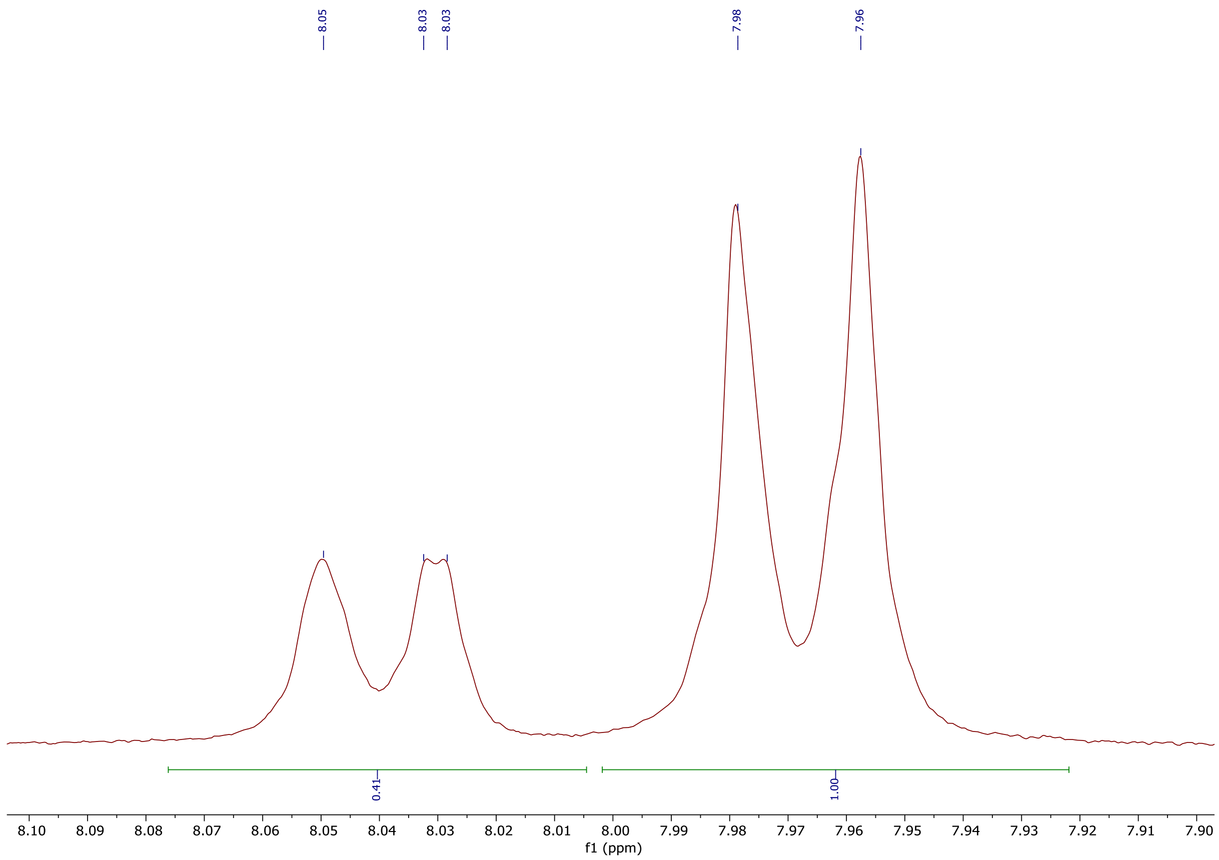

^{1}H NMR spectrum of the crude reaction mixture in $CDCl_3$

(±)-4c:

^{1}H NMR (500 MHz, $CDCl_3$) δ_H: distinguishable/diagnostic peaks only: 7.97 – 7.91 (2H, m, **3-H**), 7.53 – 7.47 (1H, m, **1-H**), 7.40 (2H, t, *J* 8.4, **2-H**), 4.68 (1H, q, *J* 6.9, **6-H**), 1.52 (3H, d, *J* 6.9, **7-H**).

^{13}C{^{1}H} NMR (126 MHz, $CDCl_3$) δ_C: distinguishable/diagnostic peaks only: 200.1 (**C5**), 140.0 (**C8**), 133.1 (**C1**), 128.8 (**C3**), 128.7 (**C2**), 47.2 (**C6**), 19.6 (**C7**).

(±)-5c:

^{1}H NMR (500 MHz, $CDCl_3$) δ_H: distinguishable/diagnostic peaks only: 7.91 – 7.86 (2H, m, **3-H**), 7.37 – 7.33 (2H, m, **2-H**), 4.62 (1H, q, *J* 6.8, **6-H**), 1.54 (3H, d, *J* 6.8, **7-H**).

^{13}C{^{1}H} NMR (126 MHz, $CDCl_3$) δ_C: δ 199.2 (**C5**), 141.3 (**C8**), 139.3 (**C1**), 134.9 (**C4**), 130.3 (**C3**), 129.2 (**C10**), 128.9 (**C2**), 127.8 (**C9**), 127.1 (**C11**), 48.2 (**C6**), 19.6 (**C7**).

(±)-1-Phenyl-2-(3-fluorophenyl)propan-1-one (**(±)-4d**) and (±)-1-(3-fluorophenyl)-2-phenylpropan-1-one (**(±)-5d**)

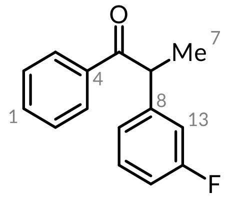

(±)-4d

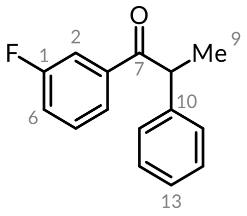

(±)-5d

Following general procedure H, 1-(3-fluorophenyl)-1-phenylprop-2-en-1-ol (**3d**) (46 mg, 0.20 mmol), cobalt catalyst **Co(II)-6** (5.8 mg, 0.01 mmol), 1-fluoro-2,4,6-trimethylpyridinium tetrafluoroborate (91 mg, 0.40 mmol), 1,1,3,3-tetramethyldisiloxane (141 μL, 0.80 mmol), PhCl (1.7 mL) gave a mixture 0.17 : 1.00 mixture of α-aryl ketones **(±)-4d** and **(±)-5d** as determined by quantitative ^{19}F NMR spectroscopy of the crude reaction mixture. After purification by column chromatography (hexane : CH_2Cl_2, 60 : 40), α-aryl ketones **(±)-4d** and **(±)-5d** were isolated as a 0.16 : 1.00 mixture as a yellow oil (41 mg, 0.18 mmol, 89%). NMR spectroscopic data were in accordance with the literature.[26]

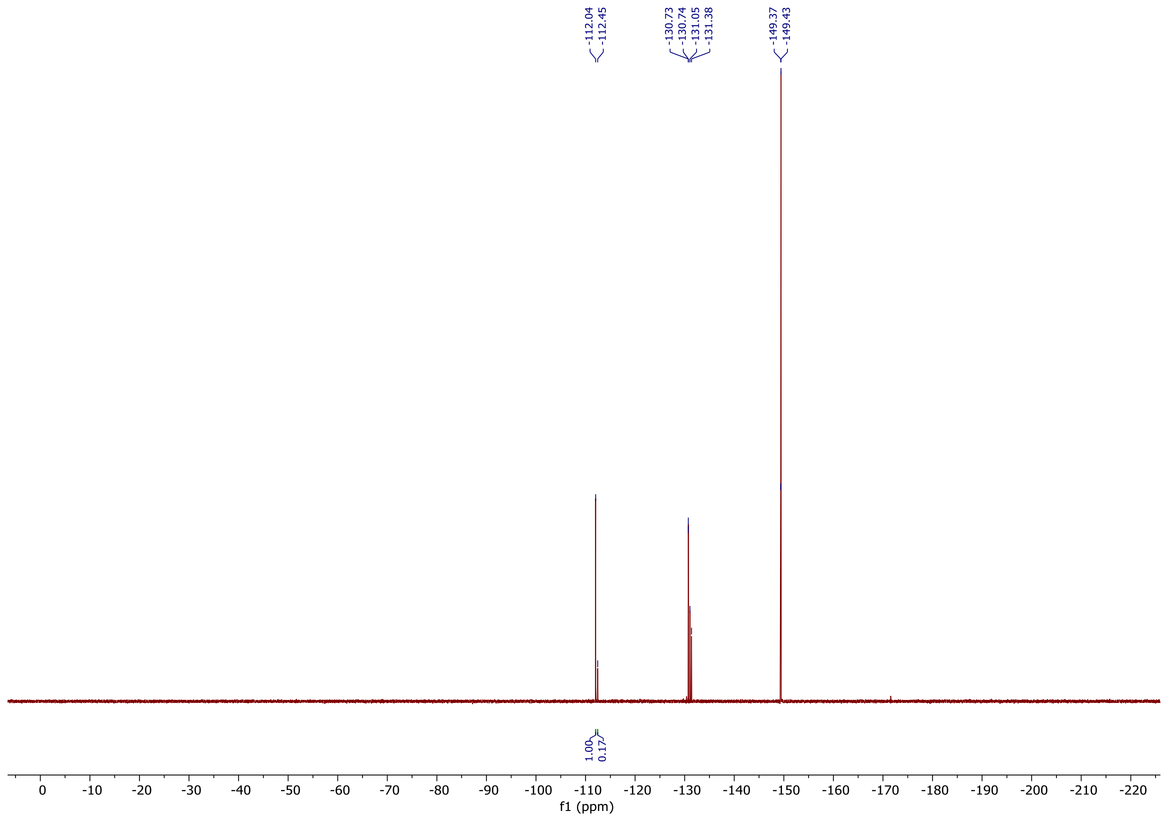

Quantitative ^{19}F NMR spectrum of the crude reaction mixture in $CDCl_3$

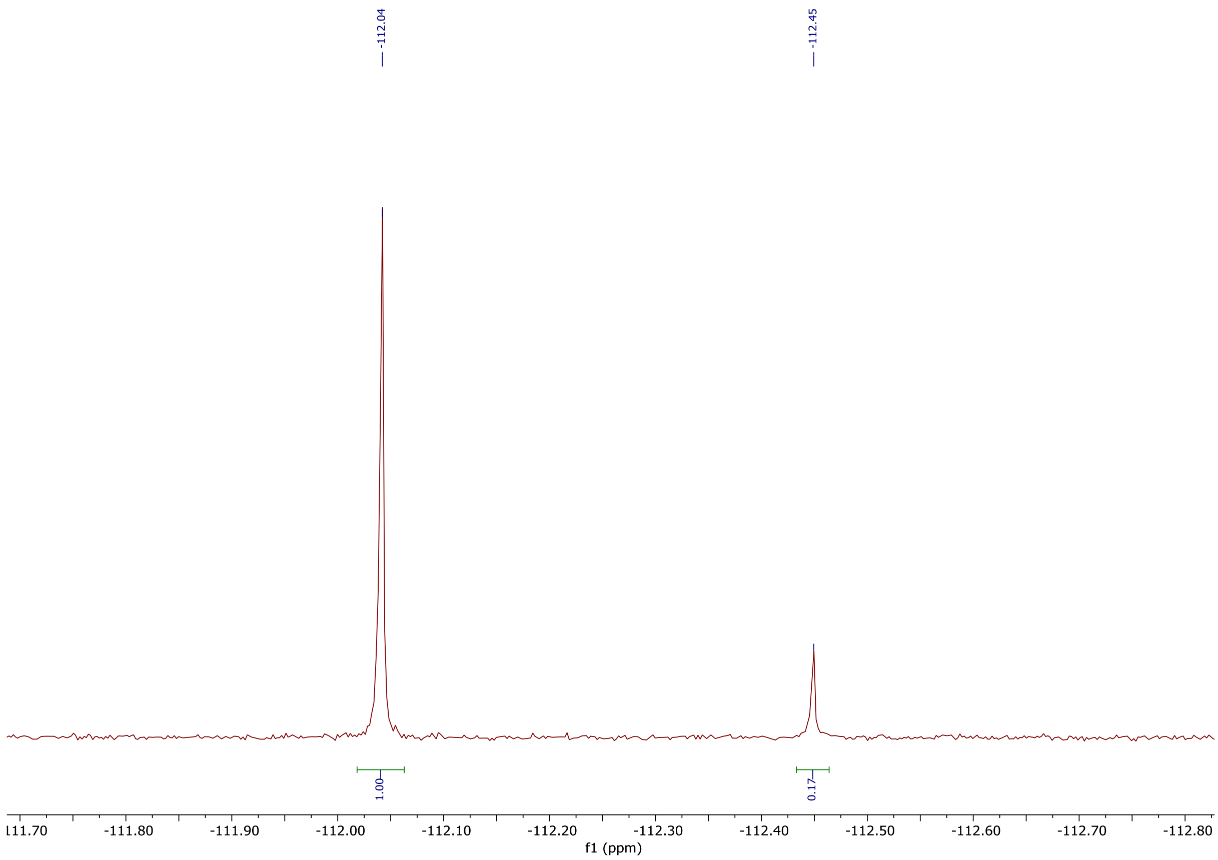

Quantitative ^{19}F NMR spectrum of the crude reaction mixture in $CDCl_3$

(±)-4d

^{1}H NMR (500 MHz, $CDCl_3$) δ_H: 7.95 (2H, d, *J* 8.0, **3-H**), 7.50 (1H, t, *J* 7.3, **1-H**), 7.40 (2H, t, *J* 7.7, **2-H**), 7.38 – 7.24 (1H, m, **10-H**), 7.07 (1H, d, 7.7, **9-H**), 7.01 (1H, dt, *J* 9.8, 2.1, **13-H**), 6.90 (1H, td, *J* 8.4, 2.6, **11-H**), 4.69 (1H, q, *J* 6.9, **6-H**), 1.54 (3H, d, *J* 6.8, **7-H**).

^{13}C{^{1}H} NMR (126 MHz, $CDCl_3$) δ_C: 199.9 (**C5**), 163.2 (d, *J* 246.6, **C12**), 144.0 (d, *J* 7.3, **C8**), 136.4 (**C4**), 133.2 (**C1**), 130.6 (d, *J* 8.4, **C10**), 128.9 (**C3**), 128.7 (**C2**), 123.6 (d, *J* 2.9, **C9**), 114.9 (d, *J* 21.8, **C13**), 114.0 (d, *J* 21.1, **C11**), 47.6 (**C6**), 9.5 (**C7**).

^{19}F NMR (471 MHz, $CDCl_3$) δ_F: –112.3 (q, *J* 8.5, **F**).

(±)-5d

^{1}H NMR (500 MHz, $CDCl_3$) δ_H: 7.72 (1H, d, *J* 7.7, **4-H**), 7.62 (1H, dt, *J* 9.6, 2.3, **2-H**), 7.38 – 7.24 (5H, m, **5-H, 11-H, 12-H**), 7.22 (1H, t, *J* 7.1, **13-H**), 7.17 (1H, td, *J* 8.3, 2.7, **6-H**), 4.62 (1H, q, *J* 6.8, **8-H**), 1.54 (3H, d, *J* 6.8. **9-H**).

$^{13}C\{^1H\}$ NMR (126 MHz, $CDCl_3$) δ_C: 199.2 (d, *J* 2.1, **C7**), 162.9 (d, *J* 247.6, **C1**), 141.2 (**C10**), 138.7 (d, *J* 6.1, **C5**), 130.2 (d, *J* 7.6, **C3**), 129.2 (**C11**), 127.9 (**C12**), 127.2 (**C13**), 124.6 (d, *J* 3.1, **C4**), 119.9 (d, *J* 21.6, **C6**), 115.6 (d, *J* 22.4, **C2**), 48.4 (**C8**), 19.6 (**C9**).

^{19}F NMR (471 MHz, $CDCl_3$) δ_F: −111.9 – −112.0 (m, **F**).

(±)-1-Phenyl-2-(4-(trifluoromethyl)phenyl)propan-1-one (**(±)-4e**) and (±)-2-Phenyl-1-(4-(trifluoromethyl)phenyl)propan-1-one (**(±)-5e**)

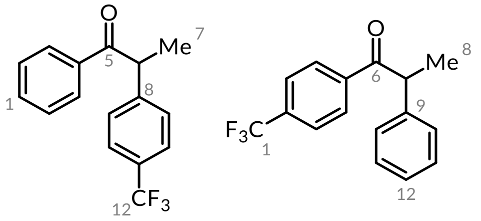

(±)-4e **(±)-5e**

Following general procedure H, 1-phenyl-1-(4-(trifluoromethyl)phenyl)prop-2-en-1-ol (**3e**) (69 mg, 0.23 mmol), cobalt catalyst **Co(II)-6** (6.5 mg, 0.01 mmol), 1-fluoro-2,4,6-trimethylpyridinium tetrafluoroborate (102 mg, 0.45 mmol), 1,1,3,3-tetramethyldisiloxane (160 µL, 0.90 mmol), PhCl (1.9 mL) gave a 0.04 : 1.00 mixture of α-aryl ketones **(±)-4e** and **(±)-5e** as determined by ^{1}H NMR spectroscopy of the crude reaction mixture. After purification by column chromatography (hexane : CH_2Cl_2, 60 : 40), α-aryl ketones **(±)-4e** and **(±)-5e** were isolated as a 0.17 : 1.00 mixture as a yellow oil (56 mg, 0.20 mmol, 88%). NMR spectroscopic data were in accordance with the literature.[27–29]

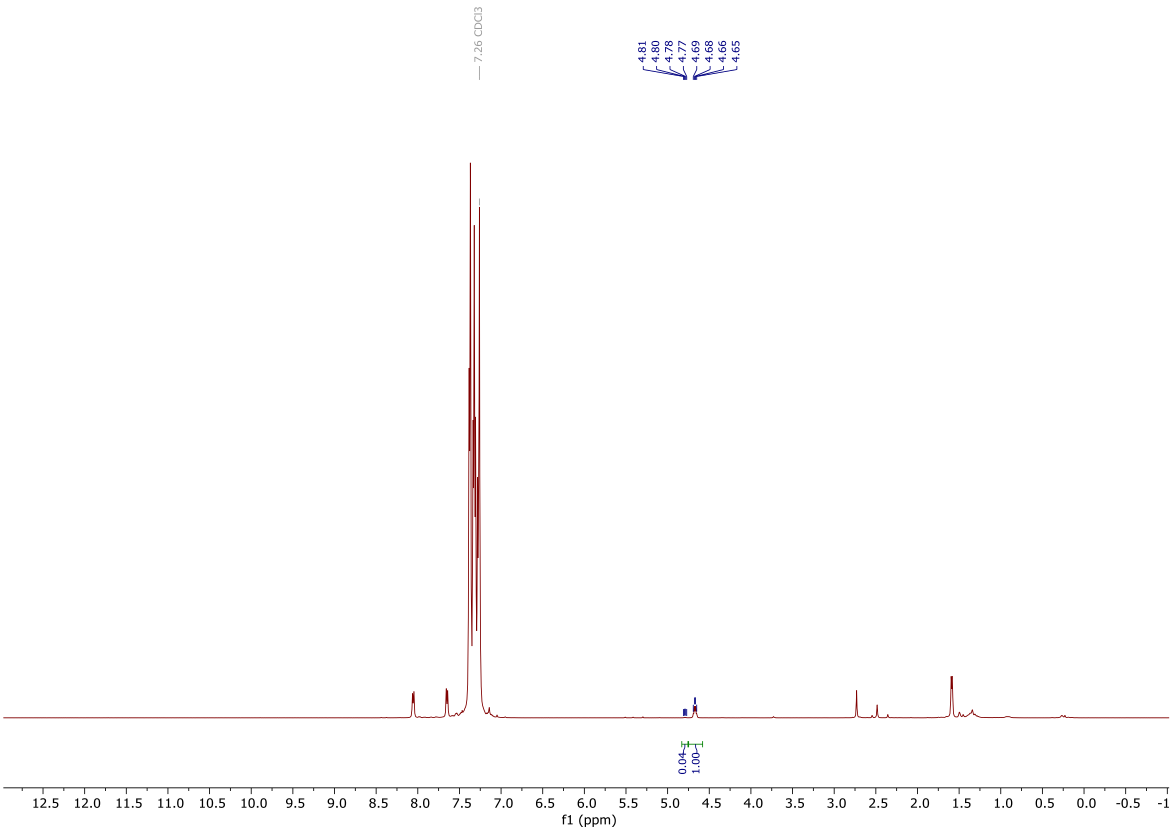

^{1}H NMR spectrum of the crude reaction mixture in $CDCl_3$

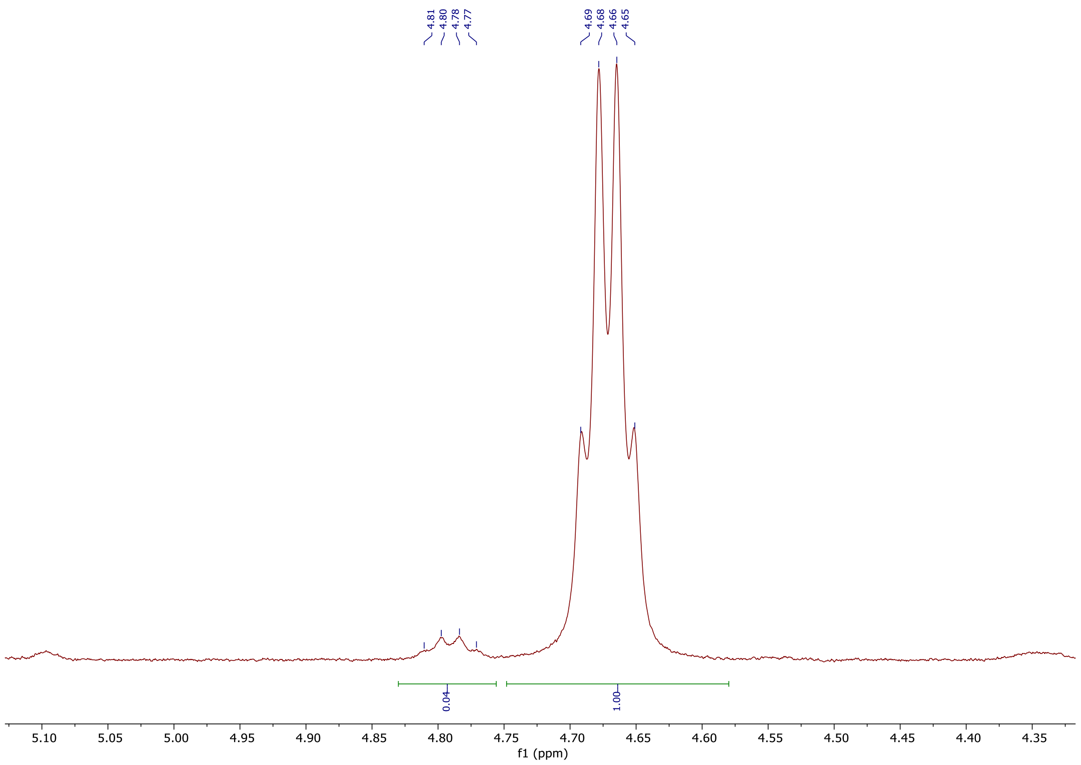

^{1}H NMR spectrum of the crude reaction mixture in $CDCl_3$

(±)-4e:

^{1}H NMR (500 MHz, $CDCl_3$) δ_H: distinguishable/diagnostic peaks only: 7.94 (2H, d, *J* 7.3, **3-H**), 7.56 (2H, d, *J* 8.1, **10-H**), 7.51 (1H, t, *J* 7.4, **1-H**), 7.44 – 7.38 (4H, m, **2-H, 9-H**), 4.77 (1H, q, *J* 6.9, **C6**).

^{13}C{^{1}H} NMR (126 MHz, $CDCl_3$) δ_C: distinguishable/diagnostic peaks only: 199.8 (**C5**), 136.3 (**C4**), 133.3 (**C1**), 128.9 (**C3**), 128.8 (**C2**), 128.3 (**C9**), 126.1 (q, *J* 3.2, **C10**), 47.7 (**C6**), 19.6 (**C7**).

^{19}F NMR (376 MHz, $CDCl_3$) δ_F: –62.5 (CF_3).

(±)-5e:

^{1}H NMR (500 MHz, $CDCl_3$) δ_H: distinguishable/diagnostic peaks only: 8.03 (2H, d, *J* 8.1, **4-H**), 7.63 (2H, d, *J* 8.1, **3-H**), 7.31 (2H, t, *J* 7.5, **11-H**), 4.65 (1H, q, *J* 6.8, **7-H**).

^{13}C{^{1}H} NMR (126 MHz, $CDCl_3$) δ_C: 199.4 (**C6**), 140.9 (**C9**), 139.3 (**C5**), 134.1 (q, *J* 32.5, **C2**), 129.4 (**C11**), 129.2 (**C4**), 127.9 (**C10**), 127.4 (**C12**), 125.7 (q, *J* 3.7, **C3**), 123.7 (q, *J* 272.6, **C1**), 48.7 (**C7**), 19.5 (**C8**).

^{19}F NMR (376 MHz, $CDCl_3$) δ_F: –63.2 (CF_3).

(±)-1-(3-Fluoro-4-(trifluoromethyl)phenyl)-2-phenylpropan-1-one (**(±)-5f**)

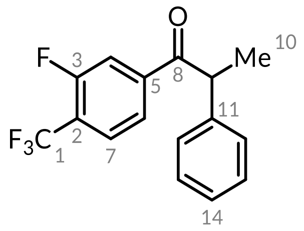

Following general procedure H, 1-(3-fluoro-4-(trifluoromethyl)phenyl)-1-phenylprop-2-en-1-ol (**3f**) (53 mg, 0.18 mmol), cobalt catalyst **Co(II)-6** (5.2 mg, 0.01 mmol), 1-fluoro-2,4,6-trimethylpyridinium tetrafluoroborate (82 mg, 0.36 mmol), 1,1,3,3-tetramethyldisiloxane (127 μL, 0.76 mmol), PhCl (1.5 mL) gave, after purification by column chromatography (hexane : CH_2Cl_2, 60 : 40), α-aryl ketone **(±)-5f** as a yellow oil (47 mg, 0.16 mmol, 88%).

ν_{max} (film): 2928, 2851, 1692 (C=O), 1558, 1539, 1317, 1246, 1134, 1045, 876, 795.

^{1}H NMR (500 MHz, $CDCl_3$) δ_H: 7.76 (1H, d, *J* 8.1, **6-H**), 7.73 (1H, d, *J* 10.9, **4-H**), 7.62 (1H, t, *J* 7.5, **7-H**), 7.32 (2H, t, *J* 7.6, **13-H**), 7.26 – 7.21 (3H, m, **12-H, 14-H**), 4.59 (1H, q, *J* 6.8, **9-H**), 1.55 (3H, d, *J* 6.8, **10-H**).

^{13}C{^{1}H} NMR (126 MHz, $CDCl_3$) δ_C: 198.1 (**C8**), 159.9 (d, *J* 258.0, **C3**), 141.7 (d, *J* 6.4, **C5**), 140.5 (**C11**), 129.5 (**C13**), 127.8 (**C12**), 127.7 (q, *J* 4.8, **C7**), 127.6 (**C14**), 124.3 (d, *J* 4.0, **C6**), 122.2 (q, *J* 272.6, **C1**), 122.0 (qd, *J* 33.1, 12.6, **C2**), 117.1 (d, *J* 21.8 **C4**), 48.9 (**C9**), 19.4 (**C10**).

^{19}F NMR (471 MHz, $CDCl_3$) δ_F: –61.9 (d, *J* 12.5, **CF_3**), –112.7 – –112.9 (m, **F**).

HRMS: (ESI^-) $C_{16}H_{11}F_4O$ $[M–H]^-$ found 295.0753, requires 295.0752 (–0.3 ppm).

Hammett Plot

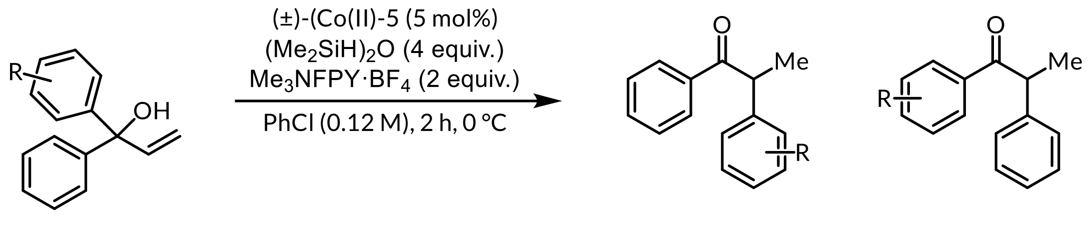

Following general procedure H, racemic allylic alcohol (±)-3 was subjected to the reaction conditions using **(±)-Co(II)-5** catalyst. The reaction mixture was analysed by quantitative NMR spectrometry using dibromomethane (1 equiv.) and fluorobenzene (1 equiv.) as internal standard.

R substituent	Sigma value	Ratio of Products (±)-4 : (±)-5	$\log_{10}(k_X/k_H)$
4-Me	-0.17	2.45 : 1	0.389
H	0	1 : 1	0.000
4-F	0.06	0.78 : 1	-0.108
4-Cl	0.23	0.66 : 1	-0.180
3-F	0.34	0.28 : 1	-0.553
4-CF_3	0.54	0.85 : 1	-0.071

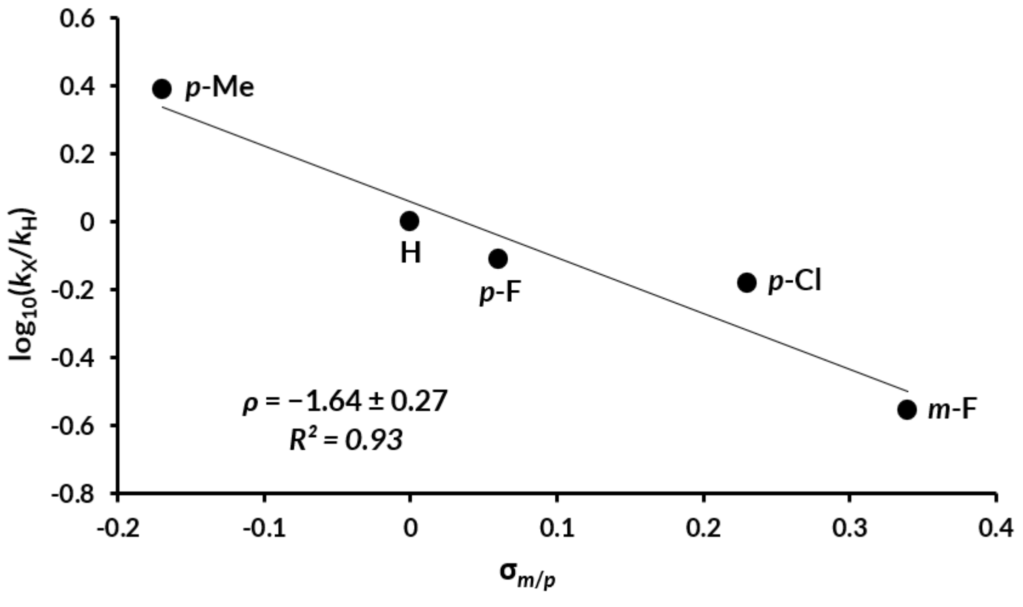

Hammett plot obtained from the ratio of products using catalyst **(±)-Co(II)-5**

Substrate (±)-**3e**, containing a CF_3 group, showed an unexpected deviation from the Hammett plot's linear correlation. We attribute this behaviour to some of product (±)-**4e** forming via a radical migration pathway rather than a polar migration pathway as electron-deficient arenes migrate more readily via a radical mechanism.

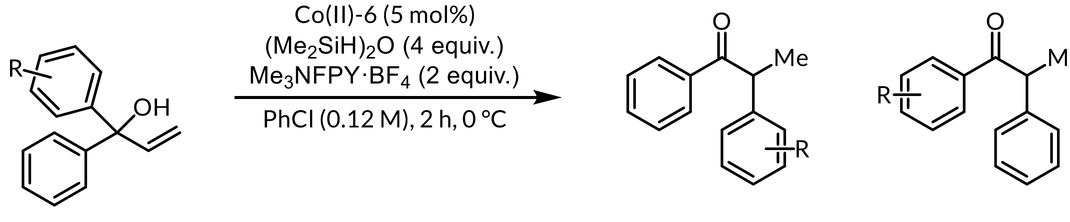

R substituent	Sigma value	Ratio of Products (±)-4 : (±)-5	$\log_{10}(k_X/k_H)$
4-Me	-0.17	3.1 : 1	0.491
H	0	1 : 1	0.000
4-F	0.06	0.82 : 1	-0.086
4-Cl	0.23	0.41 : 1	-0.387
3-F	0.34	0.17 : 1	-0.770
4-CF_3	0.54	0.04 : 1	-1.398

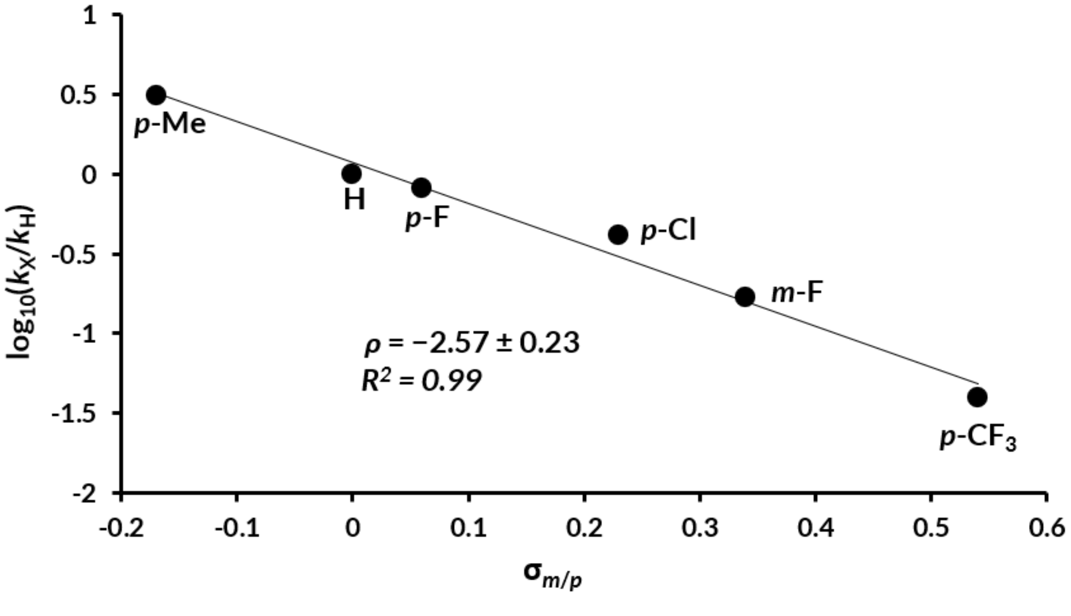

Hammett plot obtained from the ratio of products using catalyst **Co(II)-6**

We observe a more negative ρ value of −2.57 with the achiral catalyst **Co(II)-6** versus **(±)-Co(II)-5**. The salen ligands bound to these cobalt catalysts are distinct and with catalyst **Co(II)-6** being achiral we cannot distinguish between a migration pathway involving displacement of the cobalt within an alkylcobalt(IV) species or migration involving a free carbocation intermediate. The more negative ρ value implies greater involvement of the free carbocation pathway with catalyst **Co(II)-6** or that the different salen ligand structures can stabilise positive charge to varying degrees.

Deuterium-Labelling Experiment

Dimethyl(phenyl)silane-*d* (**Si-D$_1$**)

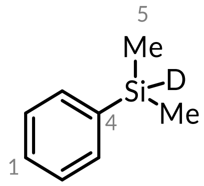

Following a literature procedure by Gellrich and co-workers,[30] chlorodimethyl(phenyl)silane (1.01 mL, 6.00 mmol) in acetonitrile (extra dry, 1 mL) was added dropwise to $NaBD_4$ in acetonitrile (extra dry, 4 mL) and stirred for 15 min at RT. The reaction mixture was cooled to 0 °C, solvent was removed at 25 mbar and the crude product was filtered and washed on a glass frit with hexane (ca. 5 mL). Solvent was removed at 0 °C and 25 mbar, the product was filtered over a silica plug with hexane (ca. 5 mL). The solvent was removed at 0 °C and 25 mbar to afford silane **Si-D$_1$** as a colourless oil (373 mg, 2.71 mmol, 45%, 98% D). NMR spectroscopic data were in accordance with the literature.[31]

ν_{max} (film): 3069, 2959, 1539, 1427, 1248, 1115, 822, 696.

^{1}H NMR (400 MHz, CDCl$_3$) δ_H: 7.61 – 7.54 (2H, m **3-H**), 7.42 – 7.35 (3H, m, **1-H**, **2-H**), 0.39 – 0.34 (6H, m **5-H**).

^{13}C{^{1}H} NMR (126 MHz, CDCl$_3$) δ_C: 137.6 (**C4**), 134.2 (**C3**), 129.3 (**C1**), 128.0 (**C2**), -3.7 (**C5**).

^{29}Si{^{1}H} NMR (79 MHz, CDCl$_3$) δ_C: –16.9 – –18.0 (m, **Si**).

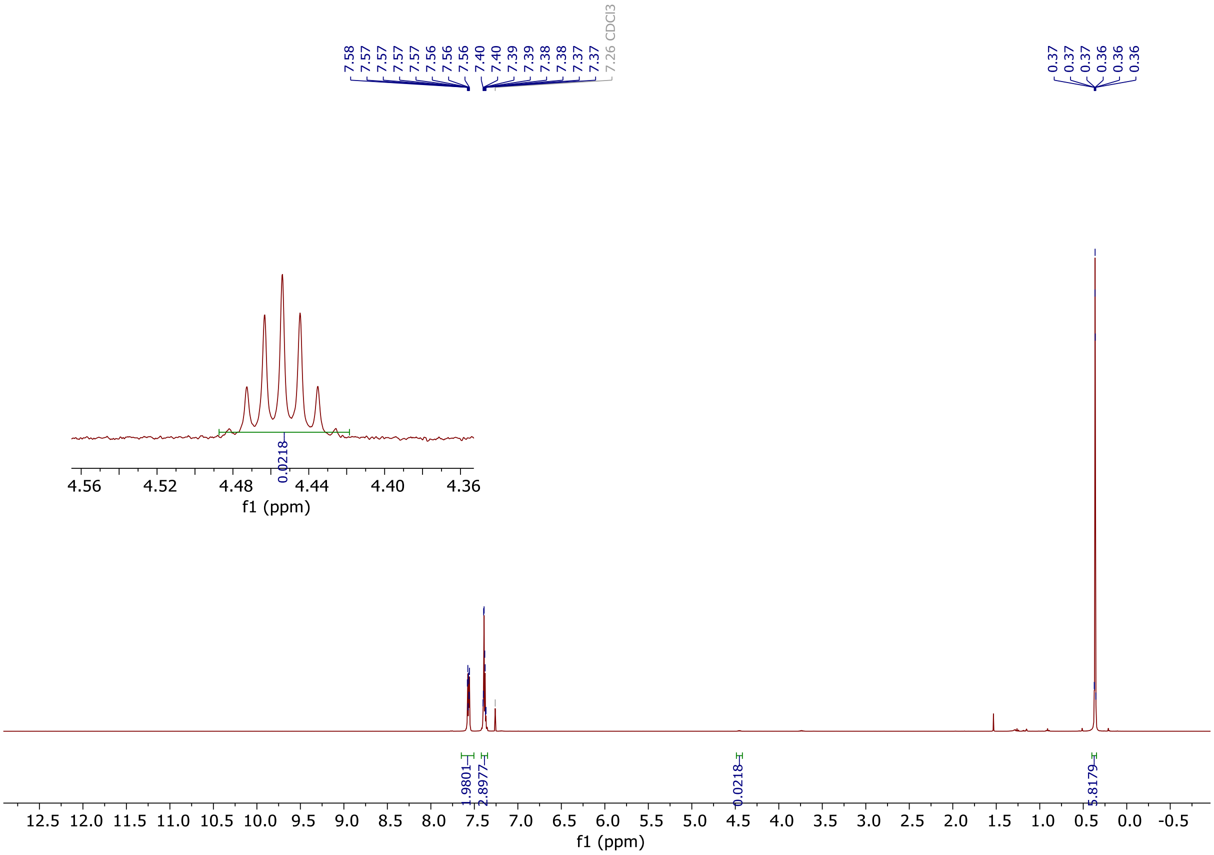

^{1}H NMR spectrum of the deuterated silane **Si-D$_1$** in $CDCl_3$

(*R*)-1,2-Diphenylpropan-1-one-3-*d* (**(*R*)-2b-D$_1$**)

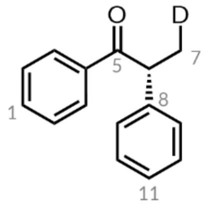

Following general procedure E, 1,1-diphenylprop-2-en-1-ol (**1b**) (21 mg, 0.10 mmol), **(*R*,*R*)-Co(II)-5** (3.9 mg, 0.005 mmol), 1-fluoro-2,4,6-trimethylpyridinium tetrafluoroborate (45 mg, 0.20 mmol), phenyldimethylsilane-d (65 μL, 0.40 mmol), PhCl (0.9 mL) gave, after purification by column chromatography (hexane : ethyl acetate, 98 : 2 to 80 : 20), α-aryl ketone **(*R*)-2b-D$_1$** as a colourless oil (20 mg, 0.096 mmol, 96%).

$[\alpha]_D^{20}$ –30.3, (*c* 0.5 in $CHCl_3$)

Chiral HPLC: Chiralcel OD-H (hexane : IPA, 99 : 1), flow rate = 0.6 mL min^{-1}, λ = 254 nm, 30 °C) t_R(*S*): 11.5 min, t_R(*R*): 14.1 min, 8 : 92 er.

ν_{max} (film): 3061, 2937, 1680 (C=O), 1448, 1213, 756, 696.

^{1}H NMR (500 MHz, $CDCl_3$) δ_H: 7.95 (2H, d, *J* 8.5, **3-H**), 7.47 (1H, t, *J* 7.4, **C1**), 7.38 (2H, t, *J* 7.7, **2-H**), 7.32 – 7.27 (4H, m, **9-H, 10-H**), 7.23 – 7.18 (1H, m, **11-H**), 4.69 (1H, t, *J* 6.8, **6-H**), 1.52 (2H, dt, *J* 6.9, 1.7, **7-H**).

^{13}C{^{1}H} NMR (126 MHz, $CDCl_3$) δ_C 200.5 (**C5**), 141.6 (**C8**), 136.6 (**C4**), 132.9 (**C1**), 129.1 (**C10**), 128.9 (**C3**), 128.6 (**C2**), 127.9 (**C9**), 127.0 (**C11**), 48.0 (**C6**), 19.4 (t, *J* 19.8, **C7**)

HRMS: (ESI$^+$) $C_{15}H_{13}{}^2HONa$ [M+Na]$^+$ found 234.0998, requires 234.1000 (–0.7 ppm)

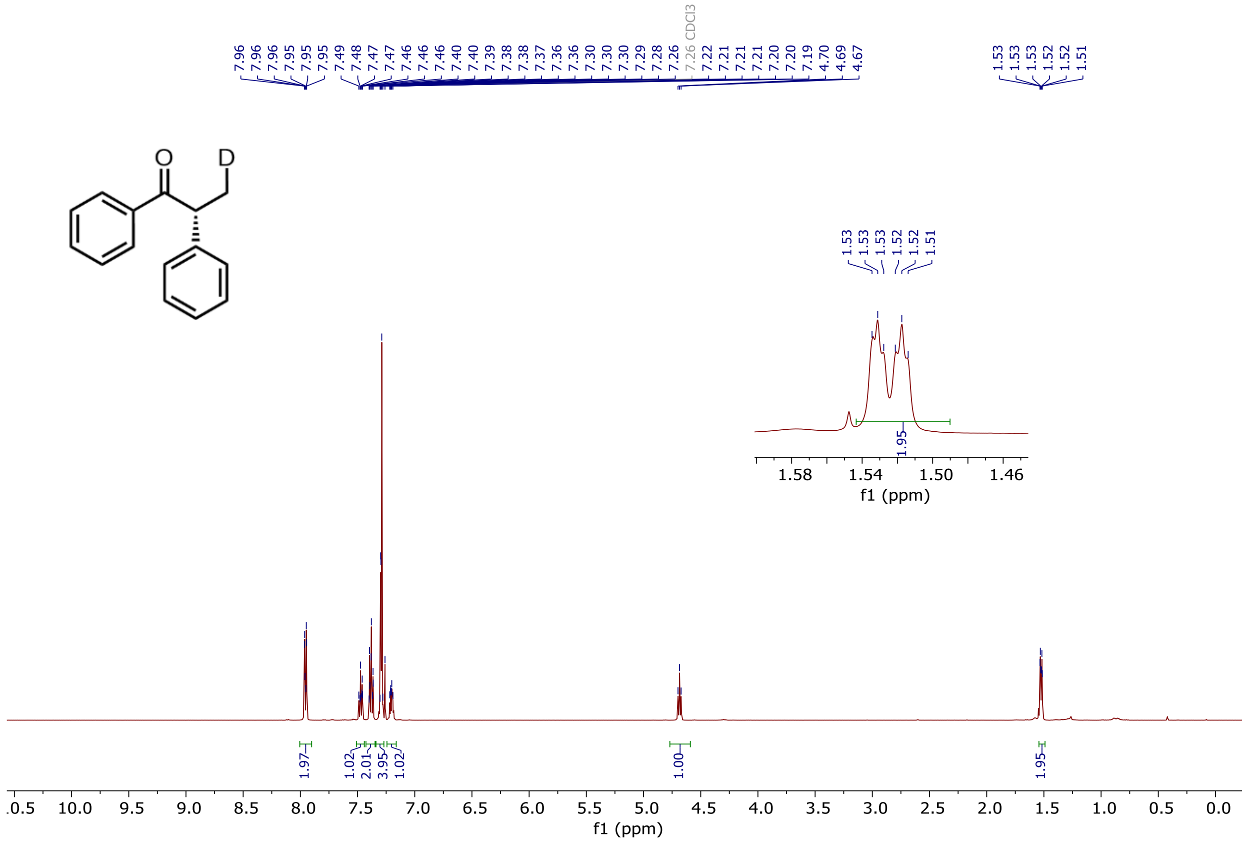

^{1}H NMR spectrum of ketone **(*R*)-2b-D$_1$** in $CDCl_3$

Semipinacol Rearrangement with Co^{III} Salen Somplex

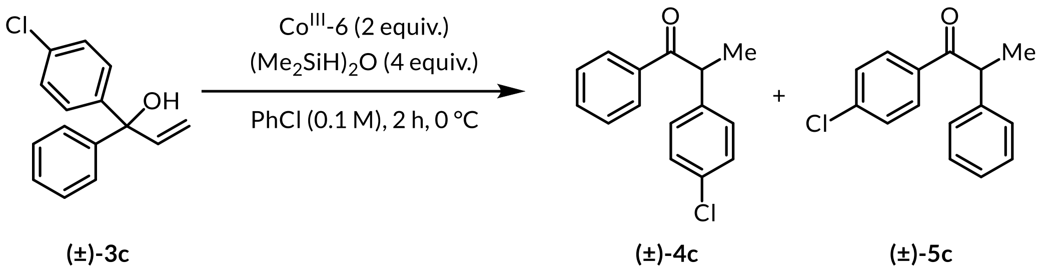

Following general procedure H, 1-(4-chlorophenyl)-1-phenylprop-2-en-1-ol (**(±)-3c**) (24 mg, 0.10 mmol), cobalt catalyst **Co(III)-6** (150 mg, 0.20 mmol), 1,1,3,3-tetramethyldisiloxane (71 μL, 0.40 mmol), PhCl (1.5 mL) gave a 0.41 : 1.00 mixture of α-aryl ketones **(±)-4c** and **(±)-5c** as determined by ^{1}H NMR spectroscopy of the crude reaction mixture. NMR spectroscopic data were in accordance with the literature.[24,25]

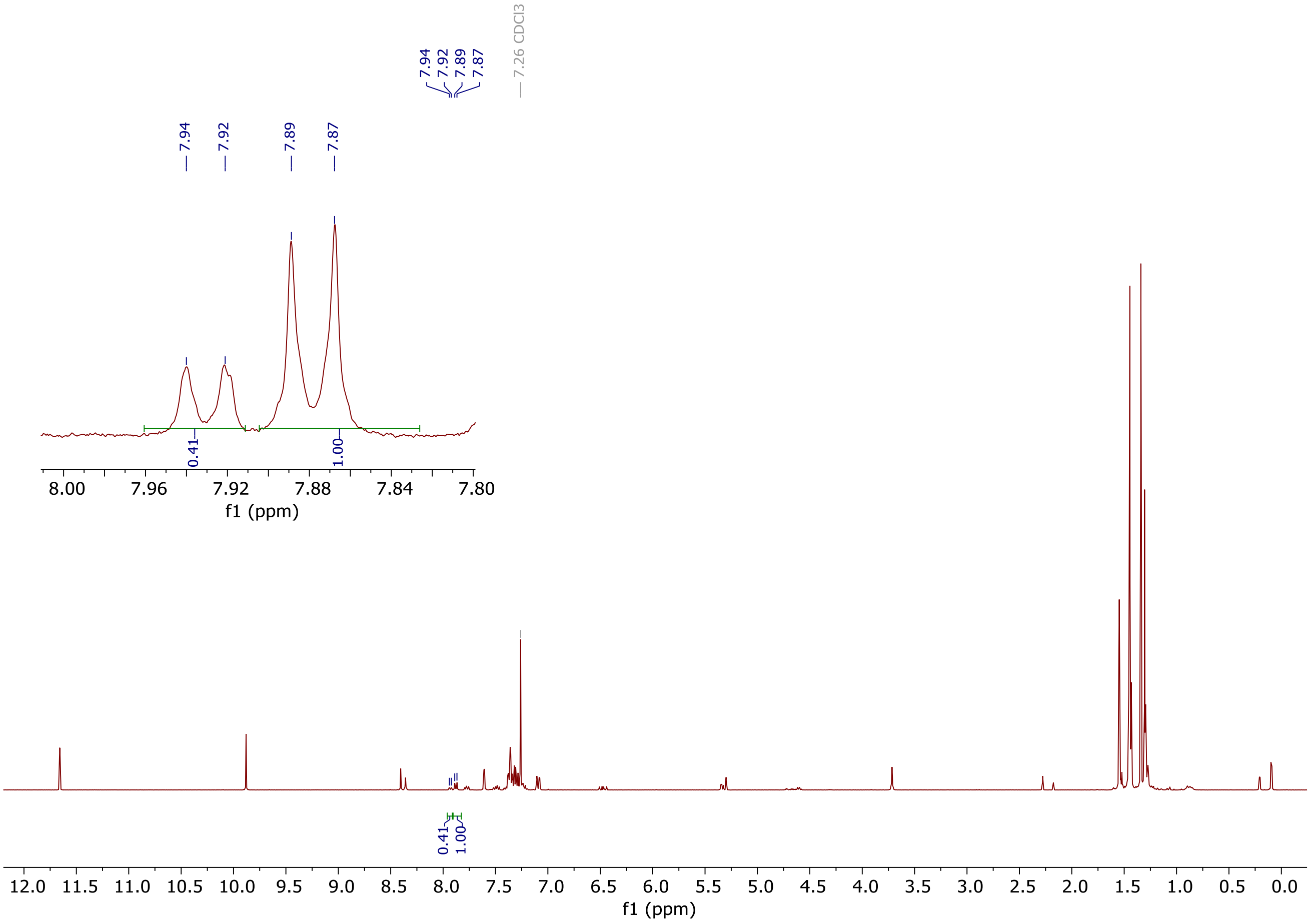

^{1}H NMR spectrum of the crude reaction mixture in $CDCl_3$

Eyring Analysis

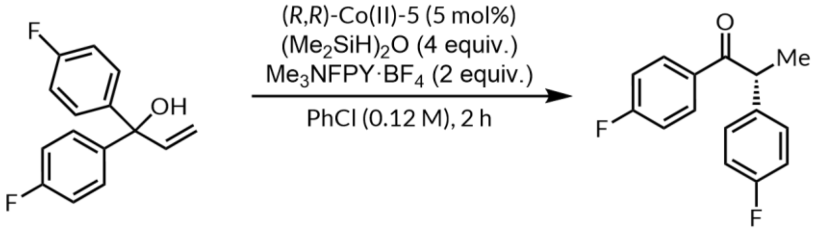

1a 2a

Following general procedure G, 1,1-bis(4-fluorophenyl)prop-2-en-1-ol (**1a**) (25 mg, 0.10 mmol), cobalt catalyst **(*R*,*R*)-Co(II)-5** (3.9 mg, 0.01 mmol), 1-fluoro-2,4,6-trimethylpyridinium tetrafluoroborate (45 mg, 0.20 mmol), 1,1,3,3-tetramethyldisiloxane (71 μL, 0.40 mmol), PhCl (0.9 mL) at the specified temperatures below gave an enantiomeric ratio determined by HPLC analysis as shown below.

Temperature (°C)	0	10	20	30	40
1/T (x10^{-3}) K^{-1}	3.30	3.41	3.53	3.60	3.19
er	98.6: 1.4	98.4 : 1.6	97.2 : 2.8	94.7 : 5.3	89.7 : 10.3

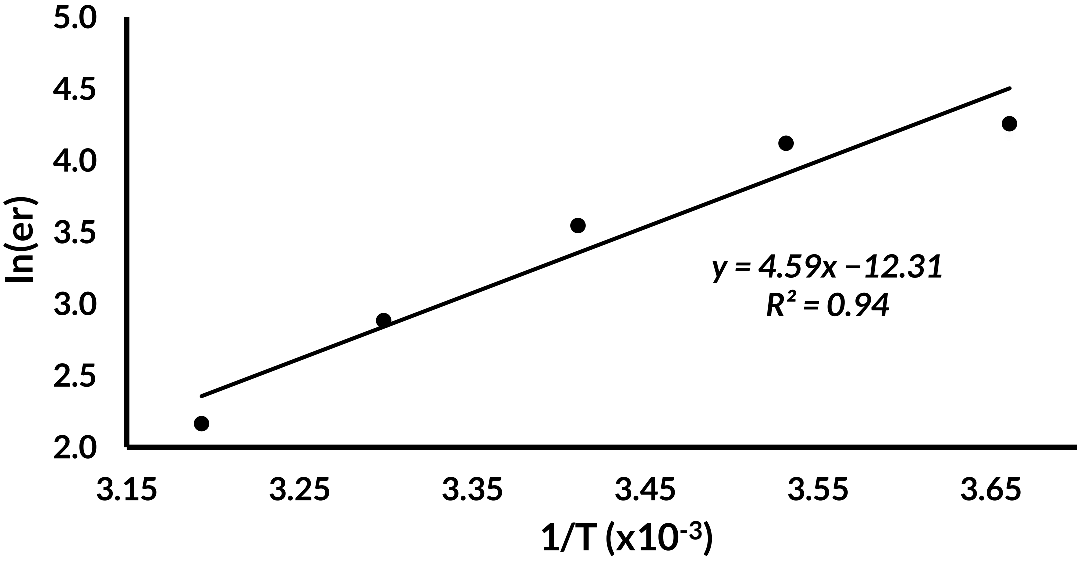

R	1.986	cal mol^{-1} K^{-1}		**error (±)**
	**ΔΔH‡**	-9.12	kcal mol^{-1}	1.32
	**ΔΔS‡**	-24.4	cal mol^{-1} K^{-1}	4.5

Further Discussion on the Enantiodetermining Step

It is challenging to delineate the exact mechanism and role of the chiral cobalt catalyst in controlling the stereochemical outcome of this transformation. Different steps within the catalytic cycle may individually or collectively contribute towards the enantiodiscrimination observed. Given the postulated catalytic cycle for this reaction, the steps that could be considered as enantiodetermining are shown in the figure below. One possibility is that the alkyl radical produced after the HAT step combines stereoselectively with the optically pure cobalt(II) complex to generate an alkylcobalt(III) species (**A**).[32] Alternatively, this alkyl radical could be stereoselectively trapped by a cationic cobalt(III) complex to directly form the key alkylcobalt(IV) intermediate (**B**).[33,34] Instead, the alkylcobalt(III) intermediate could be enriched by a radical chain process with the dominant organocobalt(III) epimer then undergoing single electron oxidation to form the alkylcobalt(IV) species (**C**).[35,36] Another option involves two epimeric alkylcobalt(IV) intermediates undergoing a dynamic kinetic resolution with one rearranging faster to give the major enantiomer of the ketone product (**D**).[37] The nature of the epimerisation process could involve either reversible homolysis or heterolysis of the carbon–cobalt bond,[38] with the former also possibly occurring via a radical chain process.[35]

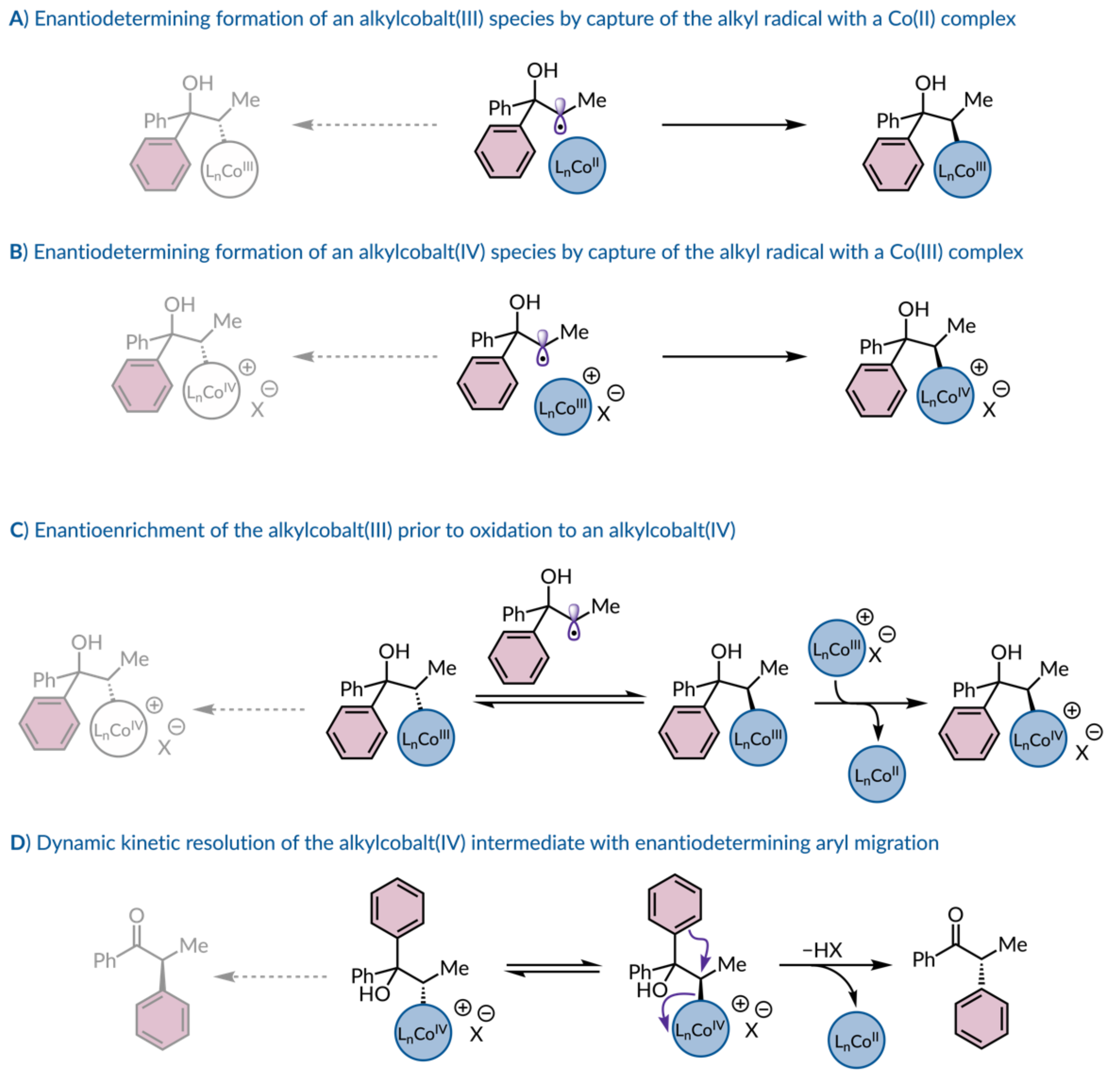

Our preliminary findings at this stage suggest that step **A** is not enantiodetermining given that the differential enthalpy of activation determined by Eyring analysis with **(*R*,*R*)-Co(II)-5** was –9.12 ± 1.32 kcal/mol, which is much larger than the known enthalpy of activation (~2 kcal/mol) for this diffusion-controlled process (see page **S129**).[32,39] We also found that the steric bulk of the silane and the presence of air had minimal impact on the enantioselectivity of the reaction (see page **S9** to **S11**). This does not support step **C** as being enantiodetermining as the concentration of the radical intermediate would be lowered by the presence of oxygen and can be influenced by the steric effects of the silane.[36] Thus, steps **B** or **D** seem more probable as the enantiodetermining steps in this transformation. However, additional investigations are needed to clarify this and to examine these possibilities more thoroughly.

Varying Combination of Catalysts with Substrate

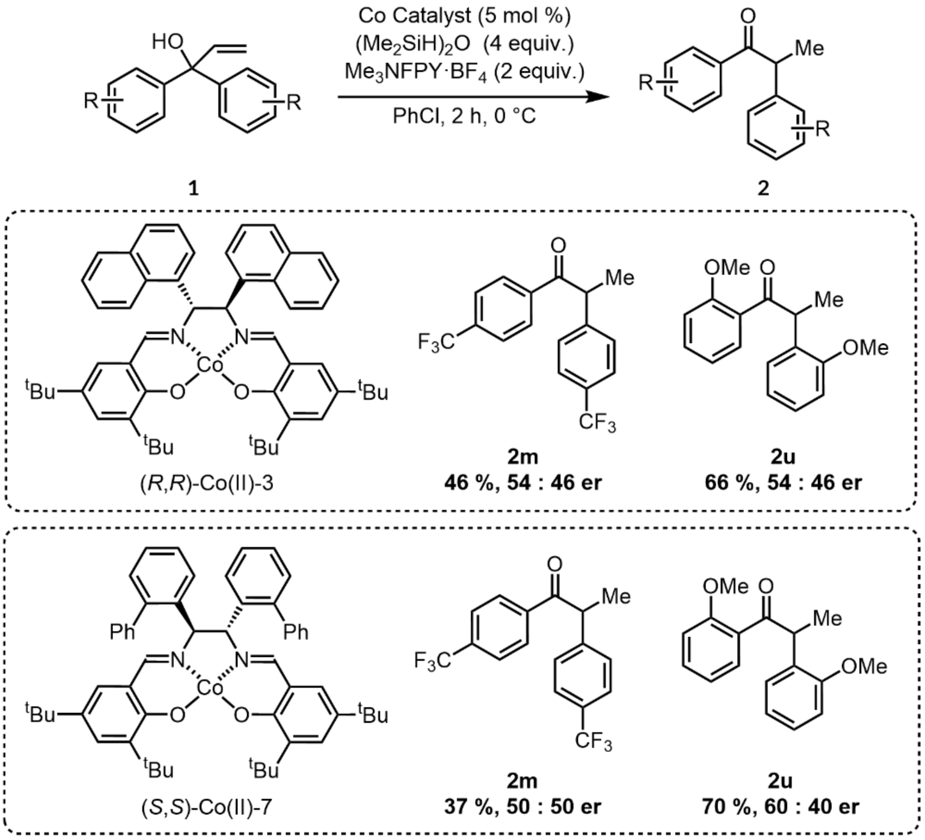

Substrates **1m** and **1u**, which showed a diminished enantioselectivity when using catalyst **(*R*,*R*)-Co(II)-5**, were subjected to catalytic procedure H using catalysts **(*R*,*R*)-Co(II)-3** and **(*S*,*S*)-Co(II)-7**. Analysis of the crude reaction mixture by ^{1}H NMR spectroscopy with dibromomethane (1 equiv.) as internal standard revealed that yield of **2m** was not improved when using either catalyst. Analysis of the crude reaction mixture by ^{1}H NMR spectroscopy with 1,3,5-trimethoxybenzene (1 equiv.) as internal standard revealed that the yield of product **2u** was improved when using either catalyst. HPLC analysis of the products revealed that enantioselectivity could not be improved with catalysts **(*R*,*R*)-Co(II)-3** and **(*S*,*S*)-Co(II)-7**.

NMR Spectra

Bis(4-(trifluoromethyl)phenyl)methanol (**6m**)

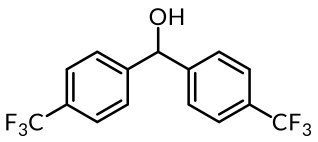

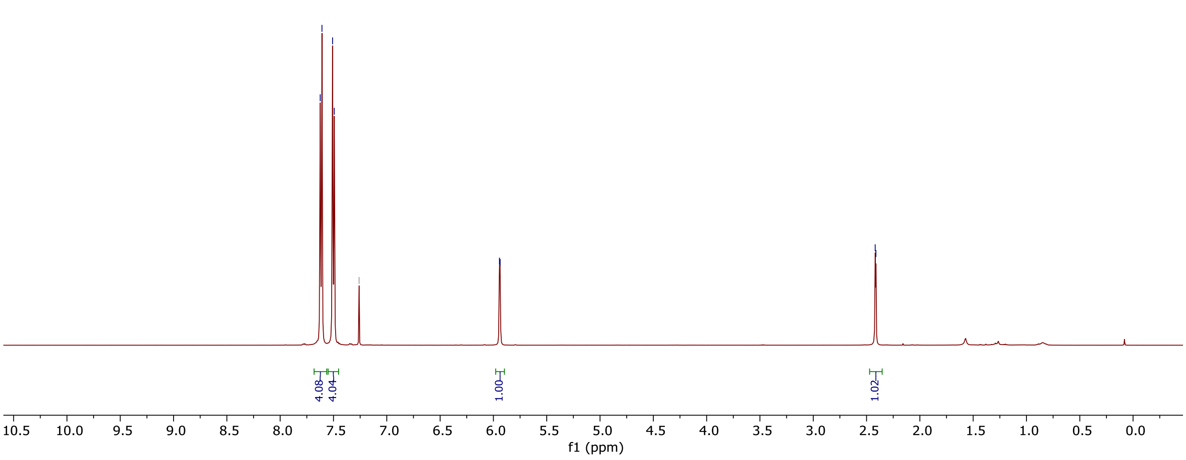

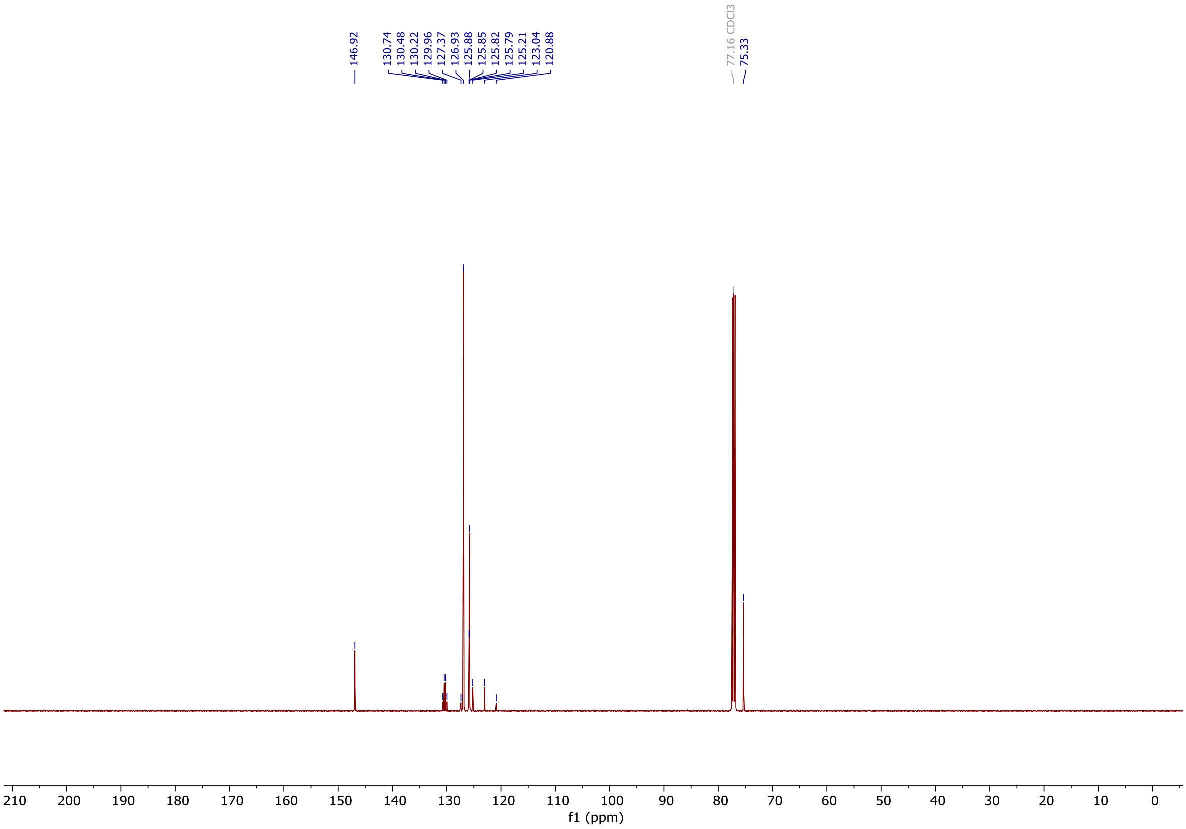

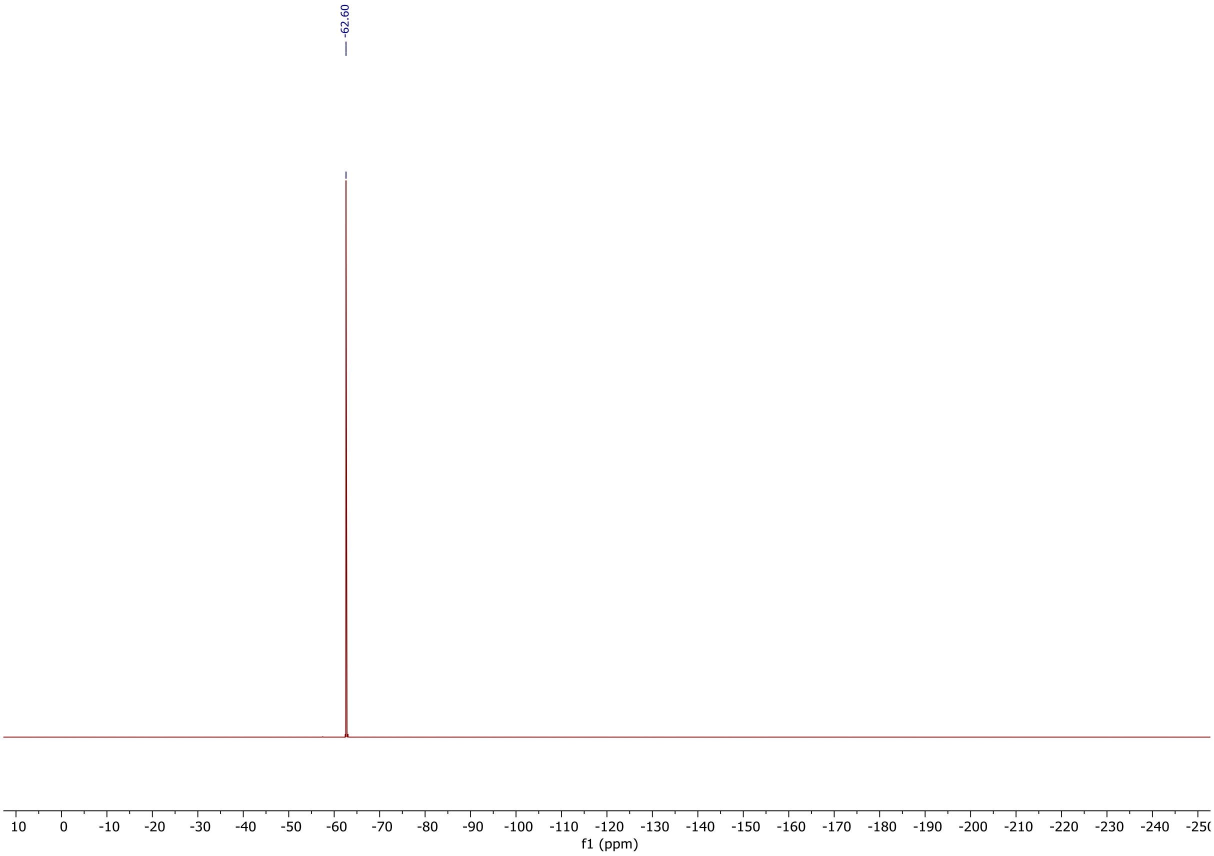
-62.60
10 0 -10 -20 -30 -40 -50 -60 -70 -80 -90 -100 -110 -120 -130 -140 -150 -160 -170 -180 -190 -200 -210 -220 -230 -240
f1 (ppm)

Bis(4-chlorophenyl)methanol (**6o**)

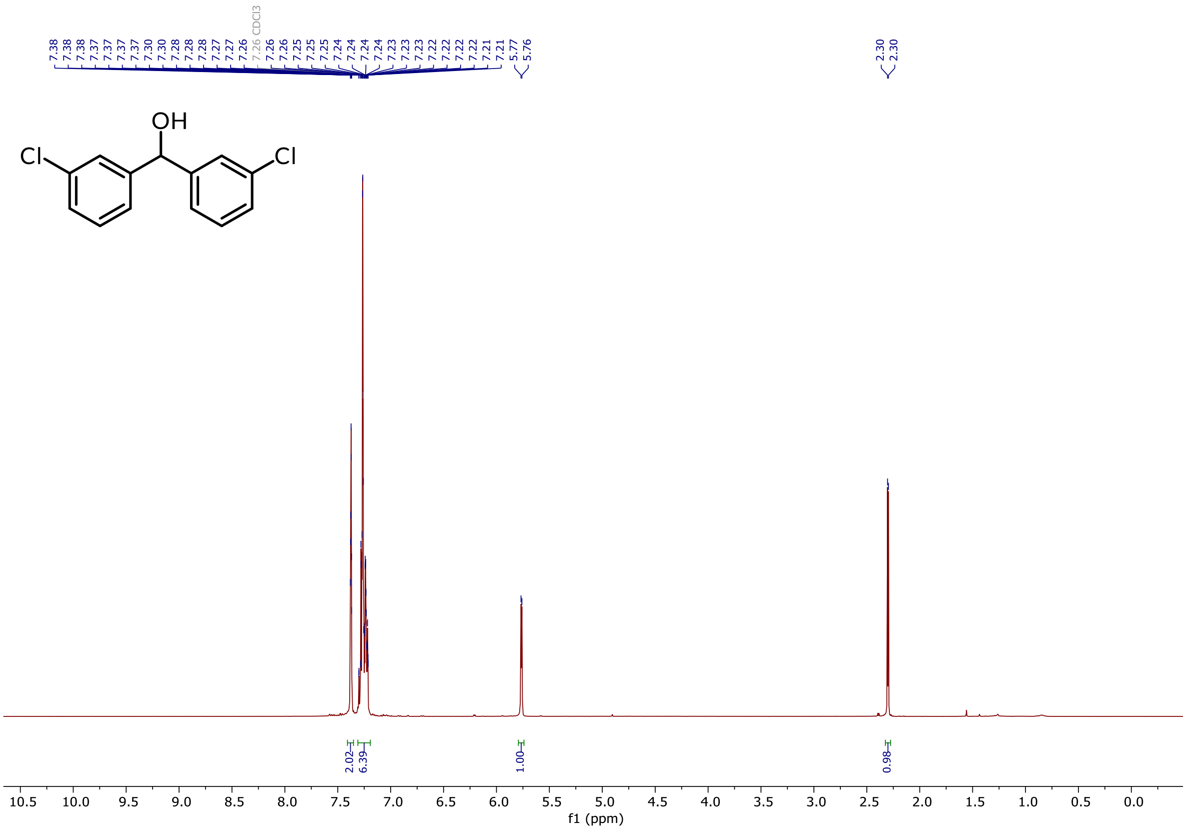

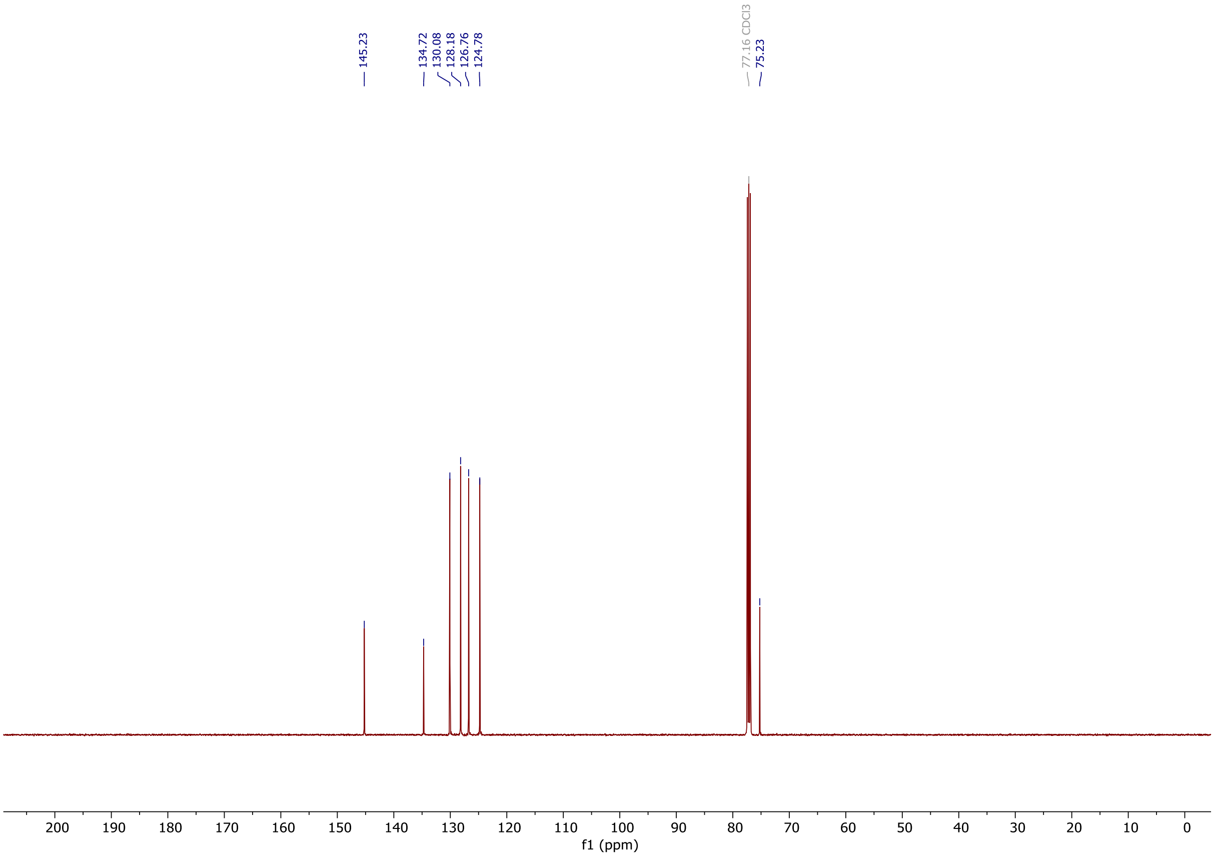

Di-*m*-tolylmethanol (**6q**)

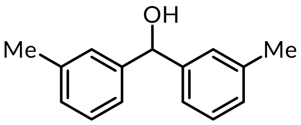

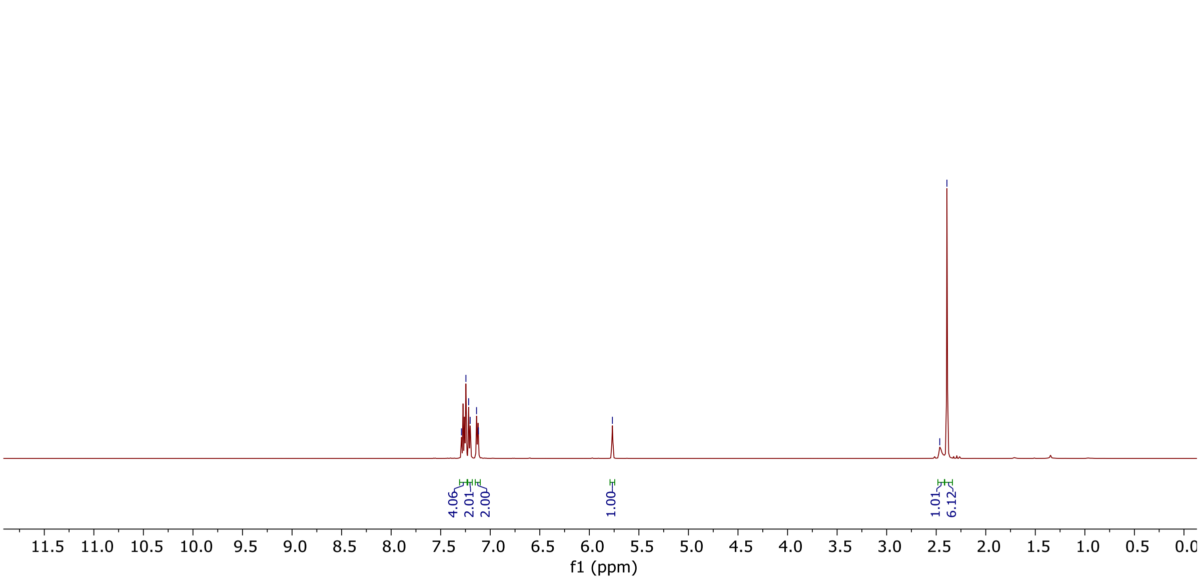

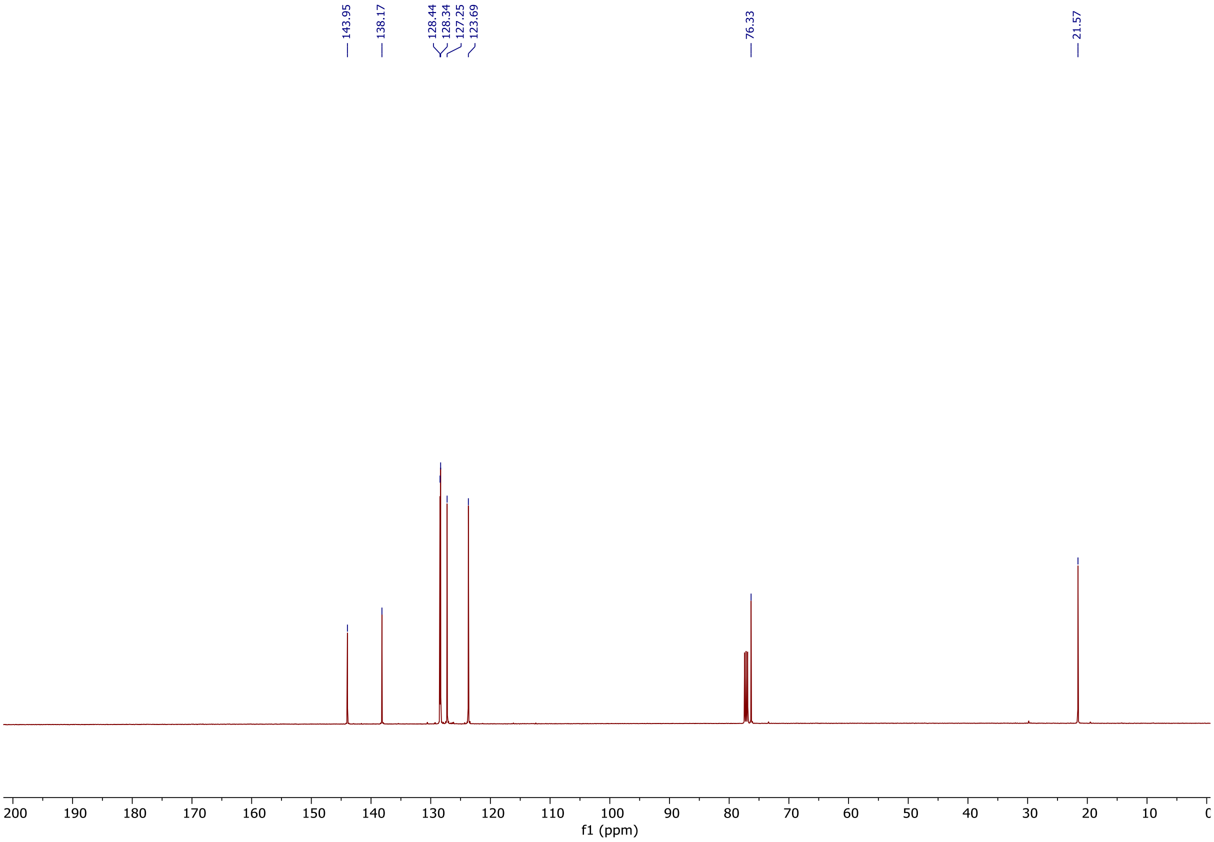

Bis(3-methoxyphenyl)methanol (**6r**)

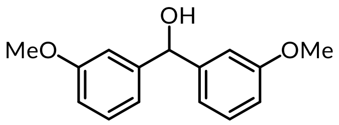

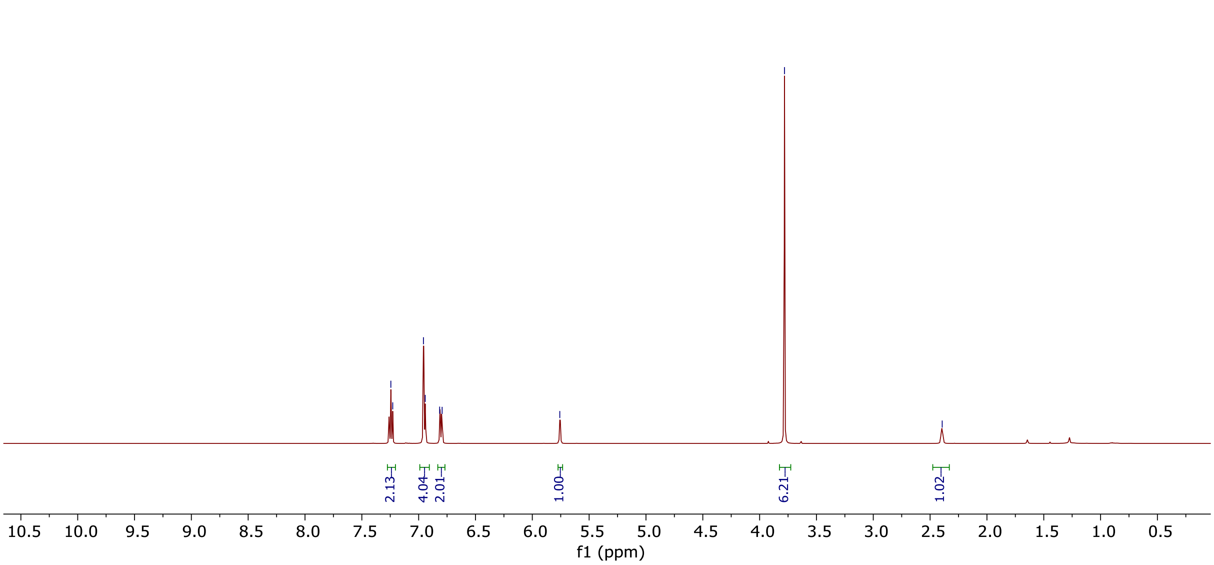

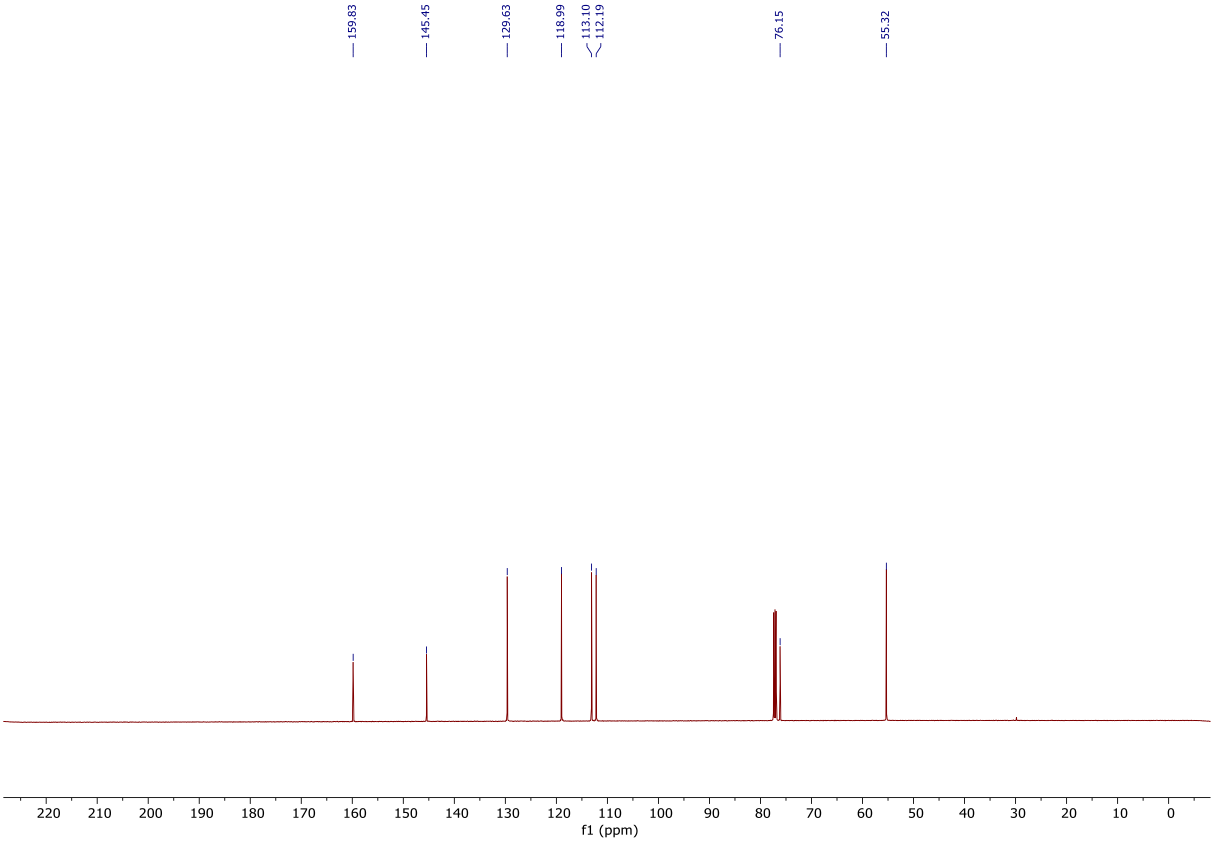

Di-o-tolylmethanol (**6t**)

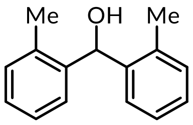

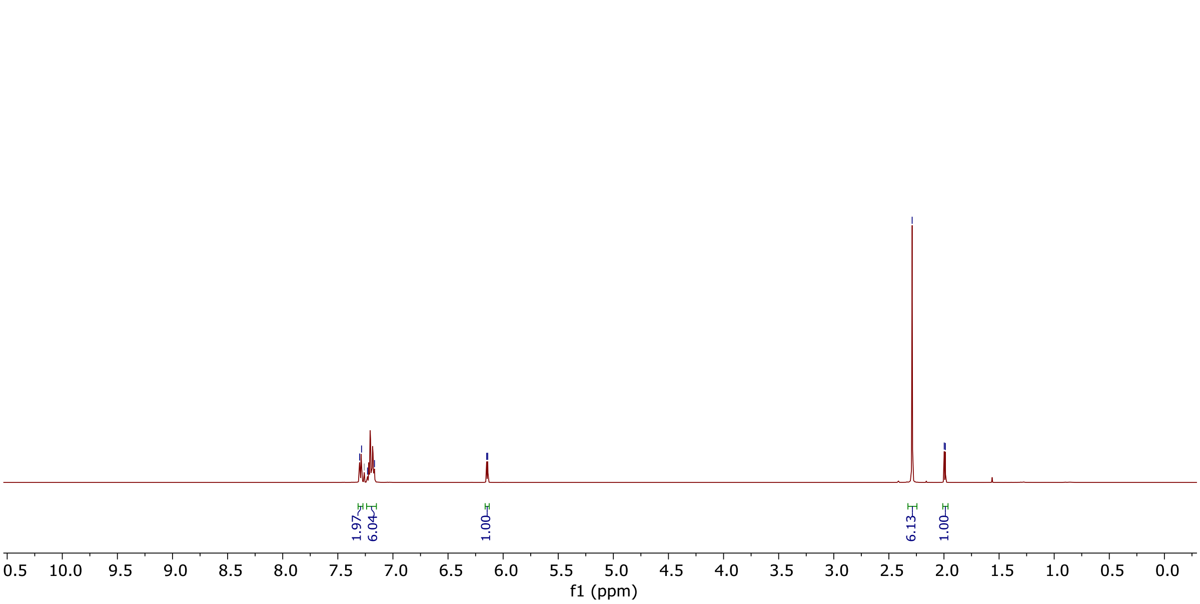

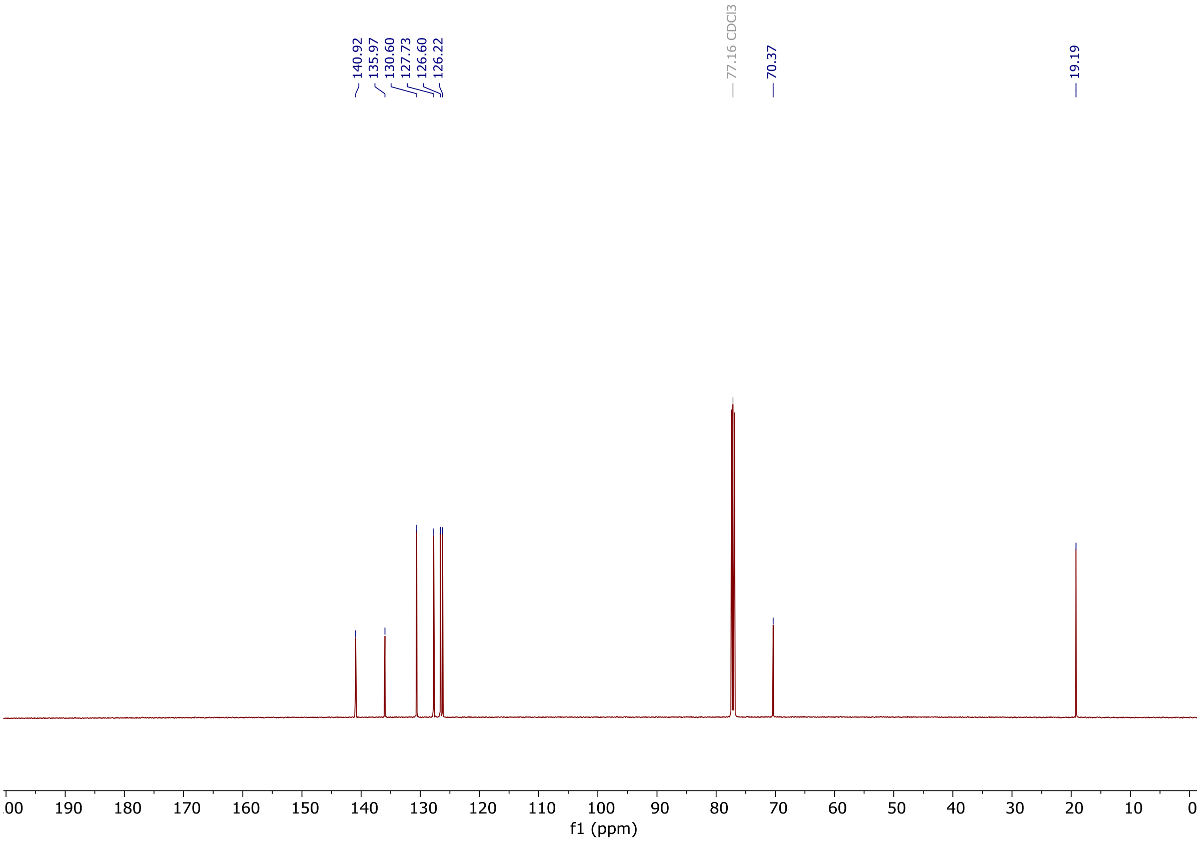

Bis(2-fluorophenyl)methanol (**6v**)

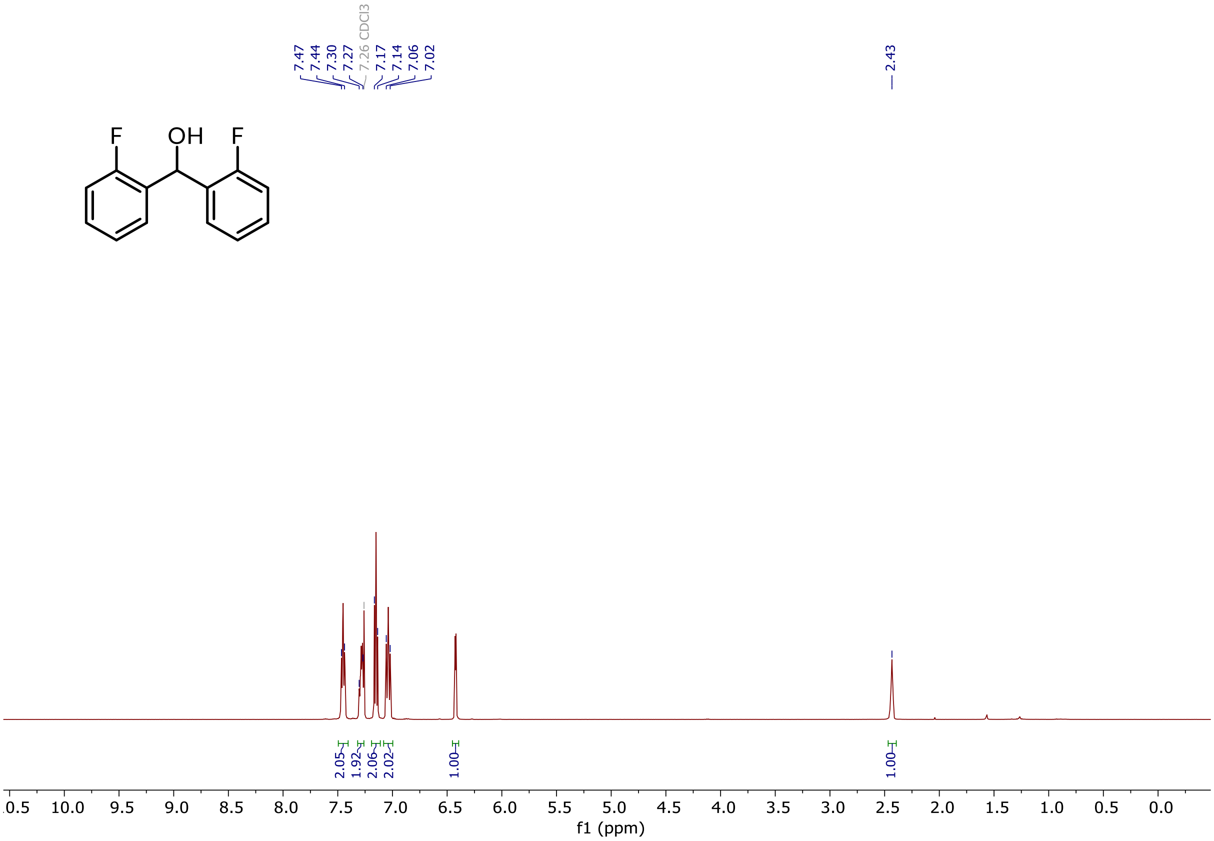

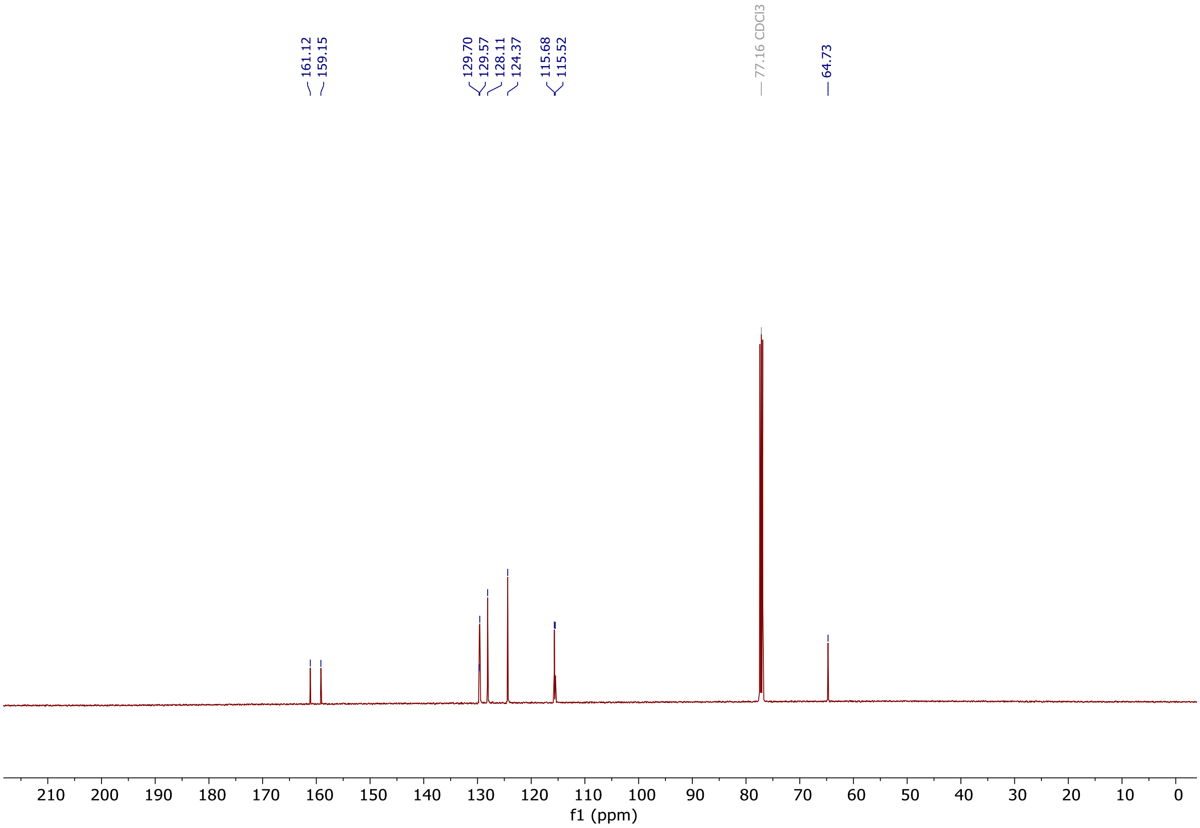

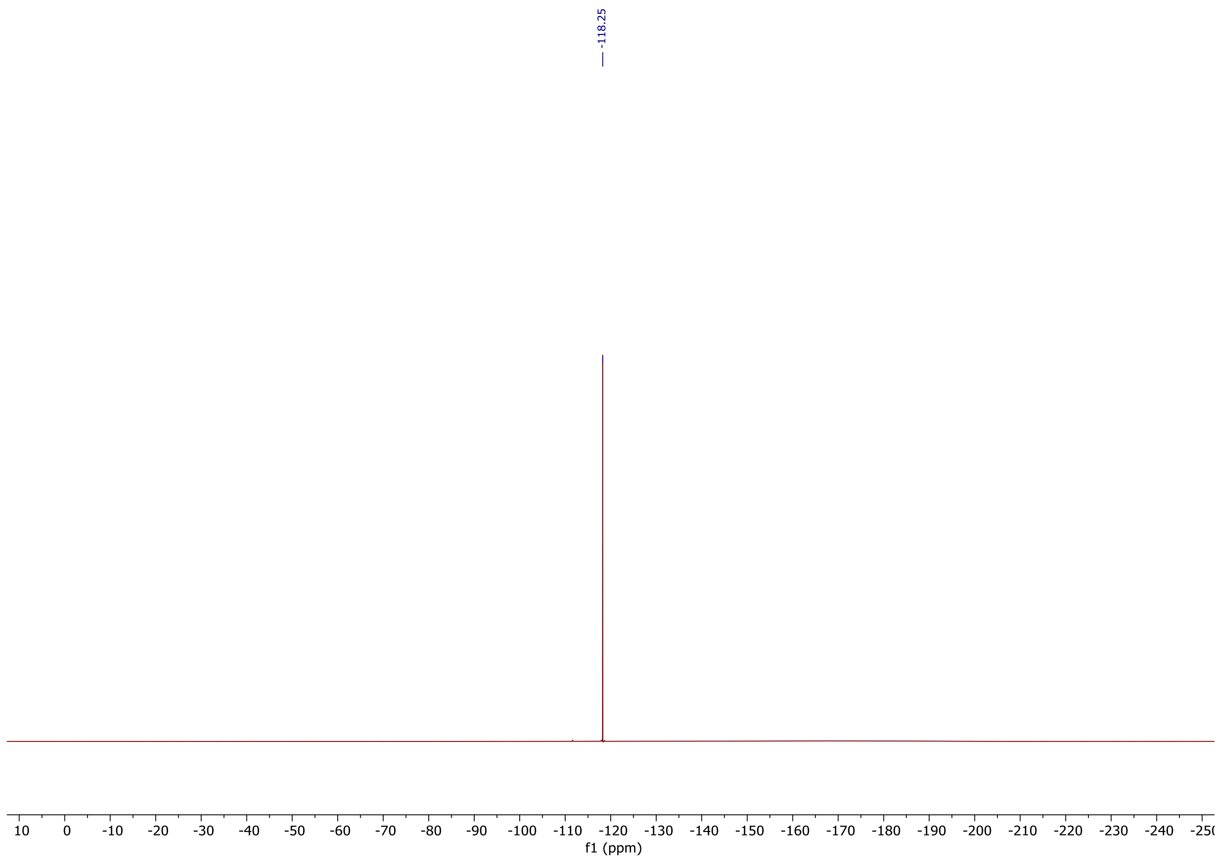

-118.25
10
0
-10
-20
-30
-40
-50
-60
-70
-80
-90
-100
-110
-120
-130
-140
-150
-160
-170
-180
-190
-200
-210
-220
-230
-240
f1 (ppm)

Bis(4-isopropylphenyl)methanone (**7d**)

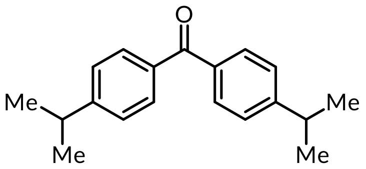

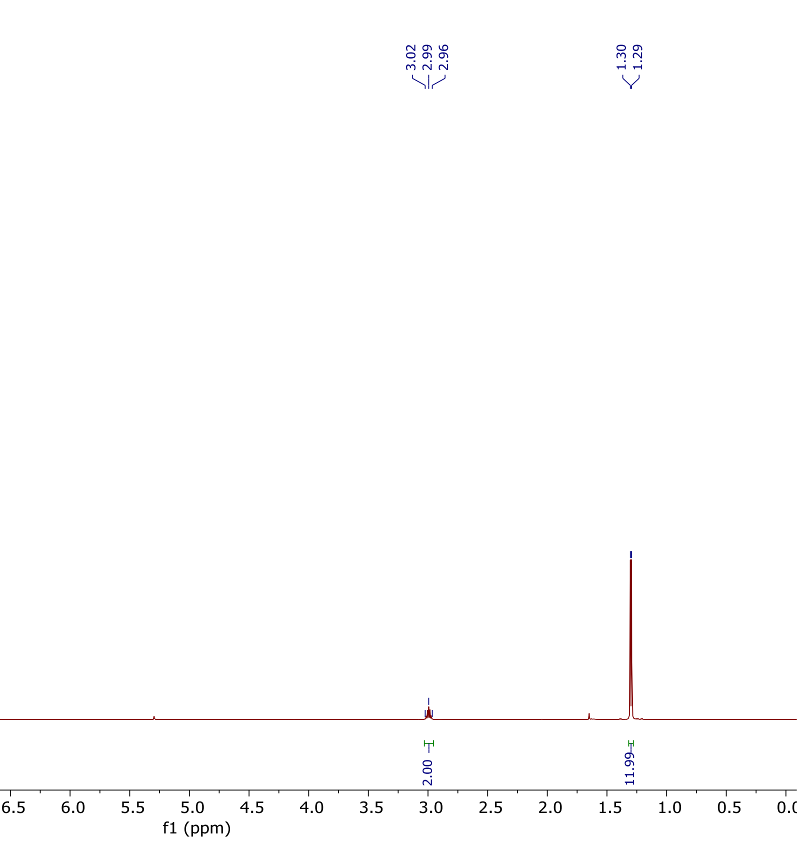

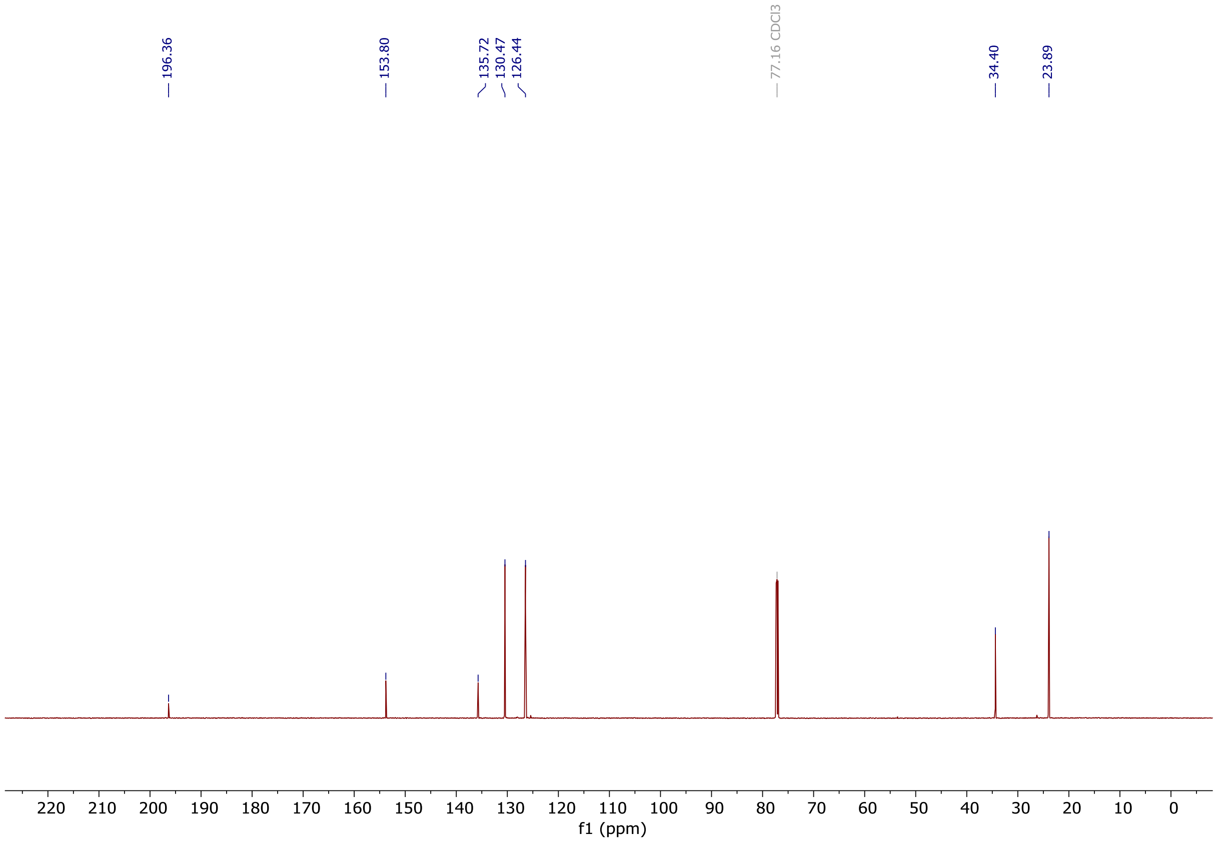

Bis(4-(*tert*-butyl)phenyl)methanone (**7e**)

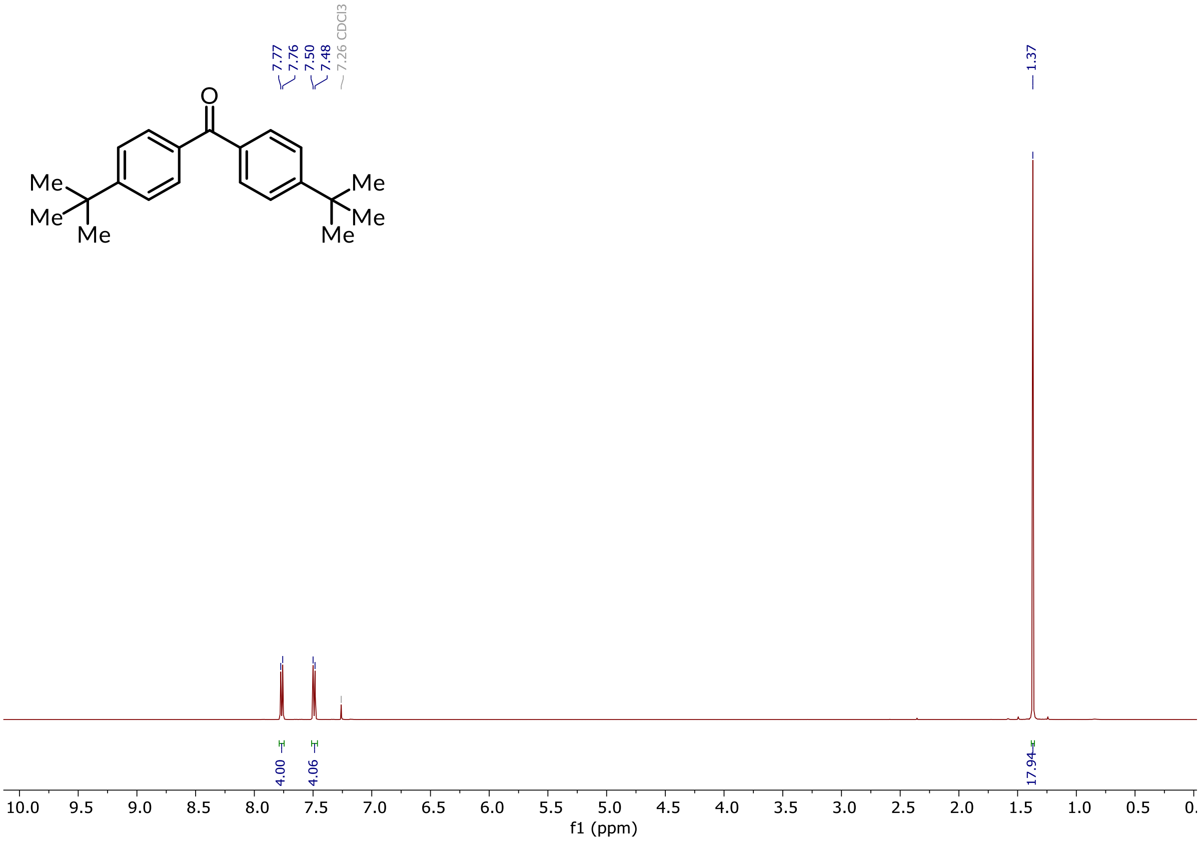

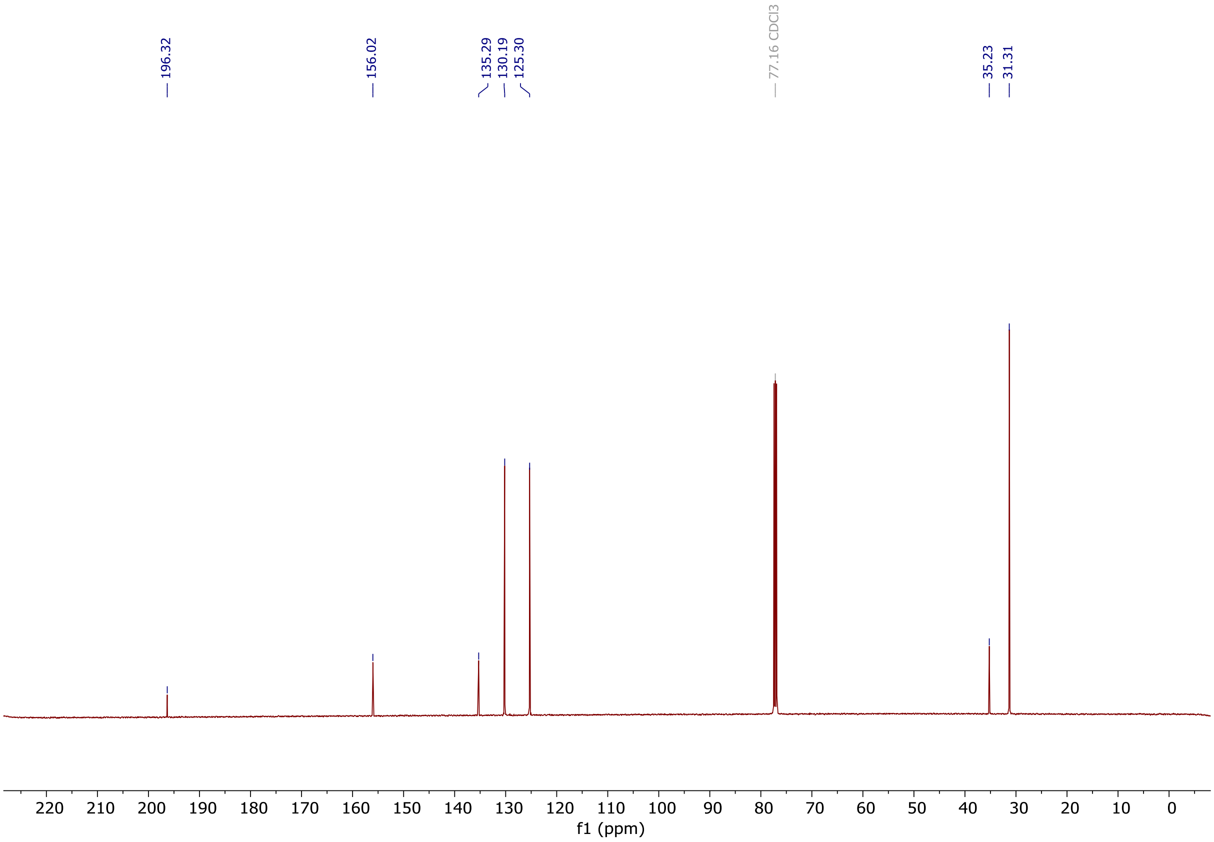

Di((1,1′-biphenyl)-4-yl)methanone (**7f**)

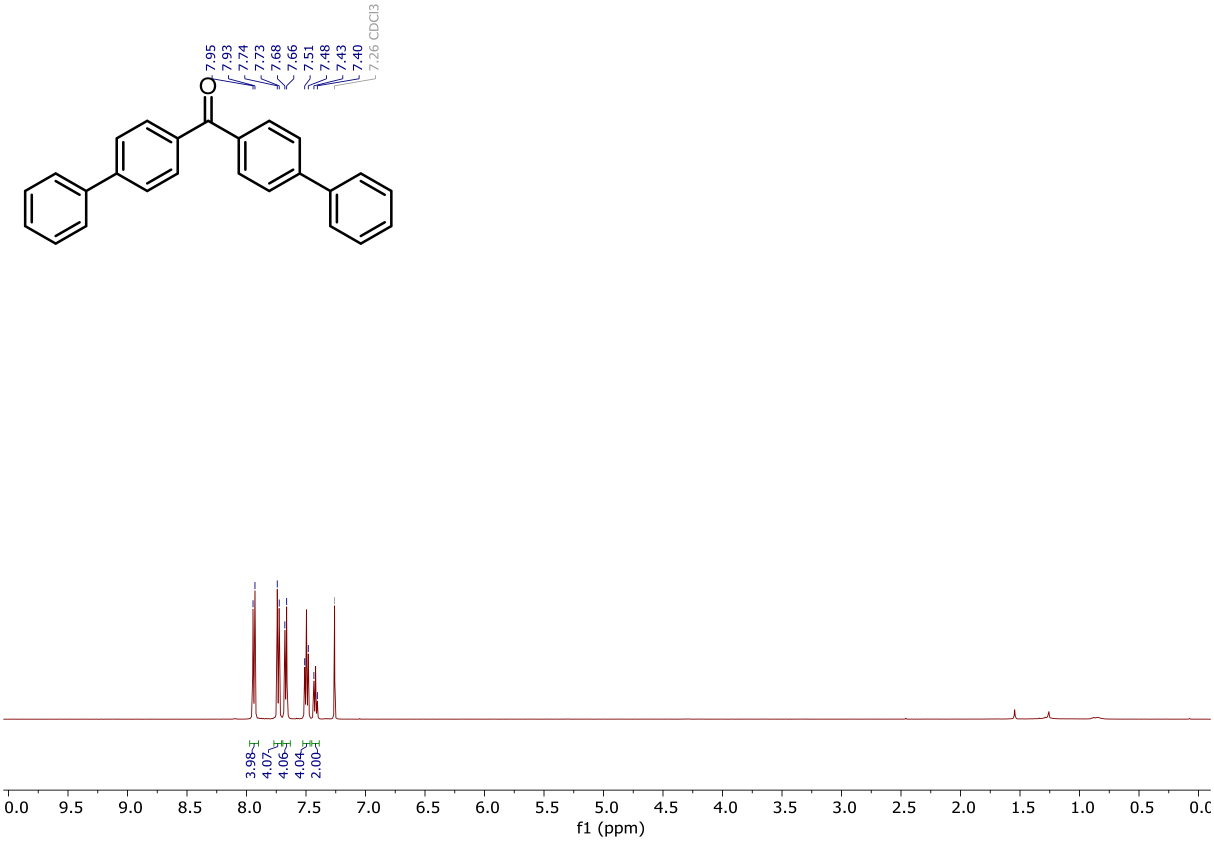

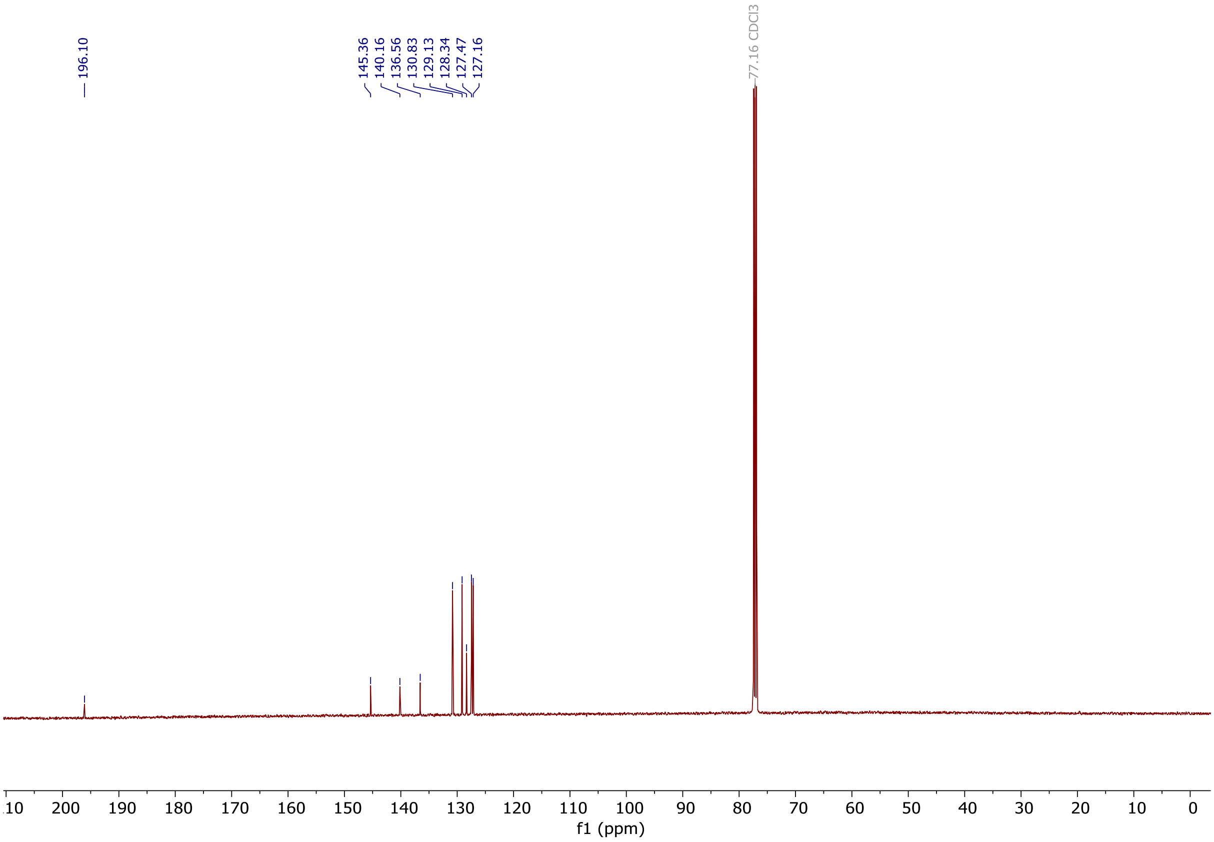

Bis(4-(methylthio)phenyl)methanone (**7k**)

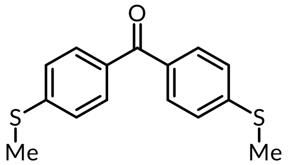

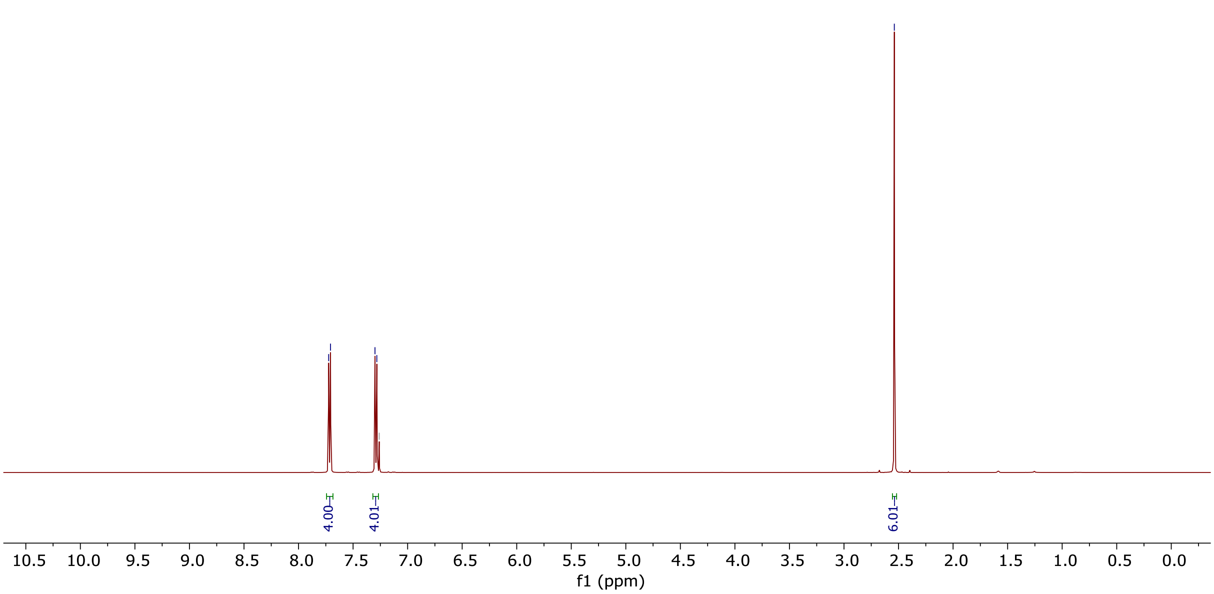

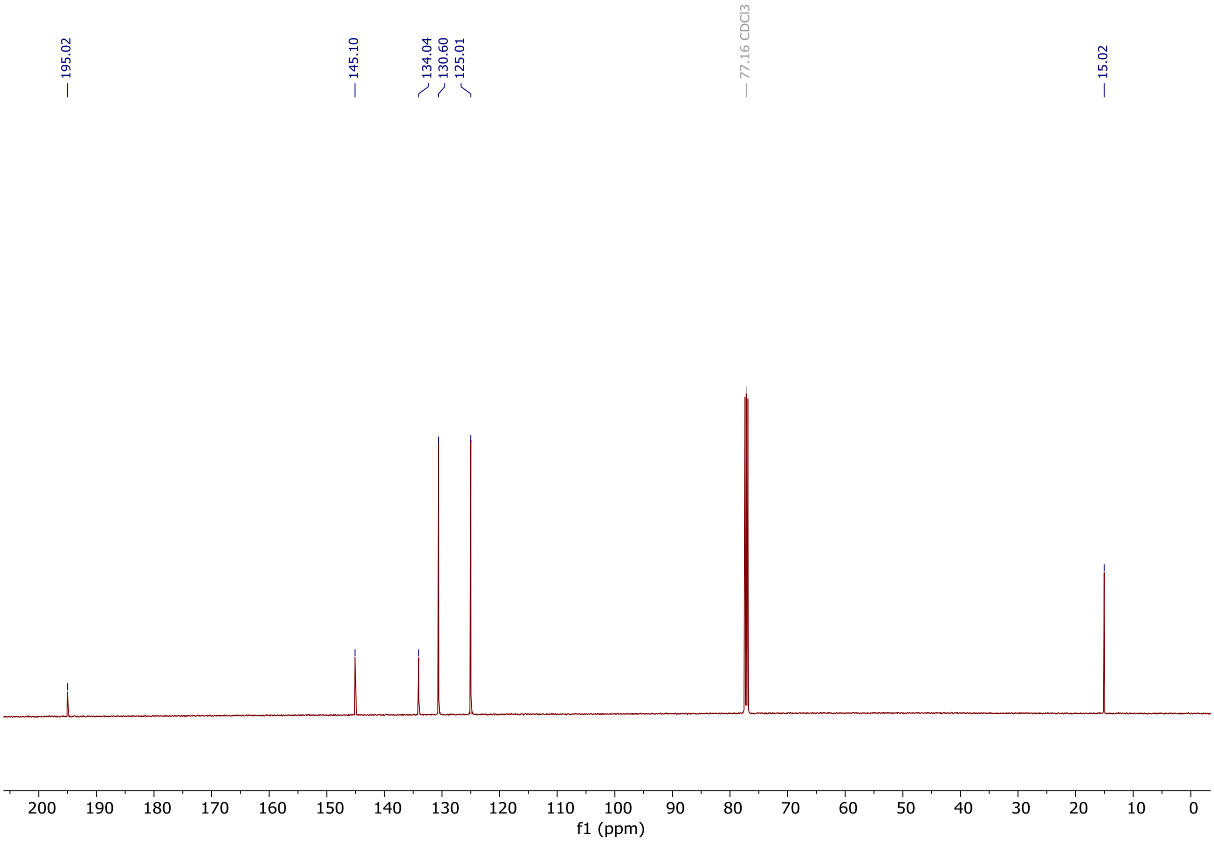

Bis(4-((trimethylsilyl)ethynyl)phenyl)methanone (**7l**)

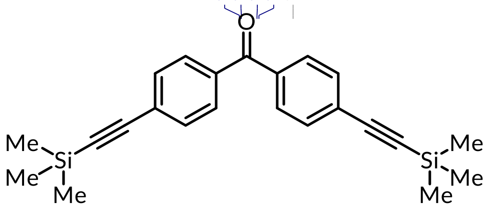

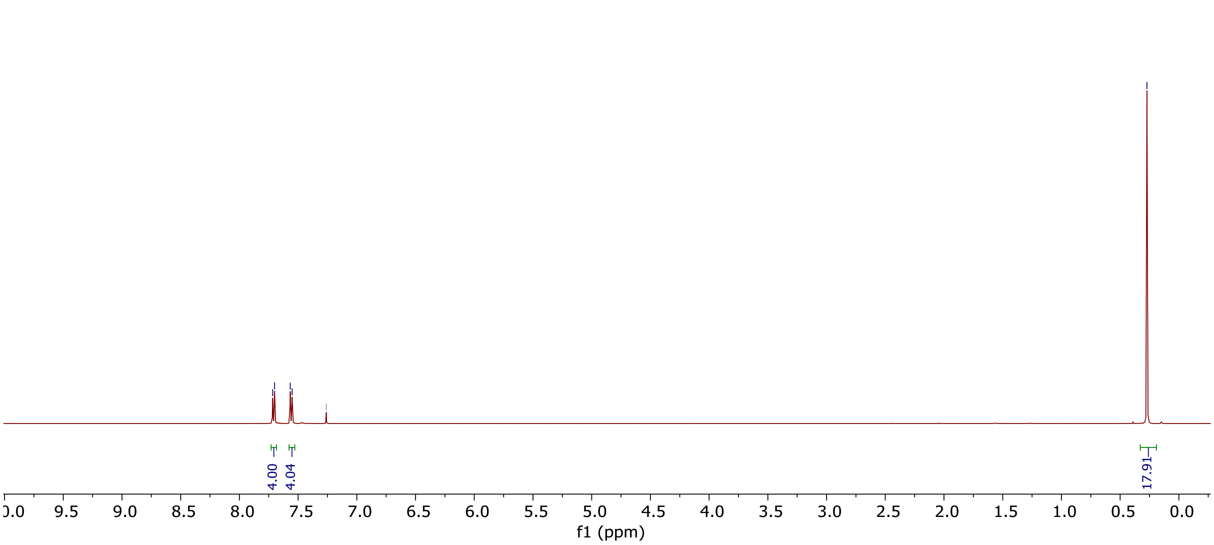

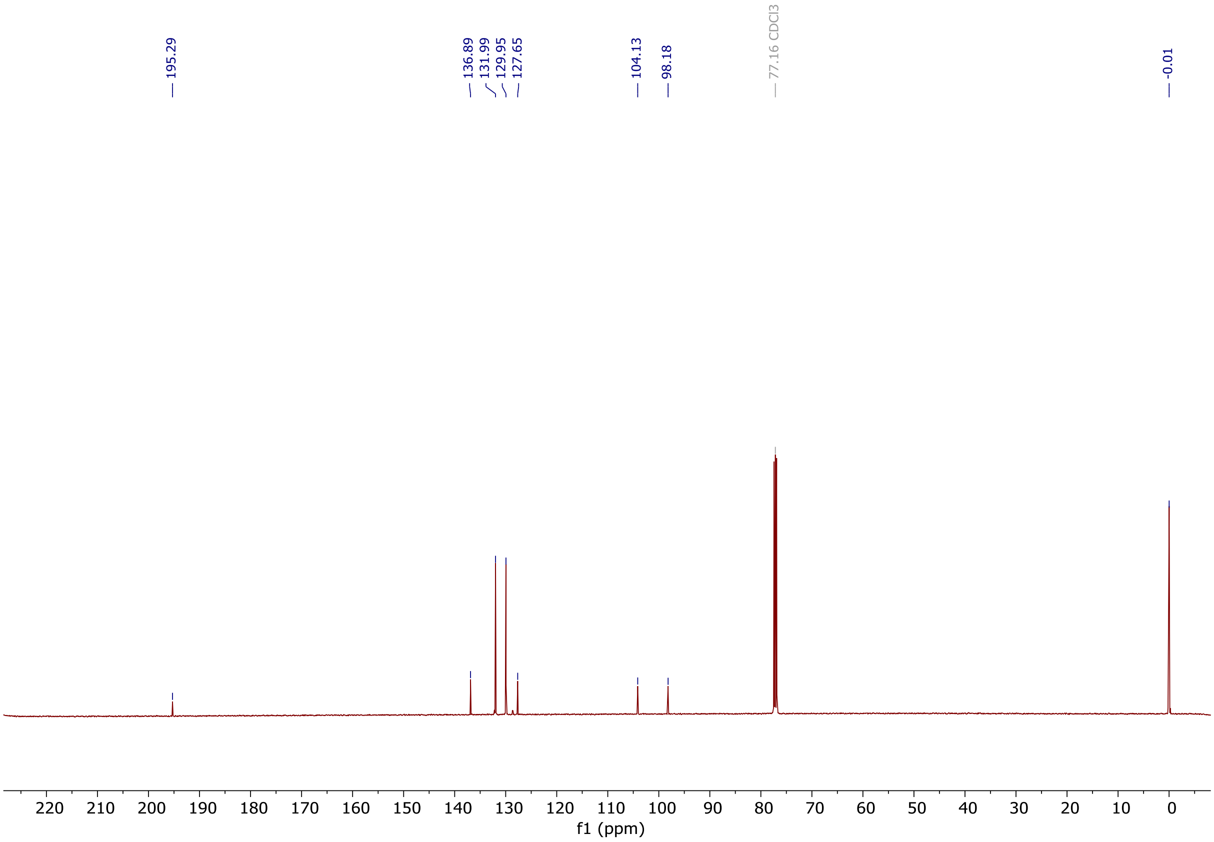

Bis(4-(trifluoromethyl)phenyl)methanone (**7m**)

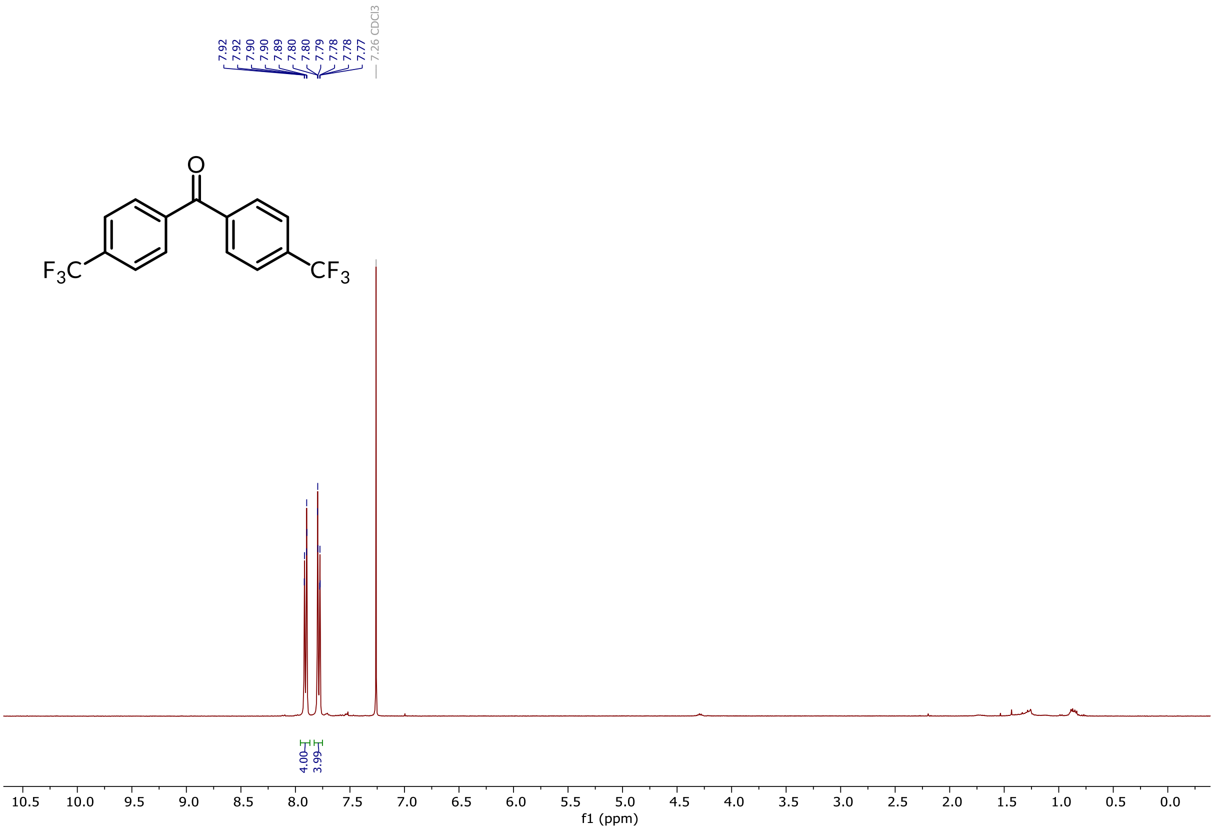

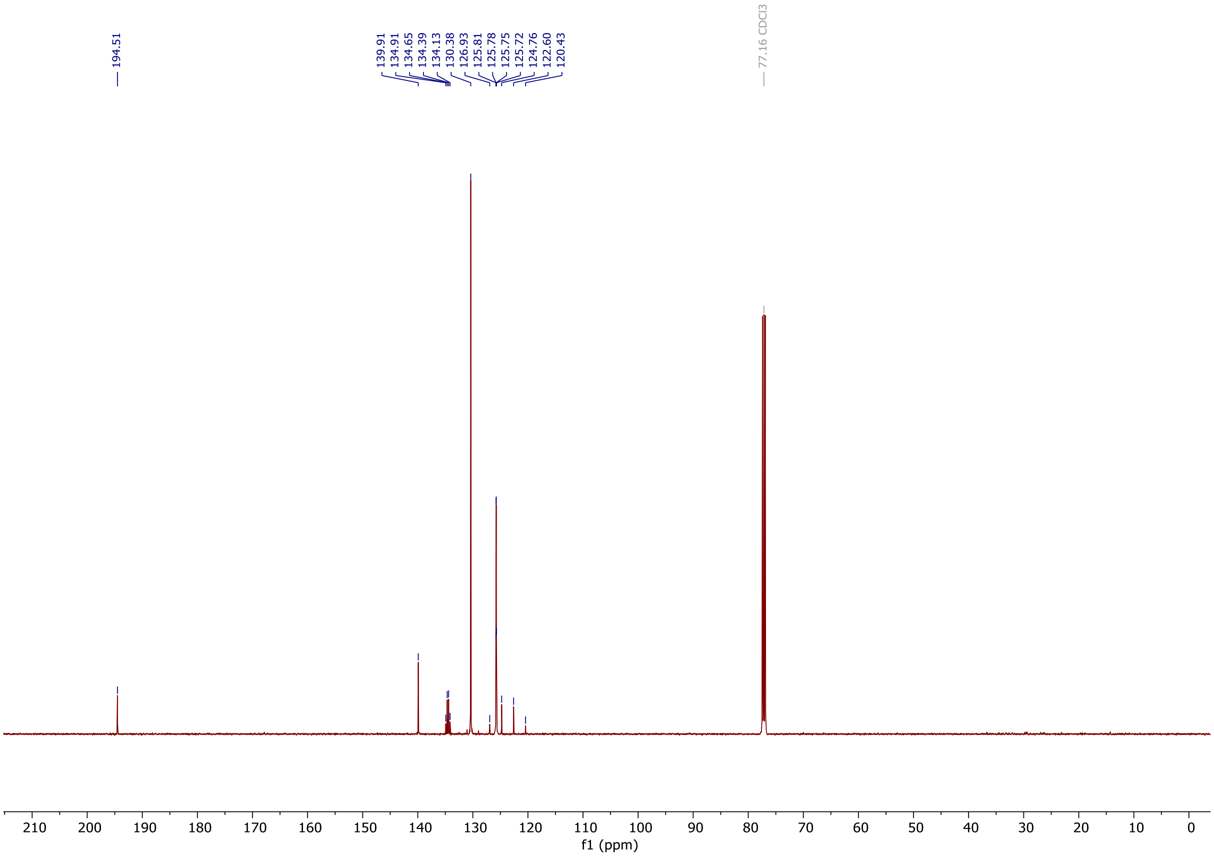

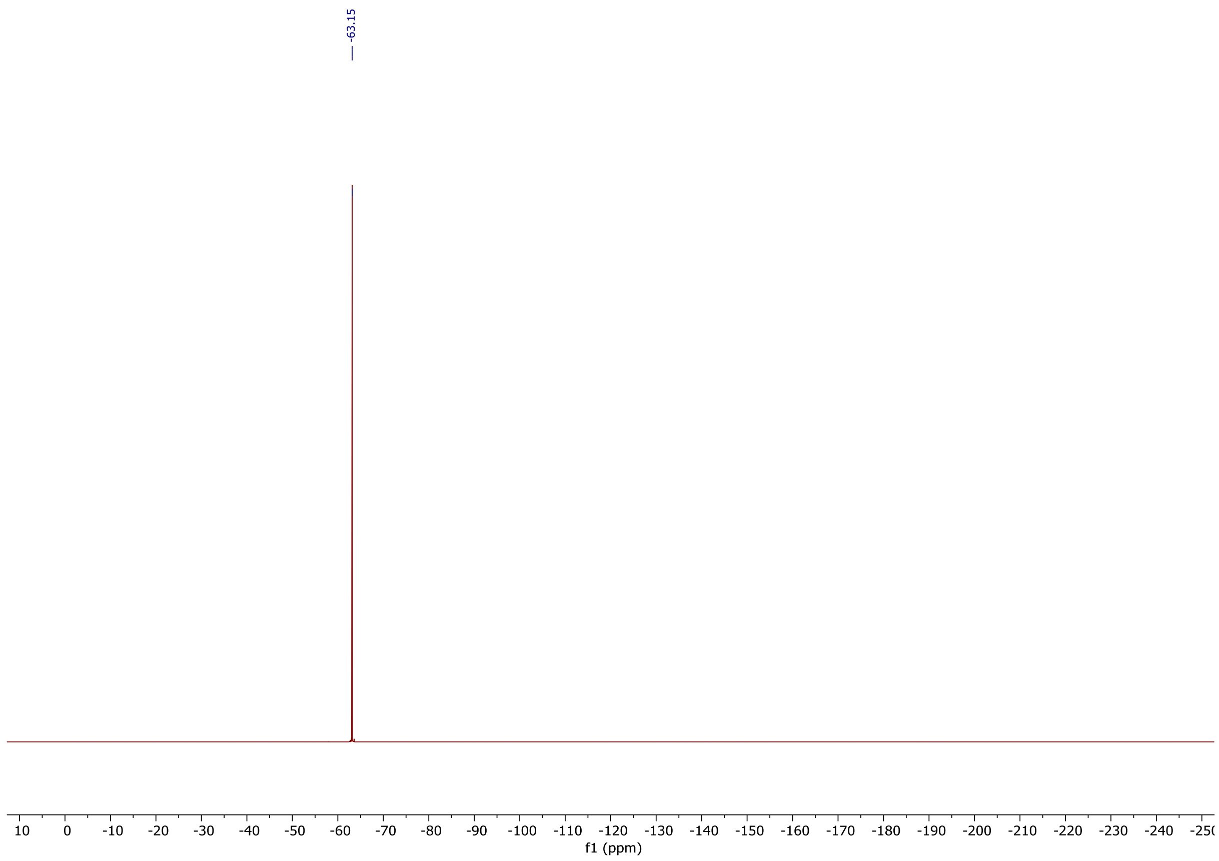
-63.15
10
0
-10
-20
-30
-40
-50
-60
-70
-80
-90
-100
-110
-120
-130
-140
-150
-160
-170
-180
-190
-200
-210
-220
-230
-240
f1 (ppm)

Bis(3-chlorophenyl)methanone (**7o**)

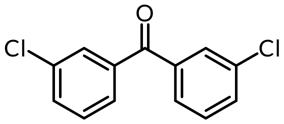

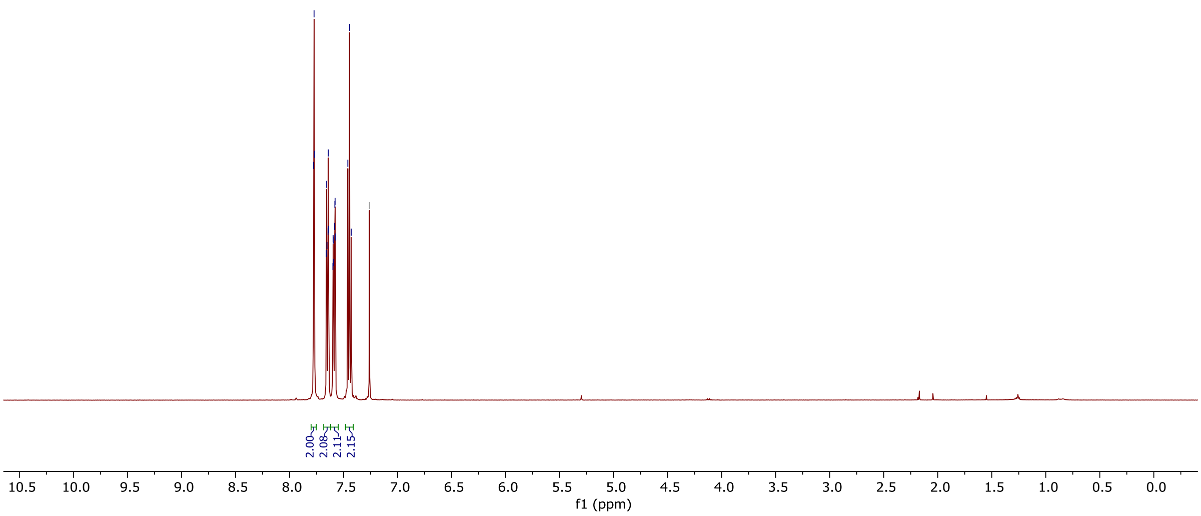

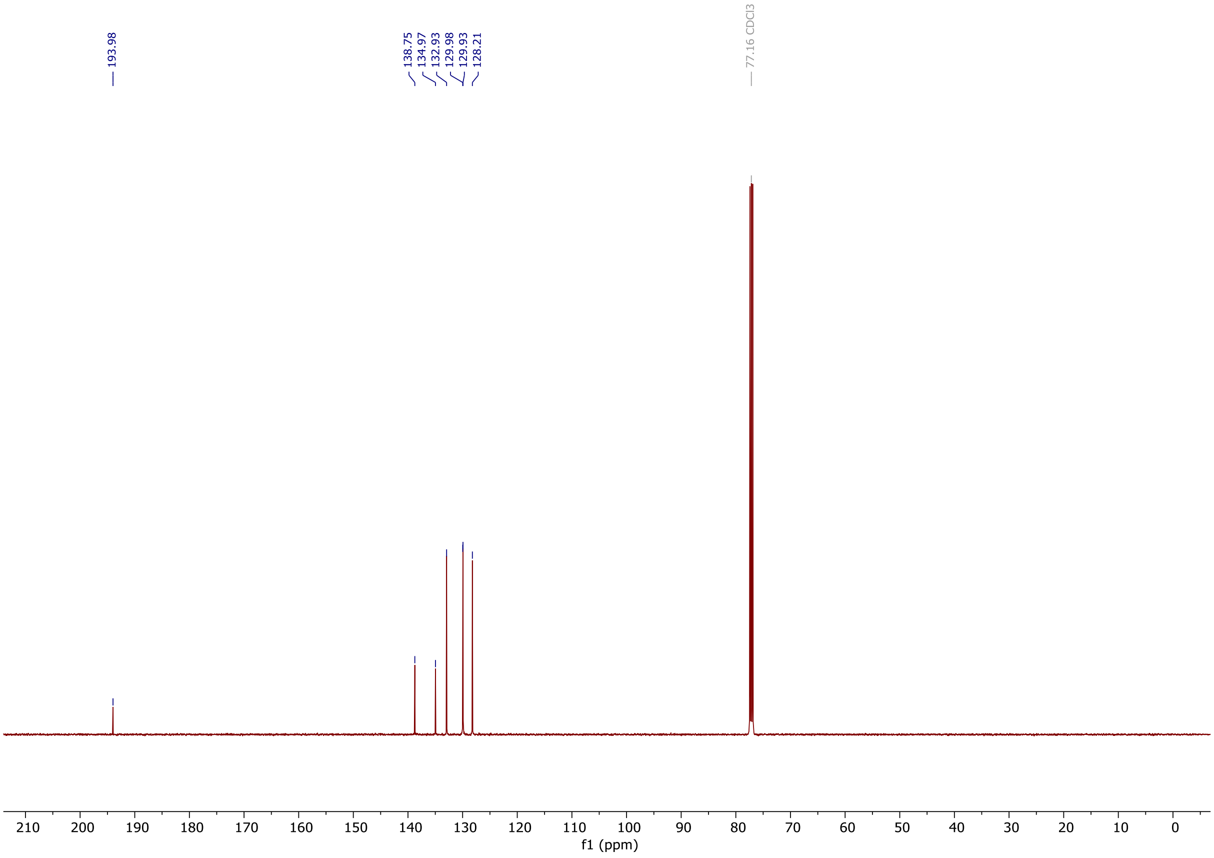

Di-*m*-tolylmethanone (**7q**)

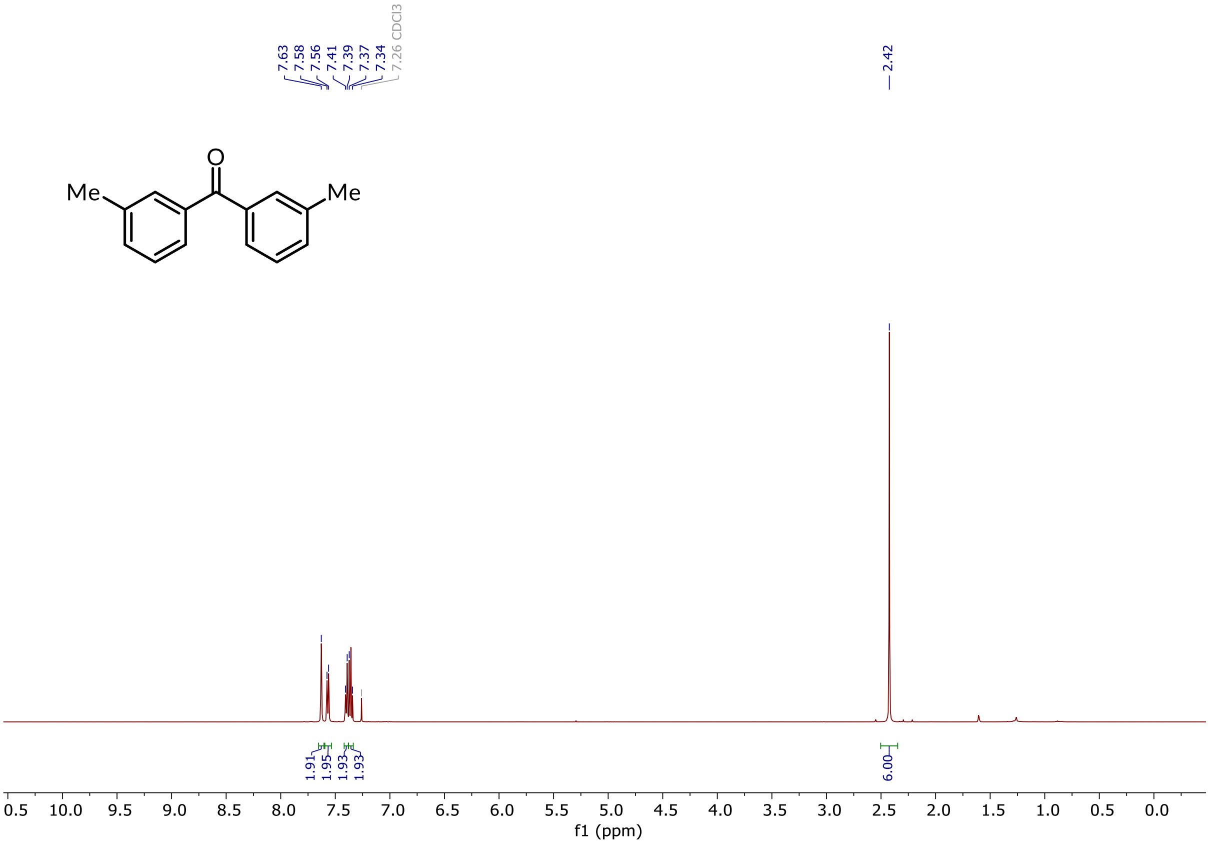

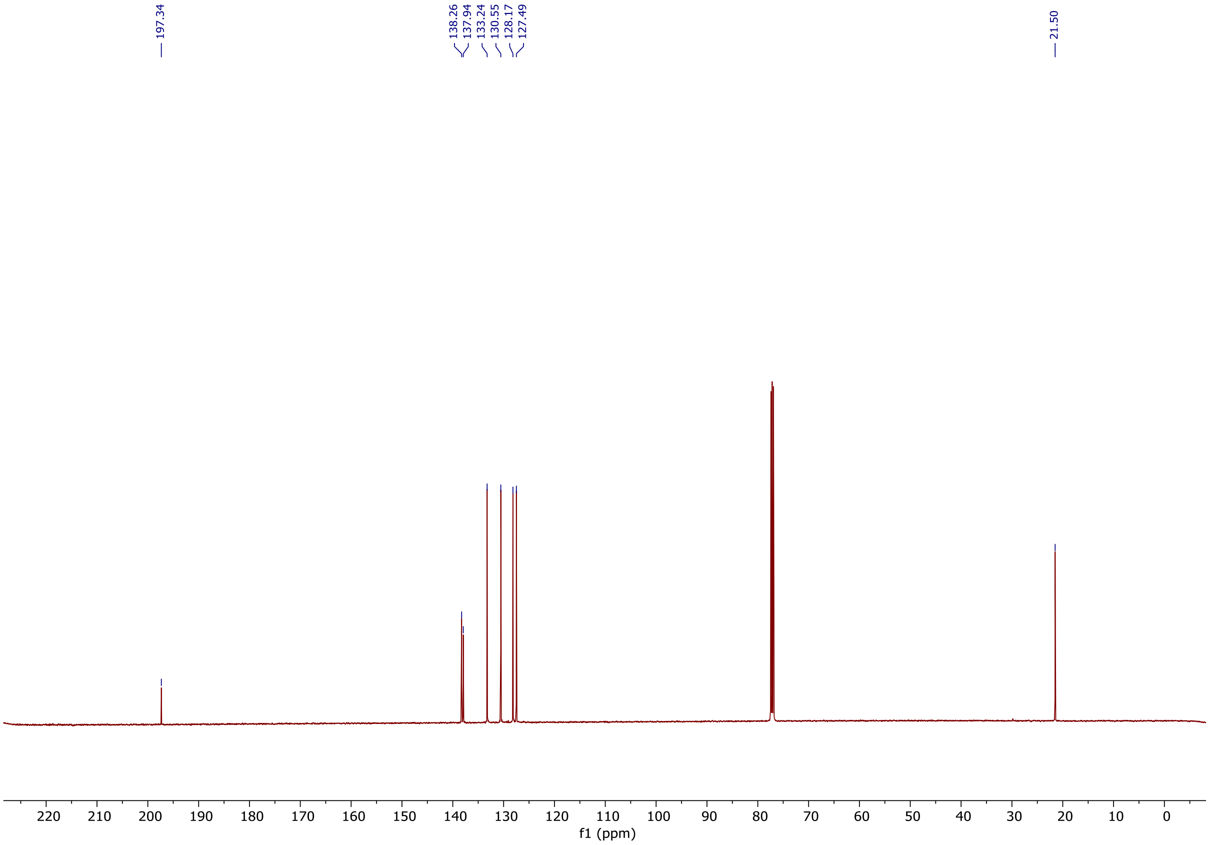

Bis(3-methoxyphenyl)methanone (**7r**)

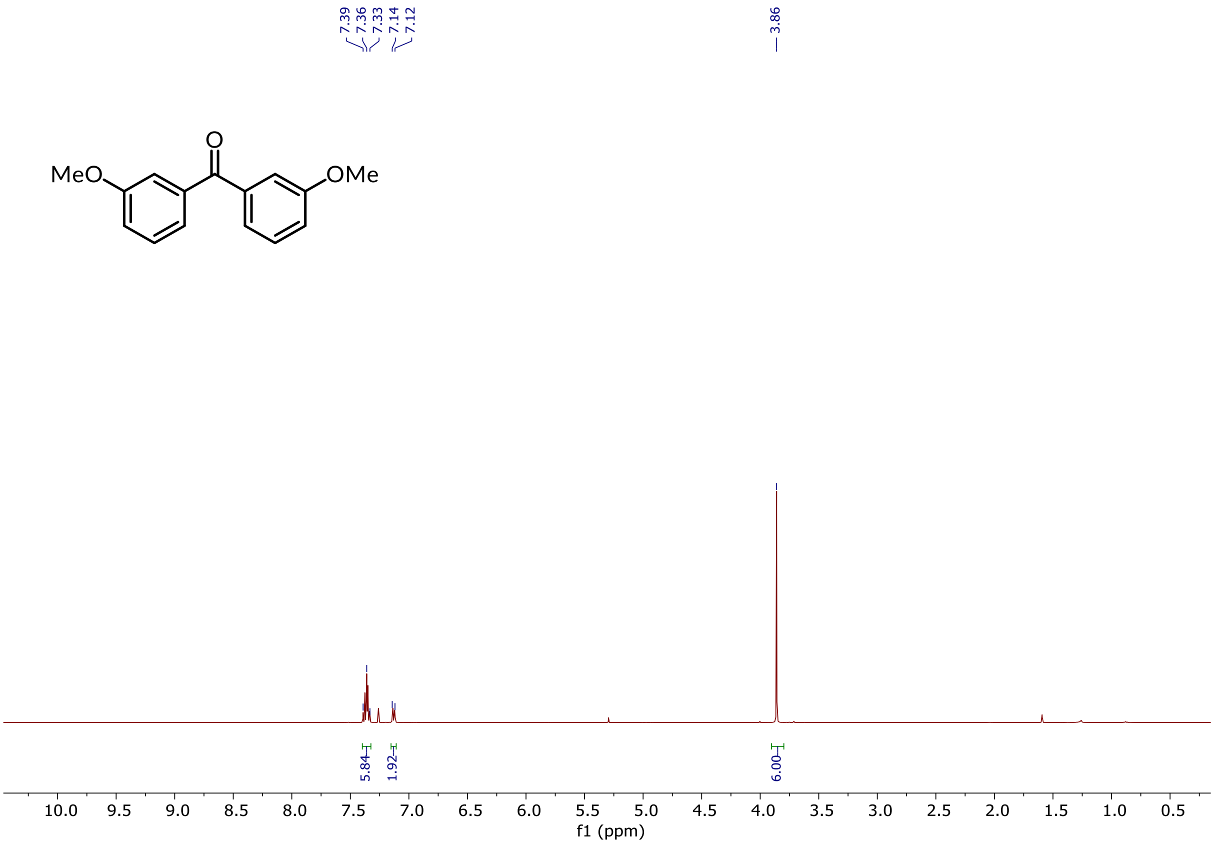

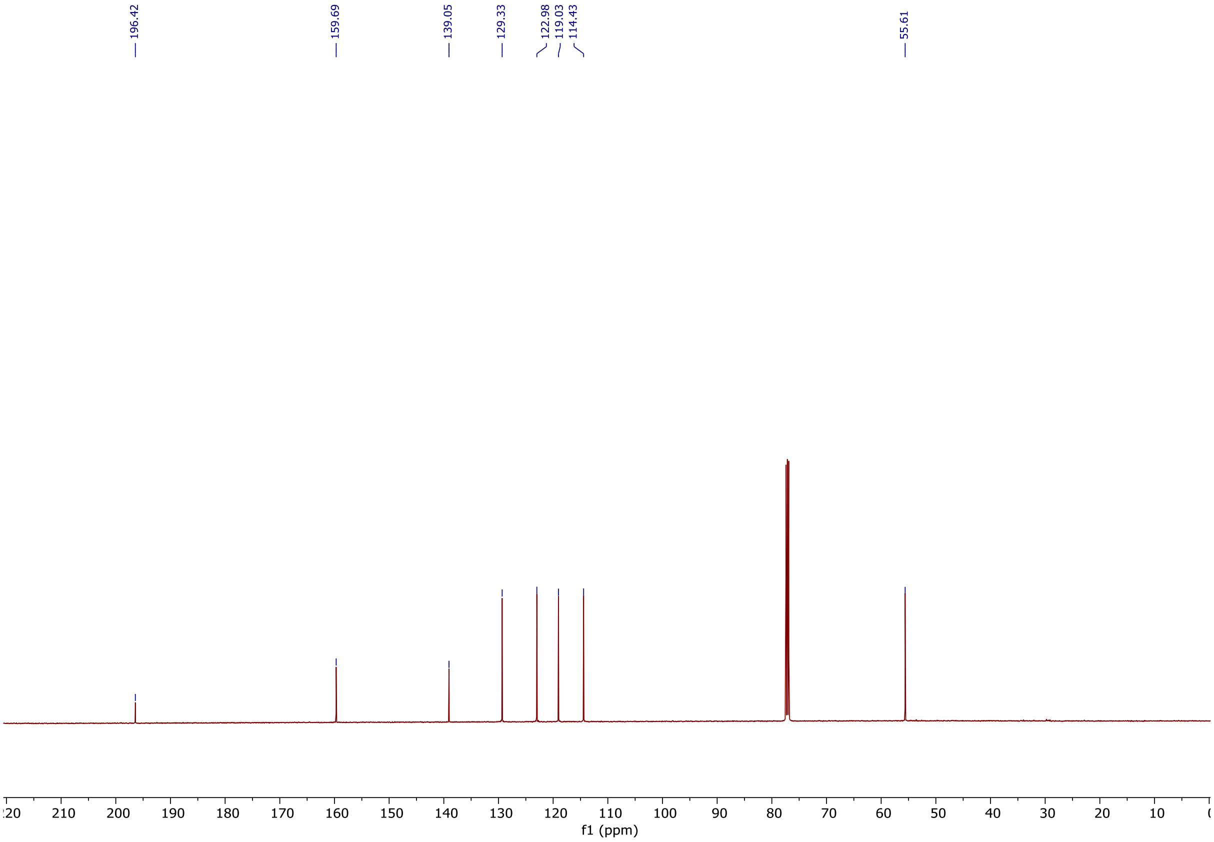

Di-*o*-tolylmethanone (**7t**)

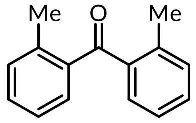

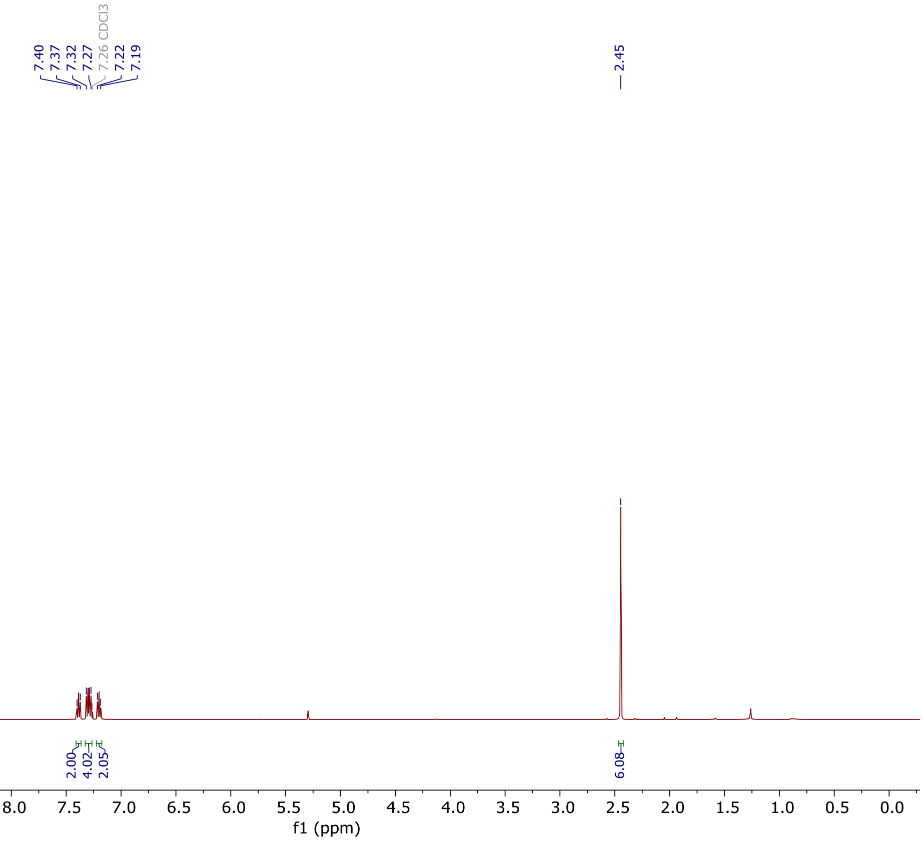

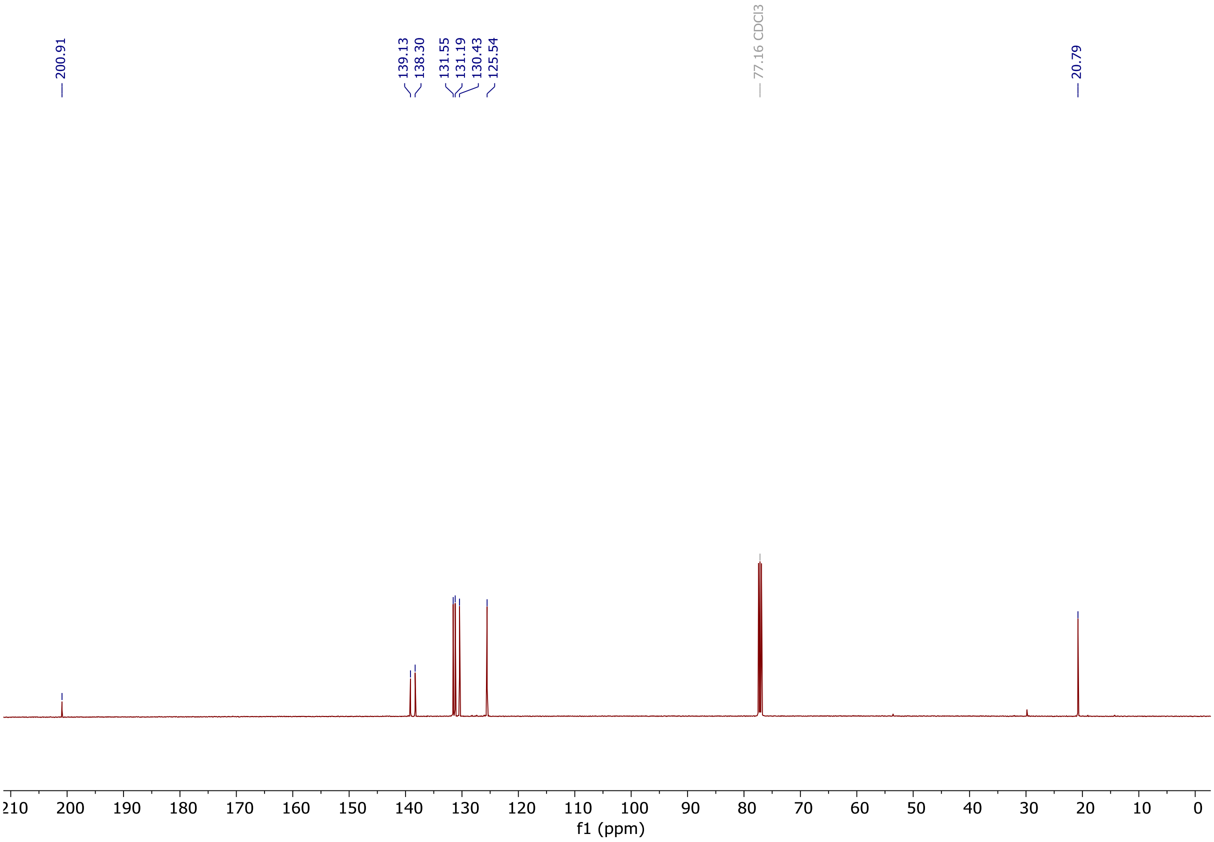

Bis(2-methoxyphenyl)methanone (**7u**)

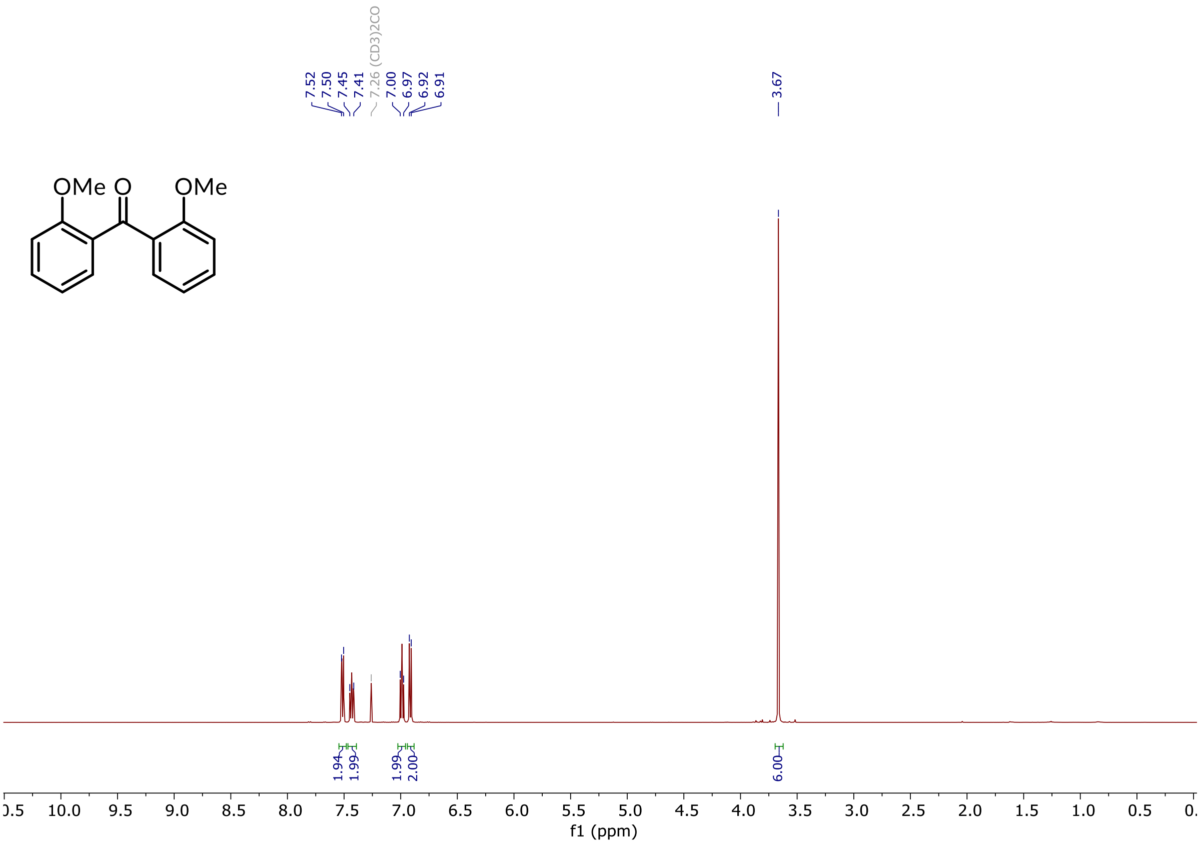

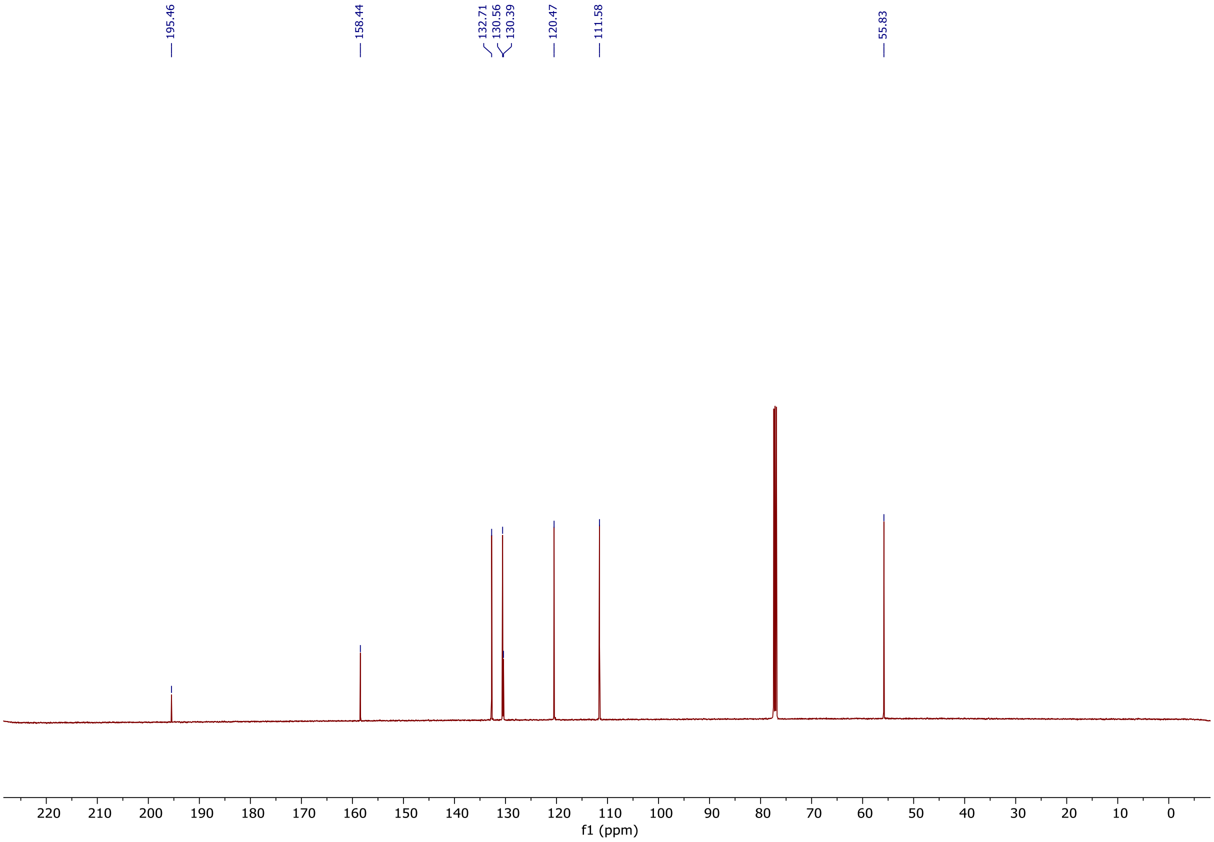

Bis(2-fluorophenyl)methanone (**7v**)

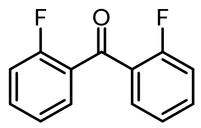

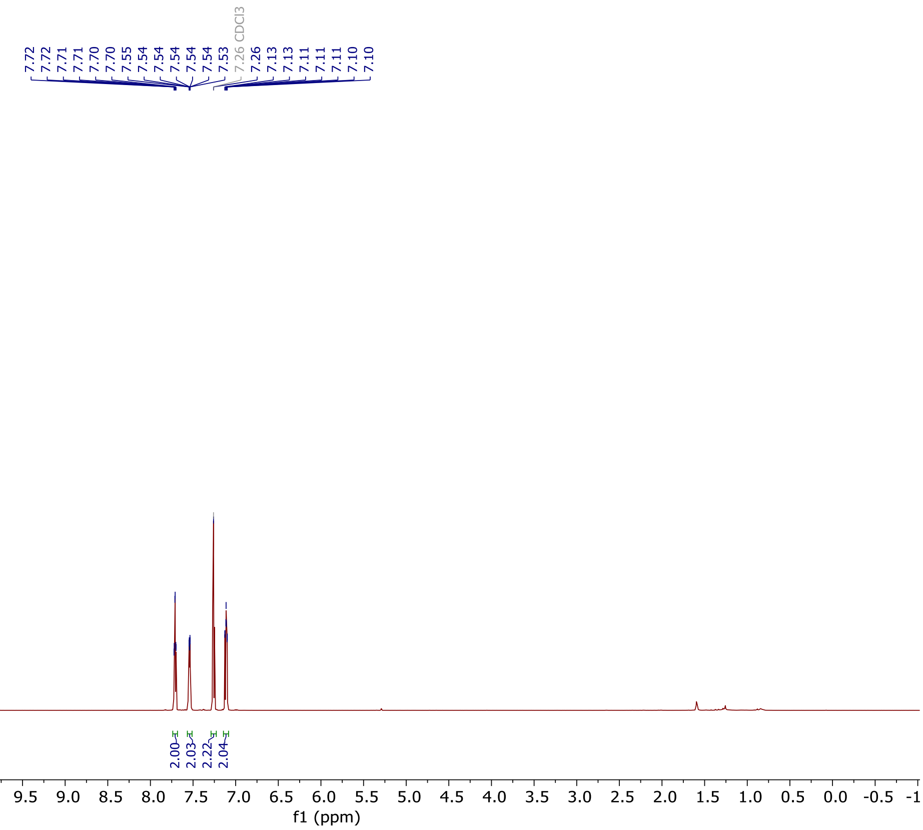

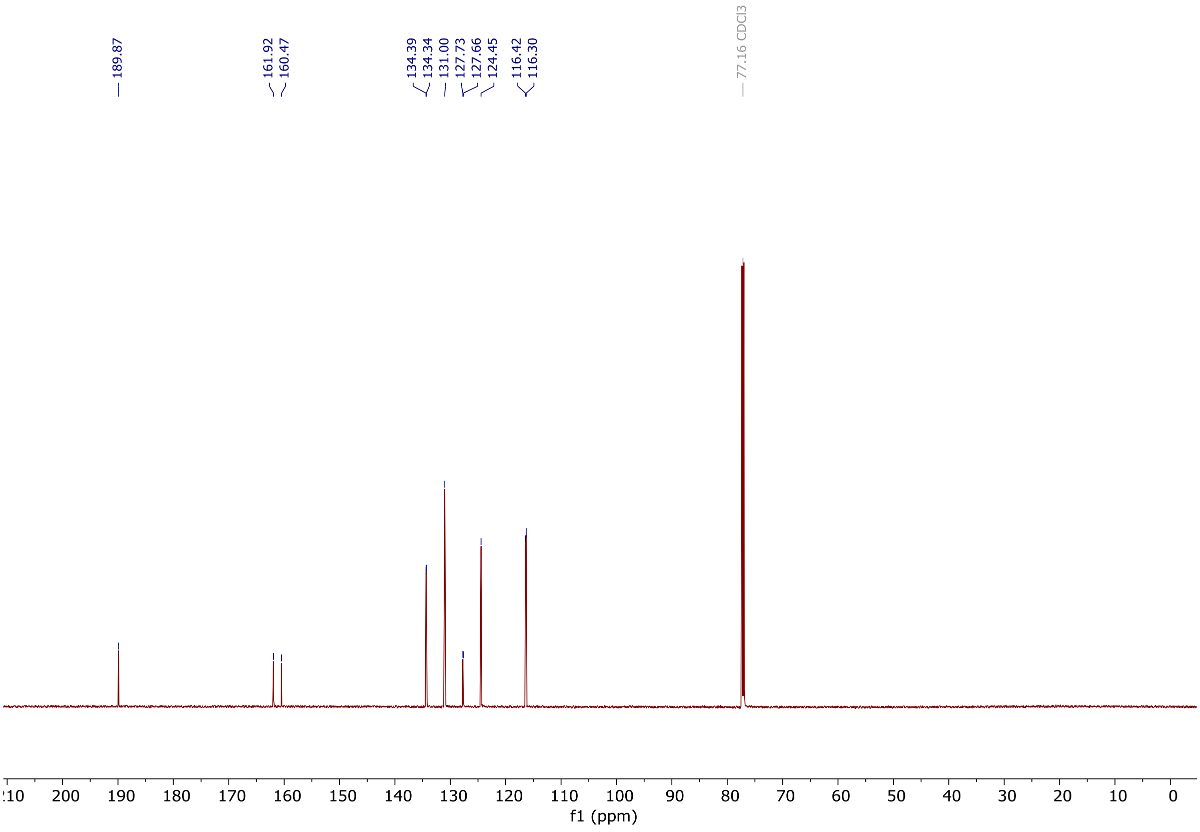

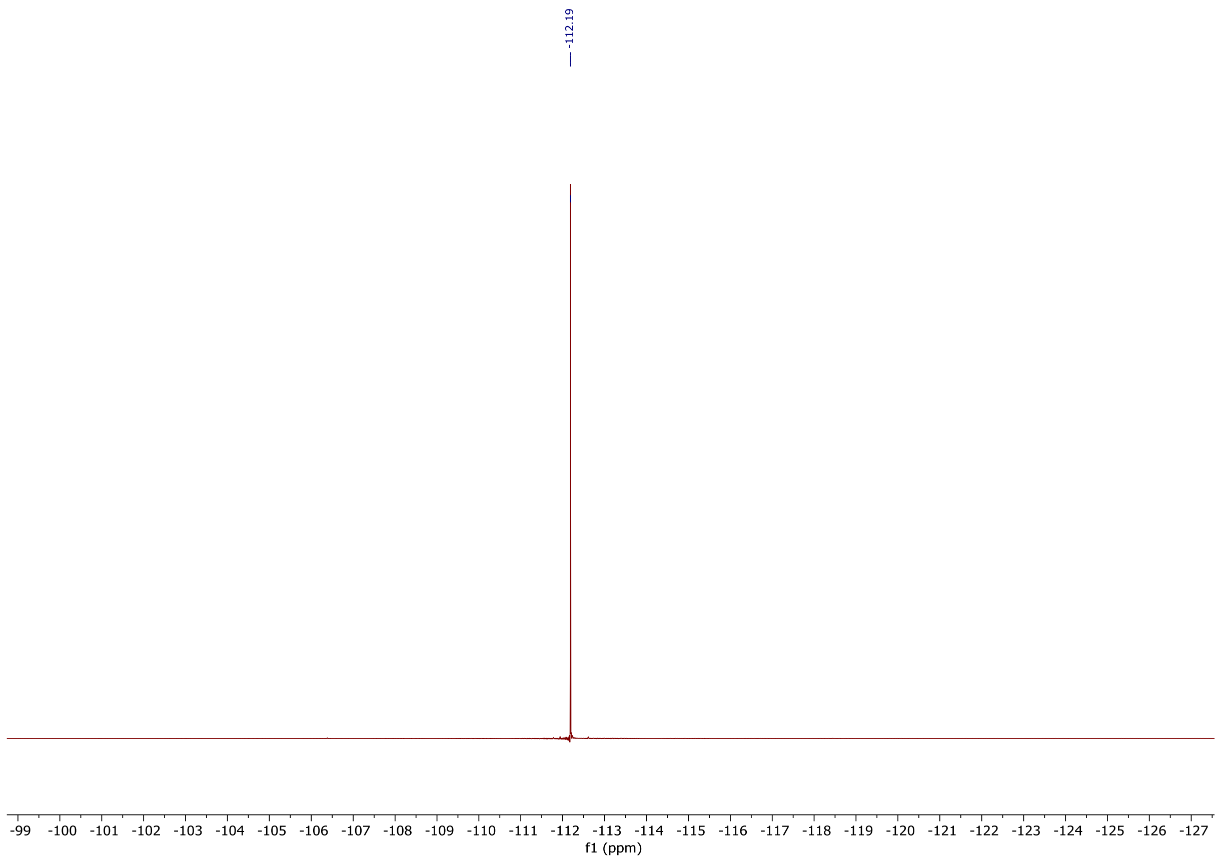
-112.19
-99 -100 -101 -102 -103 -104 -105 -106 -107 -108 -109 -110 -111 -112 -113 -114 -115 -116 -117 -118 -119 -120 -121 -122 -123 -124 -125 -126 -127
f1 (ppm)

Di(benzofuran-2-yl)methanone (**7w**)

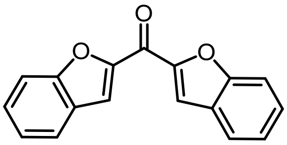

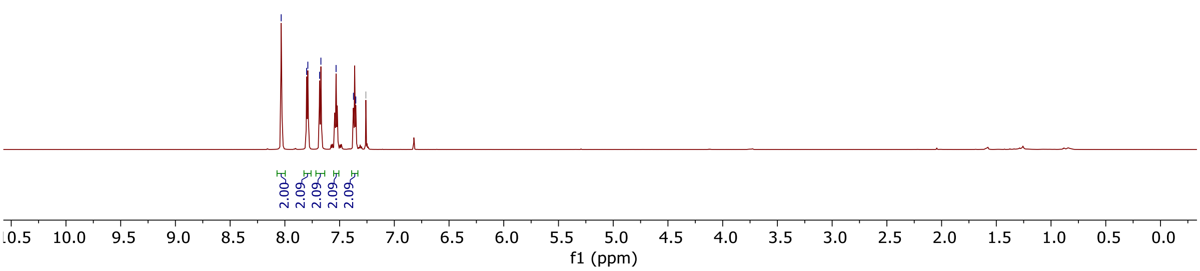

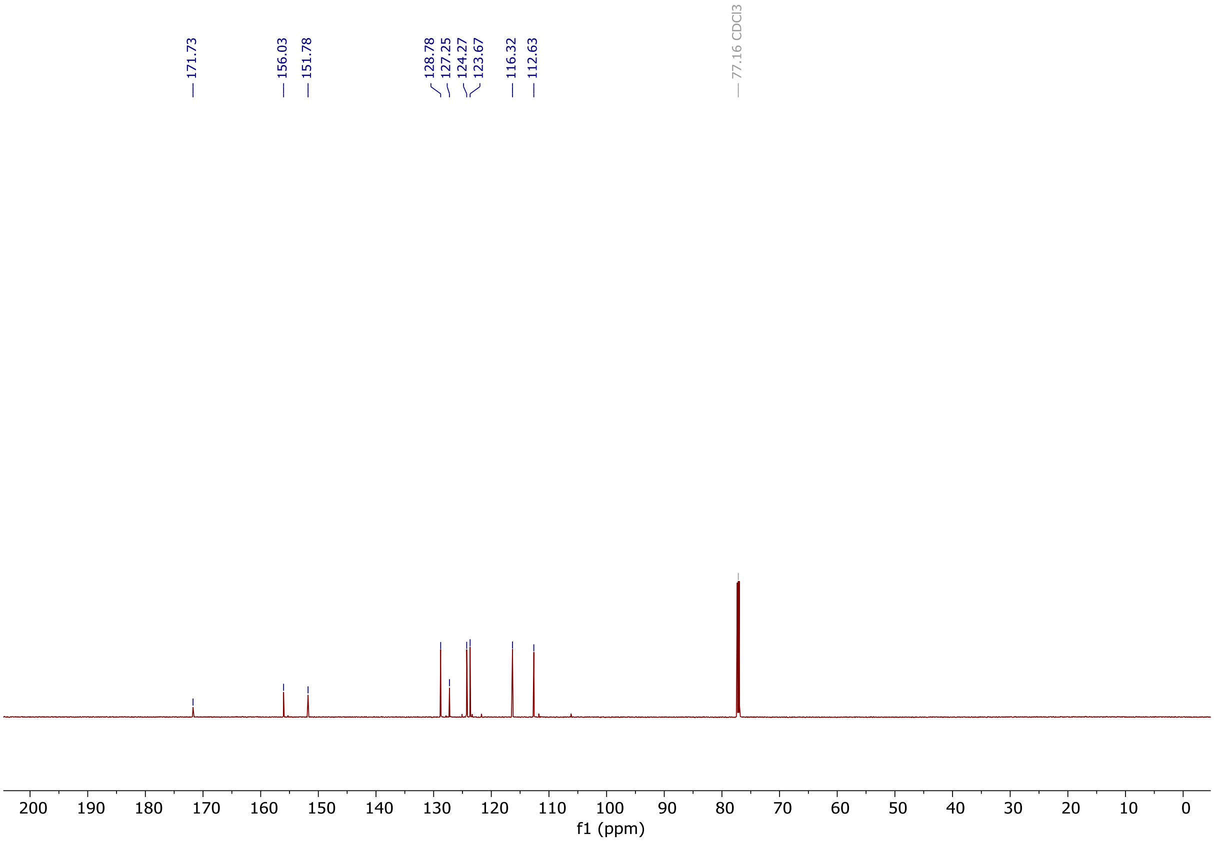

Bis(thiobenzophen-2-yl)methanone (**7x**)

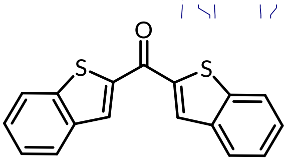

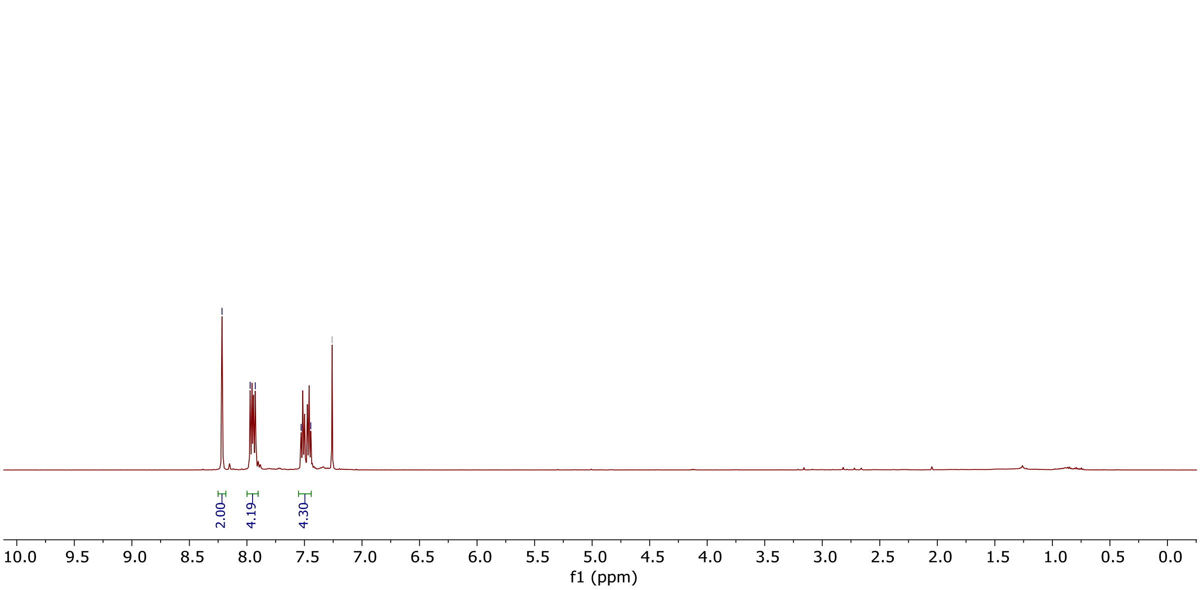

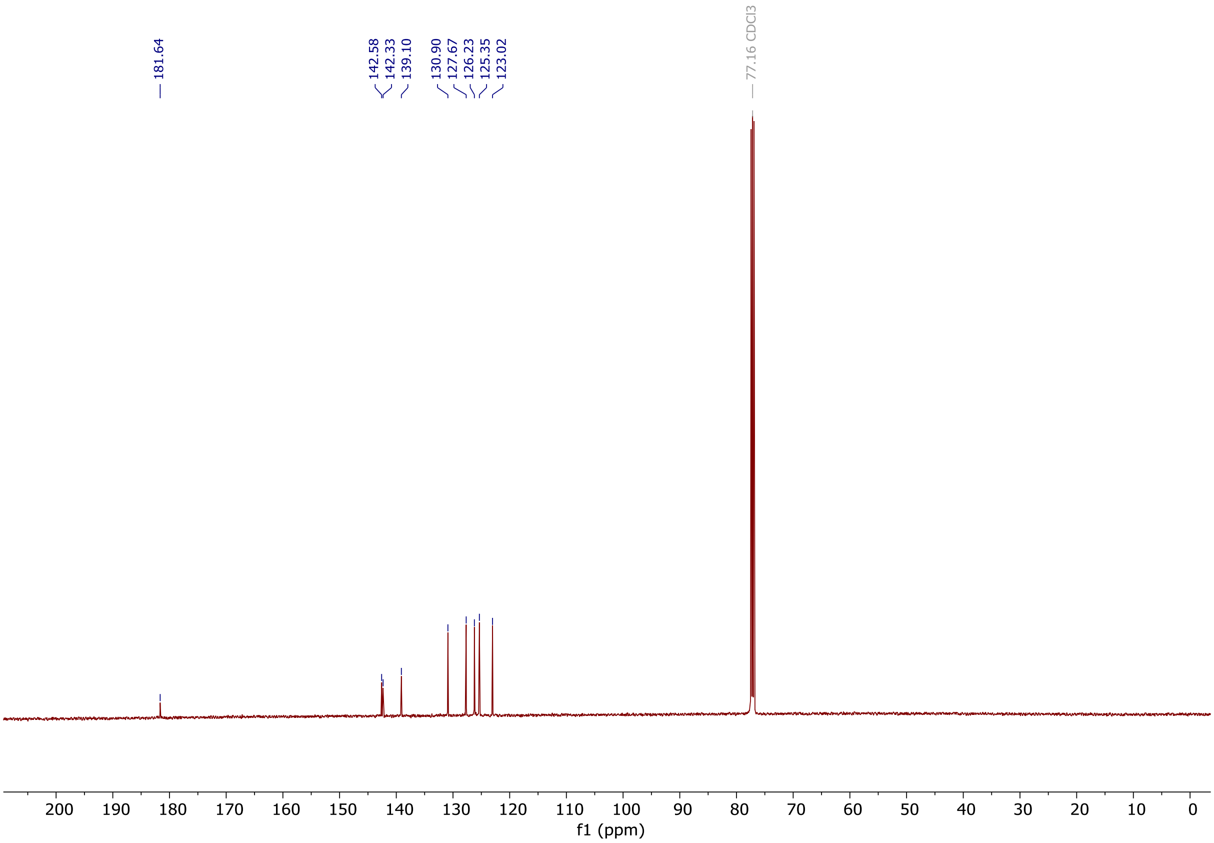

Bis(6-methoxynaphthalen-2-yl)methanone (**7z**)

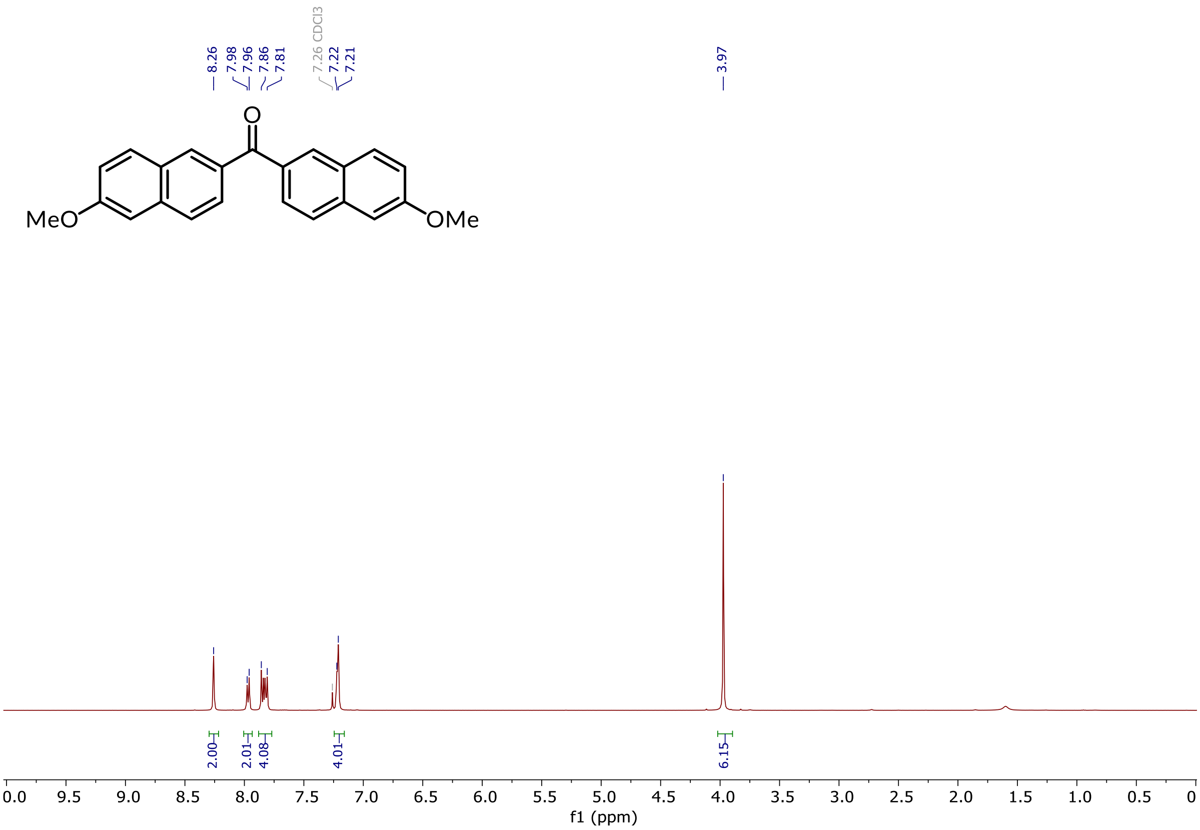

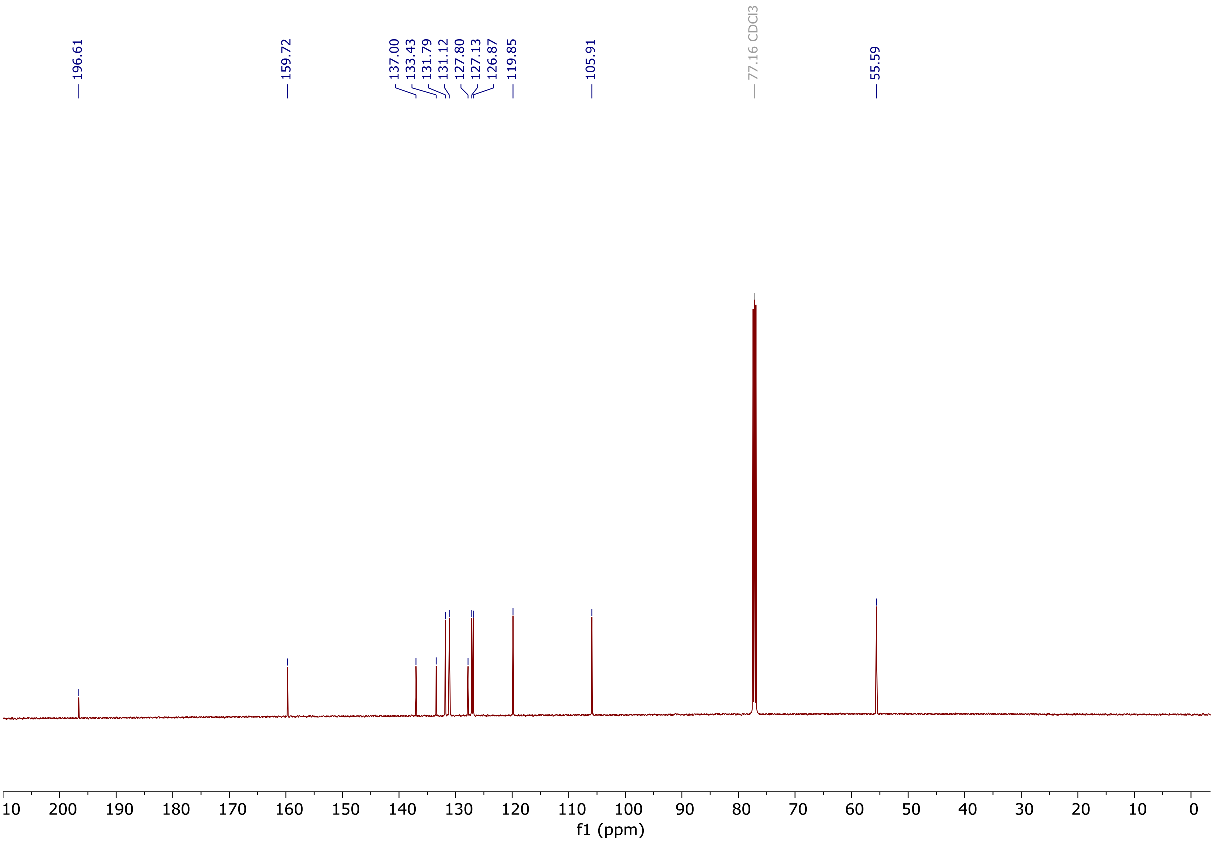

Bis(4-(pyridine-4-yl)phenyl)methanone (**7za**)

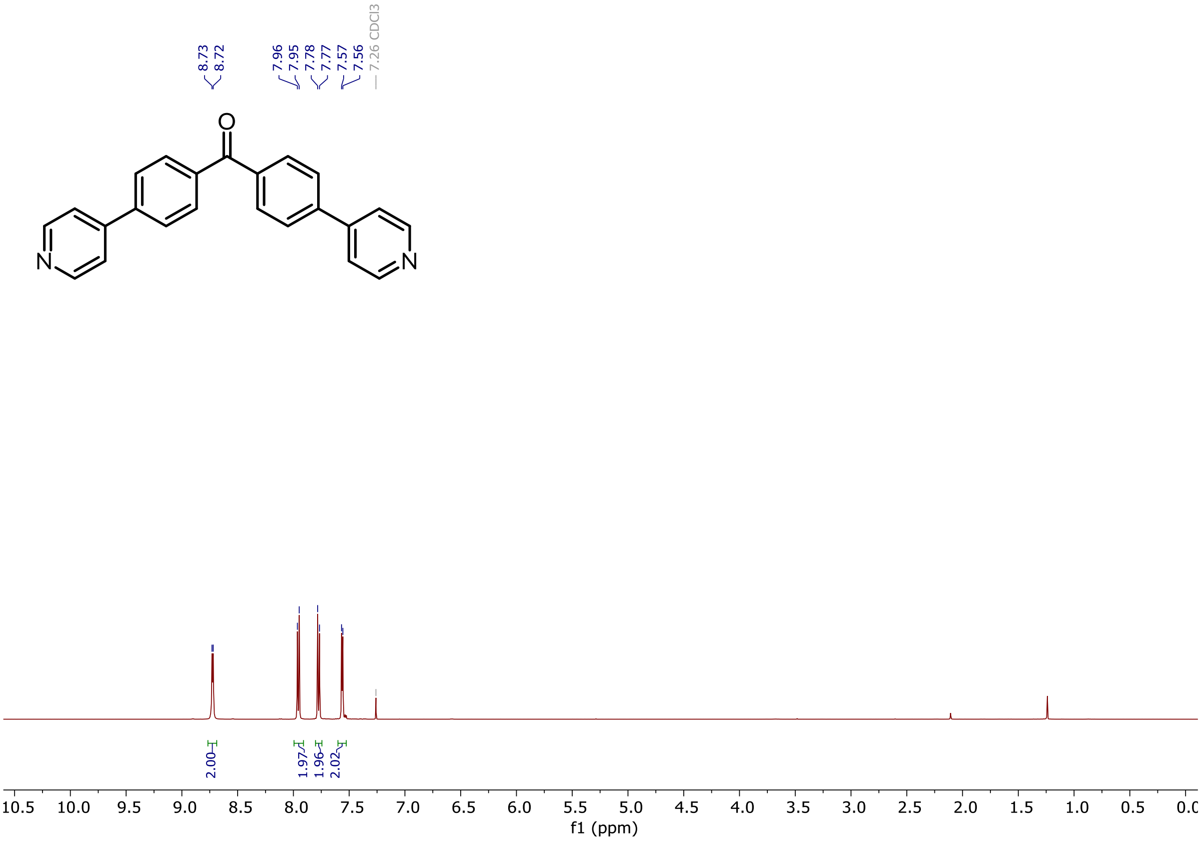

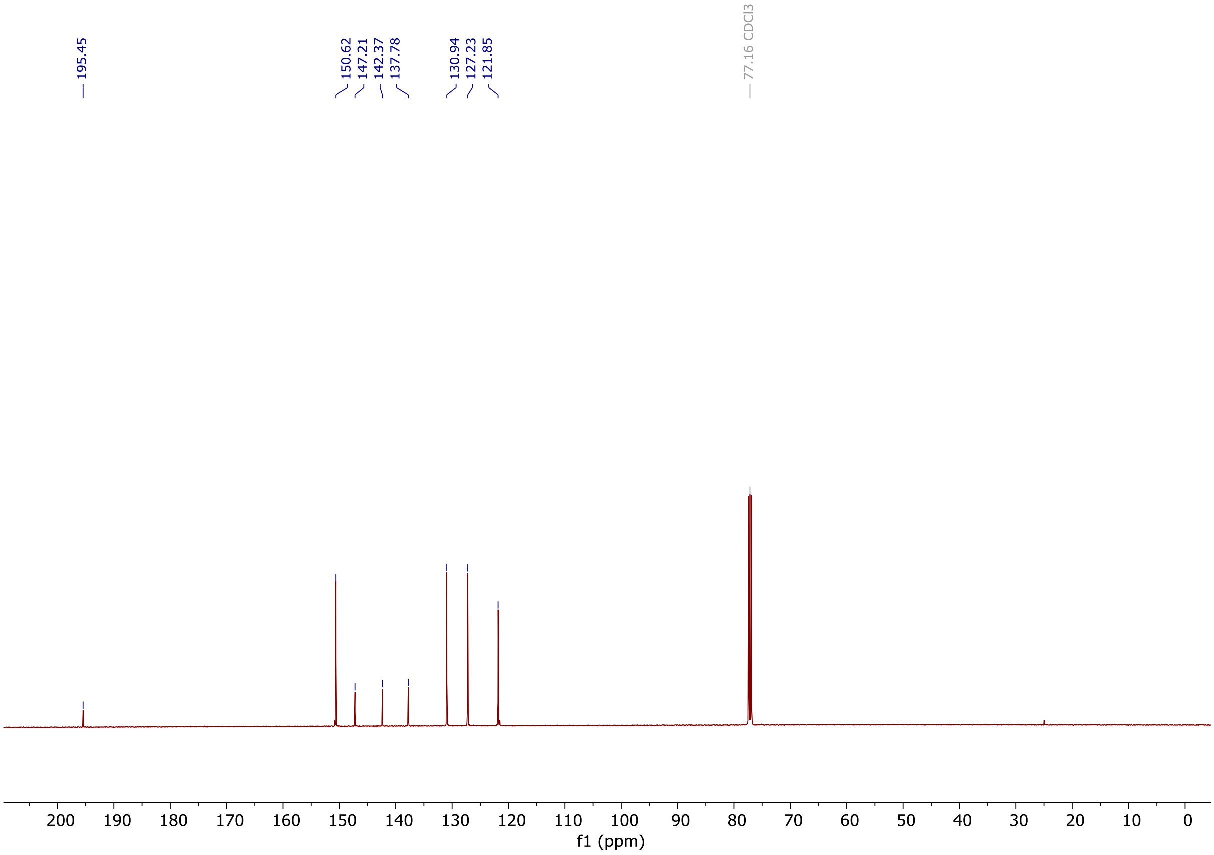

1,1-Bis(4-fluorophenyl)prop-2-en-1-ol (**1a**)

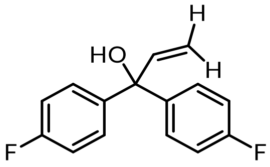

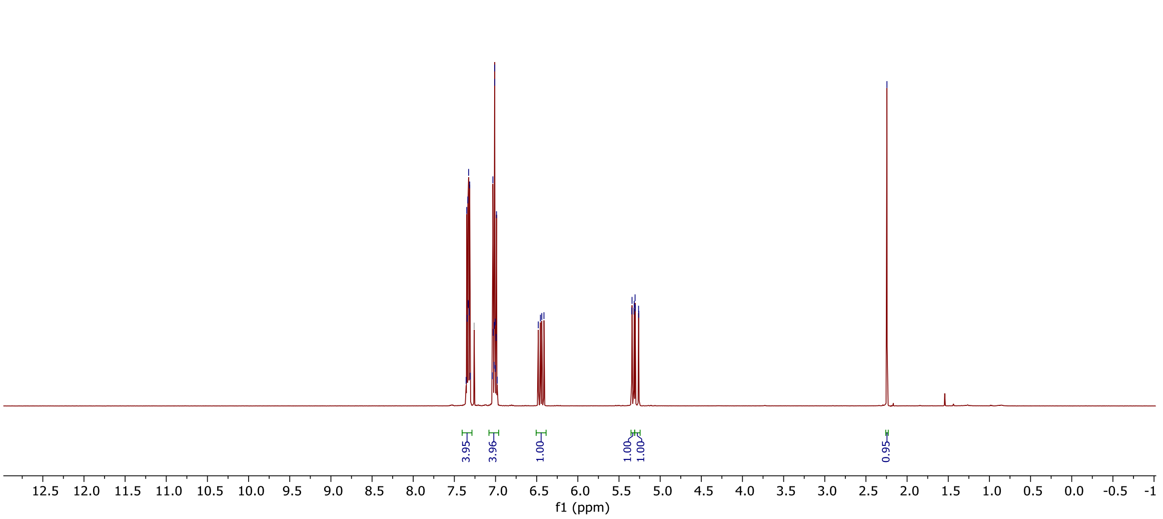

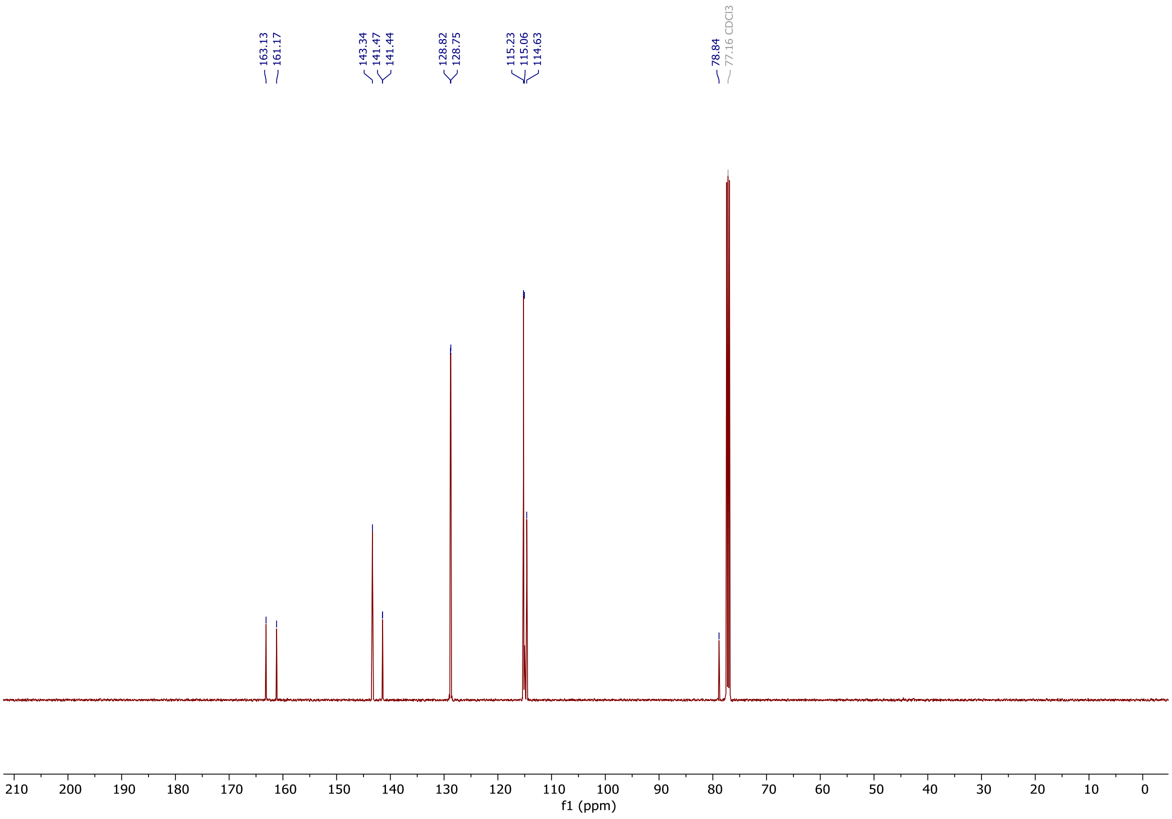

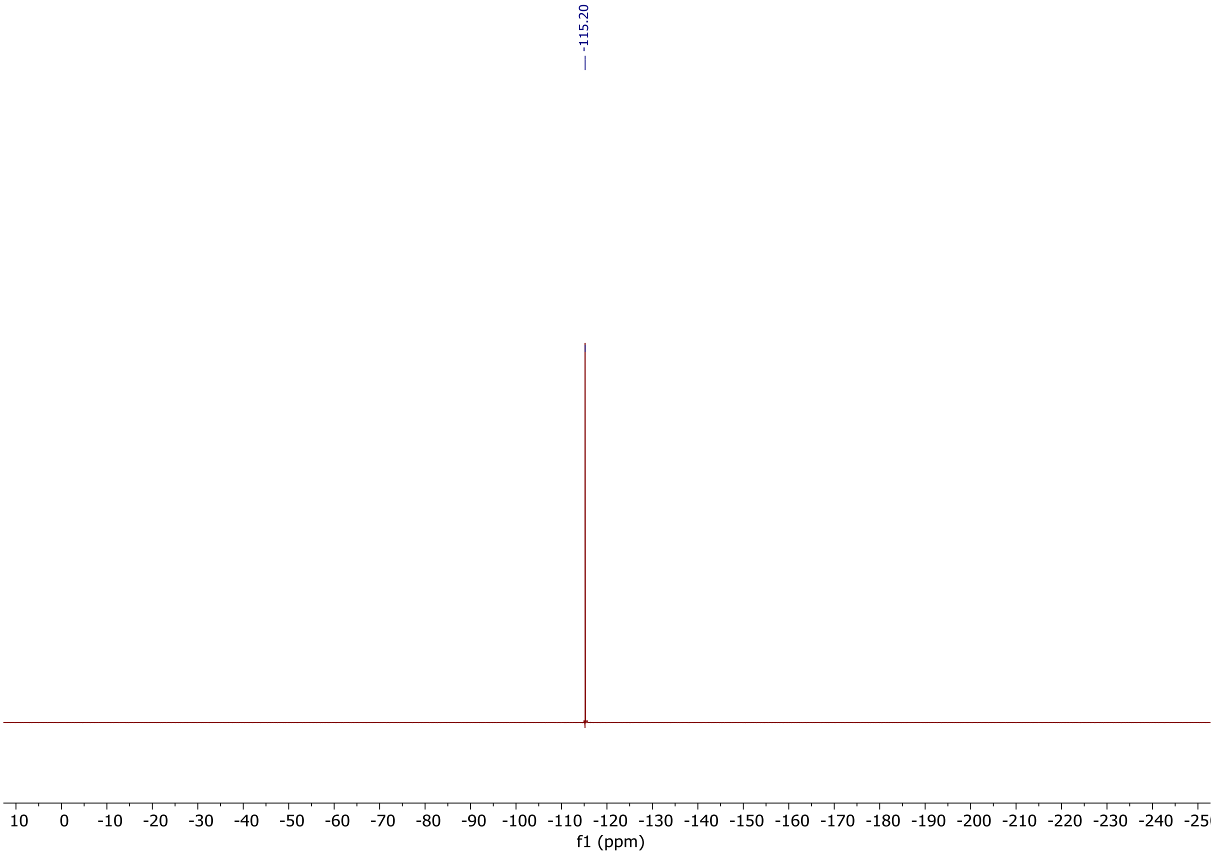
-115.20
10 0 -10 -20 -30 -40 -50 -60 -70 -80 -90 -100 -110 -120 -130 -140 -150 -160 -170 -180 -190 -200 -210 -220 -230 -240
f1 (ppm)

1,1-Diphenylprop-2-en-1-ol (**1b**)

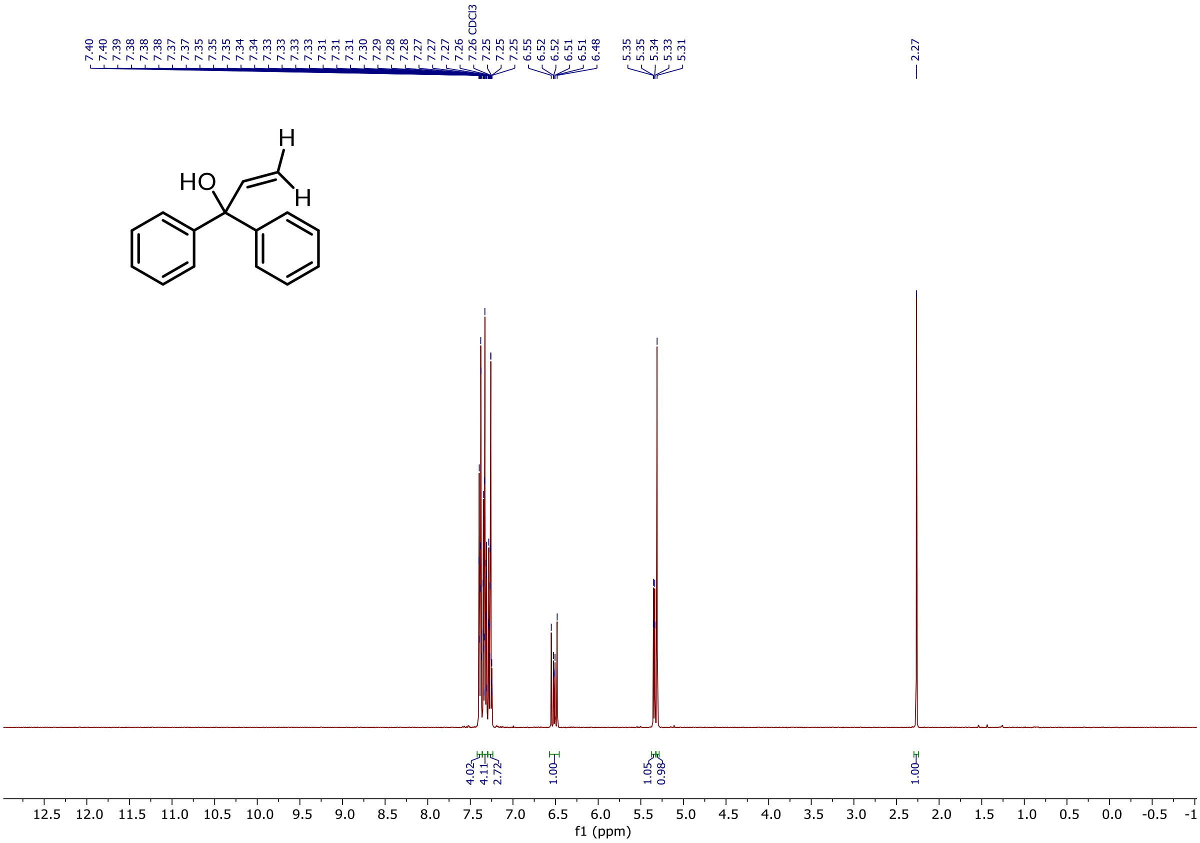

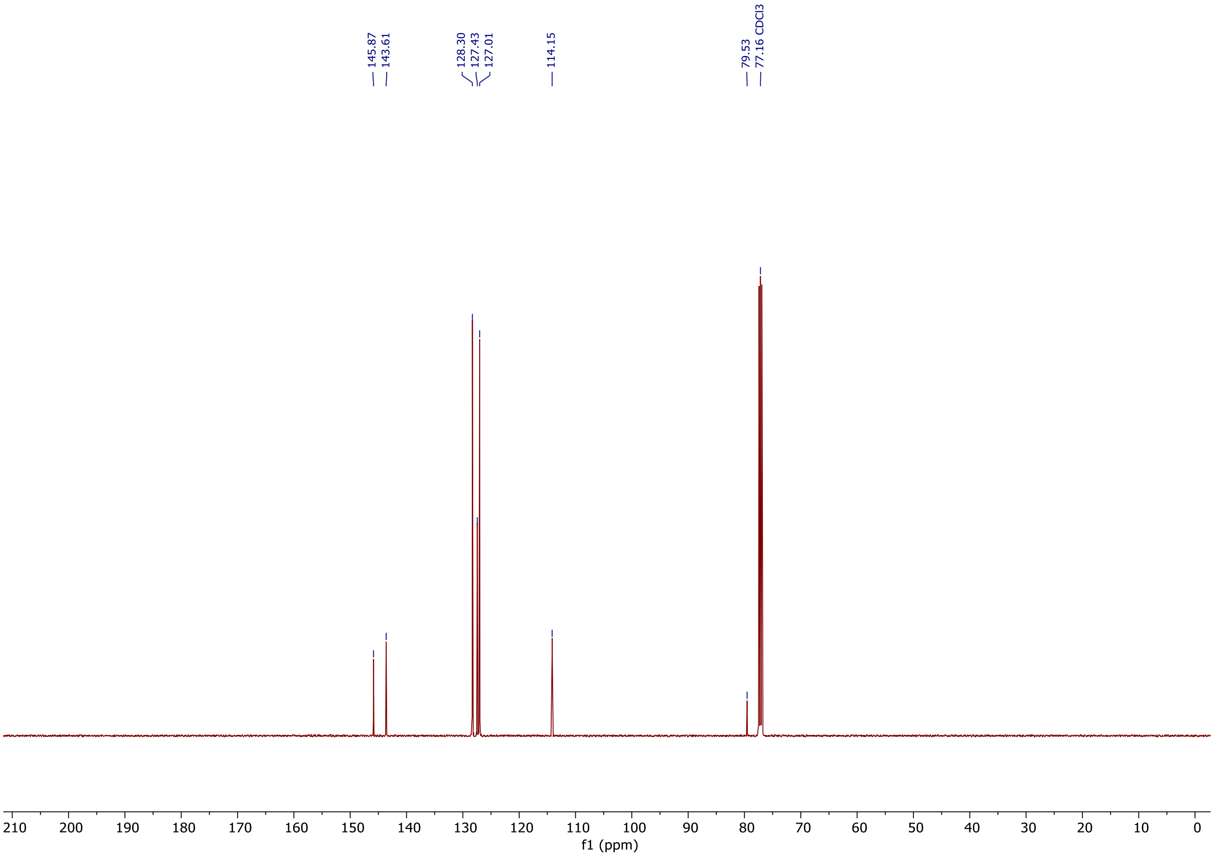

1,1-Di-*p*-tolylprop-2-en-1-ol (**1c**)

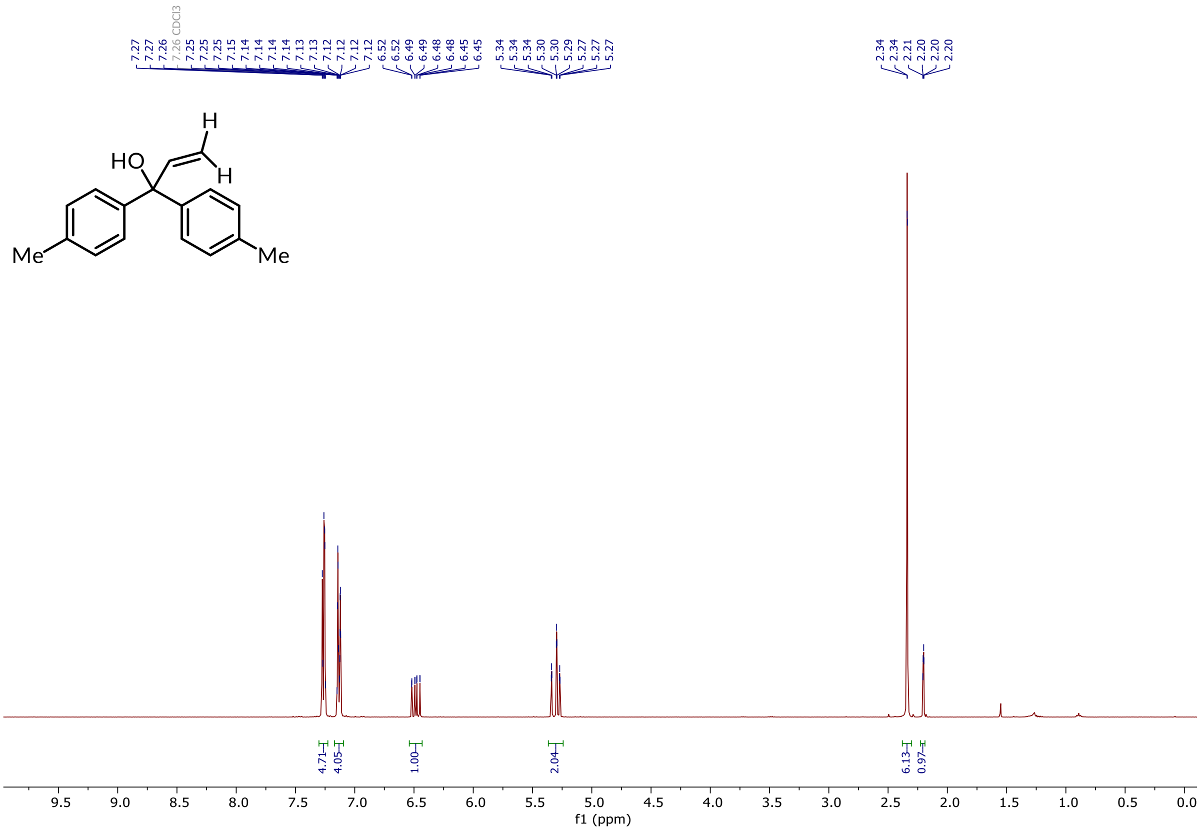

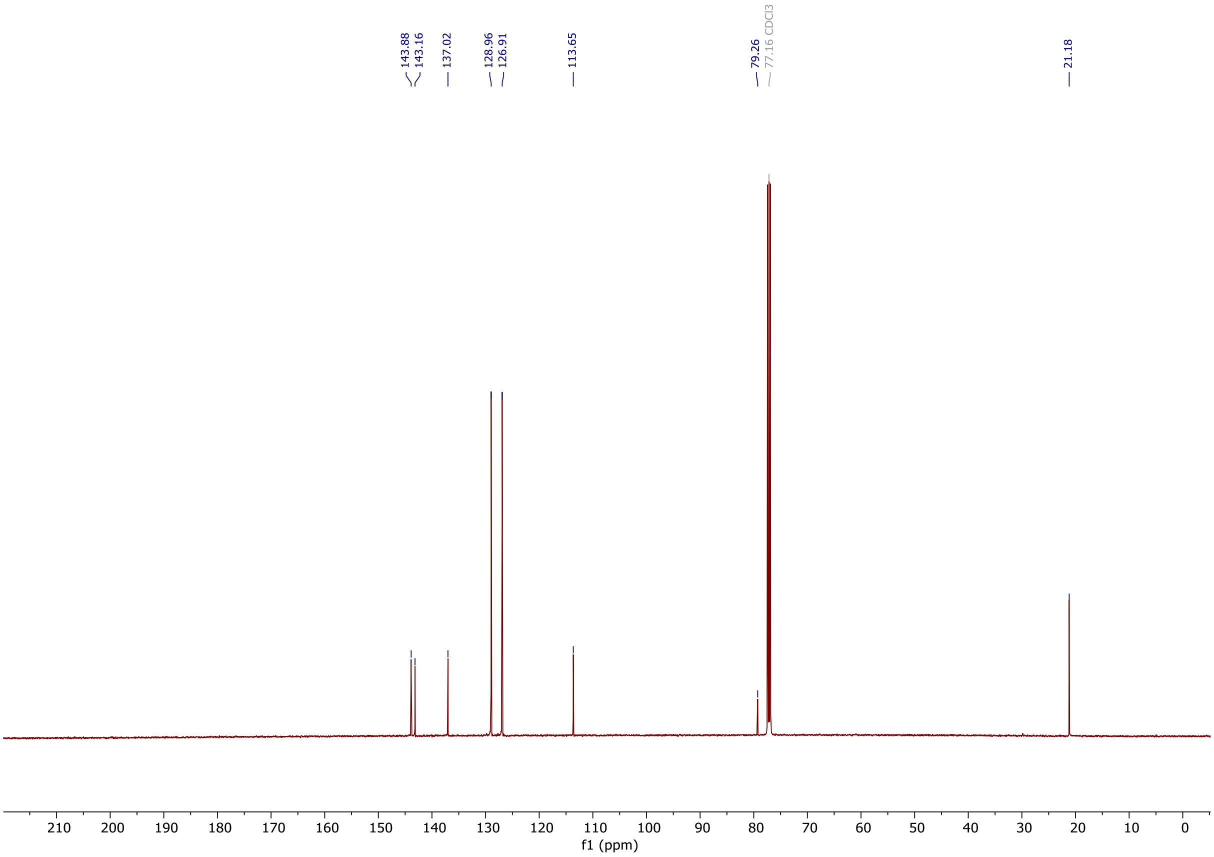

1,1-Bis(4-isopropylphenyl)prop-2-en-1-ol (**1d**)

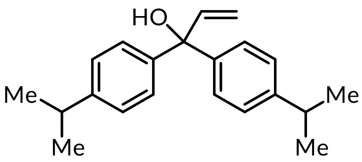

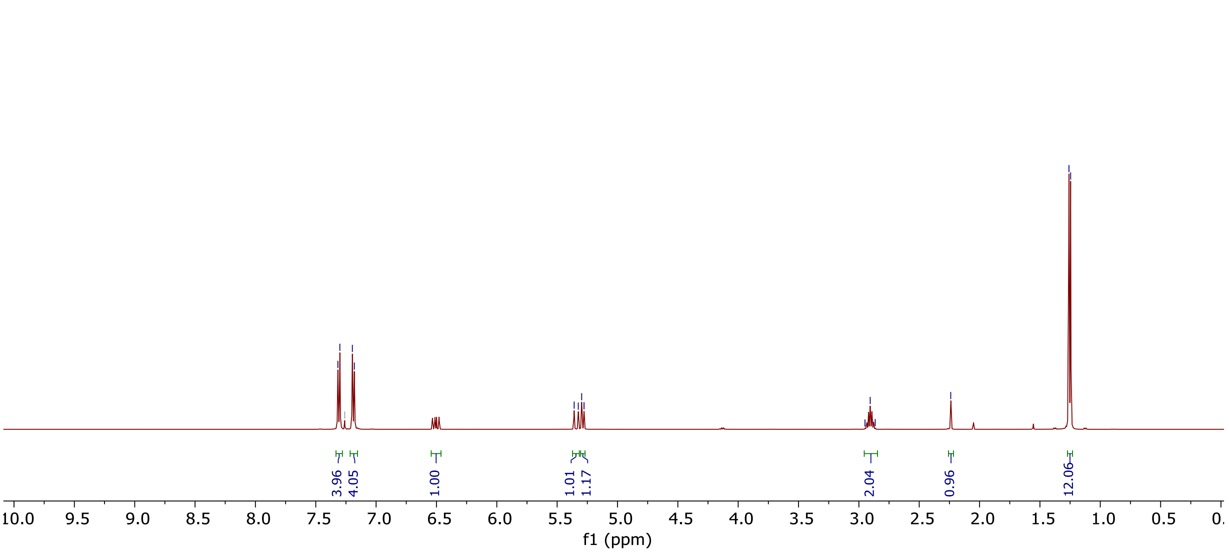

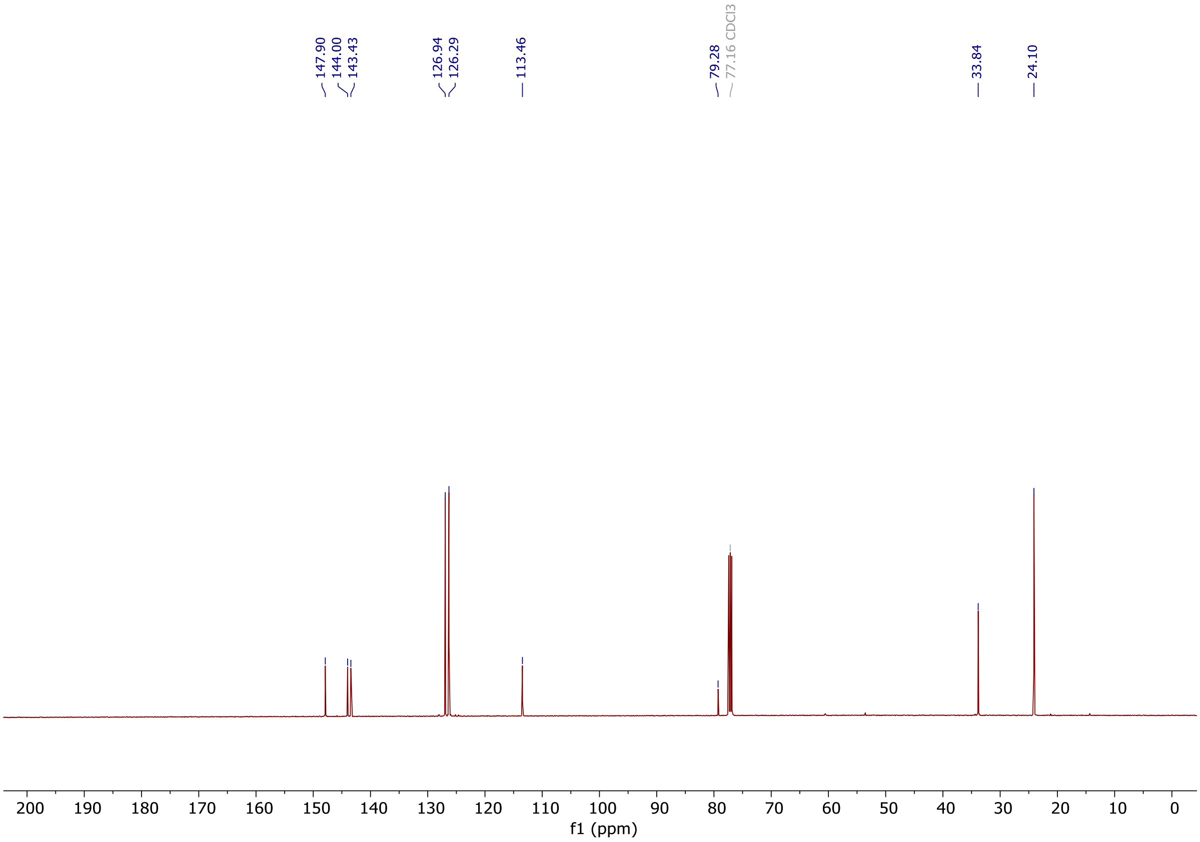

1,1-Bis(4-(*tert*-butyl)phenyl)prop-2-en-1-ol (**1e**)

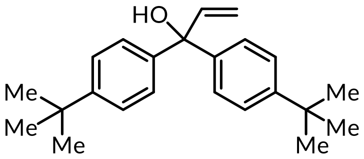

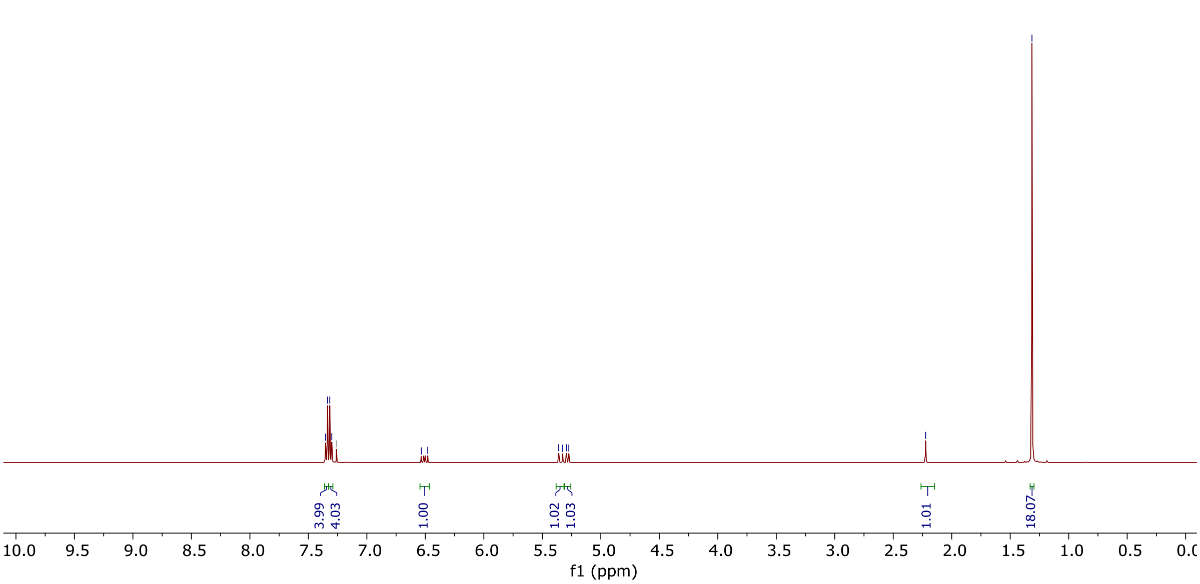

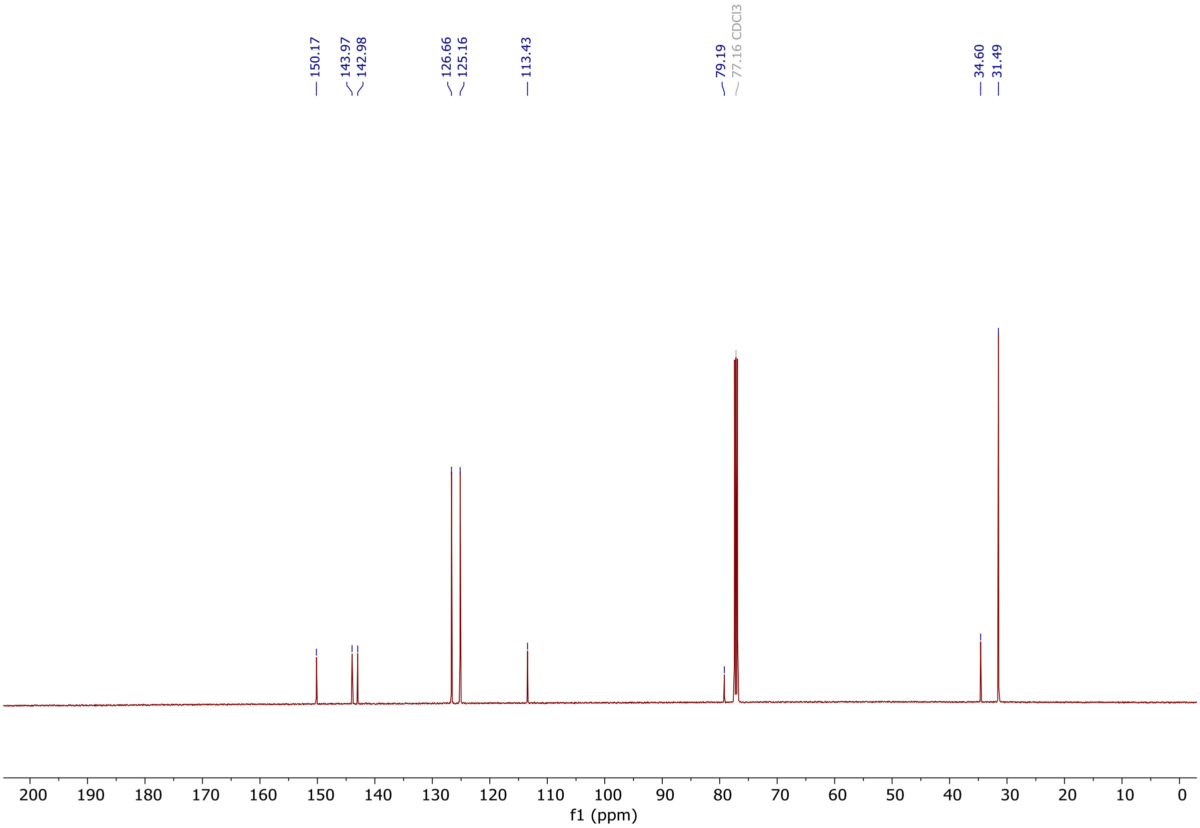

1,1-Di([1,1′-biphenyl]-4-yl)prop-2-en-1-ol (**1f**)

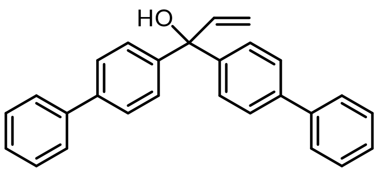

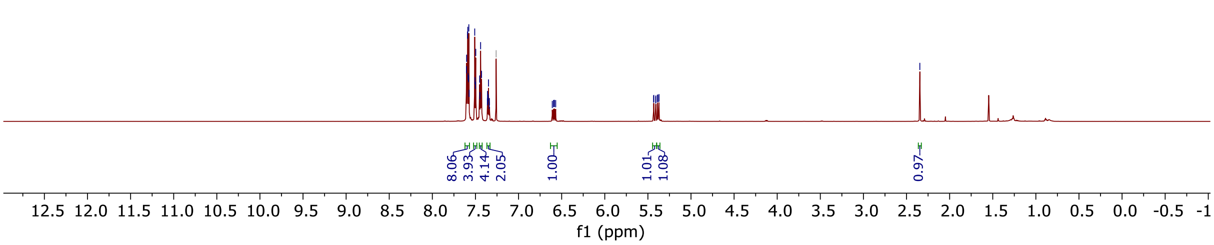

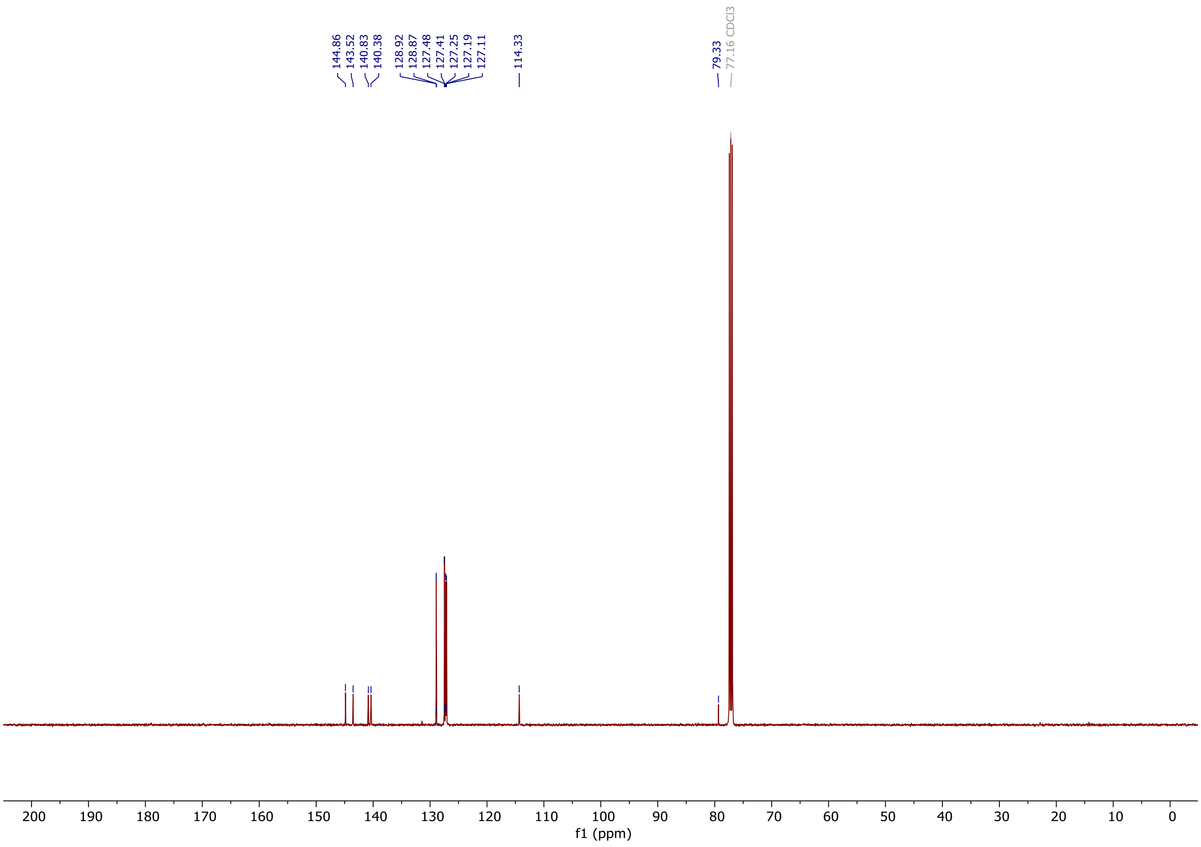

1,1-Bis(4-bromophenyl)prop-2-en-1-ol (**1g**)

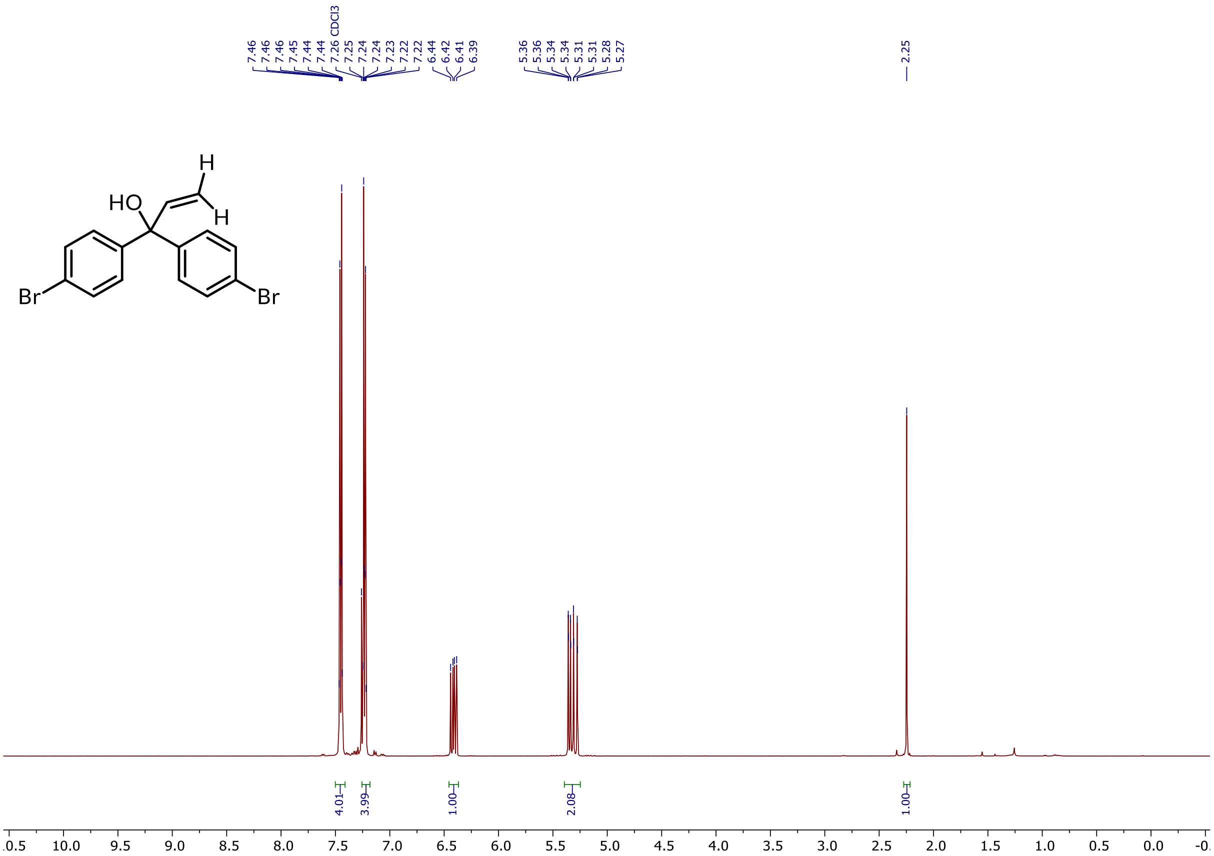

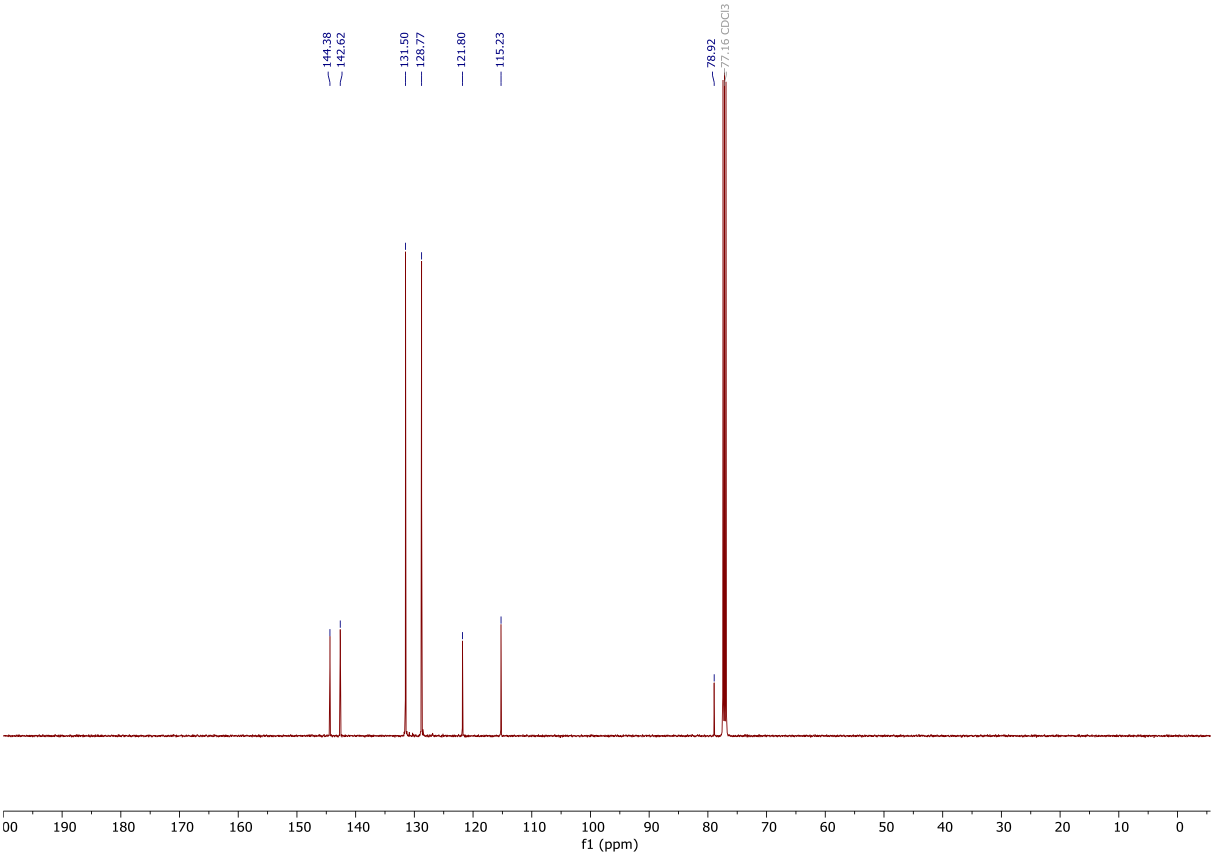

1,1-Bis(4-chlorophenyl)prop-2-en-1-ol (**1h**)

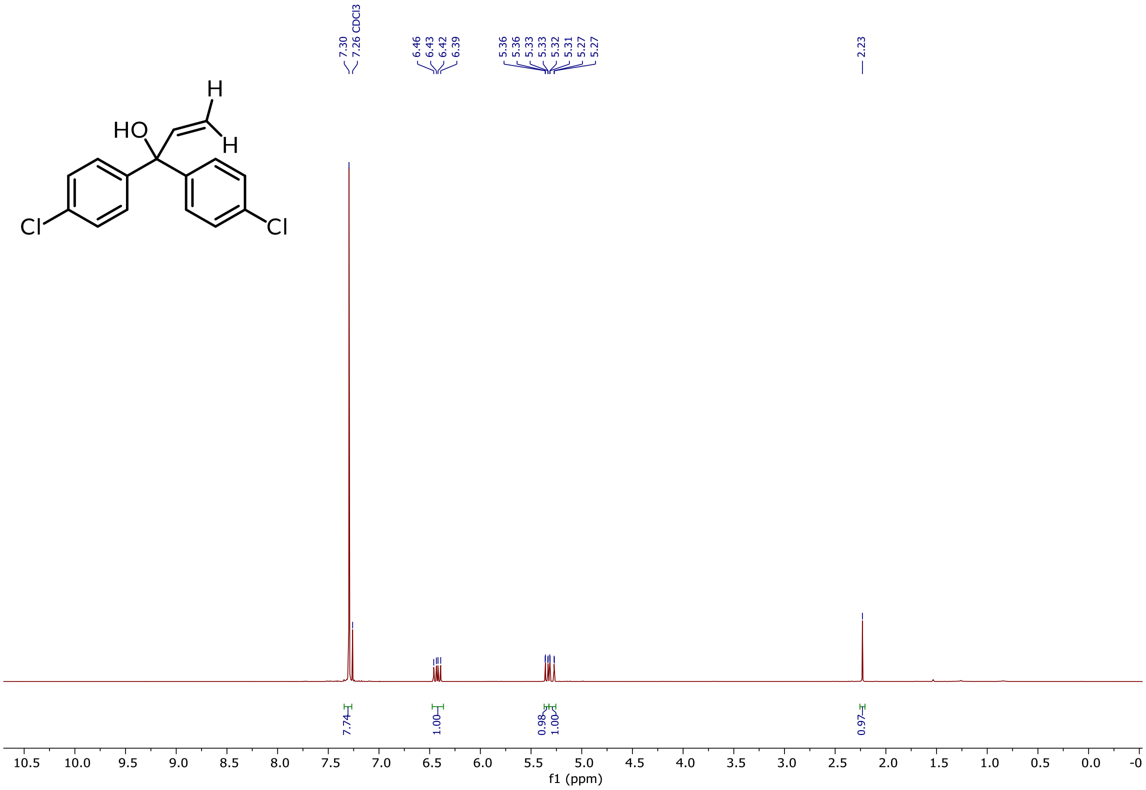

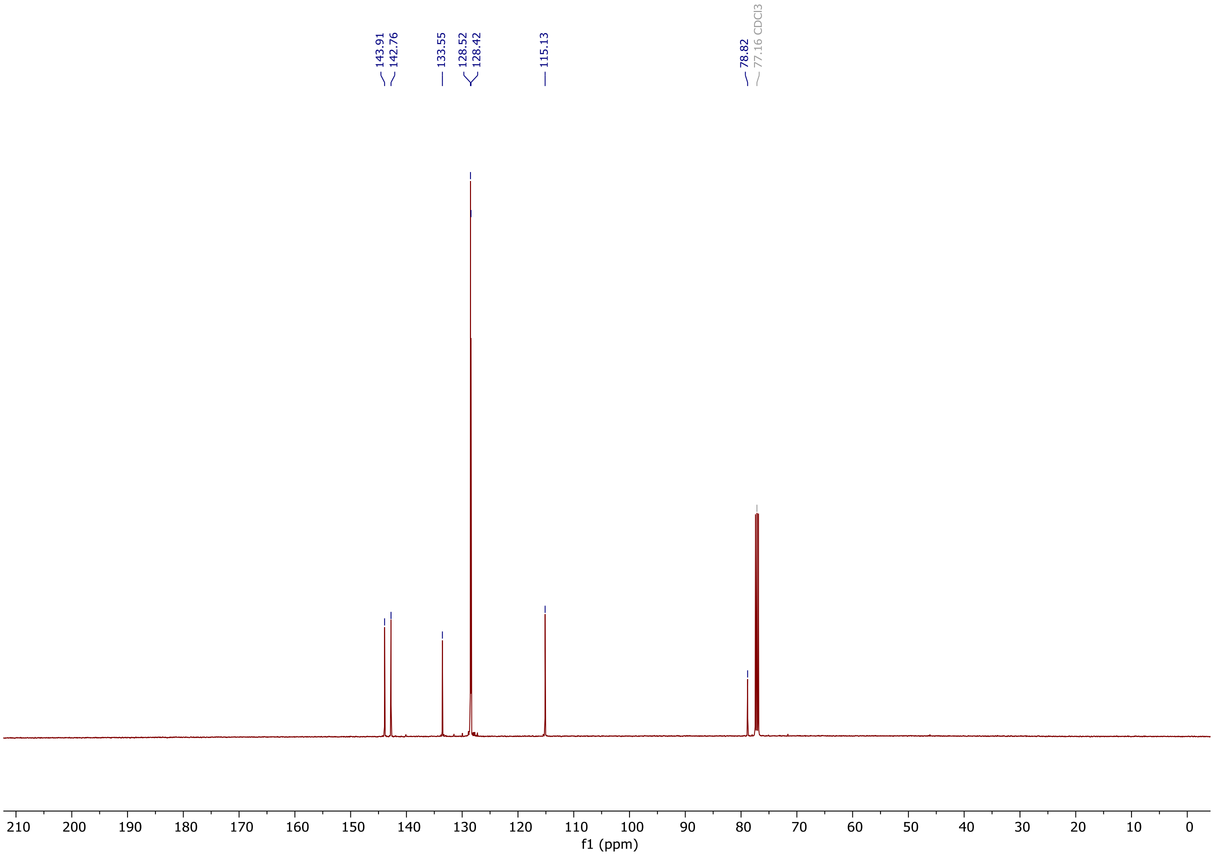

1,1-Bis(4-methoxyphenyl)prop-2-en-1-ol (**1i**)

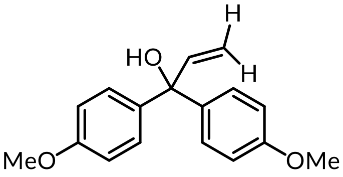

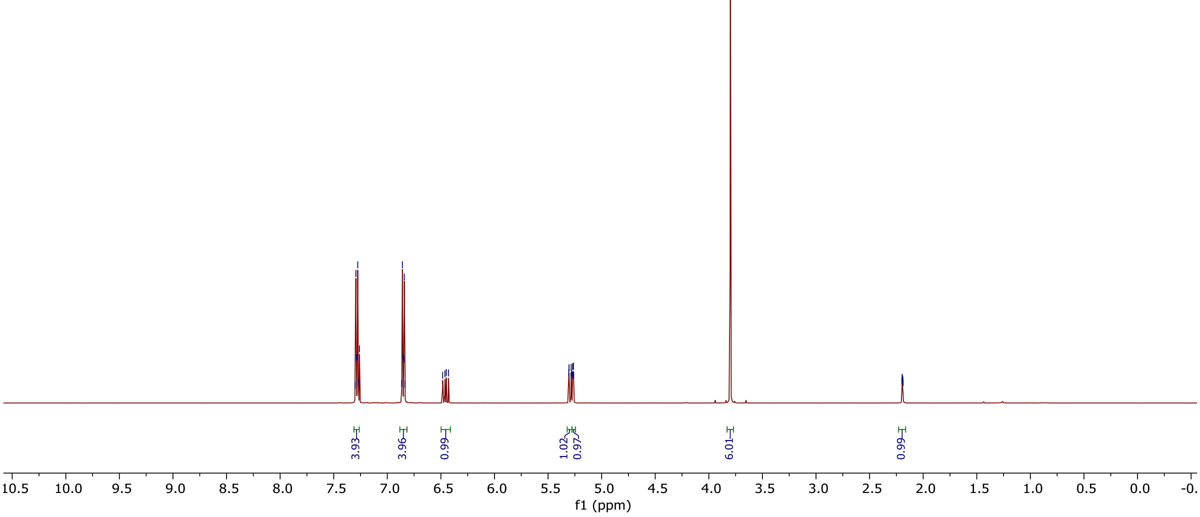

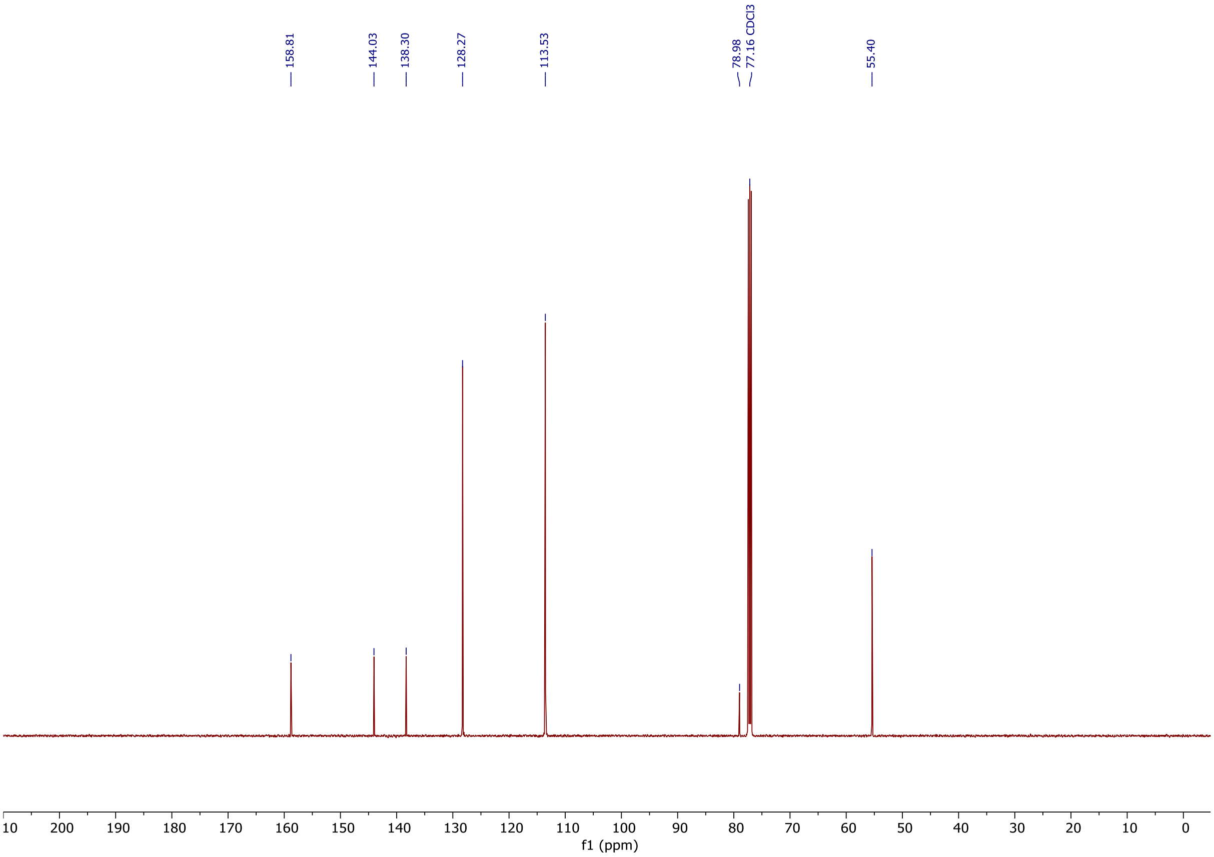

1,1-Bis(4-phenoxyphenyl)prop-2-en-1-ol (**1j**)

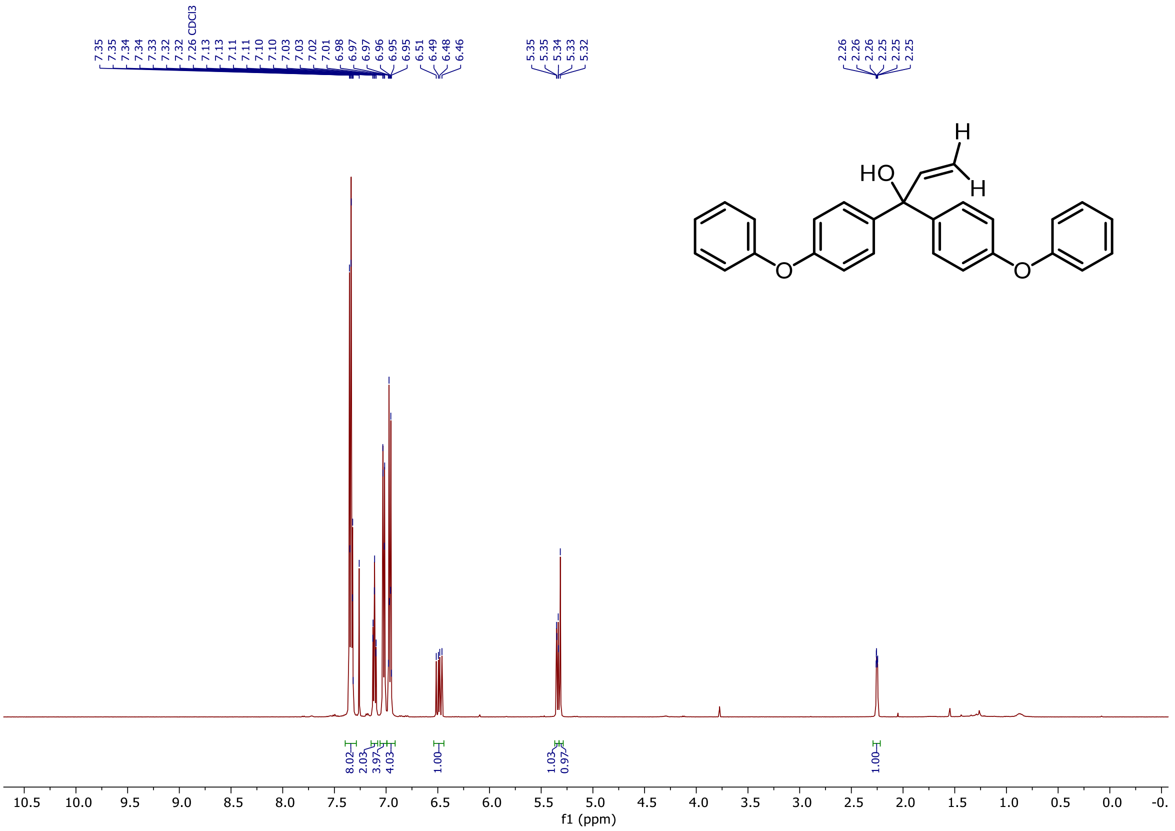

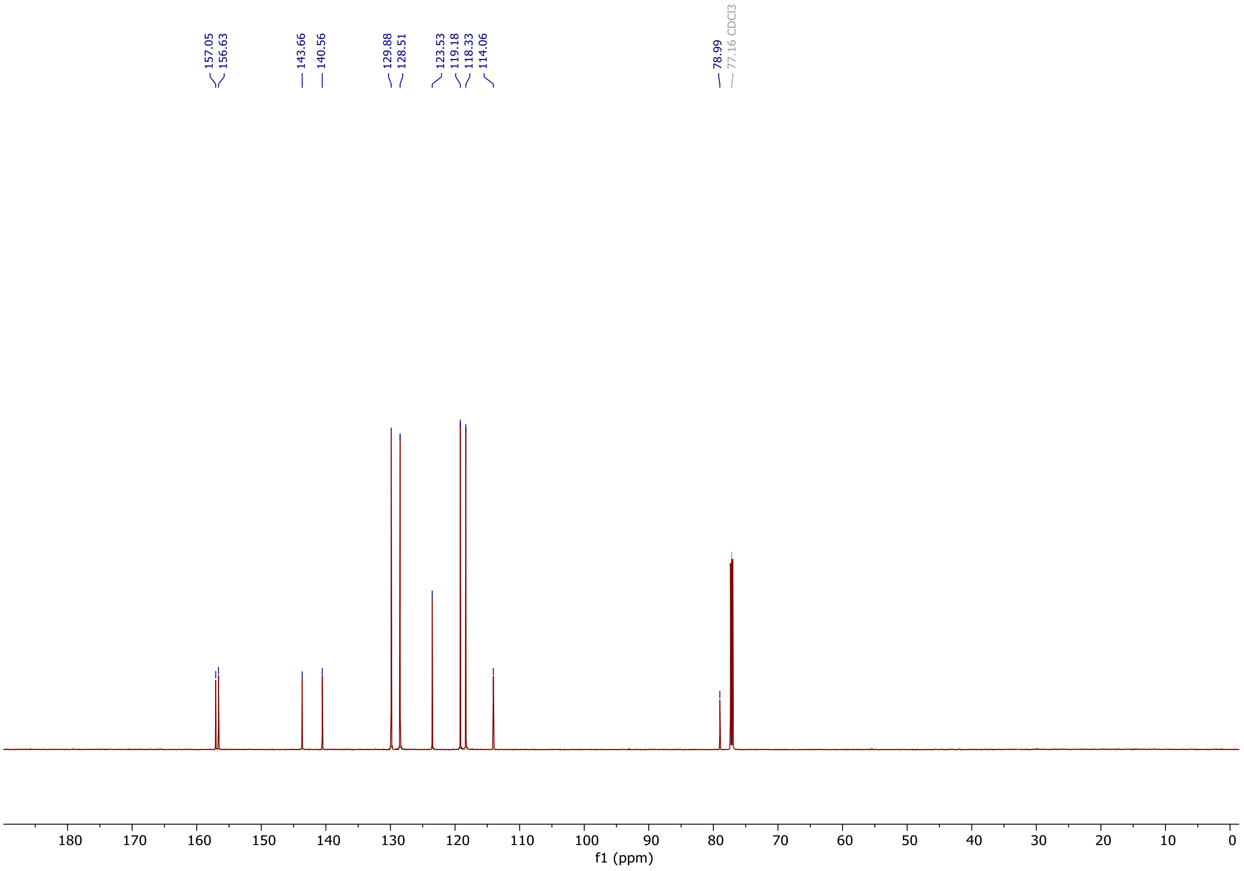

1,1-Bis(4-(methylthio)phenyl)prop-2-en-1-ol (**1k**)

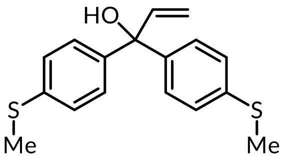

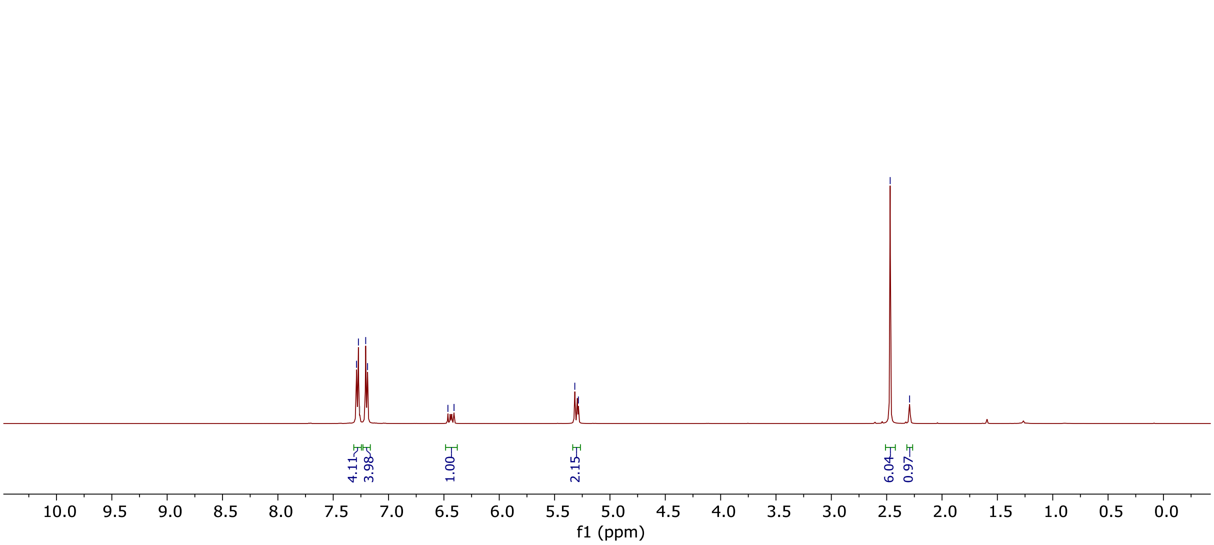

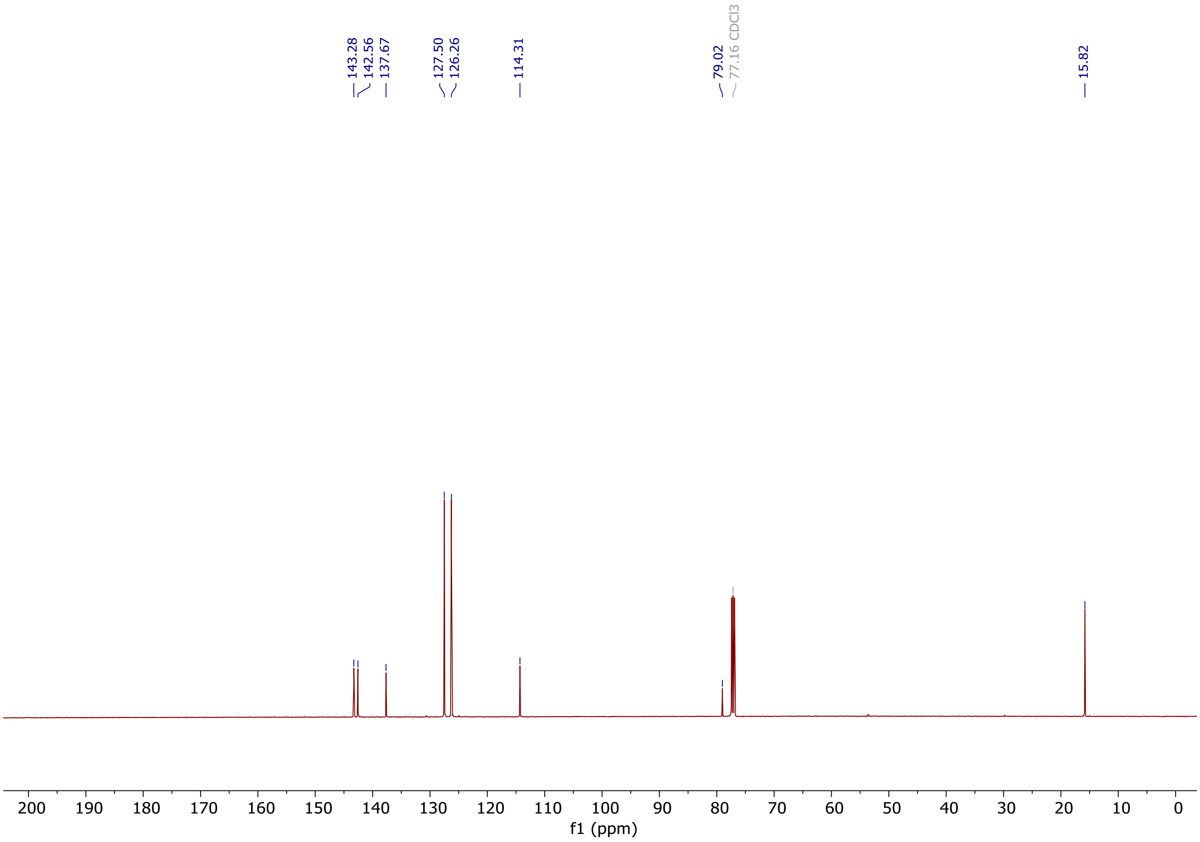

1,1-Bis(4-((trimethylsilyl)ethynyl)phenyl)prop-2-en-1-ol (**1l**)

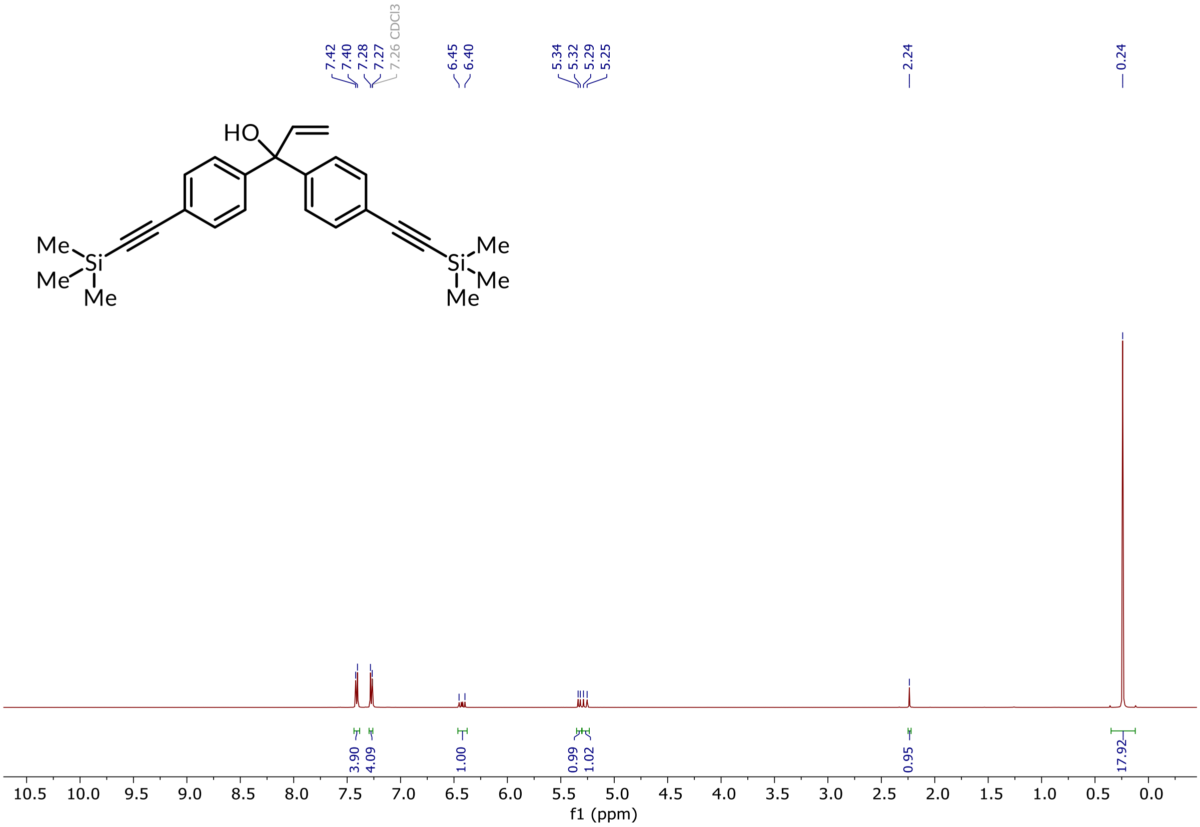

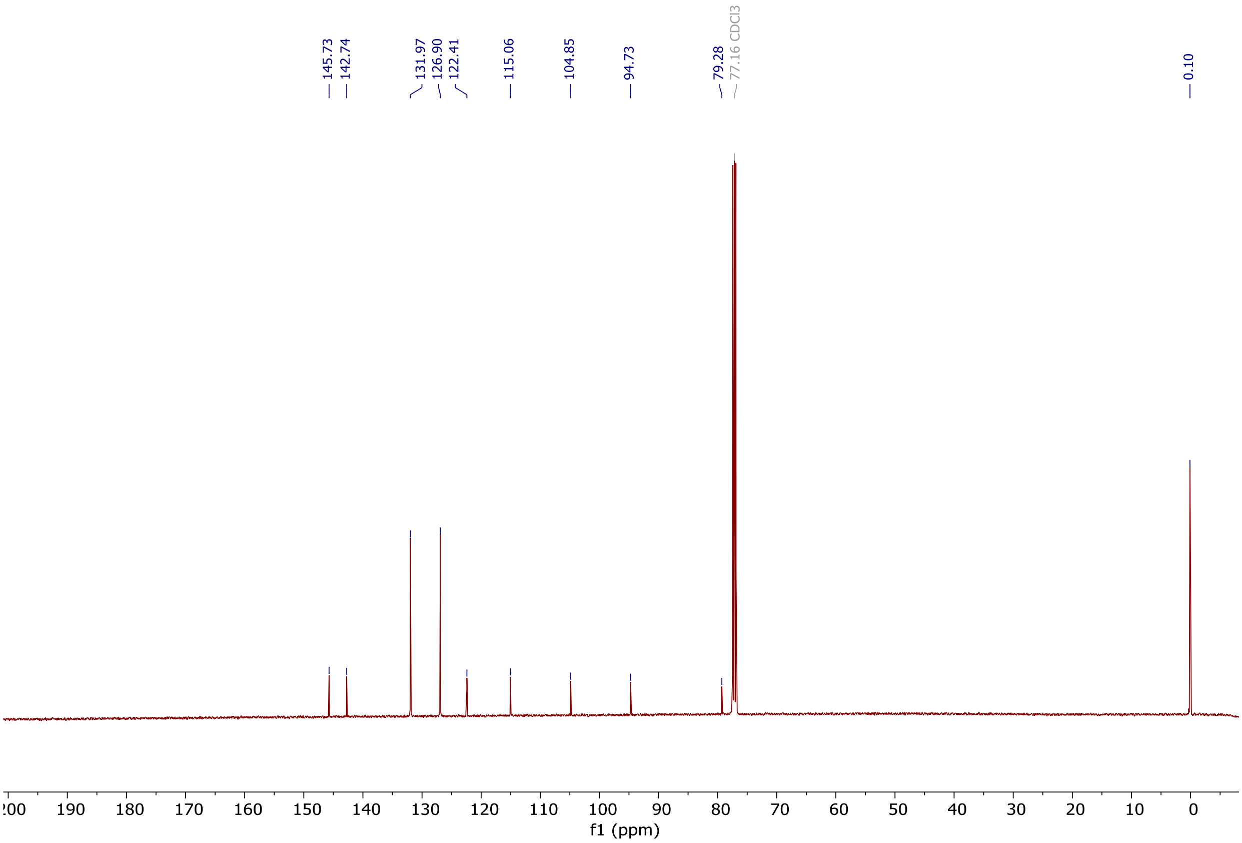

1,1-Bis(4-(trifluoromethyl)phenyl)prop-2-en-1-ol (**1m**)

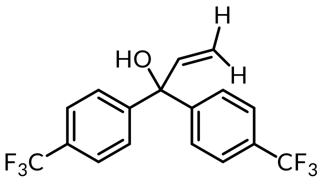

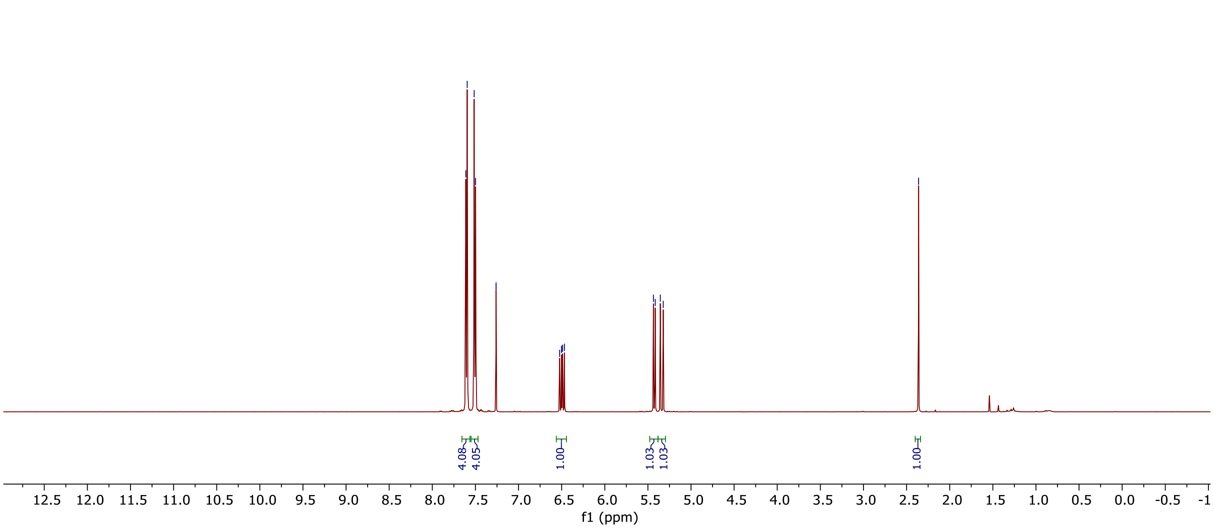

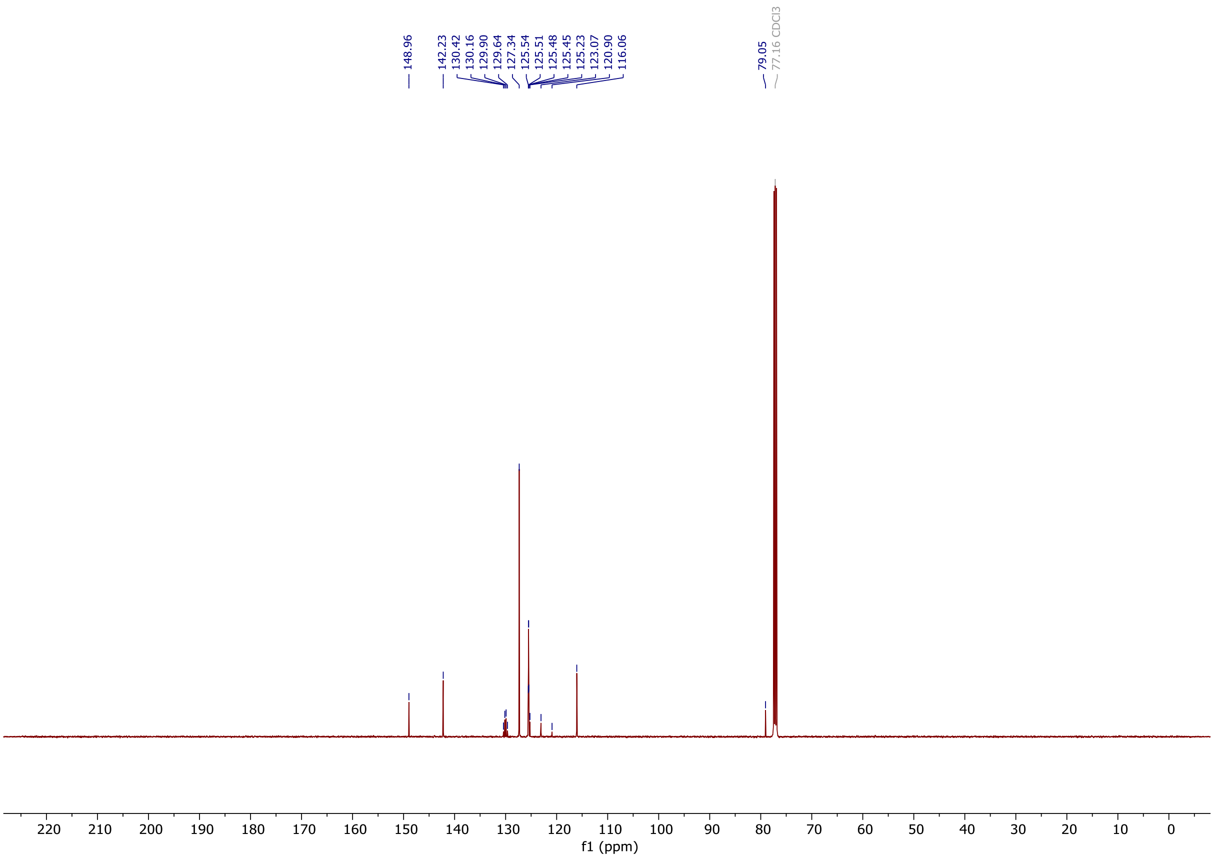

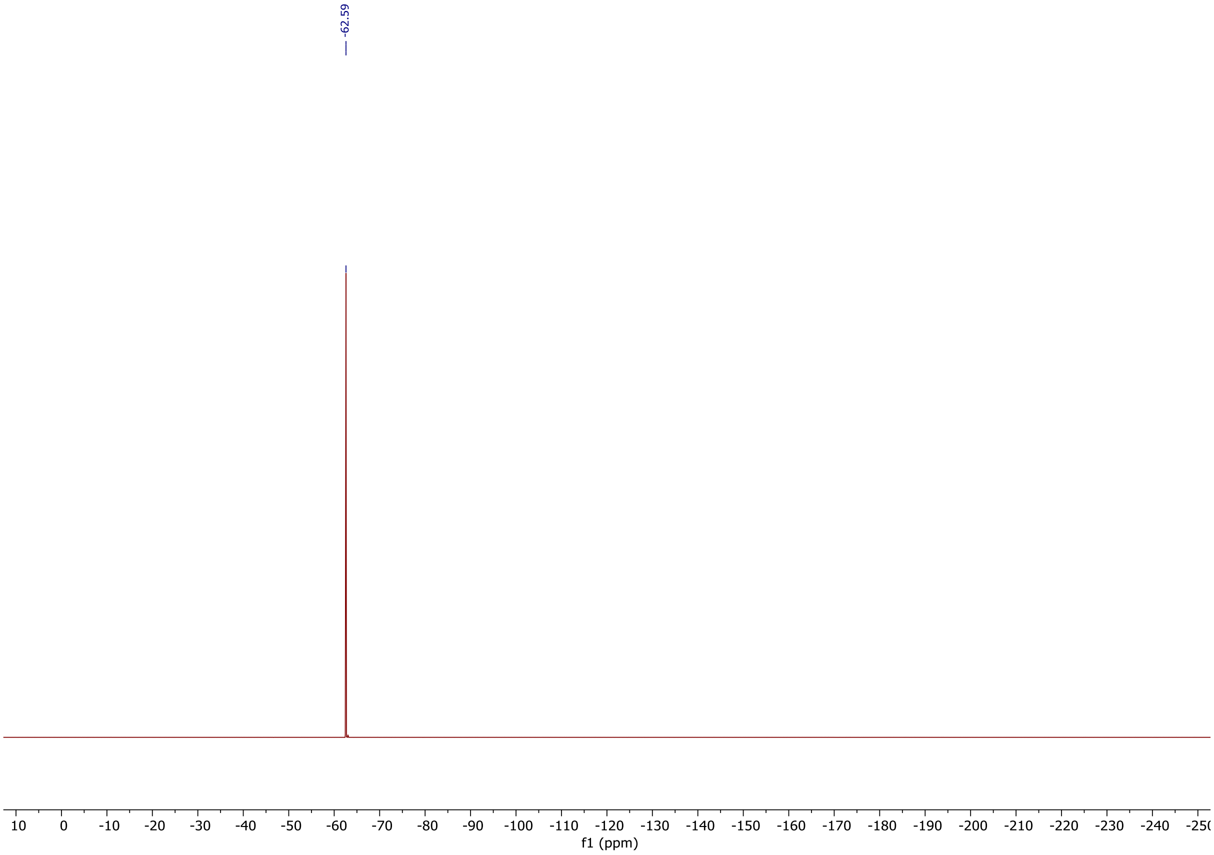

1,1-Bis(3-(trifluoromethyl)phenyl)prop-2-en-1-ol (**1n**)

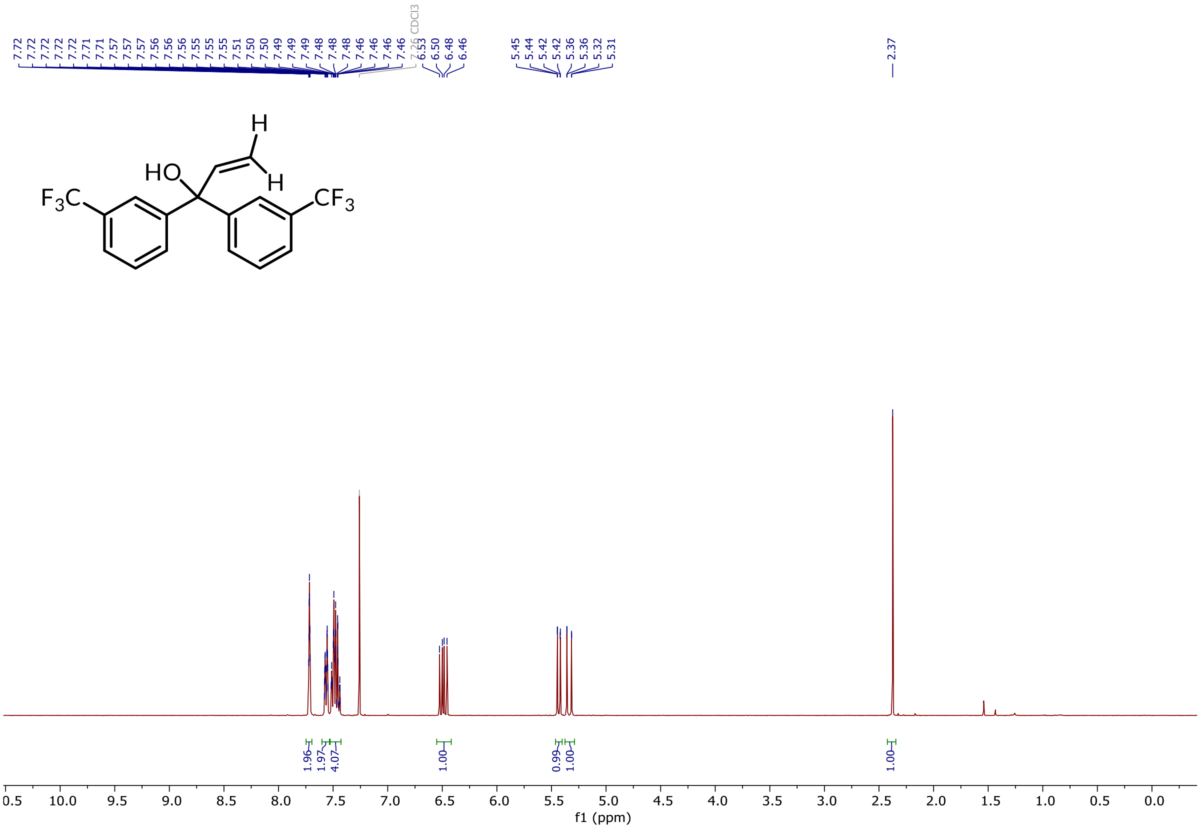

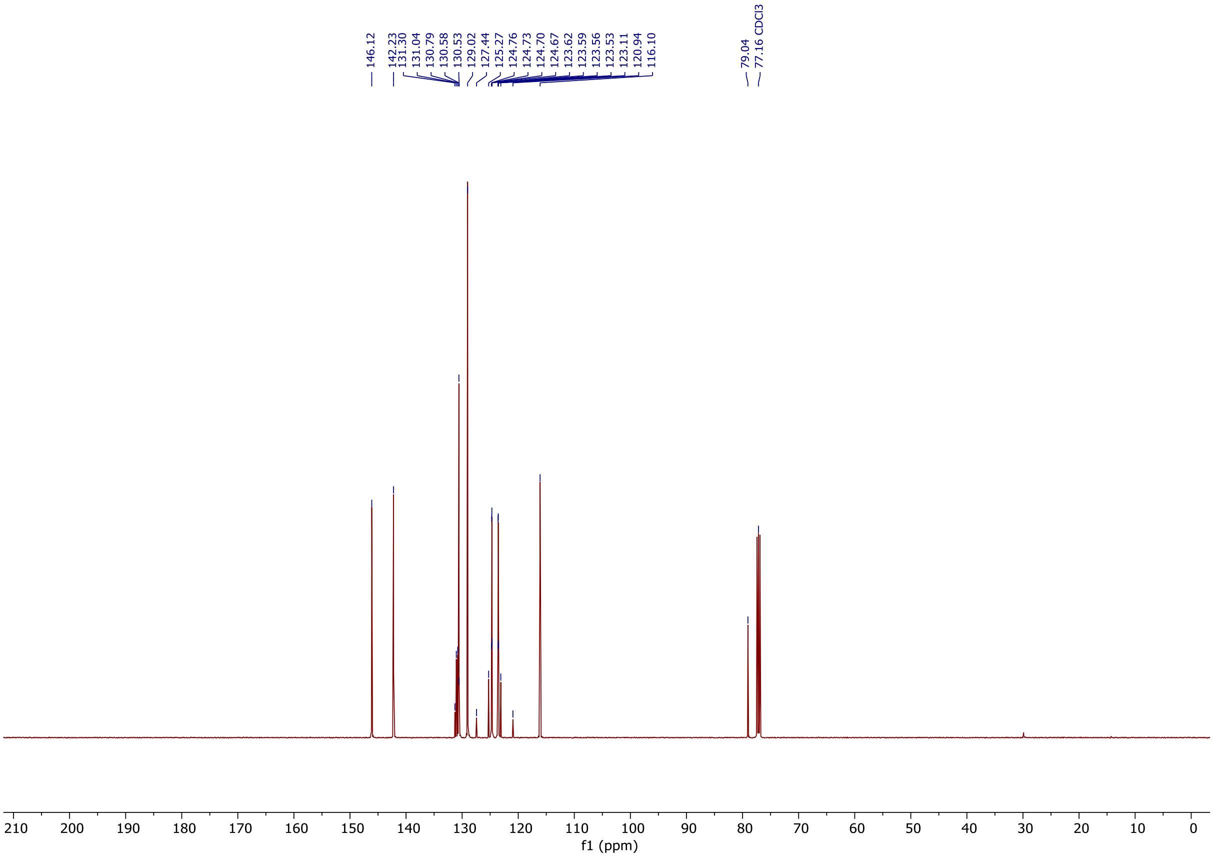
146.12
142.23
131.30
131.04
130.79
130.58
130.53
129.02
127.44
125.27
124.76
124.73
124.70
124.67
123.62
123.59
123.56
123.53
123.11
120.94
116.10
79.04
77.16 CDCl3
210 200 190 180 170 160 150 140 130 120 110 100 90 80 70 60 50 40 30 20 10 0
f1 (ppm)

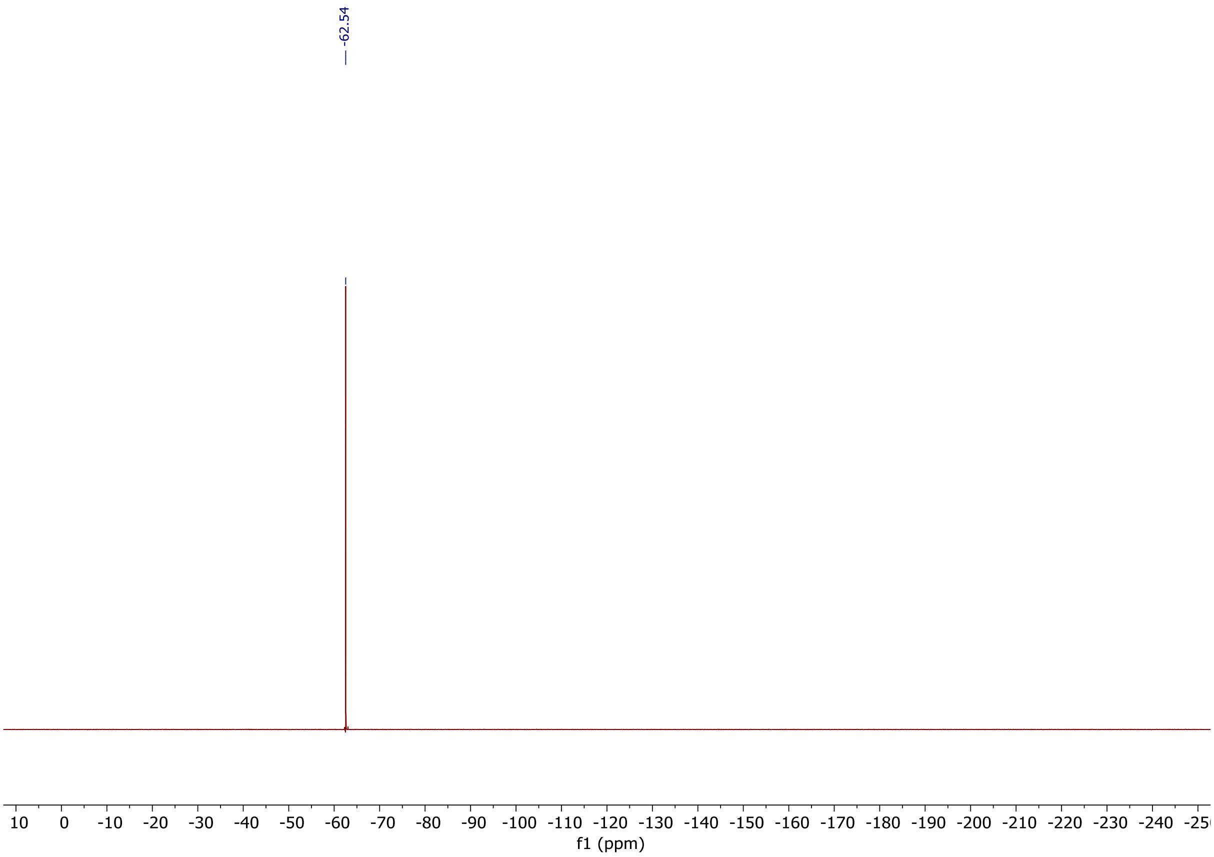
-62.54
10 0 -10 -20 -30 -40 -50 -60 -70 -80 -90 -100 -110 -120 -130 -140 -150 -160 -170 -180 -190 -200 -210 -220 -230 -240
f1 (ppm)

1,1-Bis(3-chlorophenyl)prop-2-en-1-ol (**1o**)

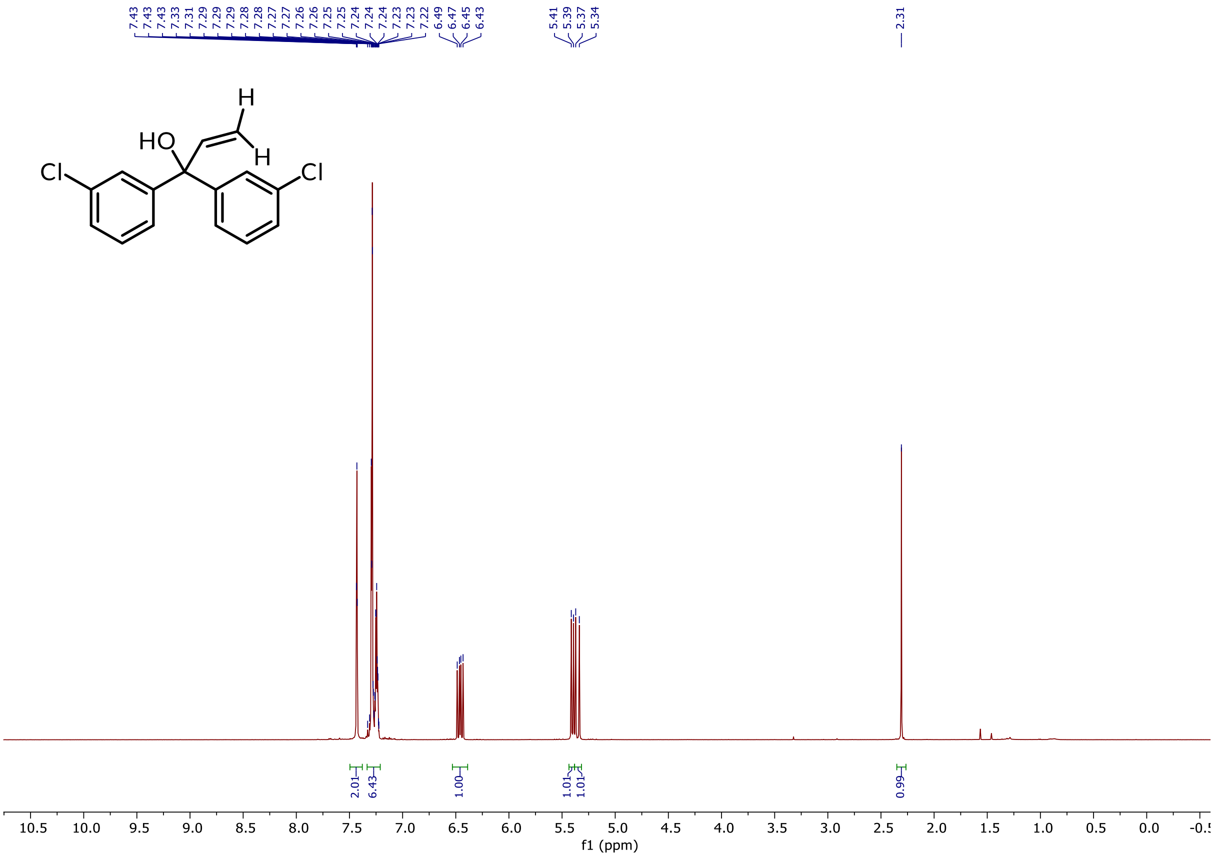

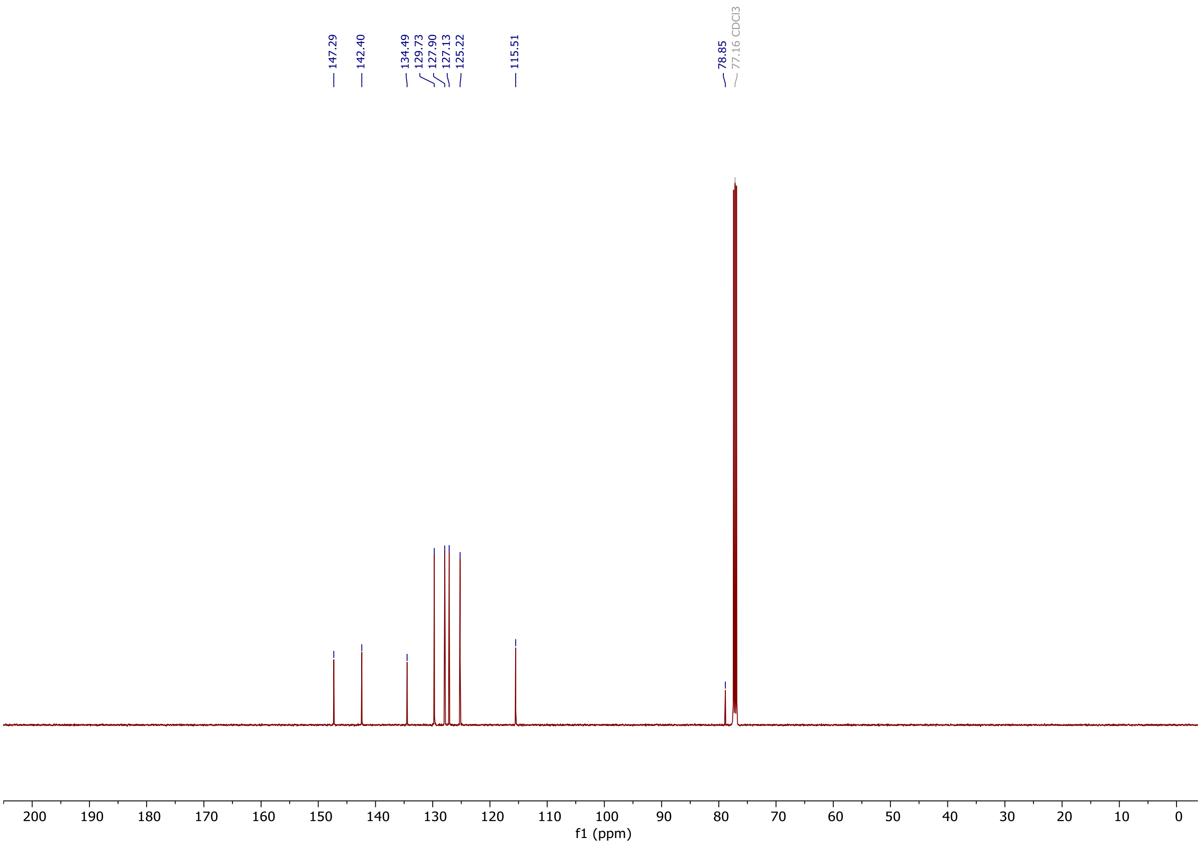

1,1-Bis(3-fluorophenyl)prop-2-en-1-ol (**1p**)

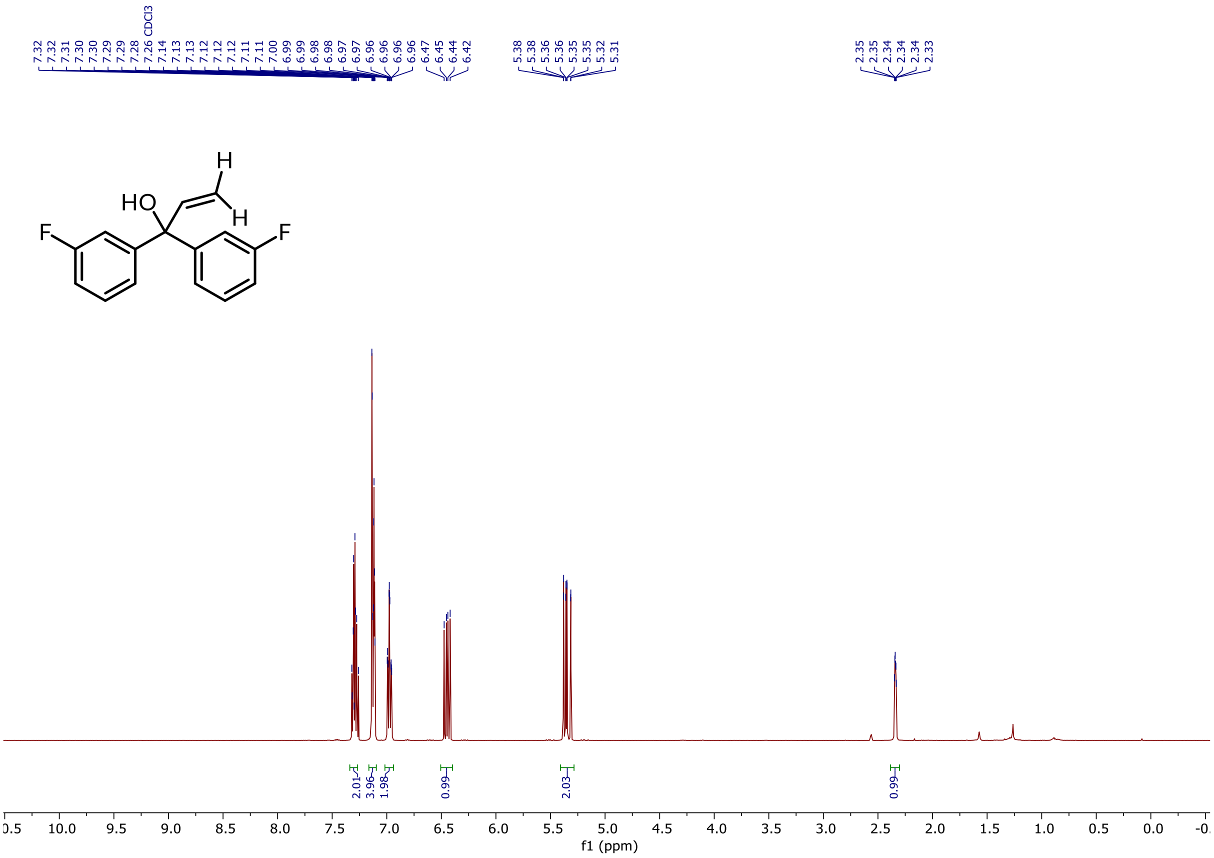

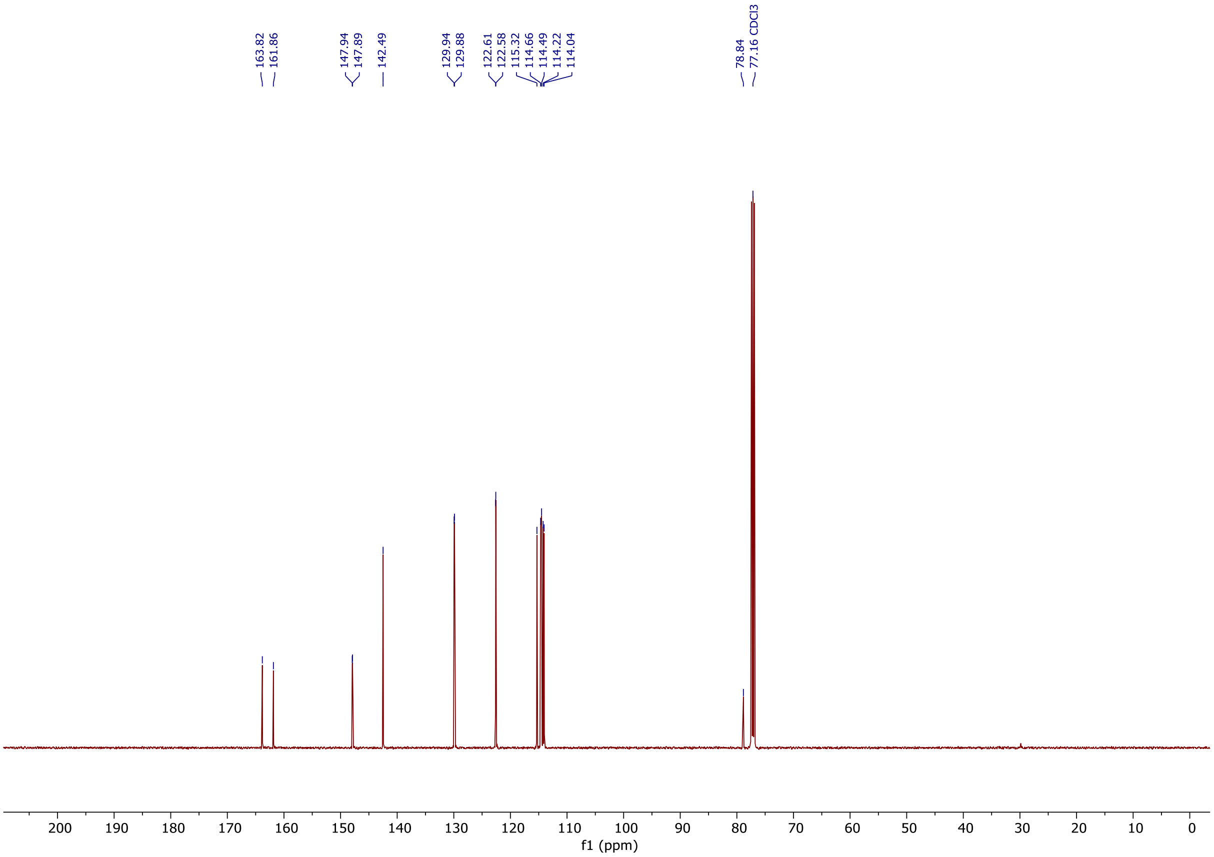

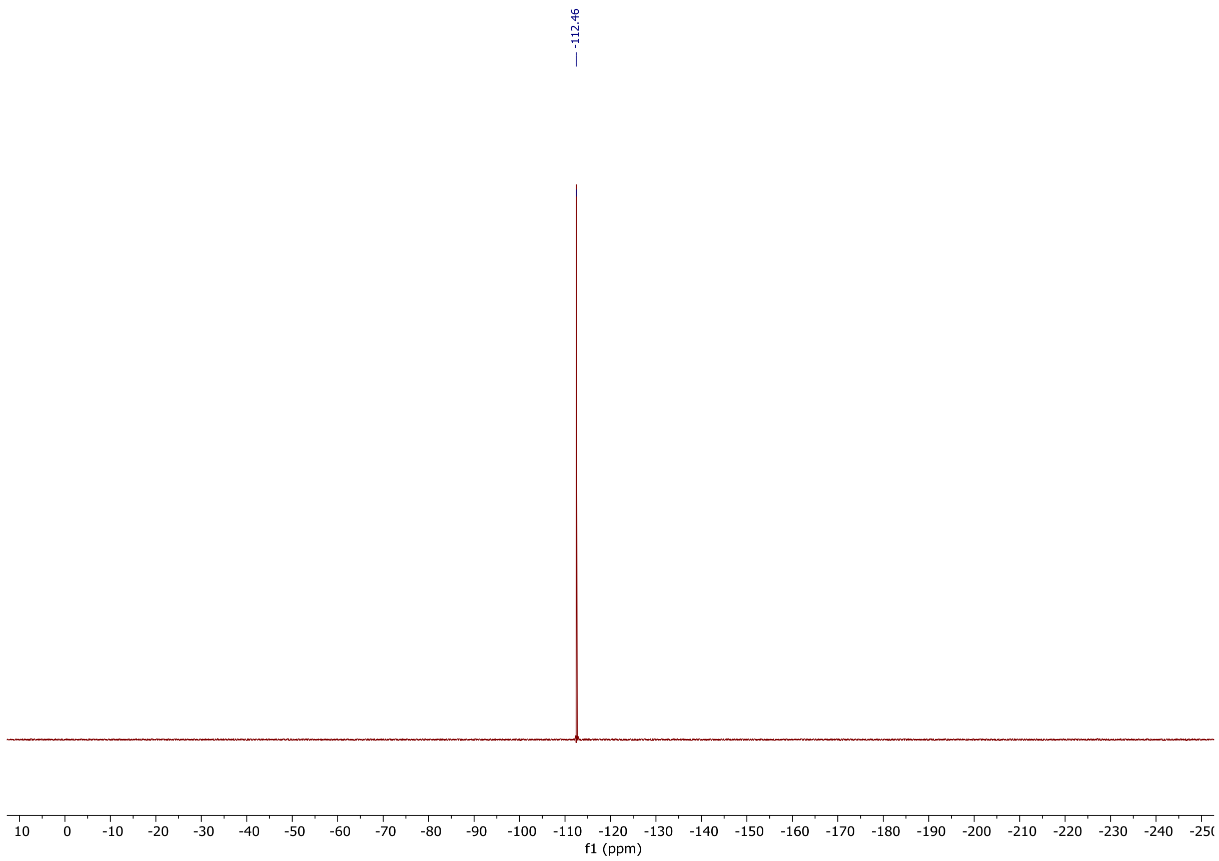
-112.46
10 0 -10 -20 -30 -40 -50 -60 -70 -80 -90 -100 -110 -120 -130 -140 -150 -160 -170 -180 -190 -200 -210 -220 -230 -240 -250
f1 (ppm)

1,1-di-*m*-tolylprop-2-en-1-ol (**1q**)

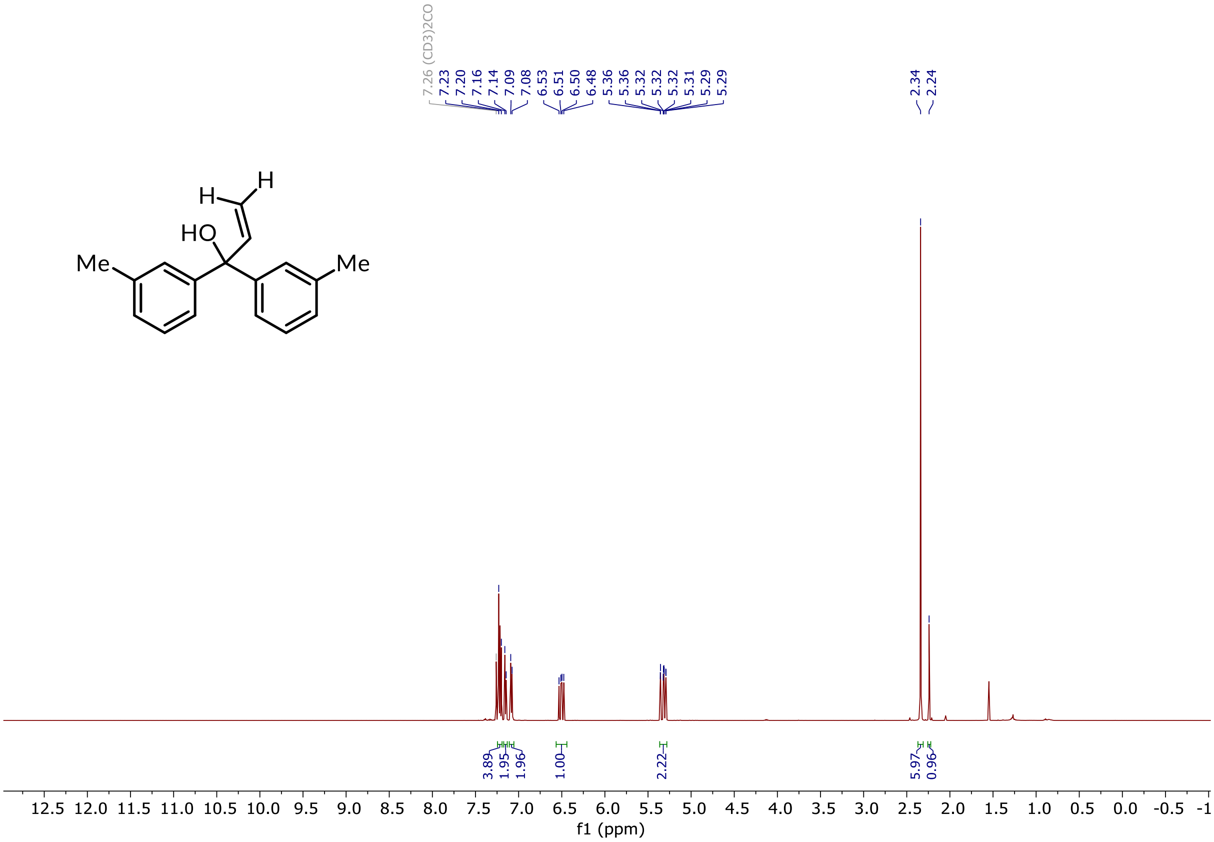

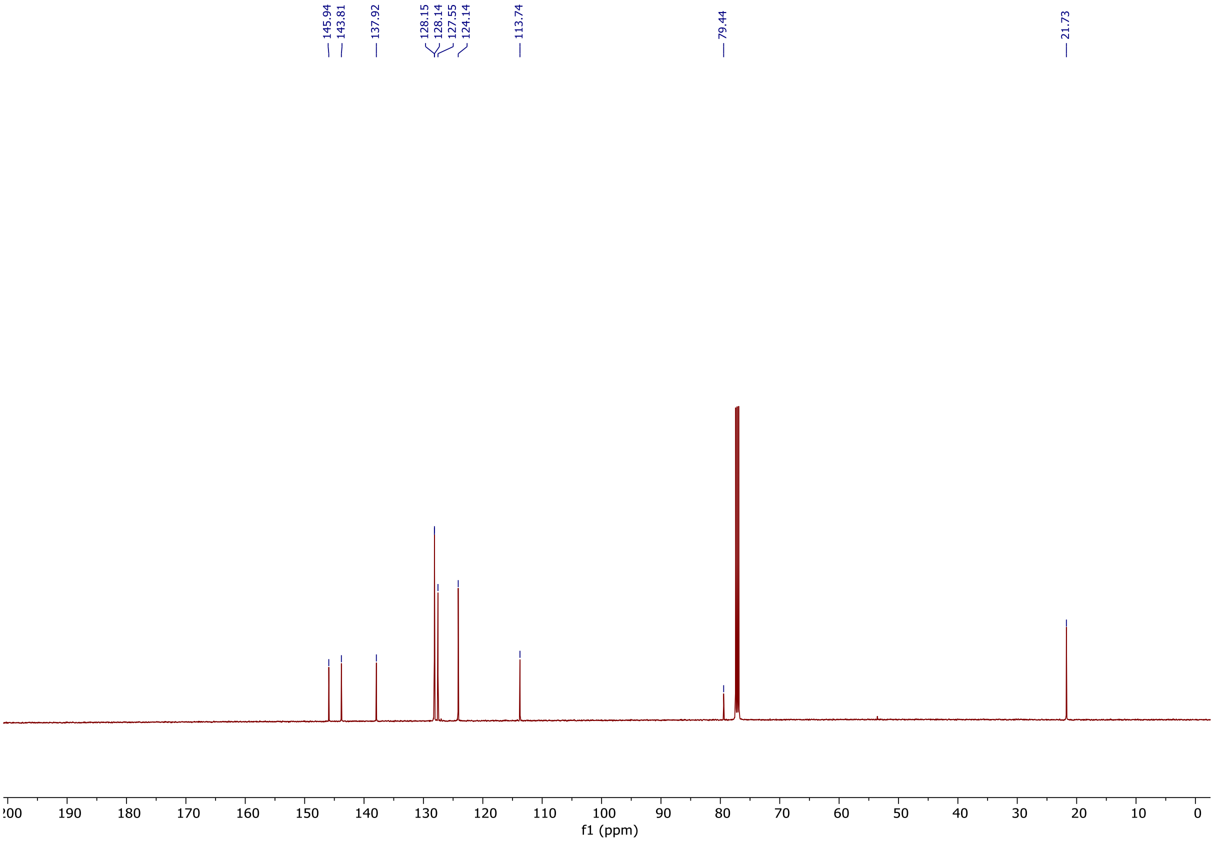

1,1-bis-(3-methoxyphenyl)prop-2-en-1-ol (**1r**)

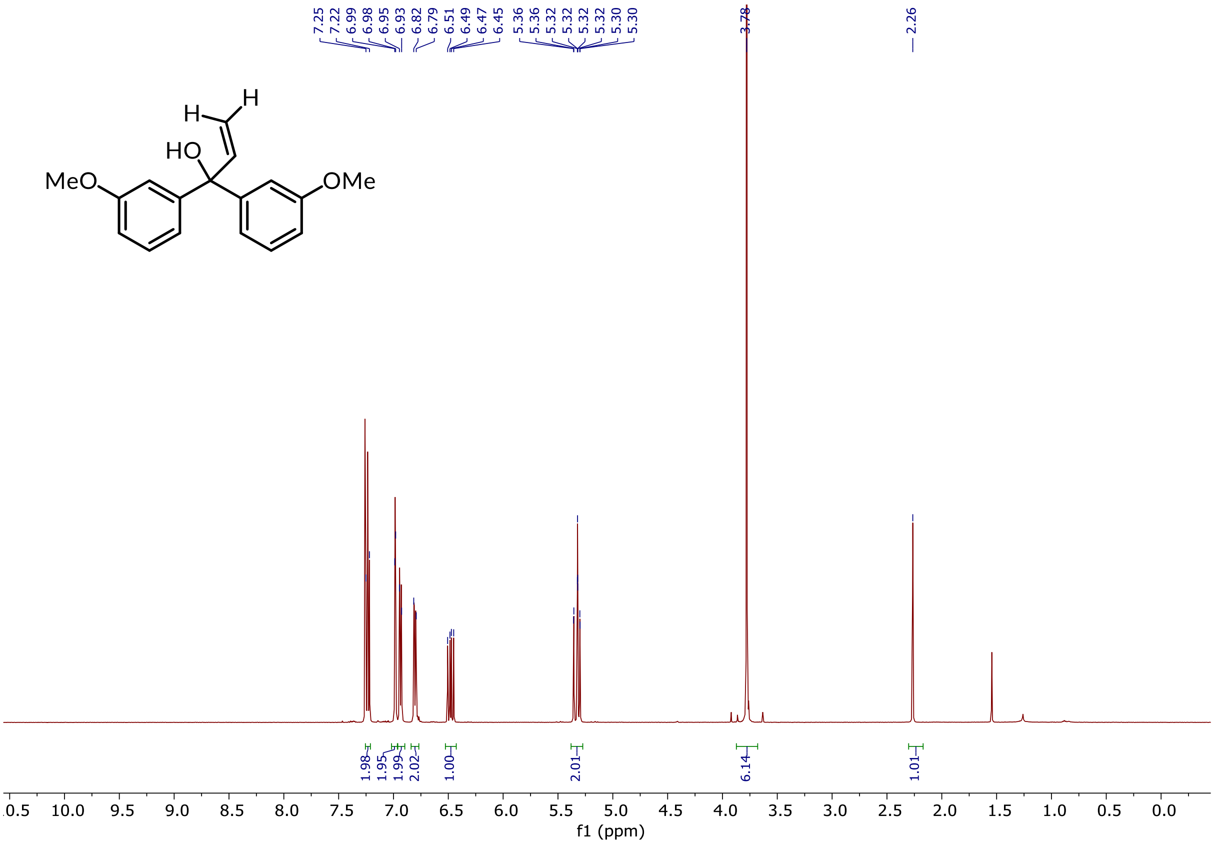

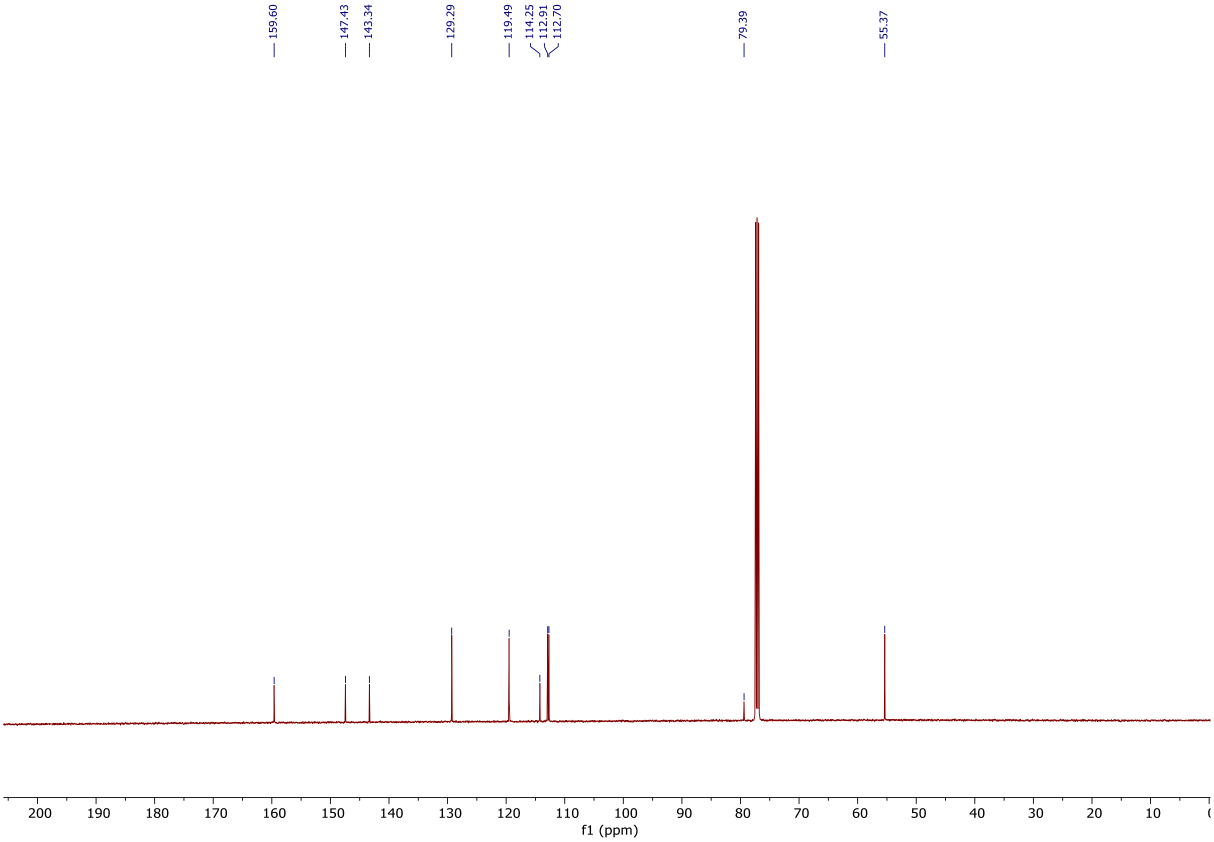

1,1-Bis(2-chlorophenyl)prop-2-en-1-ol (**1s**)

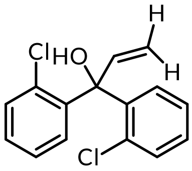

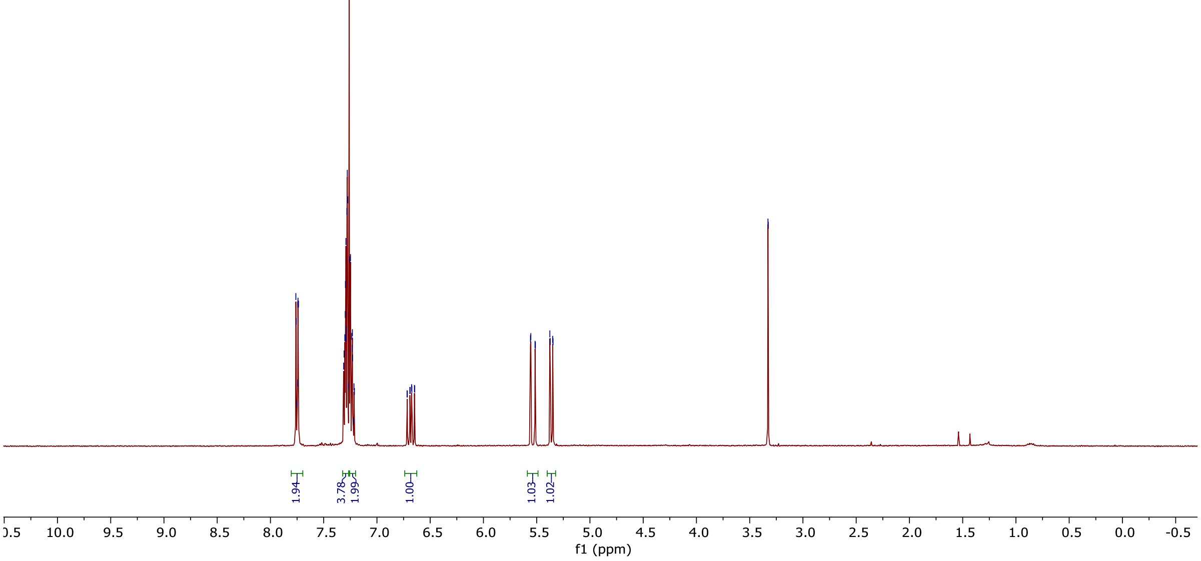

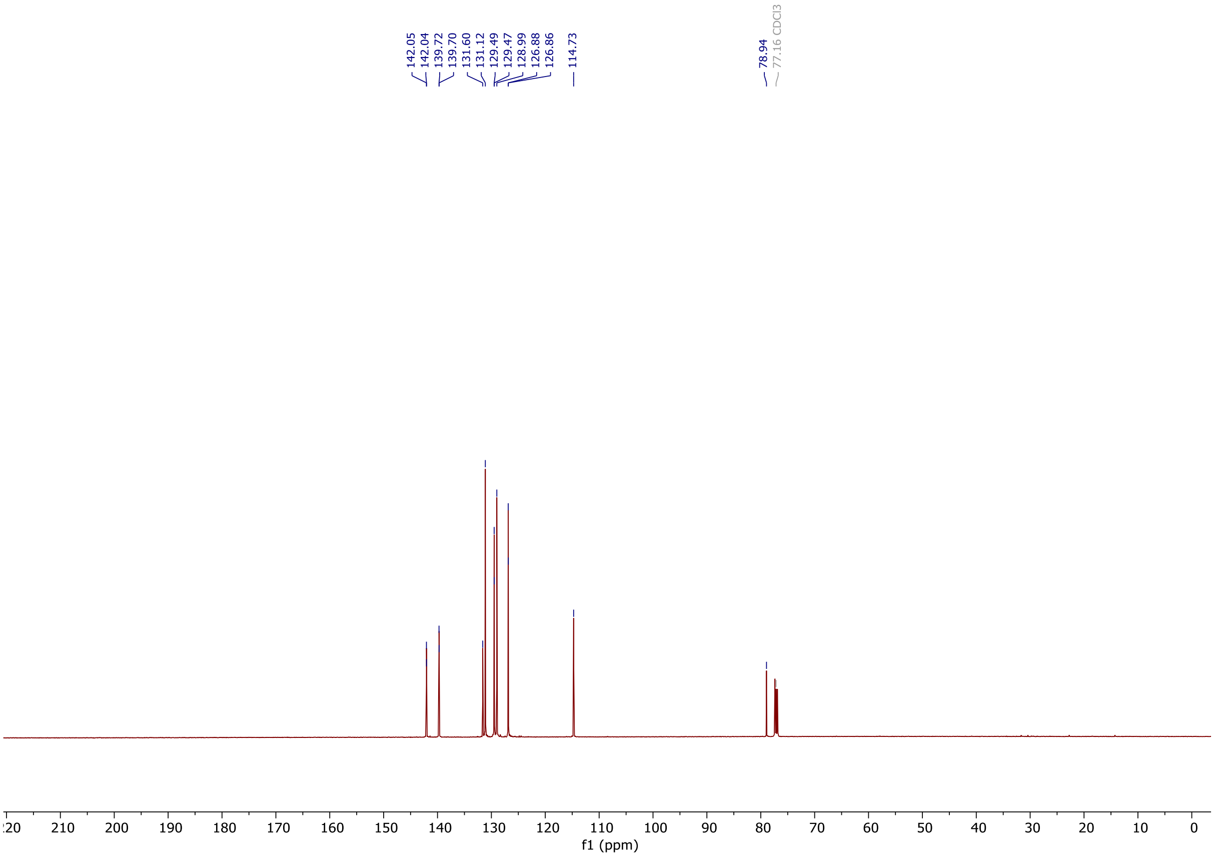

1,1-Di-*o*-tolylprop-2-en-1-ol (**1t**)

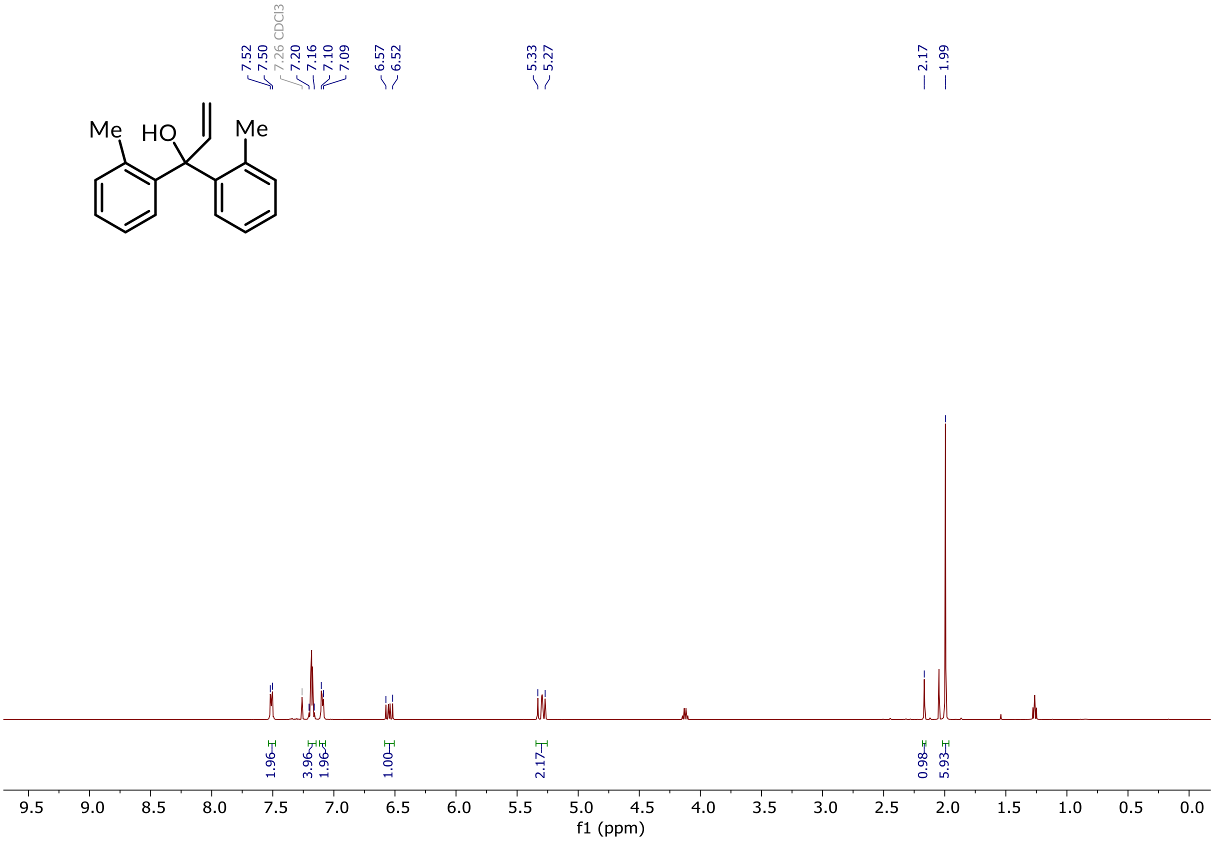

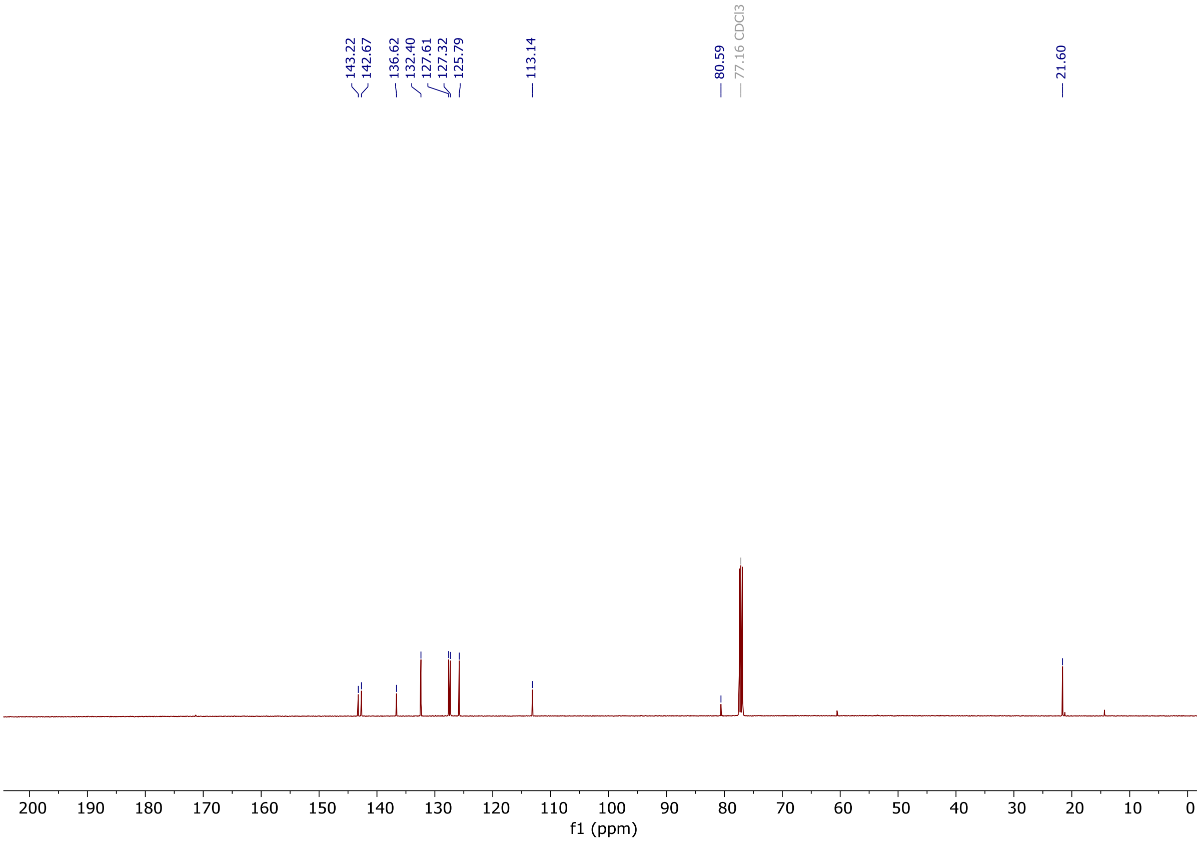

1,1-Bis(2-methoxyphenyl)prop-2-en-1-ol (**1u**)

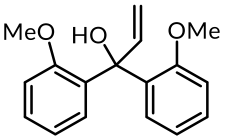

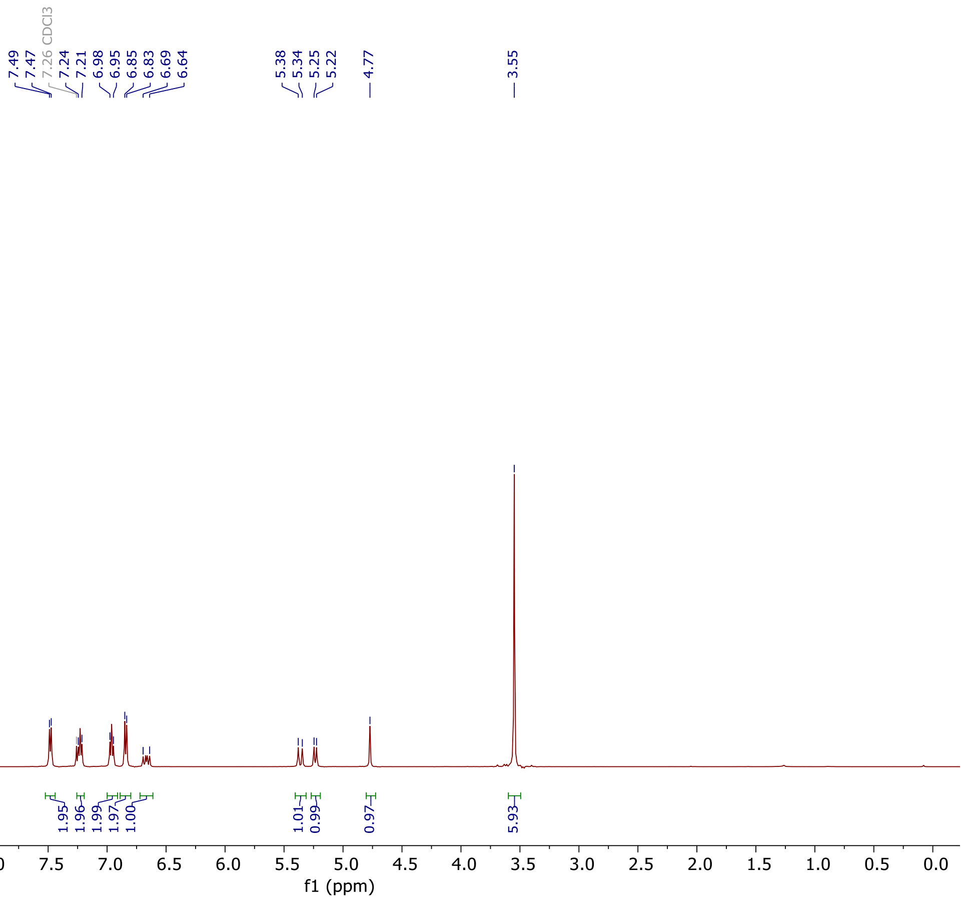

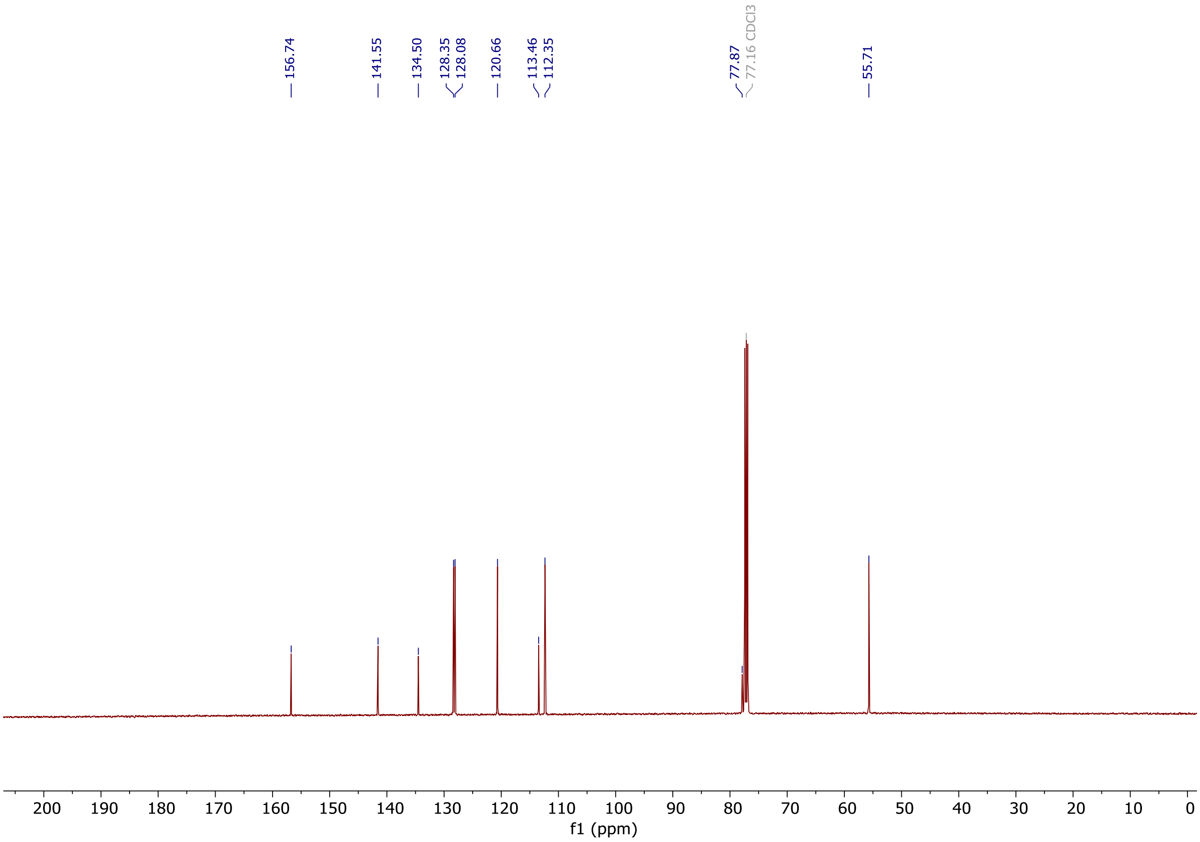

1,1-Bis(2-fluoroyphenyl)prop-2-en-1-ol (**1v**)

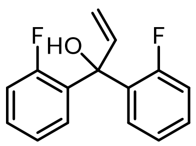

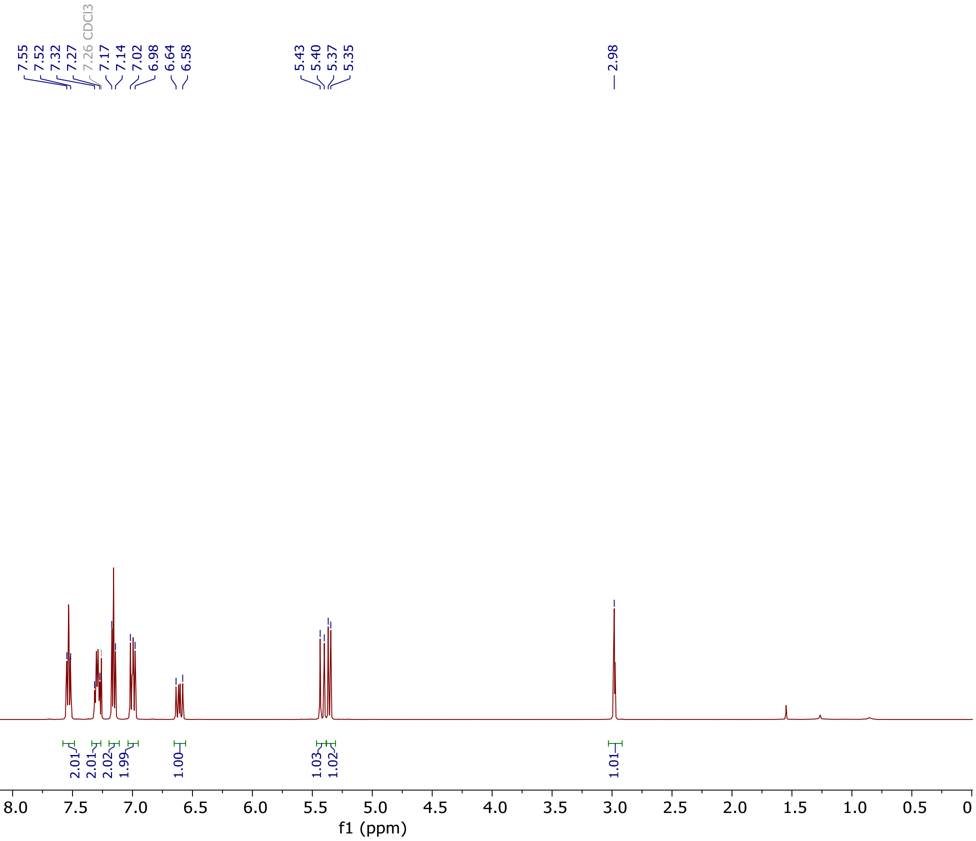

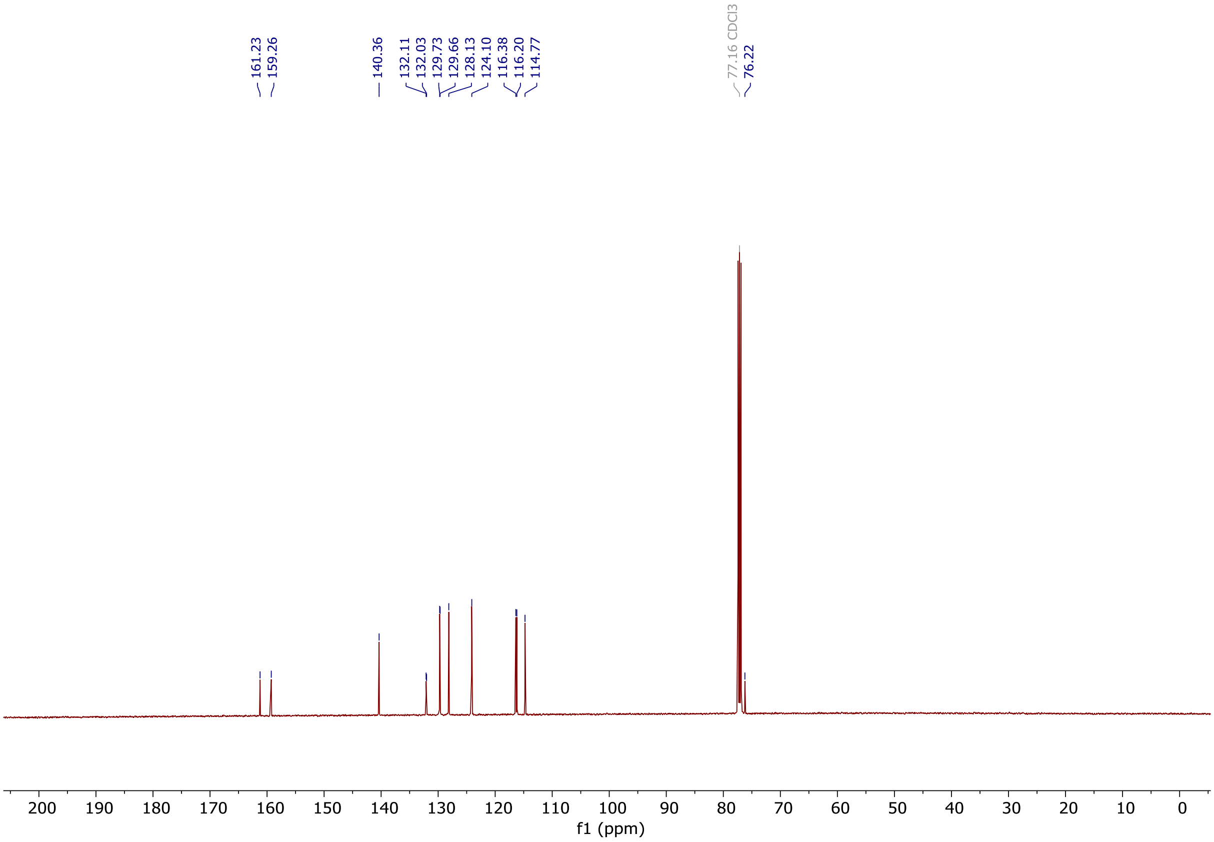

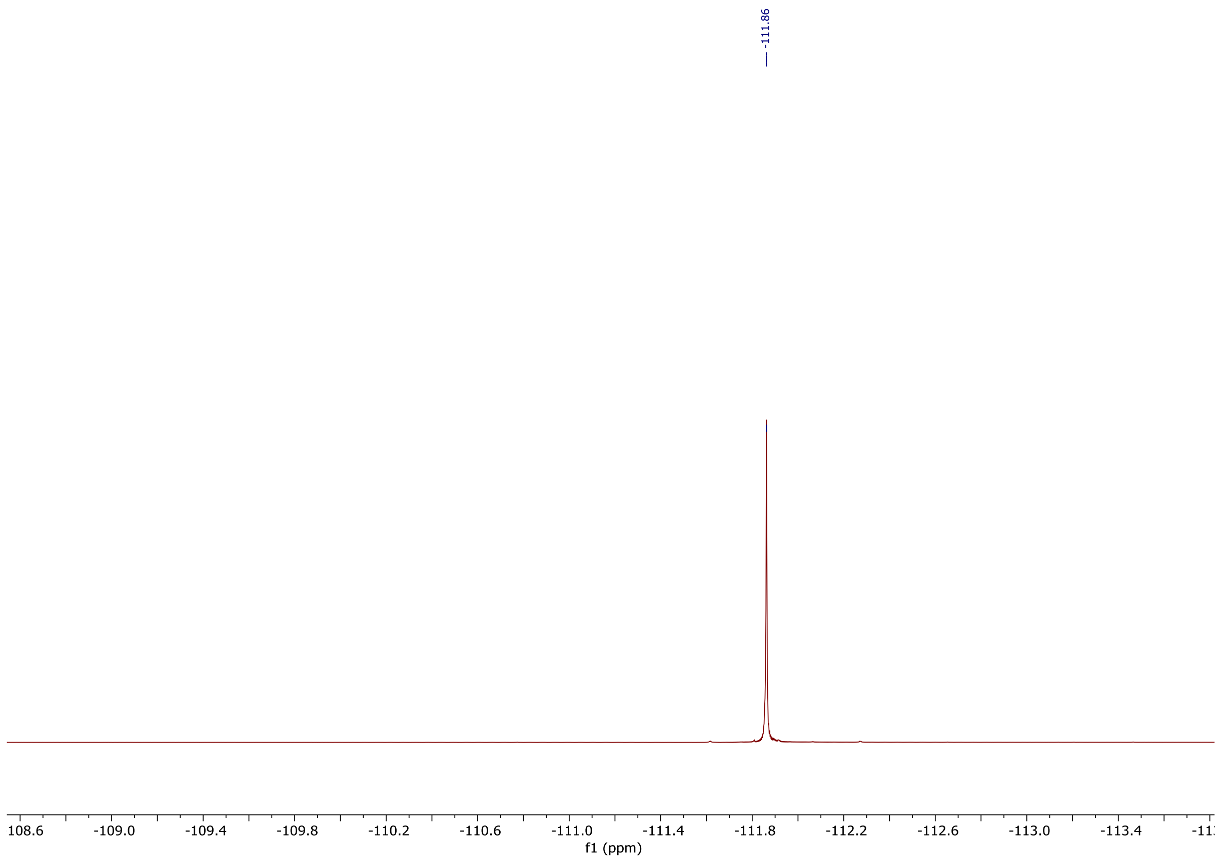

-111.86
108.6
-109.0
-109.4
-109.8
-110.2
-110.6
-111.0
-111.4
-111.8
-112.2
-112.6
-113.0
-113.4
f1 (ppm)

1,1-Di(benzofuran-2-yl)prop-2-en-1-ol (**1w**)

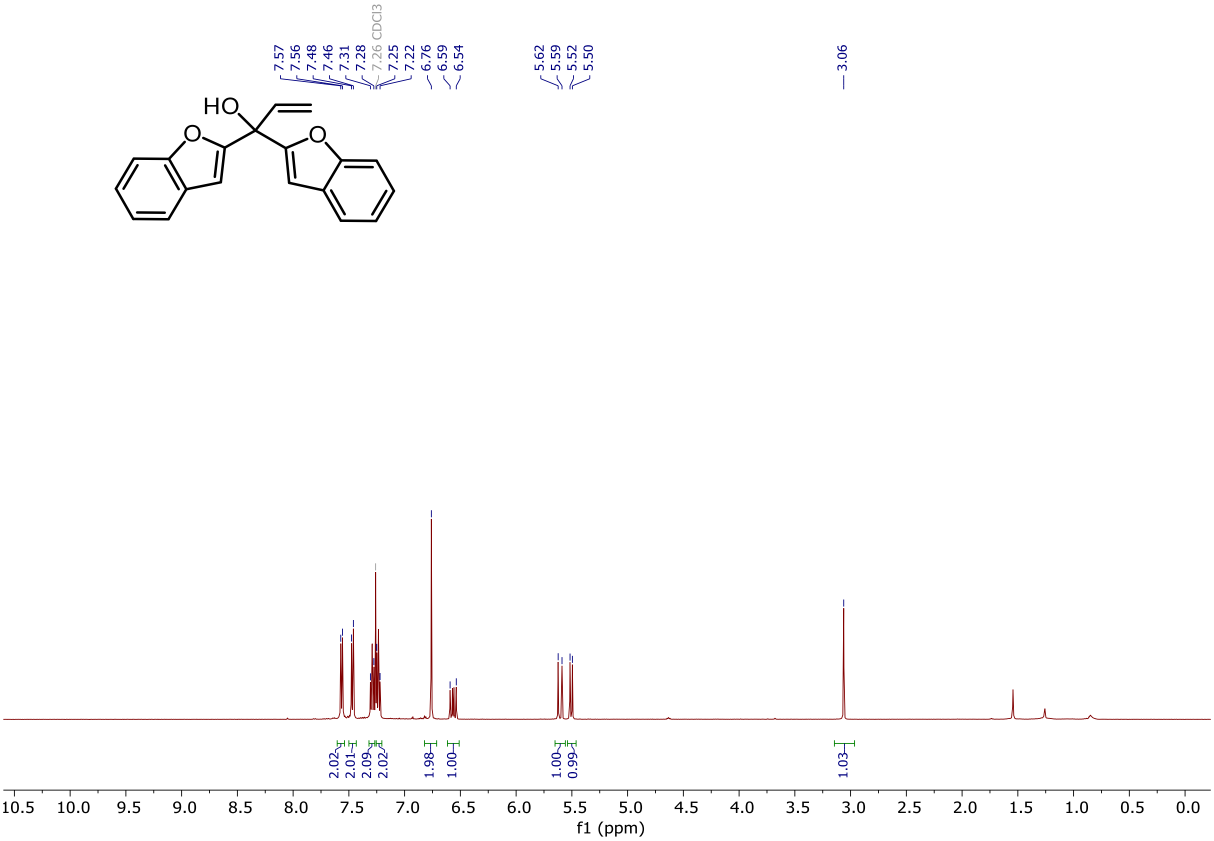

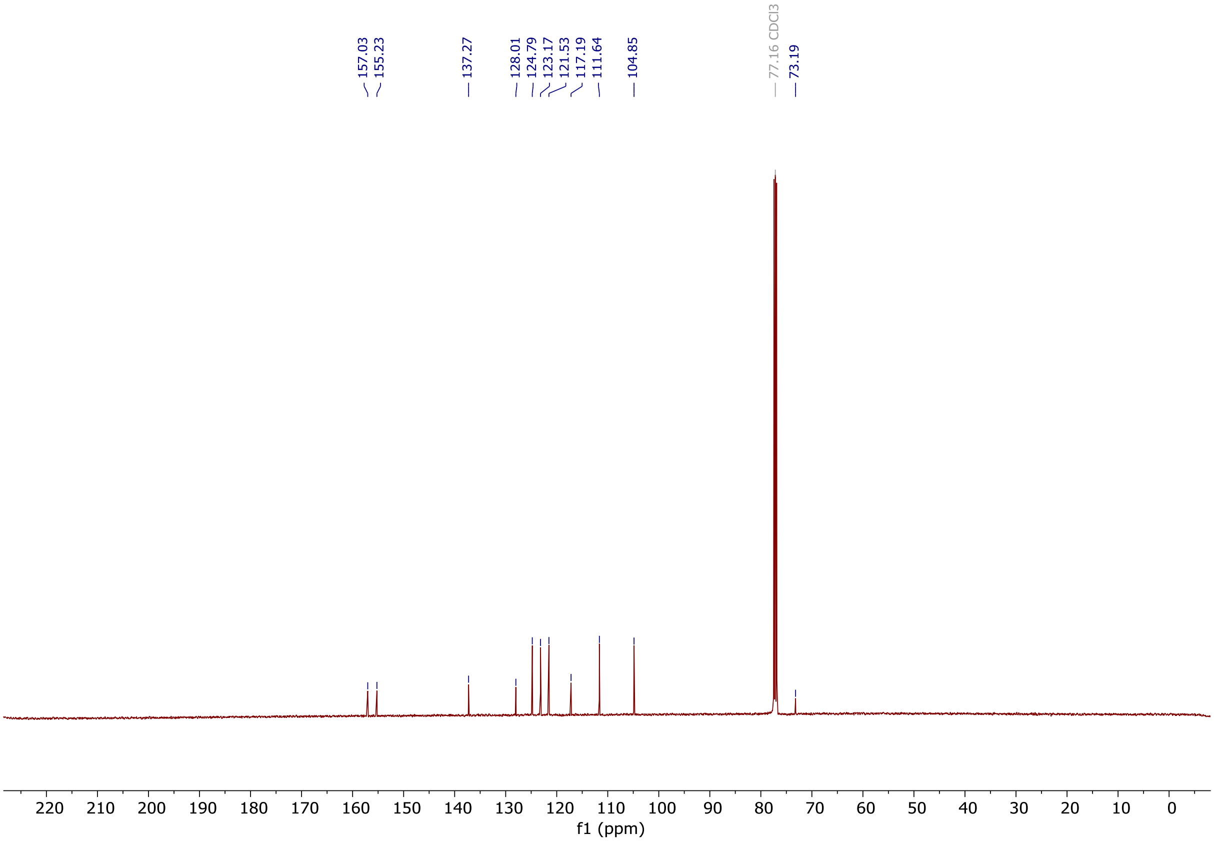

1,1-Bis(benzothiophen-2-yl)prop-2-en-1-ol (**1x**)

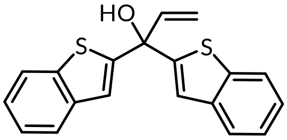

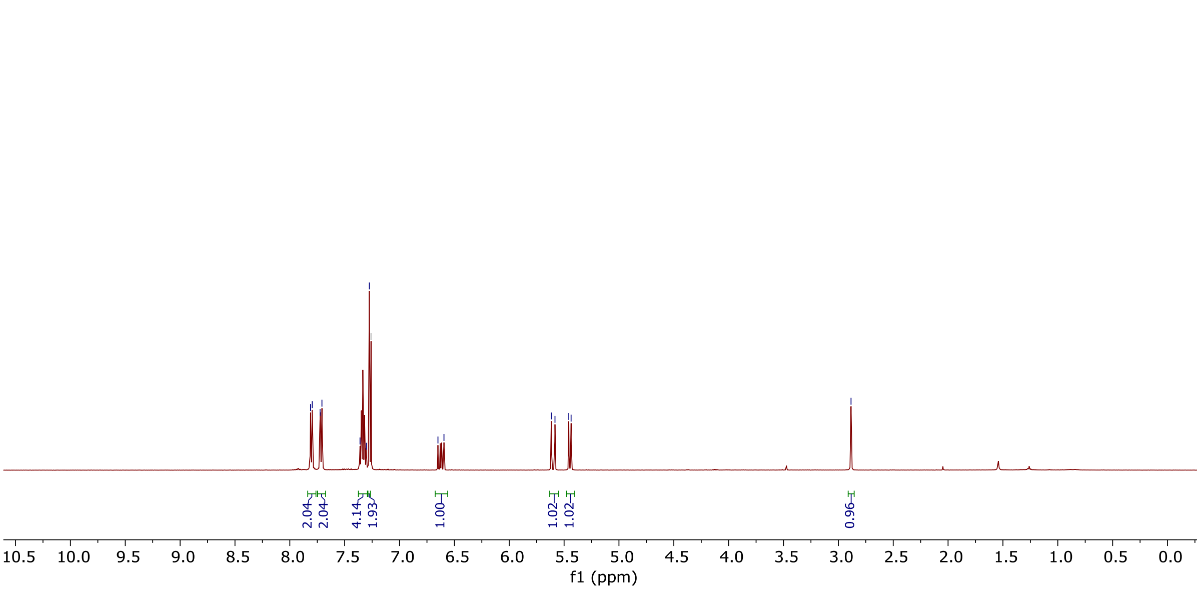

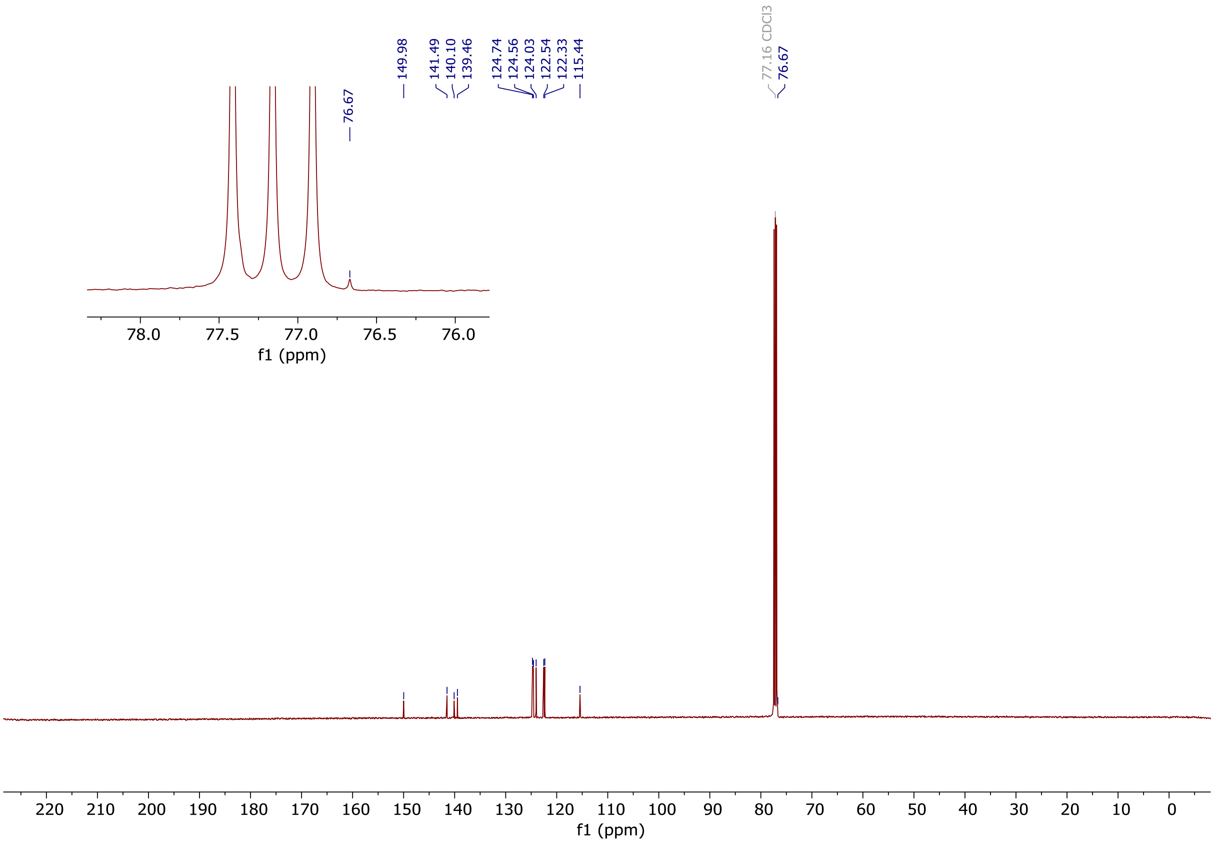

1,1-Di(thiophen-2-yl)prop-2-en-1-ol (**1y**)

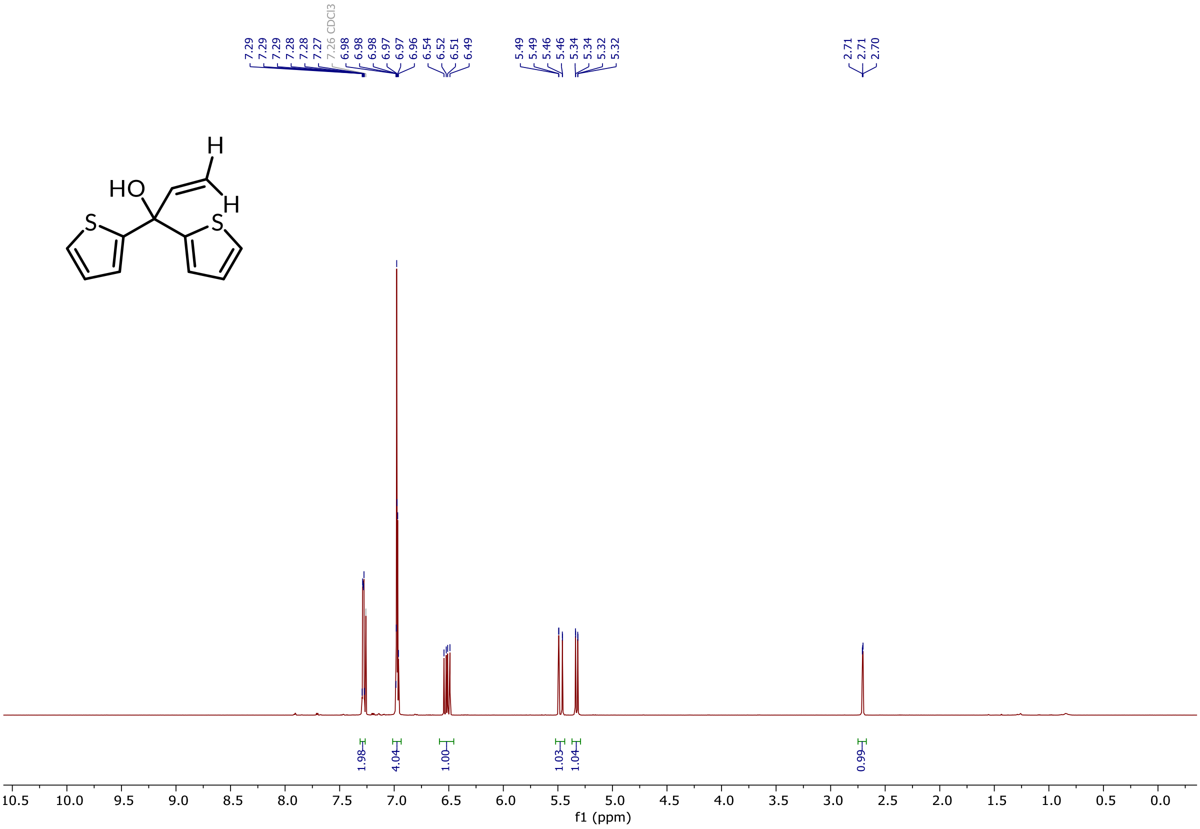

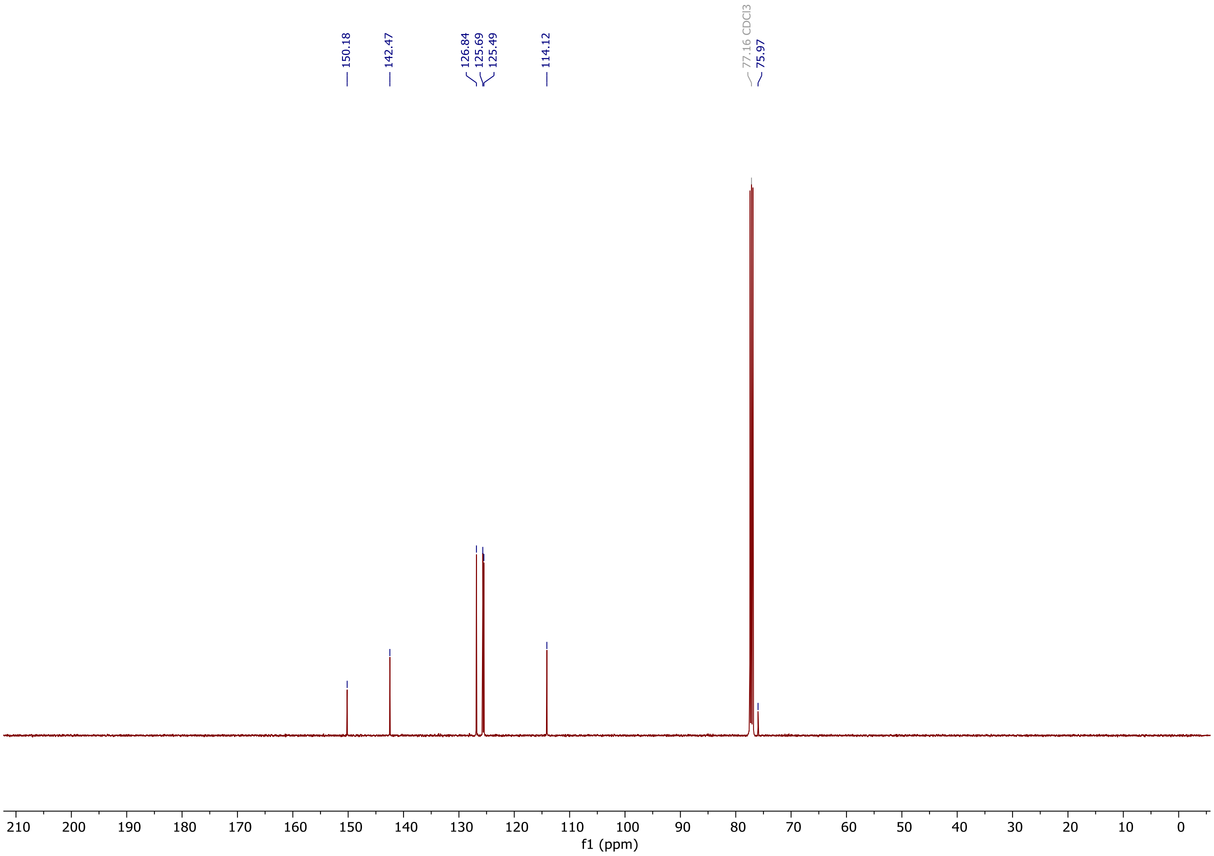

1,1-Bis(6-methoxynaphthalen-2-yl)prop-2-en-1-ol (**1z**)

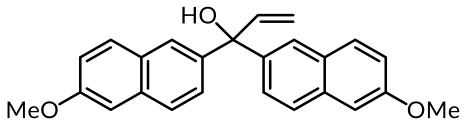

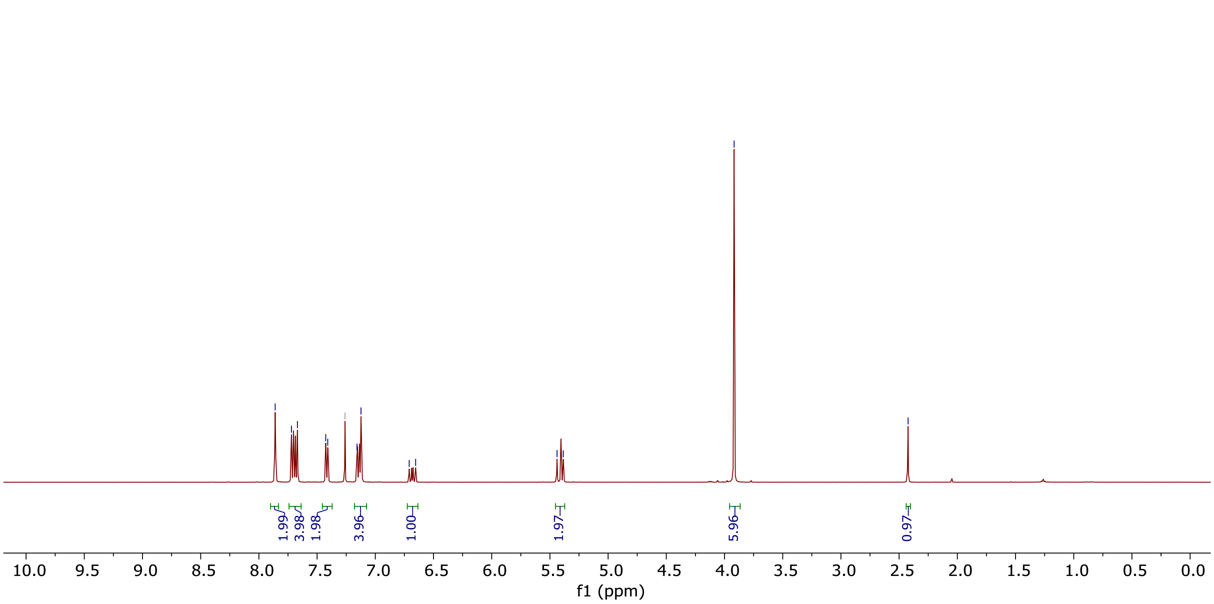

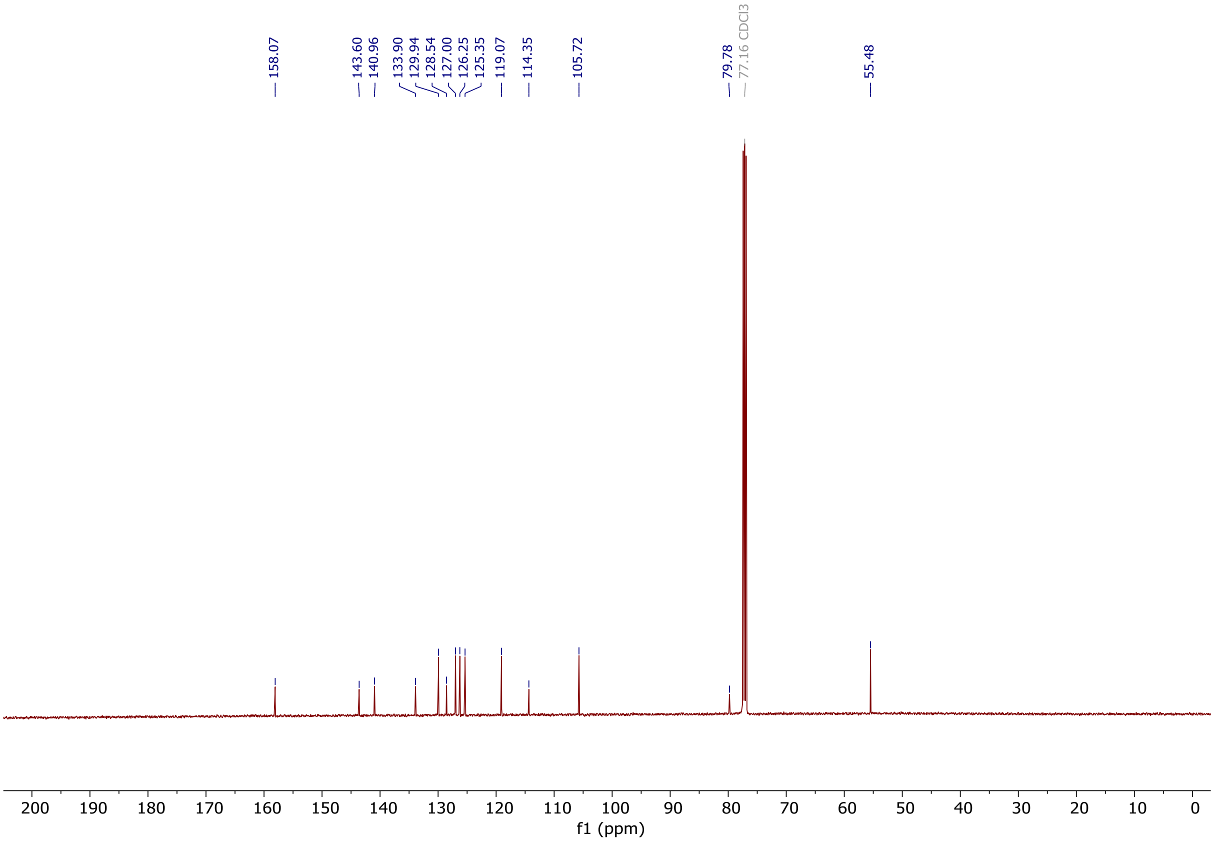

2-Methylene-1,1-diphenylbutan-1-ol (**1aa**)

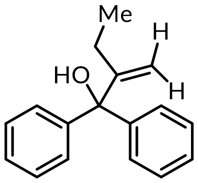

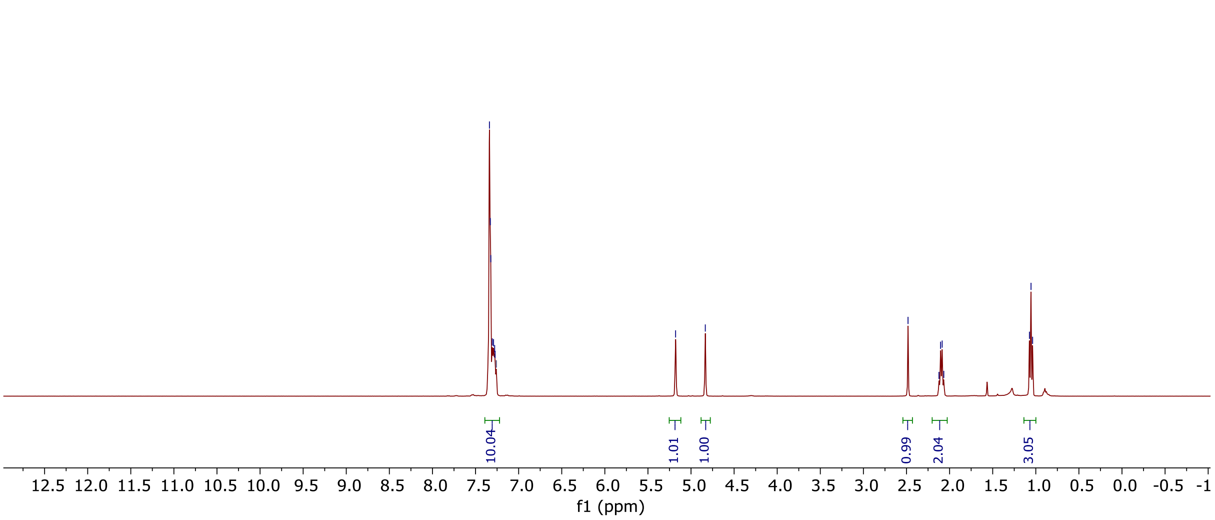

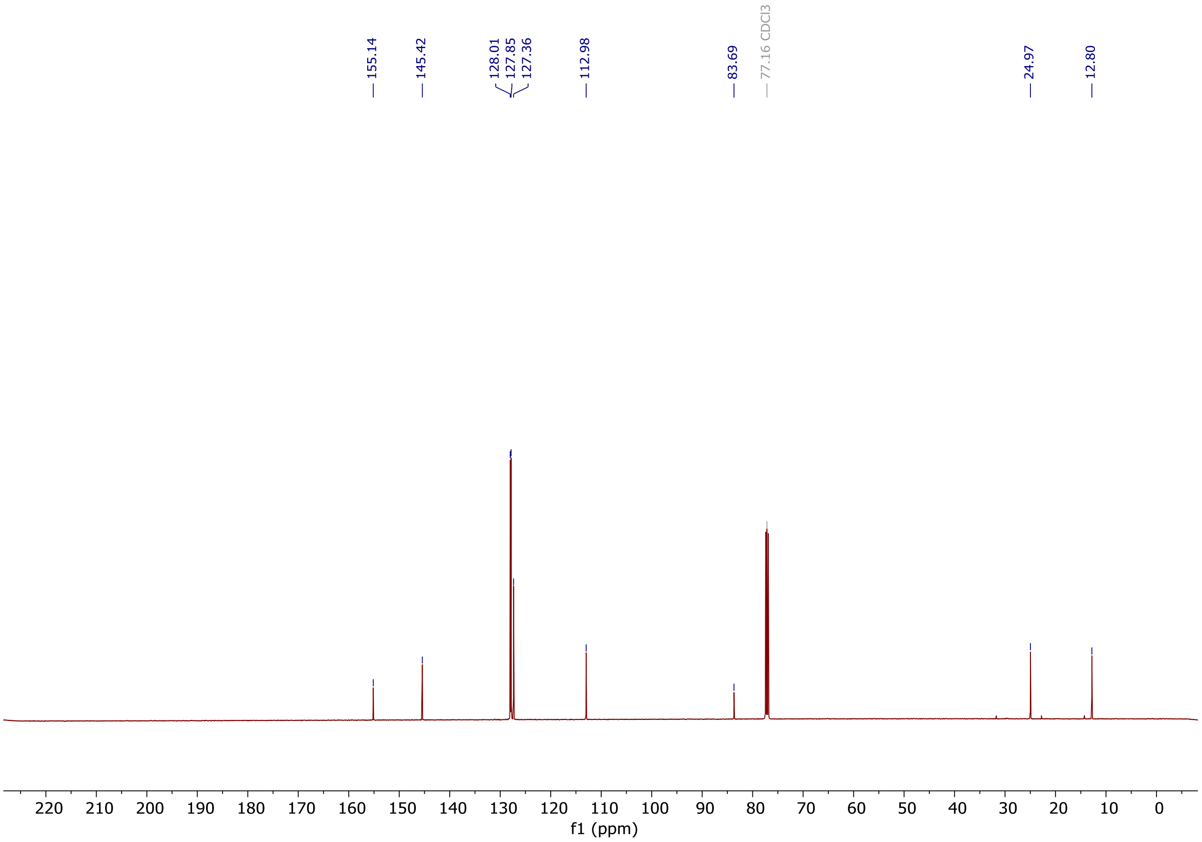

(*Z*)-1,1-diphenylbut-2-en-1-ol (**1ab**)

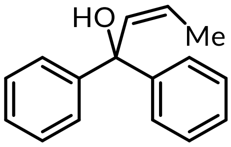

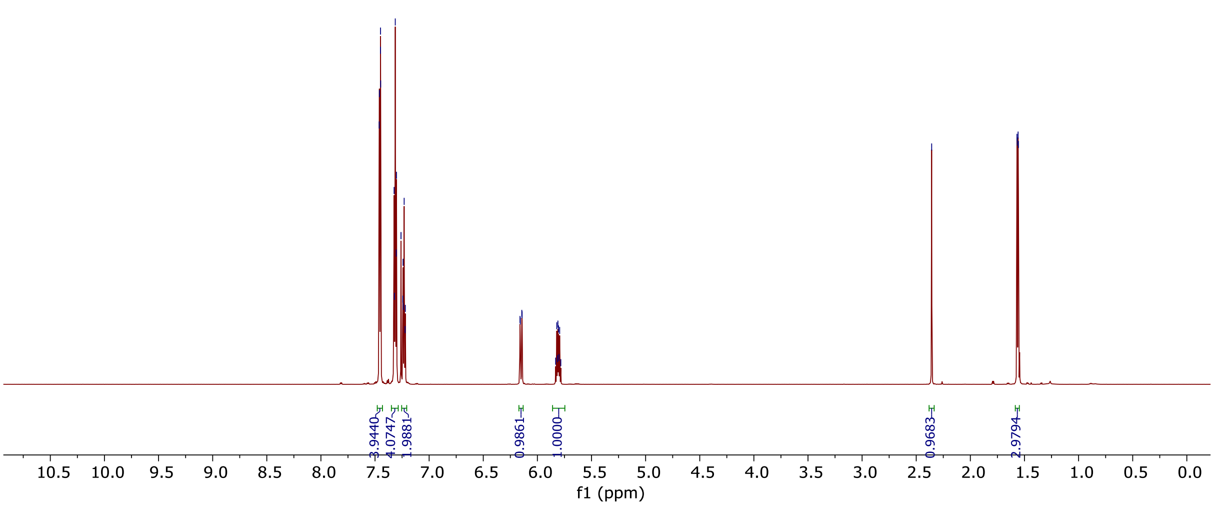

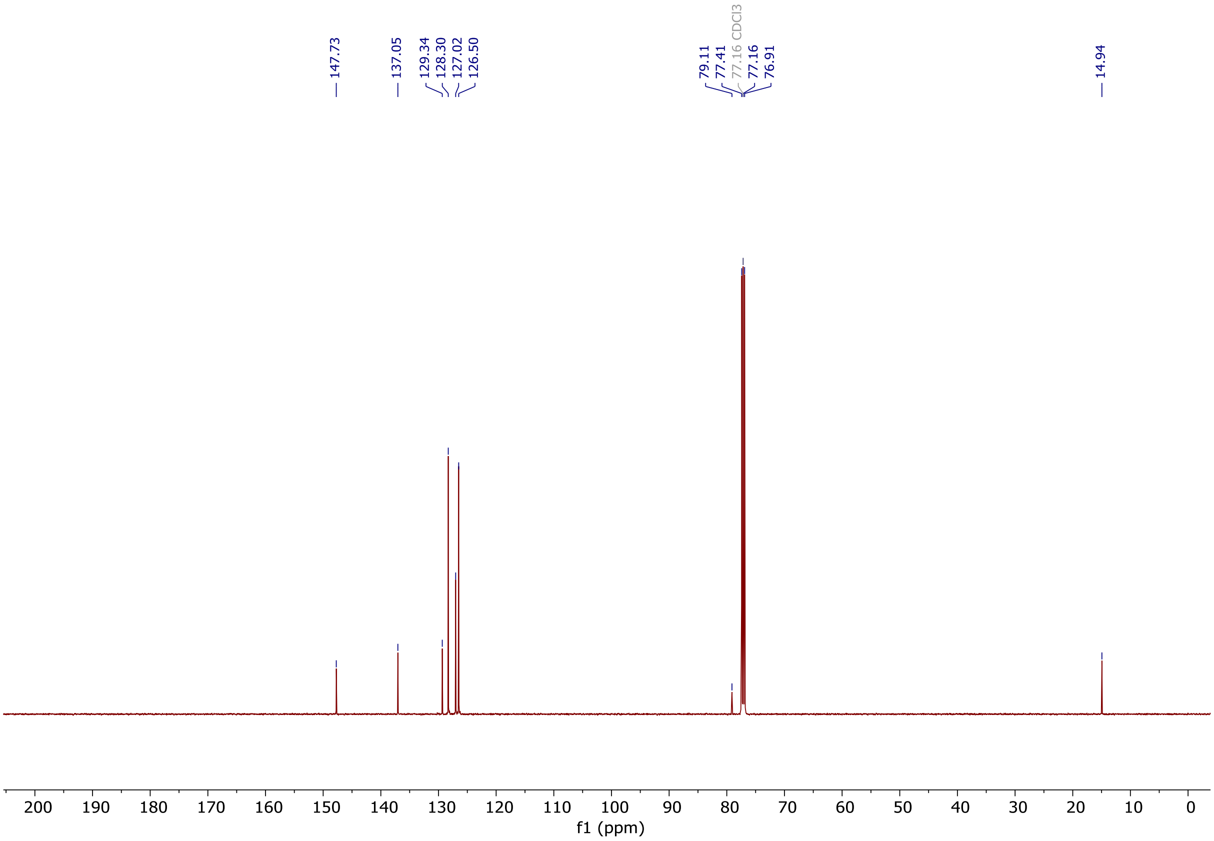

1,1-Bis(4-(pyridine-4-yl)phenyl)prop-2-en-1-ol (**1za**)

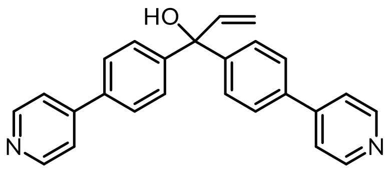

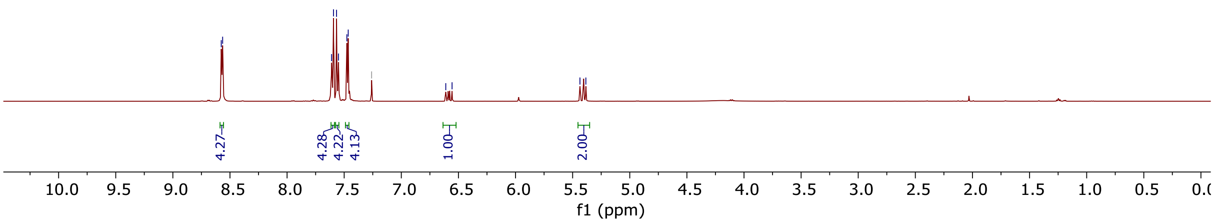

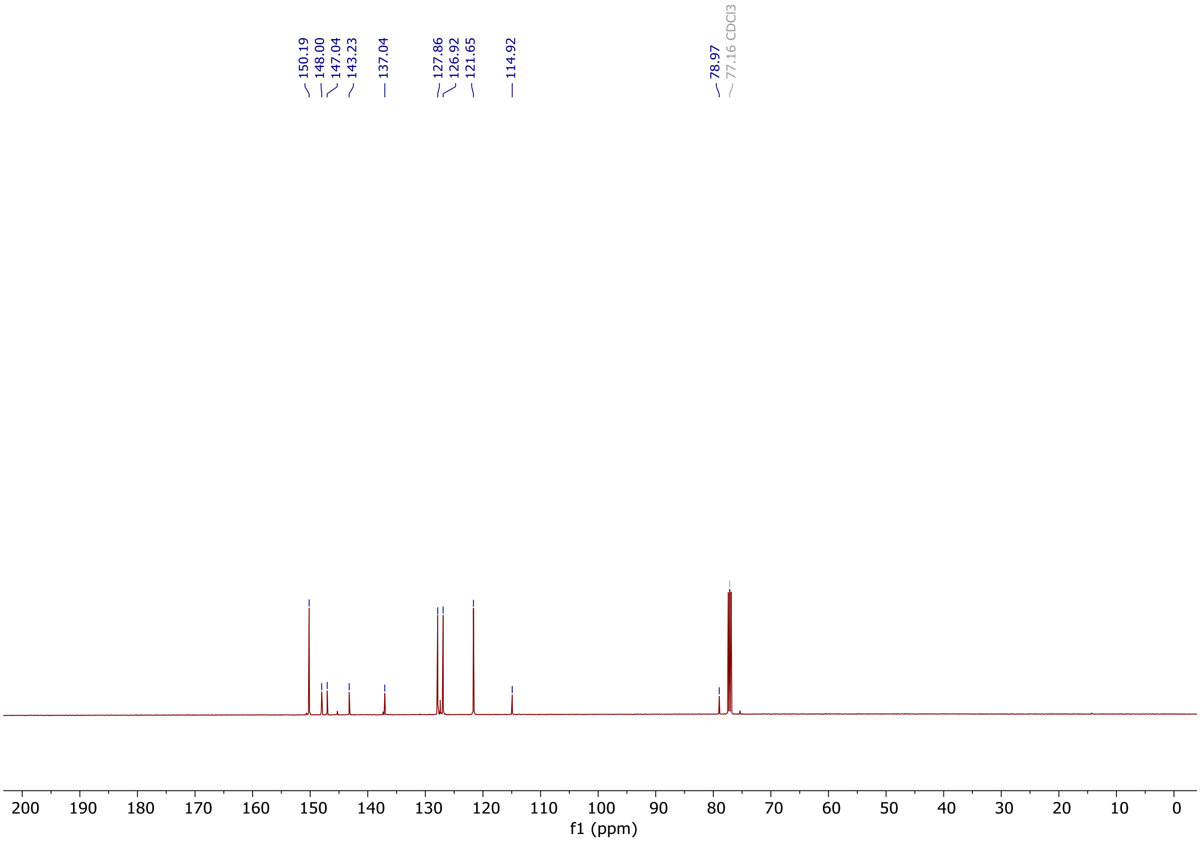

(±)-1-Phenyl-1-(*p*-tolyl)prop-2-en-1-ol (**3a**)

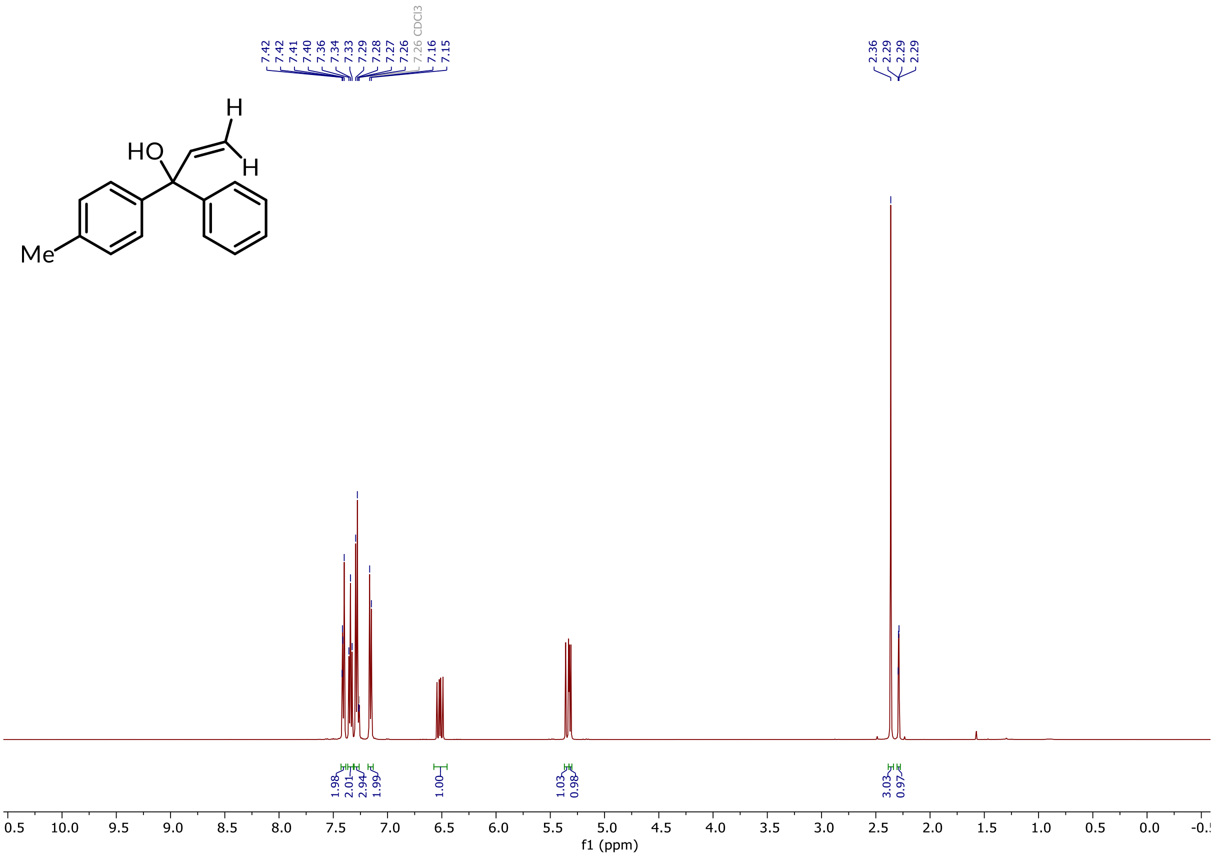

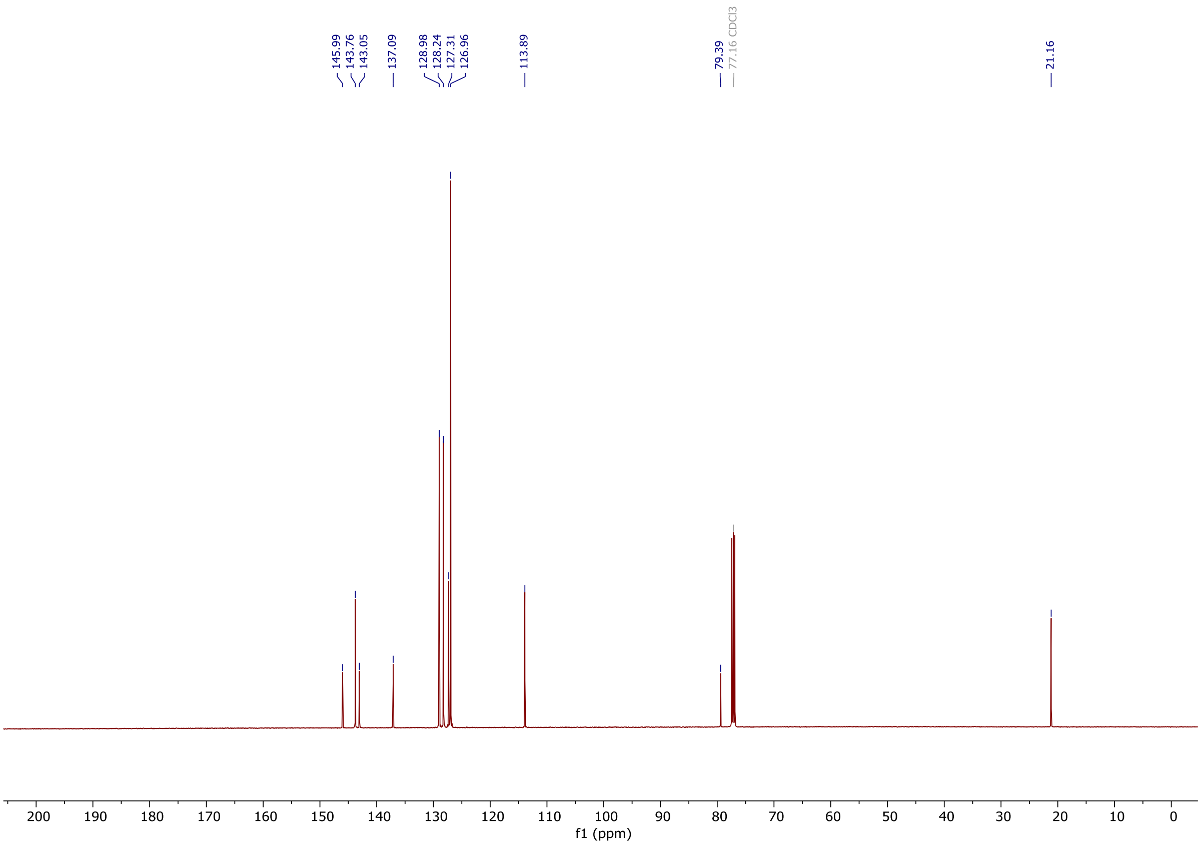

(±)-1-(4-Fluorophenyl)-1-phenylprop-2-en-1-ol (**3b**)

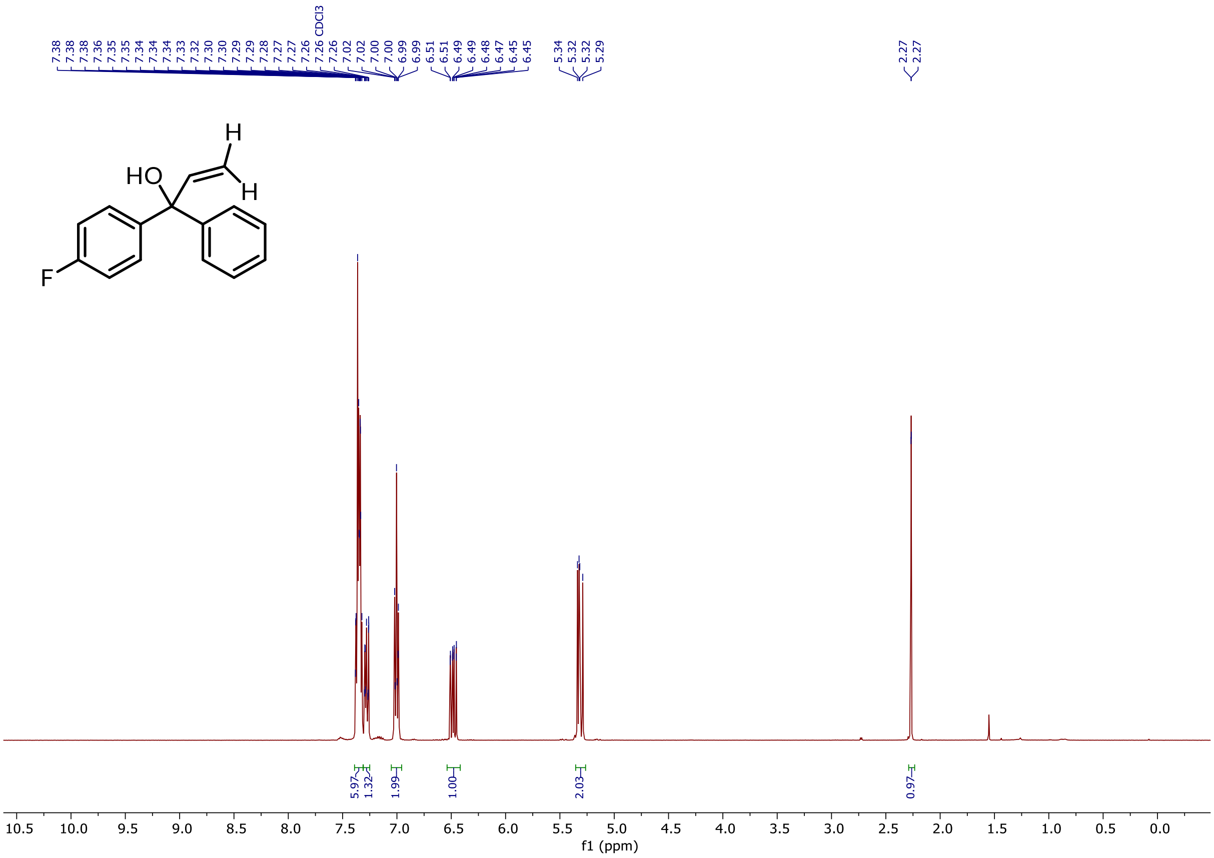

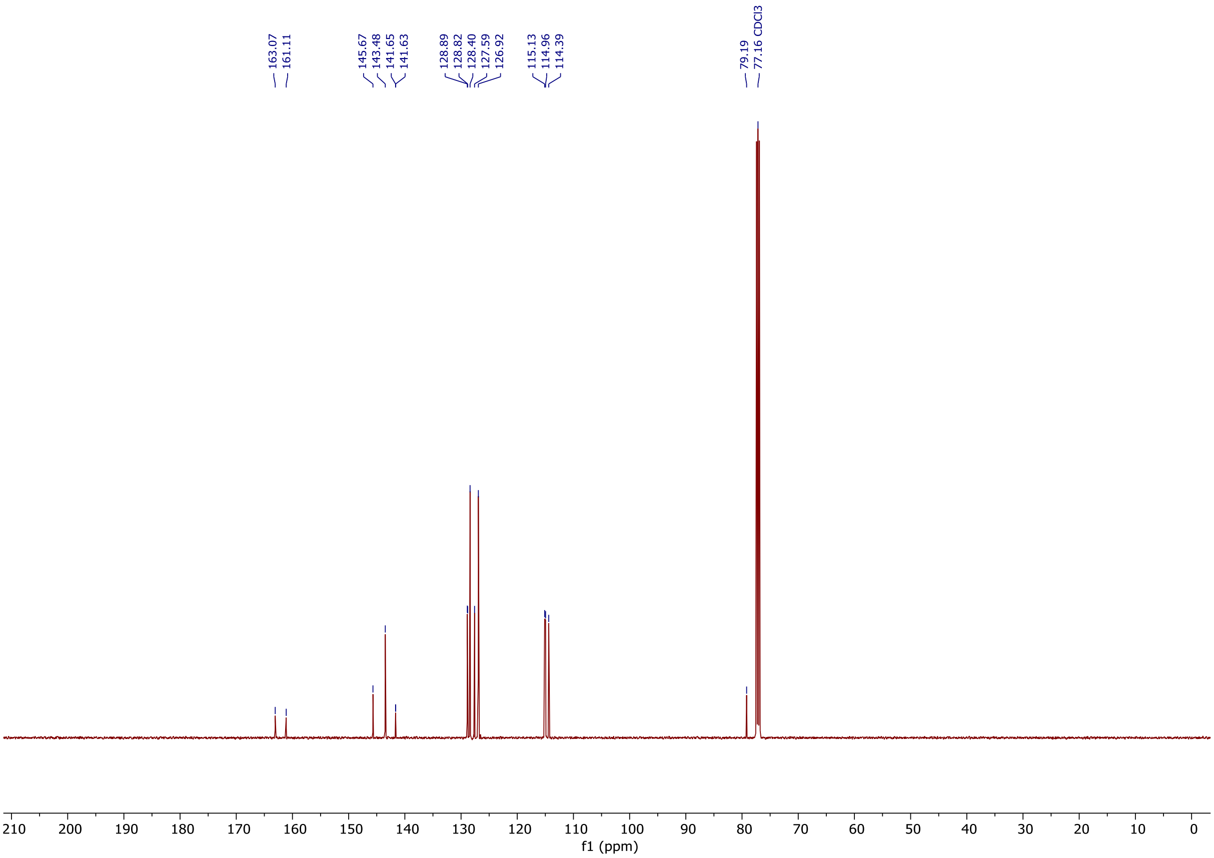

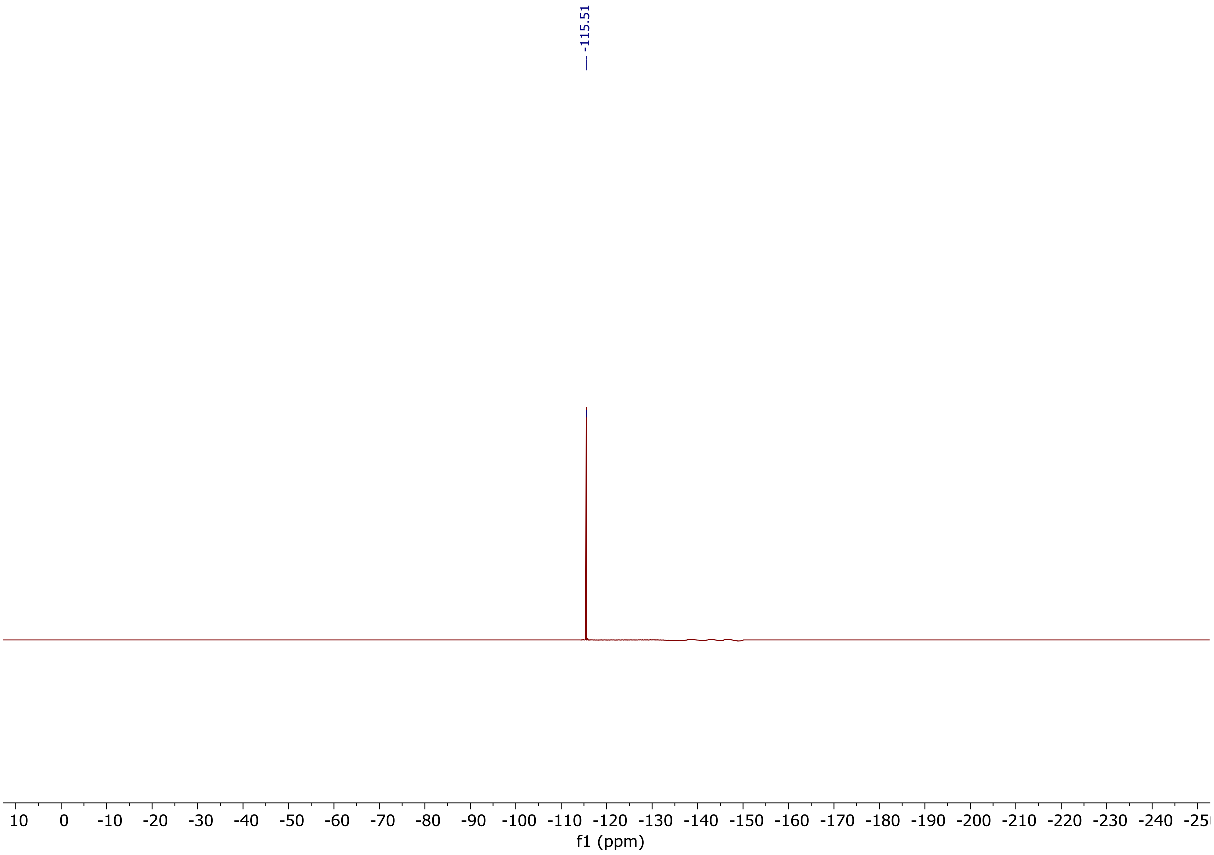
-115.51
10 0 -10 -20 -30 -40 -50 -60 -70 -80 -90 -100 -110 -120 -130 -140 -150 -160 -170 -180 -190 -200 -210 -220 -230 -240 -250
f1 (ppm)

(±)-1-(4-Chlorophenyl)-1-phenylprop-2-en-1-ol (**3c**)

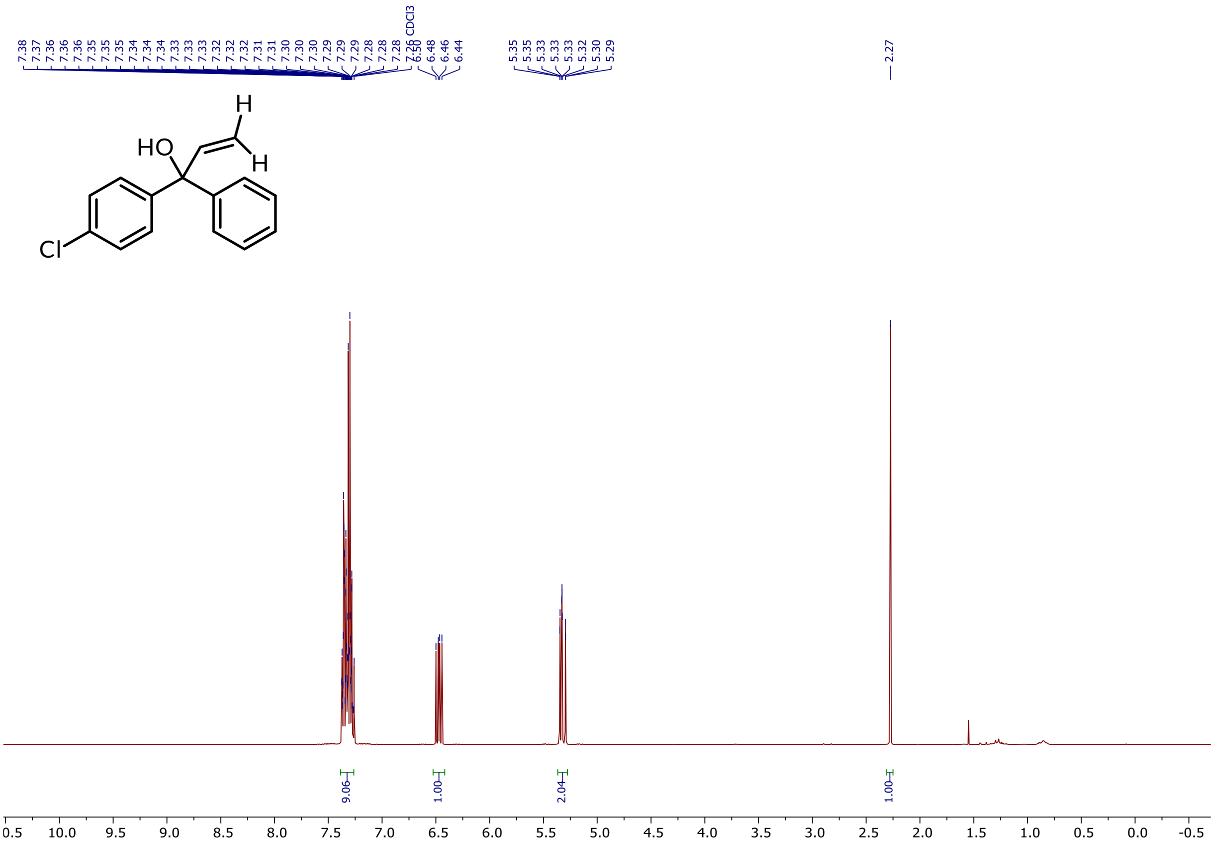

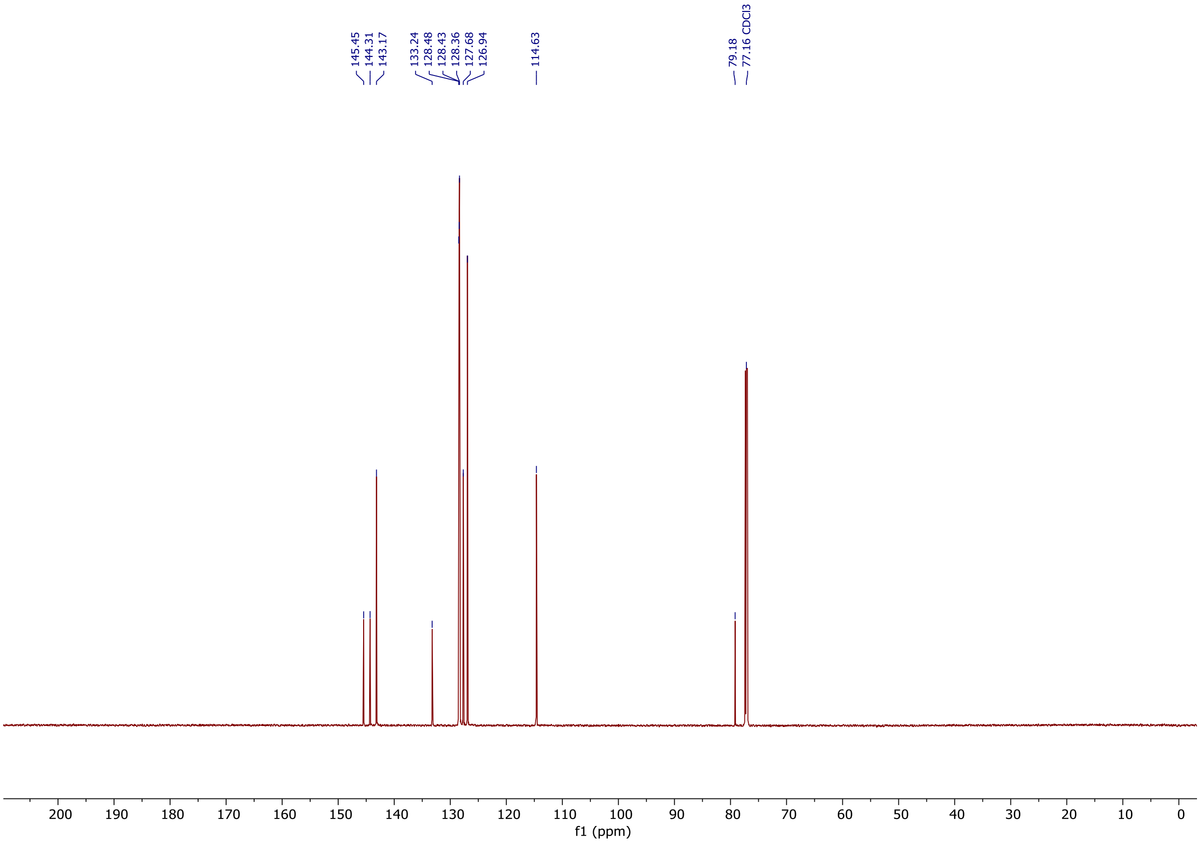

(±)-1-(3-Fluorophenyl)-1-phenylprop-2-en-1-ol (**3d**)

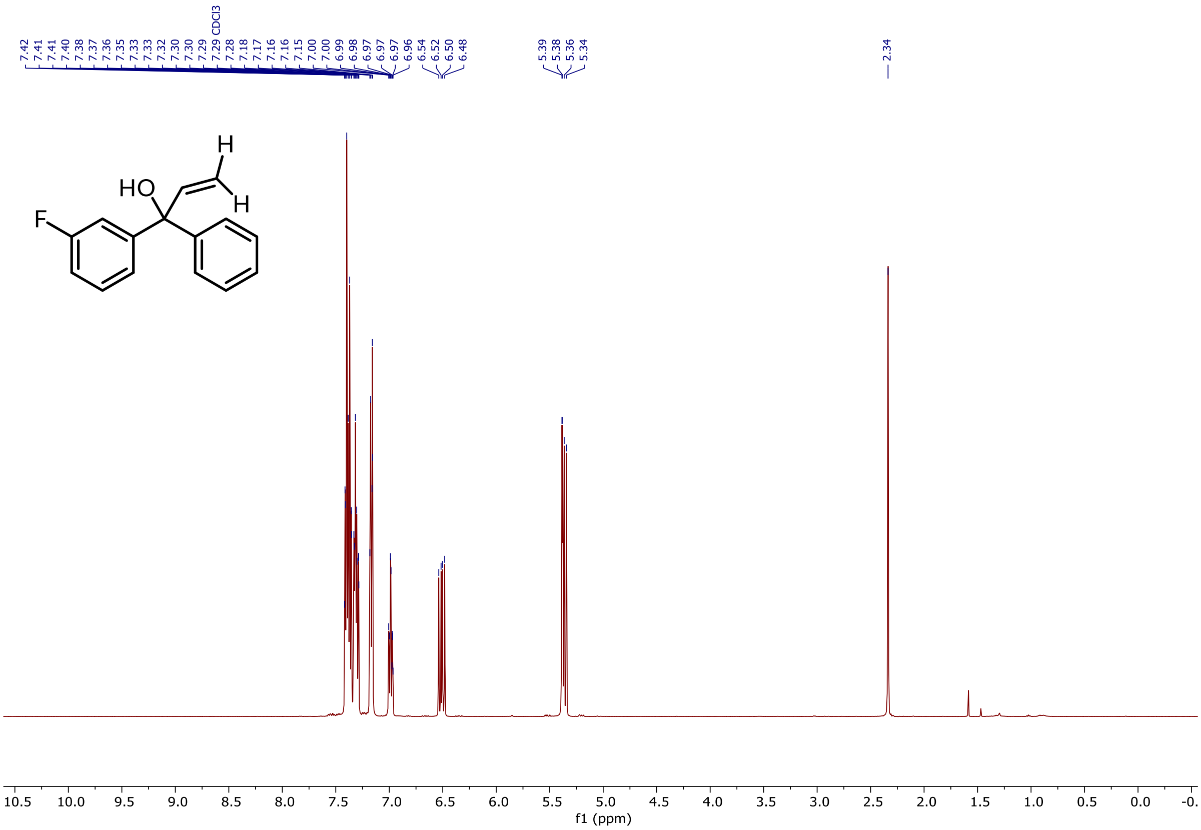

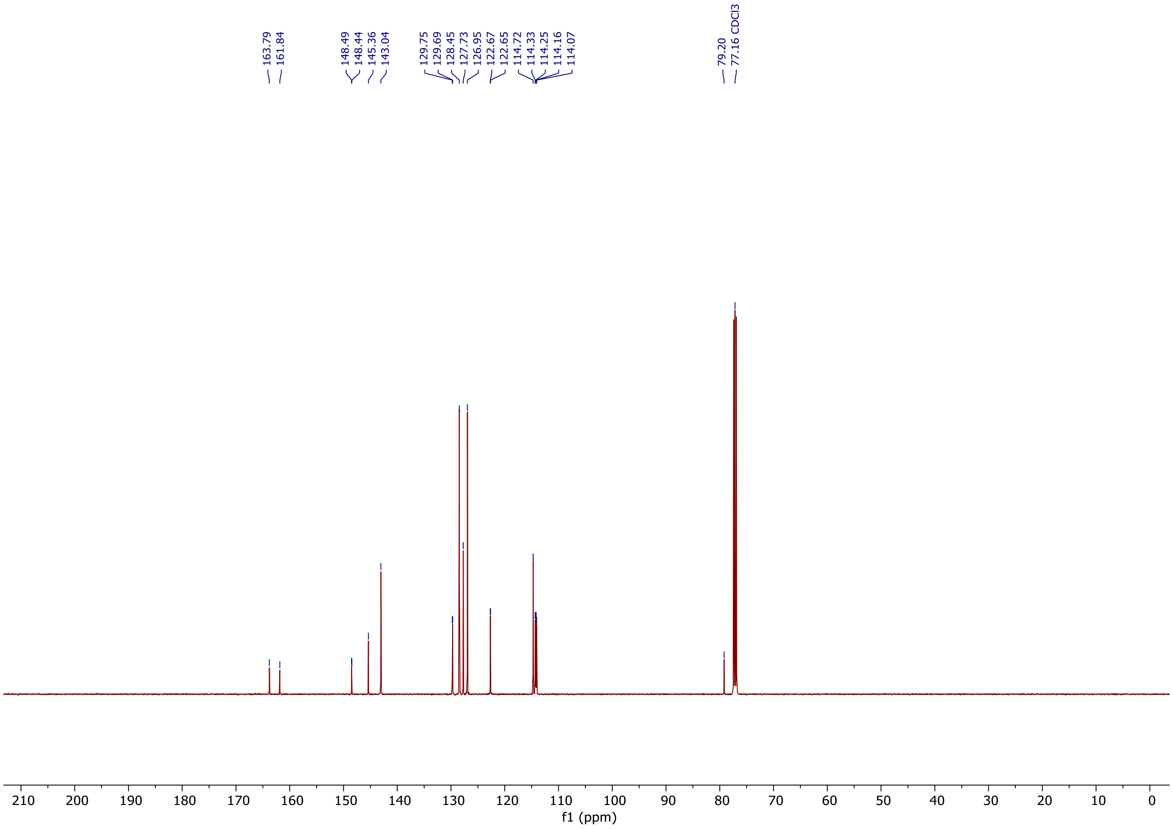

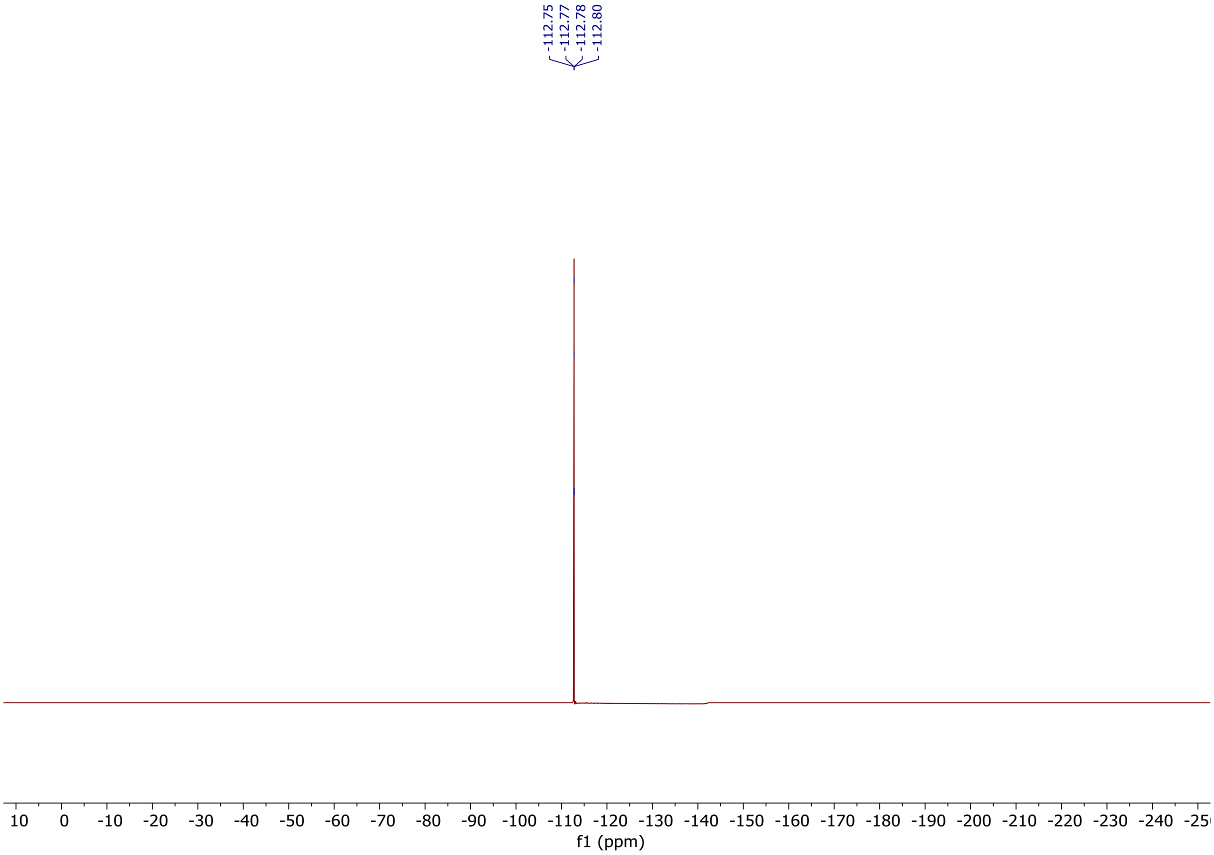

-112.75
-112.77
-112.78
-112.80
10 0 -10 -20 -30 -40 -50 -60 -70 -80 -90 -100 -110 -120 -130 -140 -150 -160 -170 -180 -190 -200 -210 -220 -230 -240
f1 (ppm)

(±)-1-Phenyl-1-(4-(trifluoromethyl)phenyl)prop-2-en-1-ol (**3e**)

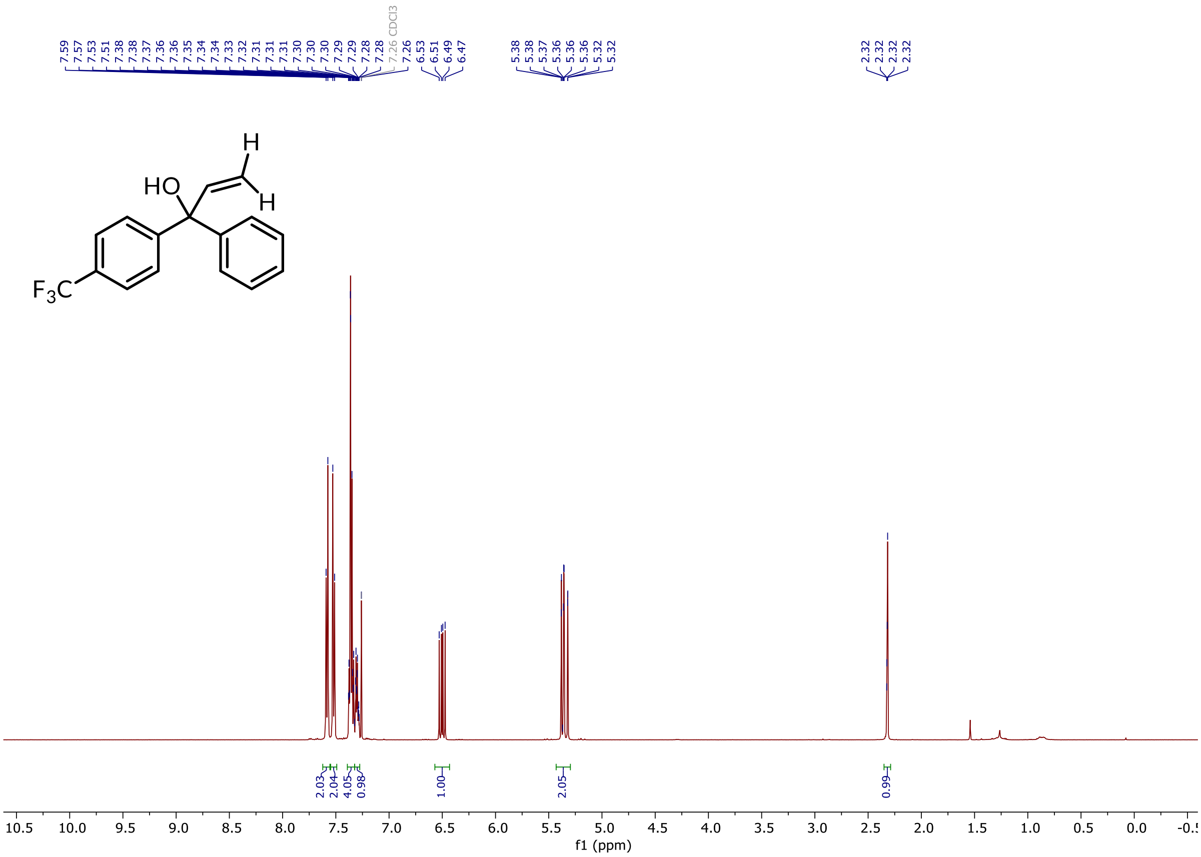

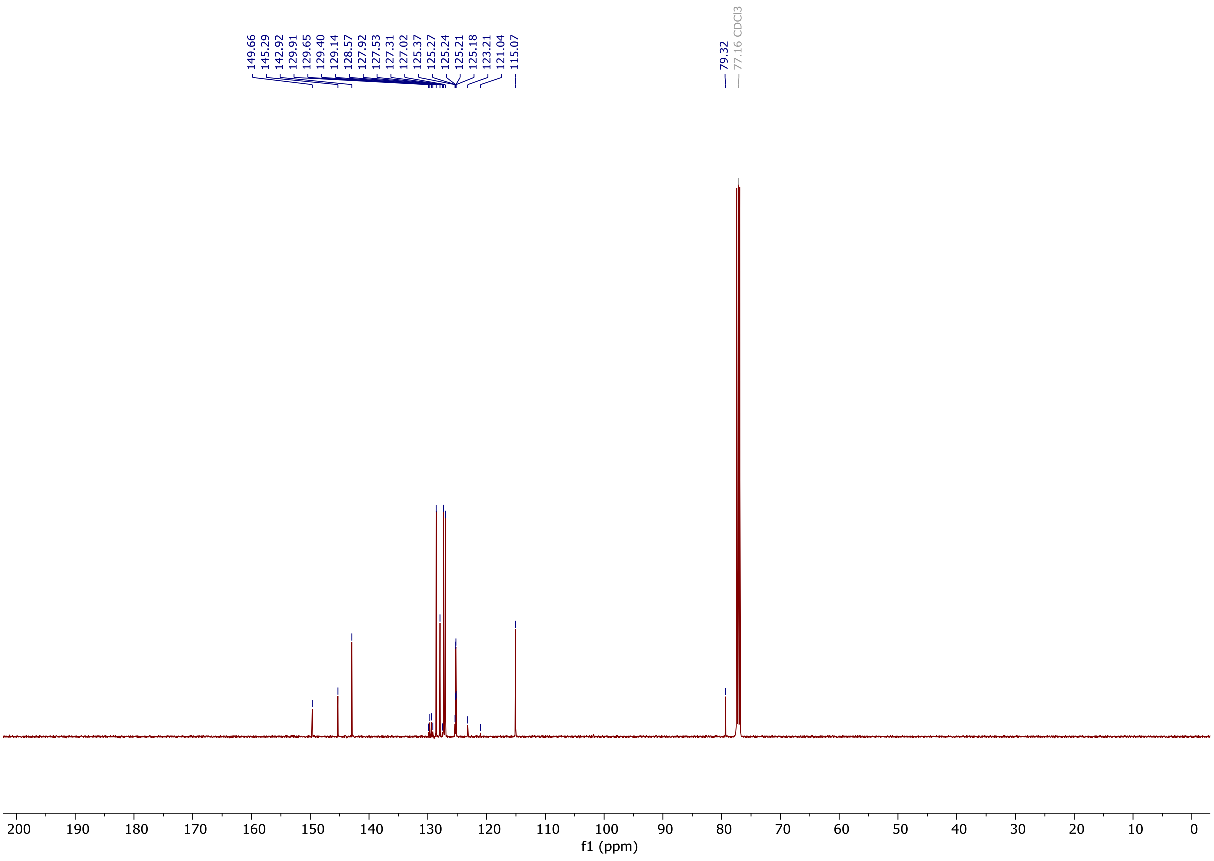

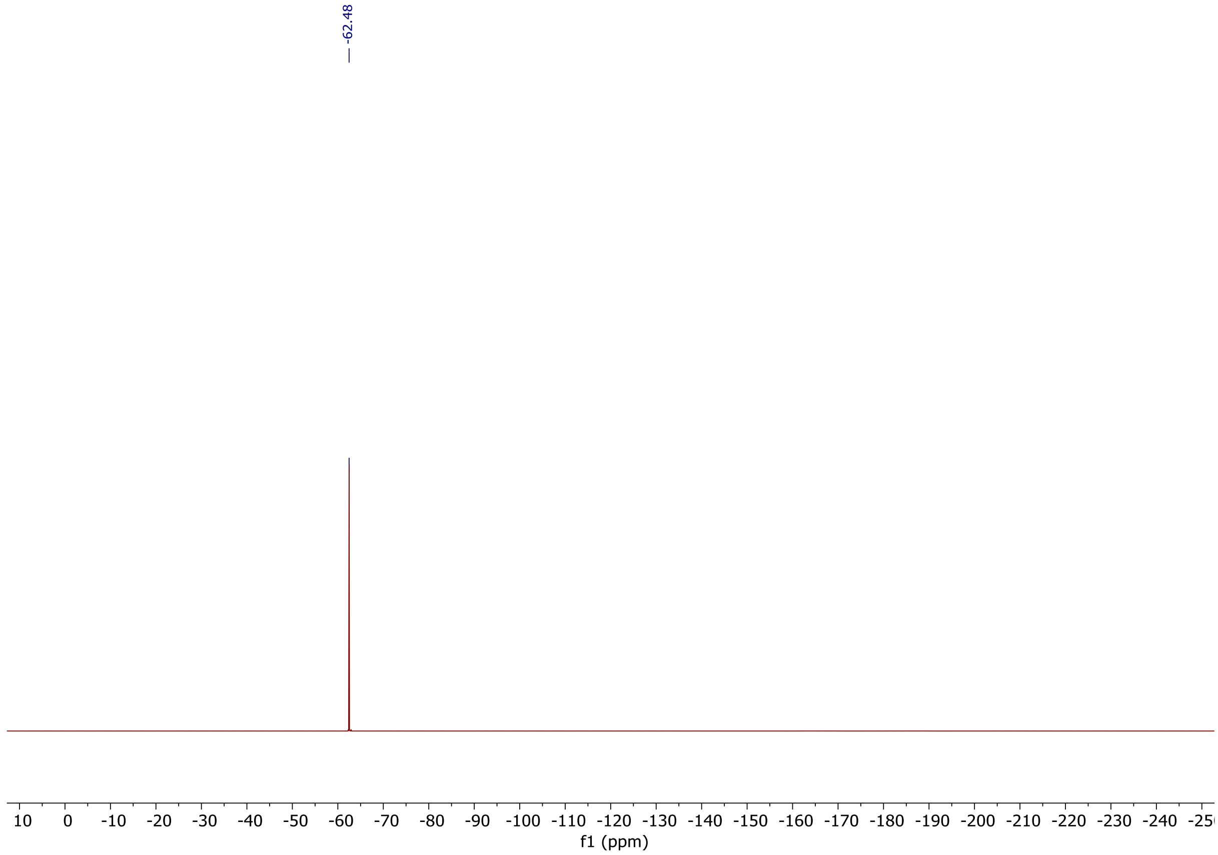
-62.48
10 0 -10 -20 -30 -40 -50 -60 -70 -80 -90 -100 -110 -120 -130 -140 -150 -160 -170 -180 -190 -200 -210 -220 -230 -240 -250
f1 (ppm)

(±)-1-(3-Fluoro-4-(trifluoromethyl)phenyl)-1-phenylprop-2-en-1-ol (**3f**)

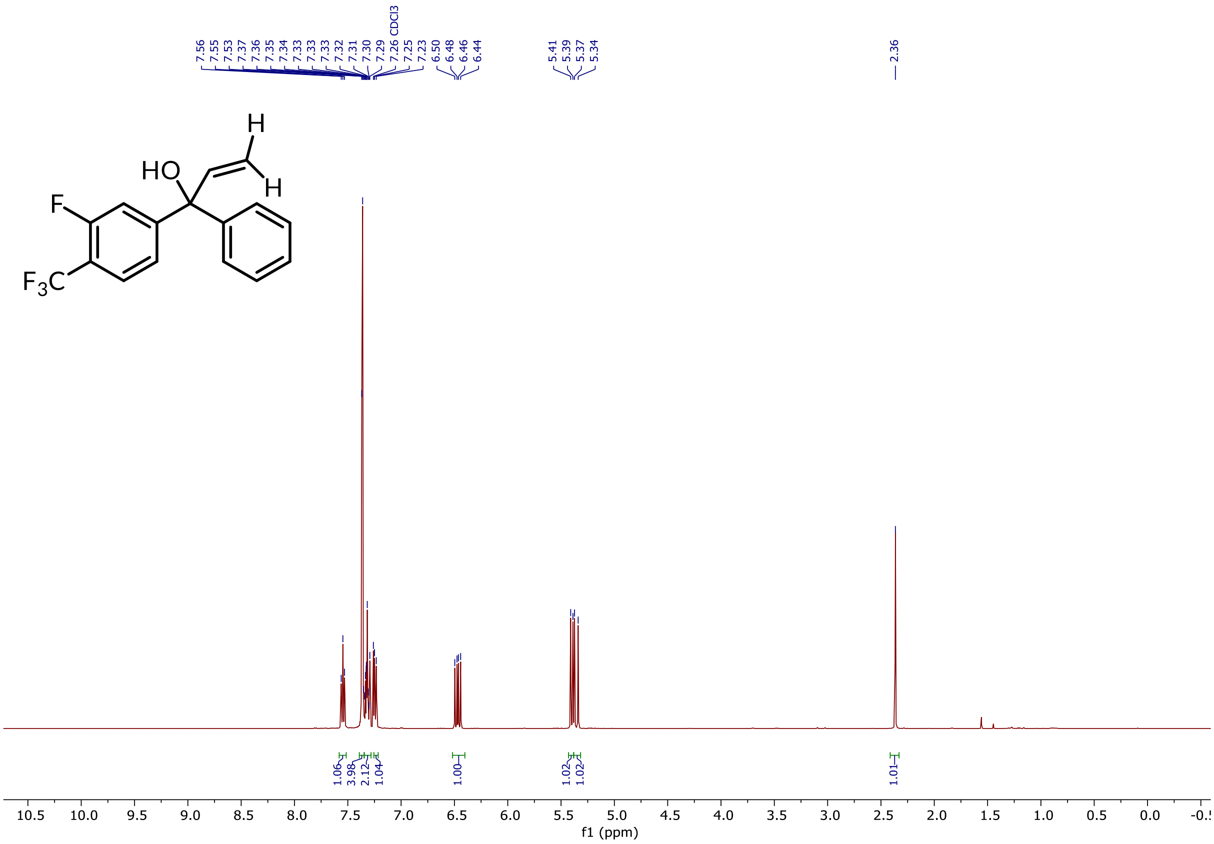

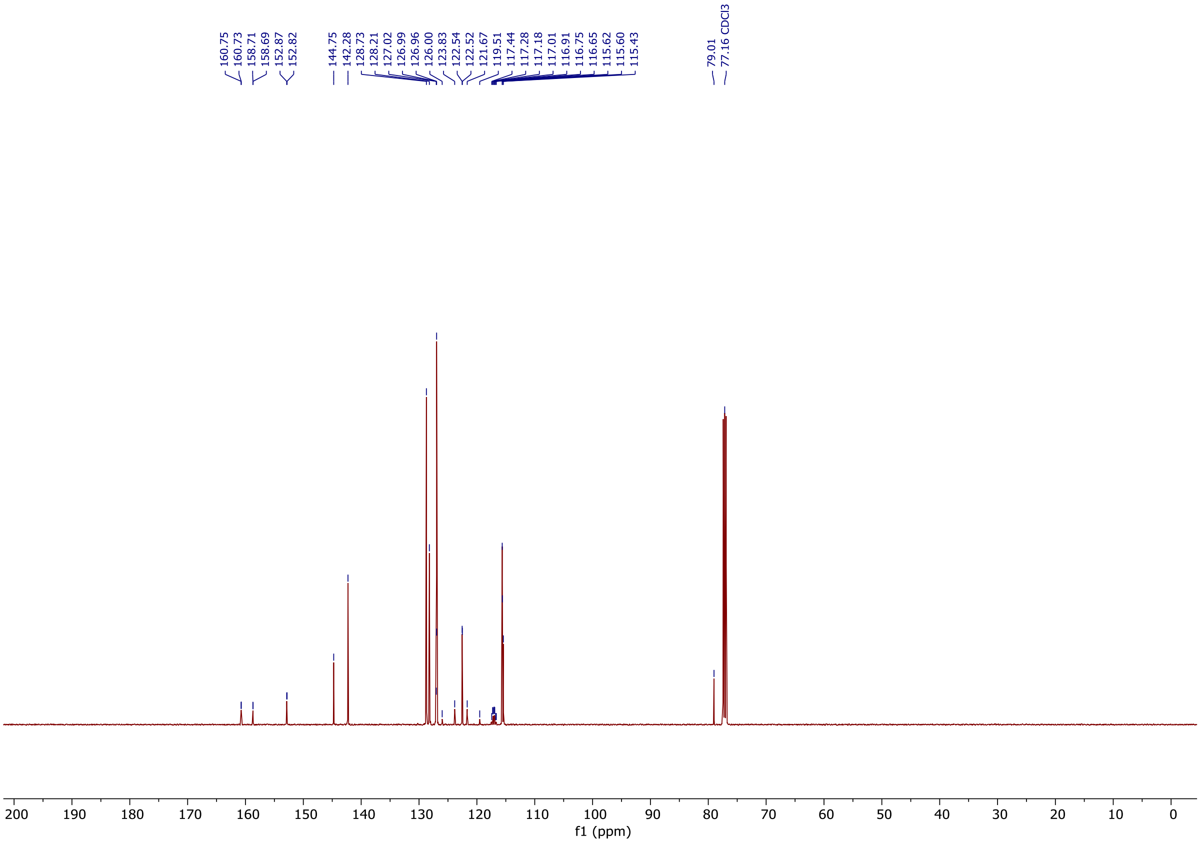

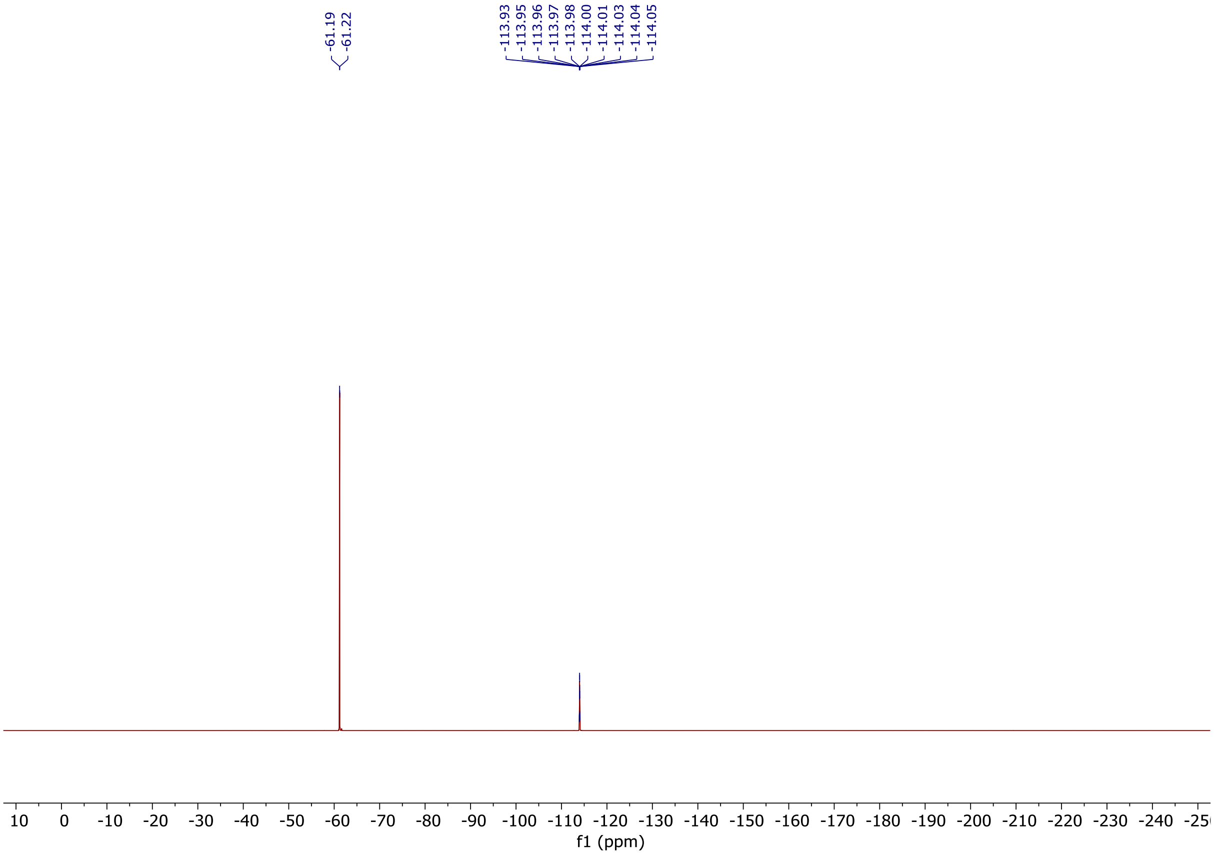

-61.19
-61.22
-113.93
-113.95
-113.96
-113.97
-113.98
-114.00
-114.01
-114.03
-114.04
-114.05
10 0 -10 -20 -30 -40 -50 -60 -70 -80 -90 -100 -110 -120 -130 -140 -150 -160 -170 -180 -190 -200 -210 -220 -230 -240
f1 (ppm)

6,6'-((1*E*,1'*E*)-(((1*R*,2*R*)-1,2-Di(naphthalen-1-yl)ethane-1,2-diyl)bis(azaneylylidene))bis(methaneylylidene))bis(2,4-di-*tert*-butylphenol) (**(*R*,*R*)-L-3**)

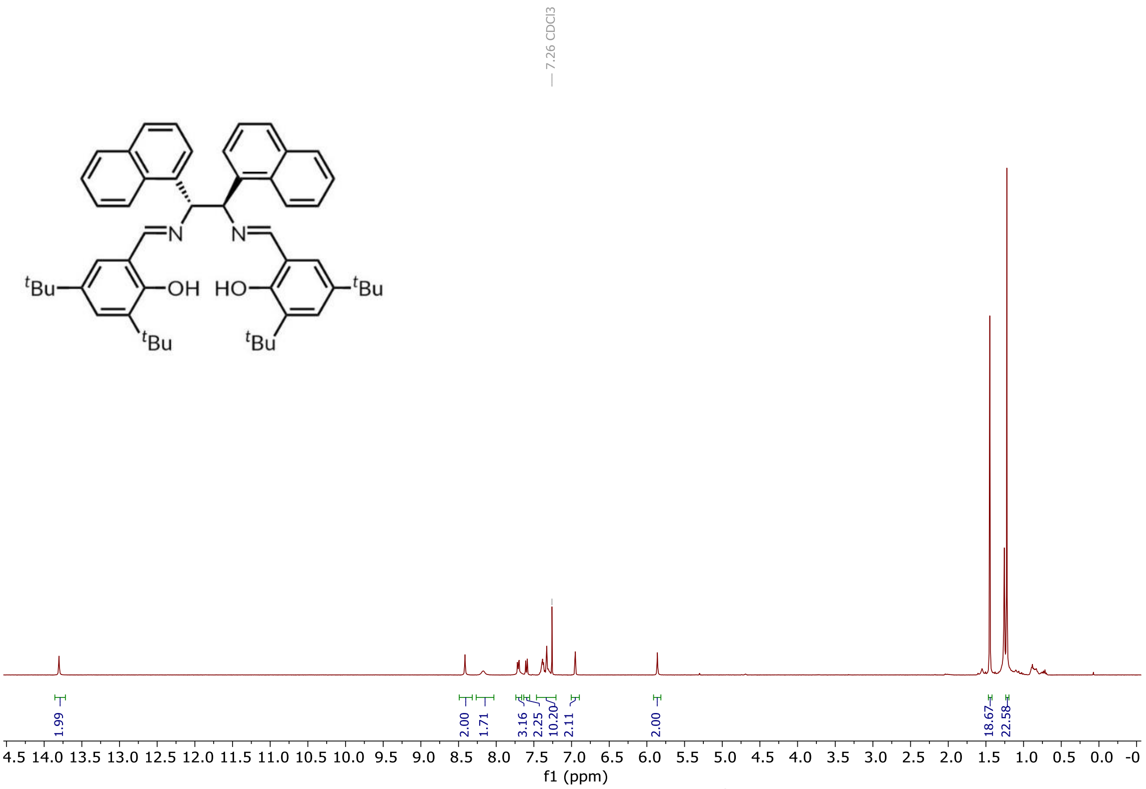

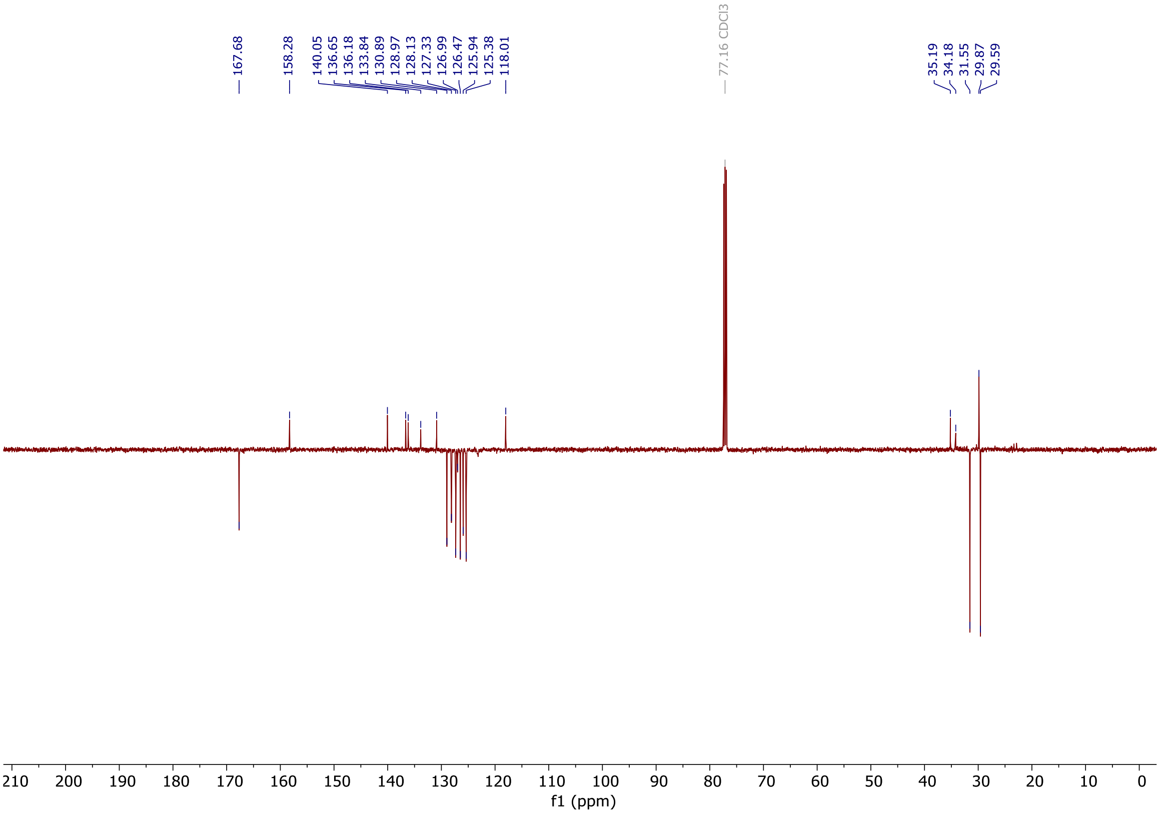

6,6'-((1*E*,1'*E*)-(((1*R*,2*R*)-1,2-Bis(4-methoxyphenyl)ethane-1,2-diyl)bis(azaneylylidene))bis(methaneylylidene))bis(2,4-di-*tert*-butylphenol) (**(*R*,*R*)-L-4**)

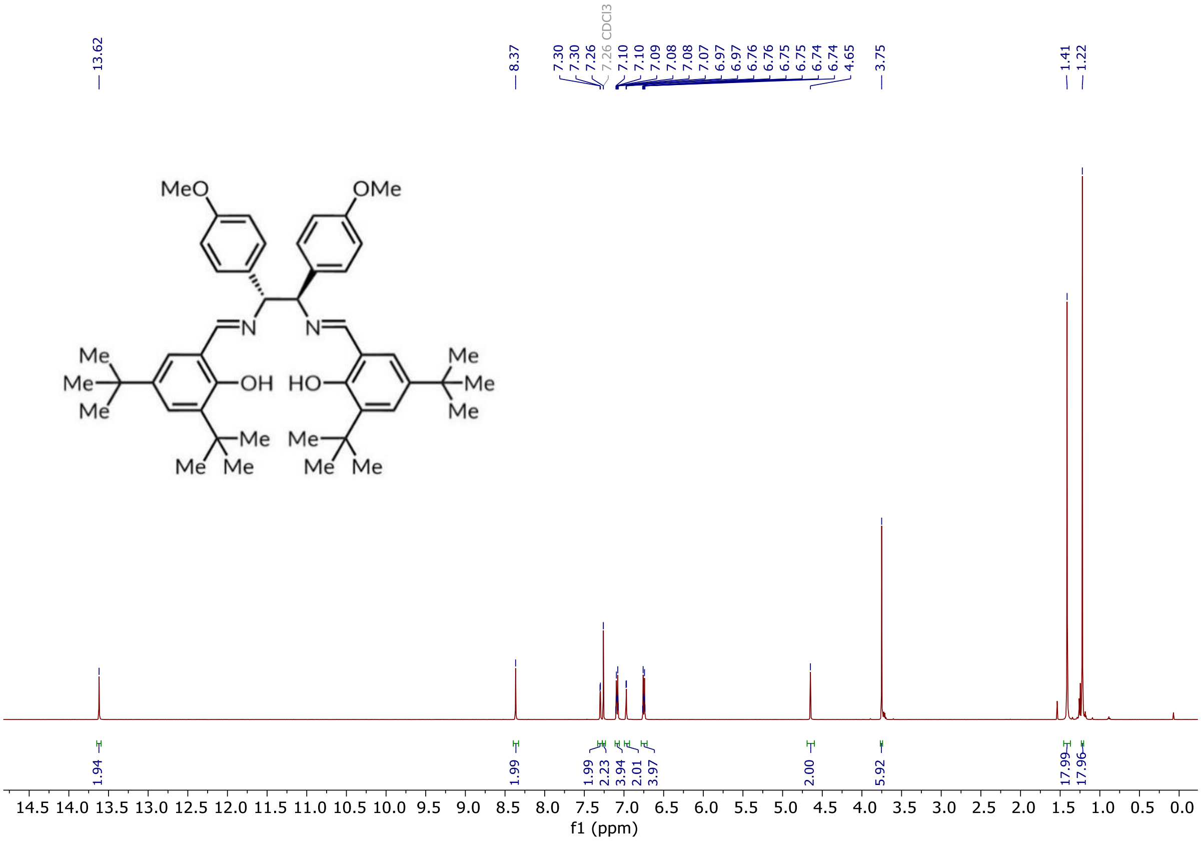

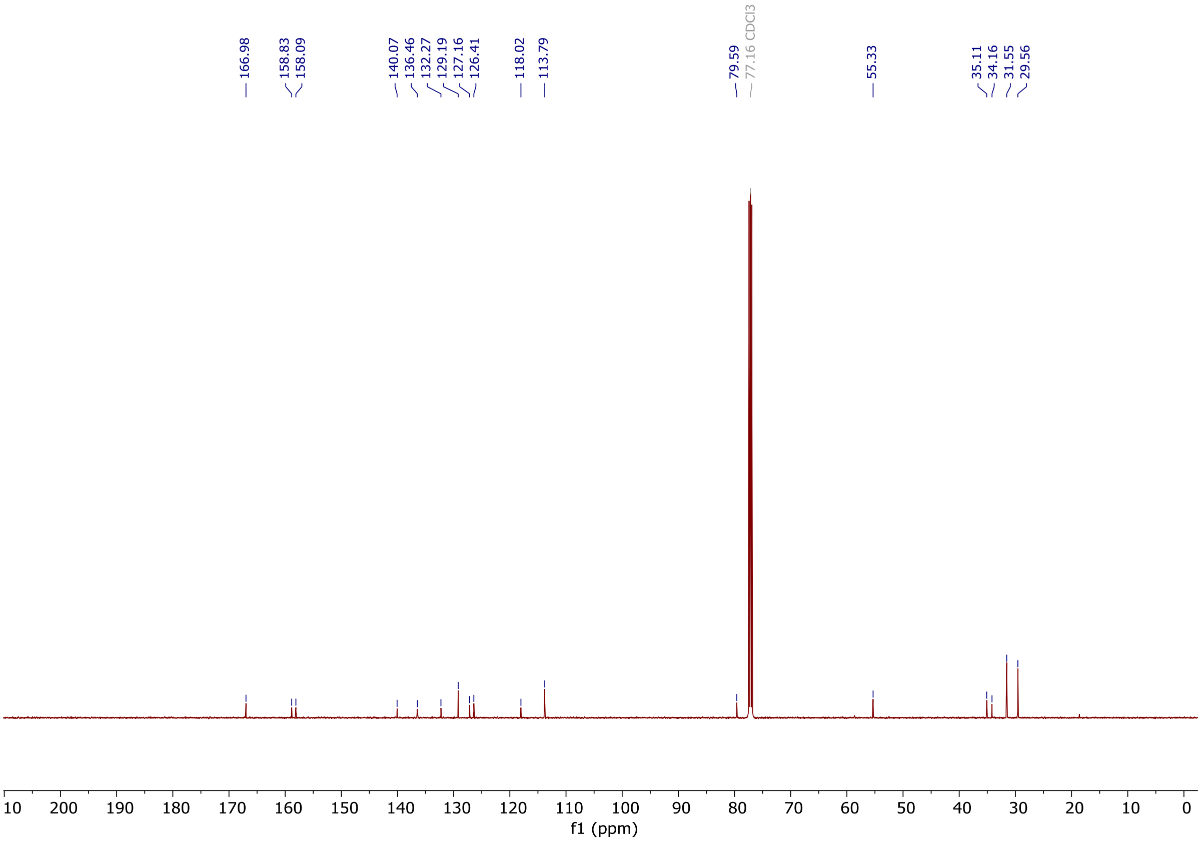

6,6'-((1*E*,1'*E*)-(((1*R*,2*R*)-1,2-Dimesitylethane-1,2-diyl)bis(azaneylylidene))bis(methaneylylidene))bis(2,4-di-*tert*-butylphenol) (**(*R*,*R*)-L-5**)

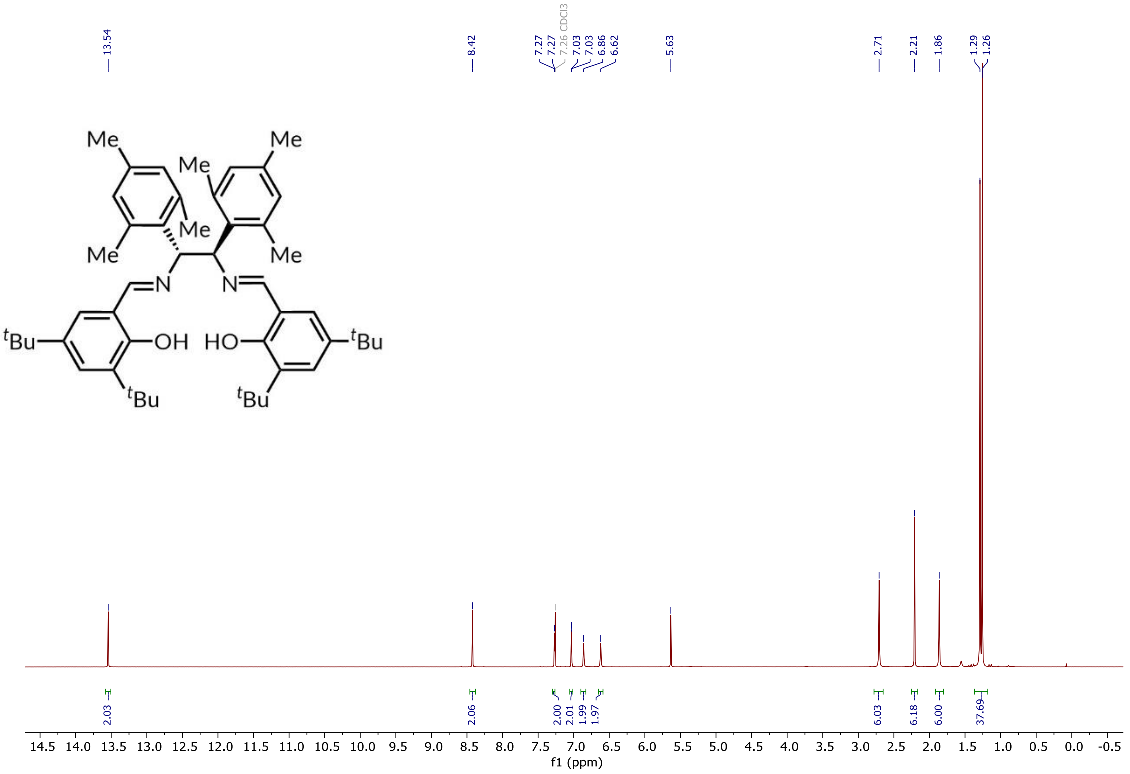

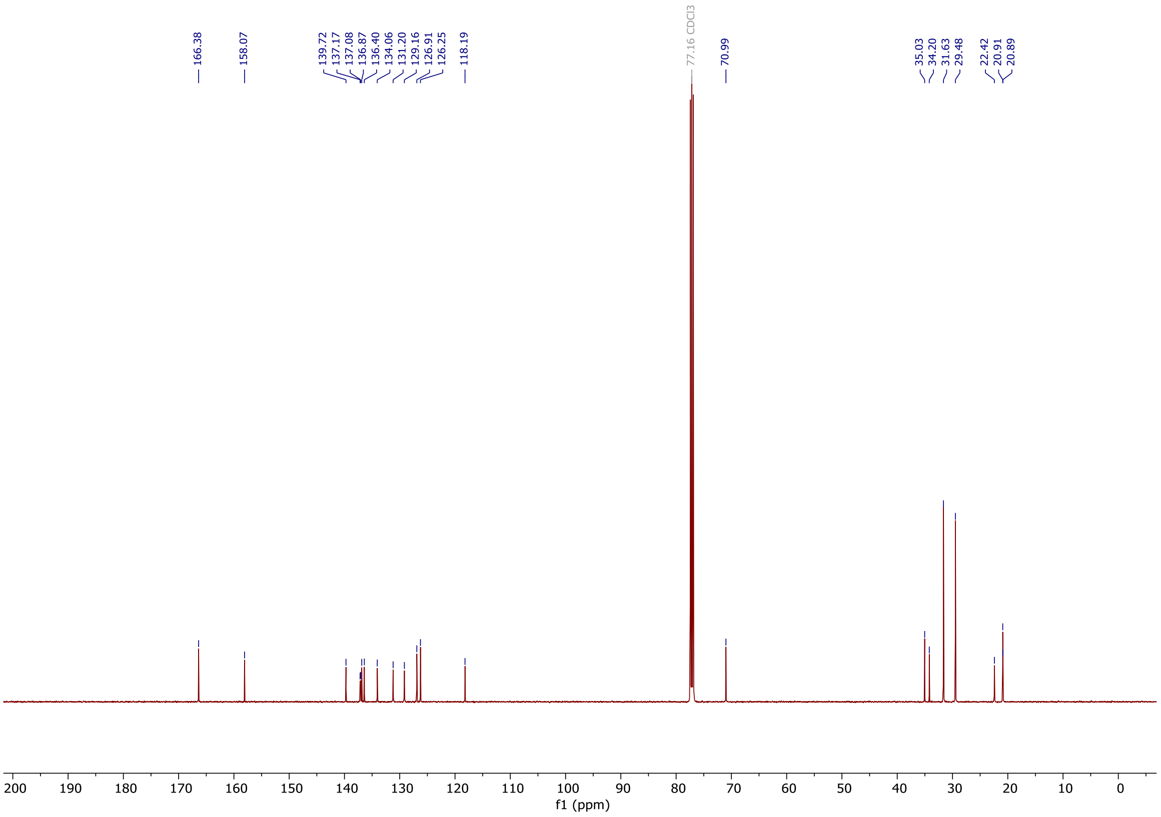

6,6'-((1*E*,1'*E*)-((2-Methylpropane-1,2-diyl)bis(azaneylylidene))bis(methaneylylidene))bis (2,4-di-*tert*-butylphenol) (**L-6**)

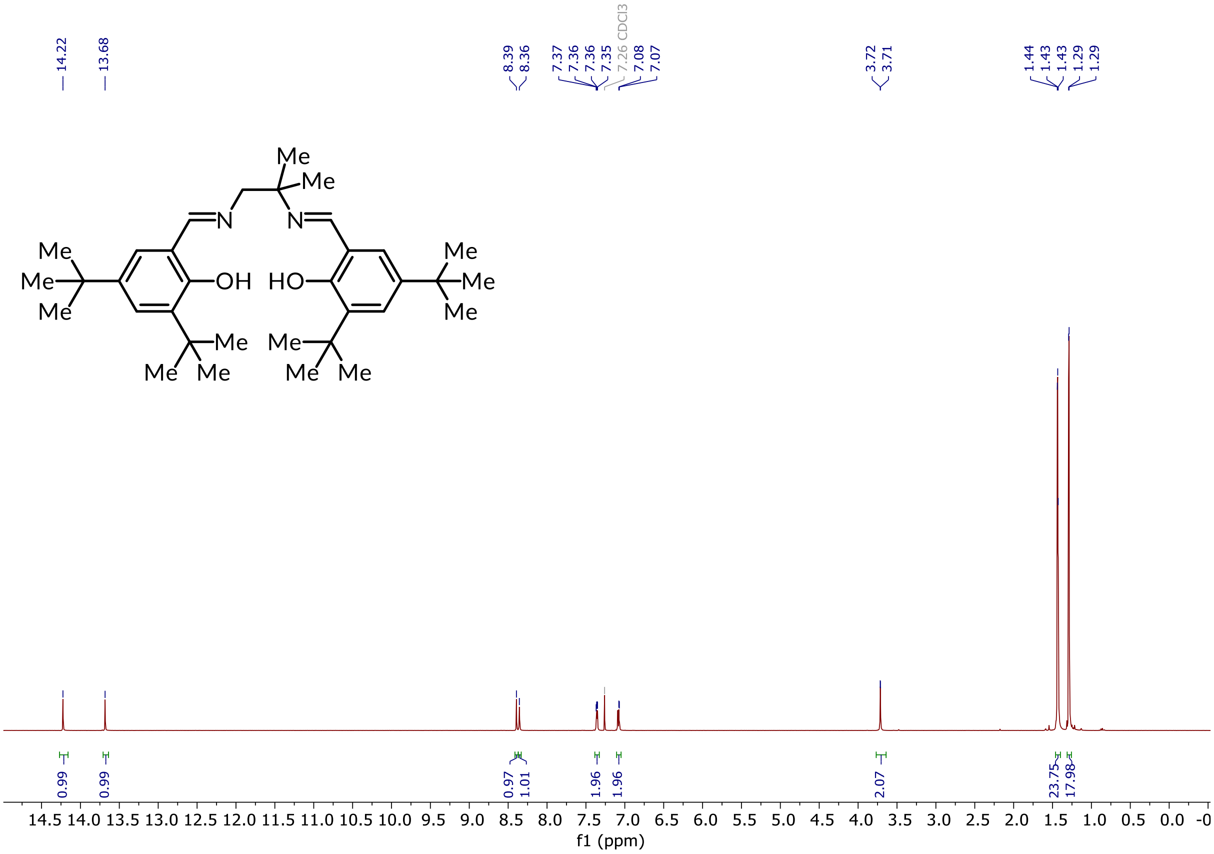

(*R*)-1,2-Bis(4-fluorophenyl)propan-1-one ((***R***)-**2a**)

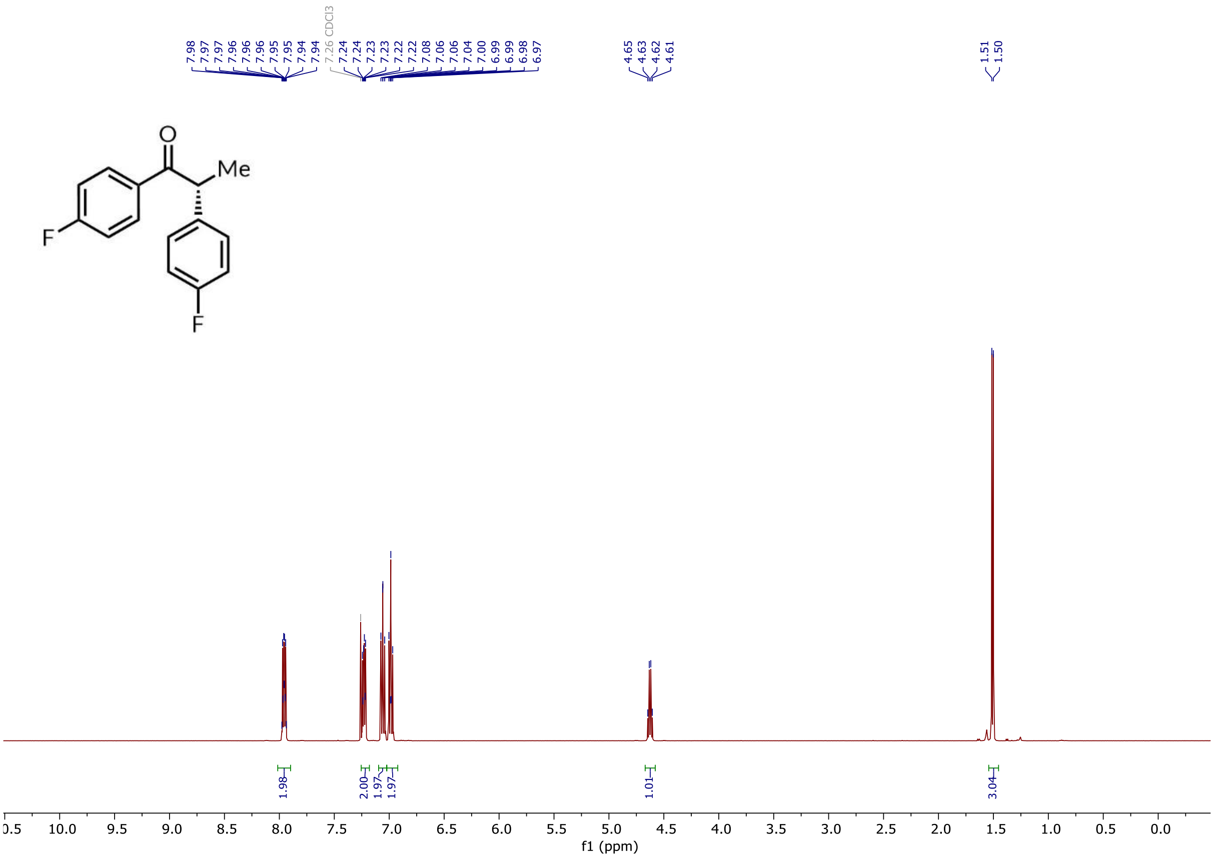

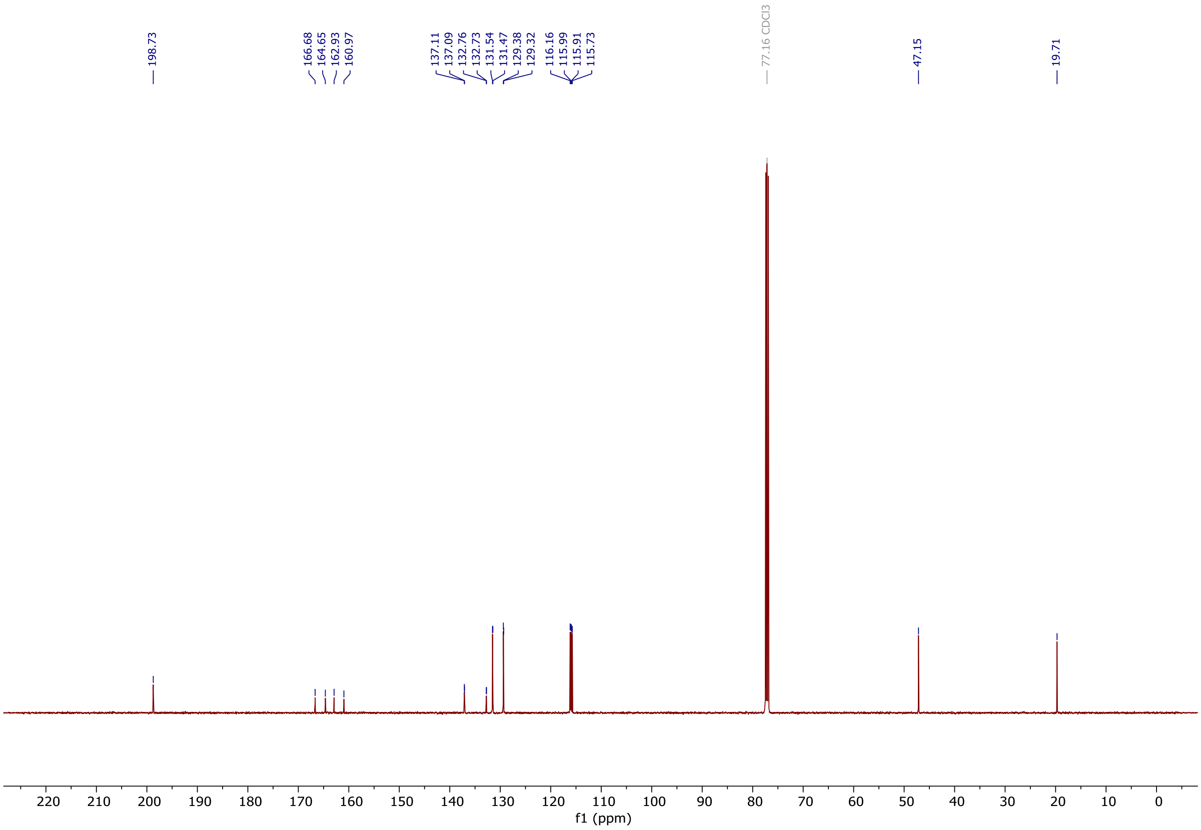

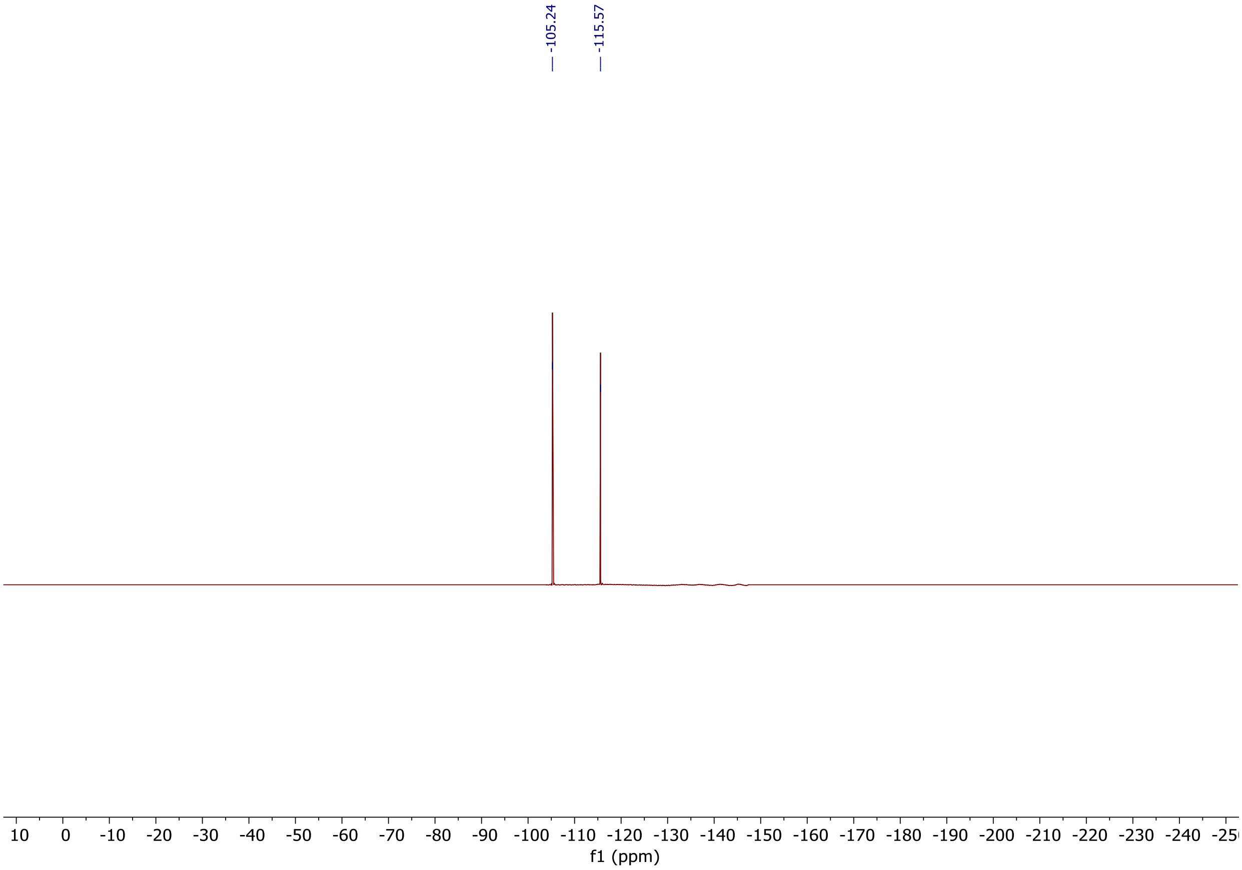

-105.24
-115.57
10 0 -10 -20 -30 -40 -50 -60 -70 -80 -90 -100 -110 -120 -130 -140 -150 -160 -170 -180 -190 -200 -210 -220 -230 -240 -25
f1 (ppm)

(*R*)-1,2-Diphenylpropan-1-one (**(*R*)-2b**)

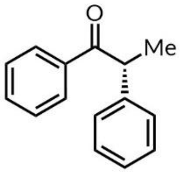

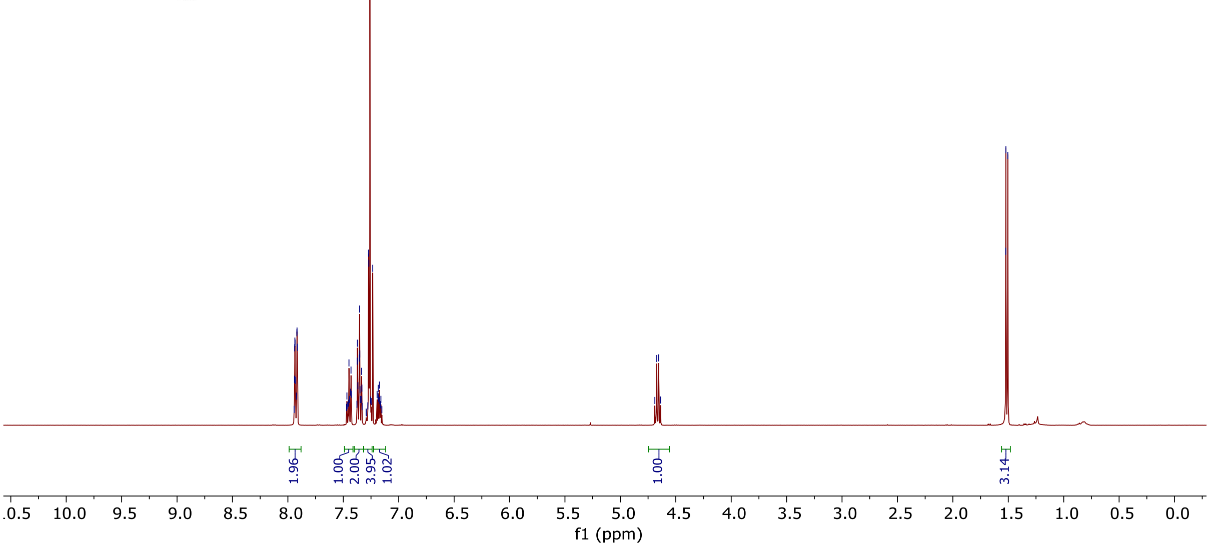

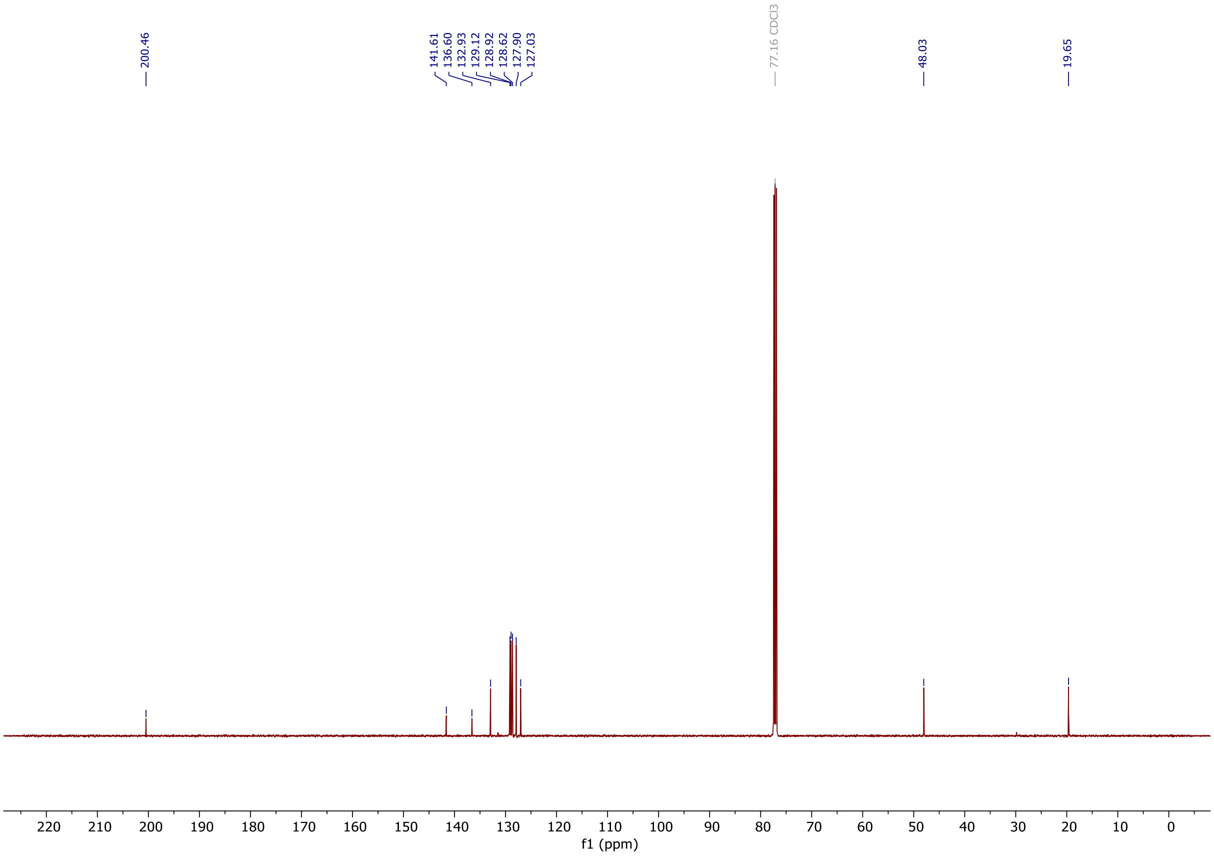

(*R*)-1,2-Bis(4-methylphenyl)propan-1-one (**(*R*)-2c**)

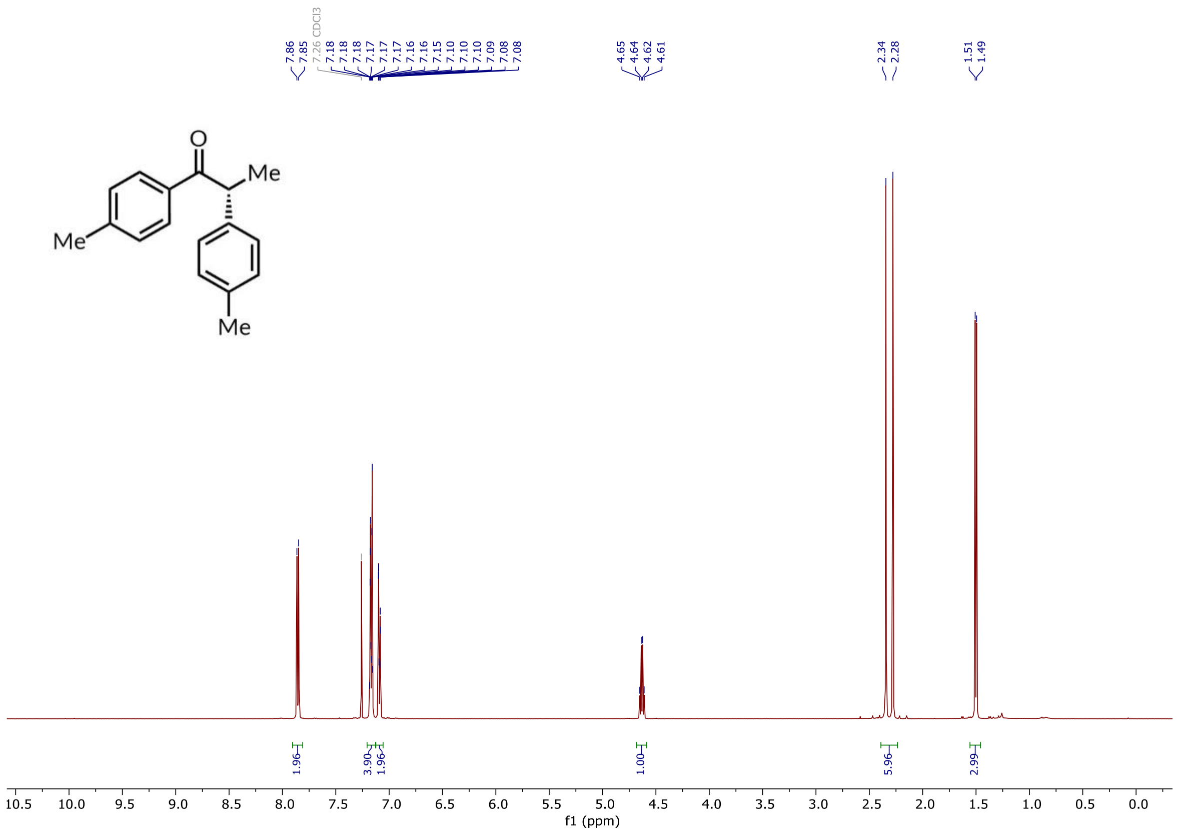

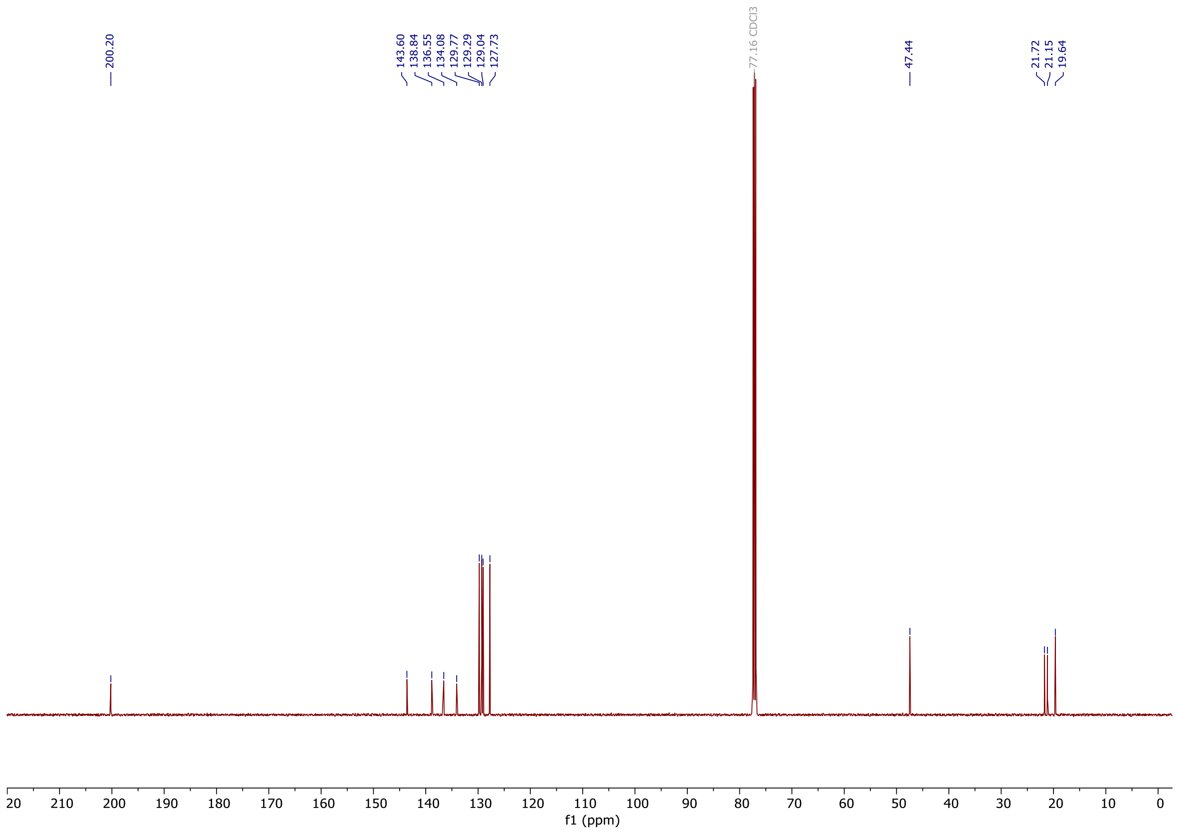

(*R*)-1,2-Bis(4-(isopropyl)phenyl)propan-1-one (**(*R*)-2d**)

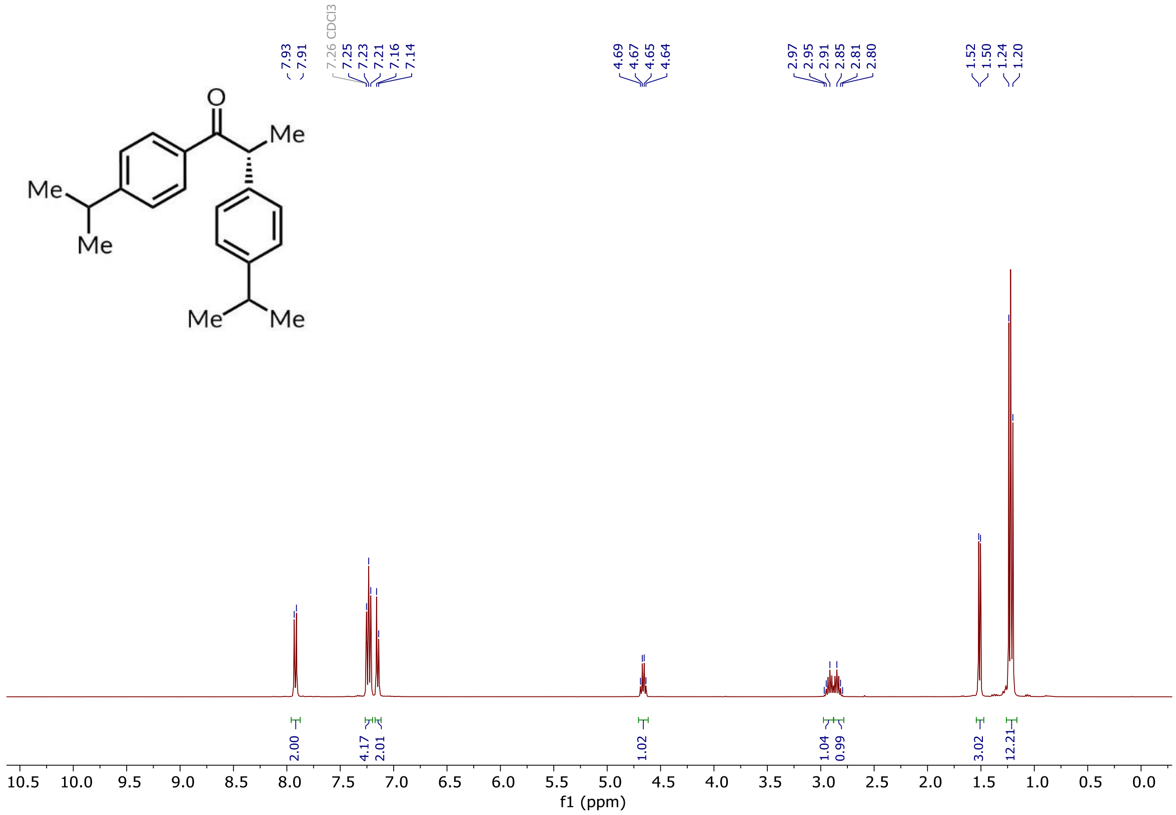

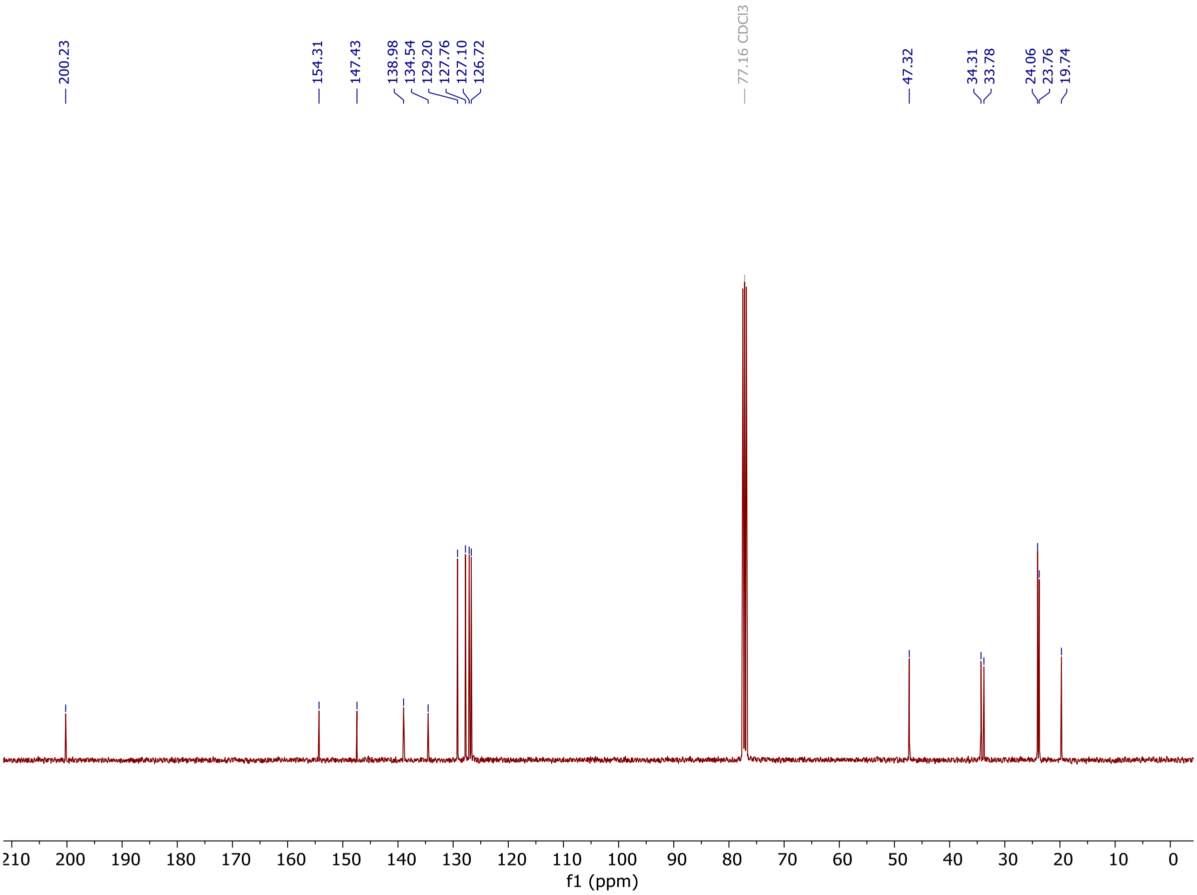

(*R*)-1,2-Bis(4-(*tert*-butyl)phenyl)propan-1-one (**(*R*)-2e**)

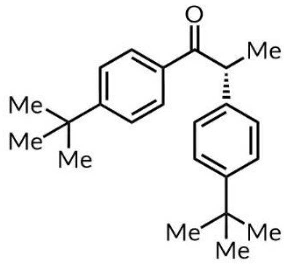

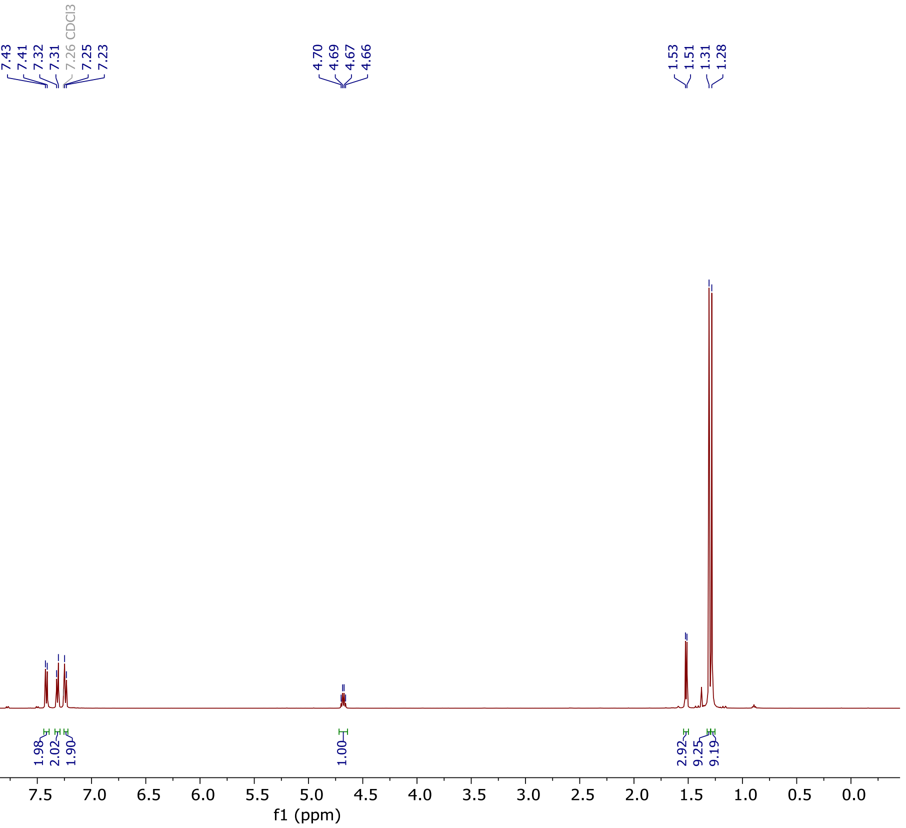

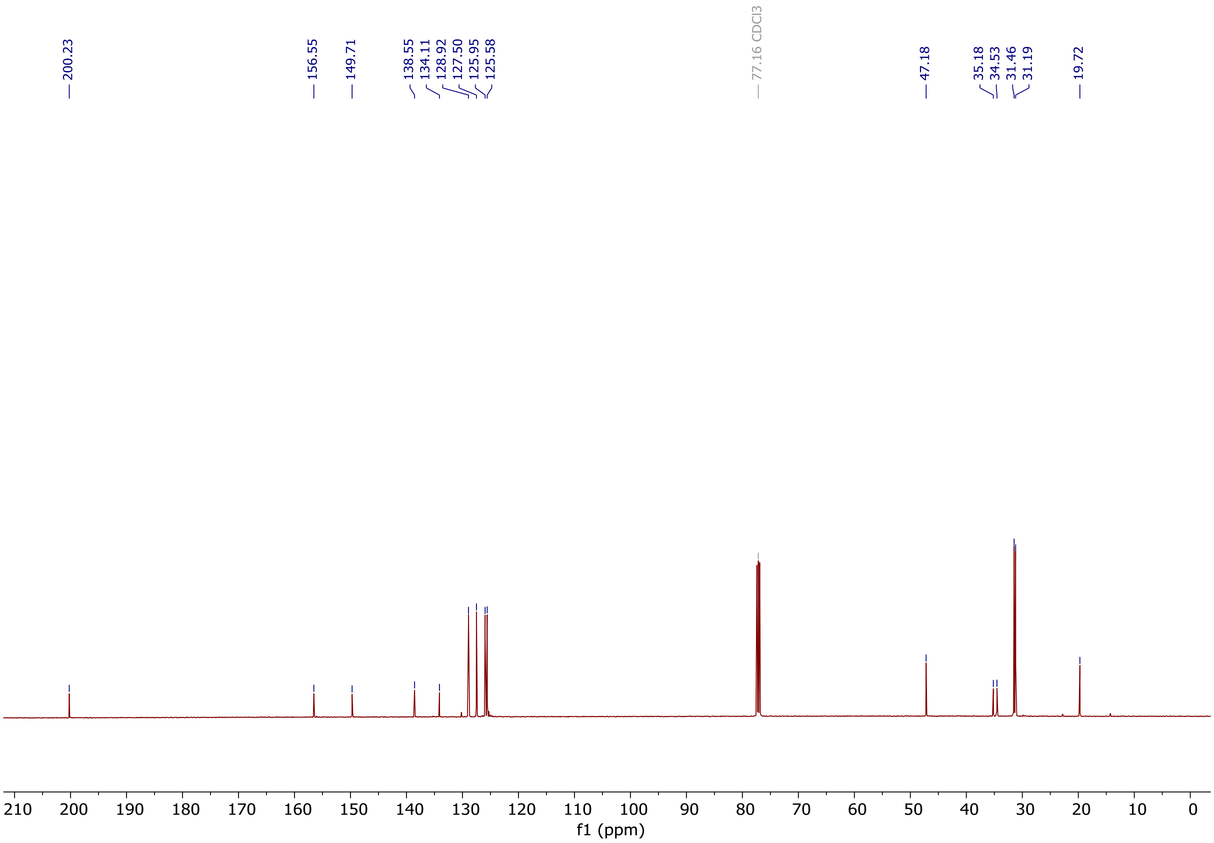

(*R*)-1,2-Di([1,1′-biphenyl]-4-yl)propan-1-one (**(*R*)-2f**)

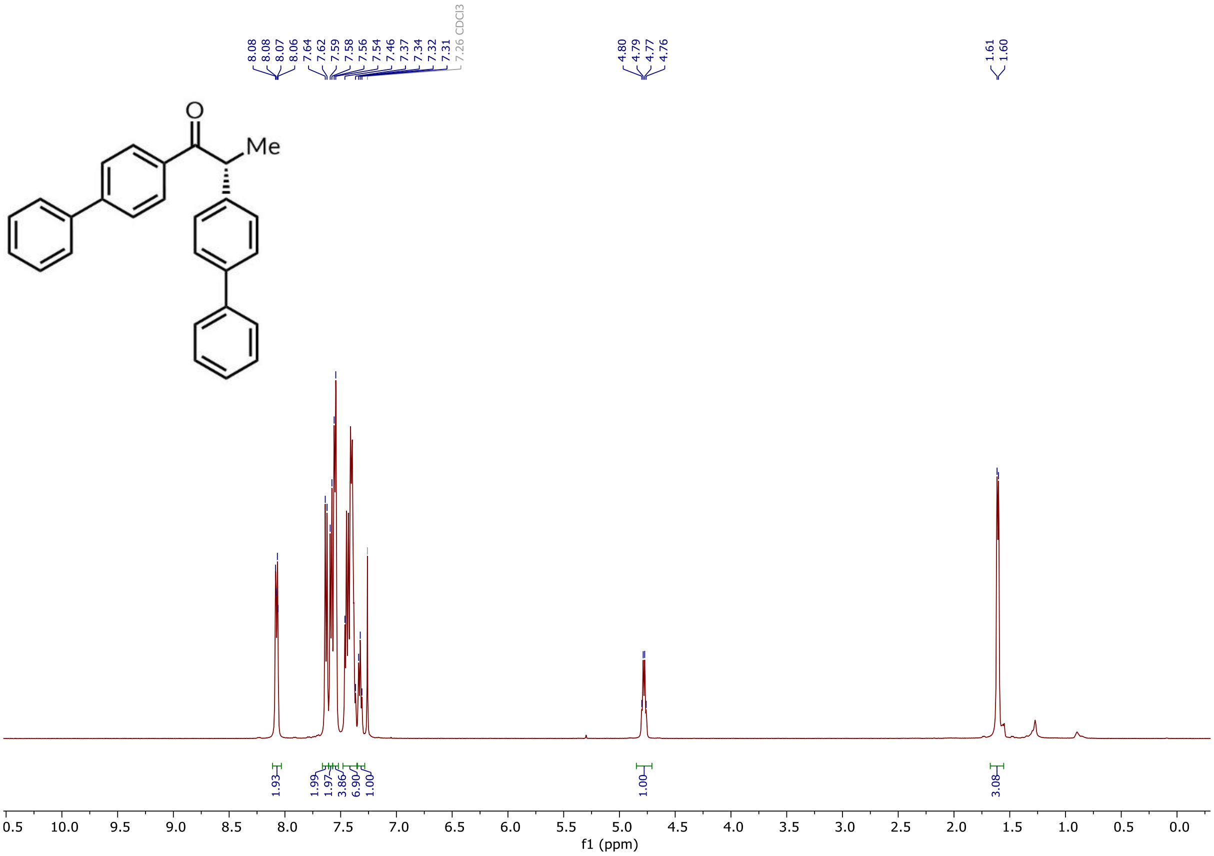

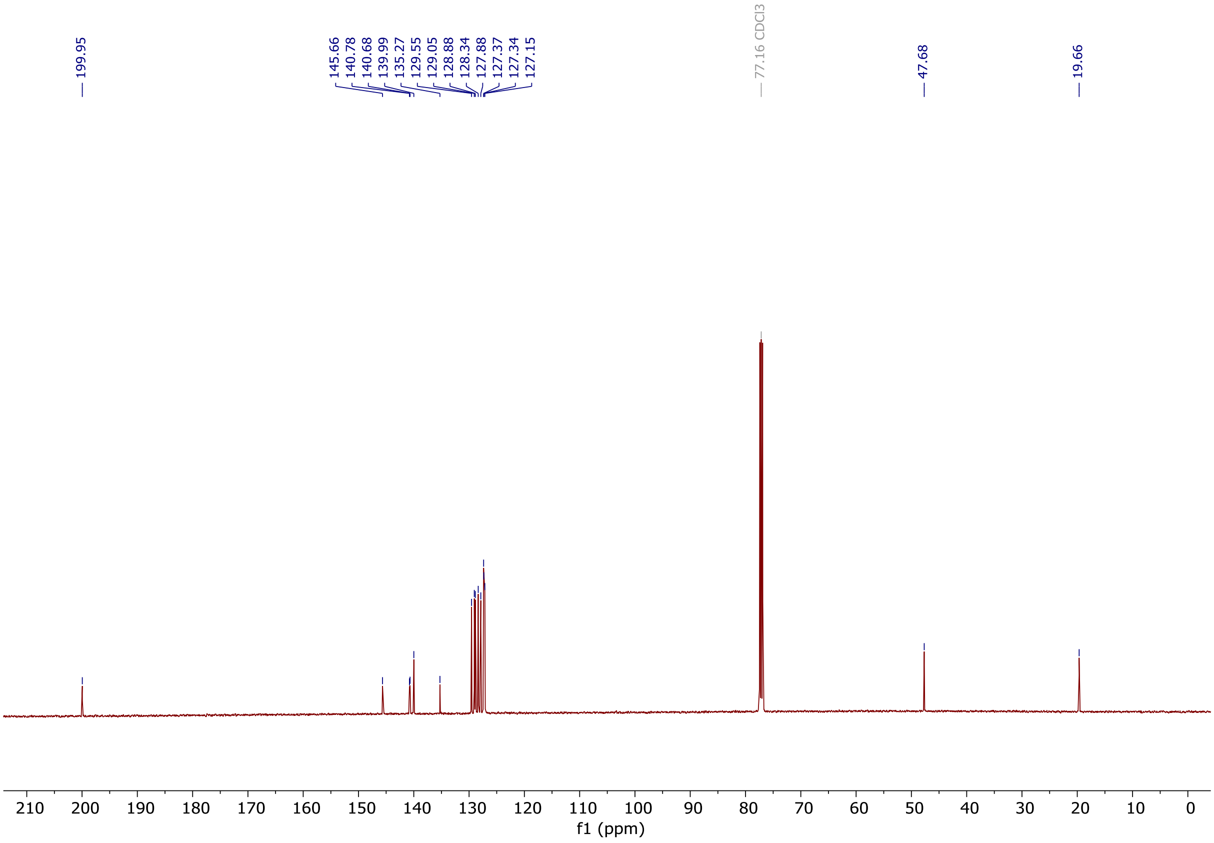

(*R*)-1,2-Bis(4-bromophenyl)propan-1-one (**(*R*)-2g**)

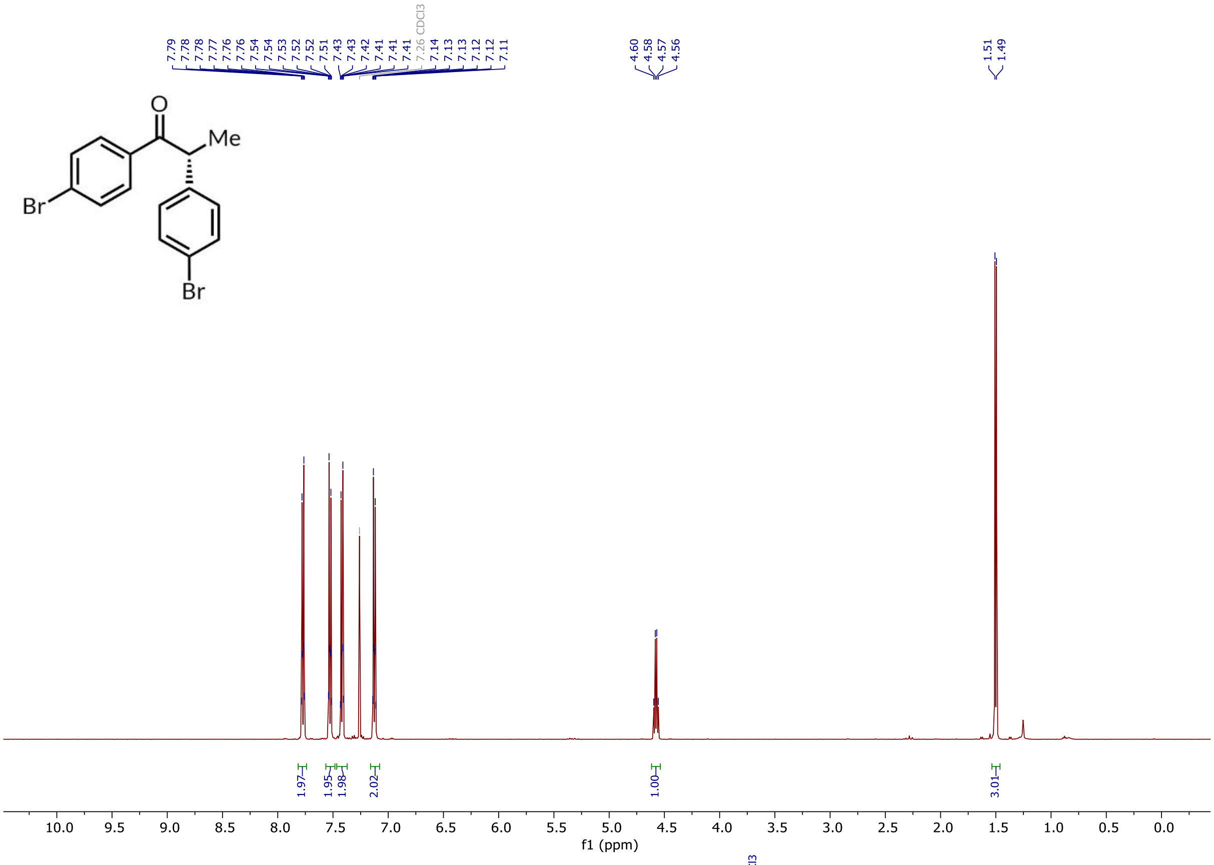

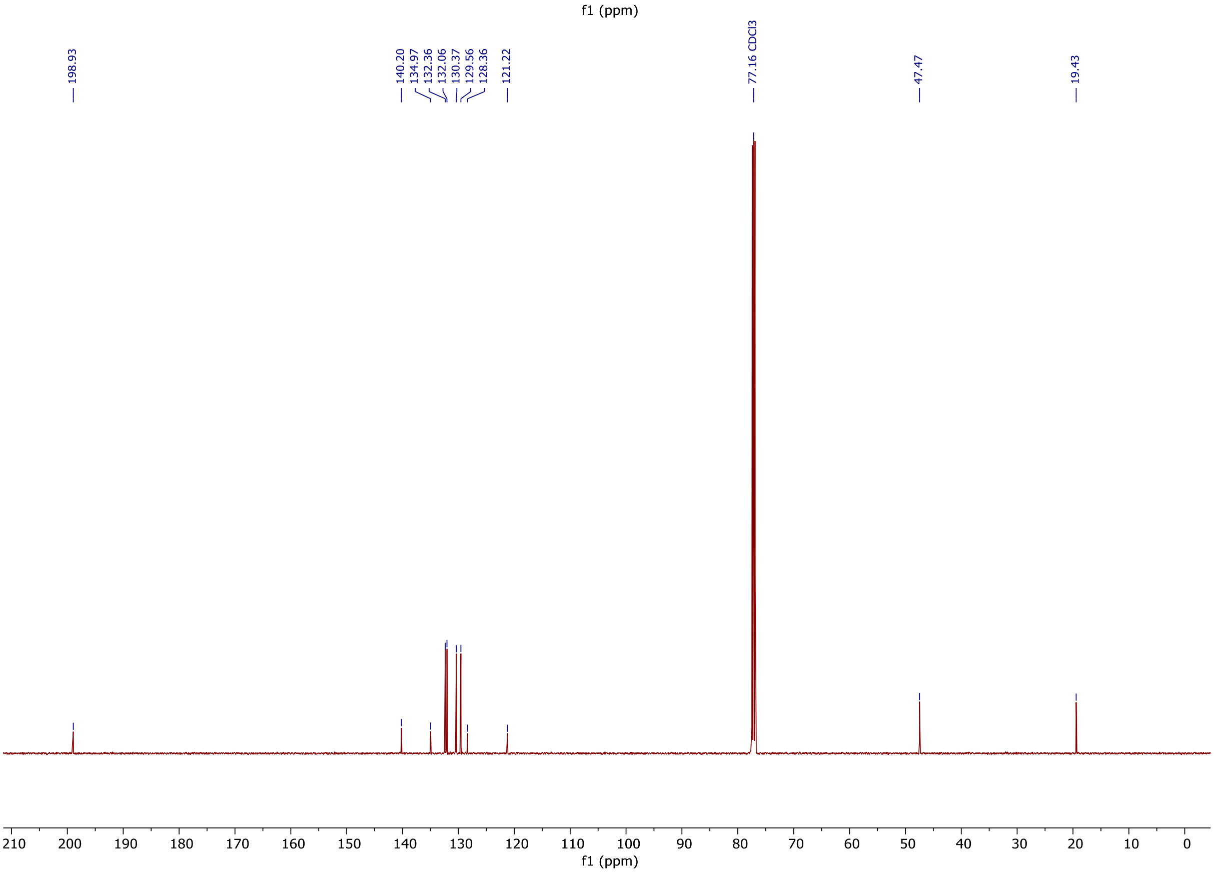

(*R*)-1,2-Bis(4-chlorophenyl)propan-1-one (**(*R*)-2h**)

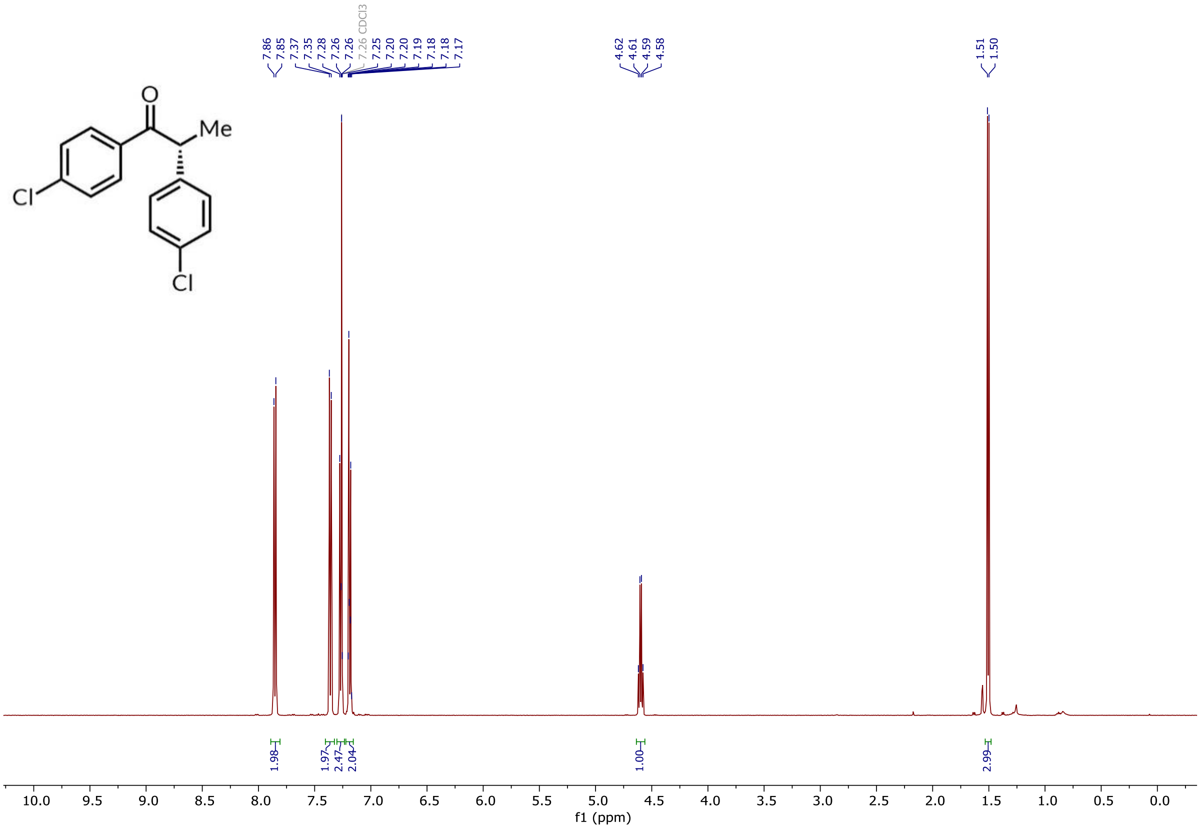

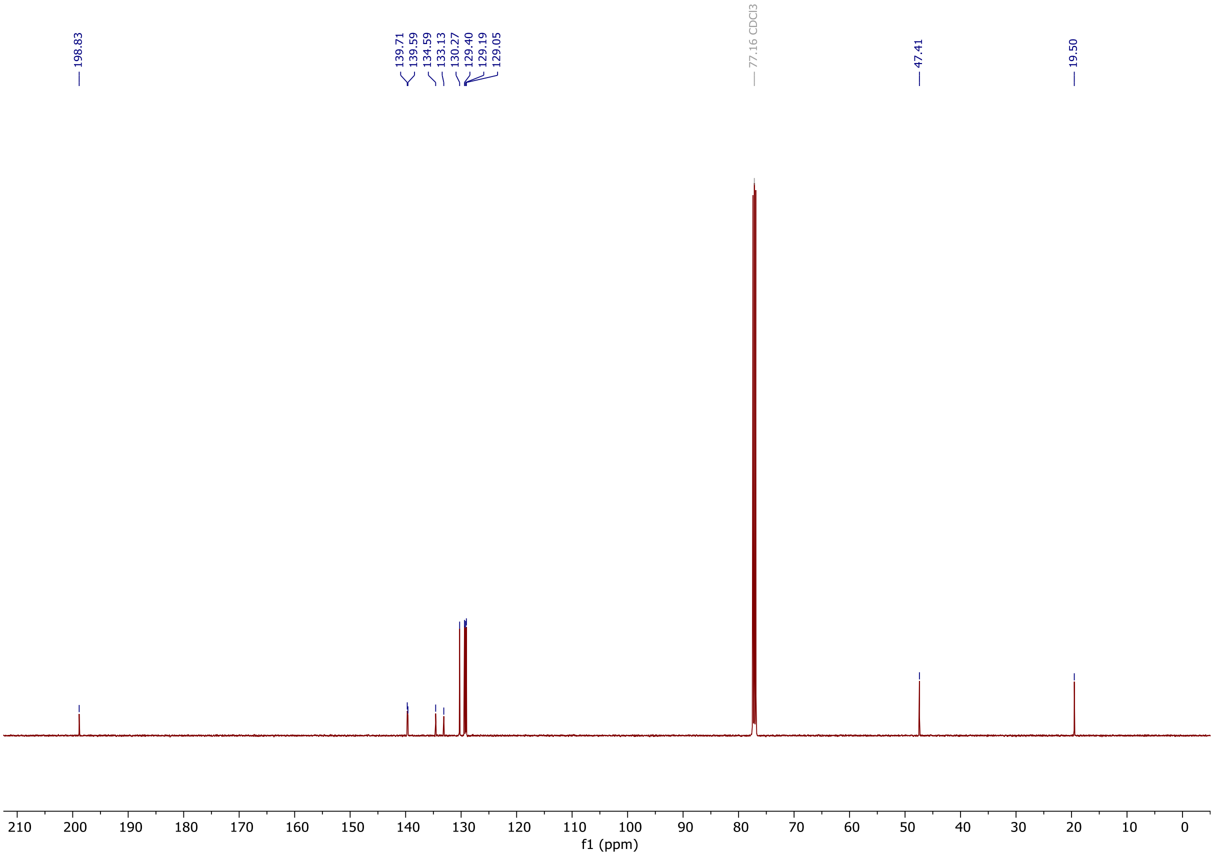

(*R*)-1,2-Bis(4-methoxyphenyl)propan-1-one (**(*R*)-2i**)

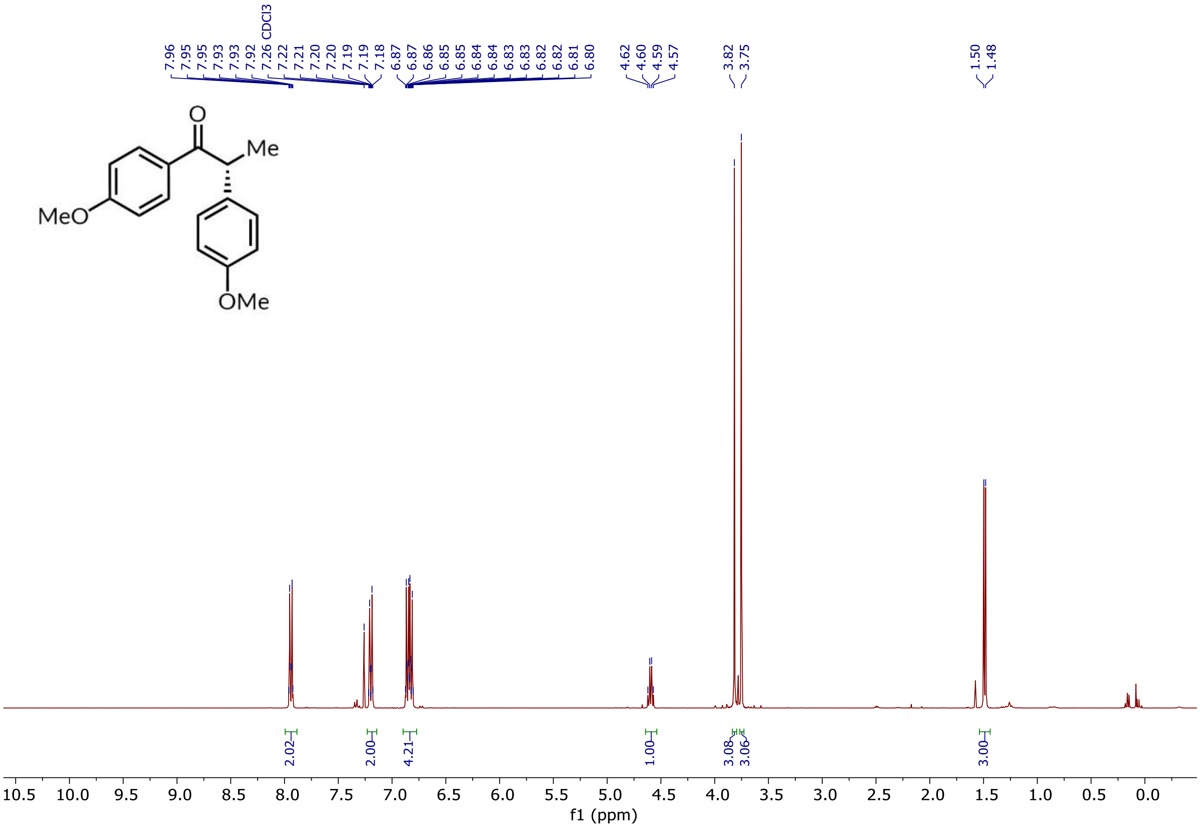

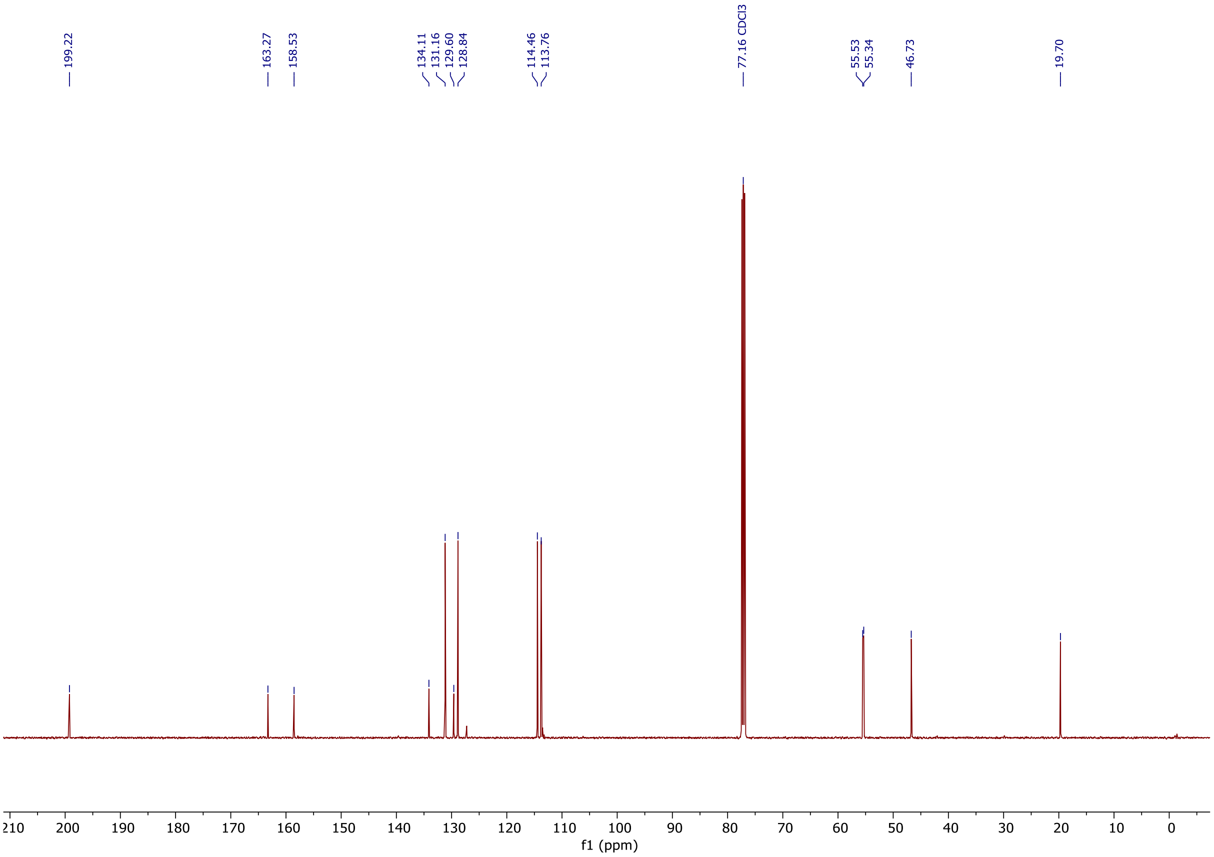

(*R*)-1,2-Bis(4-phenoxyphenyl)propan-1-one (**(*R*)-2j**)

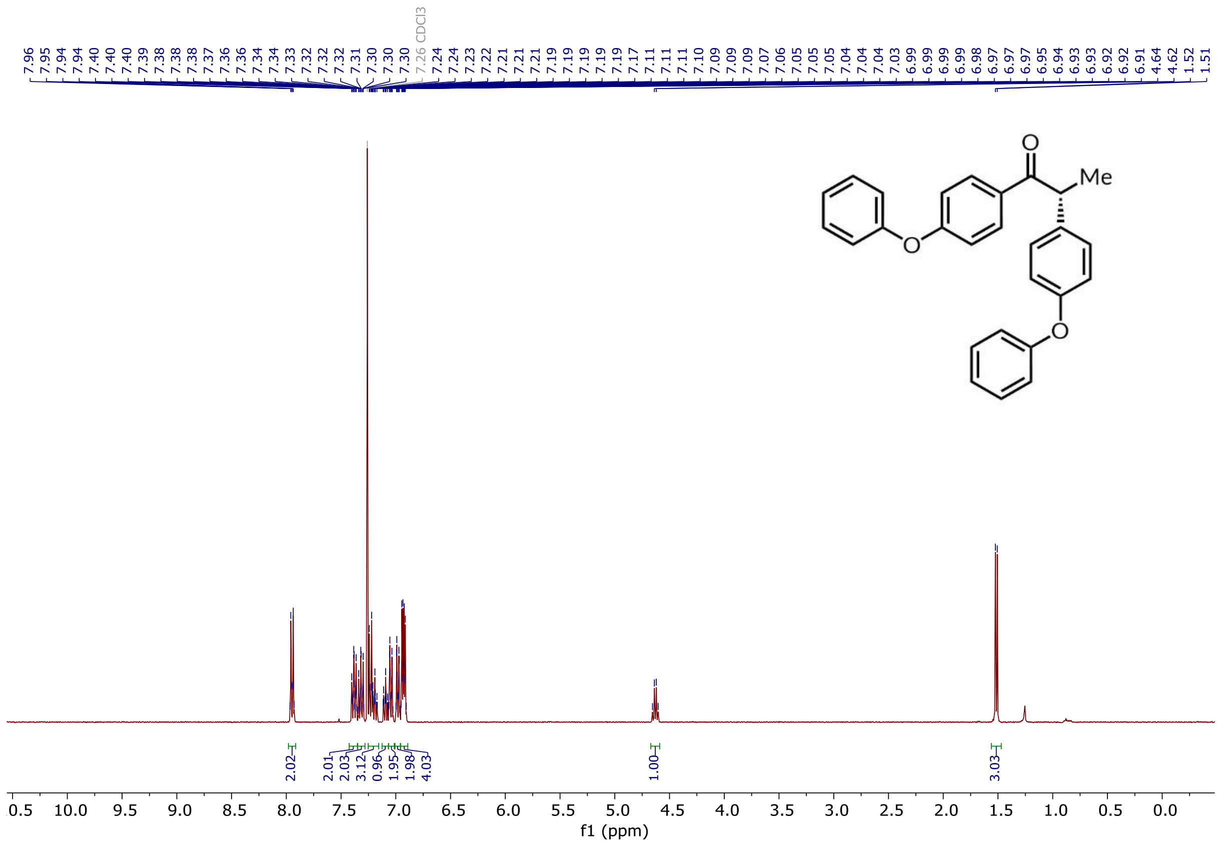

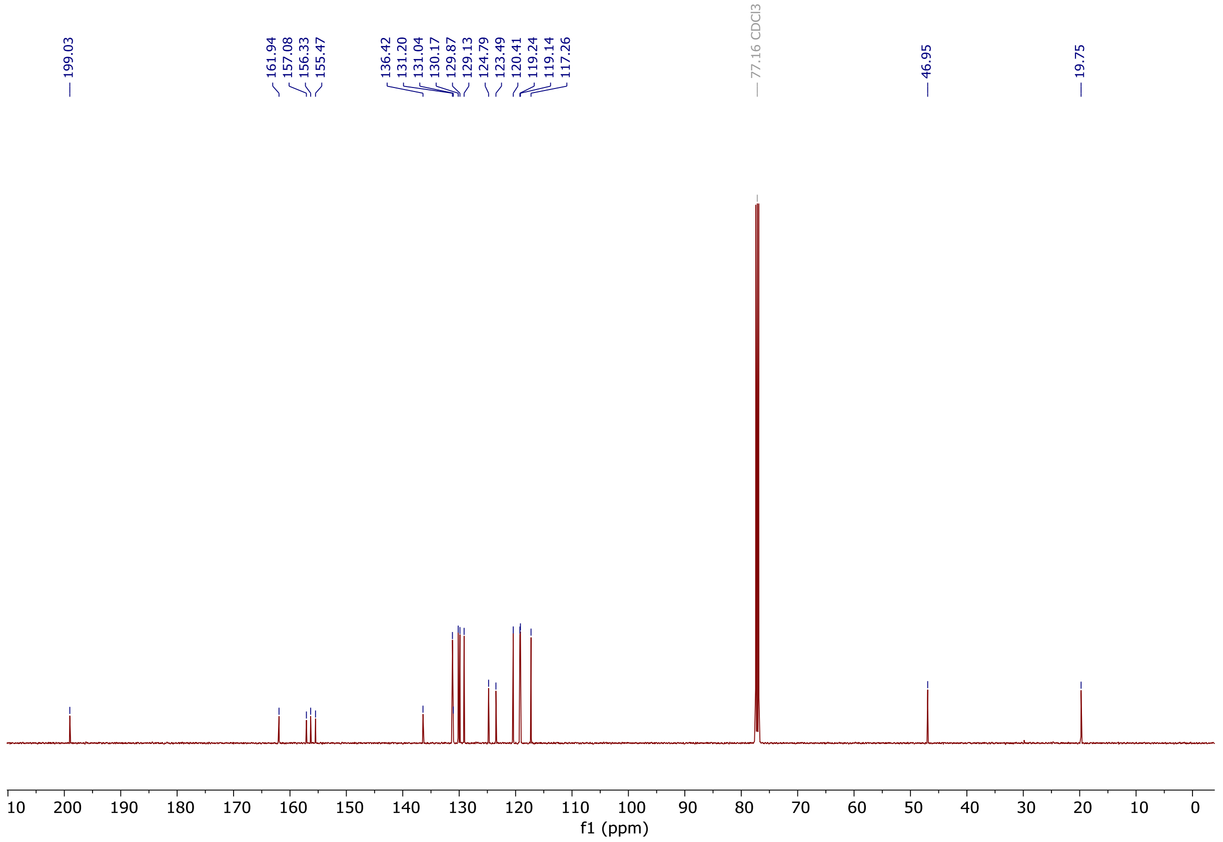

(*R*)-1,2-Bis(4-(methylthio)phenyl)propan-1-one ((***R***)-**2k**)

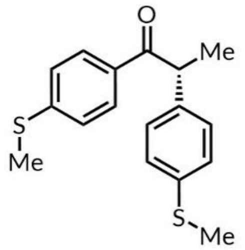

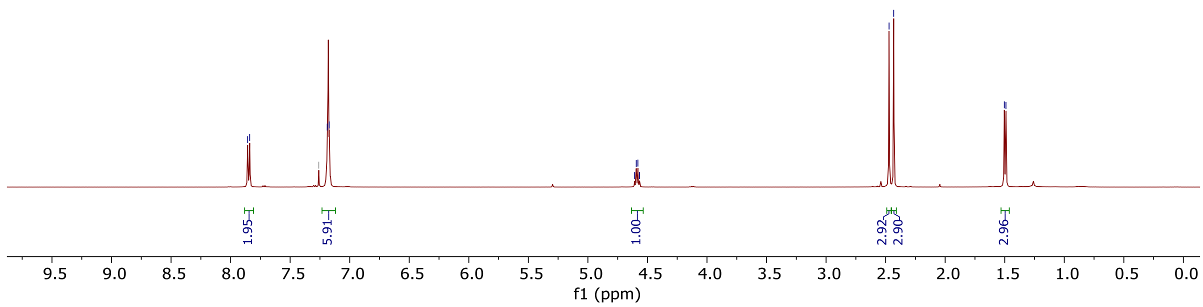

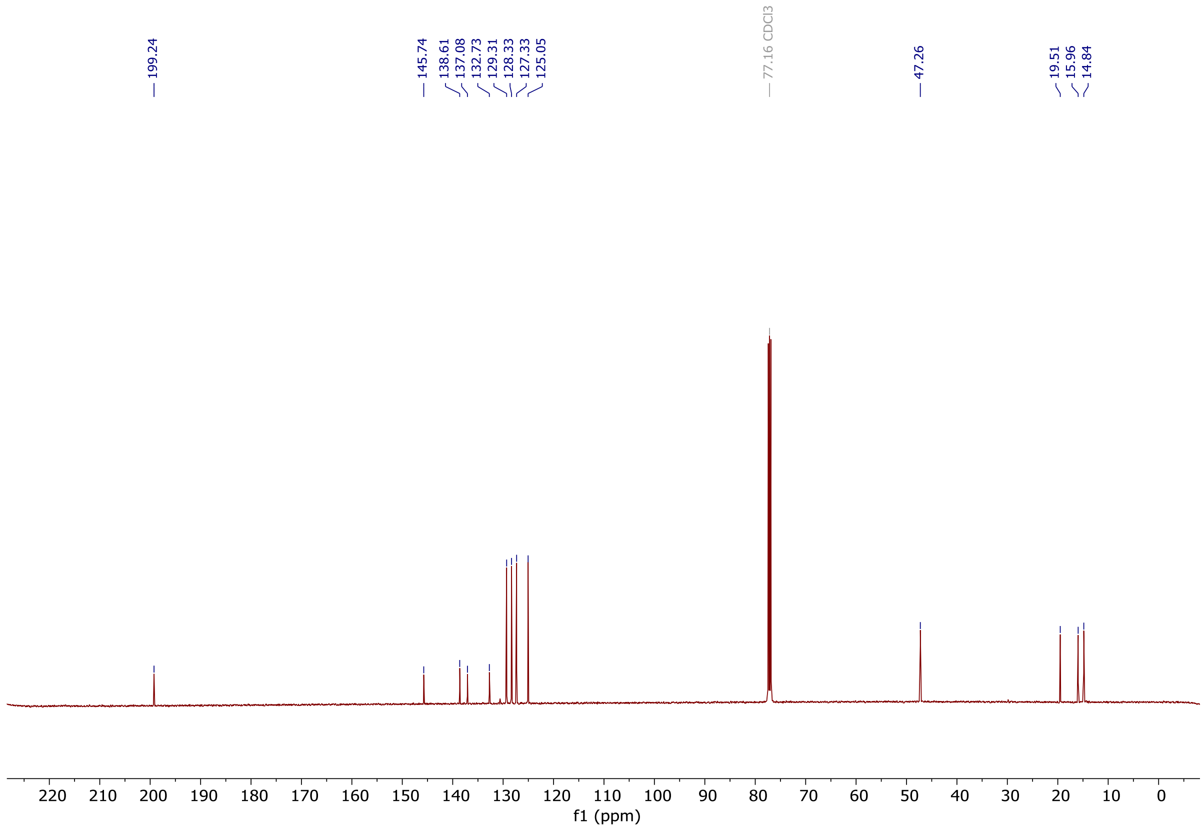

(*R*)-1,2-Bis(4-((trimethylsilyl)ethynyl)phenyl)propan-1-one (**(*R*)-2l**)

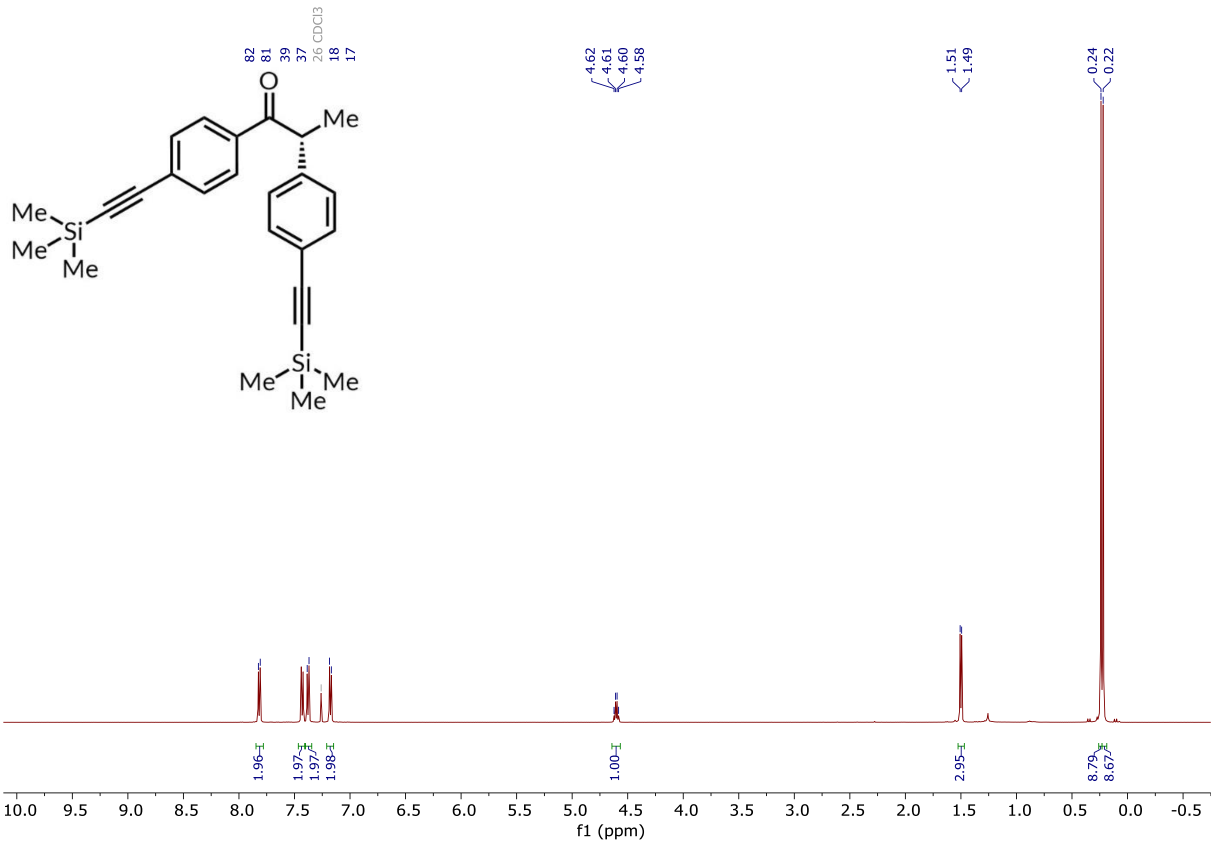

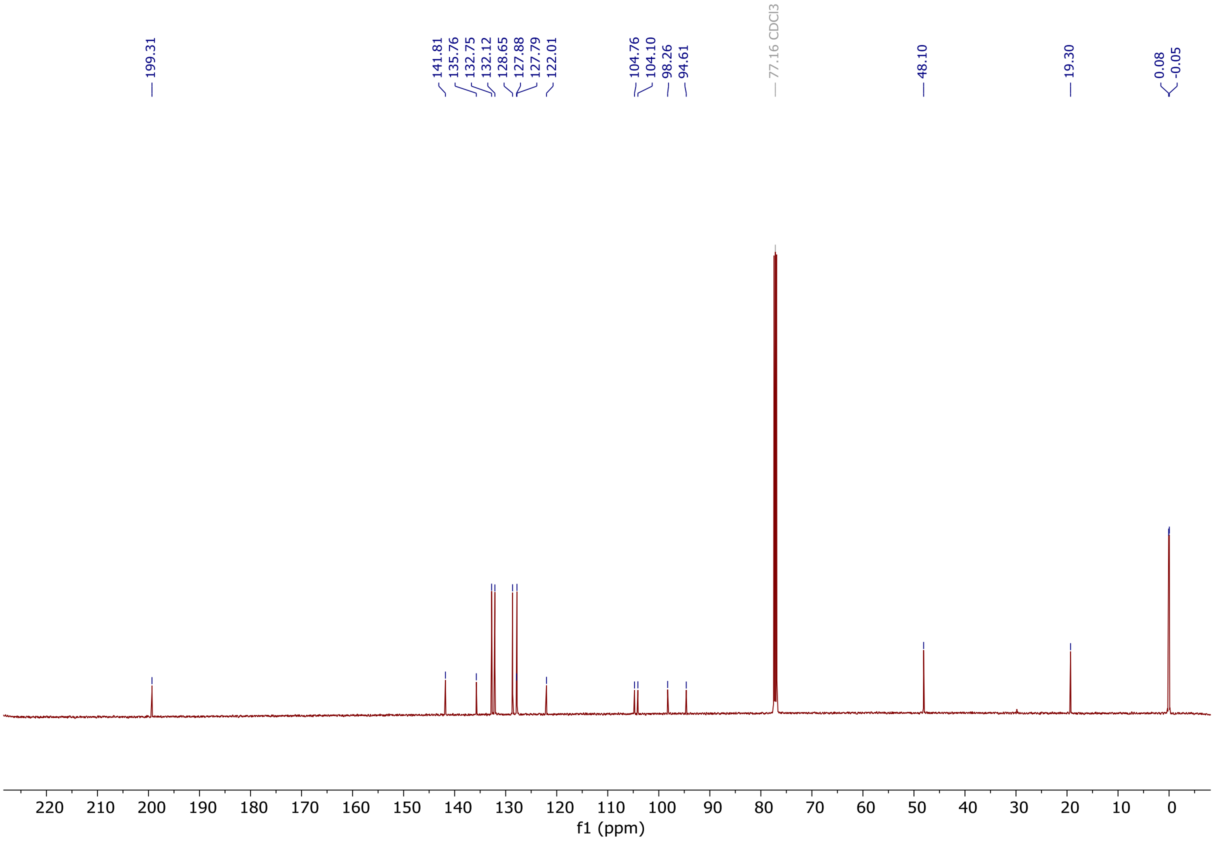

(*R*)-1,2-Bis(4-(trifluoromethyl)phenyl)propan-1-one (**(*R*)-2m**)

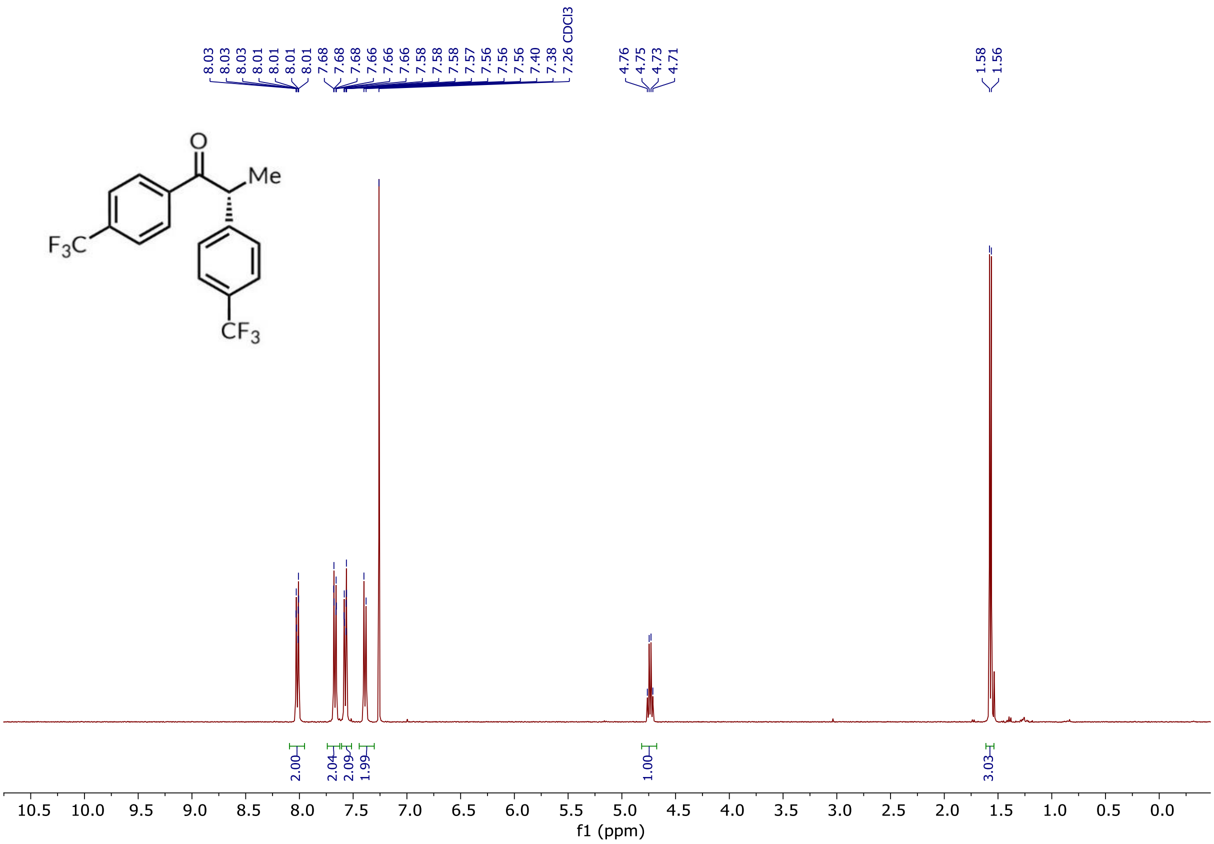

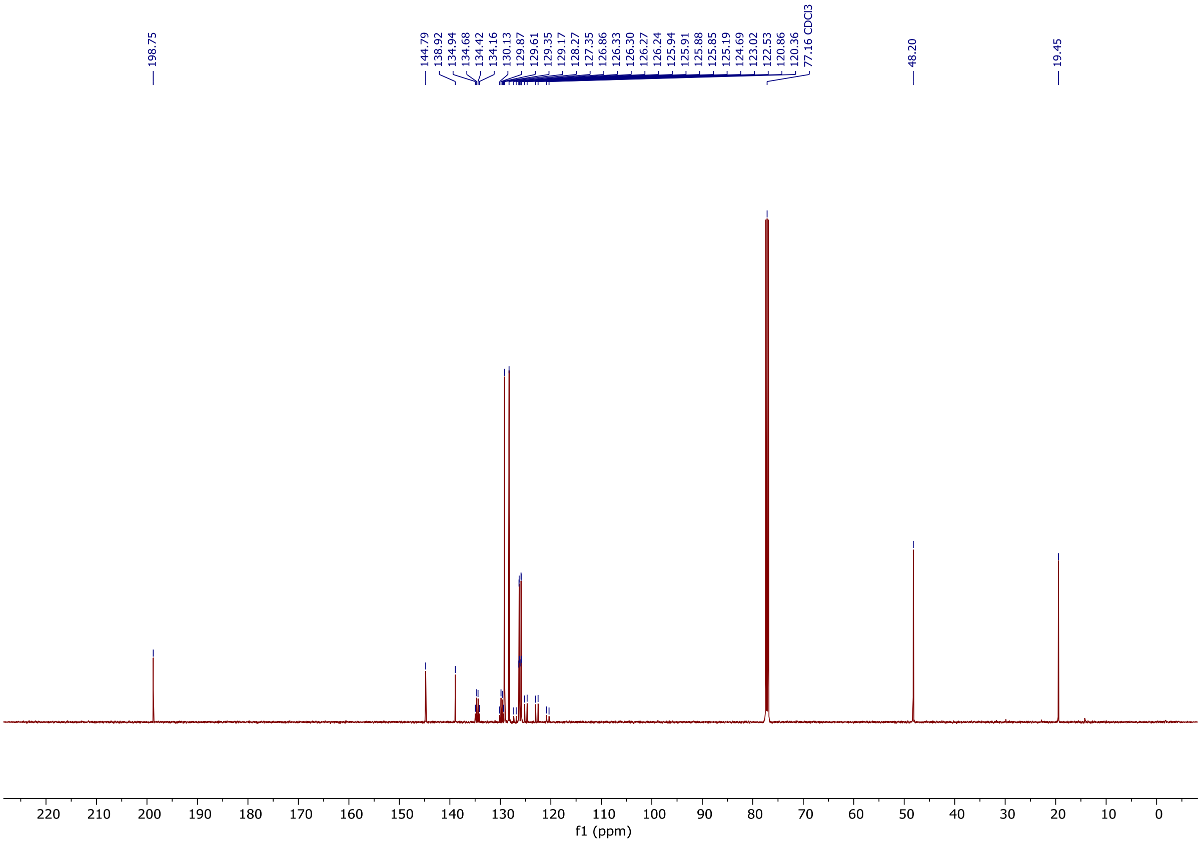

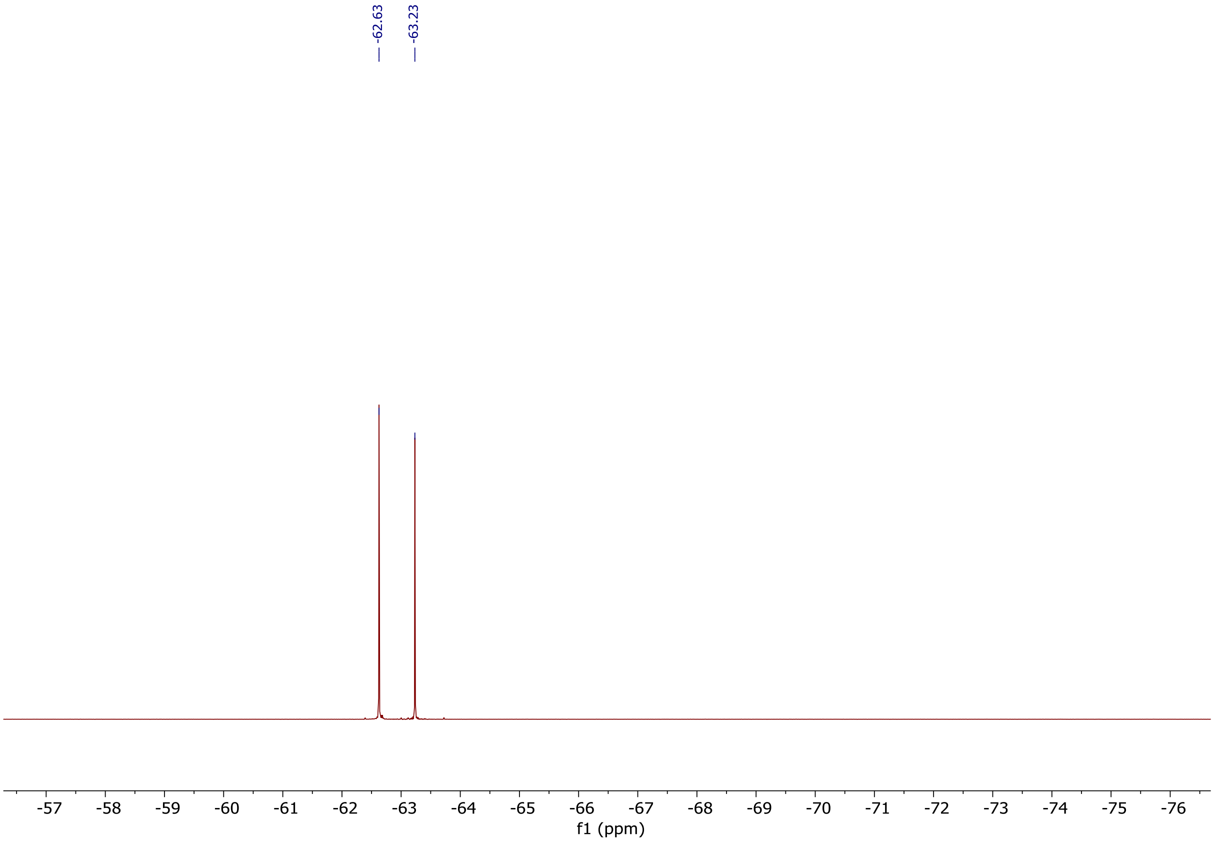
-62.63
-63.23
-57
-58
-59
-60
-61
-62
-63
-64
-65
-66
-67
-68
-69
-70
-71
-72
-73
-74
-75
-76
f1 (ppm)

(*R*)-1,2-Bis(3-trifluoromethylphenyl)propan-1-one (**(*R*)-2n**)

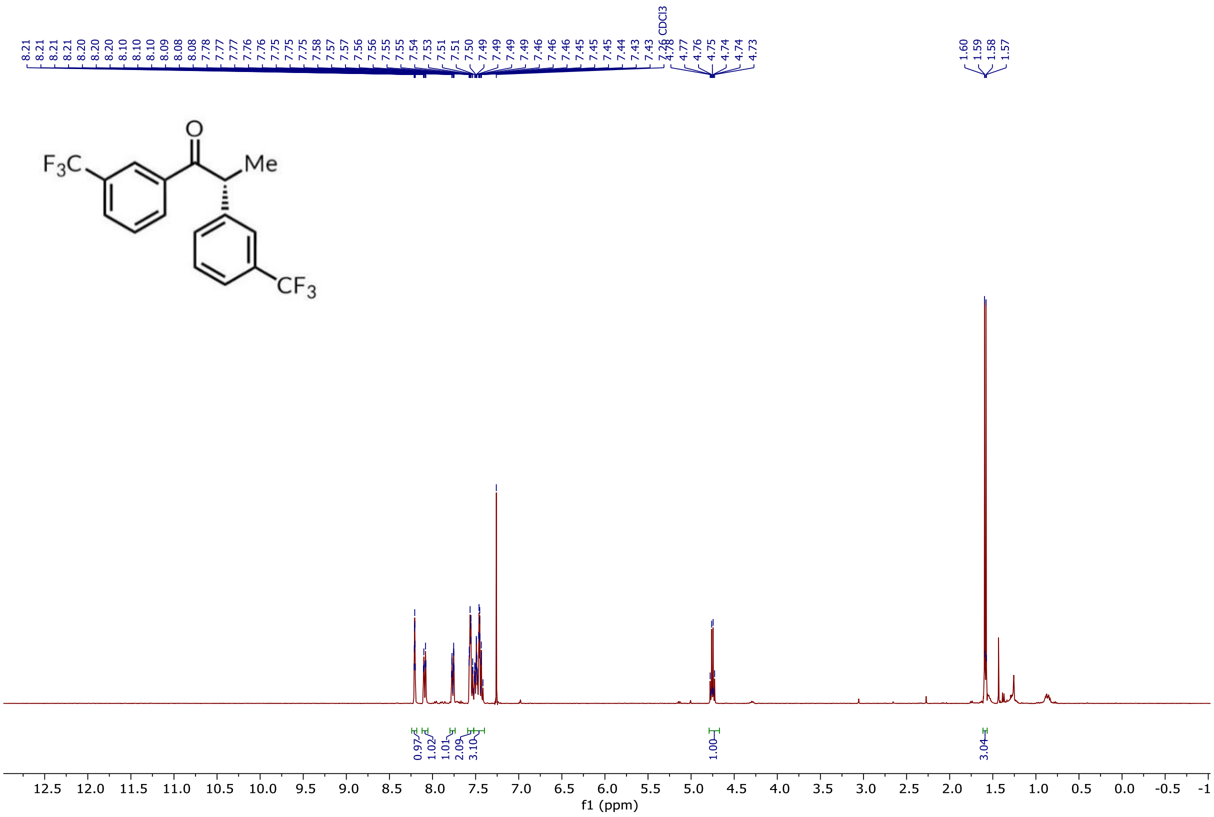

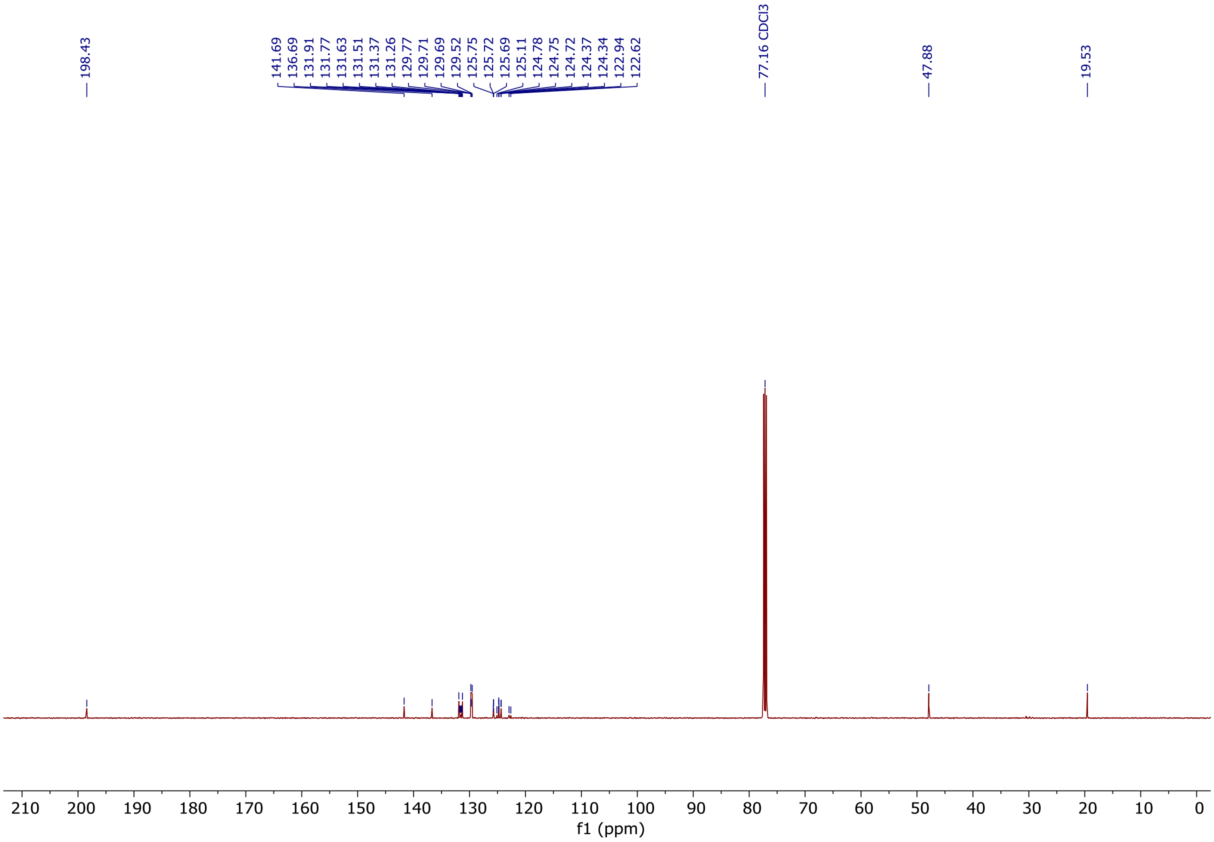

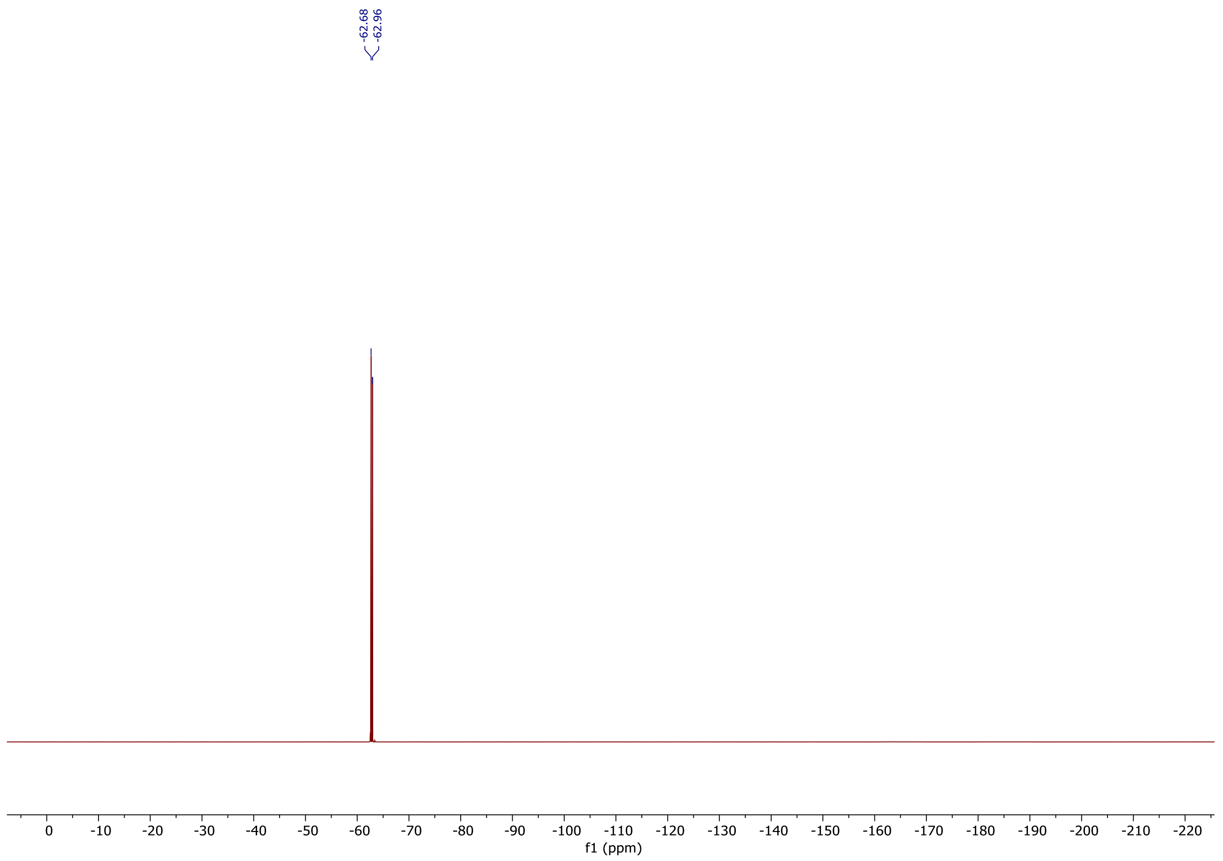
-62.68
-62.96
0 -10 -20 -30 -40 -50 -60 -70 -80 -90 -100 -110 -120 -130 -140 -150 -160 -170 -180 -190 -200 -210 -220
f1 (ppm)

(*R*)-1,2-Bis(3-chlorophenyl)propan-1-one (**(*R*)-2o**)

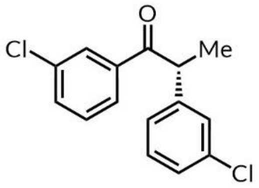

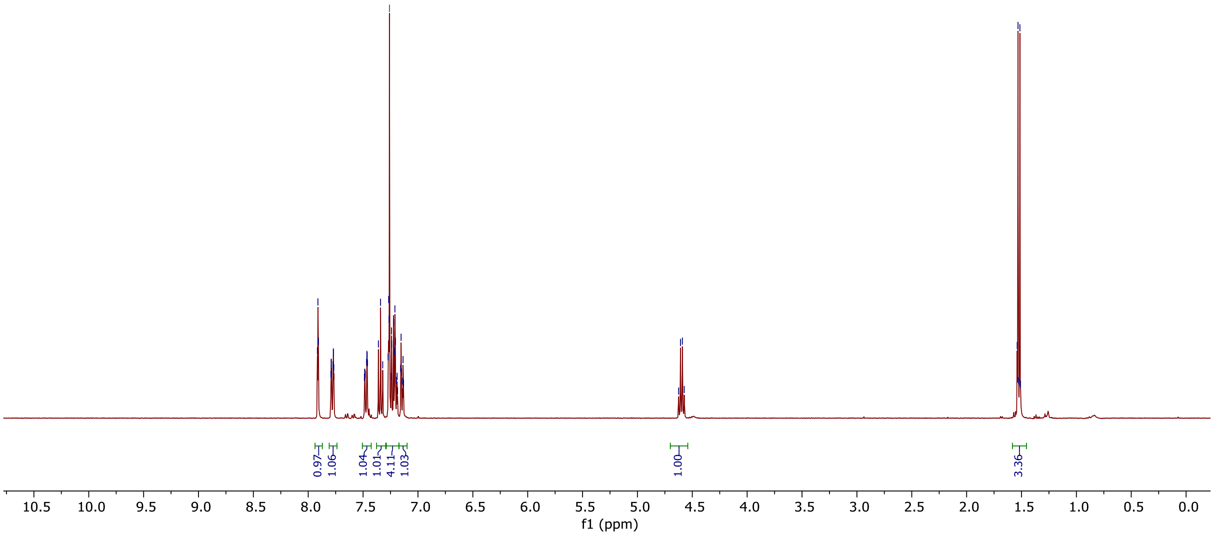

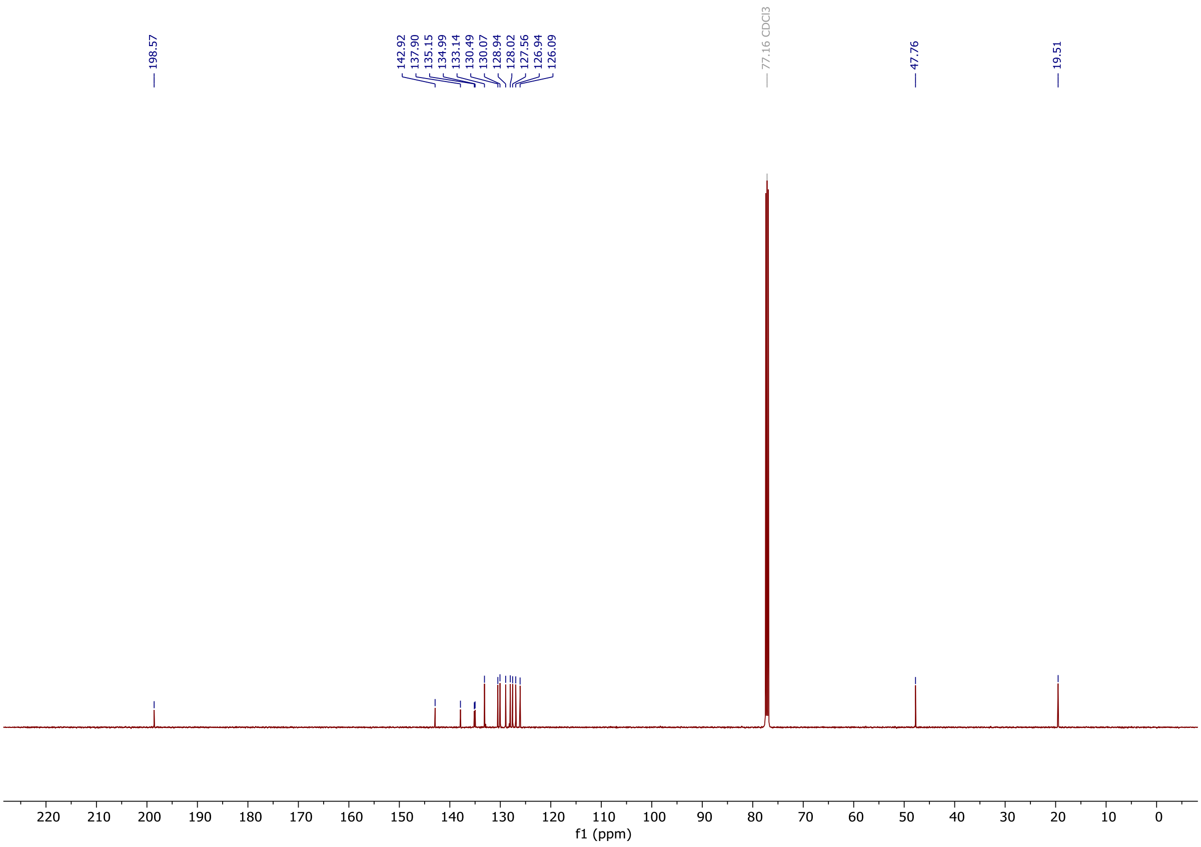

(*R*)-1,2-Bis(3-fluorophenyl)propan-1-one (**(*R*)-2p**)

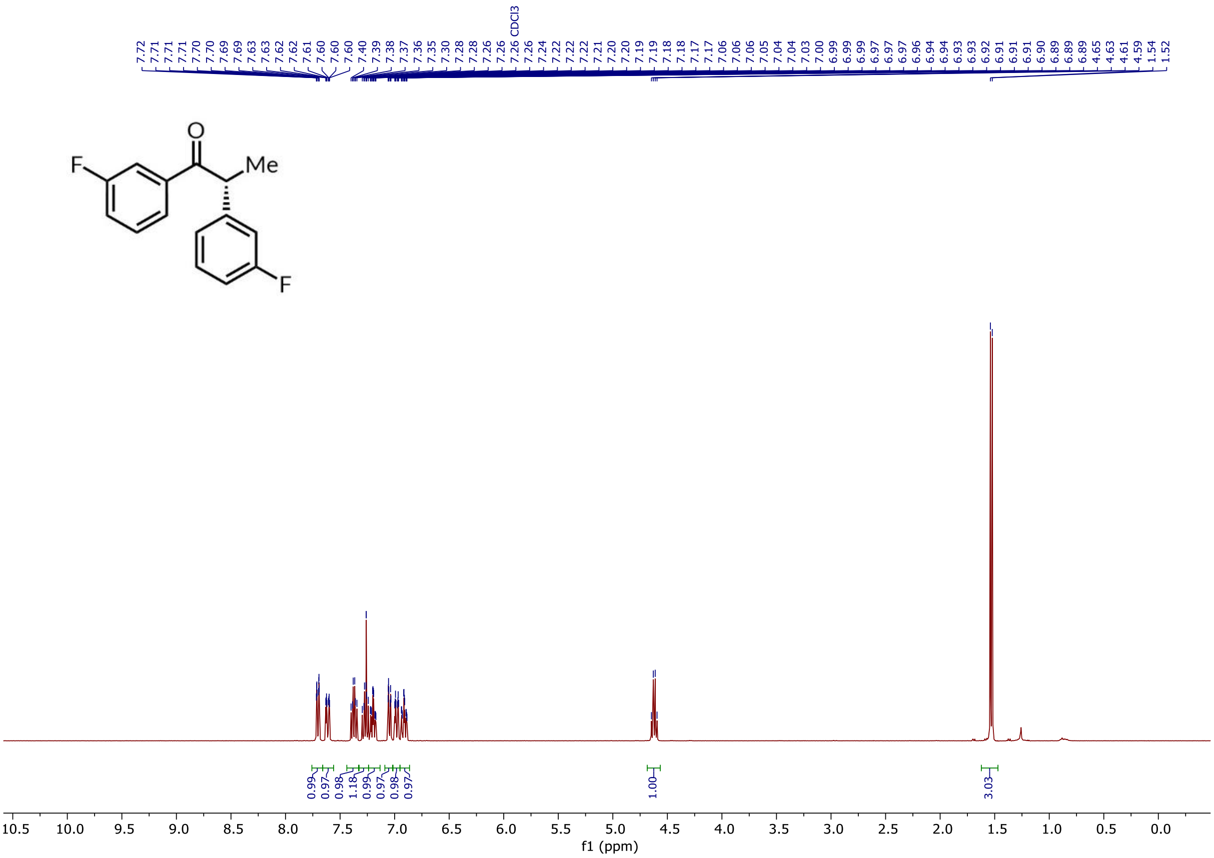

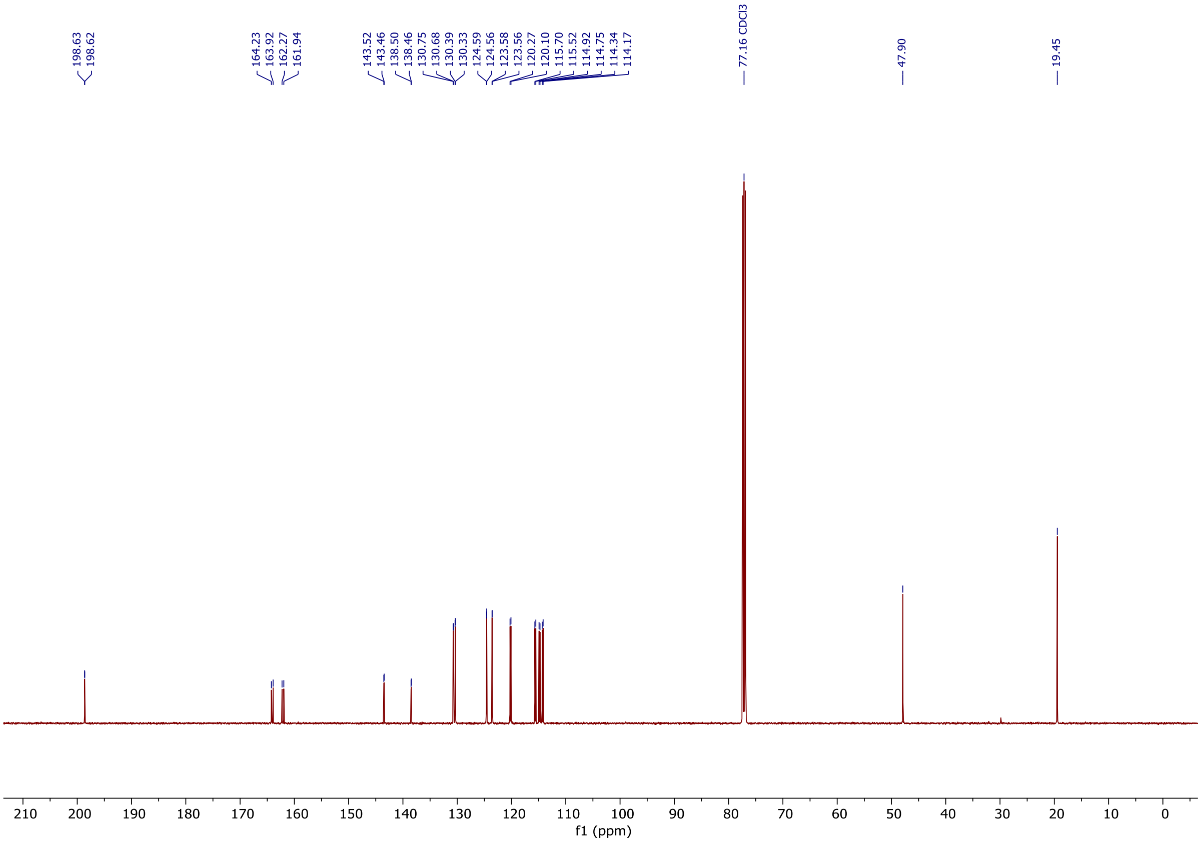

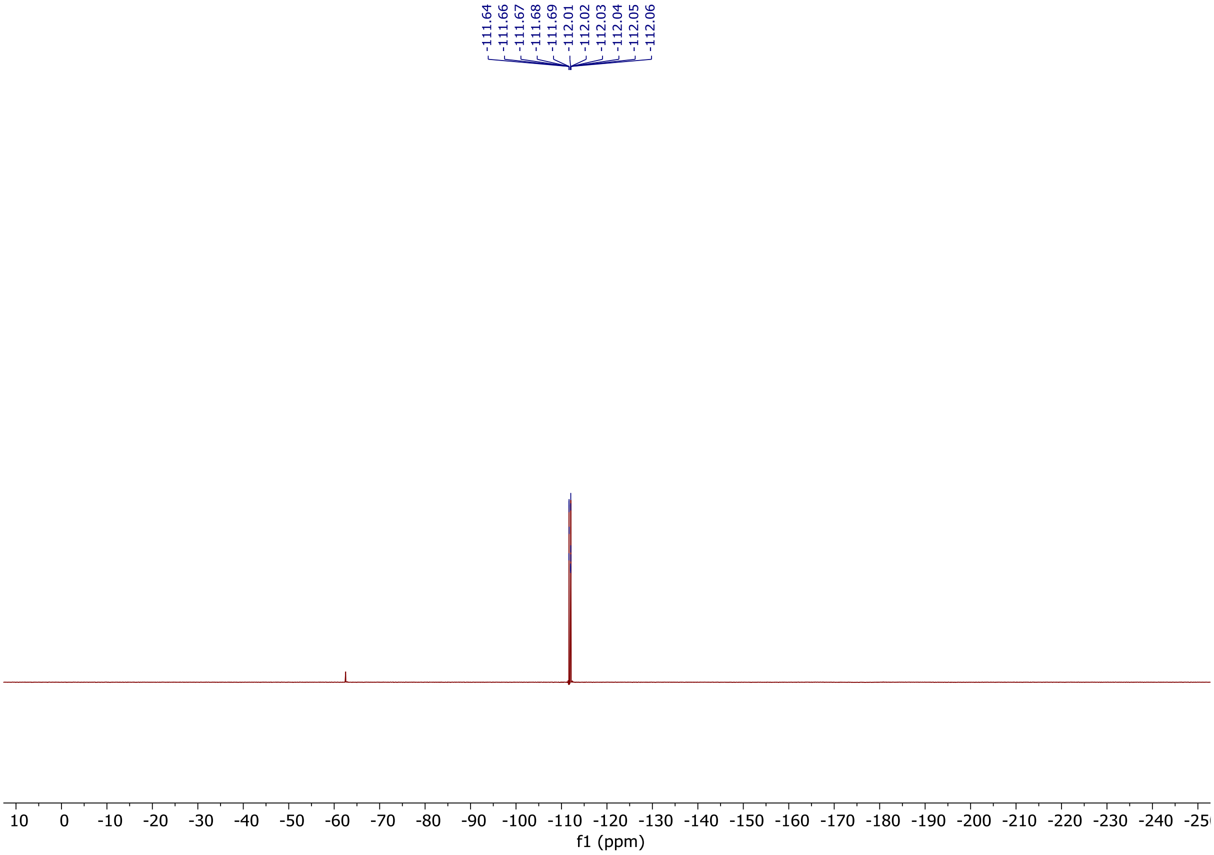
-111.64
-111.66
-111.67
-111.68
-111.69
-112.01
-112.02
-112.03
-112.04
-112.05
-112.06
10 0 -10 -20 -30 -40 -50 -60 -70 -80 -90 -100 -110 -120 -130 -140 -150 -160 -170 -180 -190 -200 -210 -220 -230 -240
f1 (ppm)

(*R*)-1,2-*m*-tolylpropan-1-one (**(*R*)-2q**)

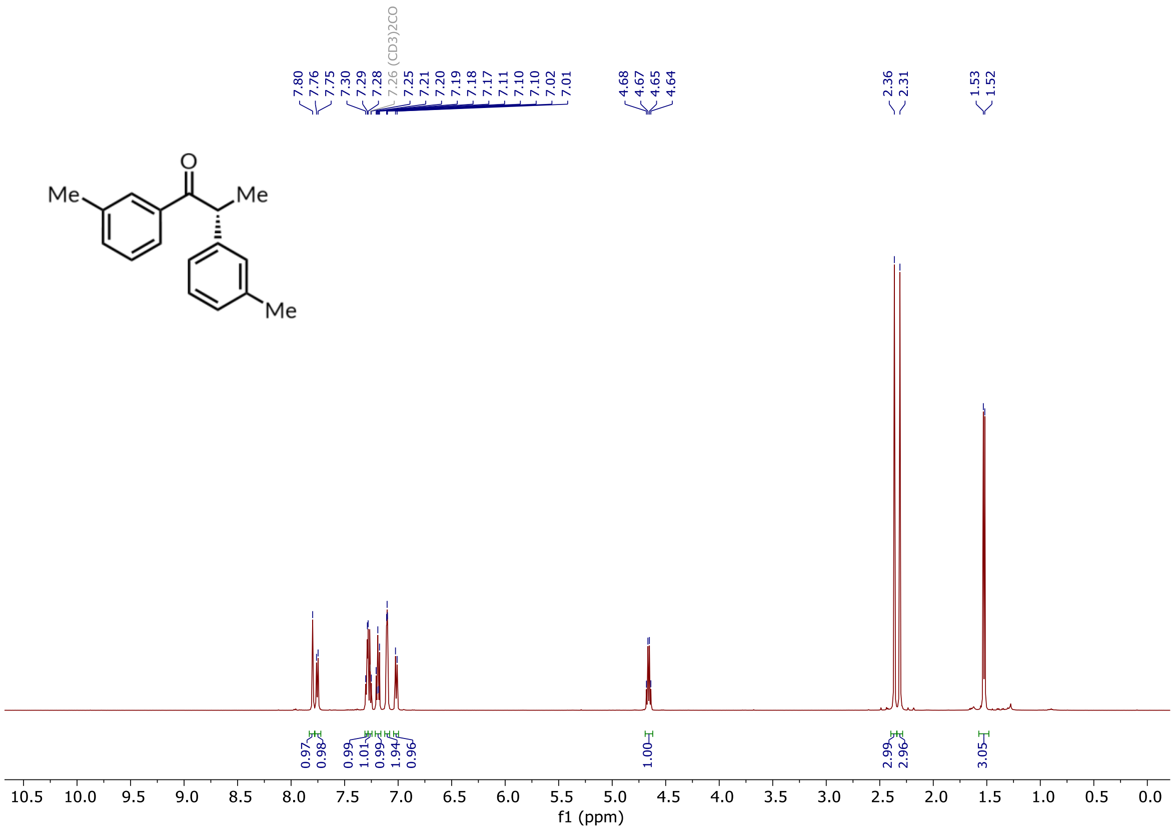

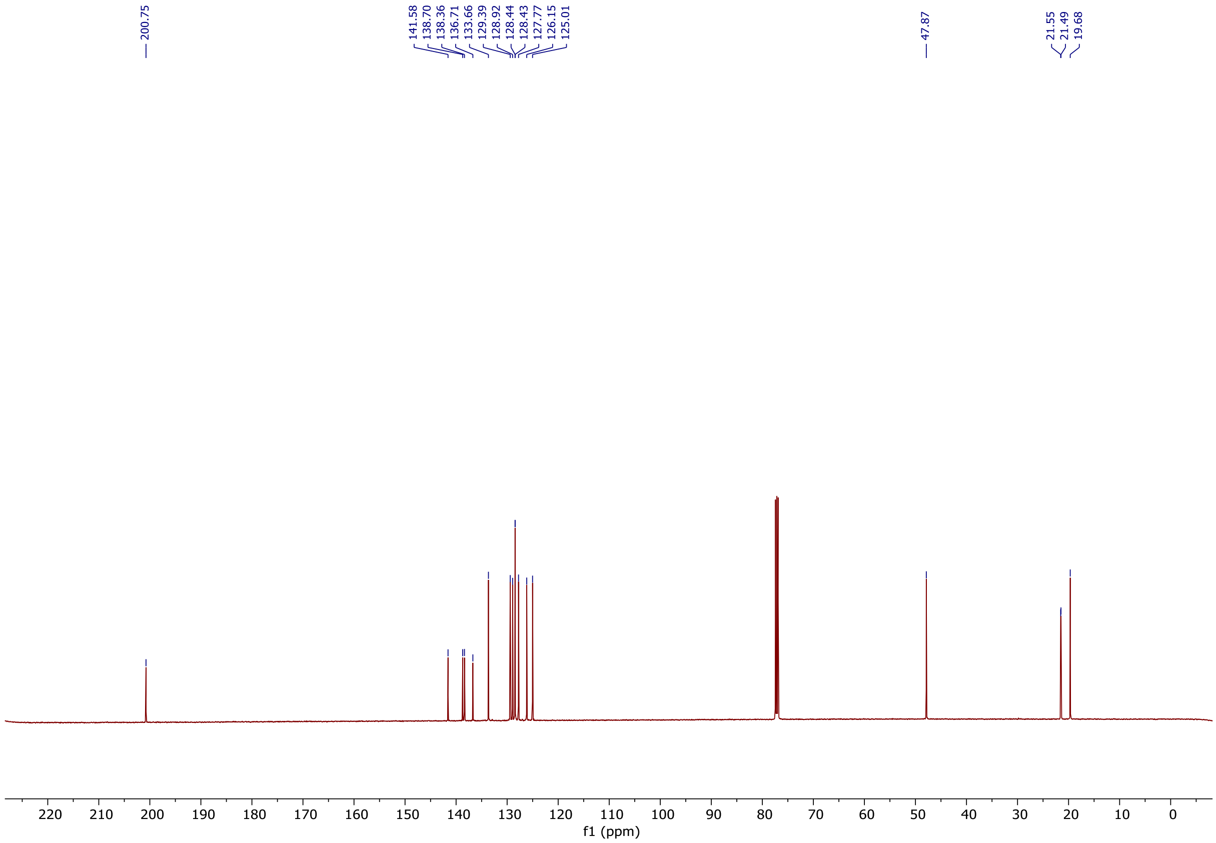

(*R*)-1,2-bis(3-methoxyphenyl)propan-1-one (**(*R*)-2r**)

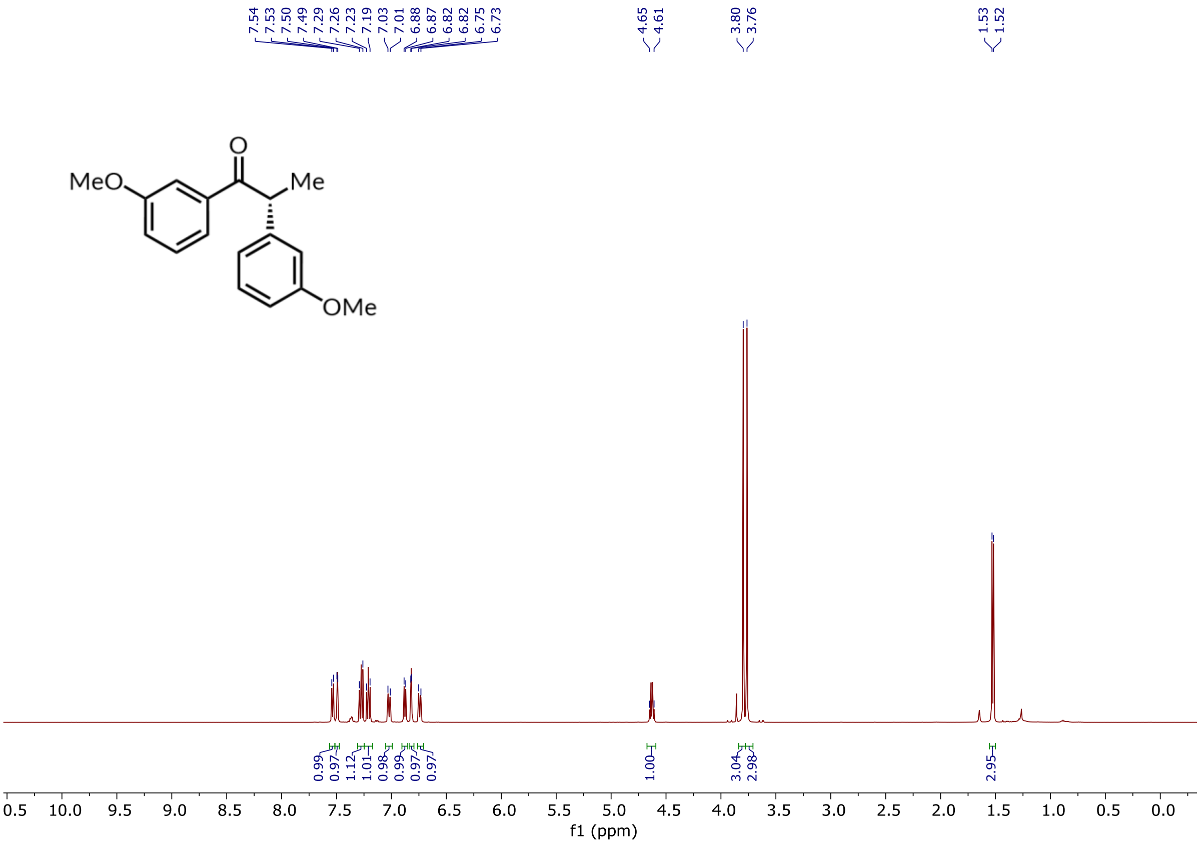

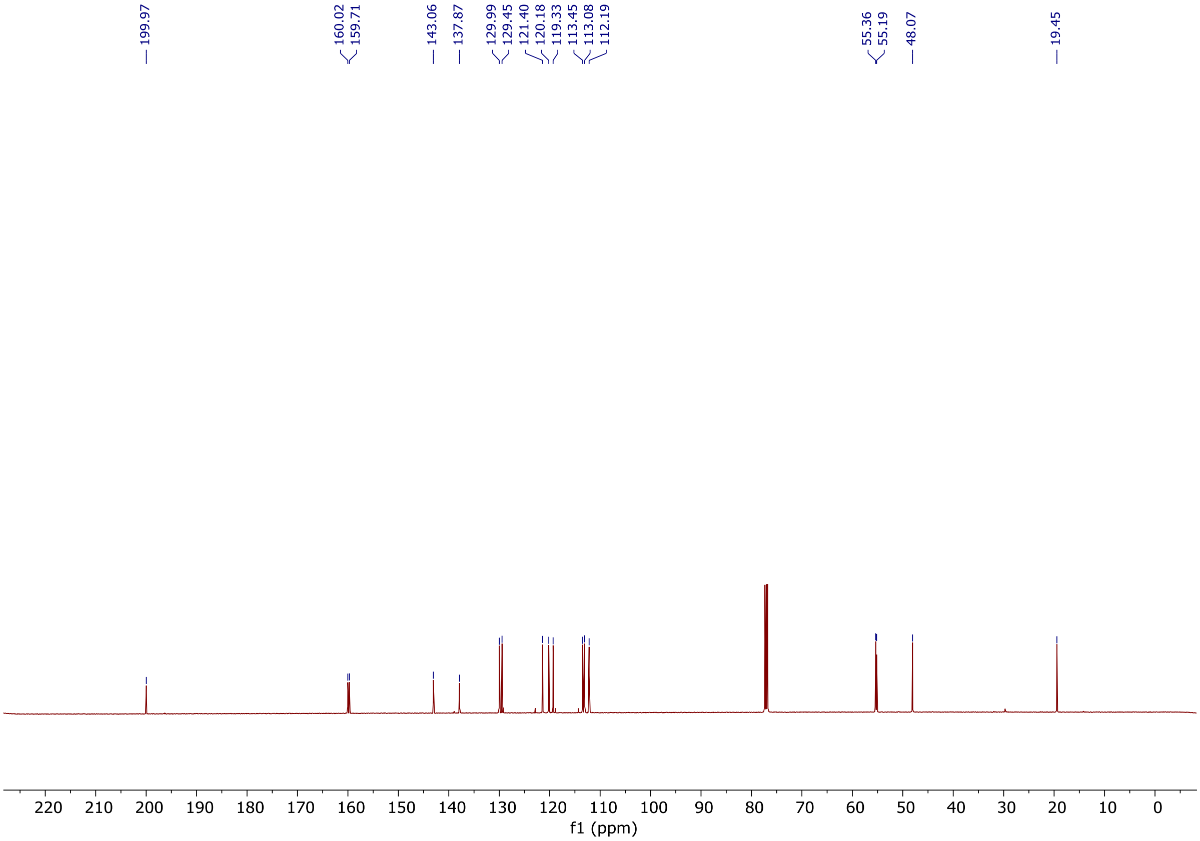

(±)-1,2-Bis(2-chlorophenyl)propan-1-one ((***R*)-2s**)

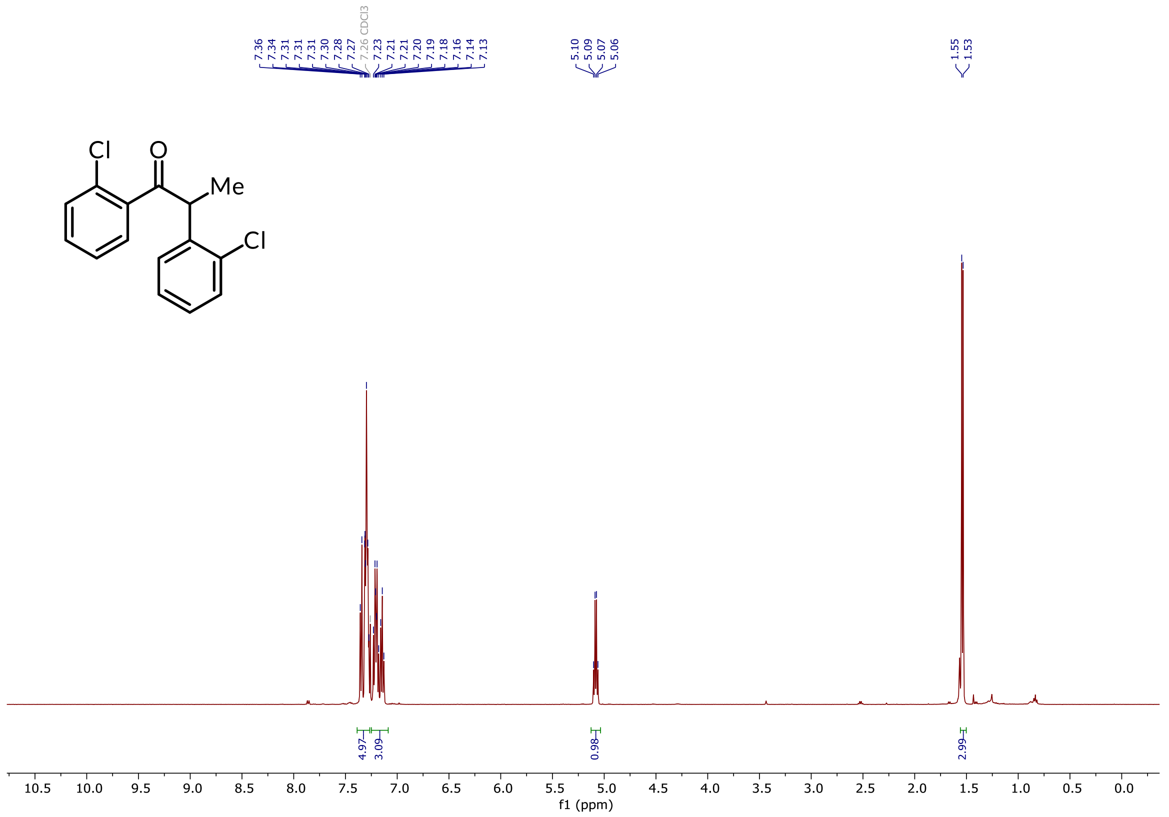

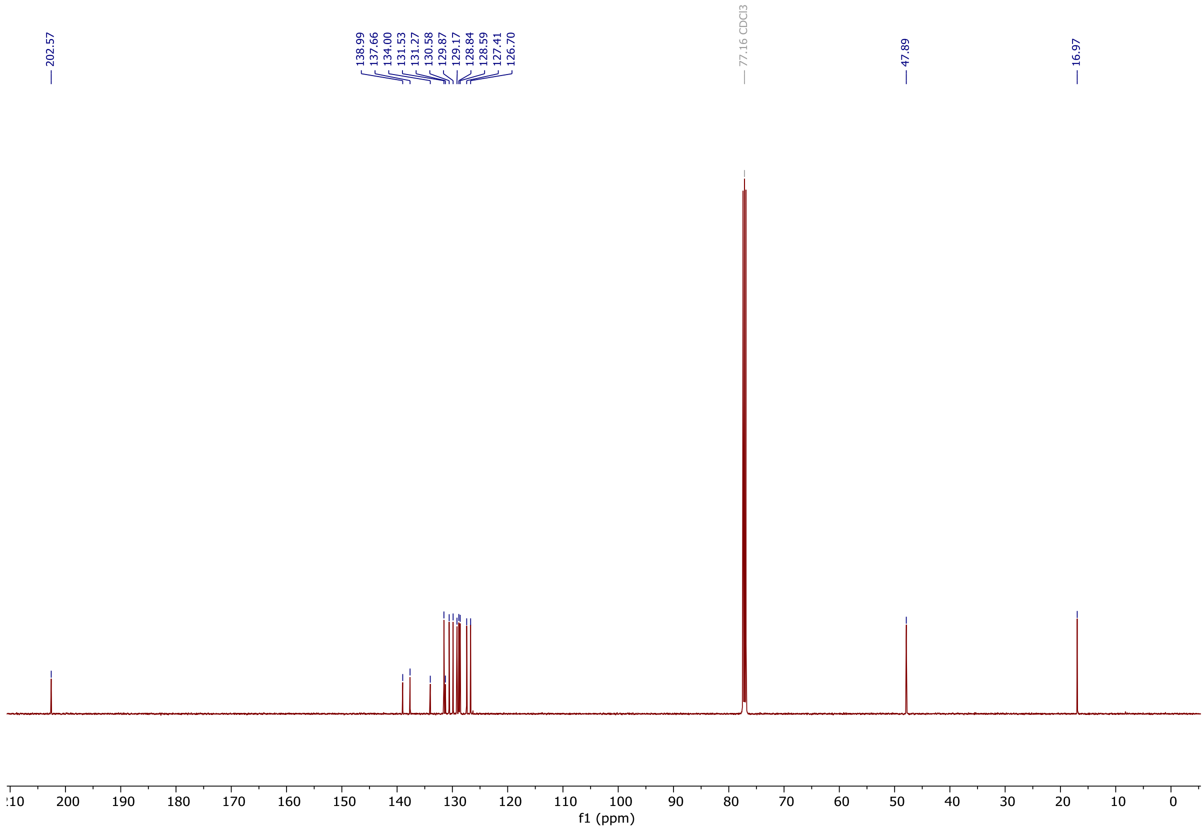

(±)-1,2-Di-*o*-tolylpropan-1-one (**(±)-2t**)

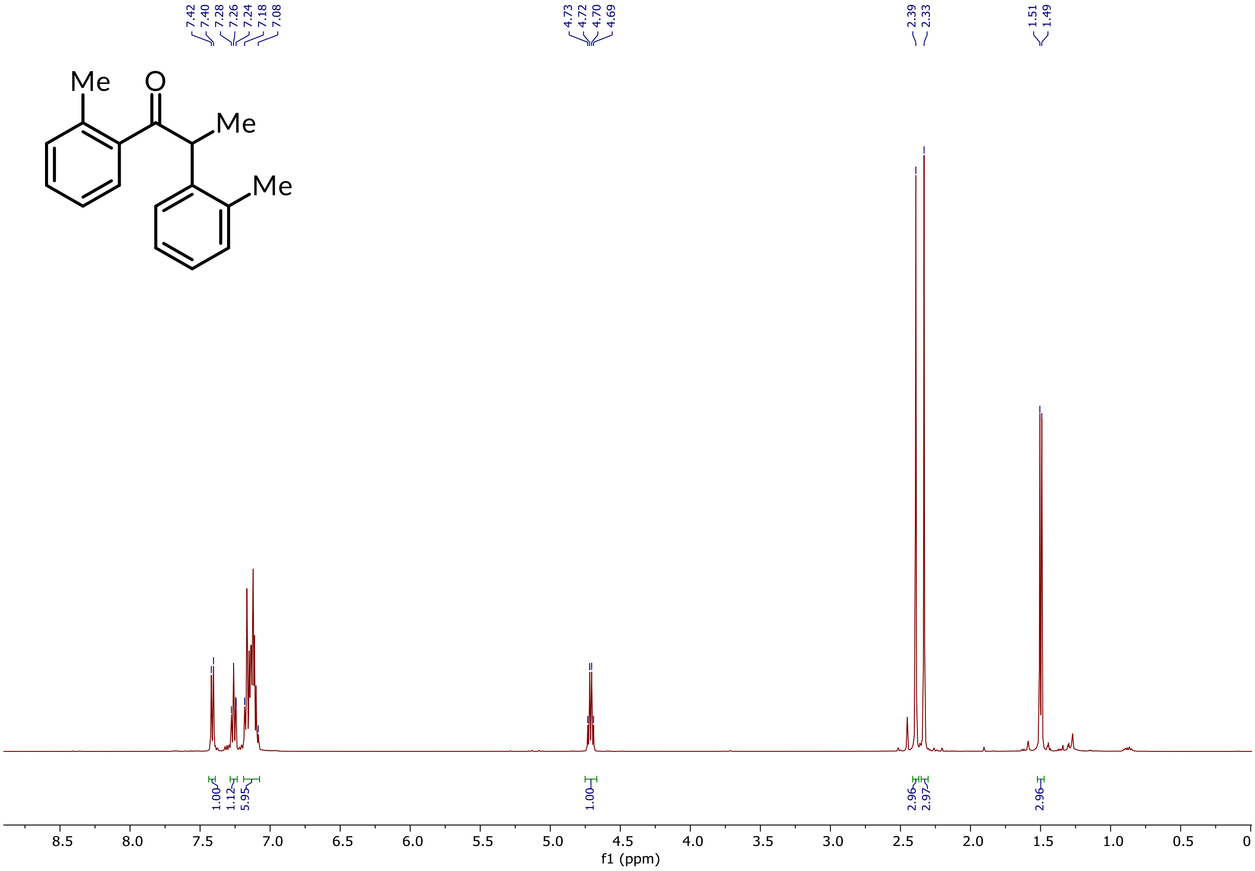

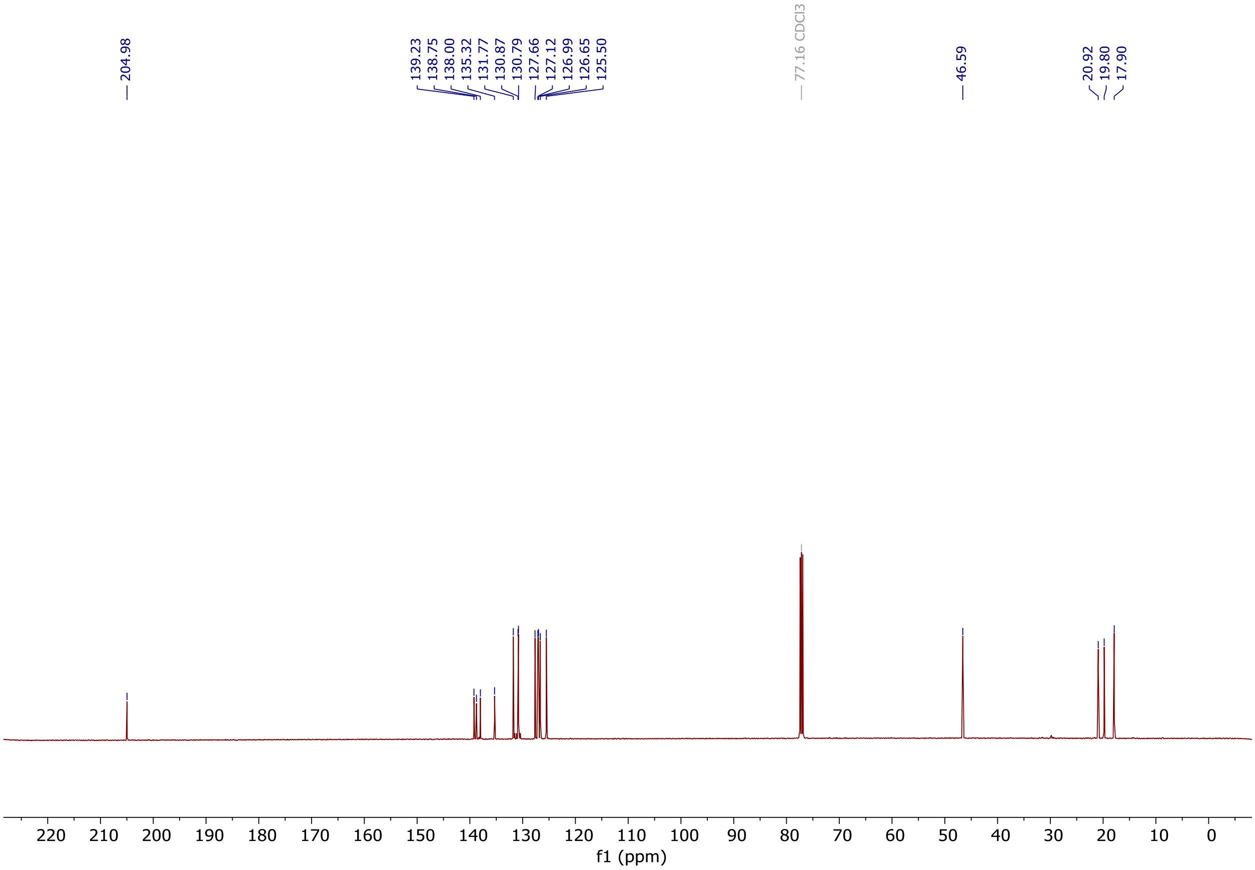

(*R*)-1,2-Bis(2-methoxyphenyl)propan-1-one (**(*R*)-2u**)

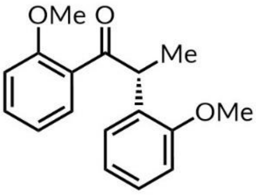

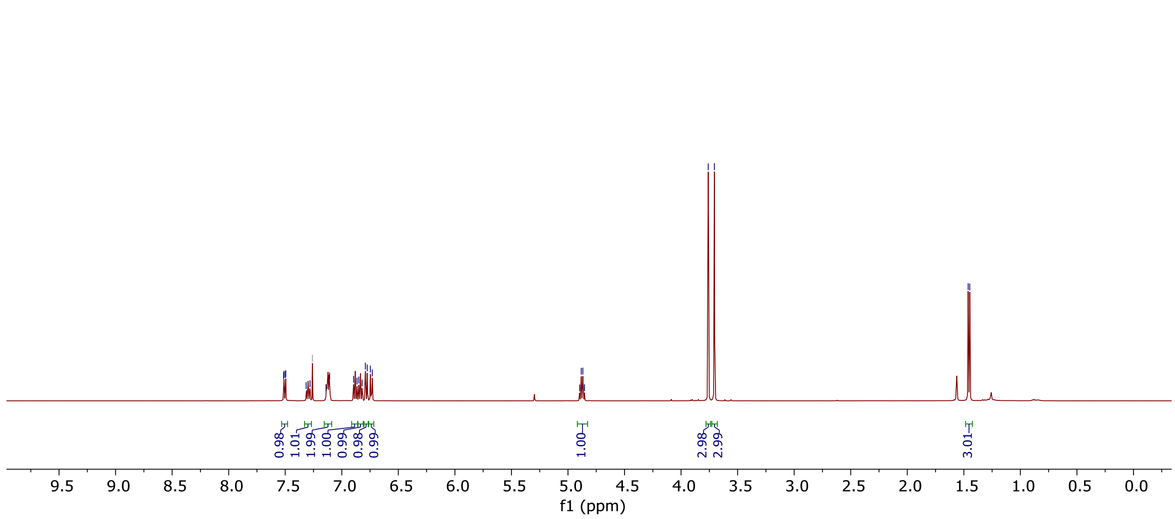

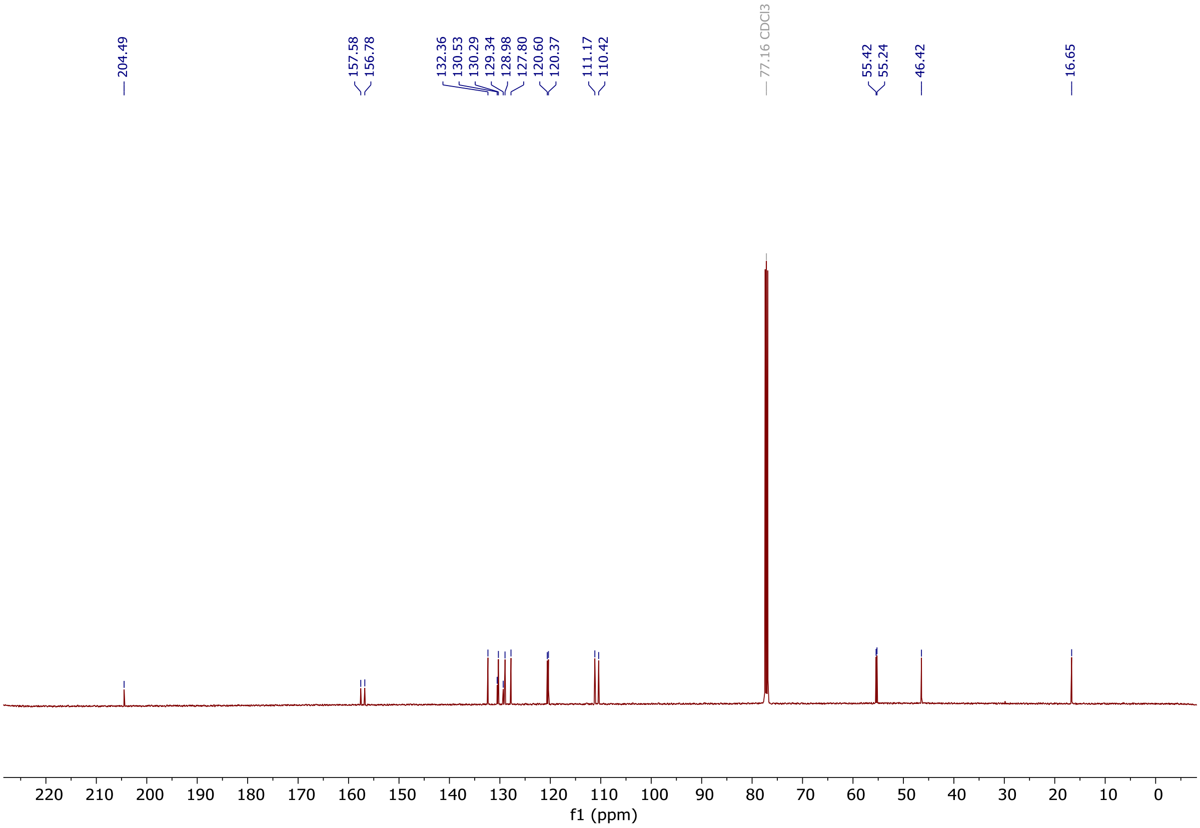

(*R*)-1,2-Bis(2-fluorophenyl)propan-1-one (**(*R*)-2v**)

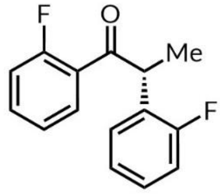

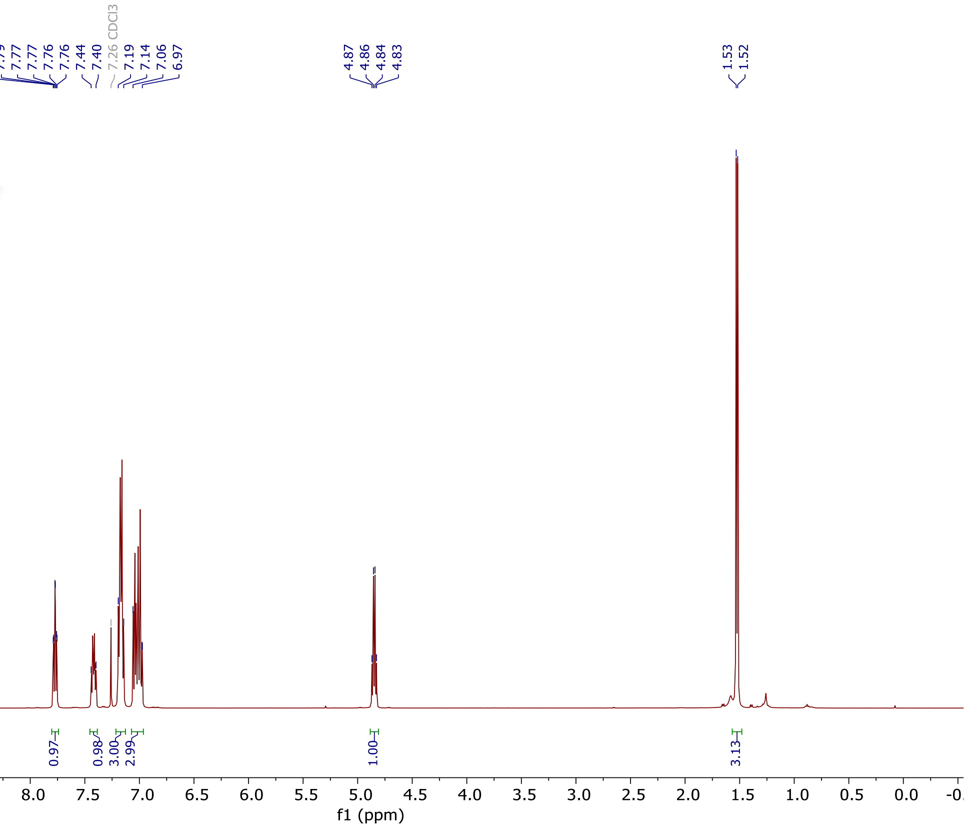

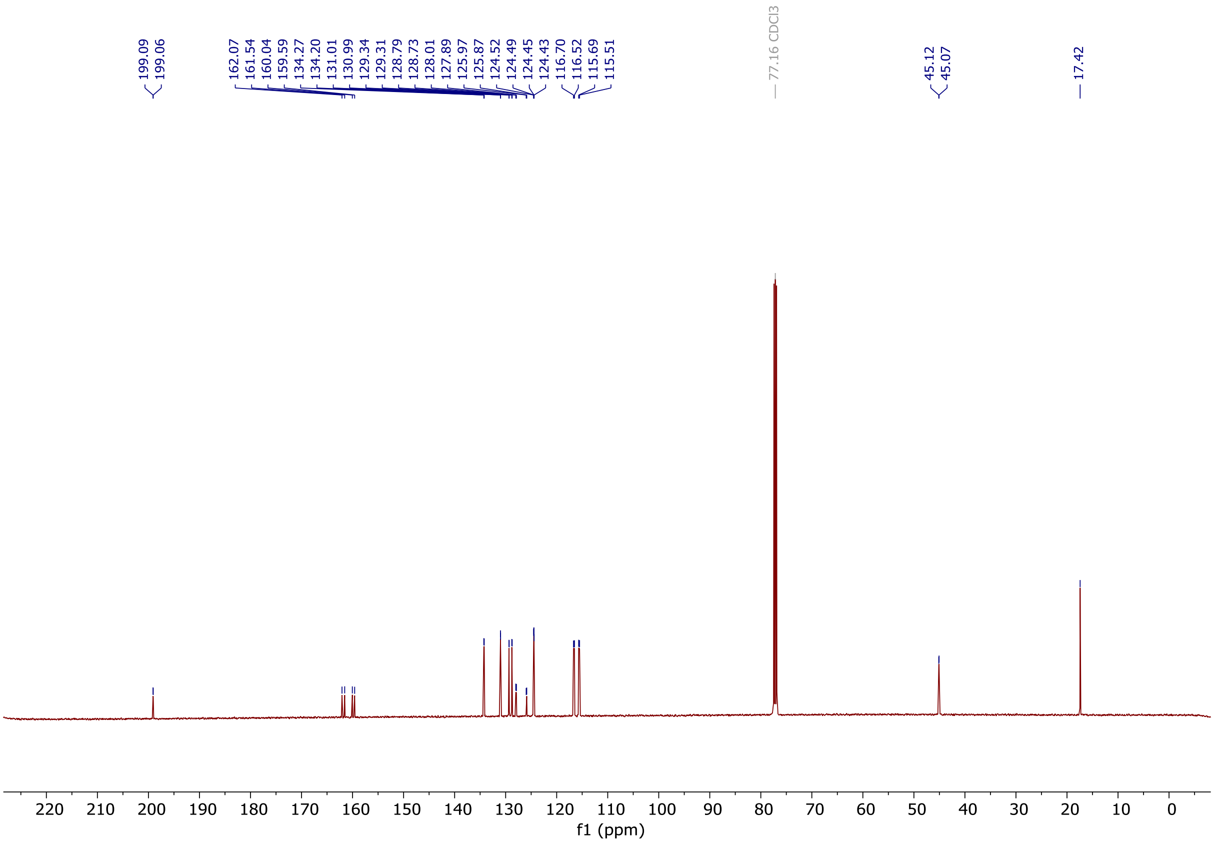

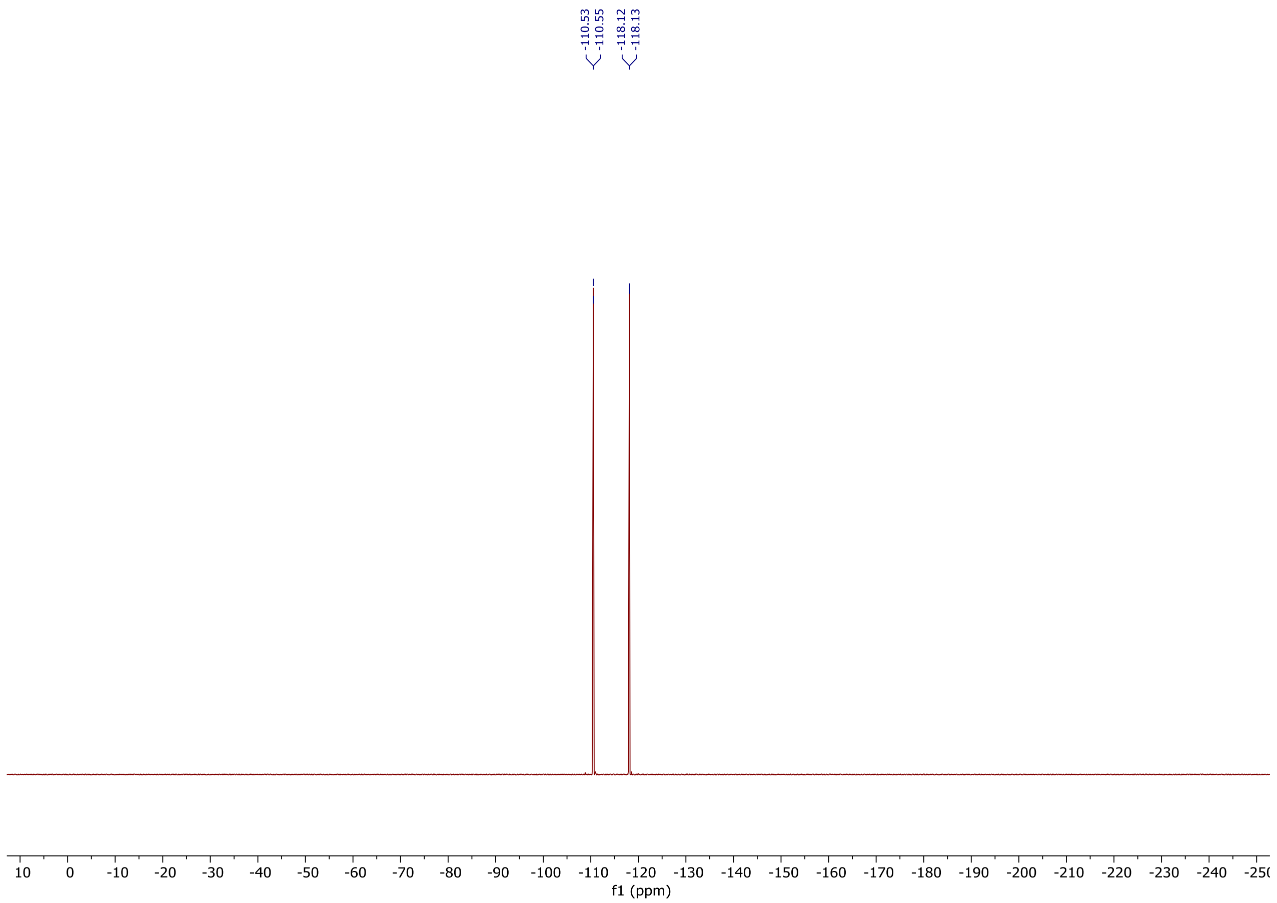

-110.53
-110.55
-118.12
-118.13
10 0 -10 -20 -30 -40 -50 -60 -70 -80 -90 -100 -110 -120 -130 -140 -150 -160 -170 -180 -190 -200 -210 -220 -230 -240
f1 (ppm)

(*R*)-1,2-Di(benzofuran-2-yl)propan-1-one (**(*R*)-2w**)

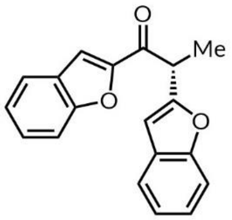

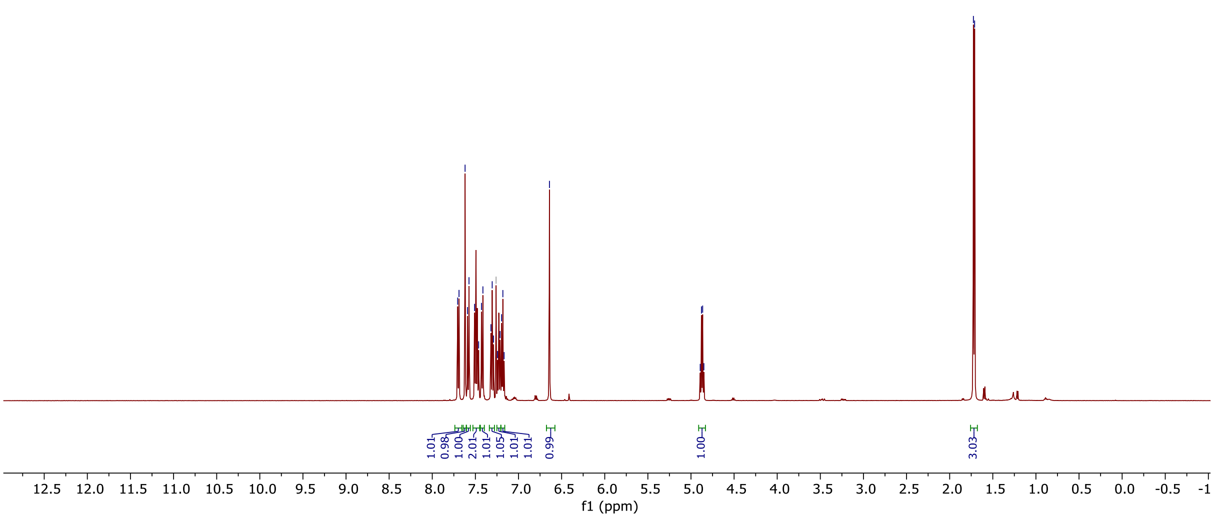

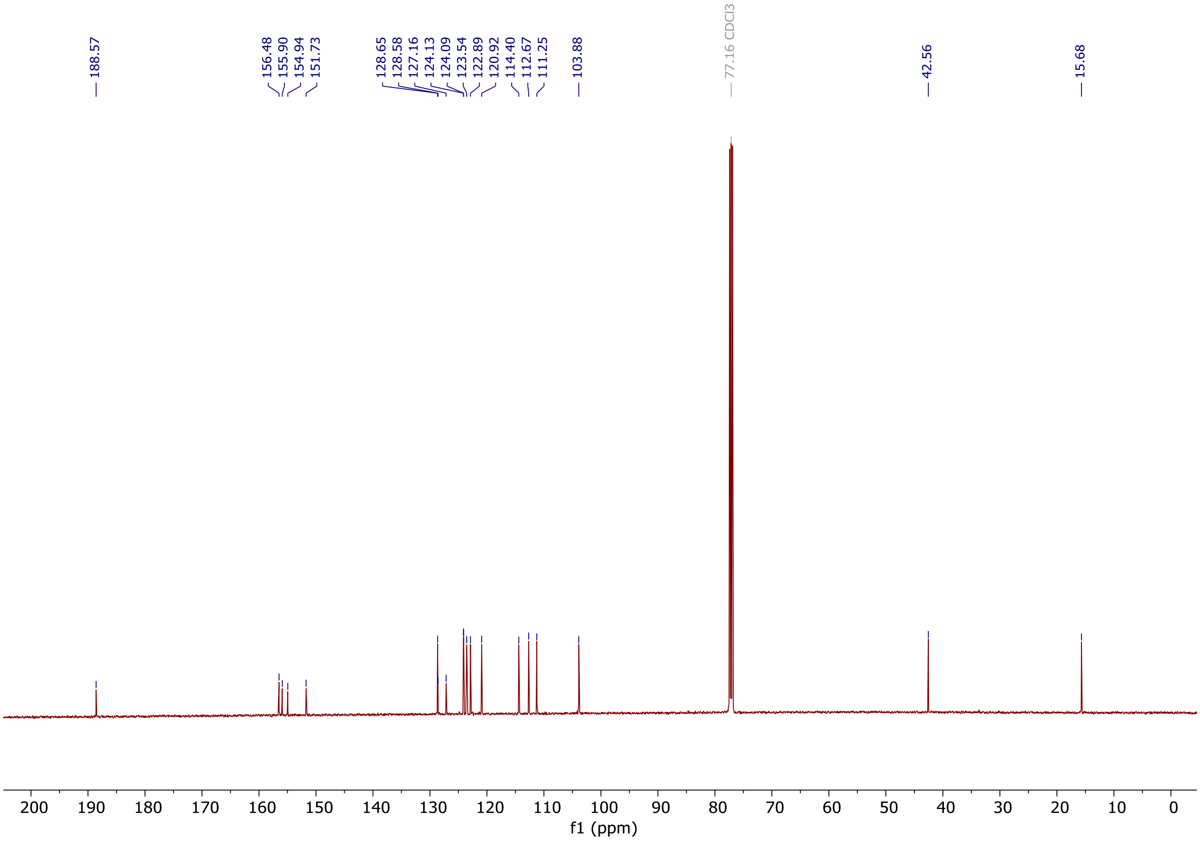

(*S*)-1,2-Bis(benzothiophen-2-yl)propan-1-one (**(*S*)-2x**)

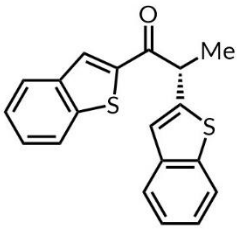

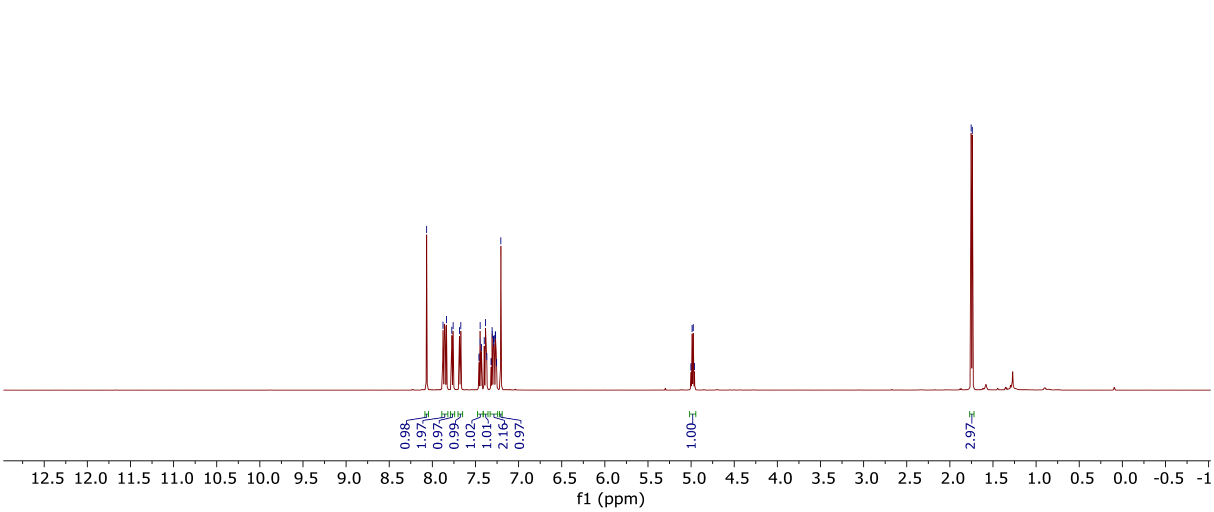

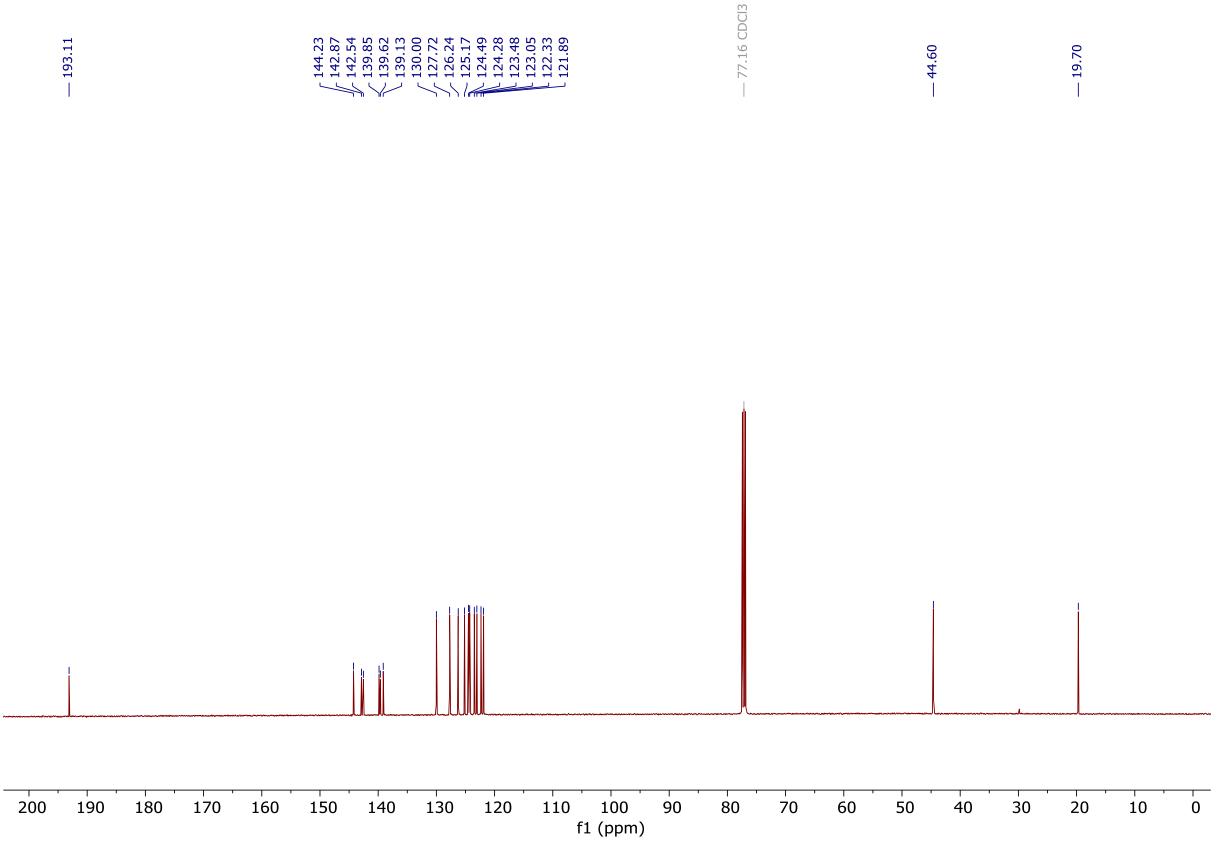

(*S*)-1,2-Di(thiophen-2-yl)propan-1-one (**(*R*)-2y**)

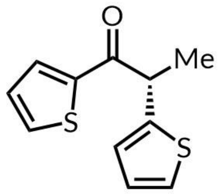

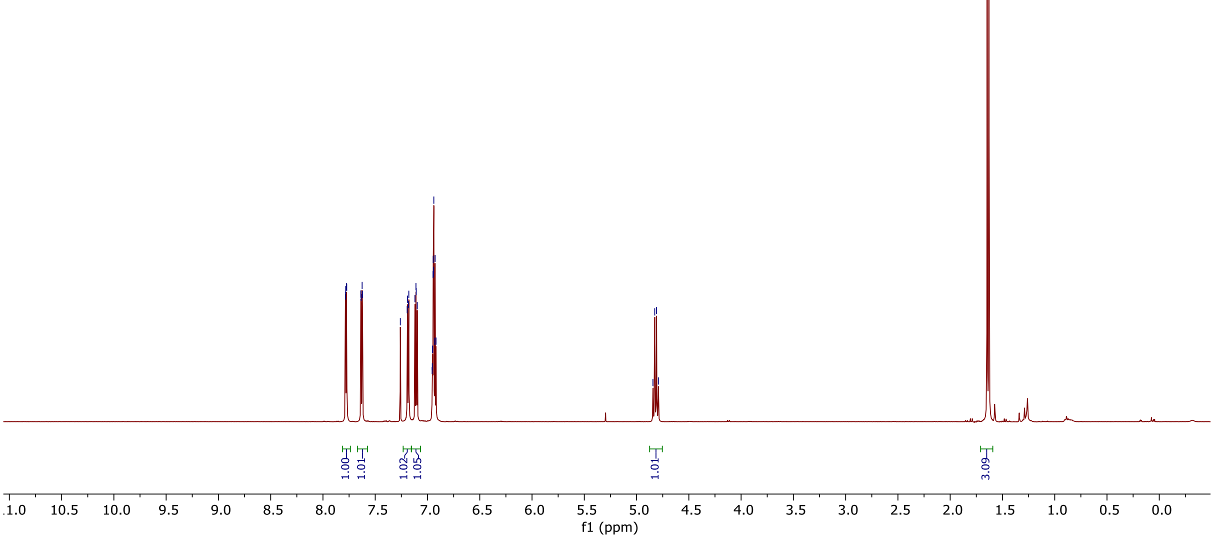

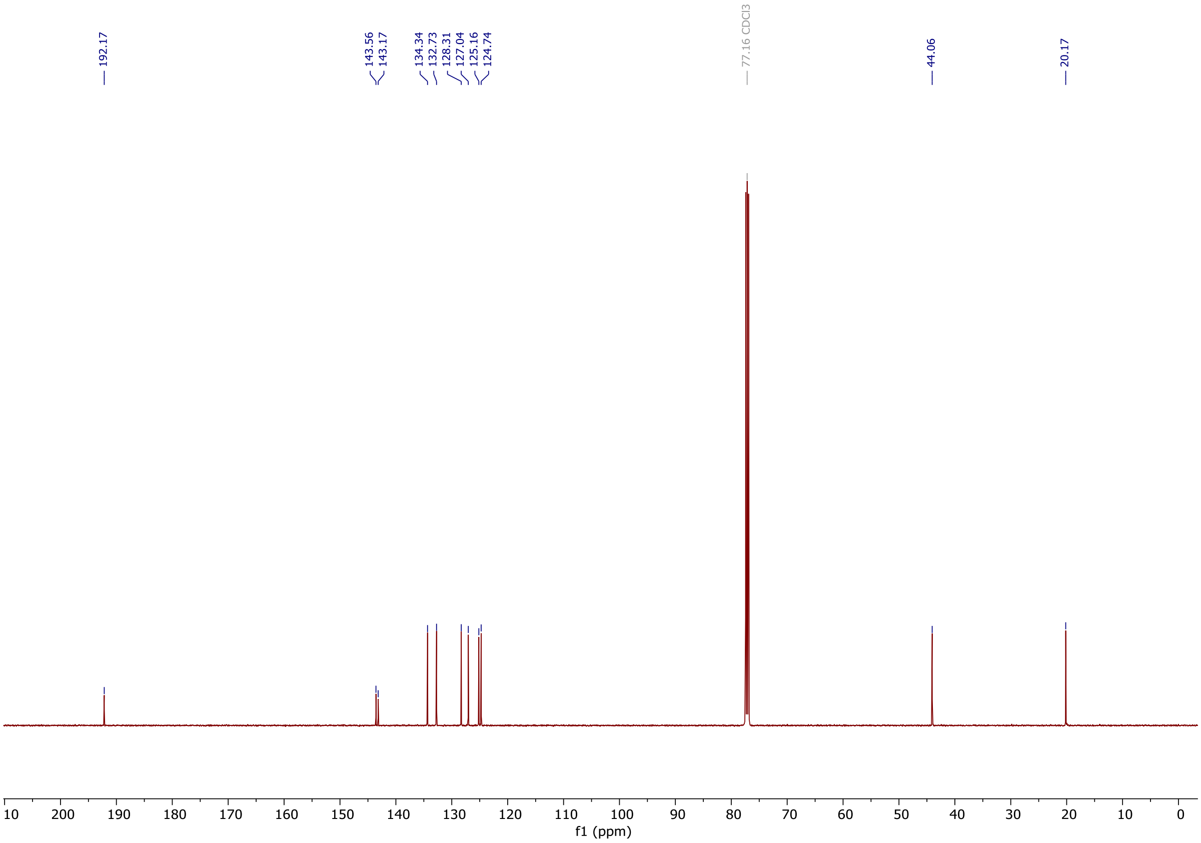

(*R*)-1,2-Bis(6-methoxynaphthalen-2-yl)propan-1-one (**(*R*)-2z**)

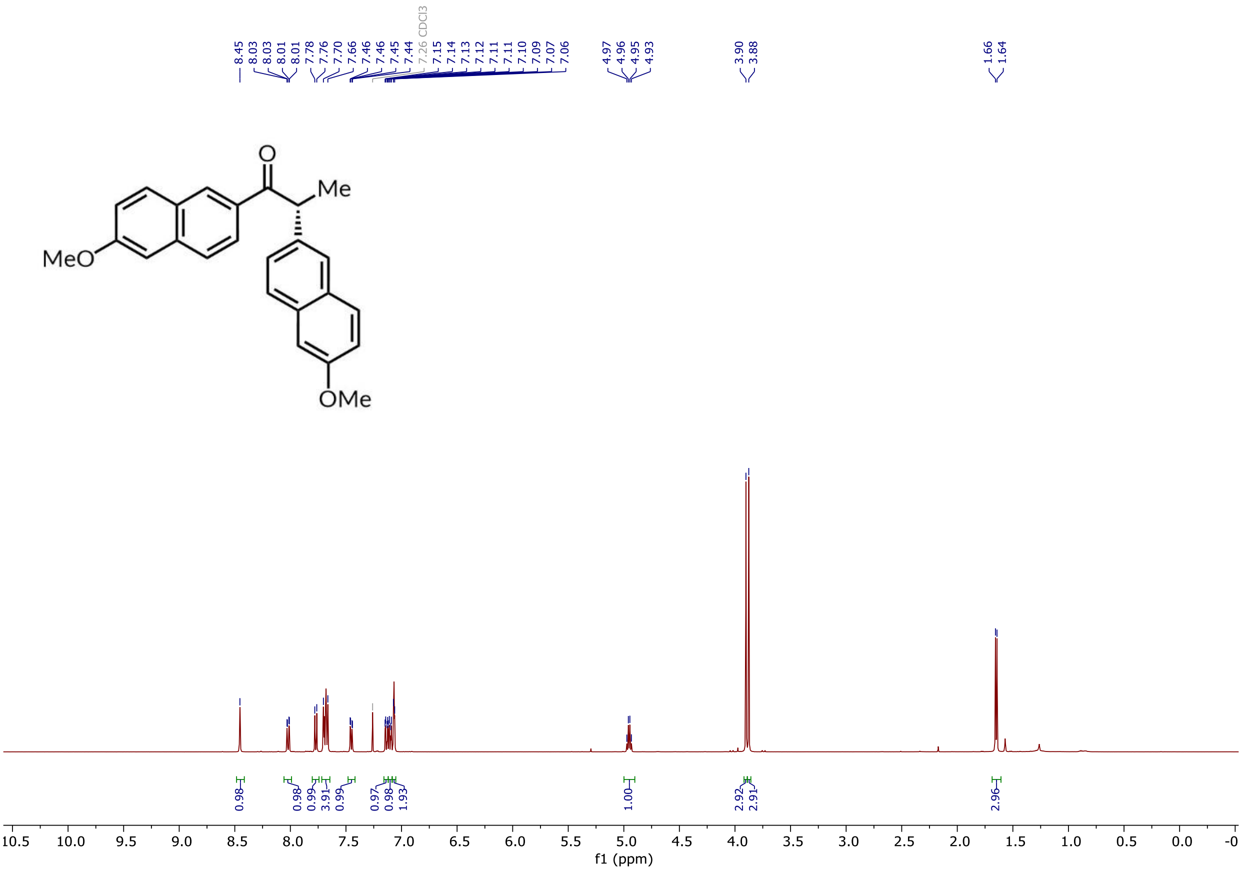

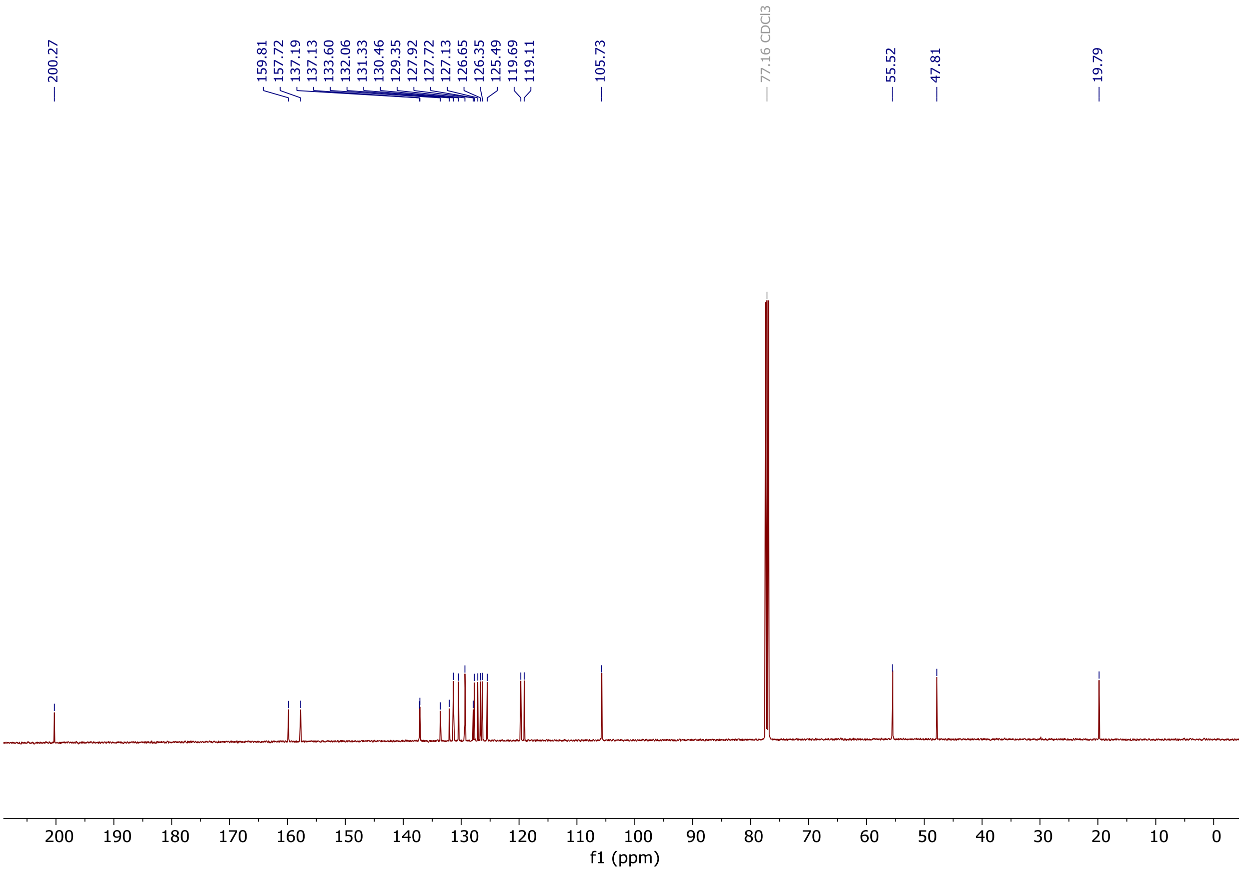

(±)-2-Methyl-1,2-diphenylbutan-1-one (**2aa**)

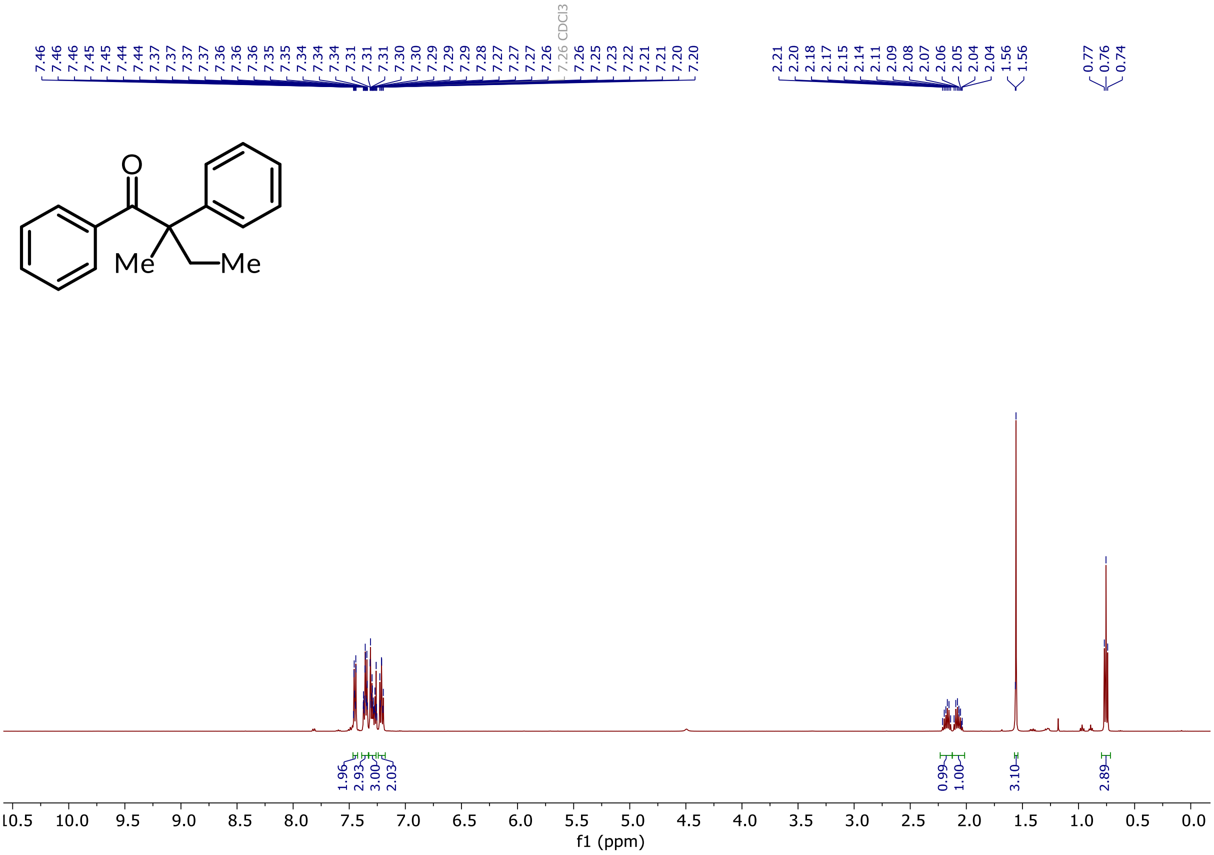

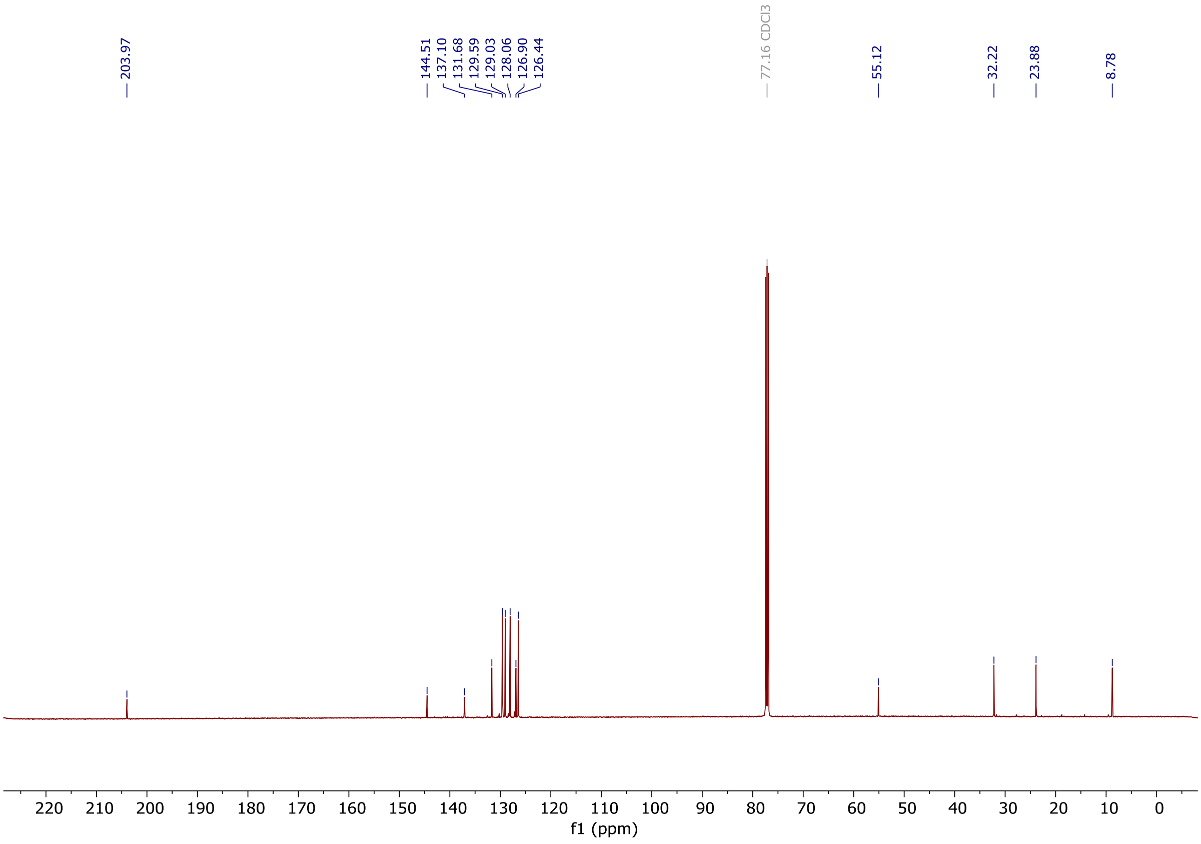

(±)-1-Phenyl-2-(p-tolyl)propan-1-one (**(±)-4a**) and (±)-2-phenyl-1-(p-tolyl)propan-1-one (**(±)-5a**)

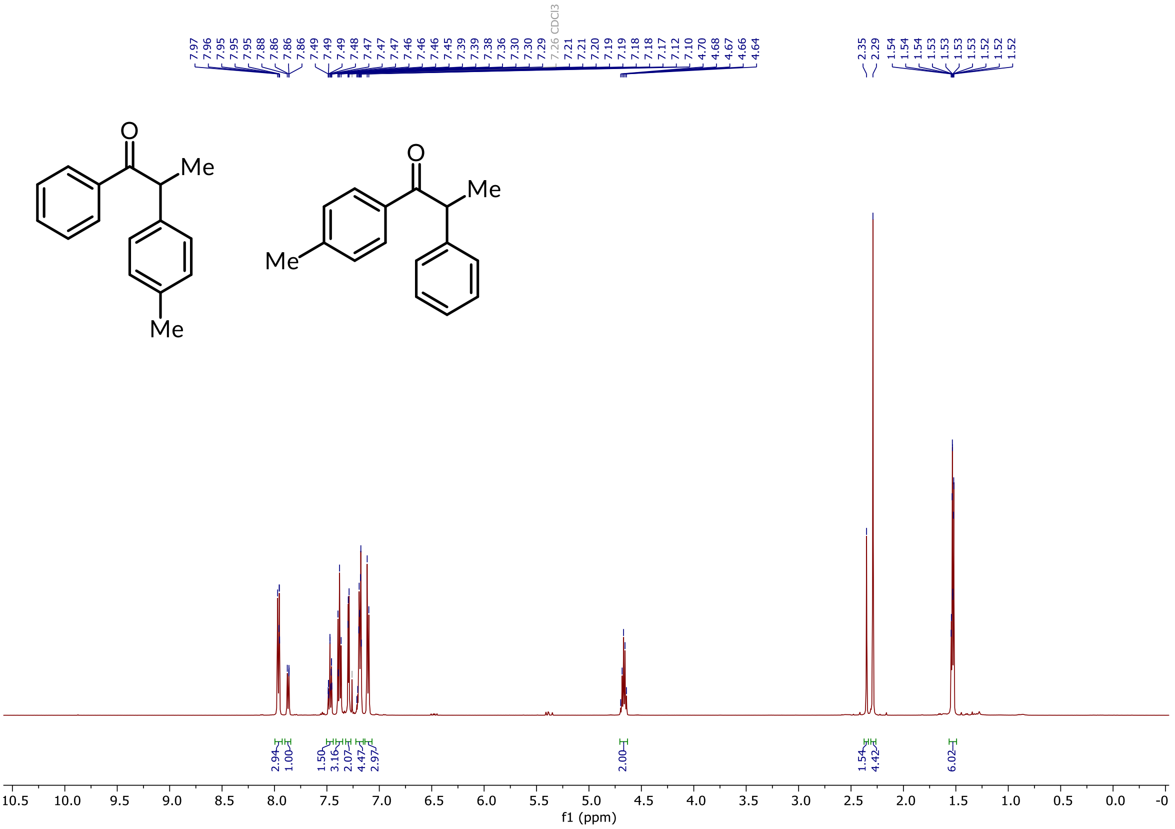

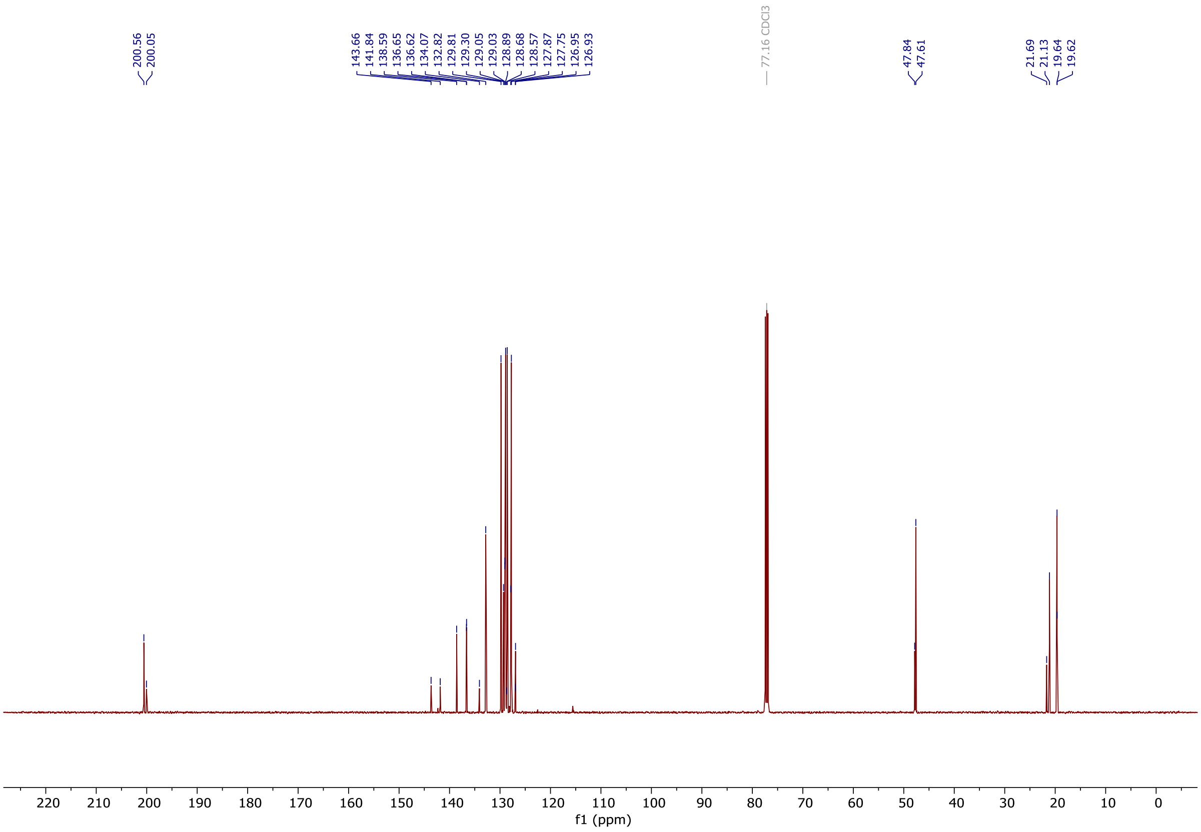

(±)-1-Phenyl-2-(4-fluorophenyl)propan-1-one (**(±)-4b**) and (±)-1-(4-fluorophenyl)-2-phenylpropan-1-one (**(±)-5b**)

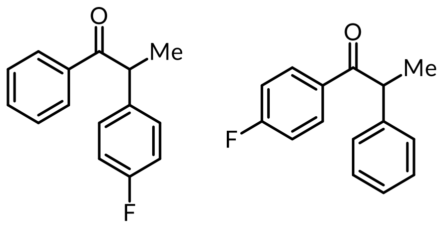

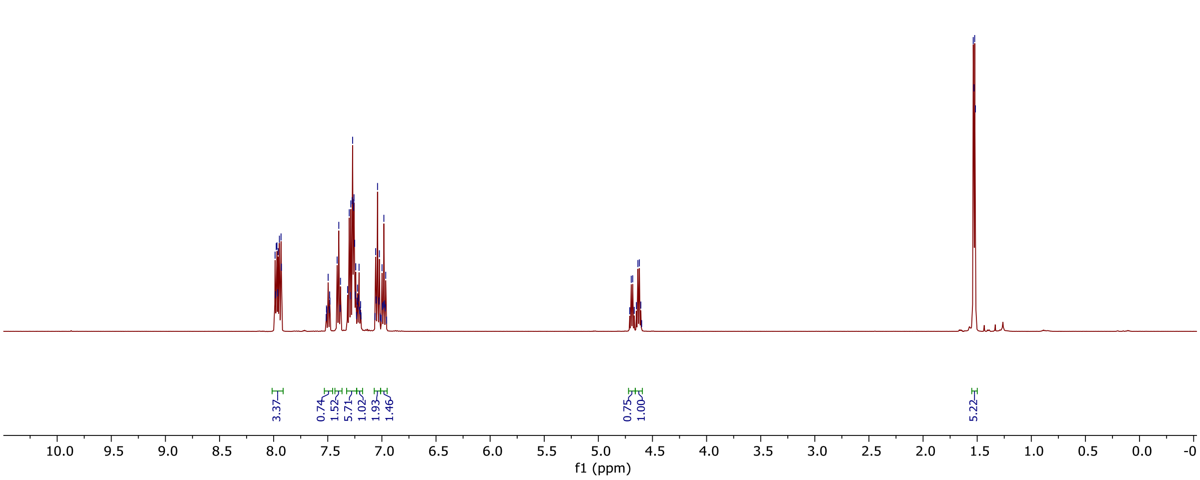

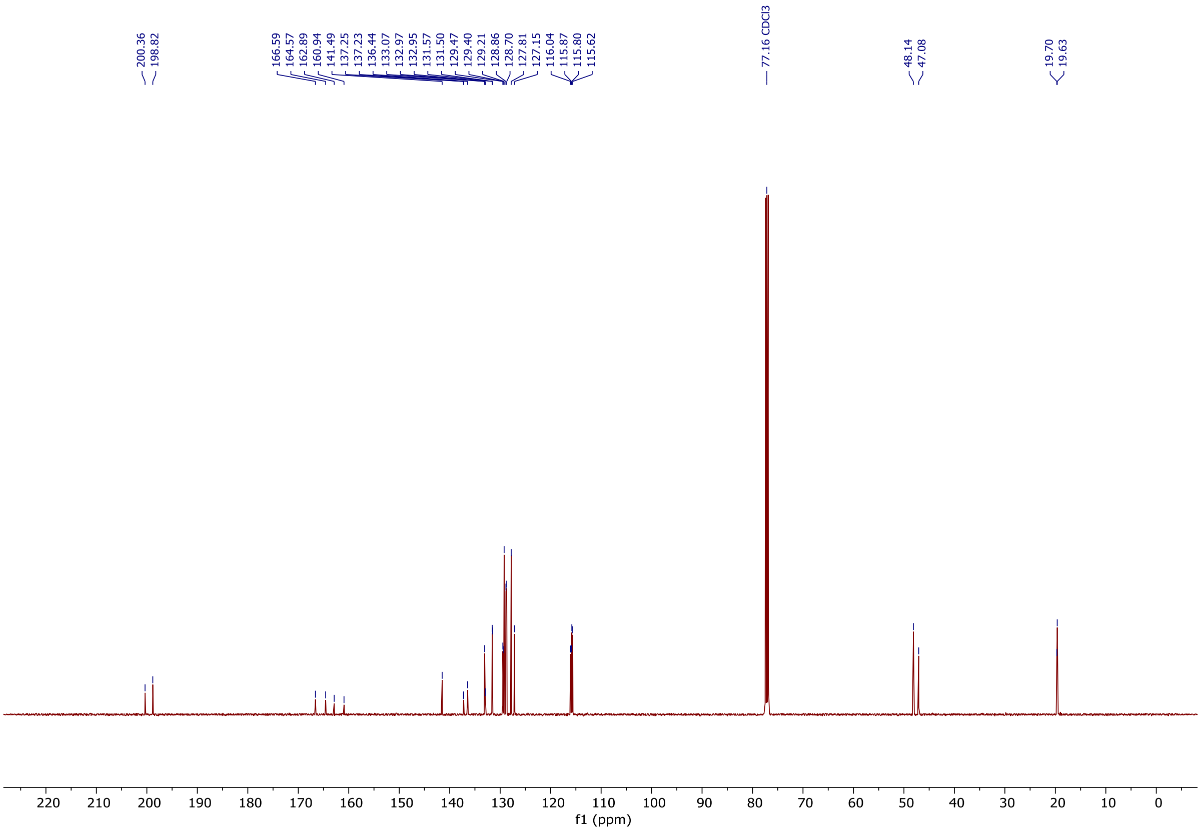

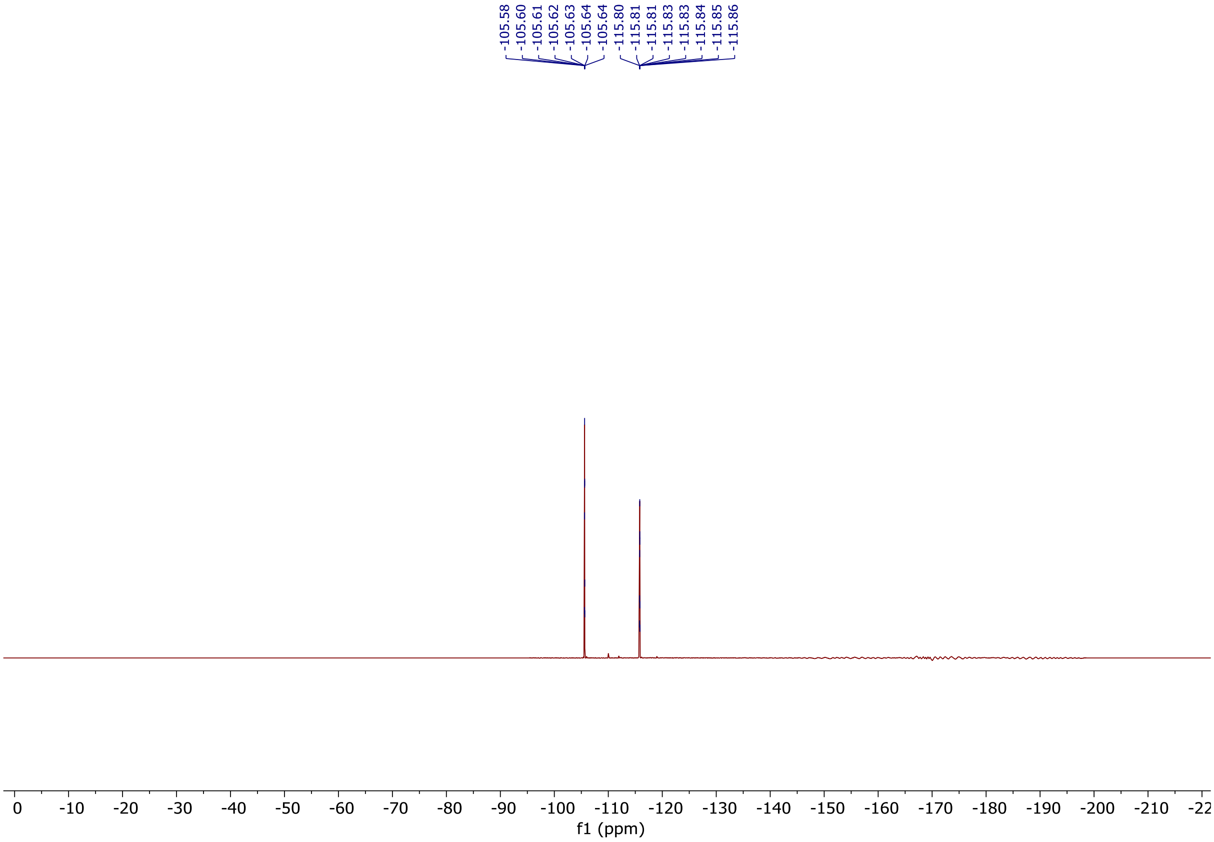

-105.58
-105.60
-105.61
-105.62
-105.63
-105.64
-105.64
-115.80
-115.81
-115.81
-115.83
-115.83
-115.84
-115.85
-115.86
0 -10 -20 -30 -40 -50 -60 -70 -80 -90 -100 -110 -120 -130 -140 -150 -160 -170 -180 -190 -200 -210 -22
f1 (ppm)

(±)-1-Phenyl-2-(4-chlorophenyl)propan-1-one (**(±)-4c**) and (±)-1-(4-chlorophenyl)-2-phenylpropan-1-one (**(±)-5c**)

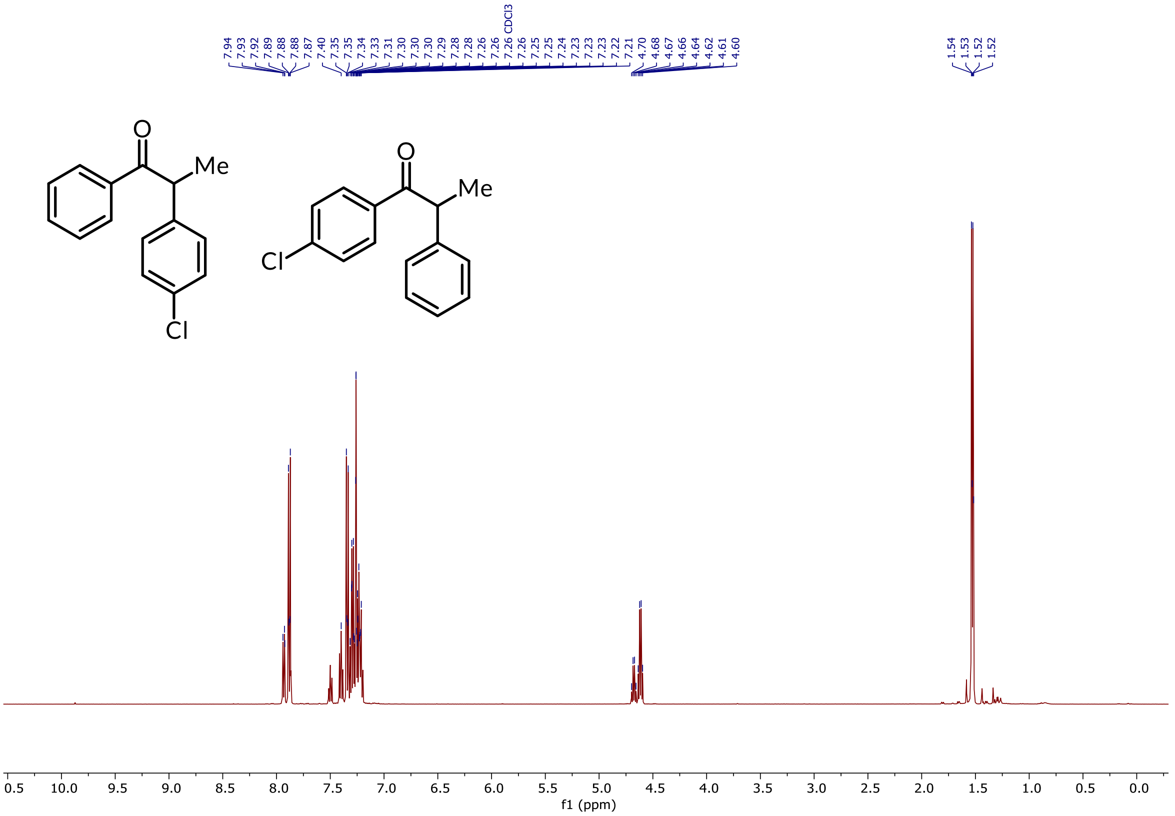

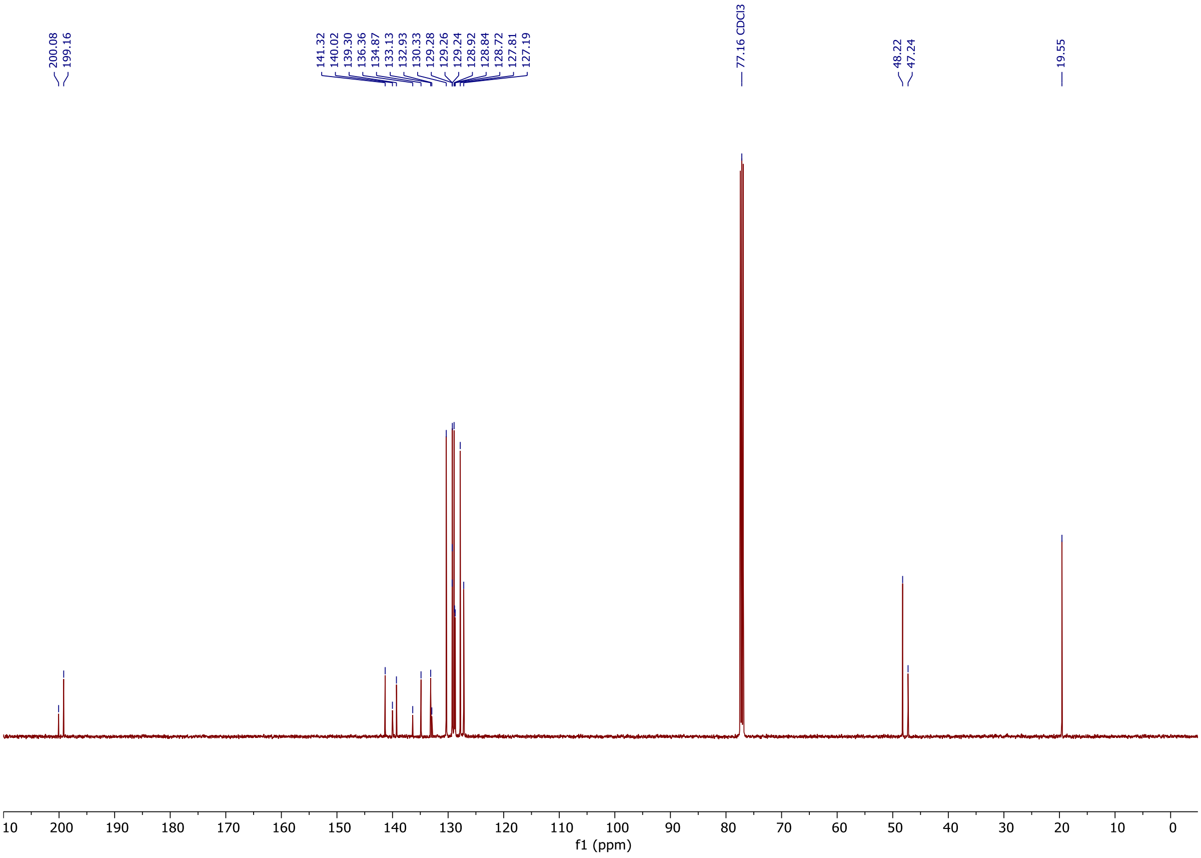

(±)-1-Phenyl-2-(3-fluorophenyl)propan-1-one (**(±)-4d**) and (±)-1-(3-fluorophenyl)-2-phenylpropan-1-one (**(±)-5d**)

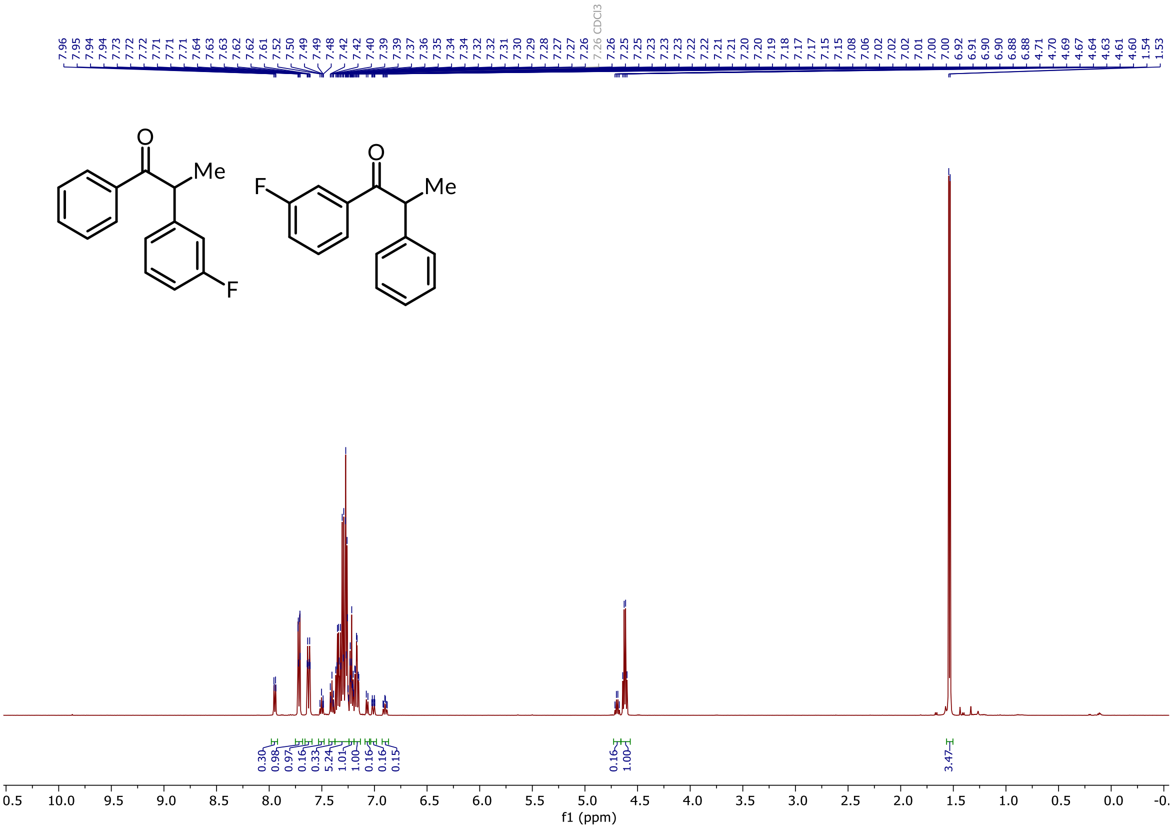

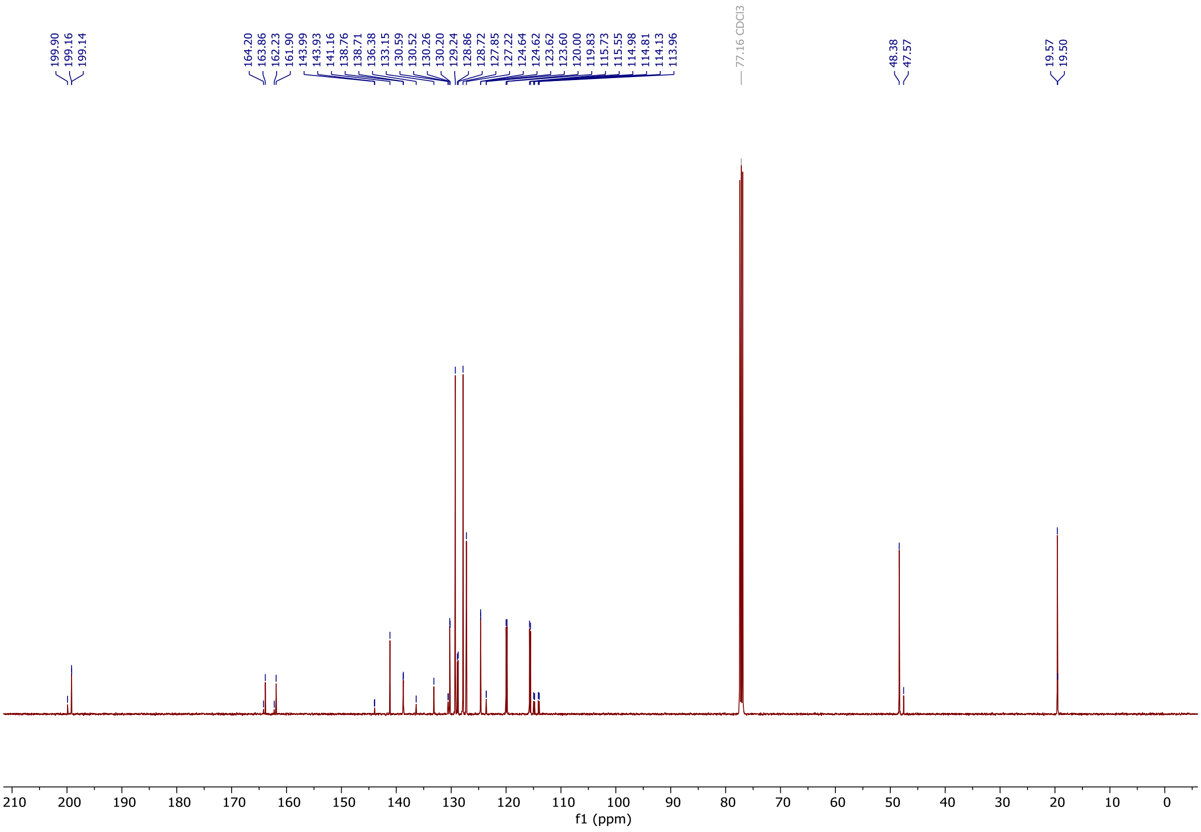

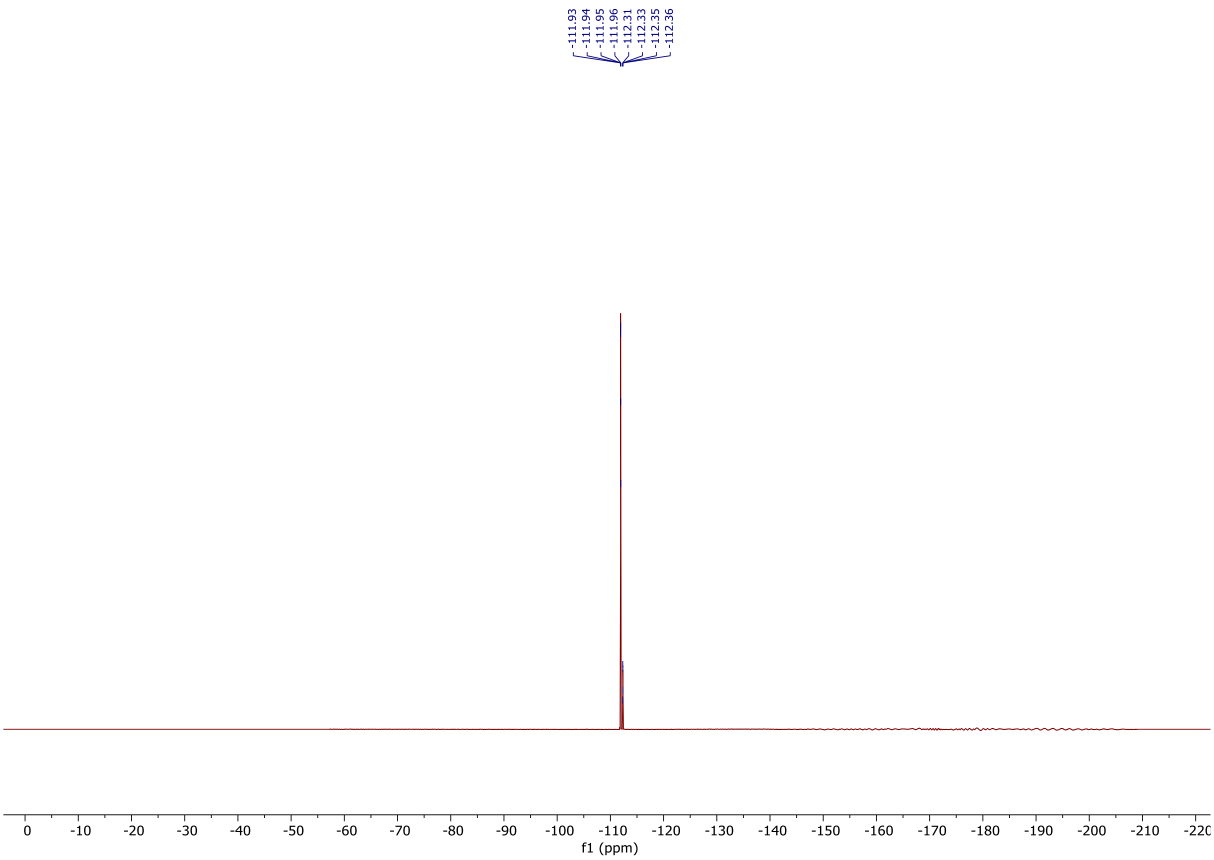

-111.93
-111.94
-111.95
-111.96
-112.31
-112.33
-112.35
-112.36
0
-10
-20
-30
-40
-50
-60
-70
-80
-90
-100
-110
-120
-130
-140
-150
-160
-170
-180
-190
-200
-210
-220
f1 (ppm)

(±)-1-Phenyl-2-(4-(trifluoromethyl)phenyl)propan-1-one (**(±)-4e**) and (±)-2-phenyl-1-(4-(trifluoromethyl)phenyl)propan-1-one (**(±)-5e**)

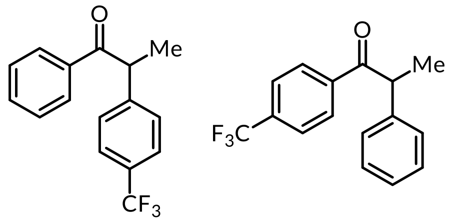

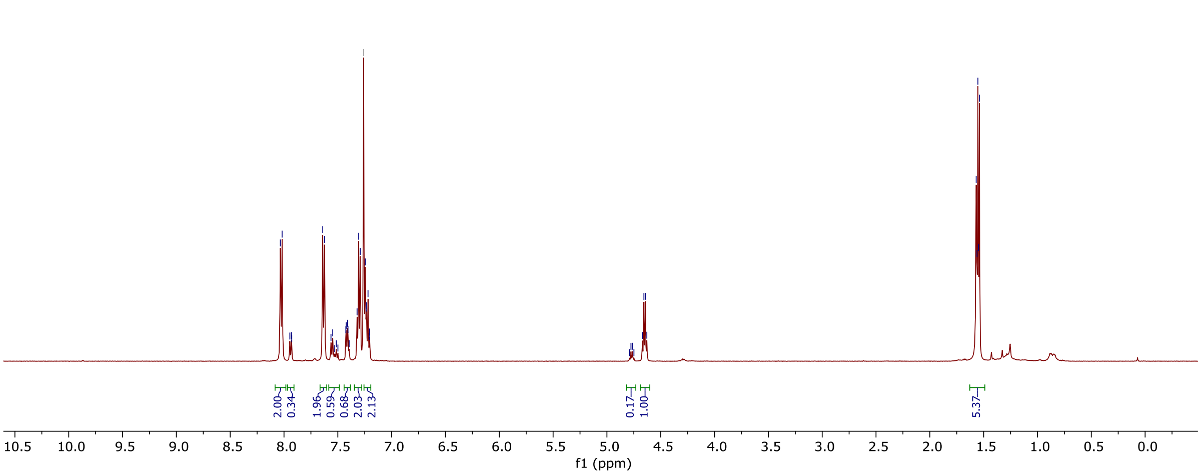

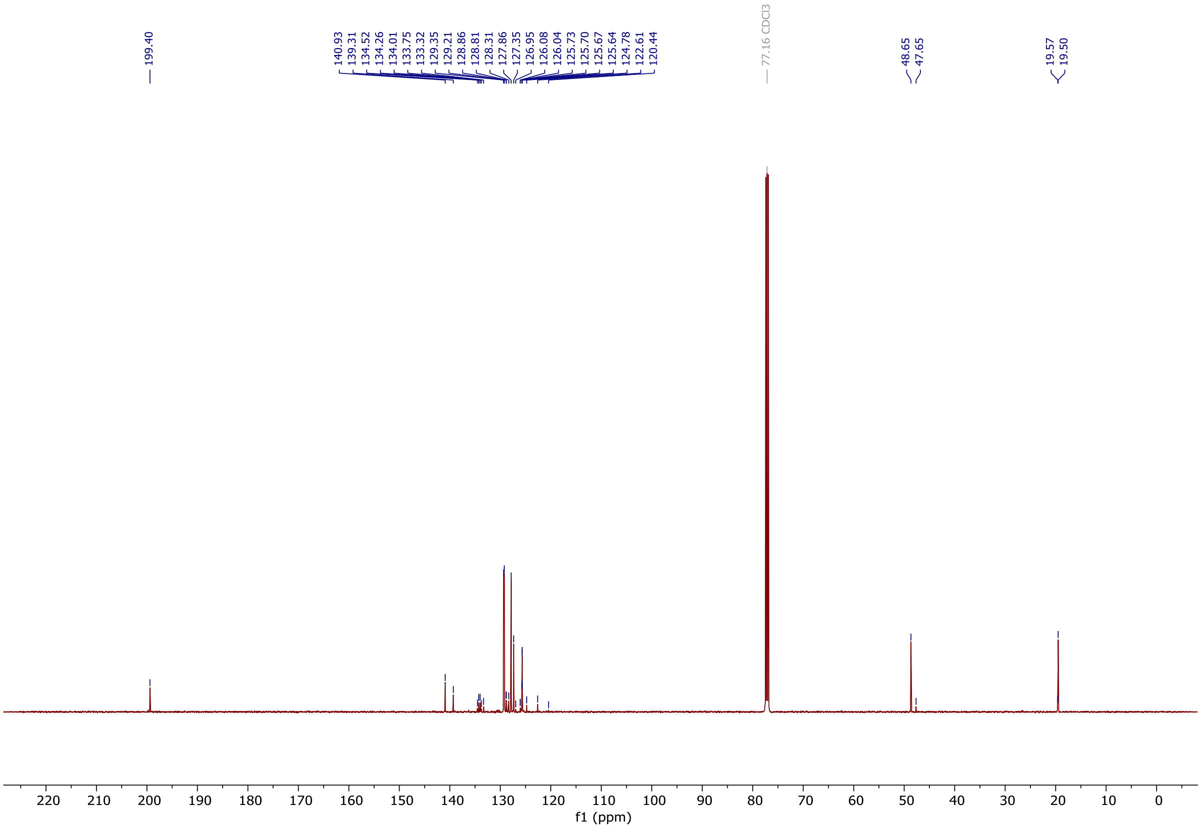

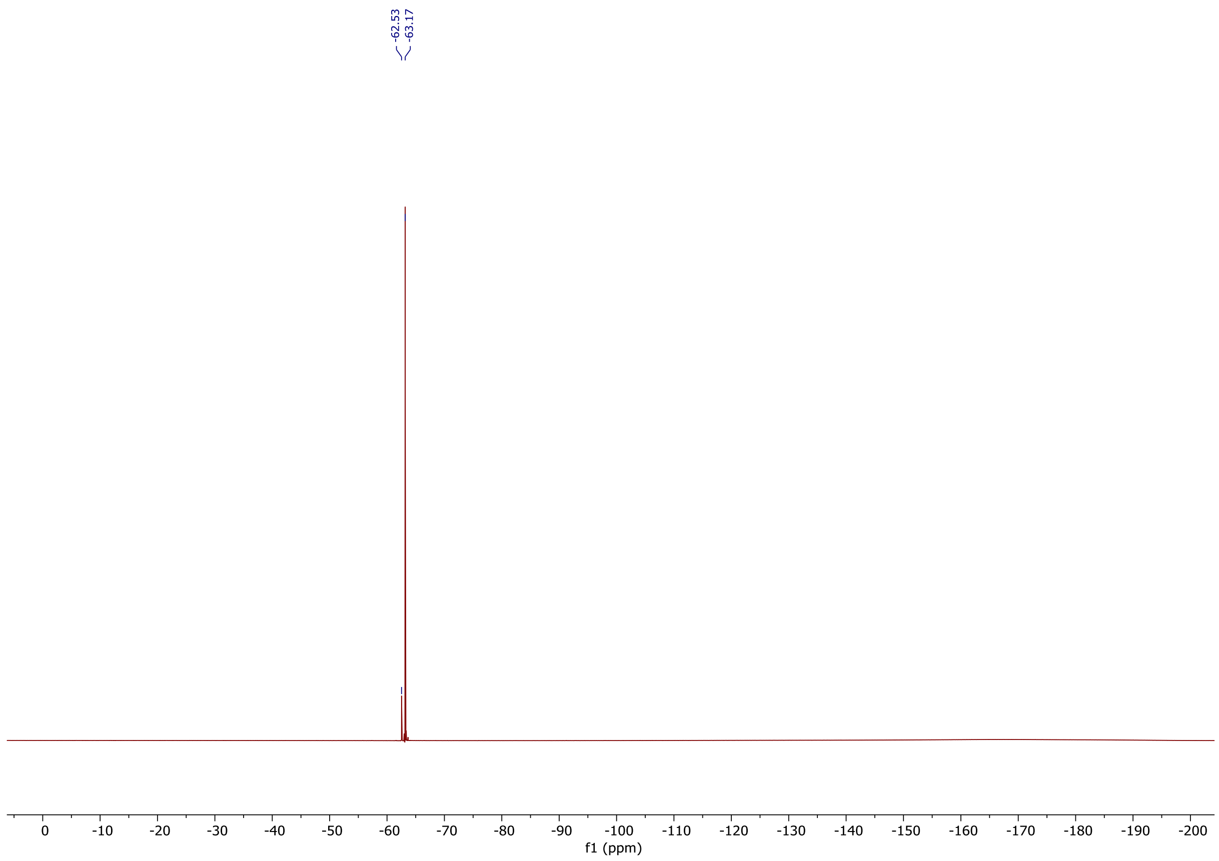

-62.53
-63.17
0
-10
-20
-30
-40
-50
-60
-70
-80
-90
-100
-110
-120
-130
-140
-150
-160
-170
-180
-190
-200
f1 (ppm)

(±)-1-(3-Fluoro-4-(trifluoromethyl)phenyl)-2-phenylpropan-1-one (**(±)-5f**)

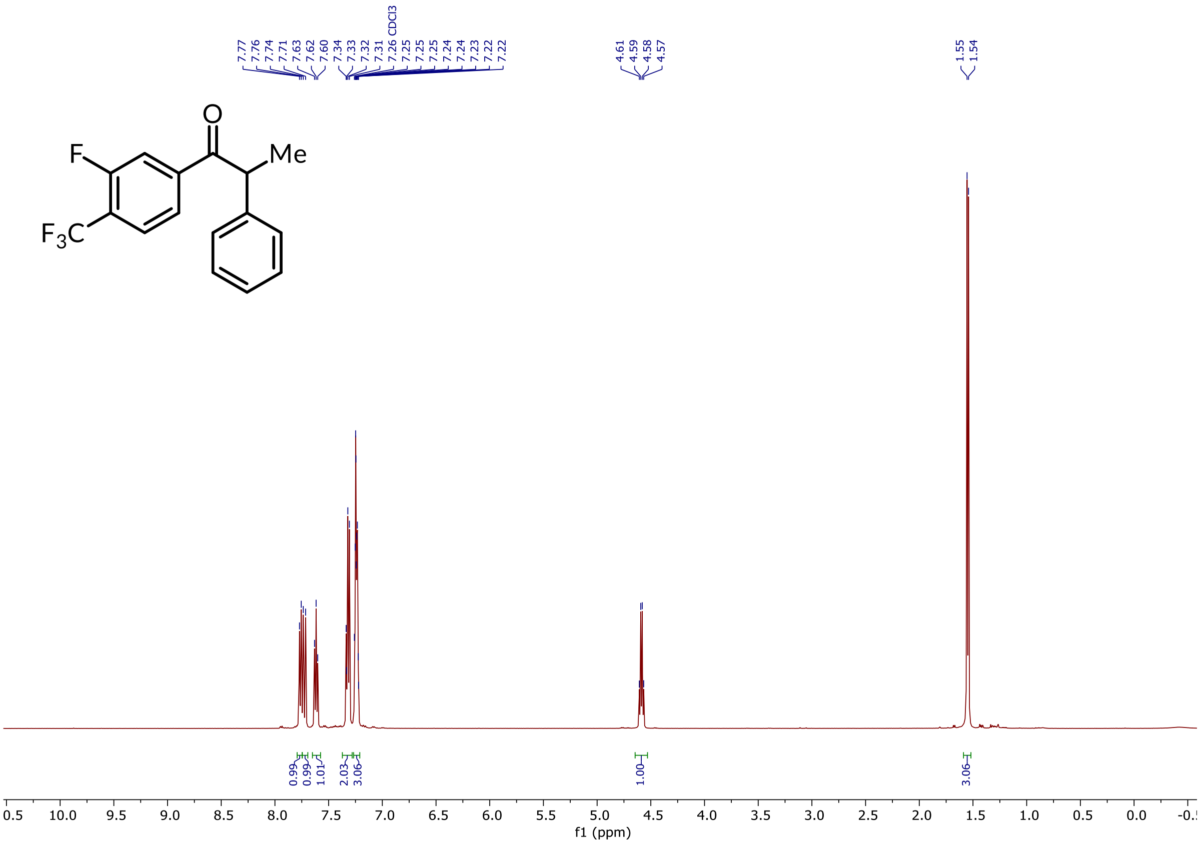

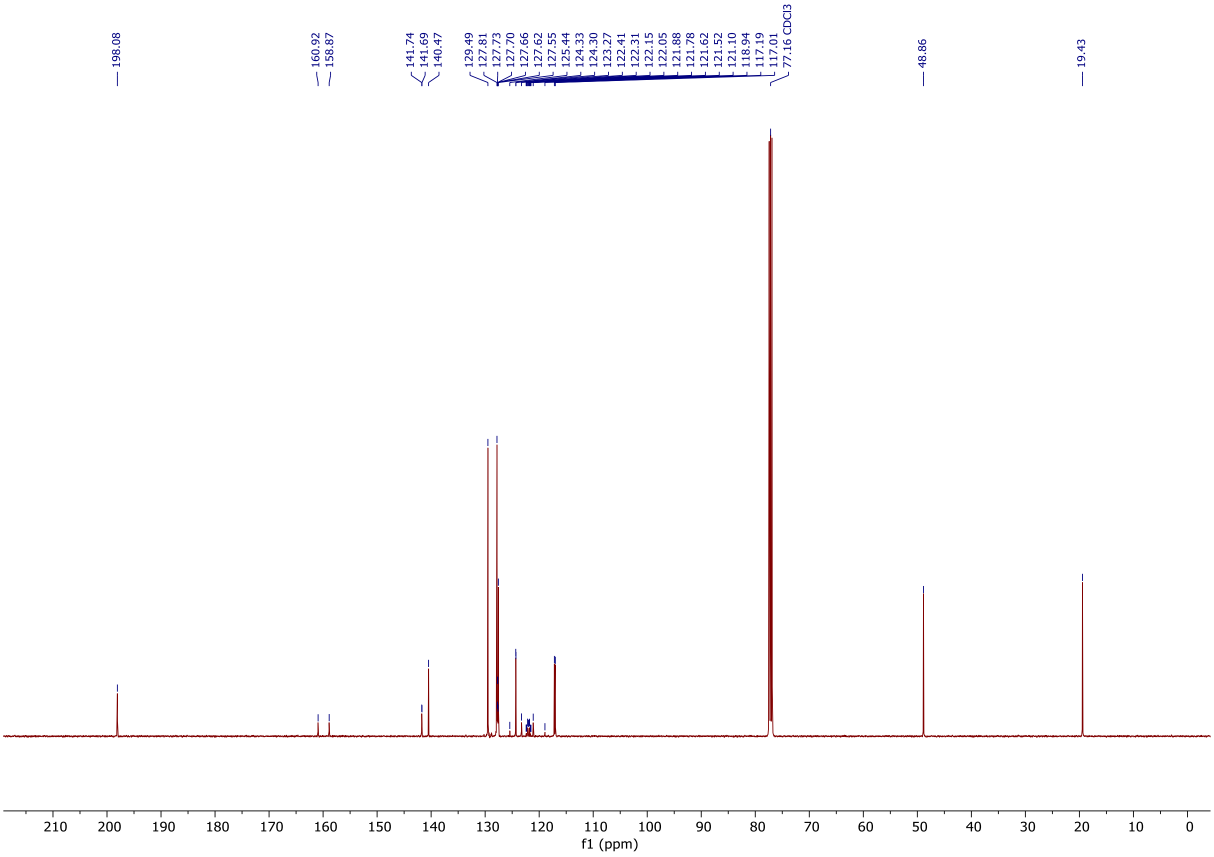

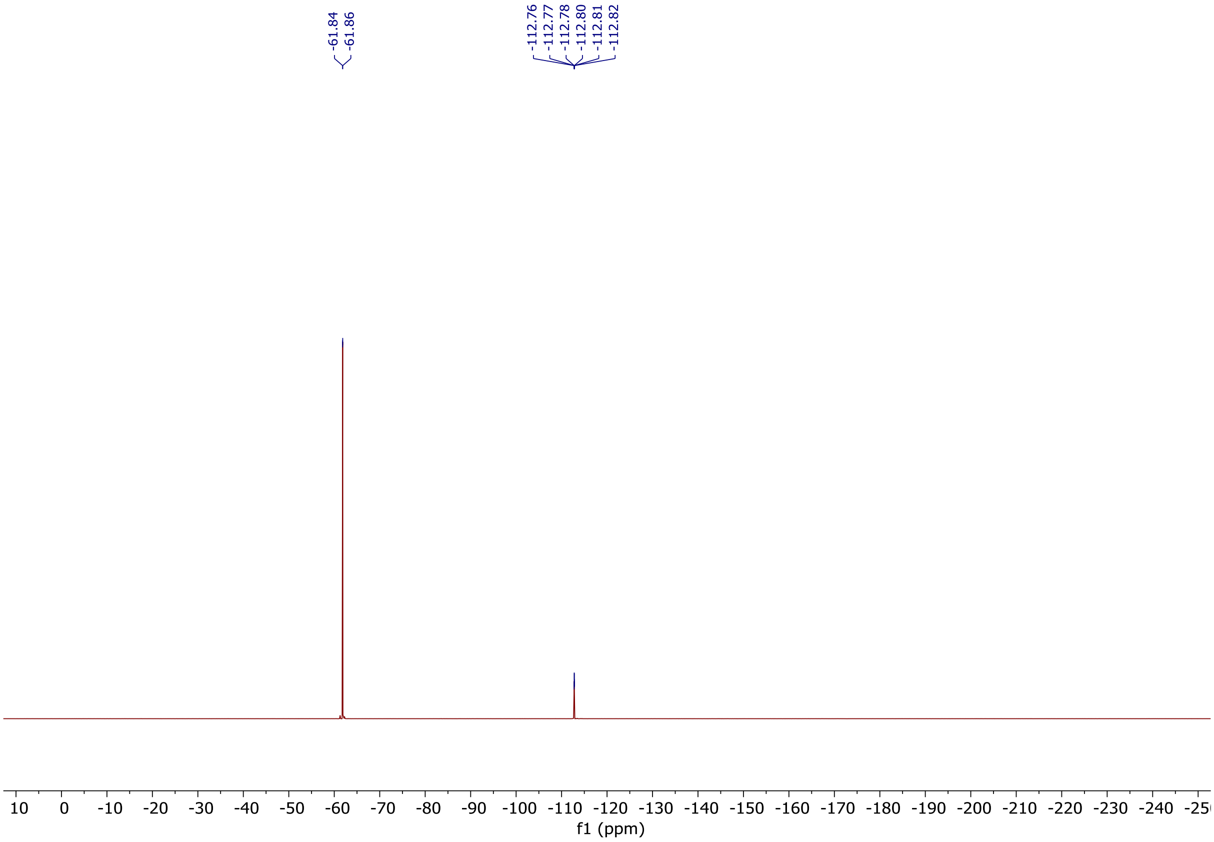

-61.84
-61.86
-112.76
-112.77
-112.78
-112.80
-112.81
-112.82
10 0 -10 -20 -30 -40 -50 -60 -70 -80 -90 -100 -110 -120 -130 -140 -150 -160 -170 -180 -190 -200 -210 -220 -230 -240
f1 (ppm)

Dimethyl(phenyl)silane-*d* (**Si-D$_1$**)

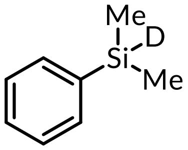

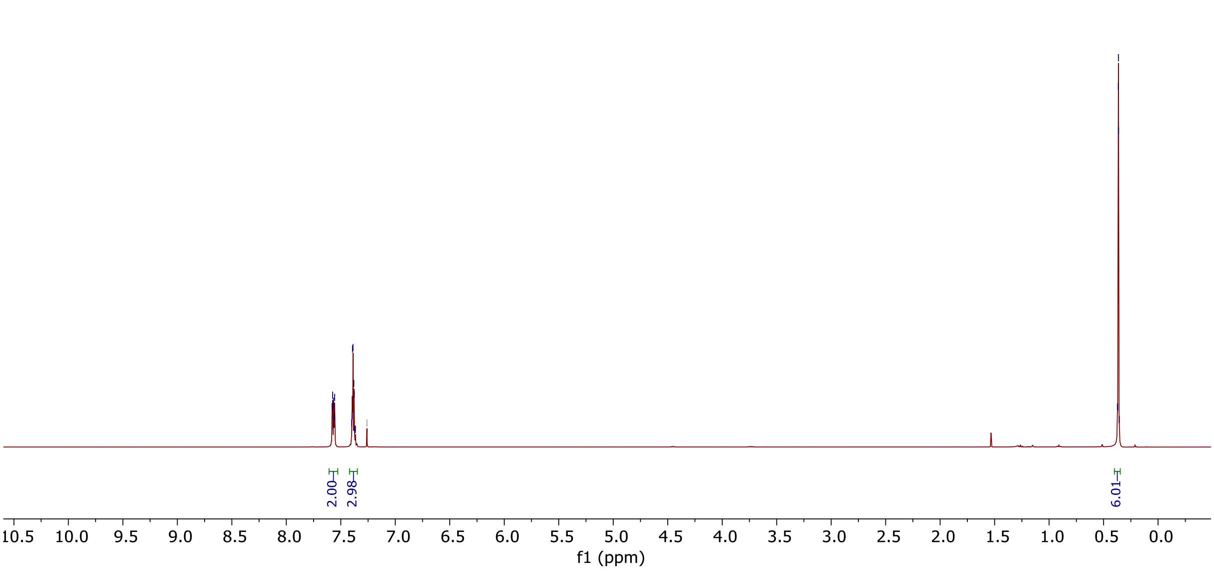

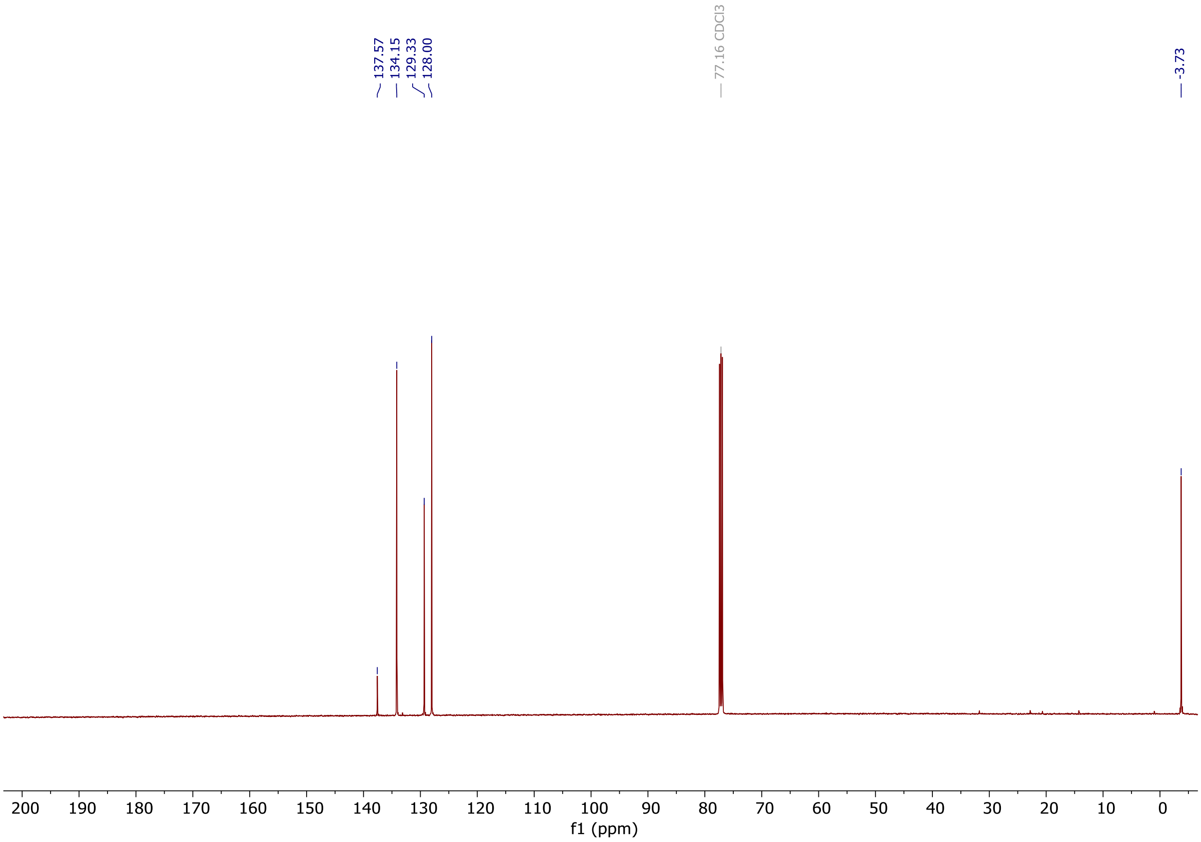

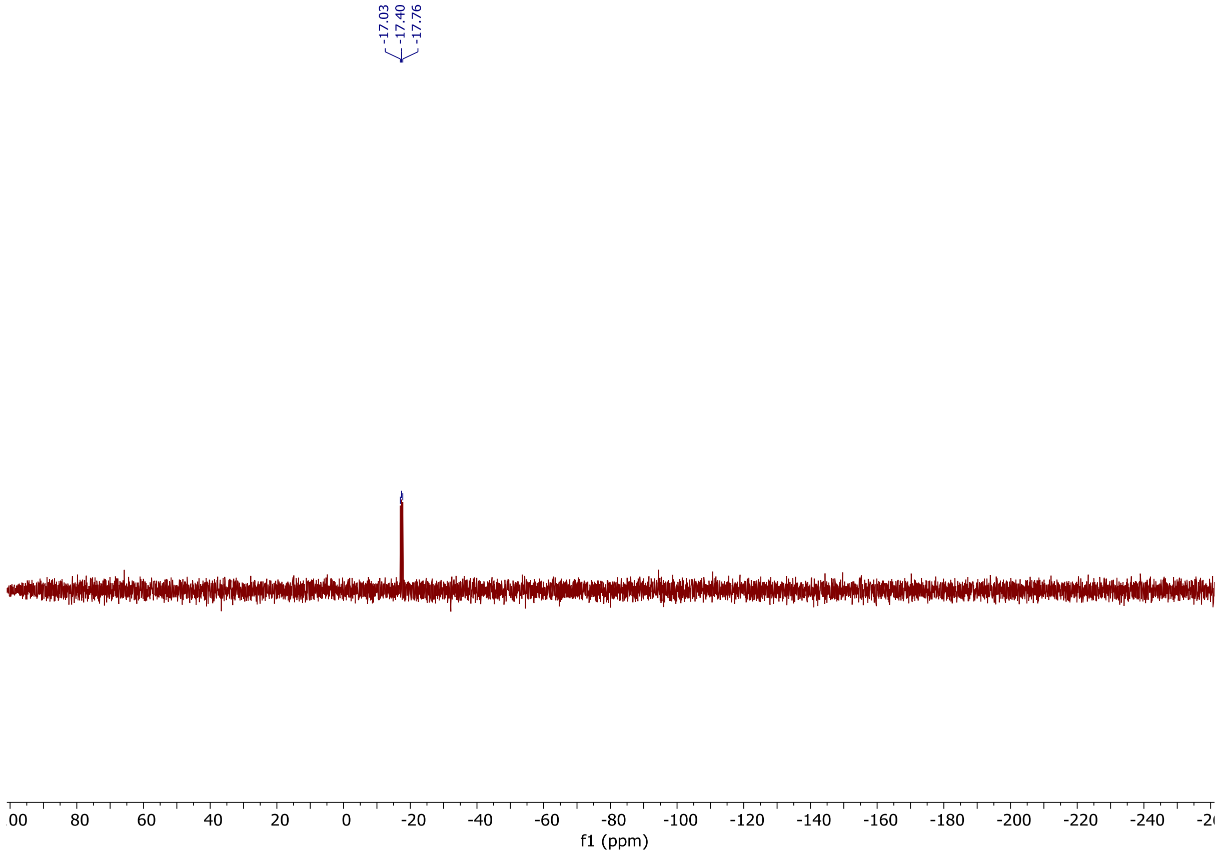
-17.03
-17.40
-17.76
f1 (ppm)

(*R*)-1,2-Diphenylpropan-1-one-3-*d* (**(*R*)-2b-D**$_1$)

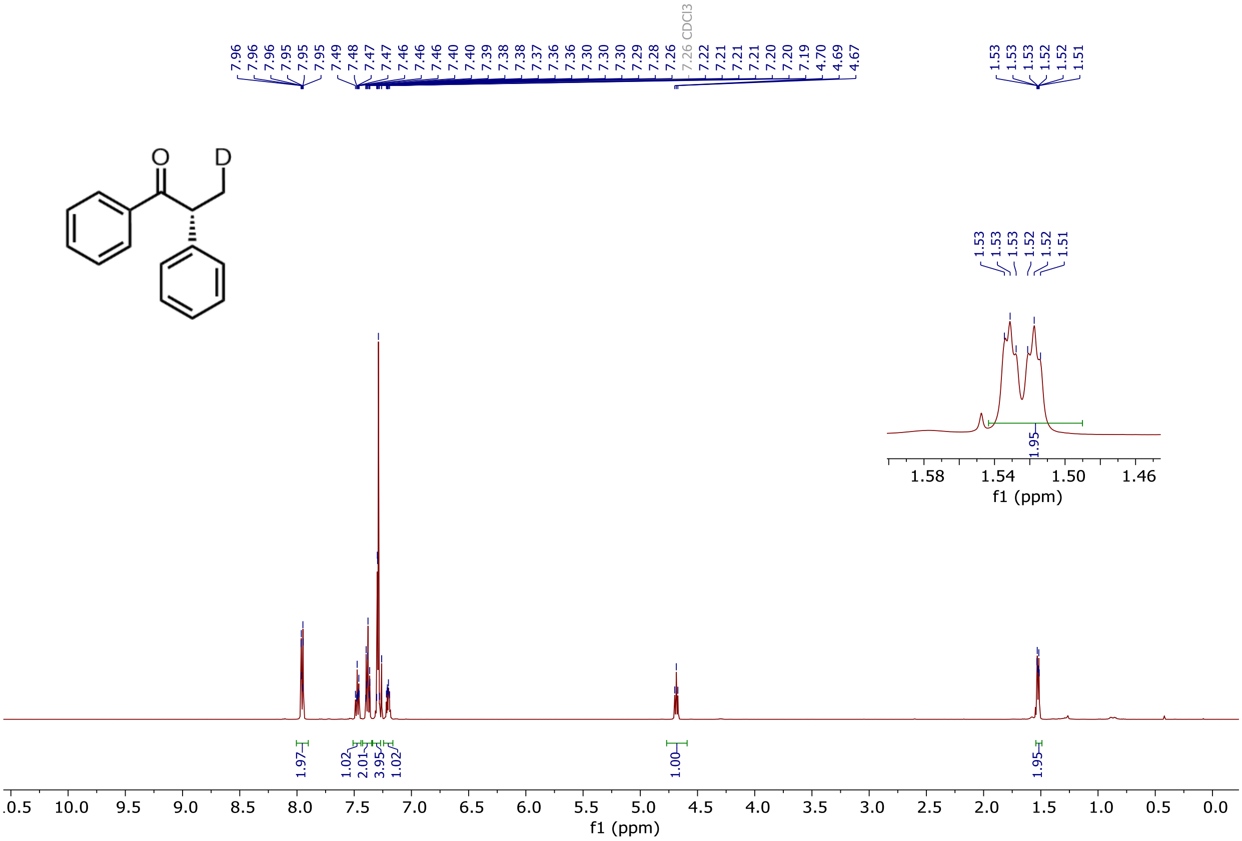

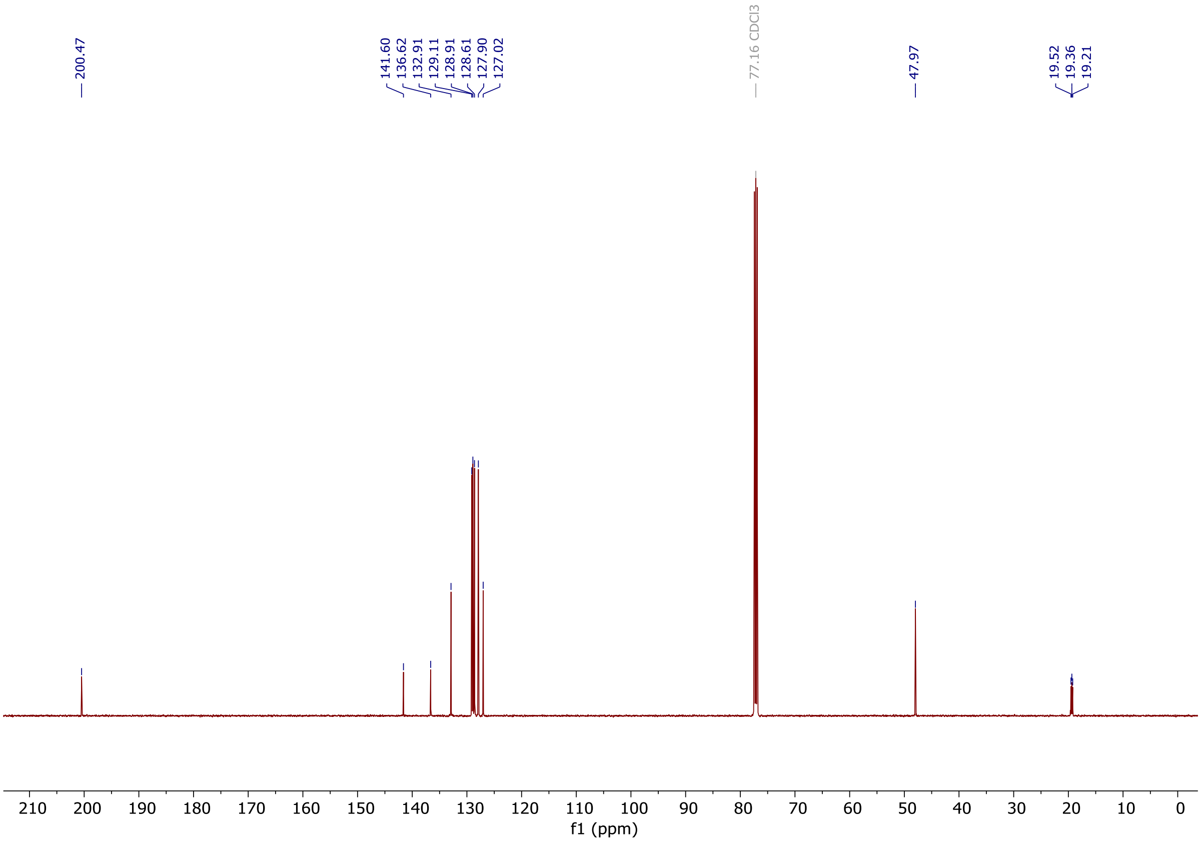

HPLC Data

6,6'-((1*E*,1'*E*)-(((1*R*,2*R*)-1,2-Dimesitylethane-1,2-diyl)bis(azaneylylidene))bis(methaneylylidene))bis(2,4-di-*tert*-butylphenol) (**(*R*,*R*)-Co(II)-5**)

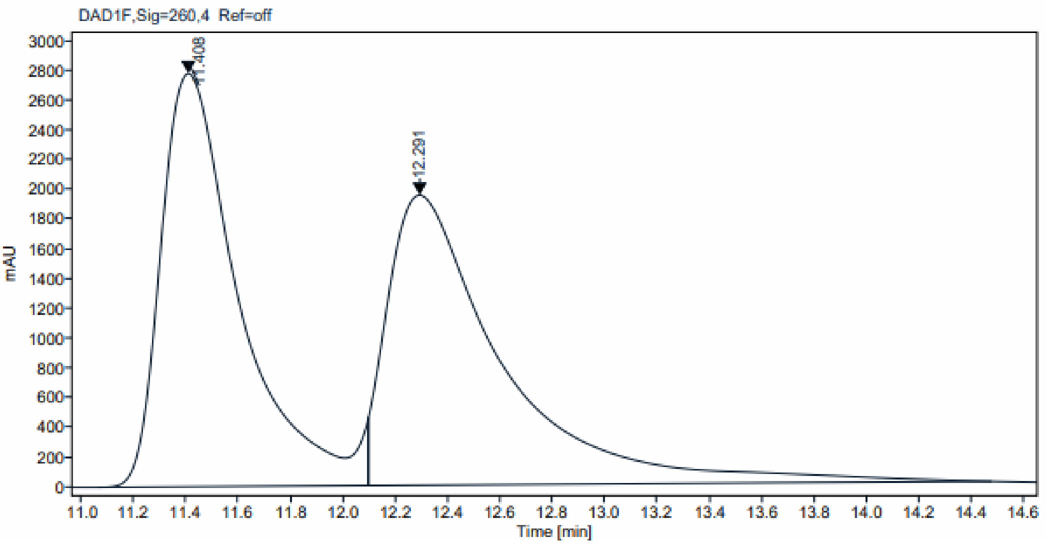

Signal: DAD1F,Sig=260,4 Ref=off

RT [min]	Type	Width [min]	Area	Height	Area%	Name
11.408	MM m	1.0319	57894.9737	2778.5781	49.4309	
12.291	MM m	2.3800	59228.0615	1951.8416	50.5691	

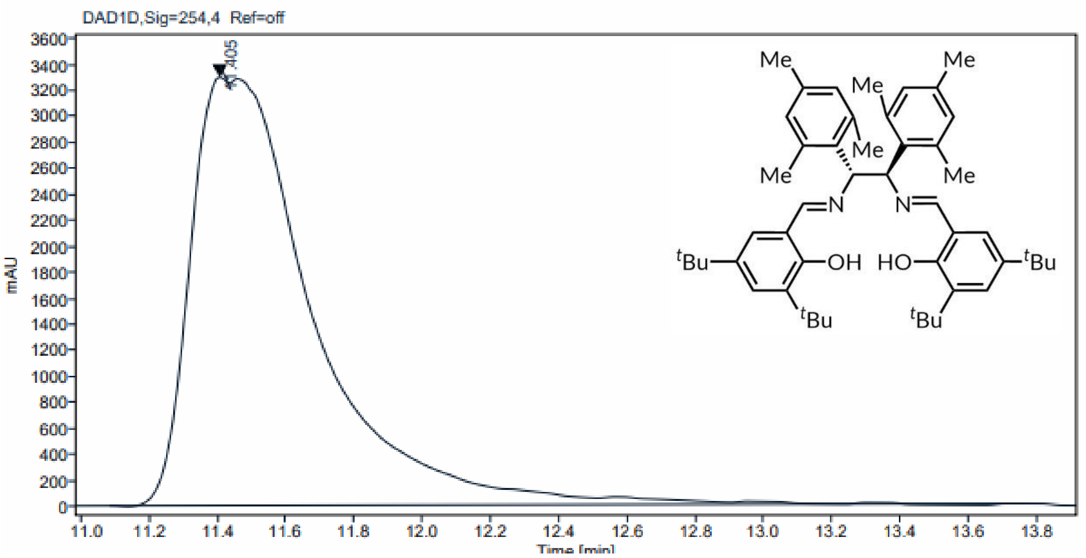

Signal: DAD1D,Sig=254,4 Ref=off

RT [min]	Type	Width [min]	Area	Height	Area%	Name
11.405	MM m	2.6748	82376.4824	3293.4985	100.0000	

(*R*)-1,2-Bis(4-fluorophenyl)propan-1-one ((***R*)-2a**)

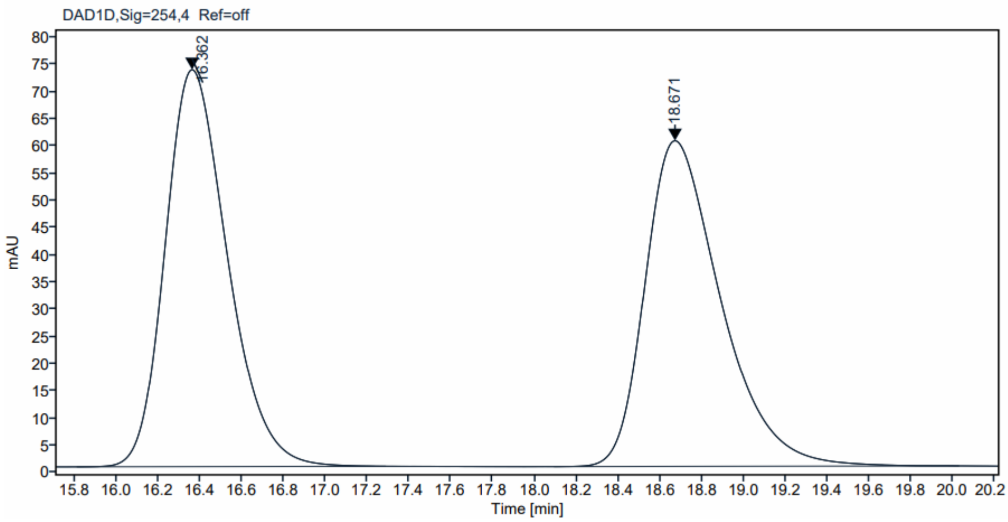

Signal: DAD1D,Sig=254,4 Ref=off

RT [min]	Type	Width [min]	Area	Height	Area%	Name
16.362	MM m	1.4780	1511.4281	72.8900	50.1192	
18.671	MM m	1.9372	1504.2395	59.8058	49.8808	

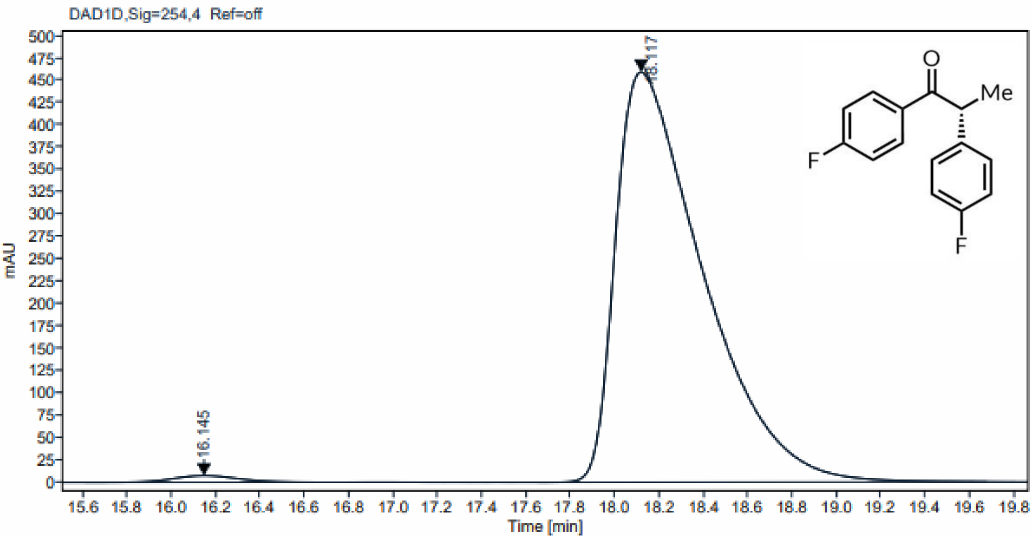

Signal: DAD1D,Sig=254,4 Ref=off

RT [min]	Type	Width [min]	Area	Height	Area%	Name
16.145	MM m	1.0884	150.5091	7.5200	1.1827	
18.117	MM m	2.0222	12575.4349	459.6055	98.8173	

(*R*)-1,2-Diphenylpropan-1-one ((***R*)-2b**)

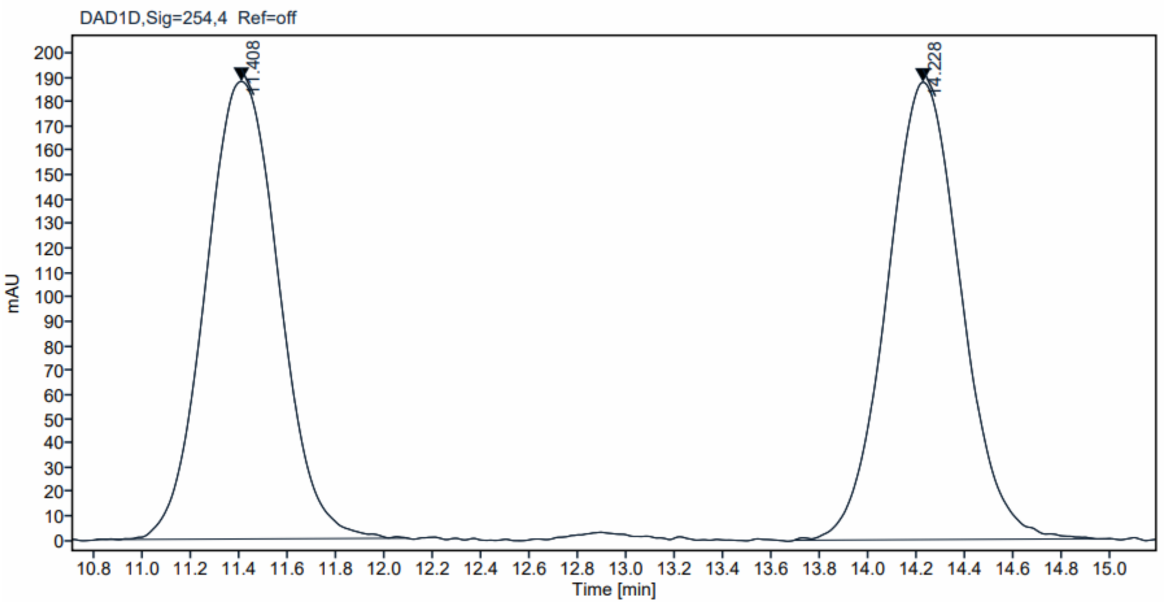

Signal:	DAD1D,Sig=254,4 Ref=off				
RT [min]	Type	Width [min]	Area	Height	Area% Name
11.408	MM m	1.2888	3980.2478	187.6761	49.8690
14.228	MM m	1.3090	4001.1670	187.6299	50.1310

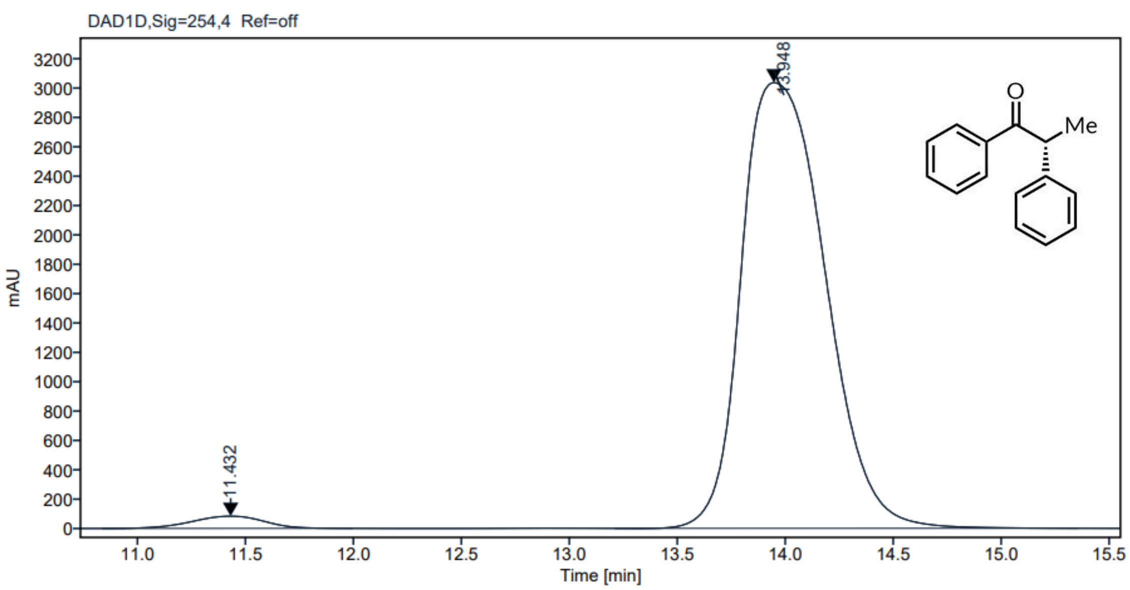

Signal:	DAD1D,Sig=254,4 Ref=off				
RT [min]	Type	Width [min]	Area	Height	Area% Name
11.432	MM m	1.1602	1951.7438	83.1752	2.3258
13.948	MM m	2.1469	81964.6645	3036.0420	97.6742

(*R*)-1,2-Bis(4-methylphenyl)propan-1-one (**(*R*)-2c**)

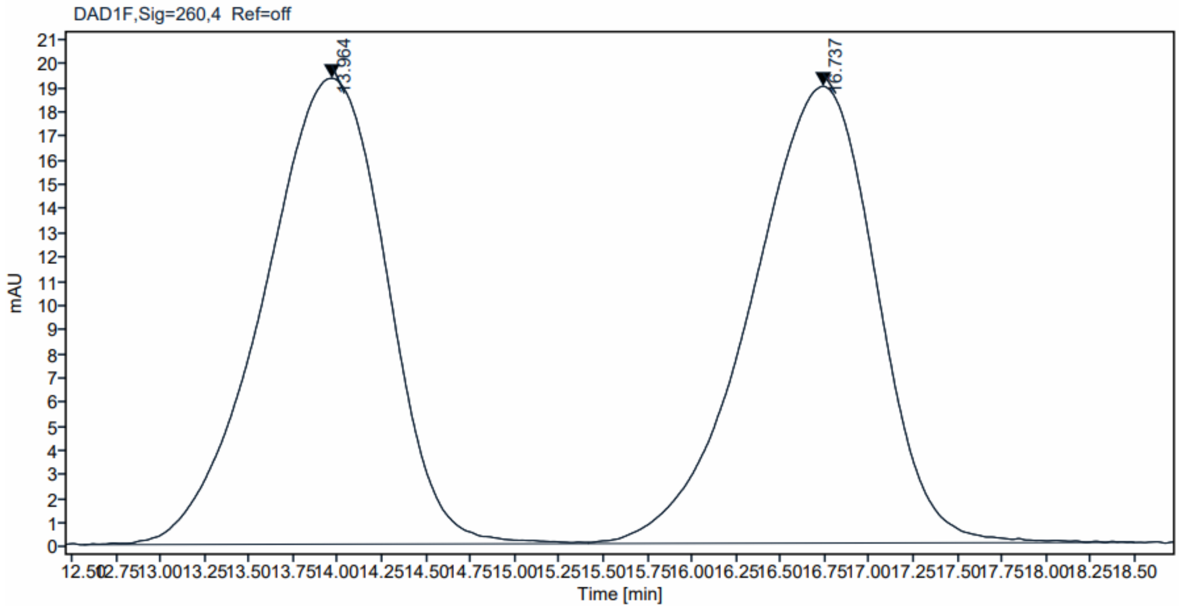

Signal:	DAD1F,Sig=260,4 Ref=off					
RT [min]	Type	Width [min]	Area	Height	Area%	Name
13.964	MM m	2.8505	930.5249	19.2711	49.9833	
16.737	MM m	3.0808	931.1467	18.9008	50.0167	

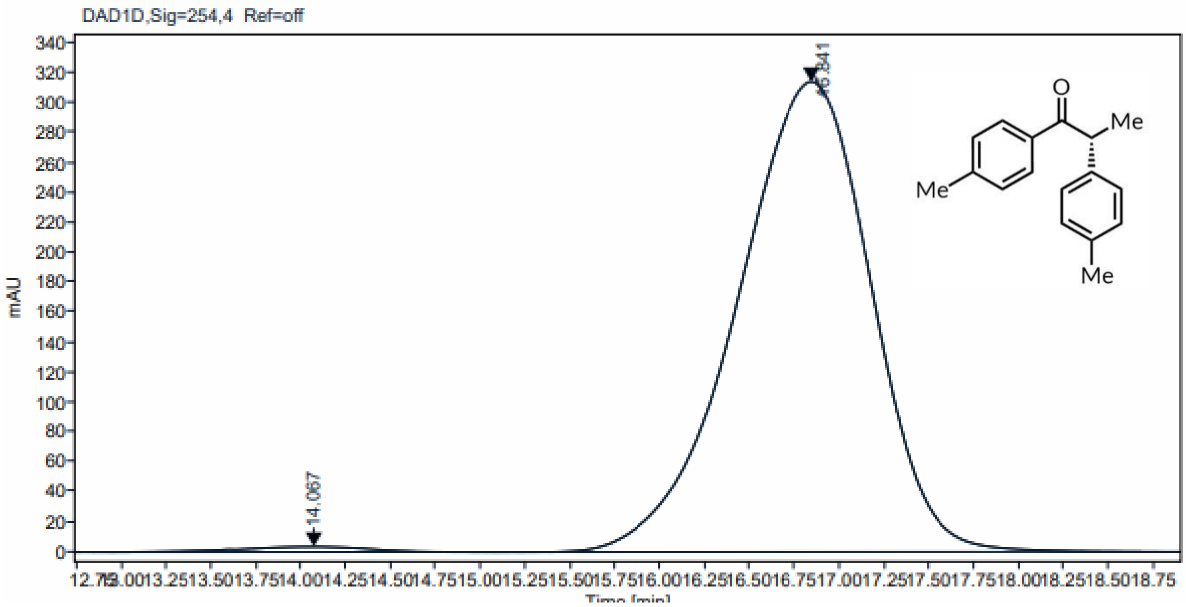

Signal:	DAD1D,Sig=254,4 Ref=off					
RT [min]	Type	Width [min]	Area	Height	Area%	Name
14.067	MM m	1.9455	169.1023	3.4863	1.0281	
16.841	MM m	3.3014	16278.2098	314.3450	98.9719	

(*R*)-1,2-Bis(4-(isopropyl)phenyl)propan-1-one (***(R)*-2d**)

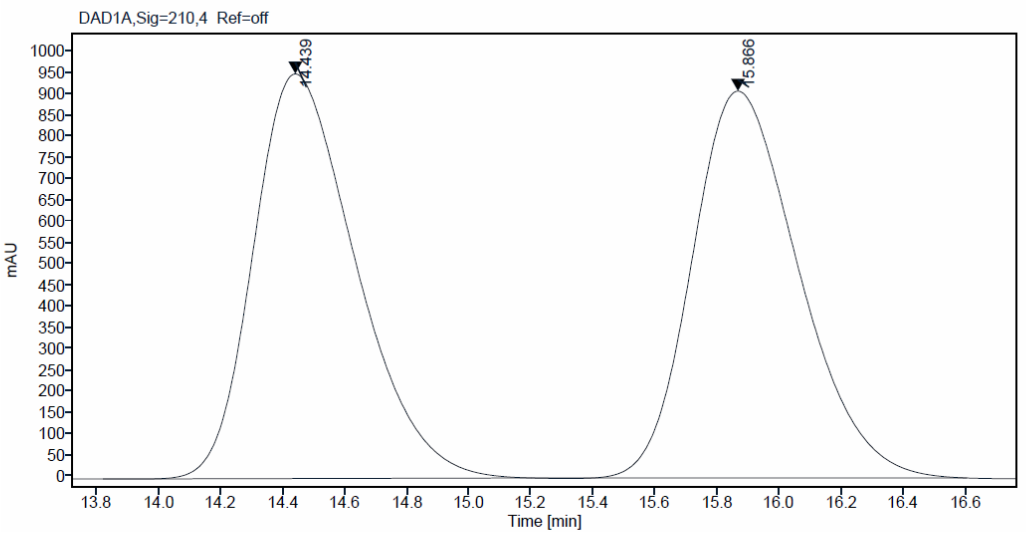

Signal: DAD1A,Sig=210,4 Ref=off

RT [min]	Type	Width [min]	Area	Height	Area%	Name
14.439	MM m	1.3790	21825.0884	953.6957	50.0652	
15.866	MM m	1.4055	21768.2585	911.7391	49.9348	

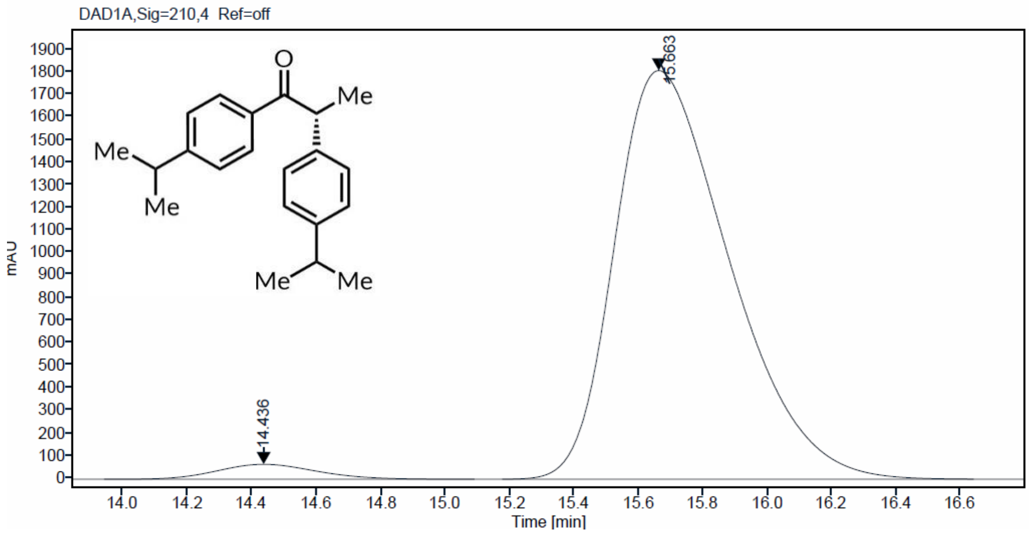

Signal: DAD1A,Sig=210,4 Ref=off

RT [min]	Type	Width [min]	Area	Height	Area%	Name
14.436	MM m	1.1500	1444.6229	66.4765	3.1114	
15.663	MM m	1.4643	44985.9715	1812.6913	96.8886	

(*R*)-1,2-Bis(4-(*tert*-butyl)phenyl)propan-1-one (**(*R*)-2e**)

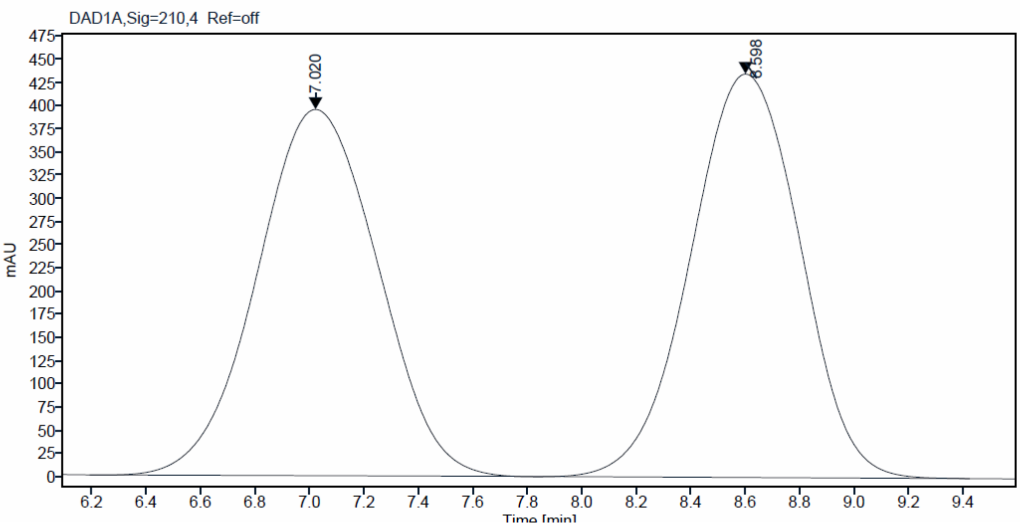

Signal: DAD1A,Sig=210,4 Ref=off

RT [min]	Type	Width [min]	Area	Height	Area%	Name
7.020	MM m	1.5584	12139.1558	394.2486	50.0370	
8.598	MM m	1.6713	12121.2128	434.3082	49.9630	

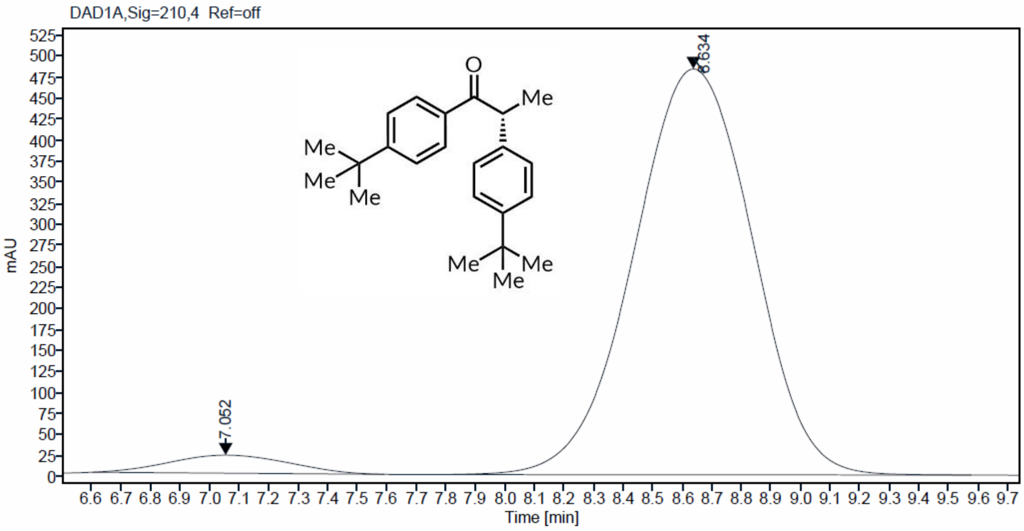

Signal: DAD1A,Sig=210,4 Ref=off

RT [min]	Type	Width [min]	Area	Height	Area%	Name
7.052	MM m	0.9863	605.8352	21.4649	4.2858	
8.634	MM m	1.8113	13530.1419	483.2120	95.7142	

(*R*)-1,2-Di([1,1′-biphenyl]-4-yl)propan-1-one (**(*R*)-2f**)

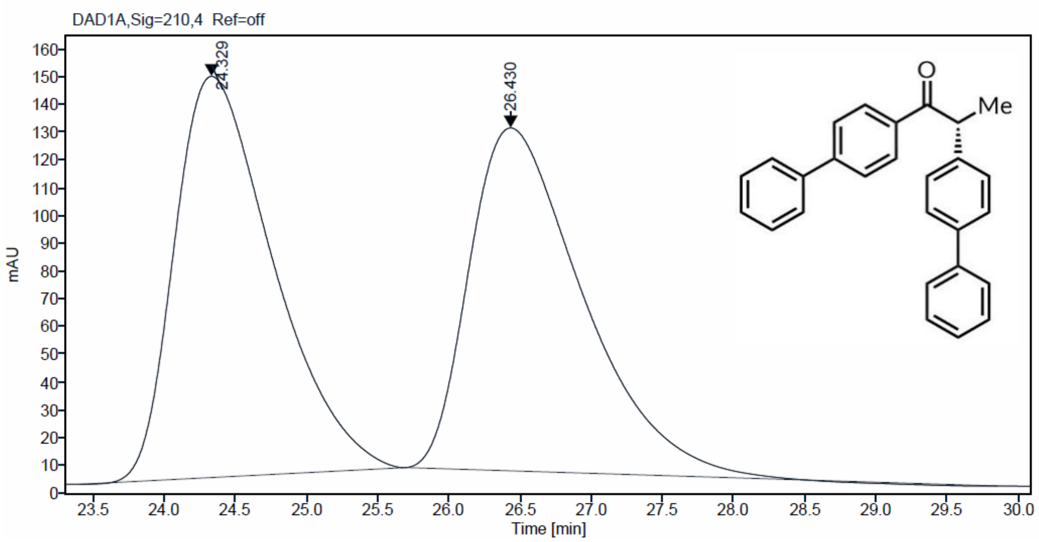

Signal: DAD1A,Sig=210,4 Ref=off

RT [min]	Type	Width [min]	Area	Height	Area%	Name
24.329	MM m	2.2888	6949.2497	144.5979	50.3616	
26.430	MM m	4.1559	6849.4569	123.5969	49.6384	

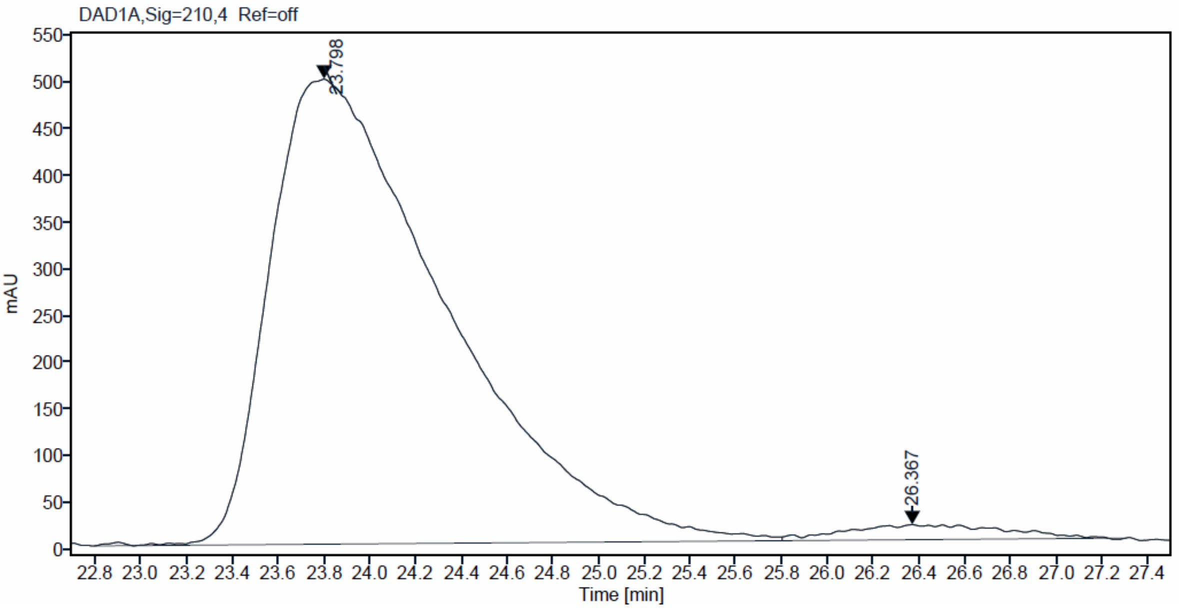

Signal: DAD1A,Sig=210,4 Ref=off

RT [min]	Type	Width [min]	Area	Height	Area%	Name
23.798	MM m	3.0040	26732.1495	497.3181	97.1848	
26.367	MM m	1.5020	774.3654	15.9871	2.8152	

(*R*)-1,2-Bis(4-bromophenyl)propan-1-one (**(*R*)-2g**)

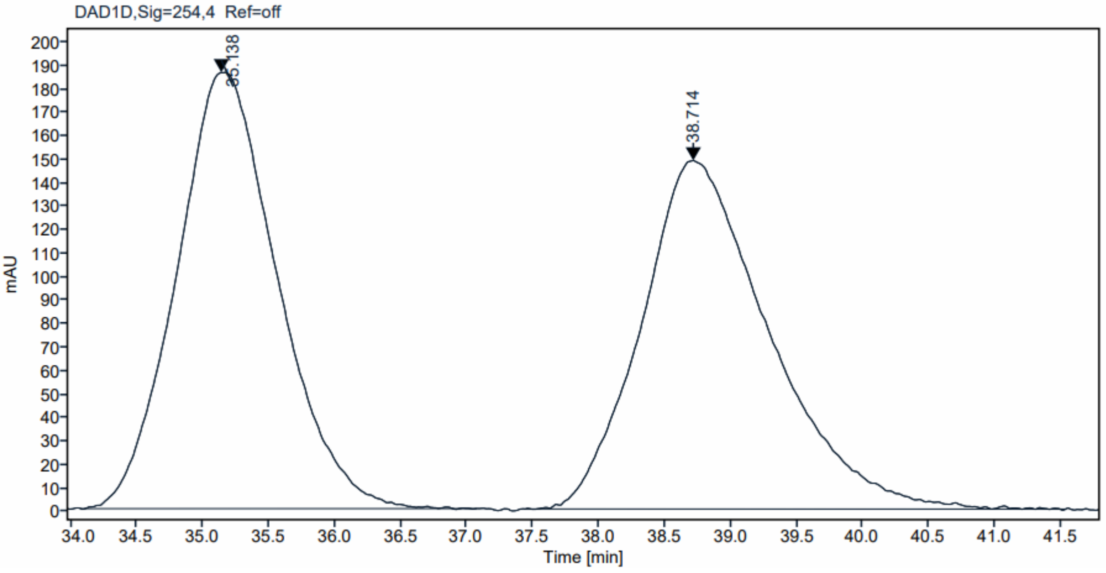

Signal: DAD1D,Sig=254,4 Ref=off

RT [min]	Type	Width [min]	Area	Height	Area%	Name
35.138	MM m	3.0001	9863.7644	185.8233	49.7359	
38.714	MM m	4.1019	9968.5176	148.4194	50.2641	

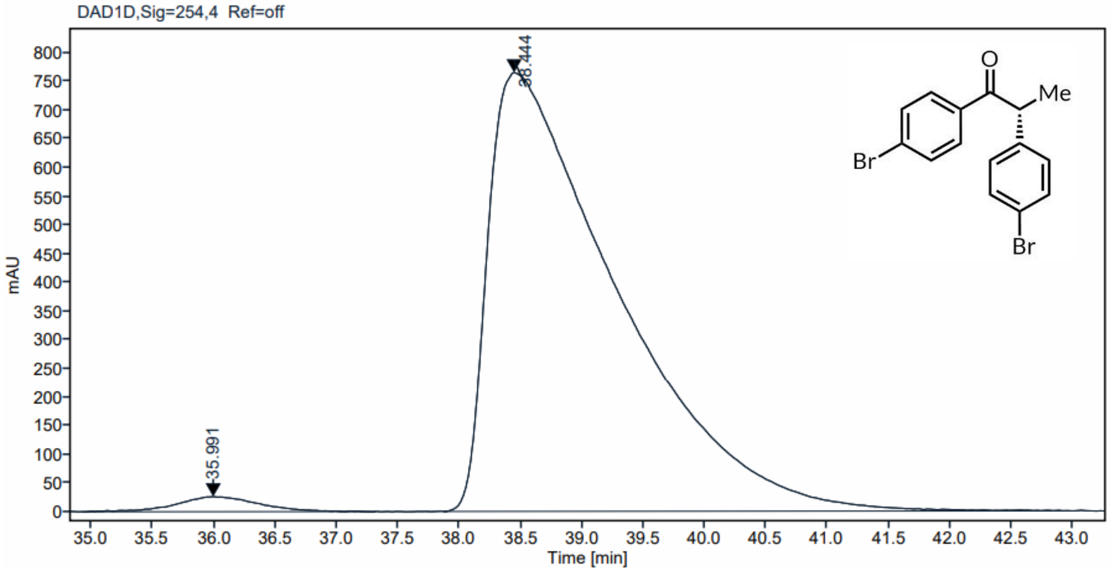

Signal: DAD1D,Sig=254,4 Ref=off

RT [min]	Type	Width [min]	Area	Height	Area%	Name
35.991	MM m	2.3891	1209.2940	26.1260	2.1088	
38.444	MM m	5.1274	56134.7394	765.0172	97.8912	

(*R*)-1,2-Bis(4-chlorophenyl)propan-1-one (**(*R*)-2h)**

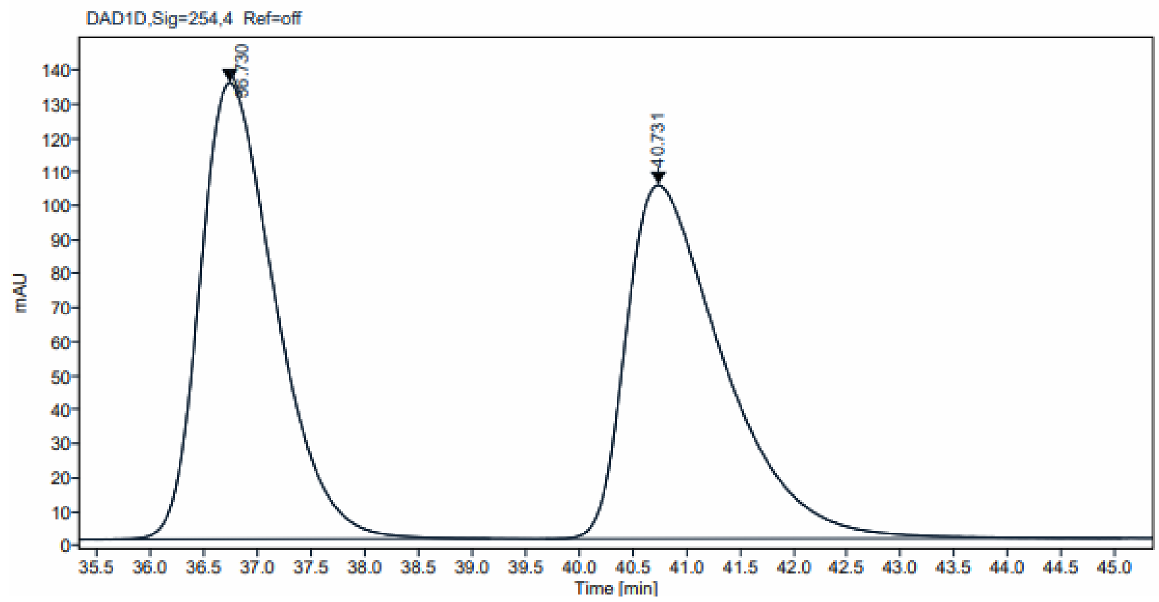

Signal:	DAD1D,Sig=254,4 Ref=off				
RT [min]	Type	Width [min]	Area	Height	Area% Name
36.730	MM m	3.7405	6567.9956	134.3022	49.9887
40.731	MM m	5.3763	6570.9693	104.0497	50.0113

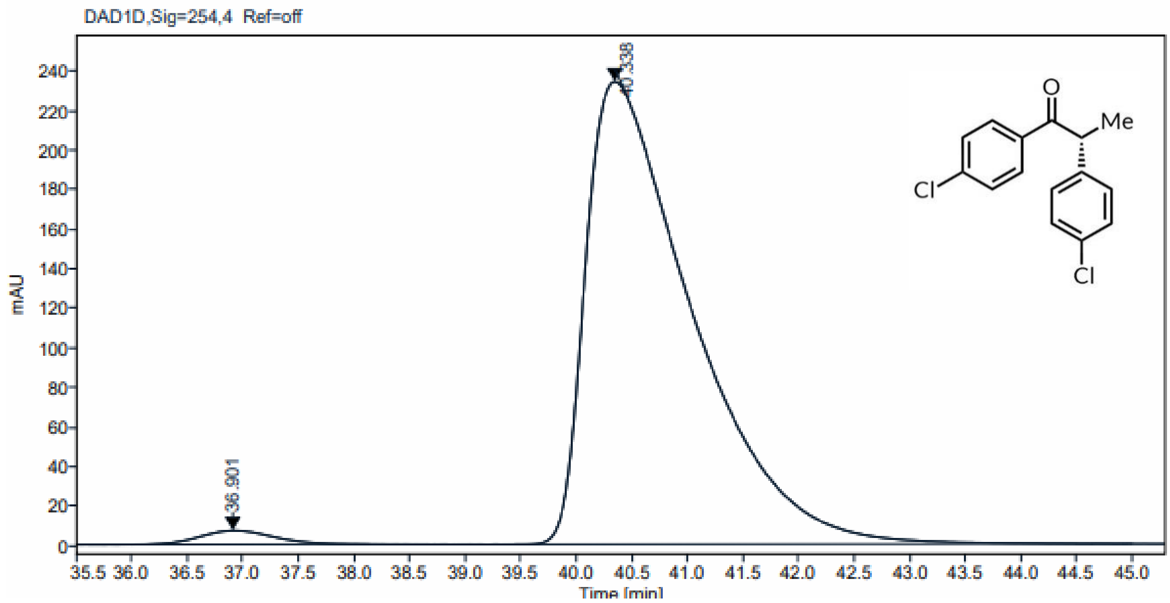

Signal:	DAD1D,Sig=254,4 Ref=off				
RT [min]	Type	Width [min]	Area	Height	Area% Name
36.901	MM m	2.5409	333.4515	7.0425	2.0938
40.338	MM m	5.5617	15592.4672	233.7770	97.9062

(*R*)-1,2-Bis(4-methoxyphenyl)propan-1-one ((***R*)-2i**)

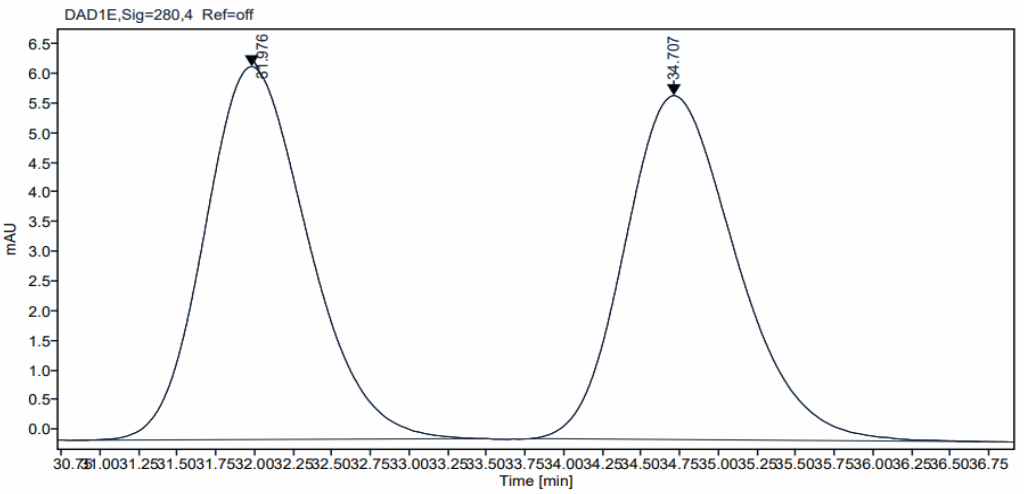

Signal: DAD1E,Sig=280,4 Ref=off

RT [min]	Type	Width [min]	Area	Height	Area%	Name
31.976	MM m	2.6494	292.0876	6.2827	50.1630	
34.707	MM m	2.9017	290.1897	5.7942	49.8370	

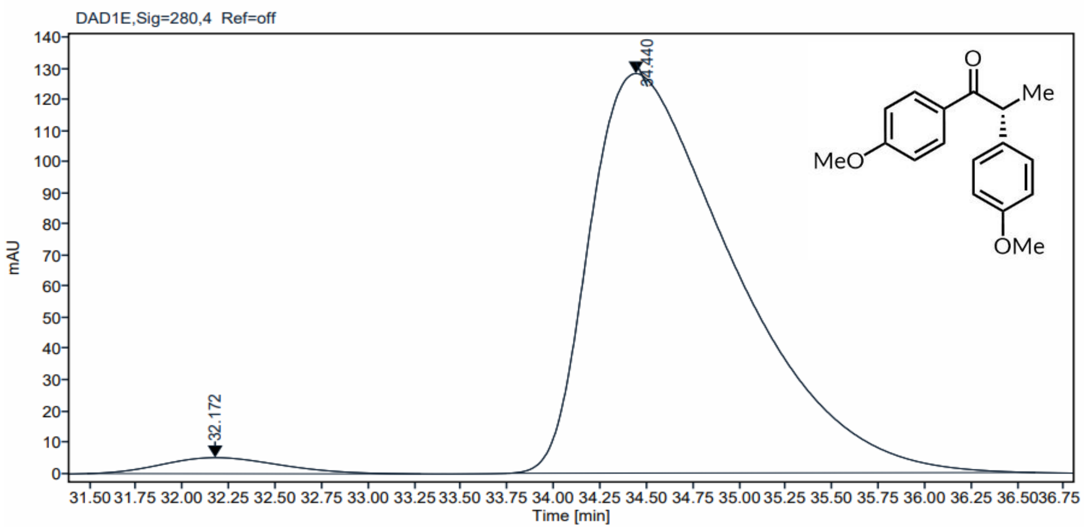

Signal: DAD1E,Sig=280,4 Ref=off

RT [min]	Type	Width [min]	Area	Height	Area%	Name
32.172	MM m	1.7978	227.4687	5.1590	3.1605	
34.440	MM m	2.8386	6969.7404	128.4273	96.8395	

(*R*)-1,2-Bis(4-phenoxyphenyl)propan-1-one (**(*R*)-2j**)

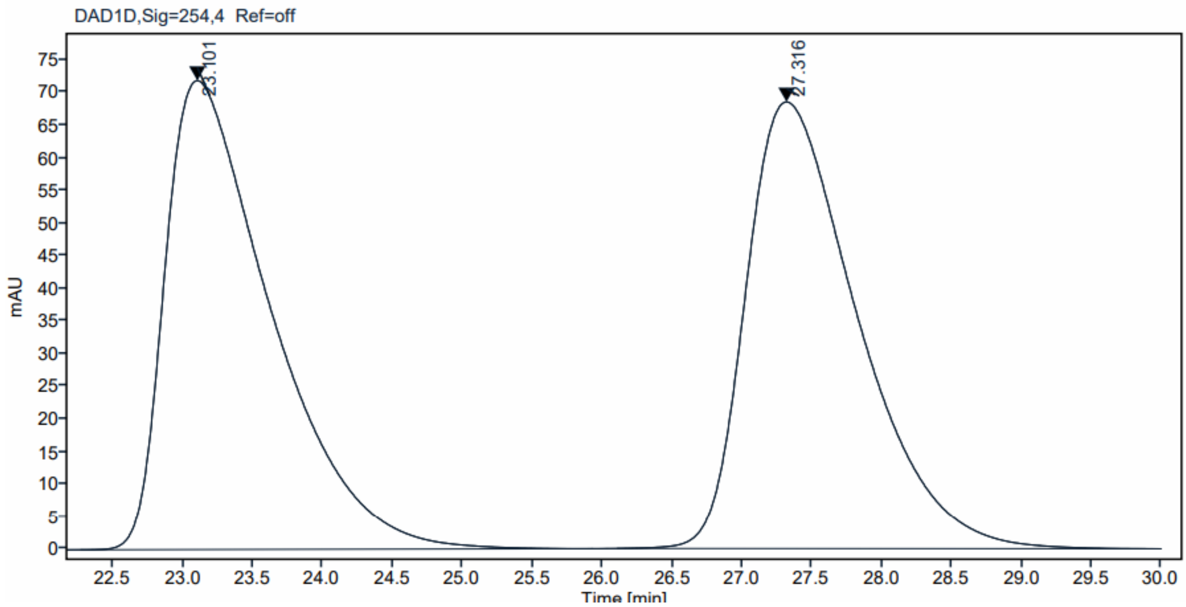

Signal:	DAD1D,Sig=254,4 Ref=off				
RT [min]	Type	Width [min]	Area	Height	Area% Name
23.101	MM m	3.6672	3797.7987	71.9188	49.9389
27.316	MM m	3.7813	3807.0859	68.5032	50.0611

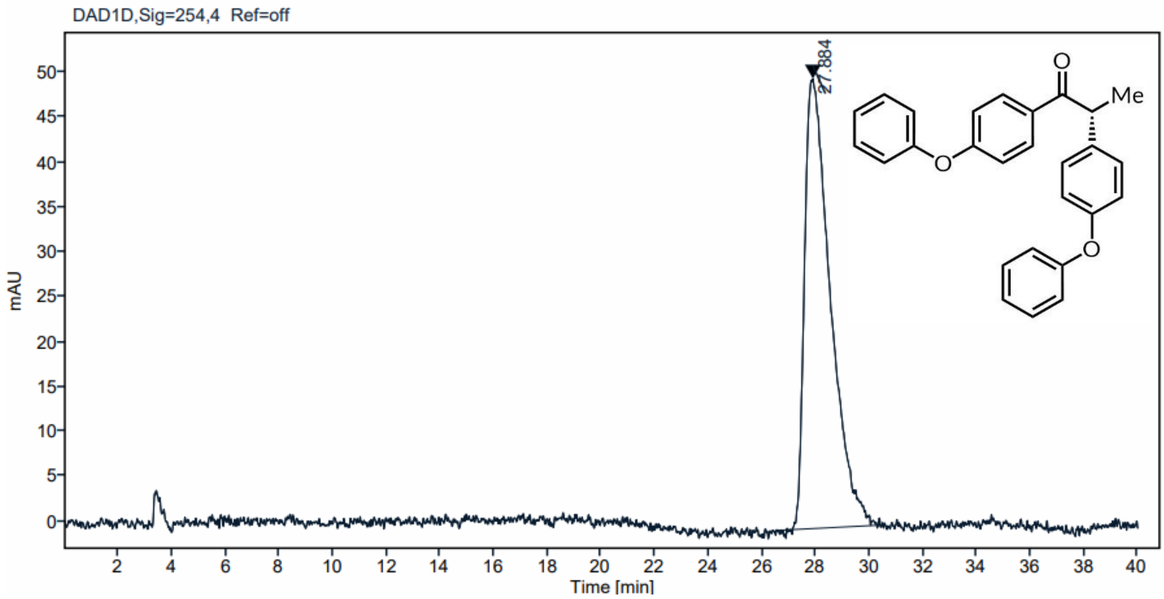

Signal:	DAD1D,Sig=254,4 Ref=off				
RT [min]	Type	Width [min]	Area	Height	Area% Name
27.884	MM m	3.9743	3337.2471	50.1050	100.0000

(*R*)-1,2-Bis(4-(methylthio)phenyl)propan-1-one (**(*R*)-2k**)

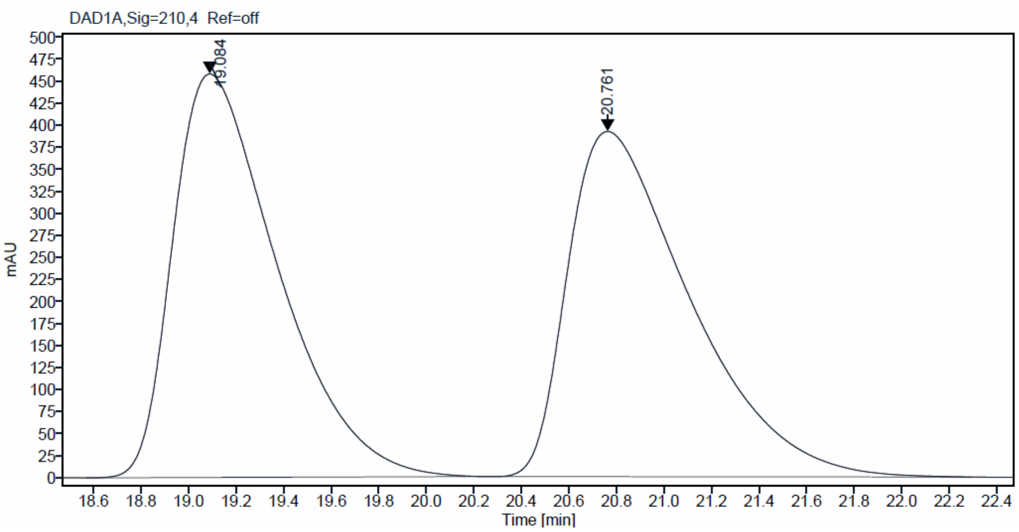

Signal: DAD1A,Sig=210,4 Ref=off

RT [min]	Type	Width [min]	Area	Height	Area%	Name
19.084	MM m	1.6566	14044.1007	458.1554	50.0301	
20.761	MM m	2.0708	14027.1968	391.8877	49.9699	

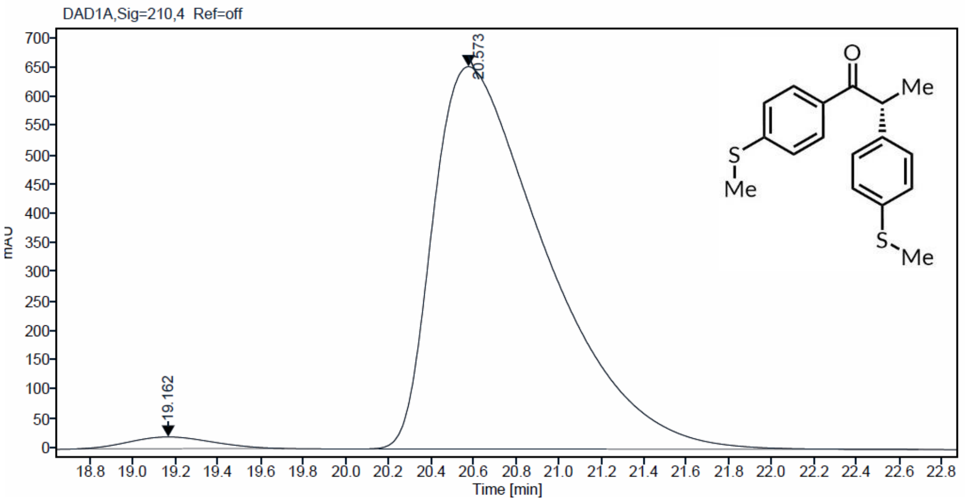

Signal: DAD1A,Sig=210,4 Ref=off

RT [min]	Type	Width [min]	Area	Height	Area%	Name
19.162	MM m	0.9698	541.5748	20.0036	2.1933	
20.573	MM m	2.5783	24150.8794	653.0561	97.8067	

(*R*)-1,2-Bis(4-((trimethylsilyl)ethynyl)phenyl)propan-1-one (**(*R*)-21**)

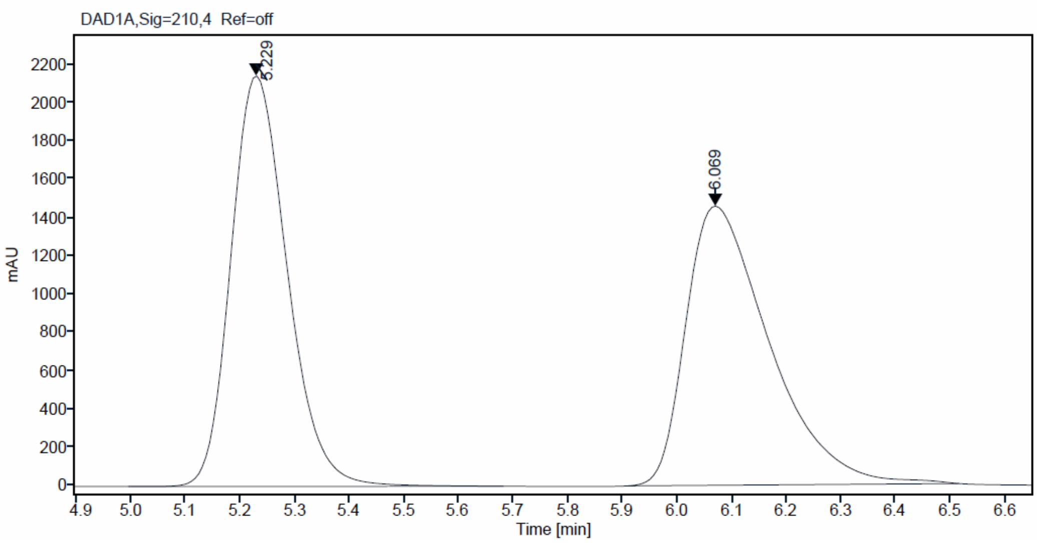

Signal: DAD1A,Sig=210,4 Ref=off

RT [min]	Type	Width [min]	Area	Height	Area%	Name
5.229	MM m	0.6872	15341.0174	2142.3200	49.8393	
6.069	MM m	0.6073	15439.9731	1456.6047	50.1607	

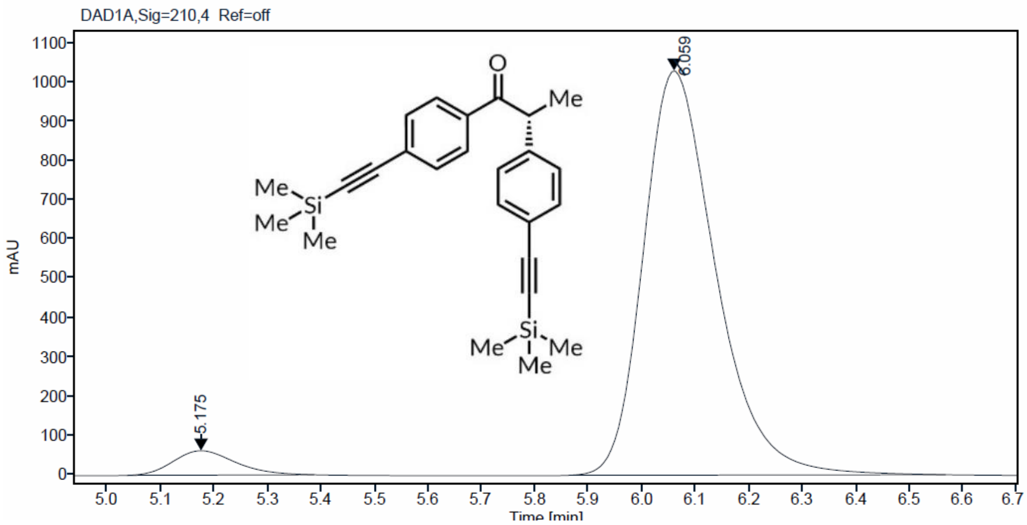

Signal: DAD1A,Sig=210,4 Ref=off

RT [min]	Type	Width [min]	Area	Height	Area%	Name
5.175	MM m	0.3497	500.7239	62.2223	4.9214	
6.059	MM m	0.7045	9673.7628	1030.7188	95.0786	

(*R*)-1,2-Bis(4-(trifluoromethyl)phenyl)propan-1-one ((***R*)-2m**)

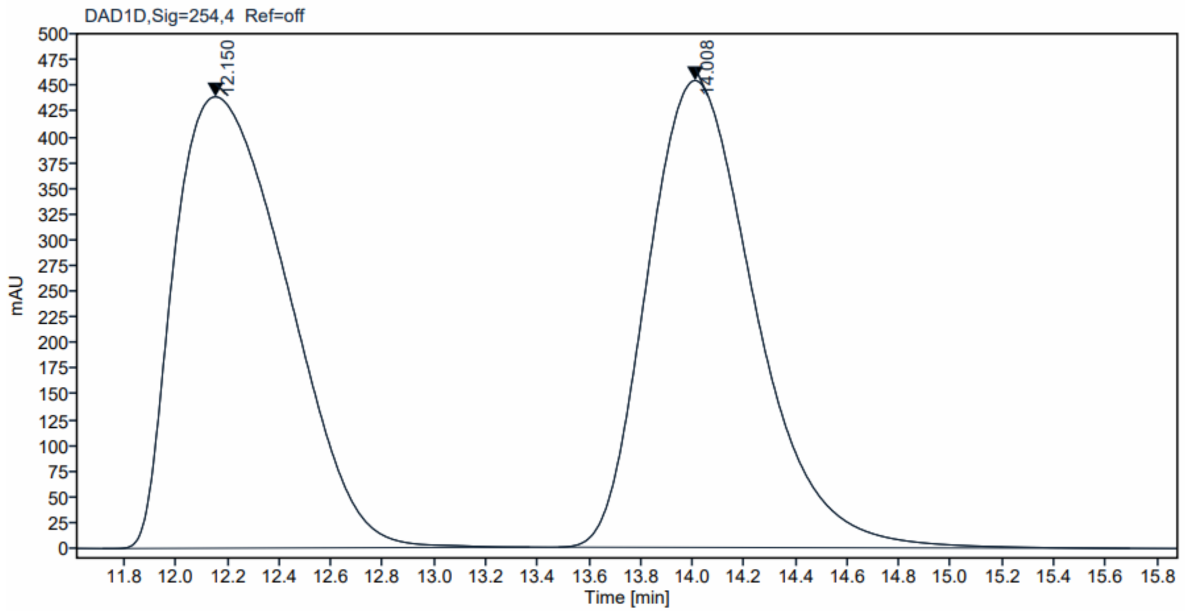

Signal:	DAD1D,Sig=254,4 Ref=off				
RT [min]	Type	Width [min]	Area	Height	Area% Name
12.150	MM m	1.6543	13299.0701	438.9135	50.2015
14.008	MM m	2.2127	13192.3362	453.9881	49.7985

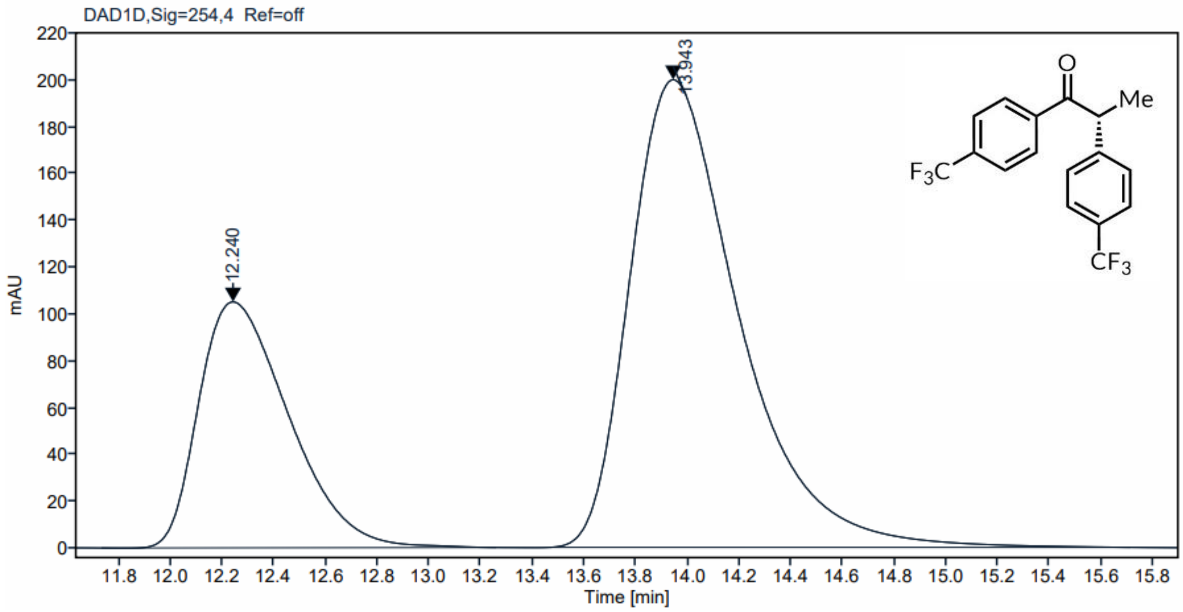

Signal:	DAD1D,Sig=254,4 Ref=off				
RT [min]	Type	Width [min]	Area	Height	Area% Name
12.240	MM m	1.5259	2536.6973	104.9488	30.2300
13.943	MM m	2.2457	5854.6313	199.5337	69.7700

(*R*)-1,2-Bis(3-trifluoromethylphenyl)propan-1-one (**(*R*)-2n**)

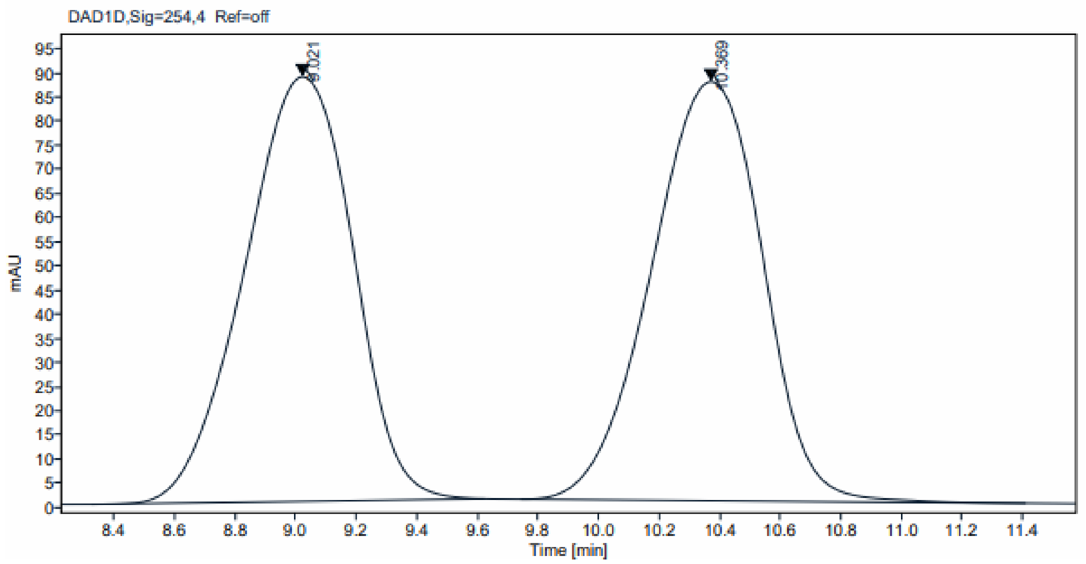

Signal:	DAD1D,Sig=254,4 Ref=off				
RT [min]	Type	Width [min]	Area	Height	Area% Name
9.021	MM m	1.2969	2186.8248	87.9157	49.6923
10.369	MM m	1.6674	2213.9035	86.7614	50.3077

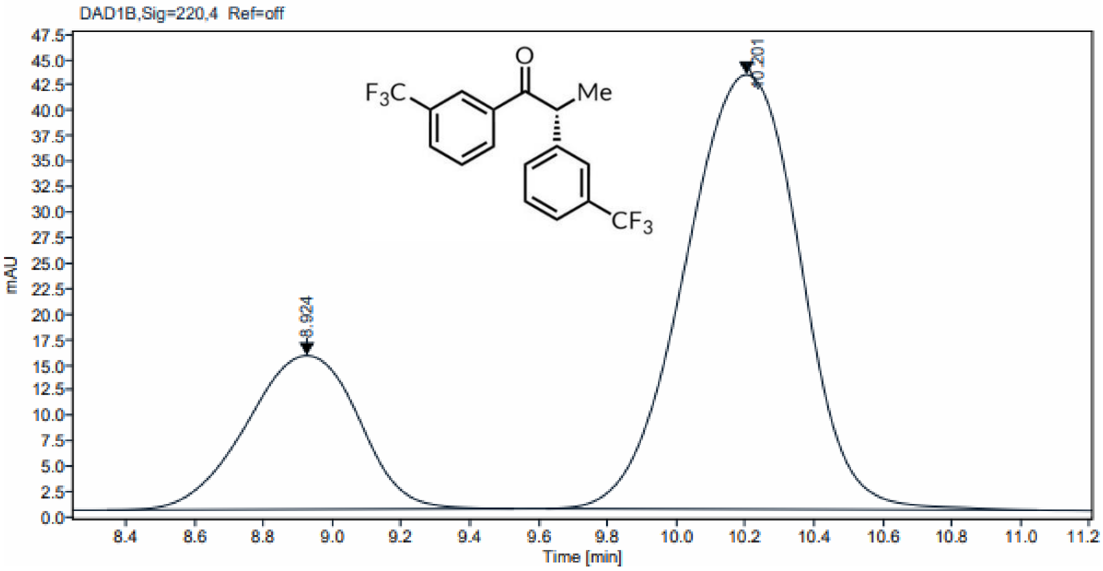

Signal:	DAD1B,Sig=220,4 Ref=off				
RT [min]	Type	Width [min]	Area	Height	Area% Name
8.924	MM m	1.1819	342.8440	15.0938	25.6521
10.201	MM m	1.4285	993.6710	42.7032	74.3479

(R)-1,2-Bis(3-chlorophenyl)propan-1-one (**(R)-2o**)

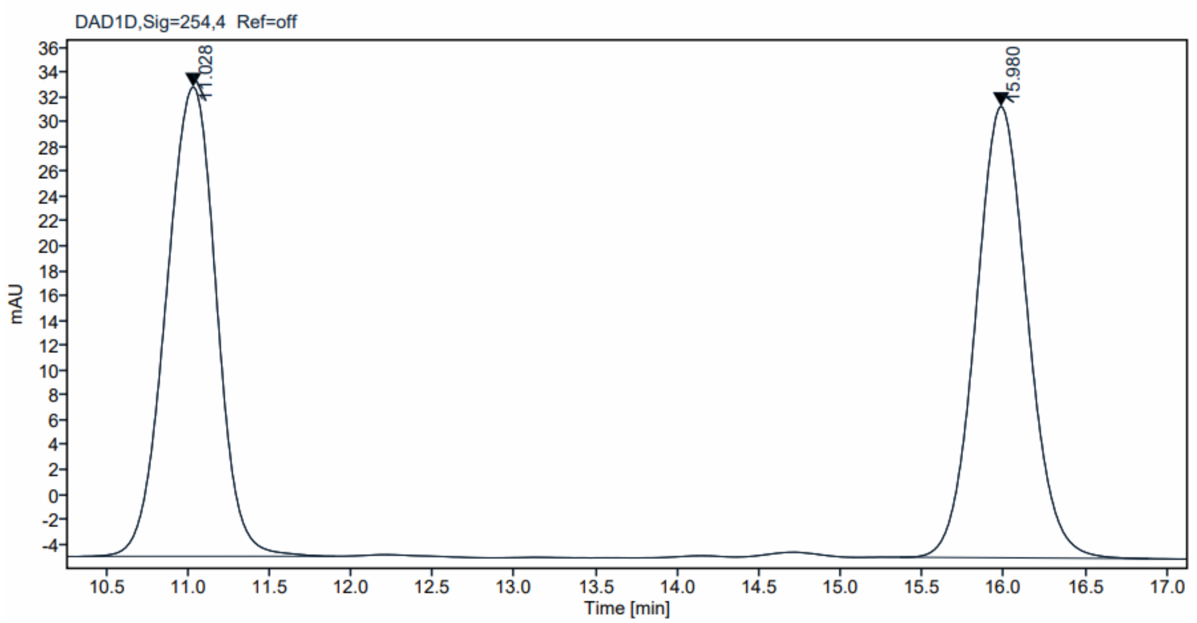

Signal: DAD1D,Sig=254,4 Ref=off

RT [min]	Type	Width [min]	Area	Height	Area%	Name
11.028	MM m	1.5433	806.6643	37.7616	50.2008	
15.980	MM m	1.5244	800.2119	36.3127	49.7992	

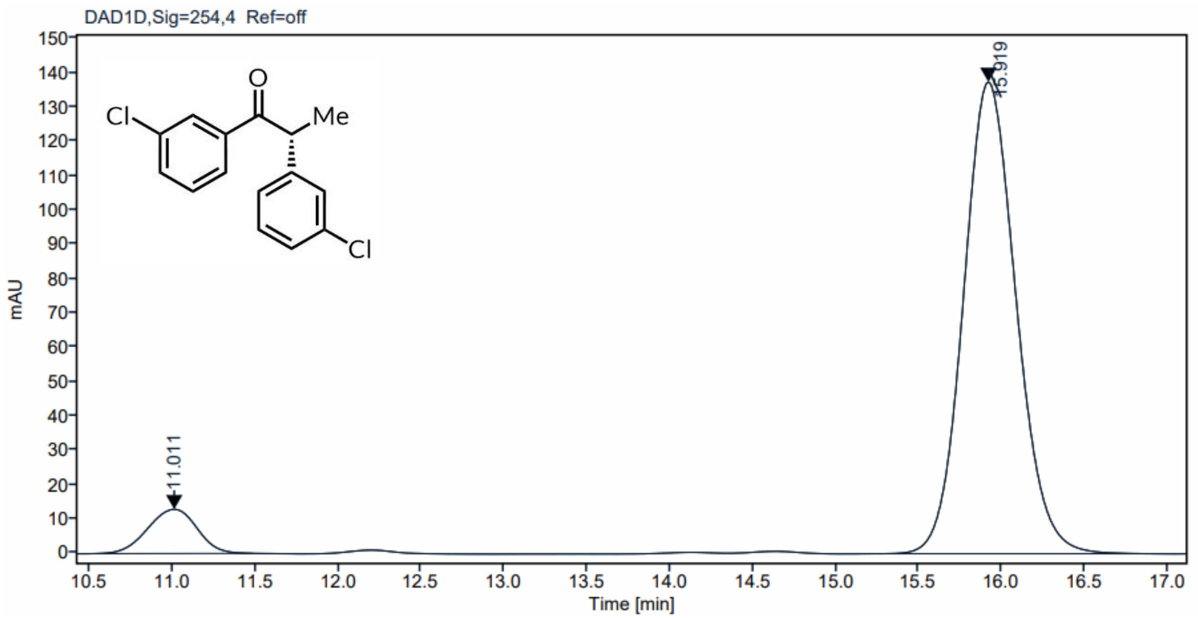

Signal: DAD1D,Sig=254,4 Ref=off

RT [min]	Type	Width [min]	Area	Height	Area%	Name
11.011	MM m	0.9598	266.7085	12.8733	8.0819	
15.919	MM m	1.6374	3033.3716	137.4777	91.9181	

(*R*)-1,2-Bis(3-fluorophenyl)propan-1-one ((***R*)-2p**)

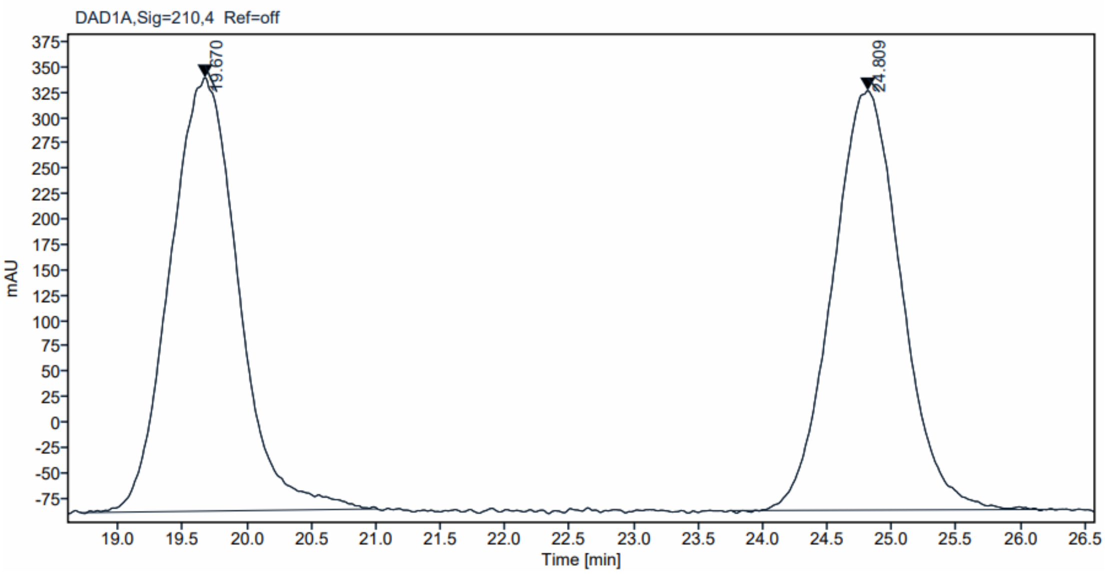

Signal: DAD1A,Sig=210,4 Ref=off

RT [min]	Type	Width [min]	Area	Height	Area%	Name
19.670	MM m	2.3000	15778.7916	427.4497	50.6293	
24.809	MM m	2.5581	15386.5193	413.6299	49.3707	

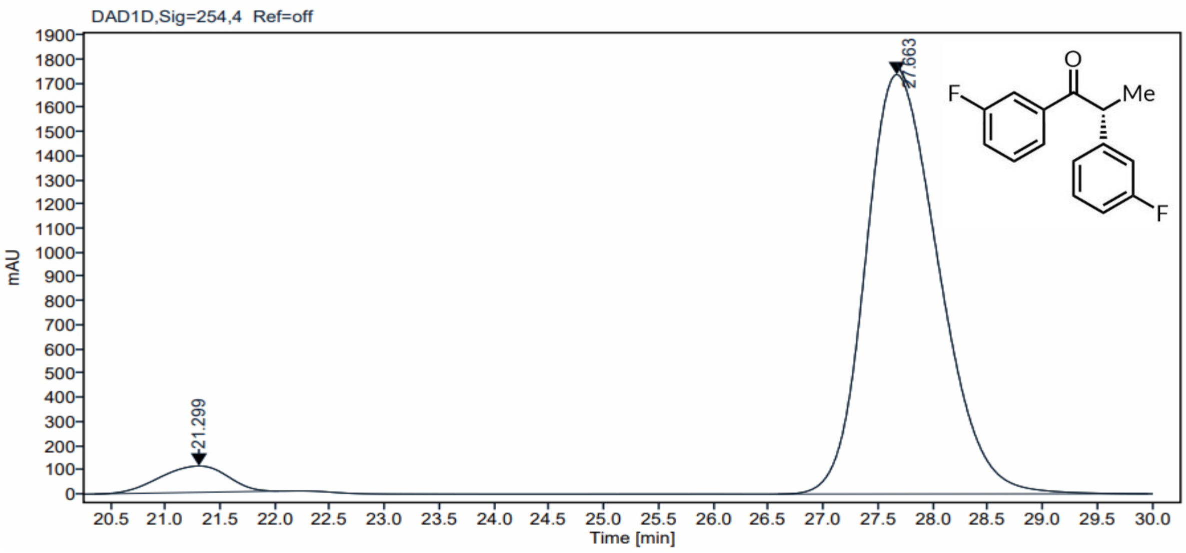

Signal: DAD1D,Sig=254,4 Ref=off

RT [min]	Type	Width [min]	Area	Height	Area%	Name
21.299	MM m	1.6782	4679.8508	109.1246	5.4895	
27.663	MM m	3.3736	80571.7069	1734.5993	94.5105	

(*R*)-1,2-*m*-tolylpropan-1-one ((***R*)-2q**)

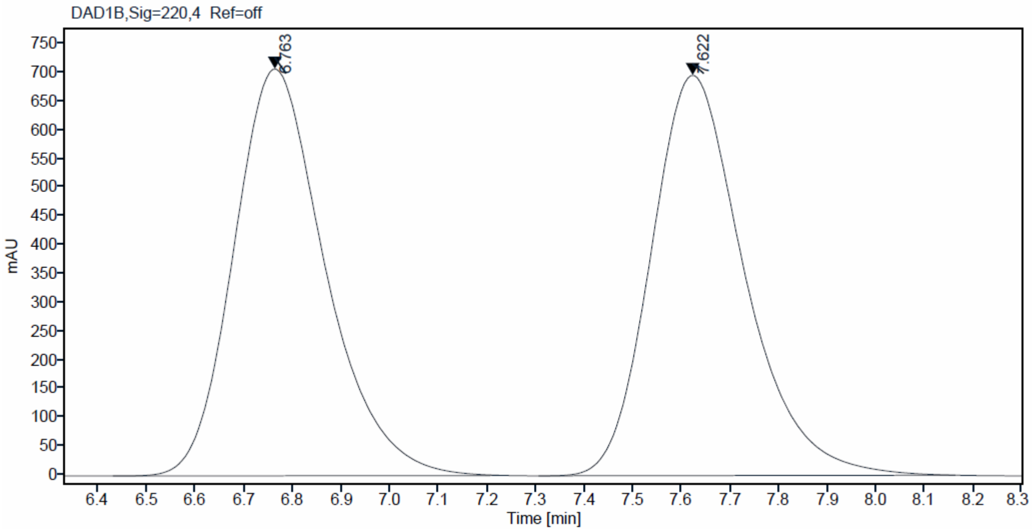

Signal: DAD1B,Sig=220,4 Ref=off

RT [min]	Type	Width [min]	Area	Height	Area%	Name
6.763	MM m	0.8118	9245.5999	706.5851	49.8686	
7.622	MM m	0.8567	9294.3049	695.3428	50.1314	

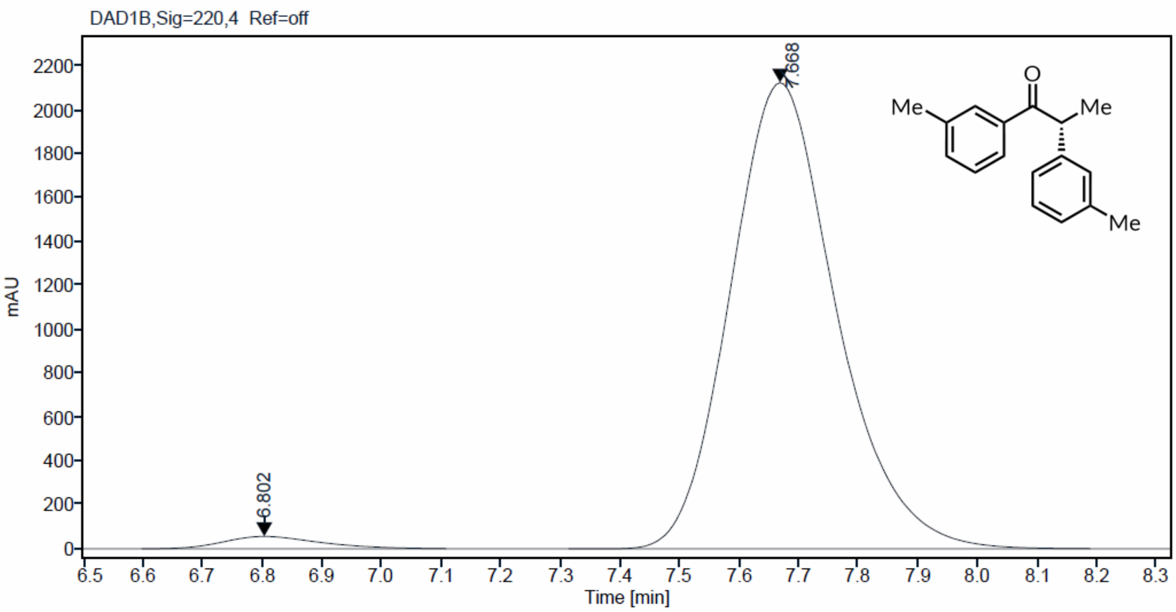

Signal: DAD1B,Sig=220,4 Ref=off

RT [min]	Type	Width [min]	Area	Height	Area%	Name
6.802	MM m	0.5108	628.7047	56.0710	2.3360	
7.668	MM m	0.8752	26285.5793	2127.2219	97.6640	

(*R*)-1,2-bis(3-methoxyphenyl)propan-1-one (**(*R*)-2r**)

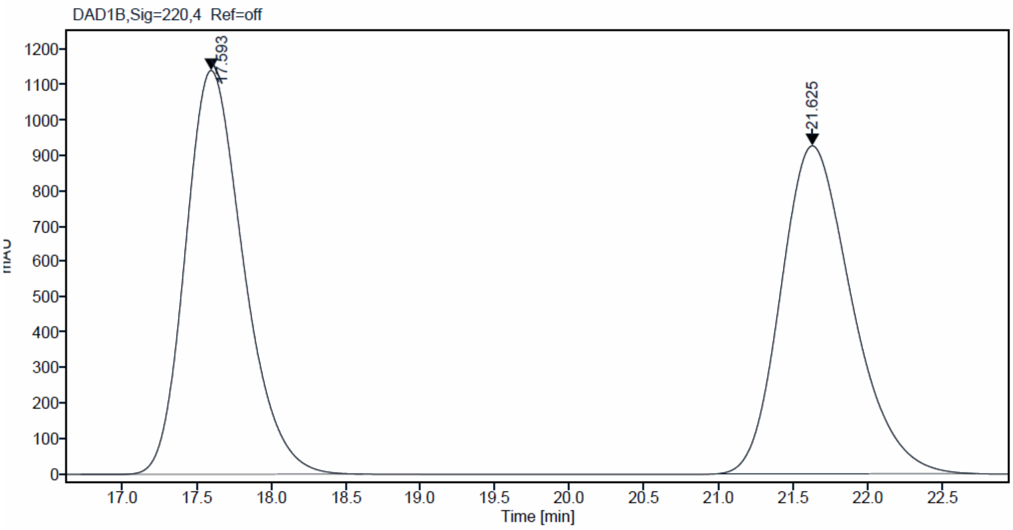

Signal: DAD1B,Sig=220,4 Ref=off

RT [min]	Type	Width [min]	Area	Height	Area%	Name
17.593	MM m	1.8909	30852.2779	1141.9856	50.1188	
21.625	MM m	1.7387	30706.0164	927.4450	49.8812	

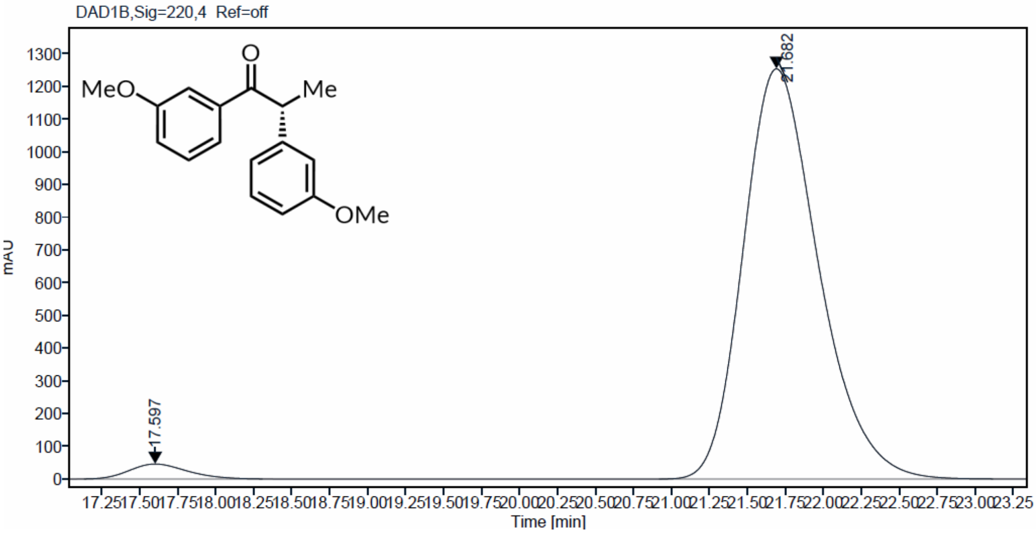

Signal: DAD1B,Sig=220,4 Ref=off

RT [min]	Type	Width [min]	Area	Height	Area%	Name
17.597	MM m	1.1737	1207.8305	45.5209	2.7240	
21.682	MM m	2.1952	43131.9569	1253.4308	97.2760	

(±)-1,2-Bis(2-chlorophenyl)propan-1-one (**(±)-2s**)

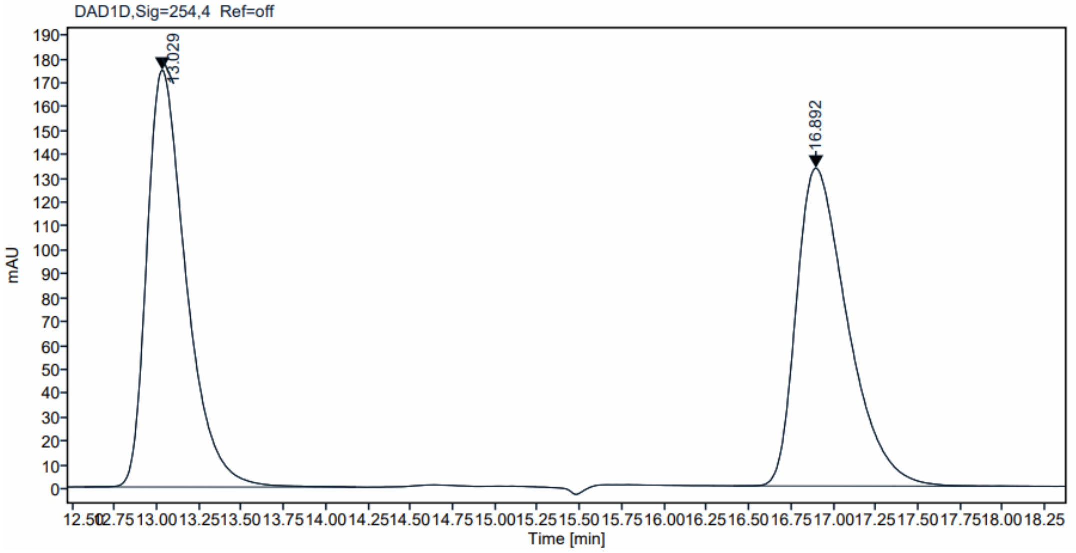

Signal:	DAD1D,Sig=254,4 Ref=off				
RT [min]	Type	Width [min]	Area	Height	Area% Name
13.029	MM m	1.6039	2818.0253	174.6497	50.1070
16.892	MM m	1.7490	2805.9879	133.1647	49.8930

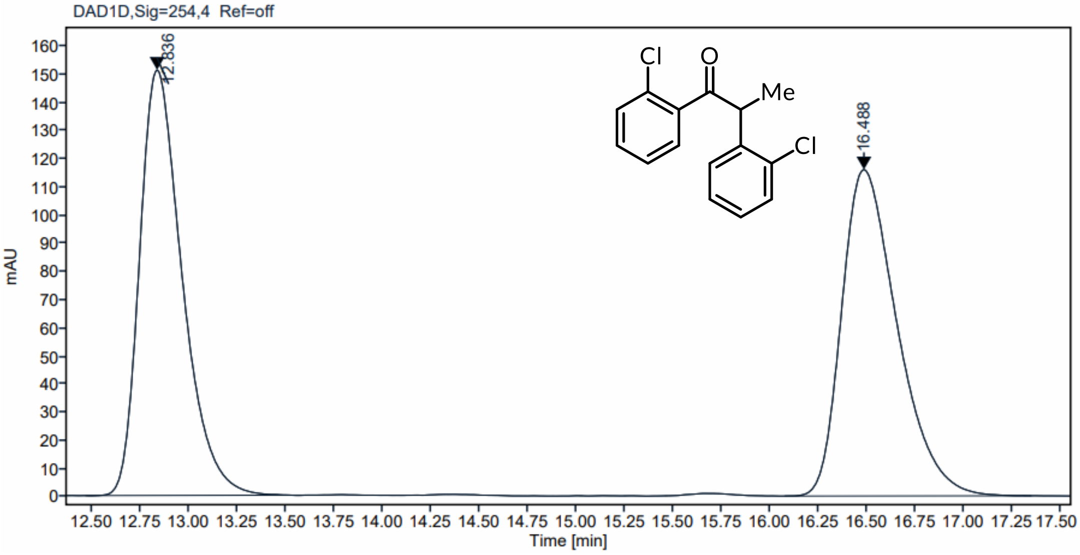

Signal:	DAD1D,Sig=254,4 Ref=off				
RT [min]	Type	Width [min]	Area	Height	Area% Name
12.836	MM m	1.1388	2308.8099	150.9210	49.9476
16.488	MM m	1.2759	2313.6565	115.8239	50.0524

(±)-1,2-Bis(2-methylphenyl)propan-1-one (**(±)-2t**)

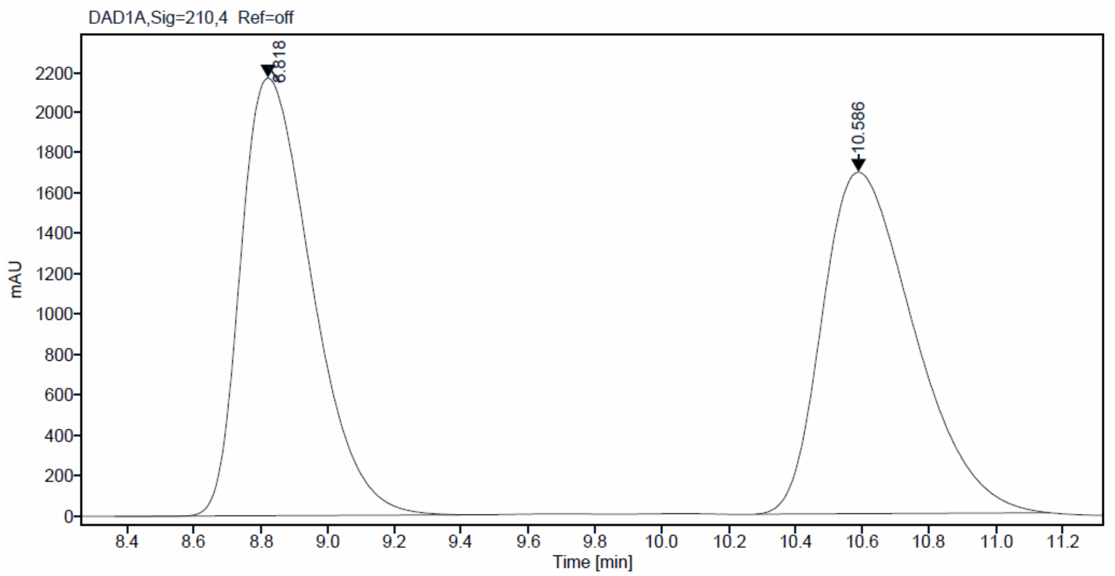

Signal: DAD1A,Sig=210,4 Ref=off

RT [min]	Type	Width [min]	Area	Height	Area%	Name
8.818	MM m	1.1067	32101.0241	2172.9181	49.7433	
10.586	MM m	0.8808	32432.3988	1695.3004	50.2567	

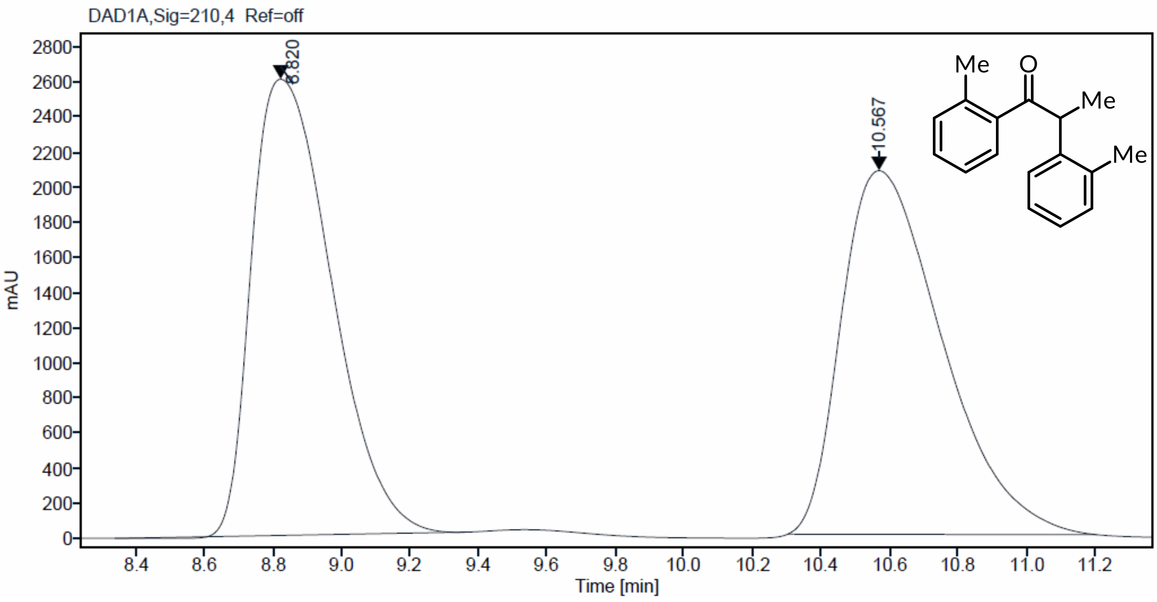

Signal: DAD1A,Sig=210,4 Ref=off

RT [min]	Type	Width [min]	Area	Height	Area%	Name
8.820	MM m	1.0389	42054.0860	2600.5057	49.7717	
10.567	MM m	0.9034	42439.9409	2073.2927	50.2283	

(*R*)-1,2-Bis(2-methoxyphenyl)propan-1-one ((***R*)-2u**)

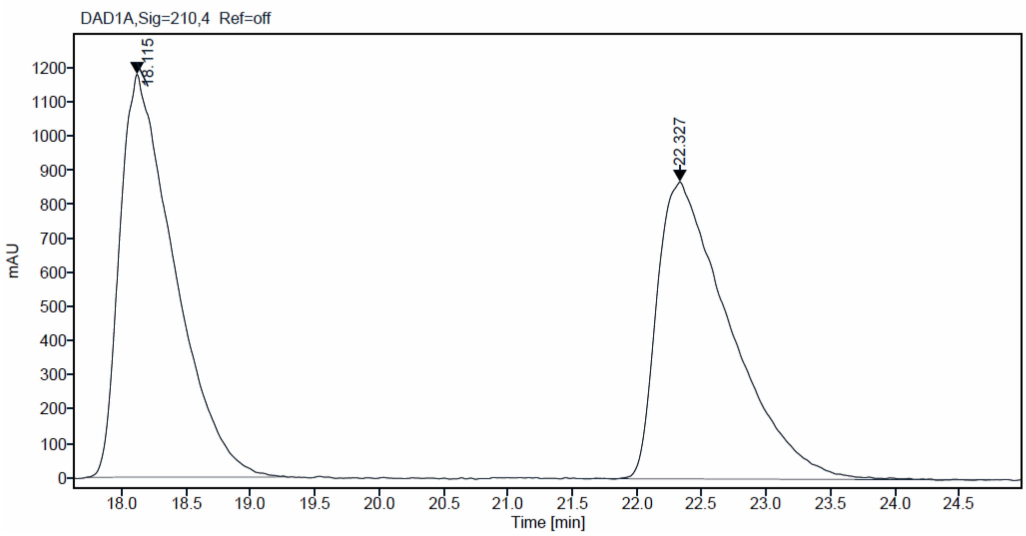

Signal: DAD1A,Sig=210,4 Ref=off

RT [min]	Type	Width [min]	Area	Height	Area%	Name
18.115	MM m	1.5539	34703.5994	1177.7916	50.3658	
22.327	MM m	3.1800	34199.5499	868.2411	49.6342	

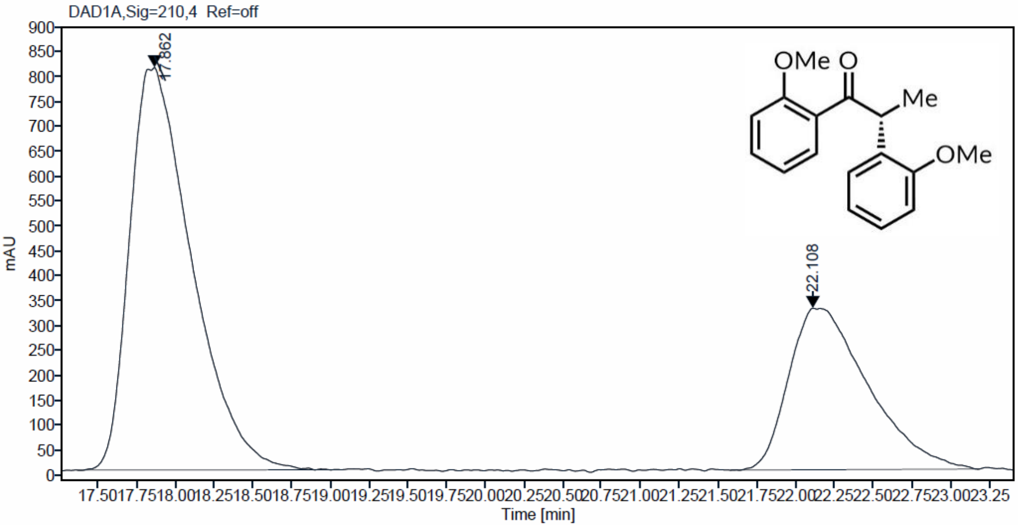

Signal: DAD1A,Sig=210,4 Ref=off

RT [min]	Type	Width [min]	Area	Height	Area%	Name
17.862	MM m	1.7027	22527.2068	807.3686	66.1759	
22.108	MM m	1.6081	11514.1921	324.0117	33.8241	

(*R*)-1,2-Bis(2-fluorophenyl)propan-1-one ((***R*)-2v**)

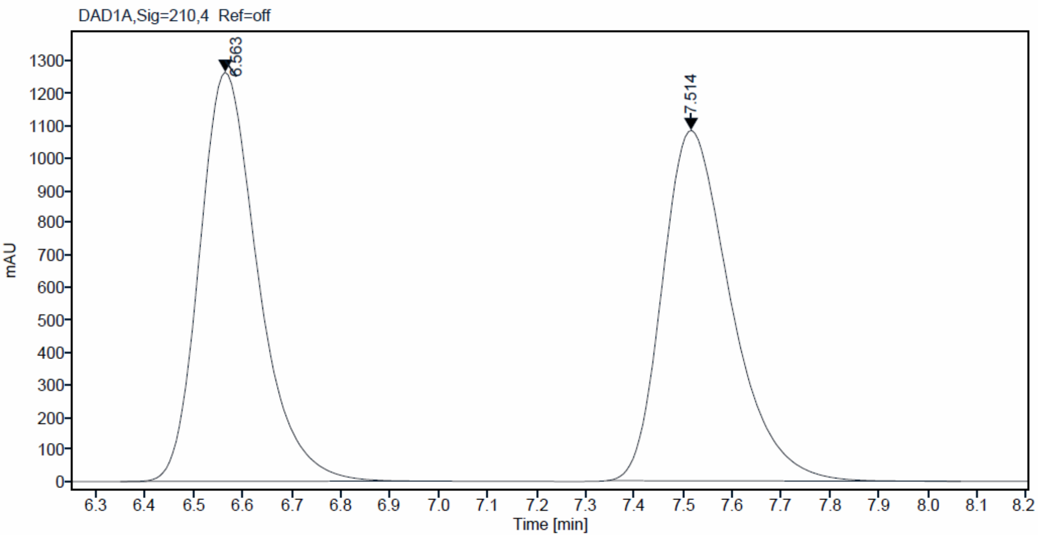

Signal:	DAD1A,Sig=210,4 Ref=off				
**RT [min]**	**Type**	**Width [min]**	**Area**	**Height**	**Area% Name**
6.563	MM m	0.6324	10489.0435	1263.6941	49.9737
7.514	MM m	0.7227	10500.0878	1082.4328	50.0263

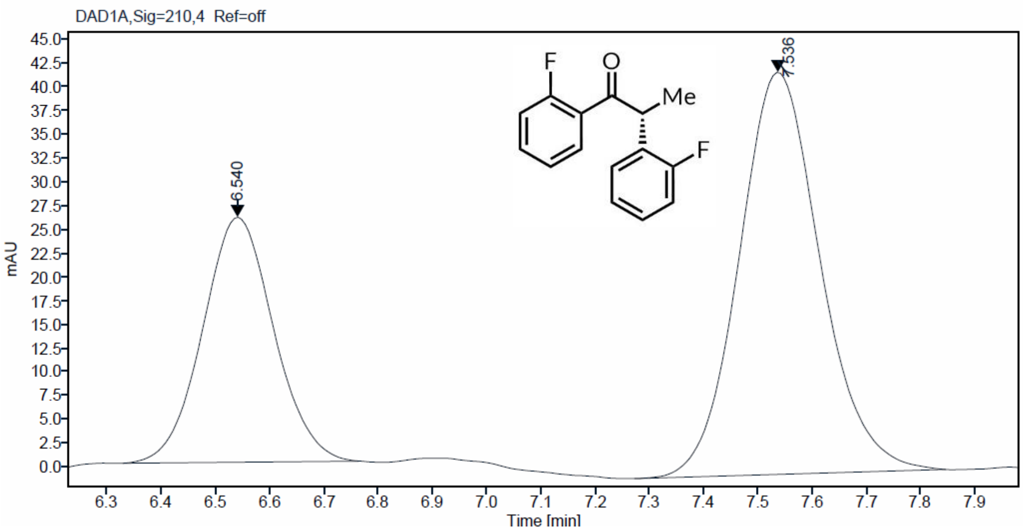

Signal:	DAD1A,Sig=210,4 Ref=off				
**RT [min]**	**Type**	**Width [min]**	**Area**	**Height**	**Area% Name**
6.540	MM m	0.4393	234.1796	25.7811	34.7913
7.536	MM m	0.5740	438.9181	42.3112	65.2087

(*R*)-1,2-Di(benzofuran-2-yl)propan-1-one ((***R*)-2w**)

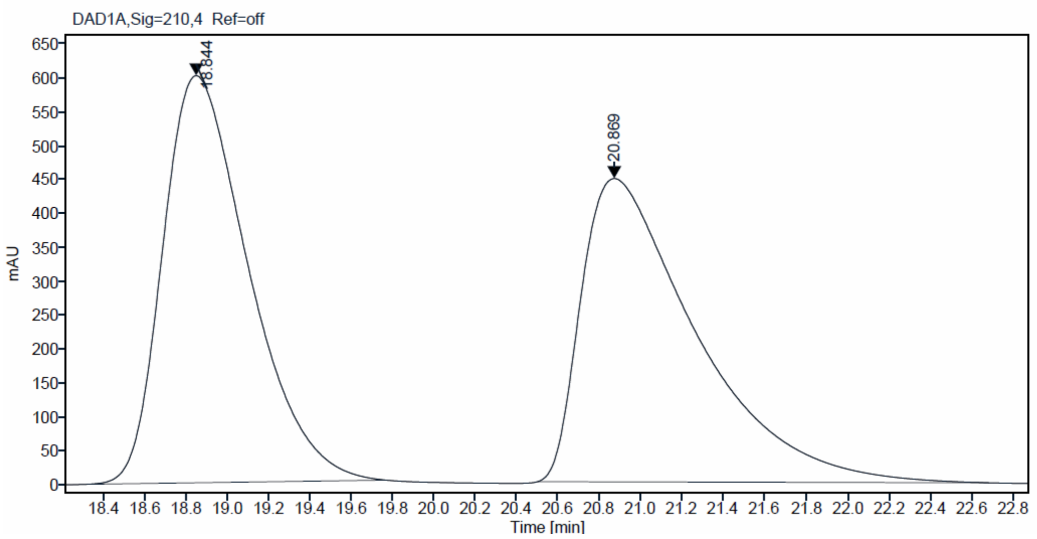

Signal: DAD1A,Sig=210,4 Ref=off

RT [min]	Type	Width [min]	Area	Height	Area%	Name
18.844	MM m	1.4500	17296.5677	599.8774	50.0432	
20.869	MM m	2.1847	17266.6965	446.9490	49.9568	

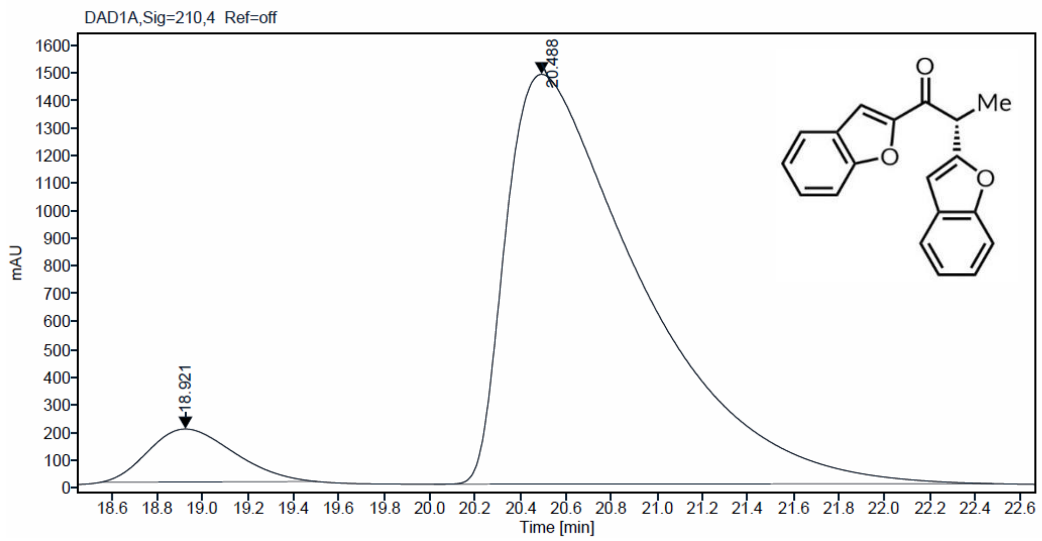

Signal: DAD1A,Sig=210,4 Ref=off

RT [min]	Type	Width [min]	Area	Height	Area%	Name
18.921	MM m	0.9507	5009.4705	192.0131	7.5207	
20.488	MM m	2.4643	61599.8615	1485.3760	92.4793	

(*S*)-1,2-Bis(benzothiophen-2-yl)propan-1-one (**(*R*)-2x**)

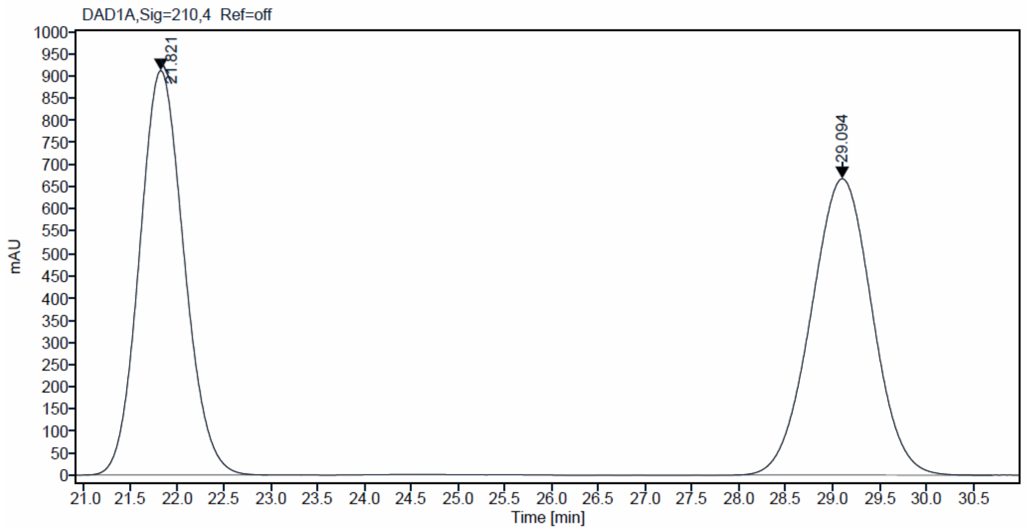

Signal: DAD1A,Sig=210,4 Ref=off

RT [min]	Type	Width [min]	Area	Height	Area%	Name
21.821	MM m	1.9552	30233.7313	914.7307	49.9412	
29.094	MM m	2.7436	30304.9281	670.1659	50.0588	

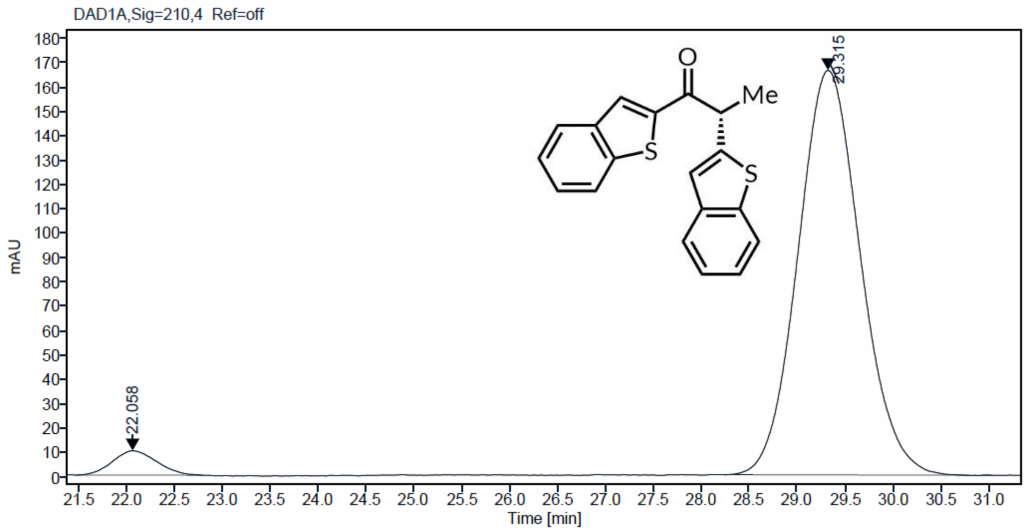

Signal: DAD1A,Sig=210,4 Ref=off

RT [min]	Type	Width [min]	Area	Height	Area%	Name
22.058	MM m	1.3251	320.6722	9.9989	4.0952	
29.315	MM m	2.8386	7509.8603	165.8807	95.9048	

(*S*)-1,2-Di(thiophen-2-yl)propan-1-one (**(*R*)-2y**)

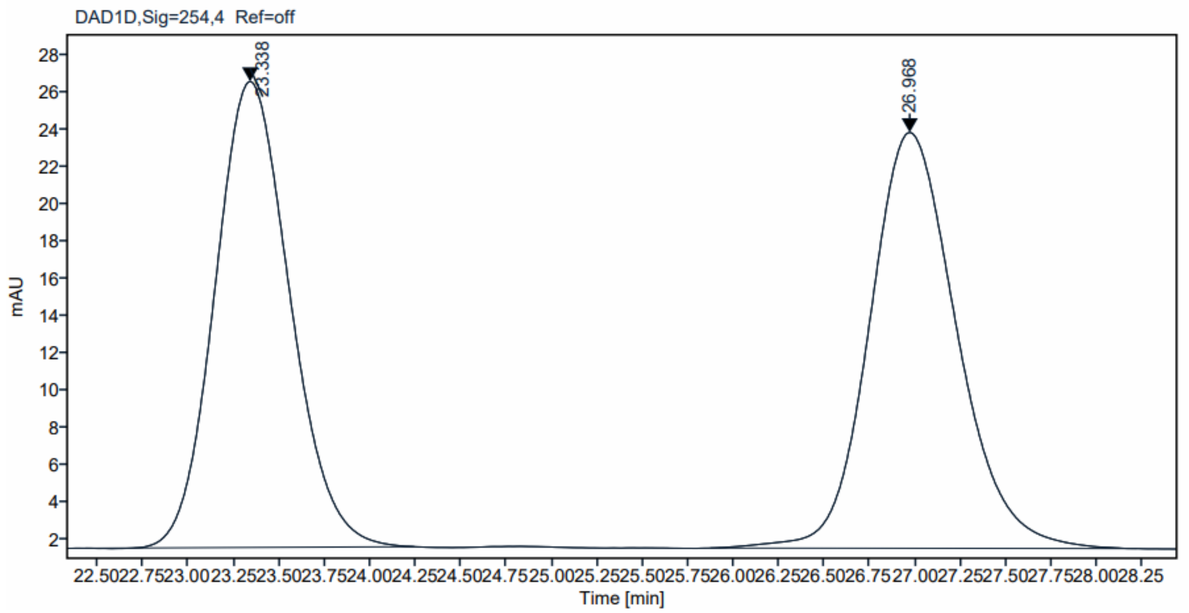

Signal:	DAD1D,Sig=254,4 Ref=off				
RT [min] Type	Width [min]	Area	Height	Area%	Name
23.338 MM m	1.8093	718.2718	25.0327	49.0564	
26.968 MM m	2.3444	745.9048	22.3384	50.9436	

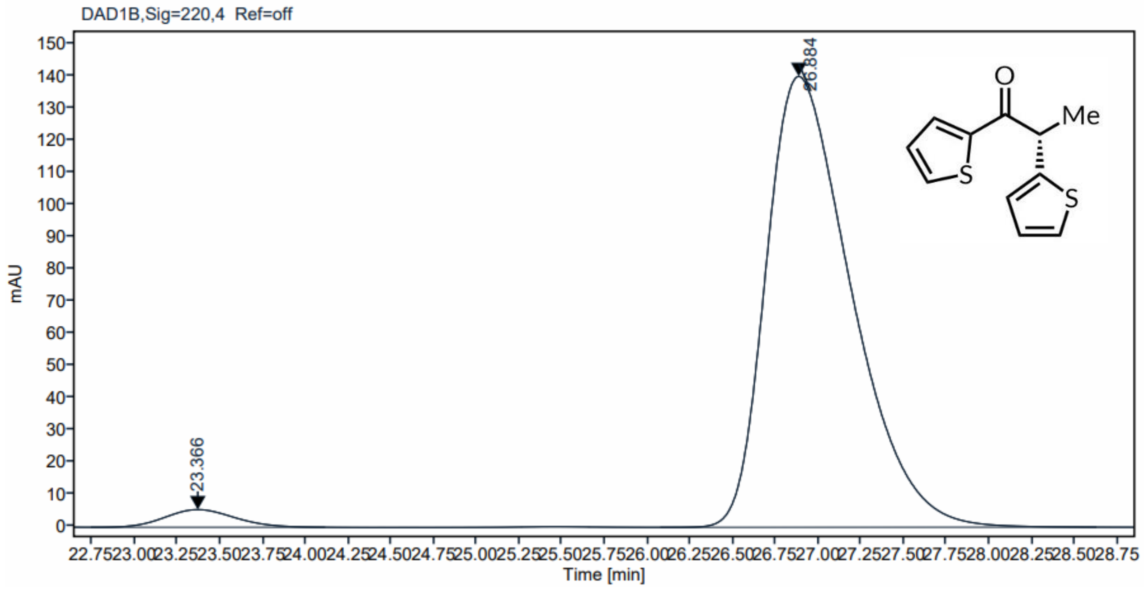

Signal:	DAD1B,Sig=220,4 Ref=off				
RT [min] Type	Width [min]	Area	Height	Area%	Name
23.366 MM m	1.3709	156.2994	5.4786	3.1167	
26.884 MM m	2.5526	4858.6684	140.2506	96.8833	

(*R*)-1,2-Bis(6-methoxynaphthalen-2-yl)propan-1-one (**(*R*)-2z**)

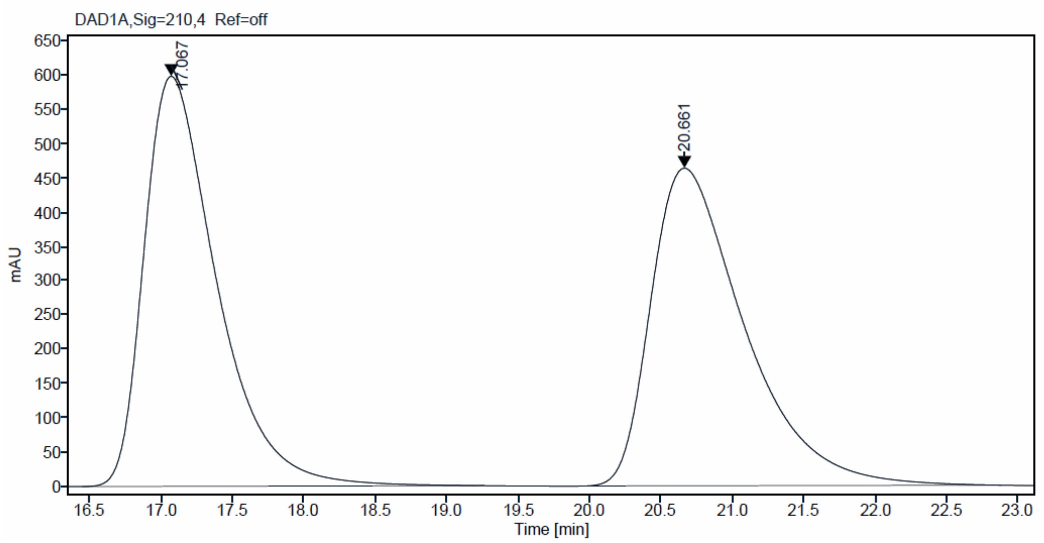

Signal: DAD1A,Sig=210,4 Ref=off

RT [min]	Type	Width [min]	Area	Height	Area%	Name
17.067	MM m	2.8148	21201.8970	599.9019	50.0142	
20.661	MM m	2.9094	21189.8481	464.6244	49.9858	

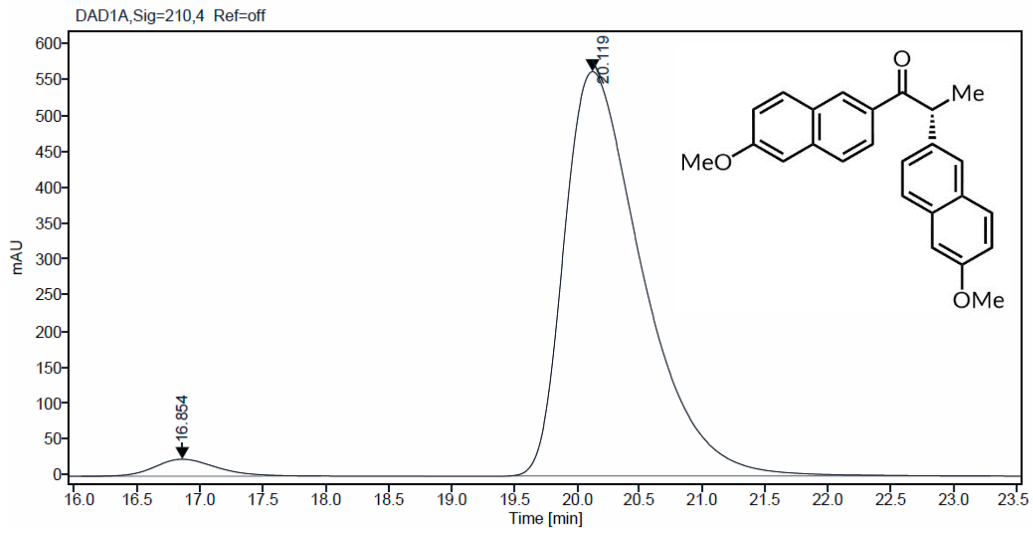

Signal: DAD1A,Sig=210,4 Ref=off

RT [min]	Type	Width [min]	Area	Height	Area%	Name
16.854	MM m	1.9056	809.4204	23.5688	3.2225	
20.119	MM m	3.8381	24308.5196	562.1626	96.7775	

(±)-2-Methyl-1,2-diphenylbutan-1-one (**2aa**)

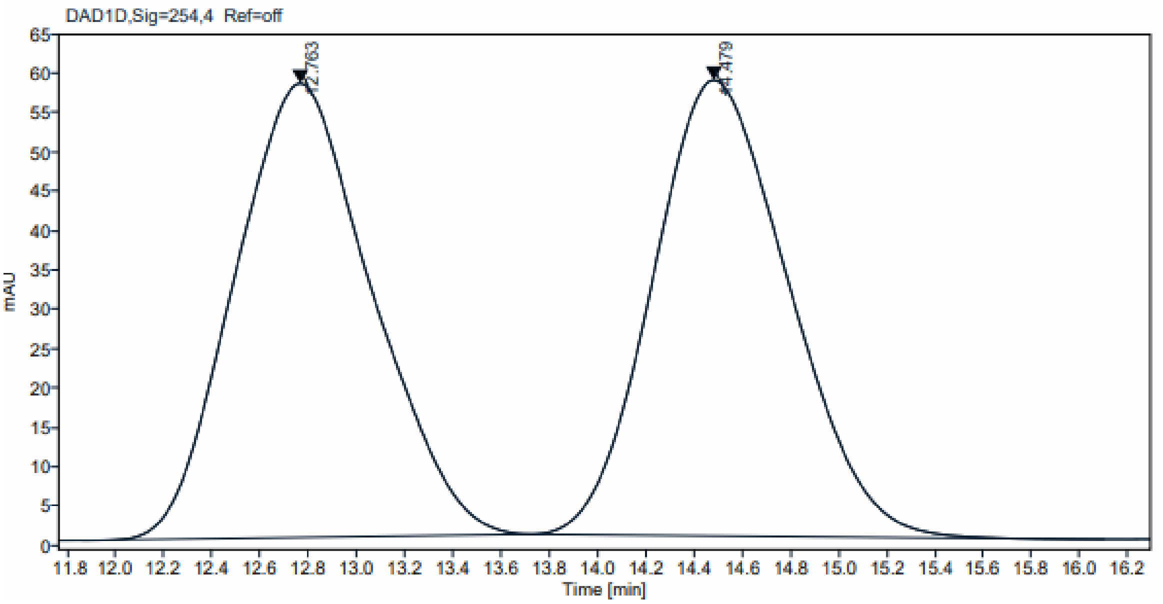

Signal:	DAD1D,Sig=254,4 Ref=off				
RT [min]	Type	Width [min]	Area	Height	Area% Name
12.763	MM m	1.8491	2275.7051	57.6362	50.0536
14.479	MM m	2.3924	2270.8321	57.8516	49.9464

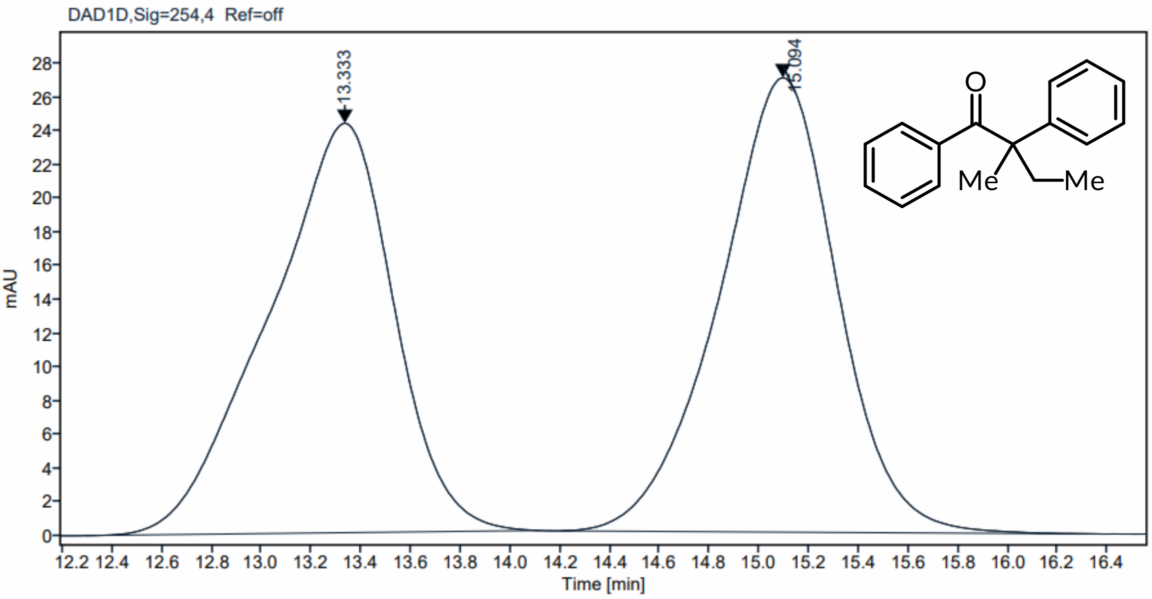

Signal:	DAD1D,Sig=254,4 Ref=off				
RT [min]	Type	Width [min]	Area	Height	Area% Name
13.333	MM m	1.8242	852.8522	24.2763	48.8646
15.094	MM m	2.1681	892.4858	26.9493	51.1354

(*R*)-1,2-Diphenylpropan-1-one-3-*d* (**(*R*)-2b-D**$_1$)

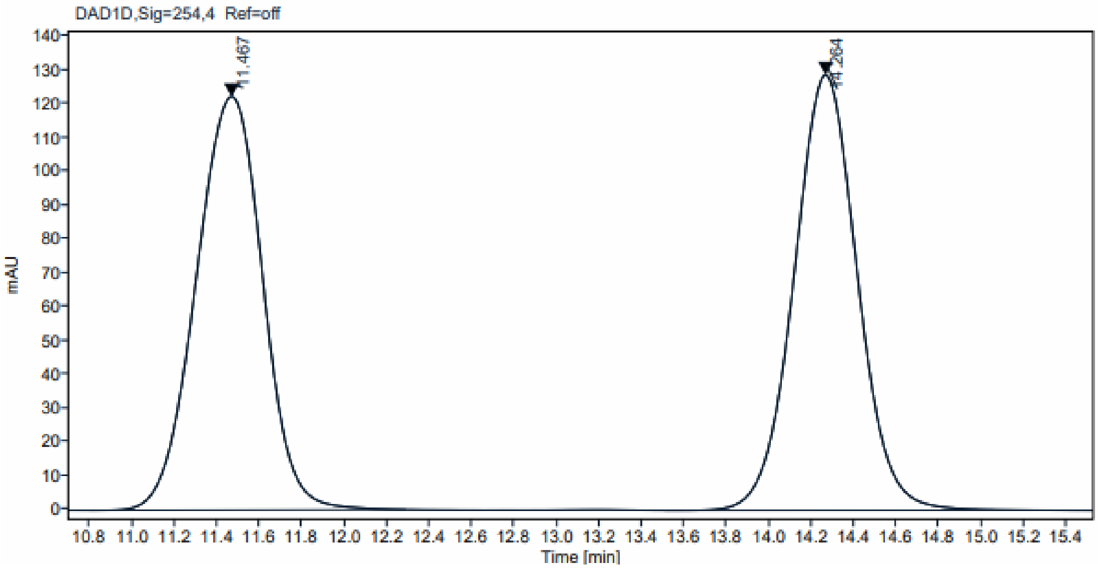

Signal: DAD1D,Sig=254,4 Ref=off

RT [min]	Type	Width [min]	Area	Height	Area%	Name
11.467	MM m	1.4912	2672.0108	122.3091	49.9522	
14.264	MM m	1.8247	2677.1280	128.8082	50.0478	
		Sum	5349.1388			

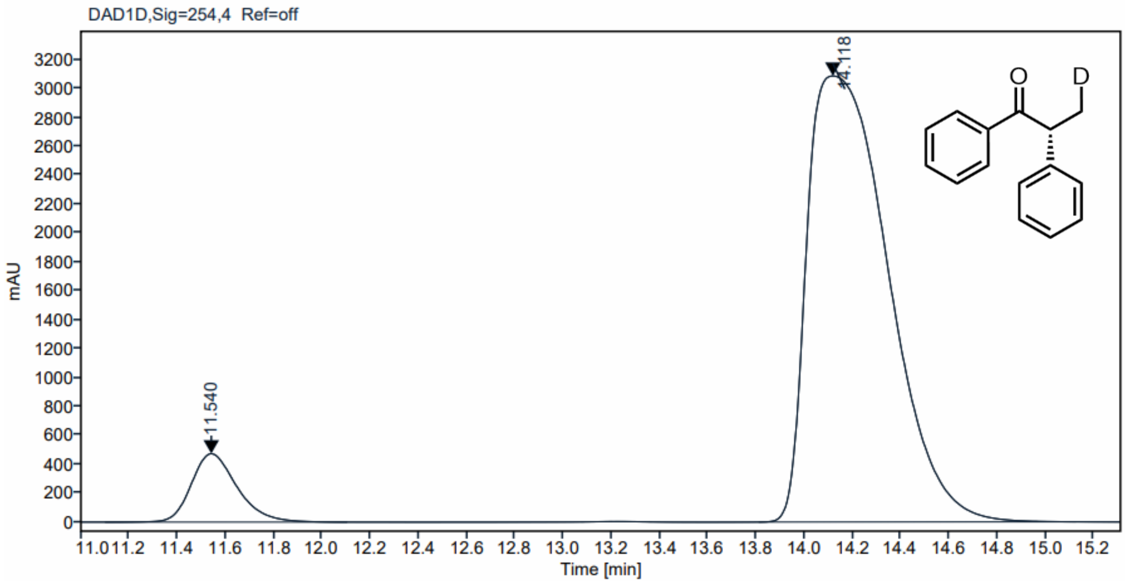

Signal: DAD1D,Sig=254,4 Ref=off

RT [min]	Type	Width [min]	Area	Height	Area%	Name
11.540	MM m	1.0045	6251.4847	473.1243	7.9949	
14.118	MM m	1.3159	71942.2120	3085.6857	92.0051	

References

1 H. Li, R.-Y. Zhu, W.-J. Shi, K.-H. He and Z.-J. Shi, *Org. Lett.*, 2012, **14**, 4850–4853.
2 C. Wang, X. Huang, X. Liu, S. Gao, B. Zhao and S. Yang, *Chin. Chem. Lett.*, 2020, **31**, 677–680.
3 P. Lucas, N. E. Mehdi, H. A. Ho, D. Bélanger and L. Breau, *Synthesis*, 2000, **2000**, 1253–1258.
4 Z. Zhang, C. Li, S.-H. Wang, F.-M. Zhang, X. Han, Y.-Q. Tu and X.-M. Zhang, *Org. Biomol. Chem.*, 2017, **15**, 3239–3247.
5 E. E. Touney, N. J. Foy and S. V. Pronin, *J. Am. Chem. Soc.*, 2018, **140**, 16982–16987.
6 G. A. McNaughton-Smith, J. F. Burns, J. W. Stocker, G. C. Rigdon, C. Creech, S. Arrington, T. Shelton and L. De Franceschi, *J. Med. Chem.*, 2008, **51**, 976–982.
7 X.-F. Li, Z.-G. Chi, B.-J. Xu, H.-Y. Li, X.-Q. Zhang, W. Zhou, Y. Zhang, S.-W. Liu and J.-R. Xu, *J Fluoresc*, 2011, **21**, 1969–1977.
8 W. Fu, L. Dong, J. Shi, B. Tong, Z. Cai, J. Zhi and Y. Dong, *Polym. Chem.*, 2018, **9**, 4404–4412.
9 Y. Li, W. Lu and D. Xue, *New York*.
10 P. Peng, F. Li, V. S. P. K. Neti, A. J. Metta-Magana and L. Echegoyen, *Angew. Chem. Int. Ed.*, 2014, **53**, 160–163.
11 H.-L. Huang, H. Yan, C. Yang and W. Xia, *Chem. Commun.*, 2015, **51**, 4910–4913.
12 X. Liu, F. Xiong, X. Huang, L. Xu, P. Li and X. Wu, *Angew. Chem. Int. Ed.*, 2013, **52**, 6962–6966.
13 D. Rosa and A. Orellana, *Org. Lett.*, 2011, **13**, 3648–3651.
14 E. C. Ashby and J. R. Bowers, *J. Am. Chem. Soc.*, 1981, **103**, 2242–2250.
15 M. A. Said, A. Al-unizi, M. Al-Mamary, S. Alzahrani and D. Lentz, *Inorg. Chim. Acta*, 2020, **505**, 119434.
16 T. Qin, G. Lv, Q. Meng, G. Zhang, T. Xiong and Q. Zhang, *Angew. Chem. Int. Ed.*, 2021, **60**, 25949–25957.
17 L. P. C. Nielsen, C. P. Stevenson, D. G. Blackmond and E. N. Jacobsen, *J. Am. Chem. Soc.*, 2004, **126**, 1360–1362.
18 J. Li and Z.-X. Wang, *Synthesis*, 2018, **50**, 3217–3223.
19 Z. Deng, C. Chen and S. Cui, *RSC Advances*, 2016, **6**, 93753–93755.
20 M. Brown, R. Kumar, J. Rehbein and T. Wirth, *Chem. - Eur. J*, 2016, **22**, 4030–4035.
21 J. S. Sharley, A. M. Collado Pérez, E. E. Ferri, A. F. Miranda and I. R. Baxendale, *Tetrahedron*, 2016, **72**, 2947–2954.
22 Y. Dokai, K. Saito and T. Yamada, *Chem. Commun.*, 2022, **58**, 9500–9503.
23 J. Templ and M. Schnürch, *J. Org. Chem.*, 2022, **87**, 4305–4315.
24 A. D. Benischke, M. Leroux, I. Knoll and P. Knochel, *Org. Lett.*, 2016, **18**, 3626–3629.
25 J. Kim, J. Jang, Y. Lee and K. Shin, *Org. Lett.*, 2022, **24**, 5412–5416.
26 F. Liu, Y.-Y. Hu, D. Li, Q. Zhou and J.-M. Lu, *Tetrahedron*, 2018, **74**, 5683–5690.
27 H. Zhang, J. Rodrigalvarez and R. Martin, *J. Am. Chem. Soc.*, 2023, **145**, 17564–17569.
28 C. H. Cheon, O. Kanno and F. D. Toste, *J. Am. Chem. Soc.*, 2011, **133**, 13248–13251.
29 M. Schedler, D. Wang and F. Glorius, *Angew. Chem. Int. Ed.*, 2013, **52**, 2585–2589.
30 T. Müller, M. Hasenbeck, J. Becker and U. Gellrich, *Z. anorg. allg. Chem*, 2023, **649**, e202200381.
31 M. Wissing and A. Studer, *Chem. Eur. J.*, 2019, **25**, 5870–5874.
32 T. T. Tsou, M. Loots and J. Halpern, *J. Am. Chem. Soc.*, 1982, **104**, 623–624.
33 S. S. Lande and J. K. Kochi, *J. Am. Chem. Soc.*, 1968, **90**, 5196–5207.

34 T. W. Koenig, B. P. Hay and R. G. Finke, *Polyhedron*, 1988, **7**, 1499–1516.
35 E. G. Samsel and J. K. Kochi, *J. Am. Chem. Soc.*, 1986, **108**, 4790–4804.
36 K. Ebisawa, K. Izumi, Y. Ooka, H. Kato, S. Kanazawa, S. Komatsu, E. Nishi and H. Shigehisa, *J. Am. Chem. Soc.*, 2020, **142**, 13481–13490.
37 O. Gutierrez, J. C. Tellis, D. N. Primer, G. A. Molander and M. C. Kozlowski, *J. Am. Chem. Soc.*, 2015, **137**, 4896–4899.
38 C. V. Wilson, D. Kim, A. Sharma, R. X. Hooper, R. Poli, B. M. Hoffman and P. L. Holland, *J. Am. Chem. Soc.*, 2022, **144**, 10361–10367.
39 C. A. Discolo, E. E. Touney and S. V. Pronin, *J. Am. Chem. Soc.*, 2019, **141**, 17527–17532.
